# Confinement-Controlled, Either *syn*- or *anti*-Selective Catalytic Asymmetric Mukaiyama Aldolizations of Propionaldehyde Enolsilanes

**DOI:** 10.1021/jacs.1c07447

**Published:** 2021-08-26

**Authors:** Tynchtyk Amatov, Nobuya Tsuji, Rajat Maji, Lucas Schreyer, Hui Zhou, Markus Leutzsch, Benjamin List

**Affiliations:** † Max-Planck-Institut für Kohlenforschung, Kaiser-Wilhelm-Platz 1, D-45470 Mülheim an der Ruhr, Germany; ‡ Institute for Chemical Reaction Design and Discovery (WPI-ICReDD), Hokkaido University, Sapporo 001-0021, Japan

## Abstract

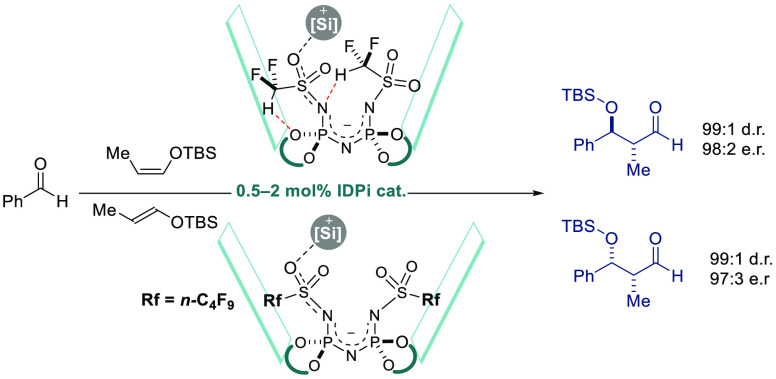

Protected aldols (i.e., true aldols derived from aldehydes) with either *syn*- or *anti*- stereochemistry are versatile intermediates in many oligopropionate syntheses. Traditional stereoselective approaches to such aldols typically require several nonstrategic operations. Here we report two highly enantioselective and diastereoselective catalytic Mukaiyama aldol reactions of the TBS- or TES- enolsilanes of propionaldehyde with aromatic aldehydes. Our reactions directly deliver valuable silyl protected propionaldehyde aldols in a catalyst controlled manner, either as *syn-* or *anti-* isomer. We have identified a privileged IDPi catalyst motif that is tailored for controlling these aldolizations with exceptional selectivities. We demonstrate how a single atom modification in the inner core of the IDPi catalyst, replacing a CF_3_-group with a CF_2_H-group, leads to a dramatic switch in enantiofacial differentiation of the aldehyde. The origin of this remarkable effect was attributed to tightening of the catalytic cavity via unconventional C–H hydrogen bonding of the CF_2_H group.

Polyketides are pharmaceutically important secondary metabolites.^1^ Erythromycin is a prototypical example, which as a synthetic target was declared by Woodward as “hopelessly complex ...in view of its plethora of asymmetric centers”.^2^ This statement encouraged the beginning of several decades of intense and highly innovative method development in acyclic stereocontrol. Generations of chemists have contributed approaches to overcoming the synthetic challenges posed by oligopropionates, typically bearing linear stereopolyads with alternating methyl and hydroxyl groups.^3^ Nonetheless, only few truly reliable methods have found general utility in numerous syntheses of complex oligopropionates.^4^ Widely used approaches, based on chiral auxiliaries, rely on diastereoselective asymmetric propionate aldolizations or crotylation reactions (Figure 1).^5,6^ Very often, both approaches converge after several steps: following the critical diastereoselective C–C bond-formation, protecting group installation and redox manipulations lead to stable protected aldol intermediates of a general structure **I**, which are ideal for downstream functionalization to construct various polyketide motifs. Catalytic asymmetric crotylation methods developed more recently by Krische et al. provide an attractive alternative but feature a moderate step-economy when protected aldols of type **I** are needed.^7^ Direct stereoselective cross-aldol reactions of aldehydes have also been described, but an additional protection step is often unavoidable.^8^ A truly practical approach, from a total synthesis chemist’s perspective, would directly deliver the protected aldols in a predictable catalytic manner, with full control over diastereoselectivity and enantioselectivity. In this regard, arguably the most useful variant of the Mukaiyama aldol reaction,^9^ the aldolization of propionaldehyde-derived enolsilanes with aldehydes, has remained elusive. In addition to directly delivering the protected aldols, such a reaction would favorably meet all of the metrics used to evaluate synthetic methods, such as atom-,^10^ redox-,^11^ and especially step-economy.^12^

**Figure 1:**
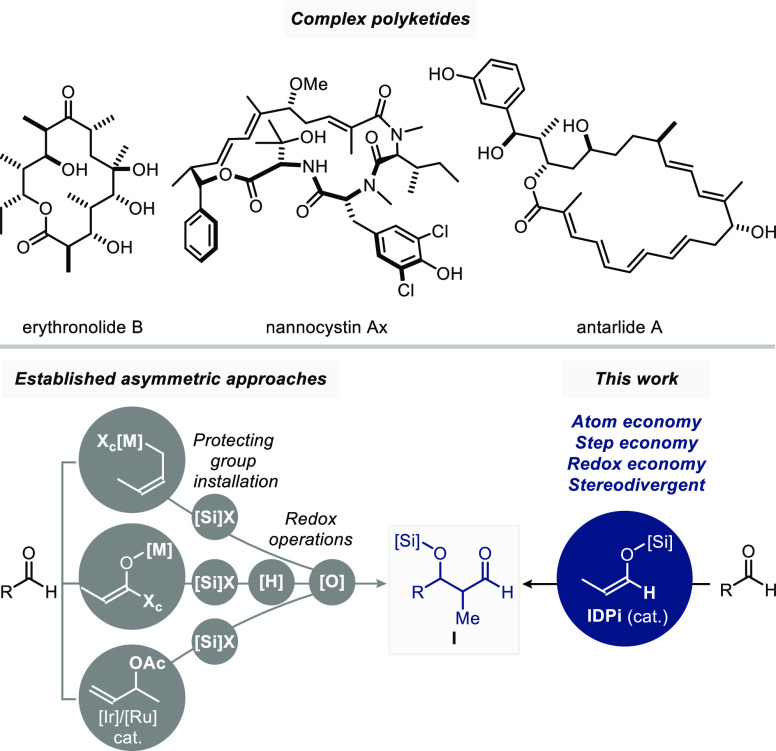
Complex bioactive polyketides. Established approaches to protected aldols **I** vs our single-step, stereodivergent method.

Yamamoto reported nonenantioselective examples using the bulky tris(trimethylsilyl)silyl (TTMSS, supersilyl) group to control selectivity toward single addition of aldehyde-derived enolsilanes.^13^ The first diastereo- and enantioselective examples by Denmark utilized trichlorosilyl enolsilanes under Lewis base catalysis.^14^ This approach is poorly atom-economic, as the silyl group is not retained in the final product and several steps are required to obtain aldols suitable for chain elongations. Kanai and Matsunaga reported an asymmetric Cu-catalyzed aldolization using *in situ* generated boron enolates of propionaldehyde, which gives mainly *syn*-aldols.^15^ Although up to quadruple aldolizations were achieved, the existence of unprotected oligoaldols in various cyclic hemiacetal forms limits their selective elaboration to useful oligopropionate motifs. Recently we reported the first, highly enantioselective Mukaiyama cross-aldol reaction with simple triethylsilyl (TES) and *tert*-butyldimethylsilyl (TBS) enolsilanes of acetaldehyde and aliphatic and aromatic aldehydes using confined and strongly acidic imidodiphosphorimidate (IDPi) catalysts developed here.^16^ Enzyme-like discrimination of the small substrate aldehyde over the larger product aldehyde is believed to be the origin of single aldolization without oligomerization.^17^ From such a vantage point, exploitation of the unique reactivity of IDPi catalysts in the Mukaiyama cross-aldol reaction with propionaldehyde-derived enolsilanes would be even more valuable as it generates two (vicinal) stereogenic centers simultaneously, which are present in many bioactive polyketides (Figure 1).^18^ We envisioned developing a fully stereodivergent method giving access to all four stereoisomers.^19^ Herein, we report that the IDPi family of catalysts provides a powerful solution to this long-standing goal.^20^

Our investigations commenced with an exploration of different IDPi catalysts in the aldolization of benzaldehyde **1** with (*E*)-enolsilanes **2a**–**b** or (*Z*)-enolsilanes **4a**–**c** (Table 1). At the onset we found that the diastereoselectivity was dependent on the nucleophile geometry, with (*E*)-enolsilanes providing *syn*-aldols and (*Z*)-enolsilanes giving *anti*-aldols, with varying degrees of diastereoselectivity, depending on the silyl group and the catalyst. IDPi **6**, which was a preferred catalyst in our acetaldehyde-derived enolsilane additions, was tested in the reaction of (*E*)-enolsilane **2a** with benzaldehyde at −20 °C. Single aldolization product **3** was indeed formed in high yield, excellent diastereoselectivity (d.r. 97.5:2.5) in favor of the *syn*-aldol **3a** ([Si] = TES), and with a promising enantiomeric ratio (e.r.) of 10.5:89.5 (Table 1A, entry 1). Given that fluorene-substituted IDPi catalysts were especially privileged in our recent silylium-ion asymmetric counteranion-directed catalysis (*Si*-ACDC)^21^ methodologies,^22a−22d^ we turned our attention to catalysts **7a**–**d** (and **S8a–c** in the Supporting Information). Indeed, these IDPis emerged as preferred catalysts for our reactions. Spirocyclobutane-substituted IDPi **7a** markedly stood out in terms of enantioselectivity, providing aldol **3a** with a 95.5:4.5 e.r. (Table 1A, entry 2). Modification of the inner core of the IDPi from the Tf-group to a Nf-group further increased diastereoselectivity and enantioselectivity. Lowering the temperature to −40 °C led to a d.r. of 99:1 and a 98:2 e.r. (Table 1A, entry 5). Variation of the silyl group was well-tolerated when the TBS-enolsilane **2b** was used instead of the TES-enolsilane **2a** (Table 1A, entry 6). An opposite trend was observed in the *anti*-selective Mukaiyama aldol addition of (*Z*)-enolsilanes (Table 1B). Both the size of the perfluorinated sulfonamide in the inner core of IDPi and the silyl group had tremendous effects on the selectivity. Longer perfluorinated groups gave poorer d.r. and e.r. At −60 °C, the addition of (*Z*)-enolsilane **4a** ([Si] = TES) to benzaldehyde proceeded with modest d.r. and e.r. using catalysts **7b** and **7c**, which performed exceptionally well previously in the *syn*-aldolization (Table 1B, entries 4–5). In contrast, IDPi **7a** with the shortest trifluoromethylsulfonamide core performed far better, but to our surprise, with inverted *benzaldehyde* enantiofacial preference. Among (*Z*)-enolsilanes **4a**–**4c** with different silyl-groups (Table 1B, entries 1–3), enolsilane **4b** with the TBS-group performed best, albeit only in 89:11 e.r. Hypothesizing that replacing one of the F atoms in the CF_3_-group to an H atom may help in positively influencing the selectivity without introducing significant steric changes,^23^ IDPi **7d** containing a CF_2_H-group was designed and synthesized. Specifically, we envisioned that CF_2_H···heteroatom interactions could lead to a modulation of the active site of the IDPi catalyst.^24^ Strikingly, the single-atom modification of the inner core of the catalyst, replacing the CF_3_-groups with CF_2_H-groups, indeed resulted in a spectacular enhancement of both the d.r. (99:1) and e.r. (98:2) in the addition of enolsilane **4b** (Table 1B, entries 7 and 8).

**Table 1:**
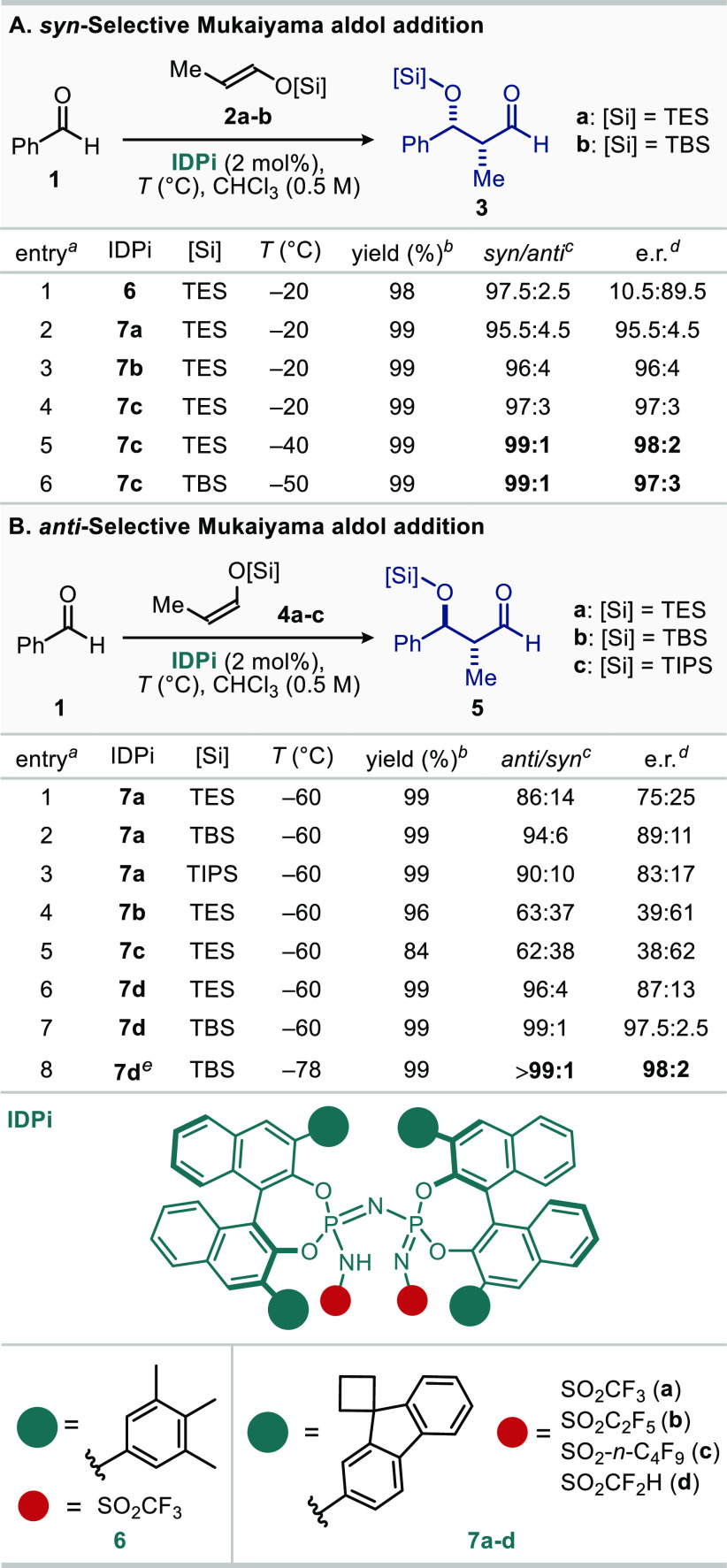
Reaction Development

a Reactions were conducted with benzaldehyde **1** (0.1 mmol), enolsilanes **2a**–**b** or **4a**–**c** (1.2 equiv), and IDPi (2 mol %) for 16–24 h at the indicated temperature.

b Determined by ^1^H NMR spectroscopy.

c Determined by crude ^1^H NMR analysis.

d The e.r. was determined by HPLC.

e At −78 °C using a 5:4 CHCl_3_/*n*-hexane mixture. TES, triethylsilyl; TIPS, triisopropylsilyl; TBS, *tert*-butyldimethylsilyl. See the Supporting Information for determination of the absolute configuration.

With these results in hand, the scope of our *syn*-selective Mukaiyama aldol reaction with various aromatic aldehydes was explored, using catalyst **7c** (Table 2A). Aromatic aldehydes with *o*-, *m*-, and *p*-substituents and heteroaromatic aldehydes (**3a**–**3j**) gave the corresponding products in excellent yields and stereoselectivity. Multisubstituted aromatic aldehydes provided products **3k**–**3m**, which contain substructures of complex polyketides.^25,26^ The catalyst loading could be reduced to 0.5 mol % without compromising the reaction time and stereoselectivity as shown with the gram scale synthesis of aldols **3h** and **3k**. A diastereoselective and enantioselective single aldolization of a dialdehyde substrate gave product **3m**.

**Table 2:**
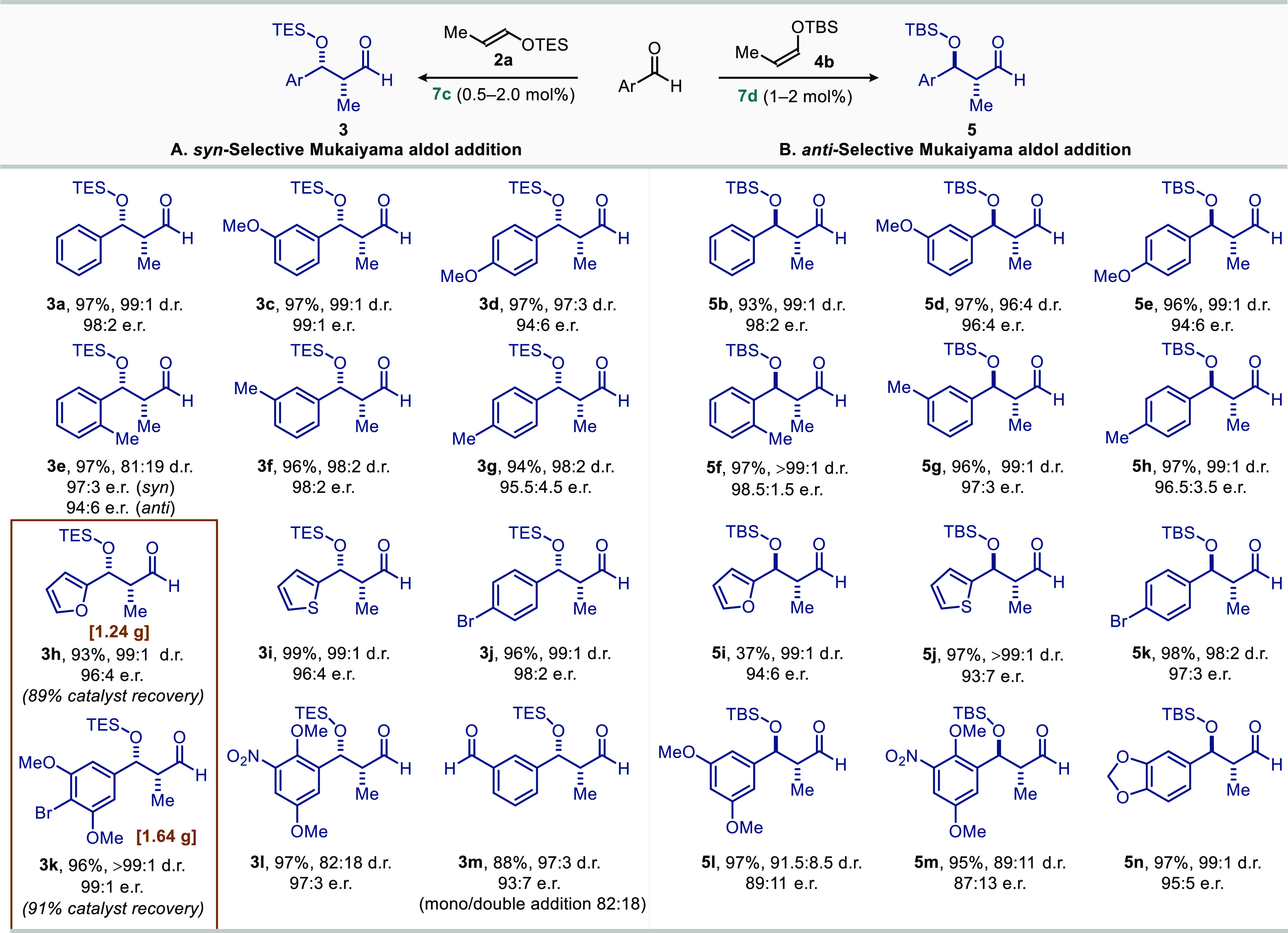
Substrate Scope for the *syn*- and *anti*-Mukaiyama Aldol Additions^a^

a Reaction scale: 0.2–5 mmol. See the Supporting Information for full reaction conditions and the determination of e.r.

Essentially the same set of aromatic aldehydes performed equally well in our *anti*-aldolization process furnishing products **5b**–**k** and **5m** (Table 2B). Electron-rich aromatic aldehydes also delivered *anti*-aldols **5l** and **5n** with good diastereoselectivity and enantioselectivity. Only furfural gave somewhat lower yield of product **5i**.

Furthermore, butyraldehyde-derived enolsilanes **8** and **9** also readily reacted to either *syn*- or *anti*-products **10** and **11** (Scheme 1A). Moreover, both enantiomers of our IDPi catalysts enabled access to all four possible stereoisomers of aldols as shown in Scheme 1B.

**Scheme 1:**
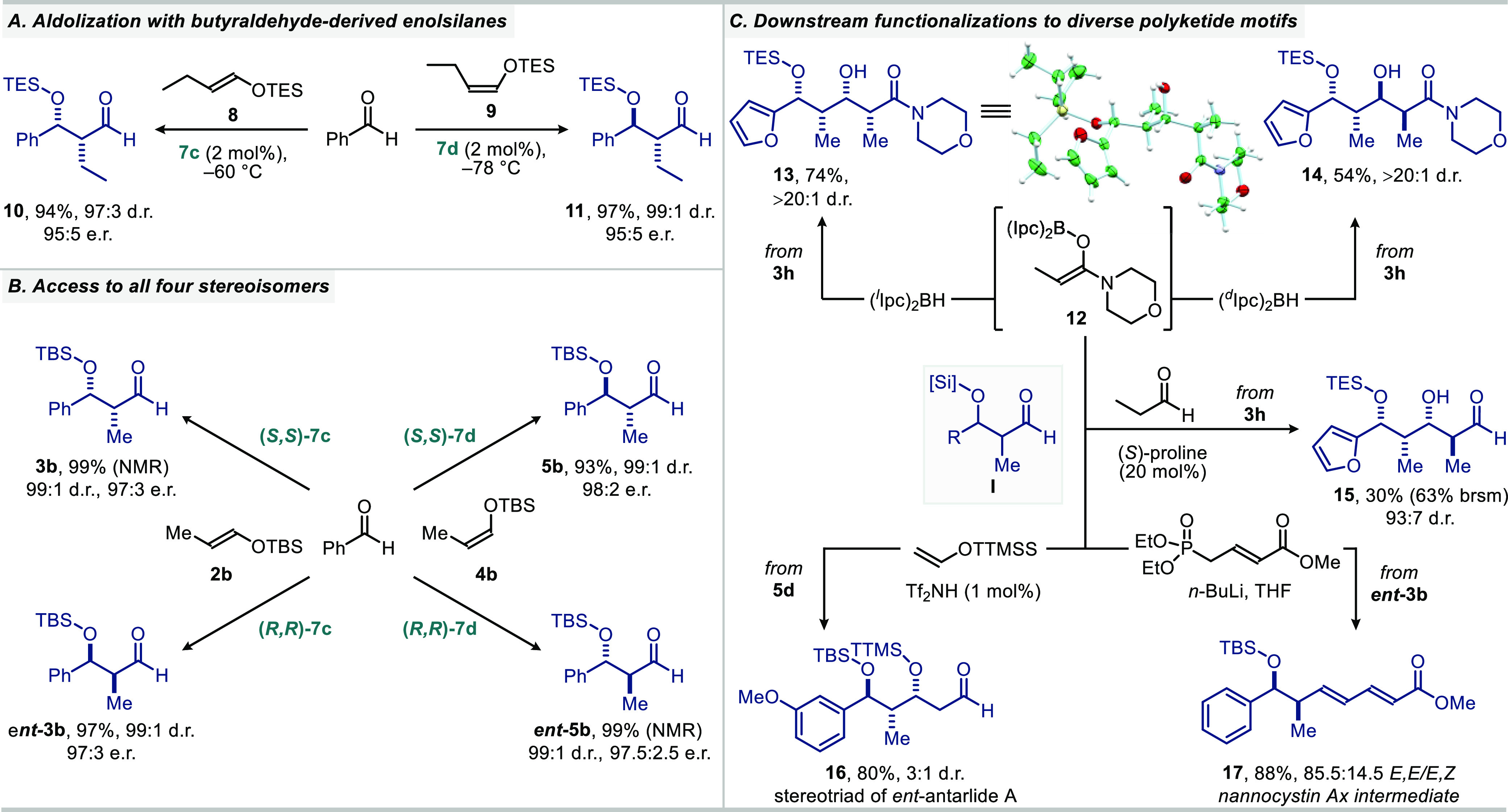
Further Applications of the Diastereoselective and Enantioselective Aldol Reactions

Our stereodivergent aldolization method is especially valuable in the context of the rapid generation of complex polyketide motifs (Scheme 1C). For example, when *syn*-aldol **3h** was subjected to a follow-up propionate aldolization using *in situ* generated chiral boron enolates **12**,^27^ either the all-*syn*-stereotetrad **13** or its *syn*, *anti*, *syn* stereoisomer **14** were obtained as single diastereomers. The structure of the polyketide-like molecule **13** was unambiguously confirmed by X-ray crystallography. Alternatively, iterative aldolization using the proline-catalyzed cross-aldol addition with propionaldehyde delivered the *syn*, *syn*, *anti*-stereotetrad **15** with excellent diastereoselectivity. Further, *anti*-aldol **5d** was converted to a fully protected double aldol adduct **16**, containing the key *anti*, *syn*-stereotriad of antarlide A,^28^ when our method was coupled with Yamamoto’s supersilyl enolate technology. Stereotriad **16** is fully suited for further aldol addition toward antarlides. 1,3-Dienyl-6-oxy polyketide motif **17**, an intermediate from a reported total synthesis of nannocystin Ax, was also obtained in only two steps starting from benzaldehyde (via *ent***-3b**). The previous synthesis of **17** involved five steps, including an Evans-aldolization.^29^ It is noteworthy that all of the polyketide motives **13**–**17** were obtained in just two steps from commercially available aldehydes such as furfural, *m*-anisaldehyde, and benzaldehyde.

To gain insight into the mechanism of our stereoselective aldol additions, experimental and computational studies were performed. Experiments were directed at probing aspects of our aldol reactions such as the origin of single aldolization and the unexpected change of facial selectivity during *anti*-aldolization using IDPi **7d**. Initially, we confirmed that our IDPi catalysts were indeed unique in promoting the single aldolization of propionaldehyde enolsilanes: the well-established Mukaiyama aldol addition catalyst triflimide did not give even a trace of the desired single aldolization products because of complete enolsilane oligomerization (Figure 2A, see the Supporting Information, Figure S2 for details). *syn*-Aldol **3c**, which was obtained using IDPi (*S,S*)-**7c** at −40 °C, underwent less than 10% conversion under the same conditions using the opposite enantiomer of the catalyst (*R*,*R*)-**7c**, excluding a potential matched–mismatched scenario (Figure 2B). The change in facial selectivity of aldehyde attack upon switching from the *n*-C_4_F_9_- and CF_3_-groups to the CF_2_H-group was also manifested when simple acetaldehyde-derived enolsilanes **19a**-**b** were used (Figure 2C). With catalysts **7a** and **7c**, acetaldehyde-derived enolsilane additions to benzaldehyde proceeded with *re*-selectivity, giving *ent***-20** irrespective of the silyl groups. In contrast, IDPi **7d**, having a difluoromethanesulfonyl group in the core, reacted with *si*-selectivity.

**Figure 2:**
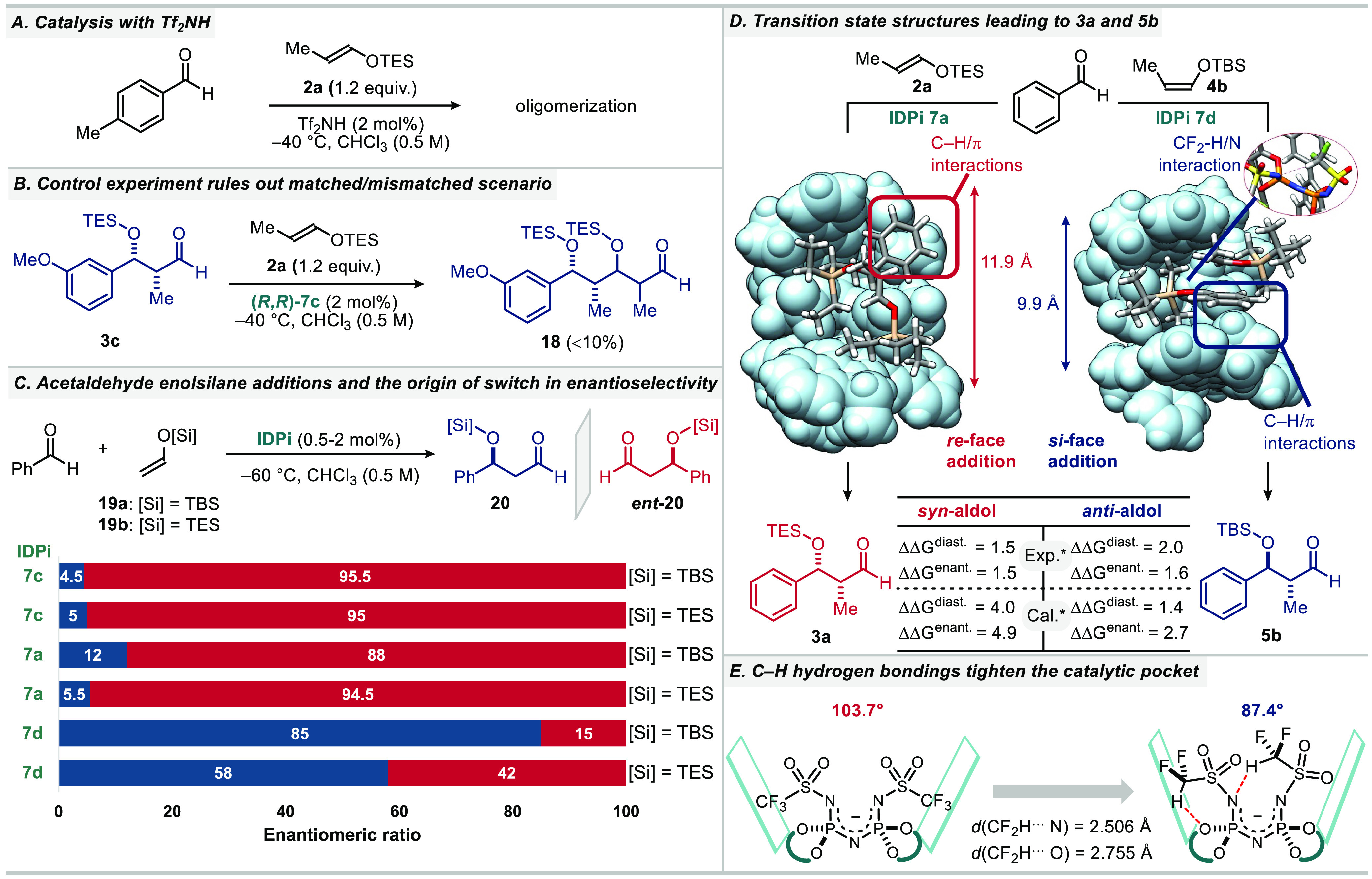
Mechanistic studies. (A–C) Control experiments. (D) Computed transition-state structures of major enantiomer of **3a** (*syn*-selective addition) with **7a** (left) and **5b** (*anti*-selective addition) with **7d** (right) at B3LYP-D3(BJ)/def2TZVP+CPCM(Chloroform)//ONIOM(PBE-D3/6-31G(d):PBE-D3/3-21G) level of theory. Distances between centroids of the two inner spirocyclobutyl-2-fluorenyl groups are shown. *Energies in kcal/mol (see the Supporting Information for details). (E) Effect of C–H hydrogen bondings on the cavity size. Angles represent centroids of the two inner spirocyclobutyl-2-fluorenyl groups and the central nitrogen.

In order to probe the origin of stereoselectivity and the switch of enantiofacial selectivity, an extensive DFT study was conducted for both *syn*- and *anti*-selective additions with IDPis **7a** and **7d**. Computed e.r.s and d.r.s were in good agreement with experimental observations in both cases (Figure 2D; see the Supporting Information, Figures S6–S10 for more details). When the optimized major transition-state structures of *syn*- and *anti*-selective aldolizations are compared, one of the most prominent differences appears to be the catalyst pocket size.^30^ While catalyst **7a** with CF_3_-cores has a relatively open cavity, the CF_2_H-groups in catalyst **7d** engage in intramolecular hydrogen bonding interactions resulting in a more compact catalytic pocket (Figure 2D and 2E). Accordingly, for the *syn*-selective addition with **7a**, the bulky (*E*)-enolsilane **2a** bearing a smaller TES group would approach from the less hindered *re*-face (Figure 2B, left). In contrast, for the case of *anti*-selective addition with **7d**, the sterically less hindered (*Z*)-enolsilane **4b**, with a slightly bulkier TBS group, provides a perfect fit into the narrower cavity, resulting in the complete switch of the facial selectivity (Figure 2D, right). The outcome of the acetaldehyde-derived enolsilane additions using catalysts **7a** and **7d** is also in good agreement with this model (Figures 2C, S12, and S13).

Additionally, our study has identified the involvement of CH/π interactions, indicated in Figure 2D,^31^ between the spirocyclic methylene groups of the catalyst counteranion and the aromatic ring of the aldehyde substrate, which contribute to the high enantioselectivities in both transition states.^32^ This is in agreement with our experimental observations, showing a strong effect of the spirocycle on the enantioselectivity (Figure S1).

Our highly stereoselective Mukaiyama aldol additions of propionaldehyde enolsilanes give access to all stereoisomers of the stable and versatile protected aldols in a predictable manner and can be used in rapid syntheses of complex polyketide motifs. Ultimately, our approach could aid in streamlining the synthesis of complex oligopropionates. We also uncovered an unusual enantioreversal effect by modifying a CF_3_-group to a CF_2_H-group. The origin of stereoselectivities and enantiofacial switch was rationalized through computational studies, revealing the cooperation of C–H hydrogen bonds and CH/π interactions to govern catalyst structure and transition states.

## Supporting Information Available

Experimental details and analytical data for all new compounds (PDF)

## Accession Codes

CCDC 2097850–2097853 contain the supplementary crystallographic data for this paper. These data can be obtained free of charge via www.ccdc.cam.ac.uk/data_request/cif, or by emailing data_request@ccdc.cam.ac.uk, or by contacting The Cambridge Crystallographic Data Centre, 12 Union Road, Cambridge CB2 1EZ, UK; fax: +44 1223 336033.
