# Supplementary material for: Confinement-Controlled, Either syn- or anti-Selective Catalytic Asymmetric Mukaiyama Aldolizations of Propionaldehyde Enolsilanes
Source: J Am Chem Soc. 2021 Aug 26;143(36):14475–81. doi: 10.1021/jacs.1c07447 (PMC8447262; doi:10.1021/jacs.1c07447)
Supplement: Supplementary file 1 — ja1c07447_si_001.pdf [file ja1c07447_si_001.pdf]

Confinement-Controlled, Either *syn*- or *anti*-Selective Catalytic Asymmetric Mukaiyama Aldolizations of Propionaldehyde Enolsilanes

Tynchtyk Amatov[1], Nobuya Tsuji[2], Rajat Maji[1], Lucas Schreyer[1], Hui Zhou[1], Markus Leutzsch[1] and Benjamin List[1,2]*.

[1]Max-Planck-Institut für Kohlenforschung, Kaiser-Wilhelm-Platz 1, D-45470 Mülheim an der Ruhr, Germany.

[2]Institute for Chemical Reaction Design and Discovery (WPI-ICReDD), Hokkaido University, Sapporo 001-0021, Japan

*Email: list@kofo.mpg.de

Contents

General information	S2
General reaction procedures	S4
syn-Aldols and their derivatives	S5
anti-Aldols and their derivatives	S17
Catalyst synthesis	S29
Synthesis of enolsilanes	S31
Reaction investigations	S34
Mechanistic investigations	S36
Determination of the absolute configuration	S43
Crystallographic data	S44
Copies of NMR spectra	S54
HPLC traces	S115
Computational studies	S130
References	S245

General information

Unless otherwise stated, all reactions were magnetically stirred and conducted in oven-dried (90 °C) or flame-dried glassware in anhydrous solvents under Ar, applying standard Schlenk techniques. Solvents and liquid reagents, as well as solutions of solid or liquid reagents were added via syringes, stainless steel or polyethylene cannulas through rubber septa or through a weak Ar counter-flow. Solid reagents were added through a weak Ar counter-flow. Cooling baths were prepared in Dewar vessels, filled with ice/water (0 °C), cooled acetone (> −78 °C) or dry ice/acetone (−78 °C). Heated oil baths were used for reactions requiring elevated temperatures. Solvents were removed under reduced pressure at 40 °C using a rotary evaporator, and unless otherwise stated, the remaining compound was dried in high vacuum (10–3 mbar) at ambient temperature. All given yields are isolated yields of chromatographically and NMR spectroscopically pure materials, unless otherwise stated.

Chemicals

Chemicals were purchased from commercial suppliers (including abcr, Acros, Alfa Aesar, Fluorochem, Merck, and TCI) and used without further purification unless otherwise stated. Et_3N was distilled from $LiAlH_4$ and stored under argon prior to use. Pyridine was dried and stored over molecular sieves. All used aldehydes were purified by vacuum distillation and stored under Ar at 5 °C. Solid aldehydes were dried under high vacuum overnight before using. Chlorotriethylsilane, triethylsilyl trifluoromethanesulfonate and *tert*-butylchlorodimethylsilane were used as received from commercial suppliers. Previously reported compounds were synthesized from commercially available starting materials according to literature procedures: P(NTf)Cl_3,[1] P($NSO_2{}^nC_4F_9$)Cl_3,[2] IDPi **6**,[3] diol **S7**,[2] IDPis **7a-c** and **S5a-c**.[2]

Solvents

Solvents (CH_2Cl_2, Et_2O, THF, toluene) were dried by distillation from an appropriate drying agent in the technical department of the Max-Planck-Institut für Kohlenforschung and received in Schlenk flasks under argon. Other anhydrous solvents (toluene, $CHCl_3$, 1,4-dioxane, DMSO, EtOAc, EtOH, MeCN, MeOH, MTBE, and *n*-hexane and *n*-pentane) were purchased from commercial suppliers and dried over molecular sieves. *For aldol reaction, dry $CHCl_3$ was filtered via HPLC-filter to exclude any particles from the drying agent.*

Inert Gas

Dry argon was purchased from Air Liquide with >99.5% purity.

Thin Layer Chromatography

Thin-layer chromatography (TLC) was performed using silica gel pre-coated glass plates (SIL G-25, with fluorescent indicator UV254; Macherey-Nagel) and aluminium oxide pre-coated plastic sheets (Polygram Alox N, 0.2 mm, with fluorescent indicator UV254; Macherey-Nagel), which were visualized by irradiation with UV light (λ = 254 or 366 nm), basic $KMnO_4$, and/or phosphomolybdic acid (PMA). PMA stain: PMA (20 g) in EtOH (200 mL), $KMnO_4$ stain: aq NaOH (10 wt%, 1.25 mL), $KMnO_4$ (1.5 g), K_2CO_3 (10 g) in H_2O (200 mL),. Preparative thin-layer chromatography was performed on silica gel pre-coated glass plates SIL G-100, with fluorescent indicator UV254 (Macherey-Nagel).

Column Chromatography

Column chromatography was carried out using Merck silica gel (60 Å, 230−400 mesh, particle size 0.040−0.063 mm) or aluminum oxide (neutral, activated, Brockmann I, Sigma-Aldrich; activity adjustment individually specified) using technical grade solvents. Elution was accelerated using compressed air. All fractions containing a desired substance were combined and concentrated in vacuo, then redissolved in an appropriate solvent and filtered through cotton plug to remove silica residues.

Nomenclature

Nomenclature follows the suggestions proposed by the computer program ChemDraw Professional 15.0 of PerkinElmer®.

Melting Points

Melting points (m.p.) were measured on a Büchi 540 melting point apparatus in open glass capillaries and are uncorrected.

Nuclear Magnetic Resonance Spectroscopy

^1H, ^{13}C, ^{19}F, ^{31}P nuclear magnetic resonance (NMR) spectra were recorded on a Bruker AVIII 500 MHz, Bruker AVIII HD 300 MHz or Bruker AVNeo 600 MHz spectrometer in a suitable deuterated solvent. The solvent employed and respective measuring frequency are indicated for each experiment. Chemical shifts are reported with Me_4Si

serving as a universal reference of all nuclides and with two or one digits after the comma. The resonance multiplicity is described as s (singlet), d (doublet), t (triplet), q (quadruplet), p (pentet), hept (heptet), m (multiplet), and br. (broad). All spectra were recorded at 298 K unless otherwise noted, processed with the program MestReNova 14.1.2, and coupling constants are reported as observed. The residual deuterated solvent signal relative to tetramethylsilane (TMS) was used as the internal reference in ^{1}H NMR spectra ($CDCl_3$ δ 7.26, CD_2Cl_2 δ 5.32), and are reported as follows: chemical shift δ in ppm (multiplicity, coupling constant J in Hz, number of protons). ^{13}C NMR spectra reported in ppm from tetramethylsilane (TMS) with the solvent resonance as the internal standard ($CDCl_3$ δ 77.16, CD_2Cl_2 δ 53.8). ^{19}F, ^{31}P and ^{29}Si NMR spectra are reported relative to CCl_3F (δ (^{19}F) = 0 ppm), H_3PO_4 (δ (^{31}P) = 0 ppm) and TMS (δ (^{29}Si) = 0 ppm), respectively. All spectra are broadband decoupled unless otherwise noted

Mass Spectrometry

Electron impact (EI) mass spectrometry (MS) was performed on a Finnigan MAT 8200 (70 eV) or MAT 8400 (70 eV) spectrometer. Electrospray ionization (ESI) mass spectrometry was conducted on a Bruker ESQ 3000 spectrometer. High resolution mass spectrometry (HRMS) was performed on a Finnigan MAT 95 (EI) or Bruker APEX III FTMS (7T magnet, ESI). The ionization method and mode of detection employed is indicated for the respective experiment and all masses are reported in atomic units per elementary charge (m/z) with an intensity normalized to the most intense peak.

Specific Rotations

Specific rotations $[\alpha]_D^T$ were measured with a Rudolph RA Autopol IV Automatic Polarimeter at the indicated temperature (T) with a sodium lamp (sodium D line, λ = 589 nm). Measurements were performed in an acid resistant 1 mL cell (50 mm length) with concentrations (g/(100 mL)) reported in the corresponding solvent.

High-Performance Liquid Chromatography

High-performance liquid chromatography (HPLC) was performed on Shimadzu LC-20AD liquid chromatograph (SIL-20AC auto sampler, CMB-20A communication bus module, DGU-20A5 degasser, CTO-20AC column oven, SPD-M20A diode array detector), Shimadzu LC-20AB liquid chromatograph (SIL-20ACHT auto sampler, DGU-20A5 degasser, CTO-20AC column oven, SPD-M20A diode array detector), or Shimadzu LC-20AB liquid chromatograph (reversed phase, SIL-20ACHT auto sampler, CTO-20AC column oven, SPD-M20A diode array detector) using Daicel columns with chiral stationary phases. All solvents used were HPLC-grade solvents, purchased from Merck. The column employed and respective solvent mixture are indicated for each experiment.

Gas Chromatography

Gas chromatography (GC) analyses on a chiral stationary phase were performed on HP 6890 and 5890 series instruments (split-mode capillary injection system, flame ionization detector (FID), hydrogen carrier gas). All of these analyses were conducted in the GC department of the Max-Planck-Institut für Kohlenforschung. The conditions employed are described in detail for the individual experiments. Liquid Chromatography-Mass Spectrometry Liquid chromatography-mass spectrometry (LC-MS) was performed on Shimadzu LC-MS 2020 liquid chromatograph. All solvents used were HPLC-grade solvents purchased from Sigma-Aldrich. The column employed, the respective solvent mixture, and the MS parameters are indicated for each experiment.

Abbreviations

e.r. = enantiomeric ratio, d.r. = diastereomeric ratio, ee = enantiomeric excess, TLC = thin layer chromatography, THF = tetrahydrofuran, MTBE = methyl *tert*-butyl ether, TES = triethylsilyl, TBS = $SiMe_2{}^tBu$, TTMSS = tris(trimethylsilyl)silyl, Tf = CF_3SO_2, Nf = n-$C_4F_9SO_2$.

General reaction procedures

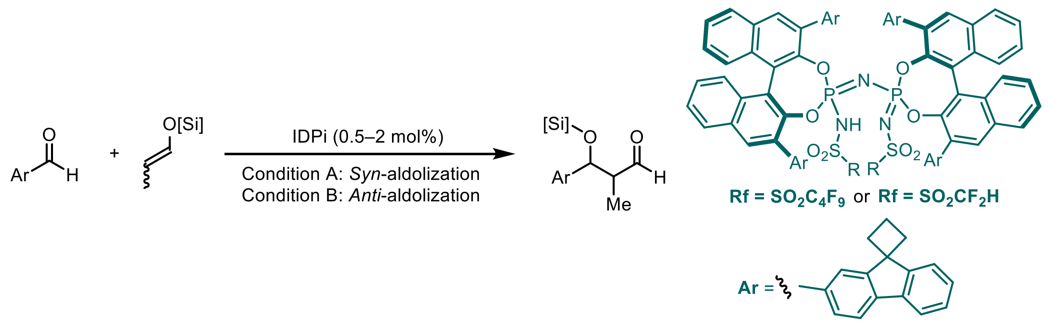

General procedure for syn-selective Mukaiyama aldol reaction*

An oven-dried 2 mL vial was charged with IDPi **7c** (4.0 µmol, 8.2 mg, 2 mol%), a magnetic stir bar and dry $CHCl_3$ (0.4 mL) and the corresponding aldehyde (0.2 mmol, 1.0 equiv.) was added to this reaction. The vial was sealed with a septum cap and cooled to -40 °C in a cryostat for 15 min. (*E*)-enolsilane of propanal **2a** (0.24 mmol, 50 µL, 1.2 equiv.) was added slowly via Hamilton syringe. The reaction mixture was stirred at -40 °C for 16-20 h and quenched with Et_3N (0.02 mmol, 2.8 µL, 0.1 equiv.). An aliquot of a crude reaction mixture was dissolved in $CDCl_3$ for determination of the d.r. The reaction mixture was evaporated and the residue was purified by column chromatography to afford pure aldol product. **For deviations from the general procedure (for example catalyst loading, different scale or temperature) see the corresponding entries.*

General procedure for anti-selective Mukaiyama aldol reaction*

An-oven dried 2 mL vial was charged with IDPi **7d** (4.0 µmol, 6.9 mg, 2 mol%), a magnetic stir bar and a 5:4 $CHCl_3$/*n*-hexane mixture (0.4 mL) and the corresponding aldehyde (0.2 mmol, 1.0 equiv.) was added to this reaction. The vial was flushed with Ar and sealed with a septum cap and cooled to -78 °C in a cryostat for 15 min. (*Z*)-Enolsilane of propanal **4c** (0.24 mmol, 50 µL, 1.2 equiv.) was added slowly via Hamilton syringe. The reaction mixture was stirred at -78 °C for 16-20 h and quenched with Et_3N (0.02 mmol, 2.8 µL, 0.1 equiv.). An aliquot of a crude reaction mixture was dissolved in $CDCl_3$ for determination of the d.r. The reaction mixture was evaporated and the residue was purified by column chromatography to afford pure aldol product. **For deviations from the general procedure (for example catalyst loading, different scale or temperature) see the corresponding entries.*

Derivatization of aldol products to 1,3-diols:

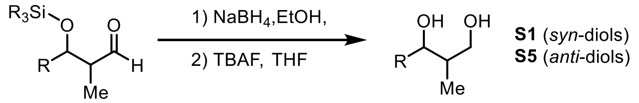

To a solution of aldol (0.1 mmol, 1 equiv.) in ethanol (2 mL) was added $NaBH_4$ (0.15 mmol, 6 mg, 1.5 equiv.) at r.t. and the resulting suspension was stirred for 1 h. The solvent was evaporated and the residue was suspended in a 1:1 CH_2Cl_2/AcOEt mixture and filtered through a short silica pad. The silica pad was washed several times with 1:1 CH_2Cl_2/AcOEt and the combined filtrate was evaporated to dryness. The resulting oil was dissolved in THF (2 mL) under Ar and TBAF (1.0 M in THF, 0.12 mmol, 120 µL, 1.2 equiv.) was added at r.t. under Ar. Full desilylation was achieved typically in 30 – 60 min (TLC with 100% CH_2Cl_2). The solvent was evaporated and the residue was purified by column chromatography (silica, hexanes/AcOEt) to afford the respective 1,3-diol.

Synthesis of racemates:

Two methods were used for the synthesis of racemic 1,3-diols for determination of enantiomeric excess by HPLC analysis. In method A, nonselective aldol reactions using the racemic IDPi **S2** and *E/Z* mixture of enolsilanes was used, which was followed by derivatization to the corresponding racemic 1,3-diols. In method B, organometallic reagent addition to the known racemic aldol **S3**, followed by desilylation provided the required racemic 1,3-diols.

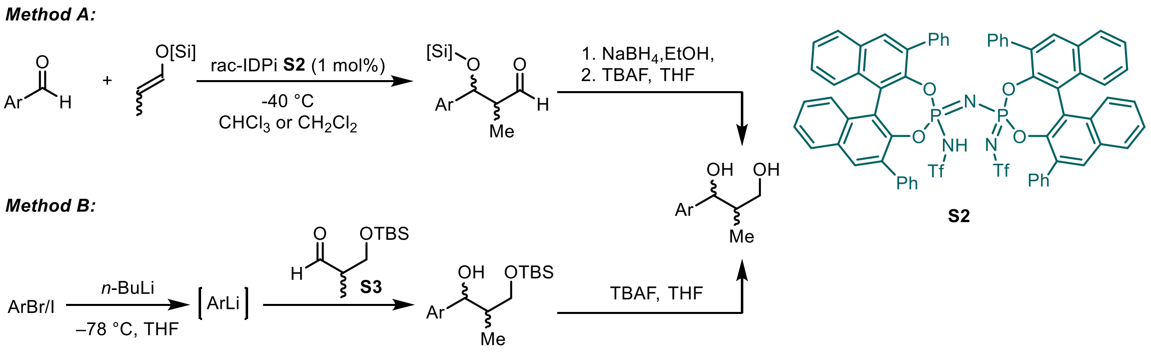

syn-Aldols and their derivatives

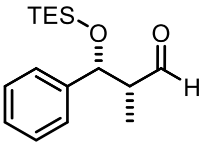

(2*R*,3*R*)-2-Methyl-3-phenyl-3-((triethylsilyl)oxy)propanal (3a):

Benzaldehyde (20 µL, 0.2 mmol), IDPi **7c** (8.11 mg, 4 µmol, 2 mol%) and (*E*)-TES enolate **2a** (50 µL, 0.24 mmol, 1.2 equiv.) were subjected to the general aldol procedure (Conditions A). Purification by column chromatography with hexanes/CH_2Cl_2 (4:1 gradient to 2:1) afforded aldol **3a** (54 mg, 97%) as a colorless oil.

^{1}H NMR (501 MHz, $CDCl_3$) δ 9.77 (d, *J* = 1.3 Hz, 1H), 7.20 – 7.40 (m, 5H), 5.14 (d, *J* = 4.5 Hz, 1H), 2.60 (qdd, *J* = 6.9, 4.4, 1.3 Hz, 1H), 1.04 (d, *J* = 6.9 Hz, 3H), 0.86 (t, *J* = 7.9 Hz, 9H), 0.52 (q, *J* = 8.2 Hz, 6H).

^{13}C NMR (126 MHz, $CDCl_3$) δ 204.5, 142.6, 128.3, 127.6, 126.3, 74.4, 54.89, 8.3, 6.8, 4.9.

ESI-HRMS: calculated for $C_{16}H_{26}O_2SiNa^+$ ($[M+Na]^+$): 301.1594, found: 301.1592.

$[\alpha]_D^{25}$ = +23.7 (*c* = 0.75, $CHCl_3$).

R_f = 0.3 (hexanes/CH_2Cl_2 3:2).

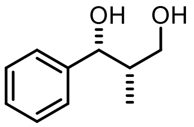

S1a

(1*R*,2*S*)-2-Methyl-1-phenylpropane-1,3-diol (S1a):

Aldol **3a** (15 mg, 0.054 mmol) was subjected to the general procedure for the derivatization of aldols **3** to diols **S1**. Purification by column chromatography with hexanes/AcOEt (3:1 gradient to 1:1) afforded diol **S1a** (8.5 mg, 95%) as a colorless oil.

^{1}H NMR (501 MHz, $CDCl_3$) δ 7.38 – 7.31 (m, 4H), 7.25 – 7.29 (m, 1H), 4.94 (d, *J* = 3.9 Hz, 1H), 3.72 – 3.64 (m, 2H), 3.09 (br. s, 1H), 2.61 (br. s, 1H), 2.05 – 2.12 (m, 1H), 0.85 (d, *J* = 7.1 Hz, 3H).

^{13}C NMR (126 MHz, $CDCl_3$) δ 142.8, 128.3, 127.4, 126.3, 76.9, 66.6, 41.6, 10.9.

ESI-HRMS: calculated for $C_{10}H_{14}NaO_2^+$ ($[M+Na]^+$): 189.0886, found: 189.0887.

$[\alpha]_D^{25}$ = +42.4 (*c* = 0.75, $CHCl_3$). *Lit.* (#): $[\alpha]_D^{27}$ +56.1 (*c* = 0.75, $CHCl_3$)

R_f = 0.20 (hexanes/AcOEt 1:1).

HPLC (IC-3, *n*-heptane/*i*-PrOH=95:5, 0.5 mL/min, 298 K, 210 nm): t_R(*syn*, minor) = 22.7 min, t_R(*syn*, major) = 28.3 min. e.r.(*syn*) = 98:2 (96% ee).

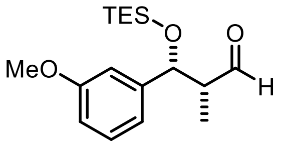

3c

(2*R*,3*R*)-3-(3-Methoxyphenyl)-2-methyl-3-((triethylsilyl)oxy)propanal (3c)

m-Anisaldehyde (24 µL, 0.2 mmol), IDPi **7c** (8.11 mg, 4 µmol, 2 mol%) and (*E*)-TES enolate **2a** (50 µL, 0.24 mmol, 1.2 equiv.) were subjected to the general aldol procedure (Conditions A). Purification by column chromatography with hexanes/CH_2Cl_2 (1:1 gradient to 1/2) afforded aldol **3c** (60 mg, 97%) as a colorless oil.
^{1}H NMR (501 MHz, $CDCl_3$) δ 9.76 (d, *J* = 1.3 Hz, 1H), 7.25 – 7.20 (m, 1H), 6.89 – 6.83 (m, 2H), 6.82 – 6.77 (m, 1H), 5.13 (d, *J* = 4.4 Hz, 1H), 3.81 (s, 3H), 2.58 (qdd, *J* = 6.9, 4.4, 1.3 Hz, 1H), 1.04 (d, *J* = 7.0 Hz, 3H), 0.88 (t, *J* = 7.9 Hz, 9H), 0.53 (q, *J* = 7.8 Hz, 6H).
^{13}C NMR (126 MHz, $CDCl_3$) δ 204.5, 159.6, 144.3, 129.3, 118.7, 112.9, 112.0, 74.3, 55.3, 54.8, 8.3, 6.9, 4.9.
ESI-HRMS: calculated for $C_{17}H_{28}O_3SiNa^+$ ($[M+Na]^+$): 331.1700, found: 331.1697.
$[\alpha]_D^{25}$= +31.3 (*c* = 0.80, $CHCl_3$).
R_f = 0.33 (hexanes/CH_2Cl_2 1:1).

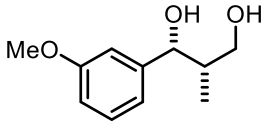

S1c

(1*R*, 2*S*)-1-(3-Methoxyphenyl)-2-methylpropane-1,3-diol (S1c)

Aldol **3c** (43 mg, 0.14 mmol) was subjected to the general procedure for the derivatization of aldols **3** to diols **S1**. Purification by column chromatography with hexanes/AcOEt (2:1 gradient to 1/2) afforded 25 mg (91%) diol **S1c** as a colorless oil.
^{1}H NMR (501 MHz, $CDCl_3$) δ 7.30 – 7.23 (m, 1H), 6.93 – 6.89 (m, 2H), 6.84 – 6.79 (m, 1H), 4.92 (d, *J* = 3.9 Hz, 1H), 3.81 (s, 3H), 3.74 – 3.63 (m, 2H), 2.67 (br. s, 1H), 2.13 (br. s, 1H), 2.11 – 1.99 (m, 1H), 0.87 (d, *J* = 7.0 Hz, 3H).
^{13}C NMR (126 MHz, $CDCl_3$) δ 159.7, 144.7, 129.4, 118.6, 112.8, 111.9, 76.7, 66.7, 55.4, 41.7, 11.0.
ESI-HRMS: calculated for $C_{11}H_{16}NaO_3^+$ ($[M+Na]^+$): 219.0992, found: 219.1000.
$[\alpha]_D^{25}$ = +36.50 (*c* = 0.40, $CHCl_3$).
R_f = 0.3 (AcOEt/hexanes 3:2).
HPLC (IC-3, *n*-heptane/*i*-PrOH = 90:10, 0.5 mL/min, 298 K, 215 nm): t_R(*syn*, minor) = 19.5, t_R(*syn*, major) = 35.4 min. e.r.(*syn*) = 98.7:1.3 (97.4% ee).

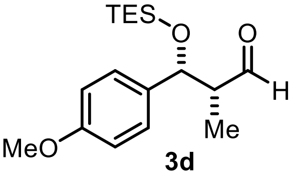

3d

(2*R*,3*R*)-3-(4-Methoxyphenyl)-2-methyl-3-((triethylsilyl)oxy)propanal (3d)

4-Methoxybenzaldehyde (24 µL, 0.2 mmol), IDPi **7c** (8.11 mg, 4 µmol, 2 mol%) and (*E*)-TES enolate **2a** (50 µL, 0.24 mmol, 1.2 equiv.) were subjected to the general aldol procedure (Conditions A). Purification by column chromatography with hexanes/CH_2Cl_2 (3:1 gradient to 1:1) afforded aldol **3d** (60 mg, 97%) as a colorless oil.
^{1}H NMR (501 MHz, $CDCl_3$) δ 9.74 (d, *J* = 1.4 Hz, 1H), 7.20 (d, *J* = 8.6 Hz, 2H), 6.85 (d, *J* = 8.7 Hz, 2H), 5.05 (d, *J* = 4.8 Hz, 1H), 3.80 (s, 3H), 2.59 (qdd, *J* = 6.9, 4.9, 1.4 Hz, 1H), 1.04 (d, *J* = 6.9 Hz, 3H), 0.86 (t, *J* = 8.0 Hz, 19H), 0.50 (q, *J* = 8.1 Hz, 6H).
^{13}C NMR (126 MHz, $CDCl_3$) δ 204.7, 159.1, 134.7, 127.5, 127.5, 113.6, 74.3, 55.4, 55.0, 8.7, 6.89, 6.86, 4.94, 4.92.
ESI-HRMS: calculated for $C_{17}H_{28}O_3SiNa^+$ ($[M+Na]^+$): 331.1700, found: 331.1697.
$[\alpha]_D^{25}$ = +21.6 (*c* = 0.49, $CHCl_3$)
R_f = 0.32 (hexanes/CH_2Cl_2 1:1).

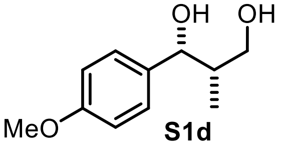

S1d

(1*R*,2*S*)-1-(4-Methoxyphenyl)-2-methylpropane-1,3-diol (S1d)
Aldol **3d** (30 mg, 0.10 mmol) was subjected to the general procedure for the derivatization of aldols **3** to diols **S1**. Purification by column chromatography with hexanes/AcOEt (2:1 gradient to 3:2) afforded 16 mg (84%) diol **S1d** as a colorless solid.
^{1}H NMR (501 MHz, $CDCl_3$) δ 7.26 (d, *J* = 8.6 Hz, 2H), 6.89 (d, *J* = 8.7 Hz, 2H), 4.87 (d, *J* = 4.2 Hz, 1H), 3.81 (s, 3H), 3.65 (d, *J* = 5.4 Hz, 2H), 2.31 (br. s, 2H), 2.06 (dtdd, *J* = 12.4, 7.0, 5.4, 4.3 Hz, 1H), 0.87 (d, *J* = 7.1 Hz, 3H).
^{13}C NMR (126 MHz, $CDCl_3$) δ 159.15, 134.8, 127.5, 113.8, 76.9, 66.6, 55.4, 41.7, 11.3.
ESI-HRMS: calculated for $C_{11}H_{15}O_3^-$ ([M-H]$^-$): 195.1027, found: 195.1028.
$[\alpha]_D^{25}$ = +46.1 (*c* = 0.36, $CHCl_3$)
R_f = 0.24 (hexanes/AcOEt 1:1).
HPLC (IC-3, *n*-heptane/*i*-PrOH=90:10, 1.0 mL/min, 298 K, 225 nm): t_R(*syn*, minor) = 10.0, t_R(*syn*, major) = 11.2 min. e.r.(*syn*) = 93.8:6.2 (87.6% ee).

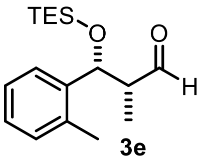

3e

(2*R*,3*R*)-2-Methyl-3-(*o*-tolyl)-3-((triethylsilyl)oxy)propanal (3e)
2-Methylbenzaldehyde (30 µL, 0.2 mmol), IDPi **7c** (2.7 mg, 1.3 µmol, 0.5 mol%) and (*E*)-TES enolate **2a** (50 µL, 0.24 mmol, 1.2 equiv) were subjected to the general aldol procedure (Conditions A, but at -50 C). Purification by column chromatography with hexanes/CH_2Cl_2 (4:1 gradient to 1:1) afforded aldol **3e** (74 mg, 97%) as a colorless oil as a mixture of diastereomers (d.r. *syn/anti* 87.5:12.5). Repurification allowed for separation of diastereomers for characterization, giving pure *syn*-aldol **3e** (62 mg, 82%) and mixture of diastereomers (10 mg, 13%, *syn/anti* 57.5:41.5).
^{1}H NMR (300 MHz, $CDCl_3$) δ 9.74 (d, *J* = 1.2 Hz, 1H), 7.47 (dd, *J* = 7.2, 1.9 Hz, 1H), 7.25-7.07 (m, 3H), 5.42 (d, *J* = 3.8 Hz, 1H), 2.51 (qdd, *J* = 7.0, 3.9, 1.2 Hz, 1H), 2.31 (s, 3H), 1.08 (d, *J* = 6.9 Hz, 3H), 0.85 (t, *J* = 7.9 Hz, 9H), 0.47 (d, *J* = 7.8 Hz, 3H), 0.50 (q, *J* = 7.8 Hz, 6H).
^{13}C NMR (75 MHz, $CDCl_3$) δ 204.6, 140.7, 133.2, 130.4, 127.31, 127.26, 125.9, 70.2, 52.8, 19.1, 7.7, 6.8, 4.9.
ESI-HRMS: calculated for $C_{17}H_{28}NaO_2Si^+$ ([M+Na]$^+$) : 315.1751, found: 315.1750.
$[\alpha]_D^{25}$ =+39.11 (*c* = 0.45, $CHCl_3$) (pure *syn*); $[\alpha]_D^{25}$ =+52.57 (*c* = 0.70, $CHCl_3$) (mixture of diastereoisomers)
R_f (*syn*) = 0.37 (hexanes/CH_2Cl_2 1:1).
R_f (*anti*) = 0.30 (hexanes/CH_2Cl_2 1:1).

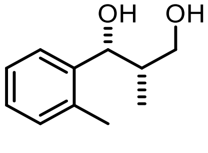

S1e

(1*R*,2*S*)-2-Methyl-1-(*o*-tolyl)propane-1,3-diol (S1e)
Aldol **3e** (25 mg, 0.086 mmol) was subjected to the general procedure for the derivatization of aldols **3** to diols **S1**. Purification by column chromatography with hexanes/AcOEt (2:1 gradient to 1:1) afforded diol **S1e** (17 mg, 92%) as a colorless oil.

^{1}H NMR (501 MHz, $CDCl_3$) δ 7.51 (dd, *J* = 7.7, 1.4 Hz, 1H), 7.26 – 7.21 (m, 1H), 7.17 (td, *J* = 7.4, 1.5 Hz, 1H), 7.13 (dd, *J* = 7.4, 1.6 Hz, 1H), 5.23 (d, *J* = 3.5 Hz, 1H), 3.79 (dd, *J* = 10.6, 4.2 Hz, 1H), 3.74 (dd, *J* = 10.6, 5.8 Hz, 1H), 2.31 (s, 3H), 2.22 (br. s, 1H), 2.01 – 1.93 (m, 1H), 1.63 (br. s, 1H), 0.92 (d, *J* = 7.0 Hz, 3H).
^{13}C NMR (126 MHz, $CDCl_3$) δ 141.4, 134.3, 130.5, 127.2, 126.2, 126.0, 72.6, 67.2, 39.9, 19.2, 10.0.
ESI-HRMS: calculated for $C_{11}H_{16}NaO_2^+$ ([M+Na]$^+$): 203.1042; found: 203.1044.
$[\alpha]_D^{25}$ = +42.0 (*c* = 0.21, $CHCl_3$).
R_f = 0.26 (hexanes/AcOEt 1:1).
HPLC (IC-3, *n*-heptane/*i*-PrOH=95:5, 0.5 mL/min, 298 K, 211 nm): t_R(*syn*, minor) = 19.1 min, t_R(*syn*, major) = 23.7 min, t_R(*anti*, minor) = 29.5 min, t_R(*anti*, major) = 33.6 min.
e.r. (*syn*) = 98:2 (96% ee); e.r. (*anti*) = 97:3 (94% ee).

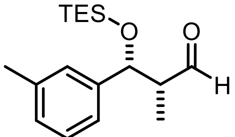

3f

(2*R*,3*R*)-2-Methyl-3-(*m*-tolyl)-3-((triethylsilyl)oxy)propanal (3f)
3-Methylbenzaldehyde (24 µL, 0.2 mmol), IDPi **7c** (8.11 mg, 4 µmol, 2 mol%) and (*E*)-TES enolate **2a** (50 µL, 0.24 mmol, 1.2 equiv.) were subjected to the general aldol procedure (Conditions A). Purification by column chromatography with hexanes/CH_2Cl_2 (4:1 gradient to 1:1) afforded aldol **3f** (56 mg, 96%) as a colorless oil.
^{1}H NMR (501 MHz, $CDCl_3$) δ 9.76 (d, *J* = 1.3 Hz, 1H), 7.23-7.17 (m, 1H), 7.11-7.04 (m, 3H), 5.10 (d, *J* = 4.5 Hz, 1H), 2.59 (qdd, *J* = 6.9, 4.5, 1.4 Hz, 1H), 2.34 (s, 3H), 1.04 (d, *J* = 6.9 Hz, 3H), 0.87 (t, *J* = 7.9 Hz, 9H), 0.51 (q, *J* = 7.5, 7.1 Hz, 6H).
^{13}C NMR (126 MHz, $CDCl_3$) δ 204.6, 142.5, 137.8, 128.3, 128.1, 127.0, 123.5, 74.5, 54.9, 21.6, 8.4, 6.8, 4.9.
ESI-HRMS: calculated for $C_{17}H_{28}O_2SiNa^+$ ([M+Na]$^+$): 315.1751, found: 315.1754.
$[\alpha]_D^{25}$ = +29.6 (*c* = 0.75, $CHCl_3$).
R_f = 0.37 (hexanes/CH_2Cl_2 3:2).

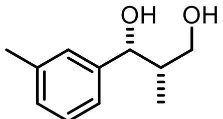

S1f

(1*R*,2*S*)-2-Methyl-1-(*m*-tolyl)propane-1,3-diol (S1f)
Aldol **3f** (43 mg, 0.15 mmol) was subjected to the general procedure for the derivatization of aldols **3** to diols **S1**. Purification by column chromatography with hexanes/AcOEt (3:1 gradient to 1:1) afforded 24 mg (91%) diol **S1f** as a colorless oil.
^{1}H NMR (501 MHz, $CDCl_3$) δ 7.23 (t, *J* = 7.5 Hz, 1H), 7.14 (s, 1H), 7.12 (d, *J* = 7.7 Hz, 1H), 7.08 (d, *J* = 7.5 Hz, 1H), 4.89 (d, *J* = 3.9 Hz, 1H), 3.68-3.63 (m, 2H), 2.89 (s, 1H), 2.36 (s, 3H), 2.34 (br. s, 2H), 2.11-2.02 (m, 1H), 0.87 (d, *J* = 7.1 Hz, 3H).
^{13}C NMR (126 MHz, $CDCl_3$) δ 142.8, 137.9, 128.2, 128.1, 126.9, 123.3, 76.9, 66.6, 41.6, 21.6, 11.0.
ESI-HRMS: calculated for $C_{11}H_{16}NaO_2^+$ ([M+Na]$^+$): 203.1042; found: 203.1043.
$[\alpha]_D^{25}$ = +47.7 (*c* = 0.60, $CHCl_3$).
R_f = 0.30 (hexanes/AcOEt 1:1).
HPLC (IC-3, *n*-heptane/*i*-PrOH=95:5, 1.0 mL/min, 298 K, 215 nm): t_R(*syn*, minor) = 12.4 min, t_R(*syn*, major) = 17.1 min. e.r.(*syn*) = 98:2 (96% ee)

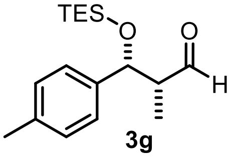

3g

(2*R*,3*R*)-2-Methyl-3-(*p*-tolyl)-3-((triethylsilyl)oxy)propanal (3g)
4-Methylbenzaldehyde (24 µL, 0.2 mmol), IDPi **7c** (8.11 mg, 4 µmol, 2 mol%) and (*E*)-TES enolate **2a** (50 µL, 0.24 mmol, 1.2 equiv.) were subjected to the general aldol procedure (Conditions A). Purification by column chromatography with hexanes/CH_2Cl_2 (4:1 gradient to 1:1) afforded aldol **3g** (55 mg, 94%) as a colorless oil.
^{1}H NMR (501 MHz, $CDCl_3$) δ 9.75 (d, *J* = 1.3 Hz, 1H), 7.17 (d, *J* = 8.1 Hz, 2H), 7.12 (d, *J* = 7.9 Hz, 2H), 5.09 (d, *J* = 4.6 Hz, 1H), 2.62 – 2.51 (m, 1H), 2.33 (s, 3H), 1.03 (d, *J* = 6.9 Hz, 3H), 0.86 (t, *J* = 7.9 Hz, 9H), 0.51 (q, *J* = 7.7 Hz, 6H).
^{13}C NMR (126 MHz, $CDCl_3$) δ 204.7, 139.5, 137.2, 129.0, 126.3, 74.4, 55.0, 21.3, 8.5, 6.9, 4.9.
ESI-HRMS: calculated for $C_{17}H_{28}NaO_2Si^+$ ($[M+Na]^+$): 315.1751, found: 315.1750.
$[\alpha]_D^{25}$ = +24.90 (*c* = 0.90, $CHCl_3$).
R_f = 0.35 (hexanes/CH_2Cl_2 = 3:2).

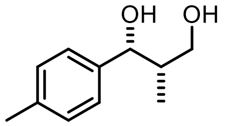

S1g

(1*R*,2*S*)-2-Methyl-1-(*p*-tolyl)propane-1,3-diol (S1g) (S1g)
Aldol **3g** (30 mg, 0.103 mmol) was subjected to the general procedure for the derivatization of aldols **3** to diols **S1**. Purification by column chromatography with hexanes/AcOEt (2:1 gradient to 1:1) afforded 16 mg (87%) diol **S1g** (e.r. = 4.5:95.5) as a colorless solid.
^{1}H NMR (501 MHz, $CDCl_3$) δ 7.23 (d, *J* = 8.1 Hz, 2H), 7.16 (d, *J* = 7.9 Hz, 2H), 4.90 (d, *J* = 4.1 Hz, 1H), 3.69 – 3.63 (m, 2H), 2.55 (br. s, 1H), 2.35 (s, 3H), 2.18 (br. s, 1H), 2.11 – 2.02 (m, 1H), 0.86 (d, *J* = 7.1 Hz, 3H).
^{13}C NMR (126 MHz, $CDCl_3$) δ 139.7, 137.1, 129.0, 126.2, 77.0, 66.6, 41.6, 21.2, 11.2.
ESI-HRMS: calculated for $C_{11}H_{16}NaO_2^+$ ($[M+Na]^+$): 203.1042; found: 203.1042.
$[\alpha]_D^{25}$ = +39.56 (*c* =0.45, $CHCl_3$).
R_f = 0.28 (hexanes/AcOEt 1:1).
HPLC (IC-3, *n*-heptane/*i*-PrOH=95:5, 1.0 mL/min, 298 K, 213 nm): t_R(*syn*, minor) = 14.5, t_R(*syn*, major) = 17.7 min.
e.r.(*syn*) = 95.5:4.5 (91% ee).

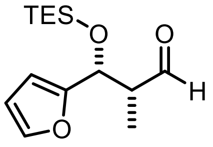

3h

(2*R*,3*R*)-3-(Furan-2-yl)-2-methyl-3-((triethylsilyl)oxy)propanal (3h)
An oven dried screw-cap vial was charged with IDPi catalyst **7c** (51.4 mg, 25 µmol, 0.5 mol%) and dissolved in 8 mL dry $CHCl_3$. Furan-2-carboxaldehyde (414 µL, 5 mmol) was added and the screw cap was sealed wrapped with parafilm. The vial was placed into a precooled –60 °C cryostat and cooled for 20 min. Neat (*E*)-TES enolate **2a** (1.26 mL, 6 mmol, 1.2 equiv.) was added slowly over 5 min. The hole of the screw-cap was sealed with a silicon grease and the reaction mixture was stirred at –60 °C for 72 h. The reaction mixture was quenched with Et_3N (12 µL) and warmed to r.t. The reaction mixture was concentrated to give a pale yellow oil, which was purified by column chromatography on silicagel (hexanes/CH_2Cl_2 3:1 gradient to 3:2) to give 1.244 g (93%) of aldol **3h** as a colorless oil. The catalyst was recovered by eluting with hexanes/MTBE (8/1 gradient to 3:1).
^{1}H NMR (501 MHz, $CDCl_3$) δ 9.83 (d, *J* = 1.2 Hz, 1H), 7.35 (d, *J* = 1.8 Hz, 1H), 6.31 (dd, *J* = 3.3, 1.8 Hz, 1H), 6.22 (d, *J* = 3.2 Hz, 1H), 5.04 (d, *J* = 5.4 Hz, 1H), 2.79 (qdd, *J* = 6.9, 5.3, 1.2 Hz, 1H), 1.07 (d, *J* = 7.0 Hz, 3H), 0.89 (t, *J* = 7.9 Hz, 9H), 0.54 (q, *J* = 7.9 Hz, 6H).
^{13}C NMR (126 MHz, $CDCl_3$) δ 204.0, 154.7, 142.1, 110.3, 107.7, 68.7, 52.4, 9.3, 6.8, 4.7.
ESI-HRMS: calculated for $C_{14}H_{24}NaO_3Si^+$ ($[M+Na]^+$): 291.1387; found: 291.1387.
$[\alpha]_D^{25}$ = +23.4 (*c* = 0.47, $CHCl_3$).
R_f = 0.26 (hexanes/CH_2Cl_2 1:1).

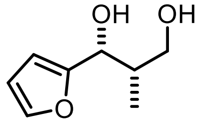

S1h

(1*R*,2*S*)-1-(Furan-2-yl)-2-methylpropane-1,3-diol (S1h)

Aldol **3h** (33 mg, 0.12 mmol) was subjected to the general procedure for the derivatization of aldols **3** to diols **S1**. Purification by column chromatography with hexanes/AcOEt (2:1 gradient to 1:1) afforded 16 mg (83%) diol **S1h** as a colorless oil.

^{1}H NMR (501 MHz, $CDCl_3$) δ 7.38 (dd, *J* = 1.7, 0.9 Hz, 1H), 6.35 (dd, *J* = 3.3, 1.9 Hz, 1H), 6.27 (d, *J* = 3.2 Hz, 1H), 4.90 (d, *J* = 4.0 Hz, 1H), 3.75 – 3.61 (m, 2H), 2.85 (br. s, 1H), 2.24 (pt, *J* = 7.0, 4.4 Hz, 1H), 2.11 (br. s, 1H), 0.93 (d, *J* = 7.0 Hz, 3H).

^{13}C NMR (126 MHz, $CDCl_3$) δ 155.6, 141.9, 110.3, 106.7, 71.4, 66.4, 39.9, 11.6.

ESI-HRMS: calculated for $C_8H_{12}O_3Na^+$ ($[M+Na]^+$): 179.0679; found: 179.0679.

$[\alpha]_D^{25}$ = +25.9 (*c* = 0.30, $CHCl_3$).

R_f = 0.26 (hexanes/AcOEt 1:1).

HPLC (IC-3, *n*-heptane/*i*-PrOH=90:10, 1.0 mL/min, 298 K, 215 nm): t_R(*syn*, minor) = 8.8 min, t_R(*syn*, major) = 11.9 min. e.r.(*syn*) = 96:4 (92% ee).

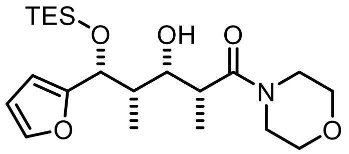

13

(2*R*,3*S*,4*S*,5*R*)-5-(Furan-2-yl)-3-hydroxy-2,4-dimethyl-1-morpholino-5-((triethylsilyl)oxy)pentan-1-one (13)

A flame-dried Schlenk flask under Ar was charged with (^{l}Ipc)$_2$BH (72 mg, 0.25 mmol, 1.17 equiv.) weighed under Ar and dry Et_2O (1 mL) was added to give a white suspension. The suspension was cooled to 0 °C and 4-acryloylmorpholine (35 µL, 0.277 mmol, 1.3 equiv.) was added. The solution was stirred for 2 h at 0 °C during which it became homogeneous. The reaction mixture was cooled to –78 °C and a solution of aldehyde **3h** (57 mg, 0.213 mmol) in dry Et_2O (0.5 mL) was added and the solution was stirred overnight at –78 °C. An aqueous pH 7 buffer solution (0.5 mL), MeOH (0.5 mL) and THF (0.5 mL) were added and the reaction was stirred for 2 h at room temperature. After two hours H_2O_2 (2 mL, 33% solution) was added and the mixture was stirred for additional 2 h. The aqueous phase was extracted with CH_2Cl_2 (x3). The combined organic extracts were dried over Na_2SO_4, filtered, and concentrated under reduced pressure. Purification of the crude product by flash chromatography (CH_2Cl_2/AcOEt 6:1 gradient to 3:1) provided all-*syn* stereotetrad **13** (65 mg, 74%) as a single diastereomer as a colorless crystalline solid.

Crystallization for X-ray analysis was achieved via slow diffusion of hexanes to a solution of **12 in ethylacetate.*

^{1}H NMR (501 MHz, $CDCl_3$) δ 7.33 (dd, *J* = 1.8, 0.8 Hz, 1H), 6.31 (dd, *J* = 3.2, 1.8 Hz, 1H), 6.17 (d, *J* = 3.2 Hz, 1H), 4.78 (d, *J* = 4.0 Hz, 1H), 3.80 (t, *J* = 5.3 Hz, 1H), 3.72 – 3.45 (m, 9H), 3.07 (qd, *J* = 7.0, 4.9 Hz, 1H), 2.08 – 1.94 (m, 1H), 1.20 (d, *J* = 7.0 Hz, 3H), 1.01 (d, *J* = 7.0 Hz, 3H), 0.88 (t, *J* = 7.9 Hz, 8H), 0.50 (qd, *J* = 7.9, 2.9 Hz, 6H).

^{13}C NMR (126 MHz, $CDCl_3$) δ 175.6, 155.2, 141.6, 110.2, 107.5, 74.3, 72.9, 67.1, 67.0, 46.3, 41.9, 41.2, 37.8, 12.8, 10.7, 6.8, 4.6.

ESI-HRMS: calculated for $C_{21}H_{37}NNaO_5Si^+$ ($[M+Na]^+$): 434.2333; found: 434.2330.

$[\alpha]_D^{25}$ = +57.1 (*c* = 0.21, $CHCl_3$).

R_f = 0.23 (CH_2Cl_2/AcOEt 3:1).

m.p. 116-118 °C.

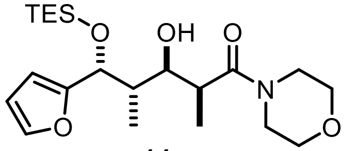

14

(2*S*,3*R*,4*S*,5*R*)-5-(Furan-2-yl)-3-hydroxy-2,4-dimethyl-1-morpholino-5-((triethylsilyl)oxy)pentan-1-one (14)
A flame-dried Schlenk flask under Ar was charged with (^{d}Ipc)$_2$BH (77 mg, 0.27 mmol, 1.2 equiv.) weighed under Ar and dry Et_2O (1 mL) was added to give a white suspension. The suspension was cooled to 0 °C and 4-acryloylmorpholine (37 µL, 0.29 mmol, 1.3 equiv.) was added. The solution was stirred for 2 h at 0 °C during which it became homogeneous. The reaction mixture was cooled to –78 °C and a solution of aldehyde aldehyde **3h** (60 mg, 0.224 mmol) in dry Et_2O (0.5 mL) was added and the solution was stirred overnight at -78 °C. An aqueous pH 7 buffer solution (0.5 mL), MeOH (0.5 mL) and THF (0.5 mL) were added and the reaction was stirred for 2 h at room temperature. After 2 h H_2O_2 (2 mL, 33% solution) was added and the mixture was stirred for additional 2 h. The aqueous phase was extracted with CH_2Cl_2 (x3). The combined organic extracts were dried over Na_2SO_4, filtered, and concentrated under reduced pressure. Purification of the crude product by flash chromatography (CH_2Cl_2/AcOEt 15:1 gradient to 3:1) provided stereotetrad **14** (50 mg, 54%) as a single diastereomer as a colorless oil.
^{1}H NMR (501 MHz, $CDCl_3$) δ 7.32 (dd, *J* = 1.9, 0.9 Hz, 1H), 6.30 (dd, *J* = 3.2, 1.8 Hz, 1H), 6.19 (d, *J* = 3.3 Hz, 1H), 5.35 (d, *J* = 1.8 Hz, 1H), 4.70 (br. s, 1H), 3.84 (dd, *J* = 10.0, 1.8 Hz, 1H), 3.76 – 3.64 (m, 5H), 3.64 – 3.55 (m, 1H), 3.53 – 3.45 (m, 2H), 2.74 (qd, *J* = 7.2, 1.8 Hz, 1H), 1.85 (dqd, *J* = 11.7, 6.9, 1.9 Hz, 1H), 1.13 (d, *J* = 7.2 Hz, 3H), 0.91 (t, *J* = 7.9 Hz, 9H), 0.78 (d, *J* = 6.9 Hz, 3H), 0.58 (q, *J* = 7.9 Hz, 6H).
^{13}C NMR (126 MHz, $CDCl_3$) δ 176.5, 157.7, 141.1, 110.0, 106.2, 71.5, 68.1, 67.0, 66.8, 46.4, 42.1, 41.3, 35.7, 9.54, 9.47, 6.9, 4.8.
ESI-HRMS: calculated for $C_{21}H_{38}NO_5Si^+$ ($[M+Na]^+$): 412.2514; found: 412.2517.
$[\alpha]_D^{25}$ = +18.3 (*c* = 0.153, $CHCl_3$).
R_f(CH_2Cl_2/AcOEt 5:1) = 0.38.

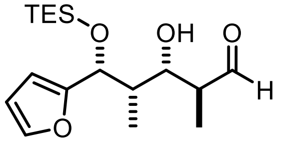

15

(2*S*,3*S*,4*S*,5*R*)-5-(Furan-2-yl)-3-hydroxy-2,4-dimethyl-5-((triethylsilyl)oxy)pentanal (15)
A 4 mL screw-cap vial with a septum was charged with the aldol **3h** (27 mg, 0.1 mmol) and 0.1 mL dry DMF was added, followed by *L*-proline (2.3 mg, 0.02 mmol). The vial was put in a precooled cryostat at 4 °C. A solution of propionaldehyde (14 µL, 0.2 mmol) in DMF (0.2 mL) was added via syringe pump over 20 h. After the addition of propionaldehyde was complete, the vial was put into a 4 °C refrigerator and kept for additional 40 h. The reaction mixture was quenched by adding excess Et_2O and water. The Et_2O phase was washed intensively with small portions of water (ca 10 times, 100 mL) to remove most of the propionaldehyde homoaldol byproduct and DMF. The organic phase was dried over anhydrous Na_2SO_4, filtered and evaporated. The residue was purified by column chromatography (hexanes/AcOEt 20:1 gradient to 10:1) to provide 10 mg (30%) of sensitive aldehyde **15** and 13 mg (48%) recovered aldol **3h**. The d.r. of the isolated product **15** was enriched to 95:5.
^{1}H NMR (501 MHz, $CDCl_3$) δ 9.74 (d, *J* = 2.0 Hz, 1H), 7.36 (dd, *J* = 1.8, 0.8 Hz, 1H), 6.33 (dd, *J* = 3.2, 1.8 Hz, 1H), 6.24 (dd, *J* = 3.3, 0.8 Hz, 1H), 4.80 (d, *J* = 6.5 Hz, 1H), 3.70 (dd, *J* = 9.4, 2.0 Hz, 1H), 2.54 (dqd, *J* = 9.3, 7.3, 2.1 Hz, 1H), 2.08 – 1.97 (m, 1H), 1.04 (d, *J* = 6.9 Hz, 3H), 1.00 (d, *J* = 7.3 Hz, 3H), 0.88 (t, *J* = 7.9 Hz, 9H), 0.56 – 0.49 (m, 6H). *The OH-resonance could not be safely assigned.*
^{13}C NMR (126 MHz, $CDCl_3$) δ 205.9, 155.5, 141.8, 110.3, 107.6, 73.4, 72.3, 49.8, 40.8, 10.6, 8.0, 6.8, 4.7.
ESI-HRMS: calculated for $C_{17}H_{30}NaO_4Si^+$ ($[M+Na]^+$): 349.1806; found: 349.1805.
R_f(hexanes/AcOEt 9:1) = 0.24.

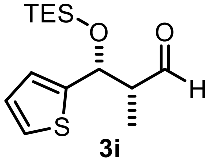

3i

(2*R*, 3*R*)-2-Methyl-3-(thiophen-2-yl)-3-((triethylsilyl)oxy)propanal (3i)

2-Thiophenecarboxaldehyde (19 µL, 0.2 mmol), IDPi **7c** (8.11 mg, 4 µmol, 2 mol%) and (*E*)-TES enolate **2a** (50 µL, 0.24 mmol, 1.2 equiv.) were subjected to the general aldol procedure (Conditions A). Purification by column chromatography with hexanes/CH_2Cl_2 (2:1 gradient to 3:2) afforded aldol **3i** (55 mg, 97%) as a colorless oil.

^{1}H NMR (501 MHz, $CDCl_3$) δ 9.77 (d, *J* = 1.3 Hz, 1H), 7.22 (dd, *J* = 5.0, 1.2 Hz, 1H), 6.94 (dd, *J* = 5.0, 3.5 Hz, 1H), 6.91 (dt, *J* = 3.5, 1.0 Hz, 1H), 5.34 (dd, *J* = 5.0, 0.7 Hz, 1H), 2.69 (qdd, *J* = 7.0, 5.1, 1.3 Hz, 1H), 1.13 (d, *J* = 7.0 Hz, 3H), 0.89 (t, *J* = 7.9 Hz, 9H), 0.56 (q, *J* = 7.9 Hz, 6H).

^{13}C NMR (126 MHz, $CDCl_3$) δ 204.0, 146.79, 126.6, 124.7, 124.0, 71.1, 55.1, 9.2, 6.8, 4.9.

ESI-HRMS: calculated for $C_{14}H_{24}NaO_2SSi^+$ ($[M+Na]^+$): 307.1158; found: 307.1157.

$[\alpha]_D^{25}$ =+37.84 (*c* =0.55, $CHCl_3$).

R_f = 0.24 (hexanes/CH_2Cl_2 3:2).

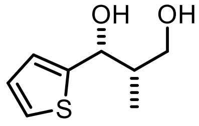

S1i

(1*R*, 2*S*)-2-Methyl-1-(thiophen-2-yl)propane-1,3-diol

Aldol **3i** (40 mg, 0.14 mmol) was subjected to the general procedure for the derivatization of aldols **3** to diols **S1**. Purification by column chromatography with hexanes/AcOEt (2:1 gradient to 3:2) afforded 22 mg (91%) diol **S1i** as a colorless crystalline solid.

Crystallization for X-ray analysis was achieved via slow diffusion of hexanes to a solution of ***S1i* in ethylacetate.*

^{1}H NMR (501 MHz, $CDCl_3$) δ 7.25 (dd, *J* = 4.9, 1.3 Hz, 1H), 7.00 (dd, *J* = 5.1, 3.5 Hz, 1H), 6.98 – 6.96 (m, 1H), 5.18 (d, *J* = 4.0 Hz, 1H), 3.67-3.76 (m, 2H), 2.91 (br. s, 1H), 2.11-2.23 (m, 1H), 2.02 (br. s, 1H), 0.96 (d, *J* = 7.0 Hz, 3H).

^{13}C NMR (126 MHz, $CDCl_3$) δ 146.8, 126.8, 124.4, 123.8, 73.6, 66.3, 42.0, 11.4.

ESI-HRMS: calculated for $C_8H_{12}NaO_2S^+$ ($[M+Na]^+$): 195.0450; found: 195.0452.

$[\alpha]_D^{25}$ = +24.2 (*c* = 0.41, $CHCl_3$).

R_f = 0.33 (hexanes/AcOEt 1:1).

m.p. 80-81 °C

HPLC (IC-3, *n*-heptane/*i*-PrOH=90:10, 0.5 mL/min, 298 K, 234 nm): t_R(*syn*, minor) = 12.9 min, t_R(*syn*, major) = 15.2 min. e.r. (*syn*) = 97.4:2.6 (94.8% ee).

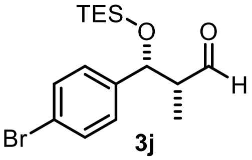

3j

(2*R*,3*R*)-3-(4-Bromophenyl)-2-methyl-3-((triethylsilyl)oxy)propanal (3j)

4-Bromobenzaldehyde (37 mg, 0.2 mmol), IDPi **7c** (8.11 mg, 4 µmol, 2 mol%) and (*E*)-TES enolate **2a** (50 µL, 0.24 mmol, 1.2 equiv.) were subjected to the general aldol procedure (Conditions A). Purification by column chromatography with hexanes/CH_2Cl_2 (4:1 gradient to 1:1) afforded aldol **3j** (69 mg, 96%) as a colorless oil.

^{1}H NMR (501 MHz, $CDCl_3$) δ 9.75 (d, *J* = 1.2 Hz, 1H), 7.45 (d, *J* = 8.4 Hz, 2H), 7.18 (d, *J* = 8.4 Hz, 2H), 5.13 (d, *J* = 4.3 Hz, 1H), 2.55 (qdd, *J* = 6.9, 4.3, 1.3 Hz, 1H), 1.03 (d, *J* = 7.0 Hz, 3H), 0.87 (t, *J* = 7.9 Hz, 9H), 0.51 (q, *J* = 7.8 Hz, 6H).

^{13}C NMR (126 MHz, $CDCl_3$) δ 204.0, 141.8, 131.5, 128.0, 121.4, 73.7, 54.7, 8.2, 6.8, 4.9.

ESI-HRMS: calculated for $C_{16}H_{25}BrNaO_2Si^+$ ($[M+Na]^+$): 379.0699, found: 379.0703.

$[\alpha]_D^{25}$ = +62.0 (*c* = 0.10, CH_2Cl_2). (For opposite enantiomer Lit. $[\alpha]_D^{25}$ = -27.01 (*c* = 1.00, CH_2Cl_2) Synlett 2012, 23, 1489–1492).
R_f = 0.42 (hexanes/CH_2Cl_2 3:2).

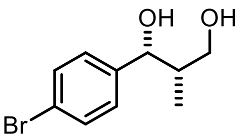

S1j

(1*R*,2*S*)-1-(4-Bromophenyl)-2-methylpropane-1,3-diol (S1i)
Aldol **3j** (43 mg, 0.12 mmol) was subjected to the general procedure for the derivatization of aldols **3** to diols **S1**. Purification by column chromatography with hexanes/AcOEt (3:1 gradient to 1:1) afforded diol **S1j** (23 mg, 78%) as a colorless solid. Crystallization for X-ray analysis was achieved via slow diffusion of hexane to AcOEt solution of diol **S1j**.
Crystallization for X-ray analysis was achieved via slow diffusion of hexanes to a solution of* ***S1j* *in ethylacetate.*
^{1}H NMR (501 MHz, $CDCl_3$) δ 7.47 (d, *J* = 8.4 Hz, 2H), 7.21 (d, *J* = 8.5 Hz, 2H), 4.93 (d, *J* = 3.6 Hz, 1H), 3.72 (dd, *J* = 10.6, 4.2 Hz, 1H), 3.66 (dd, *J* = 10.6, 6.4 Hz, 1H), 2.32 (br. s, 2H), 2.09-1.96 (m, 1H), 0.82 (d, *J* = 7.0 Hz, 3H).
^{13}C NMR (126 MHz, $CDCl_3$) δ 141.9, 131.4, 128.0, 121.1, 76.2, 66.7, 41.4, 10.6.
ESI-HRMS: calculated for $C_{10}H_{13}BrNaO_2^+$ ($[M+Na]^+$): 266.9991; found: 266.9991.
$[\alpha]_D^{25}$ =+39.6 (*c* = 0.45, $CHCl_3$) (Literature value for the opposite enantiomer (d.r. 94:6, 95% ee): $[\alpha]_D^{23.9}$ = −44.4 (c = 0.50, $CHCl_3$) J. Am. Chem. Soc. 2015, 137, 15418−15421)
R_f = 0.28 (hexanes/AcOEt 1:1).
m.p. 119-120 °C
HPLC (IC-3, *n*-heptane/*i*-PrOH=95:5, 0.5 mL/min, 298 K, 220 nm): t_R(syn, minor) = 14.7 min, t_R(syn, major) = 16.2 min. e.r.(*syn*) = 98.4:1.6 (96.8% ee)

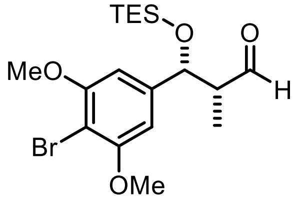

3k

(2*R*,3*R*)-3-(4-Bromo-3,5-dimethoxyphenyl)-2-methyl-3-((triethylsilyl)oxy)propanal (3k)
4-Bromo-3,5-dimethoxybenzaldehyde (1.0 g, 4.1 mmol) was dried overnight under high vacuum in a flame-dried Schlenk flask. IDPi catalyst **7c** (42 mg, 20.4 µmol, 0.5 mol%) was kept under high vacuum in a vial for 3 h before use. Under Ar, the catalyst was dissolved in 2 mL of dry $CHCl_3$ and added to the Schlenk flask containing the aldehyde under Ar. The vial containing the catalyst was washed once with 2 mL $CHCl_3$ and added to the reaction mixture. Additional 4.2 mL $CHCl_3$ were added to the reaction mixture to make up for total concentration of 0.5 M giving a pale yellow solution. The solution was cooled to -40 °C for 15 minutes. Most of the aldehyde precipitated resulting in a slurry and stirring is hampered. Neat enolsilane **2a** was added slowly via the cold walls of the Schlenk flask instead of directly dropping it to the slurry. The Schlenk flask is gently shaken to ensure some level of mixing during the addition of enolsilane. After the addition of enolsilane was complete, the reaction mixture was left in a cooling bath at -40 °C under Ar. After overnight (ca 18 h) the reaction mixture was completely homogeneous. The reaction mixture was stirred further 6 h. After 26 h an aliquot was taken and quenched with Et_3N solution in $CDCl_3$: crude ^{1}H NMR shows starting aldehyde to product ratio SM:Product=3:97. Ca 17% enolsilane is still present. After stirring for additional 2 h (total 28 h) it was quenched with 10 microliters of Et_3N and warmed to r.t. Crude ^{1}H NMR showed almost no difference from the ^{1}H NMR spectrum of aliquot. The reaction mixture was concentrated to give a yellow oil, which was purified by column chromatography on silicagel (hexanes/AcOEt 30/1 to 25/1 to 20/1 to 15/1 to give 1.64 g (96%) aldol **3k** as a colorless viscous oil. The product solidified during transferring to a Schlenk tube.

^{1}H NMR (501 MHz, $CDCl_3$) δ 9.75 (d, *J* = 1.3 Hz, 1H), 6.53 (s, 2H), 5.15 (d, *J* = 4.1 Hz, 1H), 3.89 (s, 6H), 2.56 (qdd, *J* = 6.9, 4.1, 1.3 Hz, 1H), 1.06 (d, *J* = 7.0 Hz, 3H), 0.90 (t, *J* = 7.9 Hz, 9H), 0.55 (q, *J* = 8.1 Hz, 6H).
^{13}C NMR (126 MHz, $CDCl_3$) δ 204.1, 157.0, 143.9, 102.6, 99.7, 74.1, 56.6, 54.7, 8.2, 6.9, 4.9.
ESI-HRMS: calculated for $C_{18}H_{29}BrNaO_4Si^+$ ($[M+Na]^+$): 439.0911; found: 439.0911.
$[\alpha]_D^{25}$ =+40.5 (*c* = 0.39, $CHCl_3$).
R_f = 0.25 (hexanes/AcOEt 15:1).
m.p. 60-62 °C.

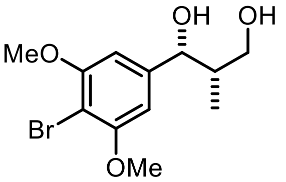

S1k

(1*R*,2*S*)-1-(4-Bromo-3,5-dimethoxyphenyl)-2-methylpropane-1,3-diol (S1k)

Aldol **3k** (30 mg, 0.072 mmol) was subjected to the general procedure for the derivatization of aldols **3** to diols **S1**. Purification by column chromatography with hexanes/AcOEt (1:1 gradient to 1/3) afforded 19 mg (87%) diol **S1k** as a colorless crystalline solid.
^{1}H NMR (501 MHz, $CDCl_3$) δ 6.56 (s, 2H), 4.94 (d, *J* = 3.6 Hz, 1H), 3.89 (s, 6H), 3.76 (dd, *J* = 10.6, 4.2 Hz, 1H), 3.71 (dd, *J* = 10.6, 6.2 Hz, 1H), 2.42 (br. s, 2H), 2.02 (dqd, *J* = 11.1, 6.9, 3.5 Hz, 1H), 0.86 (d, *J* = 7.1 Hz, 3H).
^{13}C NMR (126 MHz, $CDCl_3$) δ 157.0, 144.1, 102.6, 99.4, 76.3, 66.8, 56.6, 41.7, 10.5.
ESI-HRMS: calculated for $C_{12}H_{17}BrNaO_4^+$ ($[M+Na]^+$): 327.0203; found: 327.0202.
$[\alpha]_D^{25}$ =+26.3 (*c* = 0.13, $CHCl_3$).
R_f = 0.21 (hexanes/AcOEt 2:3).
m.p. 109-111 °C.
HPLC (IA-3, *n*-heptane/*i*-PrOH=90:10, 1.0 mL/min, 220 nm): t_R(*syn*, minor) = 9.7 min, t_R(*syn*, major) = 11.6 min.
e.r.(*syn*) = 99.2:0.8 (98.4% ee).

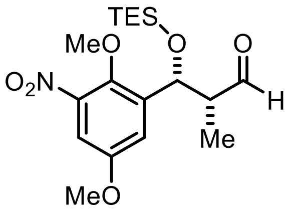

(2*R*,3*R*)-3-(2,5-Dimethoxy-3-nitrophenyl)-2-methyl-3-((triethylsilyl)oxy)propanal (3l)

2,5-Dimethoxy-3-nitrobenzaldehyde (63 mg, 0.3 mmol), IDPi **7c** (6.2 mg, 3 µmol, 1 mol%) and (*E*)-TES enolate **2a** (75 µL, 0.36 mmol, 1.2 equiv.) were subjected to the general aldol procedure until the reaction mixture turns homogeneous. (Conditions A, -40 °C, 2 days). Purification by column chromatography with hexanes/AcOEt (25/1 gradient to 20/1) afforded *syn*-aldol **3l** (92 mg, 80%) as a yellow oil and 20 mg (17%) mixture of syn/anti diastereomers. Overall yield 97%.
^{1}H NMR (501 MHz, $CDCl_3$) δ 9.78 (d, *J* = 0.6 Hz, 1H), 7.31 (d, *J* = 3.3 Hz, 1H), 7.29 (d, *J* = 3.3 Hz, 1H), 5.67 (d, *J* = 2.8 Hz, 1H), 3.88 (s, 3H), 3.84 (s, 3H), 2.64 (qd, *J* = 7.2, 2.9 Hz, 1H), 1.00 (d, *J* = 7.1 Hz, 3H), 0.90 (t, *J* = 8.0 Hz, 9H), 0.57 (q, *J* = 8.1 Hz, 6H).
^{13}C NMR (126 MHz, $CDCl_3$) δ 203.3, 155.2, 143.5, 140.5, 140.5, 119.5, 108.9, 67.6, 62.6, 56.1, 52.5, 7.0, 6.9, 4.9.
ESI-HRMS: calculated for $C_{18}H_{29}NNaO_6Si^+$ ($[M+Na]^+$): 406.1656; found: 406.1657.
$[\alpha]_D^{25}$ =+8.30 (*c* = 0.53, $CHCl_3$).
R_f(*syn*) = 0.22 (hexanes/AcOEt 20:1).
R_f(*anti*) = 0.15 (hexanes/AcOEt 20:1).

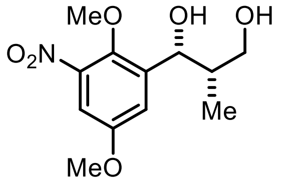

S1l

(1*R*,2*S*)-1-(2,5-Dimethoxy-3-nitrophenyl)-2-methylpropane-1,3-diol (S1l)
Aldol **3l** (30 mg, 0.08 mmol) was subjected to the general procedure for the derivatization of aldols **3** to diols **S1**. Purification by column chromatography with hexanes/AcOEt (1:1 gradient to 1/2) afforded 13 mg (61%) diol **S1l** as a colorless oil.
^{1}H NMR (501 MHz, $CDCl_3$) δ 7.37 (d, *J* = 3.2 Hz, 1H), 7.27 (d, *J* = 3.3 Hz, 1H), 5.32 (d, *J* = 3.3 Hz, 1H), 3.86 (dd, *J* = 10.7, 3.8 Hz, 1H)*, 3.85 (s, 3H), 3.84 (s, 3H), 3.73 (dd, *J* = 10.7, 5.4 Hz, 1H), 2.38 (br. s, 2H), 2.05 – 2.02 (m, 1H), 0.87 (d, *J* = 7.1 Hz, 3H). *Partially overlaps with methoxy singlet resonances.
^{13}C NMR (126 MHz, $CDCl_3$) δ 155.3, 144.1, 143.6, 140.7, 119.2, 108.6, 70.9, 67.3, 62.8, 56.1, 40.1, 10.0.
ESI-HRMS: calculated for $C_{12}H_{17}NNaO_6^+$ ($[M+Na]^+$): 294.0948; found: 294.0948.
$[\alpha]_D^{25}$ = +6.1 (*c* = 0.28, $CHCl_3$).
R_f = 0.34 (hexanes/AcOEt 1:2).
HPLC (IB-N3, *n*-heptane/*i*-PrOH=95:5, 1.0 mL/min, 220 nm): t_R(*syn*, minor) = 12.8 min, t_R(*syn*, major) = 13.6 min. e.r.(*syn*) = 97.1:2.9 (94.2% ee).

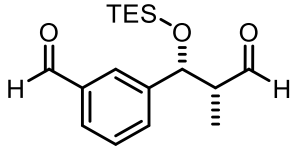

3m

3-((1*R*,2*R*)-2-Methyl-3-oxo-1-((triethylsilyl)oxy)propyl)benzaldehyde (3m)
Isopthalaldehyde (13.4 mg, 0.2 mmol), IDPi **7c** (4.10 mg, 2 µmol, 2 mol%) and (*E*)-TES enolate **2a** (25 µL, 0.12 mmol, 1.2 equiv.) were subjected to the general aldol procedure (Conditions A, -50 °C). Purification by column chromatography with hexanes/AcOEt (20/1 gradient to 10/1) afforded aldol **3m** (27 mg, 88%) as a colorless oil.
^{1}H NMR (501 MHz, $CDCl_3$) δ 10.02 (s, 1H), 9.79 (d, *J* = 1.1 Hz, 1H), 7.83 (s, 1H), 7.79 (dt, *J* = 7.5, 1.5 Hz, 1H), 7.60 (dt, *J* = 7.7, 1.6 Hz, 1H), 7.52 (t, *J* = 7.6 Hz, 1H), 5.29 (d, *J* = 4.2 Hz, 1H), 2.61 (qdd, *J* = 6.9, 4.2, 1.1 Hz, 1H), 1.03 (d, *J* = 7.0 Hz, 3H), 0.85 (t, *J* = 7.9 Hz, 9H), 0.51 (q, *J* = 7.7 Hz, 6H).
^{13}C NMR (126 MHz, $CDCl_3$) δ 203.9, 192.4, 144.0, 136.4, 132.4, 129.3, 129.1, 127.2, 73.5, 54.6, 8.0, 6.8, 4.8.
ESI-HRMS: calculated for $C_{17}H_{26}NaO_3Si^+$ ($[M+Na]^+$): 329.1543; found: 329.1542.
$[\alpha]_D^{25}$ = + 30.0 (*c* = 0.43, $CHCl_3$).
R_f =0.35 (hexanes/AcOEt 8:1).

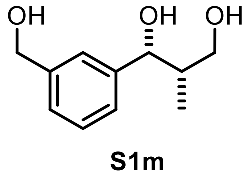

S1m

(1*R*,2*S*)-1-(3-(Hydroxymethyl)phenyl)-2-methylpropane-1,3-diol (S1m)
Aldol **3m** (19 mg, 0.062 mmol) was subjected to the general procedure for the derivatization of aldols **3** to diols **S1**. Purification by column chromatography with hexanes/AcOEt (1:1 gradient to 1/3) afforded 10 mg (82%) diol **S1m** as a colorless oil.
^{1}H NMR (501 MHz, $CDCl_3$) δ 7.37 – 7.31 (m, 2H), 7.28 – 7.22 (m, 2H), 4.94 (d, *J* = 3.9 Hz, 1H), 4.69 (s, 2H), 3.73 – 3.59 (m, 2H), 2.37 (br. s, 3H), 2.13 – 2.02 (m, 1H), 0.85 (d, *J* = 7.0 Hz, 3H).
^{13}C NMR (126 MHz, $CDCl_3$) δ 143.3, 141.0, 128.6, 126.1, 125.6, 124.8, 76.8, 66.6, 65.5, 41.5, 10.9.
ESI-HRMS: calculated for $C_{11}H_{16}O_3^-$ ($[M–H]^-$): 195.1027; found: 195.1027.
$[\alpha]_D^{25}$ = +27.4 (*c* = 0.27, $CHCl_3$).

R_f = 0.18 (hexanes/AcOEt 1:2).
HPLC (IC-3, *n*-heptane/*i*-PrOH=90:10, 1.0 mL/min, 298 K, 220 nm): t_R(*syn*, minor) = 16.8 min, t_R(*syn*, major) = 20.3 min. e.r. (*syn*) = 93.4:6.6 (86.8% ee)

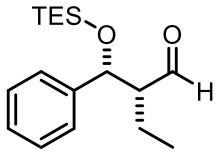

10

(*R*)-2-((*R*)-Phenyl((triethylsilyl)oxy)methyl)butanal (10)
Benzaldehyde (20 µL, 0.2 mmol), IDPi **7c** (4.11 mg, 2 µmol, 1 mol%) and TES enolate **8** (48 µL, 0.24 mmol, 1.2 equiv.) were subjected to the general aldol procedure (Conditions A, –60 °C, 3 days). Purification by column chromatography with hexanes/CH_2Cl_2 (4:1 gradient to 2:1) afforded aldol **10** (47 mg, 80%) as a colorless oil.
^{1}H NMR (501 MHz, $CDCl_3$) δ 9.69 (d, *J* = 2.7 Hz, 1H), 7.35 – 7.21 (m, 5H), 5.02 (d, *J* = 5.5 Hz, 1H), 2.47 (dddd, *J* = 9.5, 5.5, 4.0, 2.7 Hz, 1H), 1.75 (ddq, *J* = 14.7, 9.5, 7.4 Hz, 1H), 1.54 (dddd, *J* = 14.0, 11.6, 7.6, 4.0 Hz, 1H), 0.86 (t, *J* = 7.9 Hz, 13H), 0.51 (qd, *J* = 7.9, 1.4 Hz, 6H).
^{13}C NMR (126 MHz, $CDCl_3$) δ 205.1, 142.51 128.3, 127.7, 126.5, 74.6, 62.0, 17.8, 12.1, 6.8, 4.9.
ESI-HRMS: calculated for $C_{17}H_{28}NaO_2Si^+$ ($[M+Na]^+$): 315.1751; found: 315.1752.
$[\alpha]_D^{25}$ = + 39.1 (*c* = 0.32, $CHCl_3$).
R_f = 0.34 (hexanes/CH_2Cl_2 2:1).

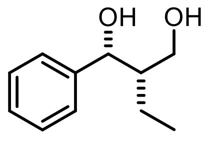

S10

(1*R*,2*S*)-2-Ethyl-1-phenylpropane-1,3-diol (S10)
Aldol **10** (45 mg, 0.15 mmol) was subjected to the general procedure for the derivatization of aldols **3** to diols **S1**. Purification by column chromatography with hexanes/AcOEt (3:1 gradient to 1:1) afforded 24 mg (87%) diol **S10** as a colorless oil.
^{1}H NMR (501 MHz, $CDCl_3$) δ 7.39 – 7.33 (m, 4H), 7.30 – 7.26 (m, 1H), 5.02 (d, *J* = 4.0 Hz, 1H), 3.76 (s, 1H), 3.75 (s, 1H), 2.18 (br. s, 2H), 1.86 (tq, *J* = 6.8, 4.6 Hz, 1H), 1.34 (p, *J* = 7.4 Hz, 2H), 0.90 (t, *J* = 7.5 Hz, 3H).
^{13}C NMR (126 MHz, $CDCl_3$) δ 142. 7, 128.4, 127.5, 126.4, 77.3, 63.9, 48.2, 18.0, 12.1.
ESI-HRMS: calculated for $C_{11}H_{16}NaO_2^+$ ($[M+Na]^+$): 203.1043; found: 203.1042.
$[\alpha]_D^{25}$ = + 36.8 (*c* = 0.26, $CHCl_3$).
R_f= 0.45 (hexanes/AcOEt 1:1).
HPLC (IC-3, *n*-heptane/*i*-PrOH = 90:10, 0.3 mL/min, 298 K, 209 nm): t_R(*syn*, minor) = 18.0 min, t_R(*syn*, major) = 20.8 min. e.r. 95.2:4.8 (90.4% ee).

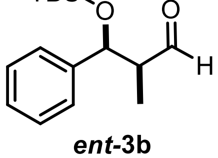

***ent*-3b**

(2*S*,3*S*)-3-((Tert-butyldimethylsilyl)oxy)-2-methyl-3-phenylpropanal (*ent*-3b)
Benzaldehyde (81 µL, 0.8 mmol), (*R*,*R*)-IDPi **7c** (8.23 mg, 4 µmol, 0.5 mol%) and (*E*)-TBS enolate **2b** (202 µL, 0.96 mmol, 1.2 equiv) were subjected to the general aldol procedure (Conditions A, -50 °C, 48 h). Purification by column chromatography with hexanes/CH_2Cl_2 (4:1 gradient to 2:1) afforded aldol ***ent*-3b** (216 mg, 97%) as a colorless oil.

^{1}H NMR (501 MHz, $CDCl_3$) δ 9.76 (d, J = 1.3 Hz, 1H), 7.36 – 7.22 (m, 5H), 5.15 (d, J = 4.3 Hz, 1H), 2.59 (qdd, J = 6.9, 4.3, 1.3 Hz, 1H), 1.04 (d, J = 7.0 Hz, 3H), 0.89 (s, 9H), 0.03 (s, 3H), -0.17 (s, 3H).
^{13}C NMR (126 MHz, $CDCl_3$) δ 204.5, 142.5, 128.3, 127.6, 126.4, 74.4, 54.9, 25.9, 18.3, 8.2, -4.4, -5.1.
ESI-HRMS: calculated for $C_{16}H_{26}NaO_2Si^+$ ($[M+Na]^+$): 301.1594; found: 301.1593.
$[\alpha]_D^{20}$ = –29.8 (c = 1, $CHCl_3$). (Lit.: $[\alpha]_D^{20}$ = −34.4 (c = 1.0, $CHCl_3$).
R_f= 0.38 (hexanes/CH_2Cl_2 3:2).

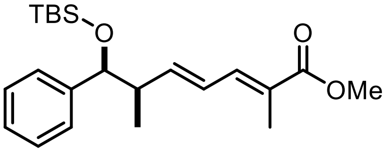

17

Methyl (2*E*,4*E*,6*R*,7*S*)-7-((tert-butyldimethylsilyl)oxy)-2,6-dimethyl-7-phenylhepta-2,4-dienoate (17)

n-Butyllithium (2.5 M in hexane, 150 µL, 0.37 mmol, 1.3 equiv) was added dropwise to a solution of methyl (*E*)-4-(diethoxyphosphoryl)-2-methylbut-2-enoate[4] (93.5 mg, 0.37 mmol, 1.3 equiv) in tetrahydrofuran (2 mL) at -78 °C. After 5 min at -78 °C the solution was warmed to 0 °C and stirred for 30 min at this temperature. Aldehyde ***ent*-3b** (80 mg, 0.29 mmol) in tetrahydrofuran (1 mL) was dropwise at room temperature and the reaction mixture stirred for 15 h at this temperature. The reaction was quenched by addition of saturated aqueous ammonium chloride solution and the aqueous layer extracted with ethyl acetate (x3). The combined organic layers were successively washed with saturated aqueous sodium bicarbonate solution and saturated aqueous sodium chloride solution, dried over $MgSO_4$ and concentrated in vacuo. The residue was purified by flash column chromatography (hexanes/AcOEt 40:1) to provide 95 mg (88%) **17** as a E:Z = 85.5:14.5 mixture of isomers. Data matches the literature, except for a significant deviation of optical rotation.
^{1}H NMR (501 MHz, $CDCl_3$) δ 7.31 – 7.26 (m, 2H), 7.25 – 7.19 (m, 3H), 7.10 (dt, J = 11.2, 1.2 Hz, 1H), 6.22 (ddd, J = 15.2, 11.2, 1.2 Hz, 1H), 5.97 (dd, J = 15.2, 7.7 Hz, 1H), 4.53 (d, J = 5.4 Hz, 1H), 3.74 (s, 3H), 2.61 – 2.52 (m, 1H), 1.88 (d, J = 1.5 Hz, 3H), 1.03 (d, J = 6.7 Hz, 3H), 0.89 (s, 9H), 0.01 (s, 3H), -0.22 (s, 3H).
^{13}C NMR (126 MHz, $CDCl_3$) δ 169.3, 145.3, 143.4, 138.9, 127.9, 127.2, 126.9, 125.9, 125.4, 78.6, 51.9, 46.2, 26.0, 18.4, 14.8, 12.7, -4.5, -4.9.
ESI-HRMS: calculated for $C_{22}H_{34}NaO_3Si^+$ ($[M+Na]^+$): 397.2169; found: 397.2173.
$[\alpha]_D^{20}$ = – 11.4 (c = 1, $CHCl_3$). Lit.[5]: $[\alpha]_D^{20}$ = – 22.3 (c = 1, $CHCl_3$)
R_f = 0.51 (hexanes/AcOEt 20:1).

***anti*-Aldols and their derivatives**

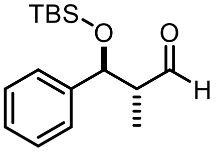

5b

(2*R*,3*S*)-3-((*tert*-Butyldimethylsilyl)oxy)-2-methyl-3-phenylpropanal (5b)

Benzaldehyde (**1a**; 20 µL, 0.2 mmol), IDPi **7d** (6.9 mg, 4 µmol, 2 mol%) and (*Z*)-TBS enolate **4b** (50 µL, 0.24 mmol, 1.2 equiv.) were subjected to the general aldol procedure (Conditions B). Purification by column chromatography with hexanes/CH_2Cl_2 (4:1 gradient to 2:1) afforded aldol **5b** (52 mg, 93%) as a colorless oil.
^{1}H NMR (501 MHz, , $CDCl_3$) δ 9.83 (d, J = 2.7 Hz, 1H), 7.37 – 7.28 (m, 5H), 4.78 (d, J = 7.6 Hz, 1H), 2.75– 2.67 (m, 1H), 0.90 (d, J = 7.0 Hz, 3H), 0.87 (s, 9H), 0.03 (s, 3H), -0.23 (s, 3H).
^{13}C NMR (126 MHz, $CDCl_3$) δ 204.7, 142.4, 128.4, 127.8, 126.8, 77.0, 54.7, 25.8, 18.2, 11.2, -4.4, -5.1.
ESI-HRMS: calculated for $C_{16}H_{26}NaO_2Si^+$ ($[M+Na]^+$): 301.1594; found: 301.1594.
$[\alpha]_D^{25}$ = –102.6 (c = 0.28, $CHCl_3$).
R_f = 0.42 (hexanes/CH_2Cl_2 3:2).

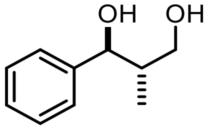

S5b

(1*S*,2*S*)-2-Methyl-1-phenylpropane-1,3-diol (S5b)
Aldol **5b** (33 mg, 0.12 mmol) was subjected to the general procedure for the derivatization of aldols **5** to diols **S5**. Purification by column chromatography with hexanes/AcOEt (3:1 gradient to 1:1) afforded 18 mg (91%) diol **S5b** as a colorless oil.
^{1}H NMR (501 MHz, $CDCl_3$) δ 7.40-7.32 (m, 4H), 7.32 – 7.27 (m, 1H), 4.54 (d, *J* = 8.4 Hz, 1H), 3.77 (dd, *J* = 10.9, 3.6 Hz, 1H), 3.71 (dd, *J* = 10.9, 7.5 Hz, 1H), 2.63 (s, 3H), 2.06 (dpd, *J* = 8.3, 7.1, 3.6 Hz, 1H), 0.71 (d, *J* = 7.0 Hz, 3H).
^{13}C NMR (126 MHz, $CDCl_3$) δ 143.5, 128.6, 128.0, 126.8, 81.0, 68.1, 41.9, 14.0.
EI-HRMS: calculated for $C_{10}H_{14}O_2^{\bullet+}$ ($[M^{\bullet+}]$): 166.0988; found: 166.0989.
$[\alpha]_D^{25}$ = -38.1 (*c* = 0.31, $CHCl_3$) - matches with the *S*,*S*-configured diol from the literature (J. Am. Chem. Soc. 2013, 135, 5316−5319): $[\alpha]_D^{25}$ = –37.5 (*c* = 0.40, $CHCl_3$).
R_f = 0.20 (hexanes/AcOEt = 1:1).
HPLC (IC-3, *n*-heptane/*i*-PrOH=95:5, 0.5 mL/min, 298 K, 209 nm): t_R(*anti*, major) = 29.8, t_R(*anti*, minor) = 42.1 min. e.r. (*anti*) = 98.2:1.8 (96.4% ee).

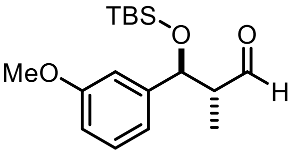

5d

(2*R*,3*S*)-3-((*tert*-Butyldimethylsilyl)oxy)-3-(3-methoxyphenyl)-2-methylpropanal (5d).
3-Methoxylbenzaldehyde (24.4 µL, 0.2 mmol), IDPi **7d** (6.9 mg, 4 µmol, 2 mol%) and (*Z*)-TBS enolate **4b** (50 µL, 0.24 mmol, 1.2 equiv.) were subjected to the general aldol procedure (Conditions B). Purification by column chromatography with hexanes/CH_2Cl_2 (1:1 gradient to 2/3) afforded aldol **5d** (60 mg, 97%) as a colorless oil.
^{1}H NMR (501 MHz, $CDCl_3$) δ 9.80 (d, *J* = 2.7 Hz, 1H), 7.24 (t, *J* = 8.0 Hz, 1H), 6.90 – 6.85 (m, 2H), 6.84 – 6.79 (m, 1H), 4.75 (d, *J* = 7.4 Hz, 1H), 3.81 (s, 3H), 2.72 – 2.64 (m, 1H), 0.90 (d, *J* = 7.0 Hz, 3H), 0.86 (s, 9H), 0.02 (s, 3H), -0.21 (s, 3H).
^{13}C NMR (126 MHz, $CDCl_3$) δ 204.6, 159.8, 144.1, 129.4, 119.2, 113.4, 112.2, 76.8, 55.3, 54.7, 25.8, 18.2, 11.3, -4.3, -5.1.
ESI-HRMS: calculated for $C_{17}H_{28}NaO_3Si^+$ ($[M+Na]^+$): 331.1700; found: 331.1699.
R_f = 0.33 (hexanes/CH_2Cl_2 1:1).
$[\alpha]_D^{25}$ = –88.7 (*c* = 0.30, $CHCl_3$).

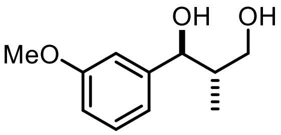

S5d

(1*S*,2*S*)-1-(3-Methoxyphenyl)-2-methylpropane-1,3-diol (S5d)
Aldol **5d** (25 mg, 0.097 mmol) was subjected to the general procedure for the derivatization of aldols **5** to diols **S5**. Purification by column chromatography with hexanes/AcOEt (2:1 gradient to 1:1) afforded 13 mg (82%) diol **S5d** as a colorless oil.
^{1}H NMR (501 MHz, $CDCl_3$) δ 7.31 – 7.19 (m, 1H), 6.96 – 6.86 (m, 2H), 6.83 (ddd, J = 8.2, 2.6, 1.1 Hz, 1H), 4.49 (d, *J* = 8.4 Hz, 1H), 3.81 (s, 3H), 3.74 (dd, J = 10.9, 3.5 Hz, 1H), 3.68 (dd, J = 10.9, 7.6 Hz, 1H), 3.04 (br. s, 2H), 2.09 – 1.95 (m, 1H), 0.70 (d, *J* = 7.0 Hz, 3H).
^{13}C NMR (126 MHz, $CDCl_3$) δ 159.8, 145.2, 129.5, 119.2, 113.4, 112.3, 80.8, 68.0, 55.4, 41.7, 14.0.

ESI-HRMS: calculated for $C_{11}H_{16}NaO_3^+$ ($[M+Na]^+$): 219.0992; found: 219.0991.
$[\alpha]_D^{25}$ = -27.2 (*c* = 0.26, $CHCl_3$).
R_f = 0.3 (EtOAc/hexanes = 3:2).
HPLC (IC-3, *n*-heptane/*i*-PrOH=90:10, 0.5 mL/min, 298 K, 215 nm): t_R(*anti*, major) = 21.8, t_R(*anti*, minor) = 42.6 min. e.r. 96:4 (92% ee).

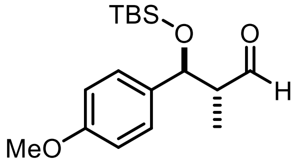

5e

(2*R*,3*S*)-3-((*tert*-Butyldimethylsilyl)oxy)-3-(4-methoxyphenyl)-2-methylpropanal (5e)
4-Methoxybenzaldehyde (24 µL, 0.2 mmol), IDPi **7d** (6.9 mg, 4 µmol, 2 mol%) and (*Z*)-TBS enolate **4b** (50 µL, 0.24 mmol, 1.2 equiv.) were subjected to the general aldol procedure (Conditions B). Purification by column chromatography with hexanes/CH_2Cl_2 (2:1 gradient to 1:1) afforded aldol **5e** (59 mg, 96%) as a colorless oil.
^{1}H NMR (501 MHz, $CDCl_3$) δ 9.80 (d, *J* = 2.8 Hz, 1H), 7.21 (d, *J* = 8.4 Hz, 2H), 6.86 (d, *J* = 8.4 Hz, 2H), 4.71 (d, *J* = 7.8 Hz, 1H), 3.81 (s, 3H), 2.71 – 2.62 (m, 1H), 0.85 (d, *J* = 7.3 Hz, 3H), 0.84 (s, 9H), 0.00 (s, 3H), -0.25 (s, 3H).
^{13}C NMR (126 MHz, $CDCl_3$) δ 204.9, 159.3, 134.6, 128.0, 113.8, 76.6, 55.4, 54.9, 25.9, 18.2, 11.2, -4.3, -5.1.
ESI-HRMS: calculated for $C_{17}H_{28}NaO_3Si^+$ ($[M+Na]^+$): 331.1700; found: 331.1700.
$[\alpha]_D^{25}$ = -77.2 (*c* = 0.32, $CHCl_3$).
R_f = 0.24 (hexanes/CH_2Cl_2 1:1).

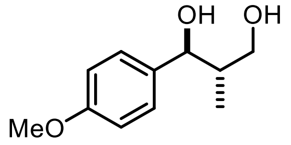

S5e

(1*S*,2*S*)-1-(4-Methoxyphenyl)-2-methylpropane-1,3-diol (S5e)
Aldol **5e** (35 mg, 0.11 mmol) was subjected to the general procedure for the derivatization of aldols **5** to diols **S5**. Purification by column chromatography with hexanes/AcOEt (2:1 gradient to 1:1) afforded 21 mg (94%) diol **S5e** as a colorless solid.
^{1}H NMR (501 MHz, $CDCl_3$) δ 7.26 (d, *J* = 8.6 Hz, 2H), 6.89 (d, *J* = 8.6 Hz, 2H), 4.49 (d, *J* = 8.6f Hz, 1H), 3.81 (s, 3H), 3.76 (dd, *J* = 10.9, 3.6 Hz, 1H), 3.71 (dd, *J* = 10.9, 7.6 Hz, 1H), 2.90 (br. s, 2H), 2.09 – 1.97 (m, 1H), 0.67 (d, *J* = 7.0 Hz, 3H).
^{13}C NMR (126 MHz, $CDCl_3$) δ 159.4, 135.8, 128.0, 114.0, 80.8, 68.3, 55.4, 41.9, 13.8.
ESI-HRMS: calculated for $C_{11}H_{16}NaO_3^+$ ($[M+Na]^+$): 219.0992; found: 219.0994.
$[\alpha]_D^{25}$ = –31.7 (*c* = 0.12, $CHCl_3$).
R_f = 0.22 (hexanes/AcOEt 1:1).
HPLC (IC-3, *n*-heptane/*i*-PrOH=90:10, 1.0 mL/min, 298 K, 225 nm): t_R(*anti*, major) = 12.8, t_R(*anti*, minor) = 15.0 min. e.r. 94.2:5.8 (88.4% ee).

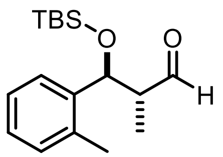

5f

(2*R*,3*S*)-3-((*tert*-Butyldimethylsilyl)oxy)-2-methyl-3-(*o*-tolyl)propanal (5f)

2-Methylbenzaldehyde (23 µL, 0.2 mmol), IDPi **7d** (6.9 mg, 4 µmol, 2 mol%) and (*Z*)-TBS enolate **4b** (50 µL, 0.24 mmol, 1.2 equiv.) were subjected to the general aldol procedure (Conditions B). Purification by column chromatography with hexanes/CH_2Cl_2 (2:1 gradient to 1:1) afforded aldol **5f** (57 mg, 97%) as a colorless oil.
^{1}H NMR (501 MHz, $CDCl_3$) δ 9.85 (d, *J* = 2.6 Hz, 1H), 7.42 (d, *J* = 7.4 Hz, 1H), 7.23-7.14 (m, 2H), 7.12 (d, *J* = 7.1 Hz, 1H), 5.01 (d, *J* = 7.3 Hz, 1H), 2.76 – 2.68 (m, 2.6 Hz, 1H), 2.35 (s, 3H), 0.93 (d, *J* = 7.1 Hz, 3H), 0.85 (s, 9H), 0.01 (s, 3H), -0.27 (s, 3H).
^{13}C NMR (126 MHz, $CDCl_3$) δ 204.7, 140.6, 134.4, 130.6, 127.6, 127.3, 126.3, 73.6, 53.8, 25.8, 19.5, 18.2, 11.4, -4.5, -5.1.
ESI-HRMS: calculated for $C_{17}H_{28}NaO_2Si^+$ ($[M+Na]^+$): 315.1751; found: 315.1755.
$[\alpha]_D^{25}$ = -93.7 (*c* = 0.35, $CHCl_3$).
R_f = 0.40 (hexanes/CH_2Cl_2 3:2).

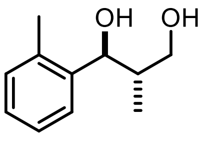

S5f

(1*S*,2*S*)-2-Methyl-1-(*o*-tolyl)propane-1,3-diol (S5f)
Aldol **5f** (30 mg, 0.10 mmol) was subjected to the general procedure for the derivatization of aldols **5** to diols **S5**. Purification by column chromatography with hexanes/AcOEt (3:1 gradient to 1:1) afforded 18 mg (97%) diol **S5f** as a colorless oil.
^{1}H NMR (501 MHz, $CDCl_3$) δ 7.46 (d, *J* = 7.5 Hz, 1H), 7.28 – 7.22 (m, 1H), 7.19 (td, *J* = 7.4, 1.5 Hz, 1H), 7.14 (d, *J* = 7.3 Hz, 1H), 4.87 (d, *J* = 8.2 Hz, 1H), 3.80 (dd, *J* = 10.9, 3.4 Hz, 1H), 3.74 (dd, *J* = 11.0, 7.2 Hz, 1H), 2.64 (br. s, 2H), 2.36 (s, 3H), 2.16 – 2.07 (m, 1H), 0.76 (d, *J* = 7.1 Hz, 3H).
^{13}C NMR (126 MHz, $CDCl_3$) δ 141.6, 135.2, 130.6, 127.7, 126.6, 126.5, 77.0, 68.0, 41.3, 19.6, 13.9.
$[\alpha]_D^{25}$ = -38.3 (*c* = 0.27, $CHCl_3$).
R_f = 0.34 (hexanes/AcOEt 1:1).
HPLC (IC-3, *n*-heptane/*i*-PrOH=95:5, 0.5 mL/min, 298 K, 211 nm): t_R(*anti*, major) = 29.5 min, t_R(*anti*, minor) = 33.6 min. e.r. = 98.5:1.5 (97% ee).

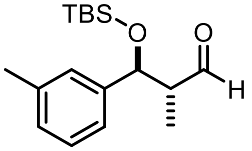

5g

(2*R*,3*S*)-3-((*tert*-Butyldimethylsilyl)oxy)-2-methyl-3-(*m*-tolyl)propanal (5g)
3-Methylbenzaldehyde (24 µL, 0.2 mmol), IDPi **7d** (6.9 mg, 4 µmol, 2 mol%) and (*Z*)-TBS enolate **4b** (50 µL, 0.24 mmol, 1.2 equiv.) were subjected to the general aldol procedure (Conditions B). Purification by column chromatography with hexanes/CH_2Cl_2 (4:1 gradient to 1:1) afforded aldol **5g** (56 mg, 96%) as a colorless oil.
^{1}H NMR (501 MHz, $CDCl_3$) δ 9.81 (d, *J* = 2.7 Hz, 1H), 7.21 (t, *J* = 7.5 Hz, 1H), 7.12 – 7.06 (m, 3H), 4.73 (d, *J* = 7.5 Hz, 1H), 2.72 – 2.64 (m, 1H), 2.35 (s, 3H), 0.89 (d, *J* = 7.0 Hz, 3H), 0.85 (s, 9H), 0.01 (s, 3H), -0.24 (s, 3H).
^{13}C NMR (126 MHz, $CDCl_3$) δ 204.8, 142.4, 138.0, 128.7, 128.3, 127.5, 123.9, 77.0, 54.7, 25.9, 21.6, 18.2, 11.3, -4.3, -5.1.
ESI-HRMS: calculated for $C_{17}H_{28}NaO_2Si^+$ ($[M+Na]^+$): 315.1751; found: 315.1749.
$[\alpha]_D^{25}$ = –99.2 (*c* = 0.36, $CHCl_3$).
R_f = 0.35 (hexanes/CH_2Cl_2 2:1).

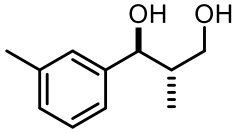

S5g

(1*S*,2*S*)-2-Methyl-1-(*m*-tolyl)propane-1,3-diol (S5g)

Aldol **5g** (35 mg, 0.12 mmol) was subjected to the general procedure for the derivatization of aldols **5** to diols **S5**. Purification by column chromatography with hexanes/AcOEt (2:1 gradient to 1:1) afforded 20 mg (93%) diol **S5g** as a colorless oil.

^{1}H NMR (501 MHz, $CDCl_3$) δ 7.24 (t, *J* = 7.6 Hz, 1H), 7.16 (s, 1H), 7.11 (t, *J* = 7.8 Hz, 2H), 4.49 (d, *J* = 8.4 Hz, 1H), 3.76 (dd, *J* = 10.9, 3.5 Hz, 1H), 3.70 (dd, *J* = 10.9, 7.5 Hz, 1H), 2.99 (br. s, 2H), 2.36 (s, 3H), 2.09 – 1.99 (m, 1H), 0.70 (d, *J* = 7.0 Hz, 3H).

^{13}C NMR (126 MHz, $CDCl_3$) δ 143.5, 138.3, 128.7, 128.5, 127.5, 123.9, 81.1, 68.2, 41.8, 21.6, 14.0.

ESI-HRMS: calculated for $C_{11}H_{16}NaO_2^+$ ($[M+Na]^+$): 203.1043; found: 203.1043.

$[\alpha]_D^{25}$ = -34.3 (*c* = 0.28, $CHCl_3$).

R_f = 0.3 (hexane/AcOEt 1:1, silicagel).

HPLC (IC-3, *n*-heptane/*i*-PrOH=95:5, 1.0 mL/min, 298 K, 215 nm): t_R(*anti*, major) = 15.4, t_R(*anti*, minor) = 27.4 min. e.r. 97.3:2.7 (94.6% ee).

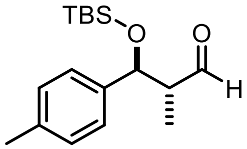

5h

(2*R*,3*S*)-3-((*tert*-Butyldimethylsilyl)oxy)-2-methyl-3-(*p*-tolyl)propanal (5h)

4-Methylbenzaldehyde (24 µL, 0.2 mmol), IDPi **7d** (6.9 mg, 4 µmol, 2 mol%) and (*Z*)-TBS enolate **4b** (50 µL, 0.24 mmol, 1.2 equiv.) were subjected to the general aldol procedure (Conditions B). Purification by column chromatography with hexanes/CH_2Cl_2 (2:1 gradient to 1:1) afforded aldol **5h** (57 mg, 97%) as a colorless oil.

^{1}H NMR (501 MHz, $CDCl_3$) δ 9.80 (d, *J* = 2.7 Hz, 1H), 7.18 (d, *J* = 8.1 Hz, 2H), 7.13 (d, *J* = 8.0 Hz, 2H), 4.73 (d, *J* = 7.6 Hz, 1H), 2.71 – 2.63 (m, 1H), 2.34 (s, 3H), 0.87 (d, *J* = 7.0 Hz, 3H), 0.85 (s, 9H), 0.01 (s, 3H), -0.25 (s, 3H).

^{13}C NMR (126 MHz, $CDCl_3$) δ 204.8, 139.4, 137.6, 129.1, 126.7, 76.8, 54.8, 25.9, 21.3, 18.2, 11.2, -4.3, -5.1.

ESI-HRMS: calculated for $C_{17}H_{28}NaO_2Si^+$ ($[M+Na]^+$): 315.1751; found: 315.1751.

$[\alpha]_D^{25}$ = -94.3 (*c* = 0.35 $CHCl_3$).

R_f= 0.34 (silicagel, hexanes/CH_2Cl_2 2:1).

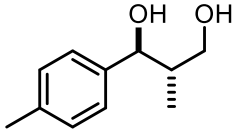

S5h

(1*S*,2*S*)-2-Methyl-1-(*p*-tolyl)propane-1,3-diol

Aldol **5h** (40 mg, 0.14 mmol) was subjected to the general procedure for the derivatization of aldols **5** to diols **S5**. Purification by column chromatography with hexanes/AcOEt (3:1 gradient to 1:1) afforded 21 mg (85%) diol **S5h** as a colorless oil.

^{1}H NMR (501 MHz, $CDCl_3$) δ 7.23 (d, *J* = 8.2 Hz, 2H), 7.16 (d, *J* = 7.8 Hz, 2H), 4.50 (d, *J* = 8.5 Hz, 1H), 3.75 (dd, *J* = 10.9, 3.6 Hz, 1H), 3.70 (dd, *J* = 10.9, 7.5 Hz, 1H), 2.64 (s, 3H), 2.35 (s, 3H), 2.04 (dddt, *J* = 10.6, 7.1, 4.8, 3.5 Hz, 1H), 0.69 (d, *J* = 7.0 Hz, 3H).

^{13}C NMR (126 MHz, $CDCl_3$) δ 140.6, 137.7, 129.3, 126.7, 80.9, 68.2, 41.8, 21.3, 14.0.

ESI-HRMS: calculated for $C_{11}H_{16}NaO_2^+$ ($[M+Na]^+$): 203.1043; found: 203.1045.

$[\alpha]_D^{25}$ = -40.0 (*c* = 0.23, $CHCl_3$).

R_f = 0.30 (hexanes/AcOEt 1:1).

HPLC (IC-3, *n*-heptane/*i*-PrOH = 95:5, 1.0 mL/min, 298 K, 213 nm): t_R(*anti*, major) = 18.9 min, t_R(*anti*, minor) = 27.3 min. e.r. = 97:3 (94% ee).

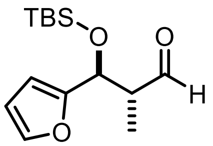

5i

(2*R*,3*S*)-3-((*tert*-Butyldimethylsilyl)oxy)-3-(furan-2-yl)-2-methylpropanal (5i)

Furfural (17 µL, 0.2 mmol), IDPi **7d** (6.9 mg, 4 µmol, 2 mol%) and (*Z*)-TBS enolate **4b** (50 µL, 0.24 mmol, 1.2 equiv.) were subjected to the general aldol procedure (Conditions B). Purification by column chromatography with hexanes/CH_2Cl_2 (3:1 gradient to 2:1) afforded aldol **5i** (20 mg, 37%) as a colorless oil.

*Note: Changing the order of addition, i.e. mixing IDPi **7d** with enolsilane **4b** overnight at –78 °C, followed by careful addition of furfural improved the ^{1}H NMR yield to 55%, which was the highest observed yield for **5i**.*

^{1}H NMR (501 MHz, $CDCl_3$) δ 9.84 (d, *J* = 2.3 Hz, 1H), 7.38 (dd, *J* = 1.9, 0.9 Hz, 1H), 6.33 (dd, *J* = 3.2, 1.8 Hz, 1H), 6.24 (d, *J* = 3.2 Hz, 1H), 4.84 (d, *J* = 7.2 Hz, 1H), 2.93 – 2.85 (m, 1H), 0.94 (d, *J* = 7.1 Hz, 3H), 0.84 (s, 9H), 0.03 (s, 3H), -0.15 (s, 3H).

^{13}C NMR (126 MHz, $CDCl_3$) δ 204.1, 154.6, 142.2, 110.3, 107.9, 70.3, 51.9, 25.8, 18.2, 10.9, -4.9, -5.2.

ESI-HRMS: calculated for $C_{14}H_{24}NaO_3Si^+$ ($[M+Na]^+$): 291.1387; found: 291.1384.

$[\alpha]_D^{25}$ = -41.1 (*c* = 0.18, $CHCl_3$).

R_f = 0.56 (silicagel, hexanes/CH_2Cl_2 1:1).

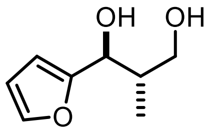

S5i

(1*S*,2*S*)-1-(Furan-2-yl)-2-methylpropane-1,3-diol (S5i)

Aldol **5i** (16 mg, 0.06 mmol) was subjected to the general procedure for the derivatization of aldols **5** to diols **S5**. Purification by column chromatography with hexanes/AcOEt (3:1 gradient to 1:1) afforded 8 mg (86%) diol **S5i** as a pale yellow oil.

^{1}H NMR (501 MHz, $CDCl_3$) δ 7.39 (dd, *J* = 1.9, 0.9 Hz, 1H), 6.35 (dd, *J* = 3.2, 1.8 Hz, 1H), 6.29 (d, *J* = 3.2 Hz, 1H), 4.62 (d, *J* = 8.4 Hz, 1H), 3.81 (dd, *J* = 11.0, 3.6 Hz, 1H), 3.71 (dd, *J* = 10.9, 7.2 Hz, 1H), 2.26 (dpd, *J* = 8.4, 7.0, 3.6 Hz, 1H), 2.09 (br. s, 2H), 0.80 (d, *J* = 7.0 Hz, 3H).

^{13}C NMR (126 MHz, $CDCl_3$) δ 155.8, 142.2, 110.3, 107.2, 73.5, 67.5, 39.8, 13.7.

ESI-HRMS: calculated for $C_8H_{12}O_3Na^+$ ($[M+Na]^+$): 179.0679; found: 179.0680.

$[\alpha]_D^{25}$ = -2.8 (*c* = 0.25, $CHCl_3$). Lit. $[\alpha]_D^{25}$ = -2.2 (*c* = 0.1, CH_2Cl_2). − anti/syn 83:17, 82% ee.

HPLC (IC-3, *n*-heptane/*i*-PrOH = 95:5, 1.0 mL/min, 298 K, 215 nm): t_R(*anti*, major) = 10.8 min, t_R(*anti*, minor) = 19.5 min. e.r. = 94:6 (88% ee).

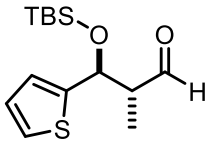

5j

(2*R*,3*S*)-3-((*tert*-Butyldimethylsilyl)oxy)-2-methyl-3-(thiophen-2-yl)propanal (5j)

Thiophen-2-carbaldehyde (19 µL, 0.2 mmol), IDPi **7d** (6.9 mg, 4 µmol, 2 mol%) and (*Z*)-TBS enolate **4b** (50 µL, 0.24 mmol, 1.2 equiv.) were subjected to the general aldol procedure (Conditions B). Purification by column chromatography with hexanes/CH_2Cl_2 (2:1 gradient to 1:1) afforded aldol **5j** (55 mg, 97%) as a colorless oil.

^{1}H NMR (501 MHz, $CDCl_3$) δ 9.82 (d, *J* = 2.5 Hz, 1H), 7.28-7.23 (m, 1H), 6.94 (dd, *J* = 4.9, 3.5 Hz, 1H), 6.93 – 6.90 (m, 1H), 5.08 (d, *J* = 7.5 Hz, 1H), 2.80 – 2.71 (m, 1H), 0.95 (d, *J* = 7.1 Hz, 3H), 0.86 (s, 9H), 0.05 (s, 3H), -0.15 (s, 3H).
^{13}C NMR (126 MHz, $CDCl_3$) δ 204.0, 146.7, 126.4, 125.2, 124.6, 72.8, 55.2, 25.8, 18.2, 11.1, -4.6, -5.1.
ESI-HRMS: calculated for $C_{14}H_{24}NaO_2SSi^+$ ($[M+Na]^+$): 307.1158; found: 307.1159.
$[\alpha]_D^{25}$ = -100.9 (*c* = 0.44, $CHCl_3$).
R_f = 0.48 (hexanes/CH_2Cl_2 2:1, silicagel).

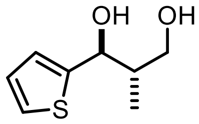

S5j

(1*S*,2*S*)-2-Methyl-1-(thiophen-2-yl)propane-1,3-diol (S5j)
Aldol **5j** (45 mg, 0.16 mmol) was subjected to the general procedure for the derivatization of aldols **5** to diols **S5**. Purification by column chromatography with hexanes/AcOEt (3:1 gradient to 1:1) afforded 25 mg (92%) diol **S5j** as a colorless solid.
^{1}H NMR (126 MHz, $CDCl_3$) δ 7.28 (d, *J* = 4.4 Hz, 2H), 7.04-6.89 (m, 2H), 4.84 (d, *J* = 8.5 Hz, 1H), 3.82 (dd, *J* = 11.0, 3.5 Hz, 2H), 3.72 (dd, *J* = 11.0, 7.5 Hz, 2H), 2.88 (br. s, 2H), 2.15 – 2.05 (m, 1H), 0.79 (d, *J* = 7.0 Hz, 3H).
^{13}C NMR (126 MHz, $CDCl_3$) δ 147.5, 126.6, 125.1, 124.8, 76.3, 67.8, 42.7, 14.0.
ESI-HRMS: calculated for $C_8H_{12}NaO_2S^+$ ($[M+Na]^+$): 195.0450; found: 195.0452.
$[\alpha]_D^{25}$ = -17.7 (*c* = 0.31 $CHCl_3$).
R_f= 0.34 (silicagel, hexanes/CH_2Cl_2 2:1)
HPLC (IC-3, *n*-heptane/*i*-PrOH=90:10, 0.5 mL/min, 298 K, 234 nm): t_R(*anti*, major) = 15.9, t_R(*anti*, minor) = 23.6 min. e.r. 93:7 (86% ee).

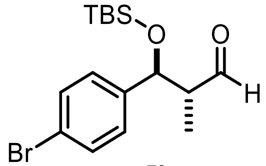

5k

(2*R*,3*S*)-3-(4-Bromophenyl)-3-((*tert*-butyldimethylsilyl)oxy)-2-methylpropanal (5k)
4-Bromobenzaldehyde (37 mg, 0.2 mmol), IDPi **7d** (6.9 mg, 4 µmol, 2 mol%) and (*Z*)-TBS enolate **4b** (50 µL, 0.24 mmol, 1.2 equiv.) were subjected to the general aldol procedure (Conditions B). Purification by column chromatography with hexanes/CH_2Cl_2 (3:1 gradient to 3:2) afforded aldol **5k** (70 mg, 98%) as a colorless oil.
^{1}H NMR (501 MHz, $CDCl_3$) δ 9.78 (d, *J* = 2.6 Hz, 1H), 7.47 (d, *J* = 8.4 Hz, 2H), 7.18 (d, *J* = 8.4 Hz, 2H), 4.74 (d, *J* = 7.5 Hz, 1H), 2.69 – 2.61 (m, 1H), 0.88 (d, *J* = 7.1 Hz, 3H), 0.85 (s, 9H), 0.02 (s, 3H), -0.23 (s, 3H).
^{13}C NMR (126 MHz, $CDCl_3$) δ 204.1, 141.5, 131.6, 128.5, 121.8, 76.2, 54.6, 25.8, 18.2, 11.1, -4.3, -5.1.
ESI-HRMS: calculated for $C_{16}H_{25}BrNaO_2Si^+$ ($[M+Na]^+$): 379.0699; found: 379.0670.
$[\alpha]_D^{25}$ = –87.3 (*c* = 0.55, $CHCl_3$).
R_f = 0.30 (silicagel, hexanes/CH_2Cl_2 2:1).

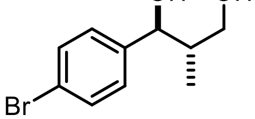

S5k

(1*S*,2*S*)-1-(4-Bromophenyl)-2-methylpropane-1,3-diol (S5k)

Aldol **5m** (55 mg, 0.14 mmol) was subjected to the general procedure for the derivatization of aldols **5** to diols **S5**. Purification by column chromatography with hexanes/AcOEt (3:1 gradient to 1:1) afforded 32 mg (94%) diol **S5k** as a colorless solid. **Crystallization for X-ray analysis was achieved via slow diffusion of hexanes to a solution of* ***S5k*** *in ethylacetate.*

^{1}H NMR (501 MHz, $CDCl_3$) δ 7.48 (d, *J* = 8.4 Hz, 2H), 7.22 (d, *J* = 8.3 Hz, 2H), 4.51 (d, *J* = 8.2 Hz, 1H), 3.77 (dd, *J* = 10.8, 3.5 Hz, 1H), 3.68 (dd, *J* = 10.9, 7.6 Hz, 1H), 2.79 (br. s, 2H), 2.03 – 1.93 (m, 1H), 0.70 (d, *J* = 7.0 Hz, 3H).
^{13}C NMR (126 MHz, $CDCl_3$) δ 142.5, 131.7, 128.6, 121.7, 80.2, 67.9, 41.8, 13.9.
ESI-HRMS: calculated for $C_{10}H_{13}BrNaO_2^+$ ($[M+Na]^+$): 266.9991; found: 266.9991.
$[\alpha]_D^{25}$ = –26.0 (*c* = 0.20, $CHCl_3$).
R_f = 0.28 (hexanes/AcOEt = 1:1).
m.p. 91-93 °C
HPLC (IC-3, *n*-heptane/*i*-PrOH = 95:5, 0.5 mL/min, 298 K, 220 nm): t_R(*anti*, major) = 22.5 min, t_R(*anti*, minor) = 24.2 min. e.r. = 97:3 (94% ee).

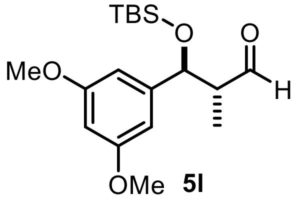

5l

(2*R*,3*S*)-3-((*tert*-Butyldimethylsilyl)oxy)-3-(3,5-dimethoxyphenyl)-2-methylpropanal (5l)
3,5-Dimethoxybenzaldehyde (33 mg, 0.2 mmol), IDPi **7d** (6.9 mg, 4 µmol, 2 mol%) and (*Z*)-TBS enolate **4b** (50 µL, 0.24 mmol, 1.2 equiv.) were subjected to the general aldol procedure (Conditions B). Purification by column chromatography with hexanes/AcOEt (25/1 gradient to 15/1) afforded aldol **5l** as a 90:10 mixture of diastereomers (68 mg, 90%) as a colorless oil. Small amount of analytically pure *anti*-diastereomer was obtained by careful repurification.
^{1}H NMR (501 MHz, $CDCl_3$) δ 9.77 (d, *J* = 2.7 Hz, 1H), 6.47 (d, *J* = 2.3 Hz, 2H), 6.36 (t, *J* = 2.3 Hz, 1H), 4.69 (d, *J* = 7.4 Hz, 1H), 3.78 (s, 6H), 2.70 – 2.62 (m, 1H), 0.91 (d, *J* = 7.0 Hz, 3H), 0.86 (s, 9H), 0.02 (s, 3H), -0.19 (s, 3H).
^{13}C NMR (126 MHz, $CDCl_3$) δ 204.7, 160.7, 144.9, 104.6, 99.6, 76.8, 55.5, 54.6, 25.8, 18.2, 11.4, -4.4, -5.1.
ESI-HRMS: calculated for $C_{18}H_{31}O_4Si+^+$ ($[M+H]^+$): 339.1986; found: 339.1983.
$[\alpha]_D^{25}$ = -66.7 (*c* = 0.32, $CHCl_3$).
R_f = 0.30 (hexanes/AcOEt 15/1).

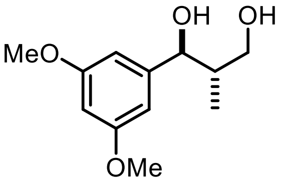

S5l

(1*S*,2*S*)-1-(3,5-Dimethoxyphenyl)-2-methylpropane-1,3-diol (S5l)
Aldol **5l** (35 mg, 0.103 mmol) was subjected to the general procedure for the derivatization of aldols **5** to diols **S5**. Purification by column chromatography with hexanes/AcOEt (3:1 gradient to 1:1) afforded 22 mg (94%) diol **S5l** as a colorless oil.
^{1}H NMR (501 MHz, $CDCl_3$) δ 6.50 (d, *J* = 2.3 Hz, 2H), 6.38 (t, *J* = 2.3 Hz, 1H), 4.46 (d, *J* = 8.3 Hz, 1H), 3.79 (s, 6H), 3.76 (dd, *J* = 10.9, 3.6 Hz, 1H), 3.69 (dd, *J* = 10.8, 7.4 Hz, 1H), 3.08 (br. s, 2H), 2.08 – 1.98 (m, 1H), 0.73 (d, *J* = 7.0 Hz, 1H).
^{13}C NMR (126 MHz, $CDCl_3$) δ 170.0, 146.0, 104.8, 99.8, 81.0, 68.0, 55.5, 41.7, 14.0.
ESI-HRMS: calculated for $C_{12}H_{18}NaO_4^+$ ($[M+Na]^+$): 249.1097; found: 249.1095.
$[\alpha]_D^{25}$ = -16.9 (*c* = 0.24, $CHCl_3$).
R_f = 0.20 (silicagel, hexanes/AcOEt 1:1)
HPLC (Amycoat RP, Acetonitrile/Water = 40:60, 1.0 mL/min, 298 K, 220 nm): t_R(*anti*, minor) = 4.4 min, t_R(*anti*, major) = 7.6 min. e.r. = 11:89 (78% ee).

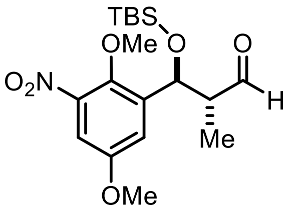

5m

(2*R*,3*S*)-3-((*tert*-Butyldimethylsilyl)oxy)-3-(2,5-dimethoxy-3-nitrophenyl)-2-methylpropanal (5m)

2,5-Dimethoxy-3-nitrobenzaldehyde[6] (42 mg, 0.2 mmol), IDPi **7d** (6.9 mg, 4 µmol, 2 mol%) and (*Z*)-TBS enolate **4b** (50 µL, 0.24 mmol, 1.2 equiv.) were subjected to the general aldol procedure (Conditions B, **–20 C°***). Purification by column chromatography with hexanes/AcOEt (25/1) afforded aldol **5m** as a 89:11 mixture of diastereomers (73 mg, 95%) as a colorless oil.

Surprisingly, no reaction was observed at temperatures below –20 C° which is in agreement with the extremely tight catalytic pocket of IDPi* ***7d**, and considering that the corresponding aldehyde is the bulkiest of all the substrates used.*

^{1}H NMR (501 MHz, CDCl$_3$) δ 9.73 (d, *J* = 2.3 Hz, 1H), 7.31 (d, *J* = 3.3 Hz, 1H), 7.29 (d, *J* = 3.1 Hz, 1H), 5.22 (d, *J* = 5.7 Hz, 1H), 3.87 (s, 3H), 3.83 (s, 3H), 2.72 – 2.65 (m, 1H), 1.04 (d, *J* = 7.1 Hz, 3H), 0.89 (s, 9H), 0.11 (s, 3H), -0.13 (s, 3H).

^{13}C NMR (126 MHz, CDCl$_3$) δ 203.1, 155.2, 143.9, 143.4, 118.7, 109.5, 70.2, 62.5, 56.0, 53.6, 25.7, 18.1, 11.4, -4.5, -5.1.

ESI-HRMS: calculated for $C_{18}H_{29}NNaO_6Si^+$ ([M+Na]$^+$): 406.1656; found 406.1657.

$[\alpha]_D^{25}$ = -42.0 (*c* = 0.20, CHCl$_3$) - 89:11 *anti/syn* mixture

R$_f$ (*anti*) =0.24 (hexanes/AcOEt 15:1).

R$_f$ (*syn*) =0.29 (hexanes/AcOEt 15:1).

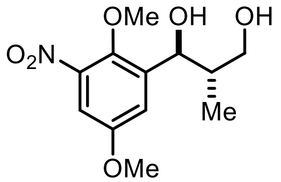

S5m

(1*S*,2*S*)-1-(2,5-Dimethoxy-3-nitrophenyl)-2-methylpropane-1,3-diol (S5m)

Aldol **5m** (35 mg, 0.091 mmol, d.r. 89:11) was subjected to the general procedure for the derivatization of aldols **5** to diols **S5**. Purification by column chromatography with hexanes/AcOEt (1:1 gradient to 1:2) afforded 19 mg (77%) diol **S5m** as a yellow gum as a 90:10 mixture of anti/syn diastereomers.

^{1}H NMR (501 MHz, CDCl$_3$) δ 7.30 (d, *J* = 3.3 Hz, 1H), 7.29 (d, *J* = 3.4 Hz, 1H), 5.00 (d, *J* = 7.6 Hz, 1H), 3.87 (s, 3H), 3.84 (s, 3H), 3.81 (dd, *J* = 10.9, 3.5 Hz, 1H), 3.70 (dd, *J* = 10.9, 7.3 Hz, 1H), 2.78 (br. s, 2H), 2.15 – 2.04 (m, 1H), 0.81 (d, *J* = 7.0 Hz, 3H).

^{13}C NMR (126 MHz, CDCl$_3$) δ 155.5, 144.6, 143.7, 140.6, 119.0, 109.0, 73.8, 67.6, 63.2, 56.0, 40.9, 13.6.

ESI-HRMS: calculated for $C_{12}H_{17}NNaO_6^+$ ([M+Na]$^+$): 294.0948; found 294.0947.

R$_f$ = 0.17 (hexanes/AcOEt 1:2)

HPLC (Chiralpak IB N-3, *n*-heptane/*i*-PrOH = 95:5, 1.0 mL/min, 298 K, 220 nm): t$_R$(*anti*, major) = 16.3 min, t$_R$(*anti*, minor) = 17.7 min. e.r. = 86.77:13.23 (73.5% ee).

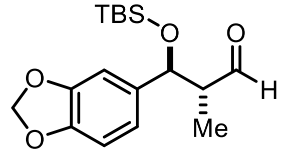

5n

(2*R*,3*S*)-3-(Benzo[d][1,3]dioxol-5-yl)-3-((*tert*-butyldimethylsilyl)oxy)-2-methylpropanal (5n)

Piperonal (30 mg, 0.2 mmol), IDPi **7d** (3.44 mg, 2 µmol, 1 mol%) and (*Z*)-TBS enolate **4b** (50 µL, 0.24 mmol, 1.2 equiv.) were subjected to the general aldol procedure (Conditions B). Purification by column chromatography with hexanes/AcOEt (25/1 gradient to 15/1) afforded aldol **5n** (61 mg, 95%) as a colorless oil.
^{1}H NMR (501 MHz, $CDCl_3$) δ 9.79 (d, *J* = 2.7 Hz, 1H), 6.82 (d, *J* = 1.6 Hz, 1H), 6.75 (d, *J* = 7.9 Hz, 1H), 6.72 (dd, *J* = 7.9, 1.6 Hz, 1H), 5.97 (d, *J* = 1.5 Hz, 1H), 5.96 (d, *J* = 1.5 Hz, 1H), 4.67 (d, *J* = 7.8 Hz, 1H), 2.69 – 2.60 (m, 1H), 0.86 (d, *J* = 7.1 Hz, 4H), 0.85 (s, 9H), 0.01 (s, 3H), -0.21 (s, 3H).
^{13}C NMR (126 MHz, $CDCl_3$) δ 204.6, 147.9, 147.3, 136.5, 120.3, 108.0, 107.0, 101.2, 76.8, 54.8, 25.8, 18.2, 11.2, -4.3, -5.1.
ESI-HRMS: calculated for $C_{17}H_{26}NaO_4Si^+$ ($[M+Na]^+$): 345.1493; found: 345.1491.
$[\alpha]_D^{25}$ = –73.6 (*c* = 0.26, $CHCl_3$).
R_f=0.23 (silicagel, hexanes/AcOEt 15:1).

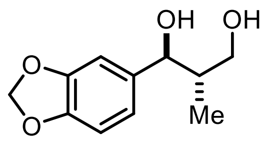

S5n

(1*S*,2*S*)-1-(Benzo[d][1,3]dioxol-5-yl)-2-methylpropane-1,3-diol (S5n)
Aldol **5n** (25 mg, 0.08 mmol) was subjected to the general procedure for the derivatization of aldols **5** to diols **S5**. Purification by column chromatography with hexanes/AcOEt (2:1 gradient to 1:1) afforded 14 mg (86%) diol **S5n** as a colorless solid.
^{1}H NMR (501 MHz, $CDCl_3$) δ 6.87 (s, 1H), 6.77 (d, *J* = 1.0 Hz, 2H), 5.96 (s, 2H), 4.45 (d, *J* = 8.6 Hz, 1H), 3.77 (dd, *J* = 10.9, 3.6 Hz, 1H), 3.70 (dd, *J* = 10.9, 7.7 Hz, 1H), 2.59 (br. s, 3H), 2.06 – 1.95 (m, 1H), 0.69 (d, *J* = 6.9 Hz, 3H).
^{13}C NMR (126 MHz, $CDCl_3$) δ 148.0, 147.3, 137.6, 120.4, 108.1, 107.1, 101.2, 80.9, 68.3, 41.9, 14.0.
ESI-HRMS: calculated for $C_{11}H_{14}NaO_4^+$ ($[M+Na]^+$): 233.0784; found: 233.0785.
$[\alpha]_D^{25}$ = -24.8 (*c* = 0.2, $CHCl_3$).
R_f = 0.21 (silicagel, hexanes/AcOEt 1:1).
HPLC (IC-3, *n*-heptane/*i*-PrOH = 90:10, 1.0 mL/min, 298 K, 220 nm): t_R(*anti*, major) = 13.1 min, t_R(*anti*, minor) = 19.5 min. e.r. = 95:5 (90% ee).

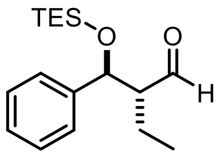

11

(*R*)-2-((*S*)-Phenyl((triethylsilyl)oxy)methyl)butanal (11)
Benzaldehyde (**1a**; 20 µL, 0.2 mmol), IDPi **7d** (6.9 mg, 4 µmol, 2 mol%) and (*Z*)-TES enolate **9** (51 µL, 0.24 mmol, 1.2 equiv.) were subjected to the general aldol procedure (Conditions B). Purification by column chromatography with hexanes/CH_2Cl_2 (3:1 gradient to 3:2) afforded aldol **11** (57 mg, 97%) as a colorless oil.
^{1}H NMR (501 MHz, $CDCl_3$) δ 9.73 (d, *J* = 3.8 Hz, 1H), 7.36 – 7.23 (m, 5H), 4.84 (d, *J* = 7.6 Hz, 1H), 2.48 (ddt, *J* = 9.7, 7.9, 4.1 Hz, 1H), 1.55 (ddq, *J* = 14.7, 9.7, 7.4 Hz, 1H), 1.30 – 1.19 (m, 1H), 0.85 – 0.77 (m, 12H), 0.46 (qd, *J* = 7.9, 4.1 Hz, 6H).
^{13}C NMR (126 MHz, $CDCl_3$) δ 204.6, 142.7, 128.4, 128.0, 126.9, 75.7, 62.2, 19.6, 11.7, 6.8, 4.9.
ESI-HRMS: calculated for $C_{17}H_{28}NaO_2Si^+$ ($[M+Na]^+$): 315.1751; found: 315.1749.
$[\alpha]_D^{25}$ = -63.0 (*c* = 0.60, $CHCl_3$).
R_f = 0.34 (hexanes/CH_2Cl_2 2:1).

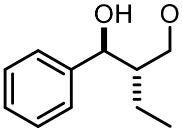

S11

(1*S*,2*S*)-2-Ethyl-1-phenylpropane-1,3-diol (S11)

Aldol **11** (44 mg, 0.15 mmol) was subjected to the general procedure for the derivatization of aldols **5** to diols **S5**. Purification by column chromatography with hexanes/AcOEt (3:1 gradient to 1:1) afforded 25 mg (92%) diol **S11** as a colorless oil.

^{1}H NMR (501 MHz, $CDCl_3$) δ 7.40-7.33 (m, 4H), 7.32 – 7.27 (m, 1H), 4.71 (dd, *J* = 7.2, 1.5 Hz, 1H), 3.85 (dt, *J* = 10.9, 1.9 Hz, 1H), 3.71 (ddd, *J* = 11.0, 6.4, 1.6 Hz, 1H), 2.96 (br. s, 1H), 2.86 (br. s, 1H), 1.79 – 1.72 (m, 1H), 1.37 – 1.22 (m, 2H), 0.87 (t, *J* = 7.5 Hz, 3H).

^{13}C NMR (126 MHz, $CDCl_3$) δ 143.7, 128.6, 127.8, 126.6, 79.2, 64.4, 48.3, 21.2, 11.8.

ESI-HRMS: calculated for $C_{11}H_{16}NaO_2^+$ ($[M+Na]^+$): 203.1043; found: 203.1041.

$[\alpha]_D^{25}$ = -49.5 (*c* = 0.21, $CHCl_3$).

R_f = 0.48 (hexanes/AcOEt 1:1).

HPLC (IC-3, *n*-heptane/*i*-PrOH = 90:10, 0.3 mL/min, 298 K, 209 nm): t_R(*anti*, major) = 22.2 min, t_R(*anti*, minor) = 25.2 min. e.r. = 95:5 (90% ee).

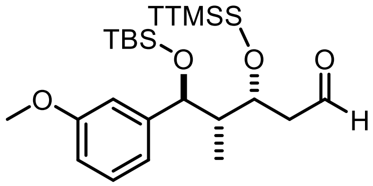

16

(3*R*,4*S*,5*S*)-5-((*tert*-Butyldimethylsilyl)oxy)-3-((1,1,1,3,3,3-hexamethyl-2-(trimethylsilyl)trisilan-2-yl)oxy)-5-(3-methoxyphenyl)-4-methylpentanal (16)

A flame dried Schlenk flask was charged with the acetaldehyde supersilyl enolate of acetaldehyde[7] (38.5 mg, 0.13 mmol) under Ar. A solution of aldol **5d** (34 mg, 0.11 mmol) in dry CH_2Cl_2 (1.1 mL) was added and the solution was cooled to -78 °C. To this mixture was added the stock solution of Tf_2NH in CH_2Cl_2 (2.2 µL, 1.1 µmol, 1 mol%) at -78 °C. Immediately a yellow color appears and the reaction mixture was stirred at -78 °C under Ar. After 2 h at -78 °C the yellow color disappeared and the TLC showed full conversion. The reaction mixture was diluted with excess CH_2Cl_2 and quenched with water and the aqueous phase extracted with CH_2Cl_2. The organic phases were dried with Na_2SO_4 and filtered. Evaporation of the filtrate gave a colorless oil which was dried under high vacuum for analysis of the crude ^{1}H NMR, which showed that the diastereoselectivity was 3:1. Purification by column chromatography (silicagel, hexanes/CH_2Cl_2 3:1 to 1:1) 53 mg (80 %) dialdolate **16** as an inseparable 3:1 mixture of diastereomers.

Major diastereomer:

^{1}H NMR (501 MHz, $CDCl_3$) δ 9.75 (t, *J* = 2.6 Hz, 1H), 7.23 – 7.15 (m, 1H), 6.84 – 6.74 (m, 3H), 4.54 (d, *J* = 7.2 Hz, 1H), 4.08 (ddd, *J* = 8.1, 5.1, 3.2 Hz, 1H), 3.79 (s, 3H), 2.69 (ddd, *J* = 15.4, 3.3, 2.3 Hz, 1H), 2.57 (ddd, *J* = 15.4, 8.0, 3.1 Hz, 1H), 1.96 – 1.87 (m, 1H), 0.85 (s, 9H), 0.65 (d, *J* = 7.0 Hz, 3H), 0.19 (s, 27H), 0.02 (s, 3H), -0.33 (s, 3H).

^{13}C NMR (126 MHz, $CDCl_3$) δ 202.05, 159.39, 144.88, 129.05, 119.83, 112.78, 112.77, 76.41, 72.10, 55.29, 48.84, 47.06, 29.86, 26.02, 11.13, 0.71, -4.25, -4.70.

Minor diastereomer (detectable non-overlapping resonances):

^{1}H NMR (501 MHz, $CDCl_3$) δ 9.82 (t, *J* = 2.7 Hz, 1H), 4.52 – 4.47 (m, 1H), 4.19 (d, *J* = 9.0 Hz, 1H), 3.79 (s, 3H), 2.49 – 2.43 (m, 2H), 2.15 (dqd, *J* = 13.9, 6.9, 3.9 Hz, 1H), 0.82 (s, 9H), 0.60 (d, *J* = 6.9 Hz, 3H), 0.18 (s, 27H), 0.03 (s, 3H), -0.33 (s, 3H). ^{13}C NMR (126 MHz, $CDCl_3$) δ 203.30, 159.58, 145.34, 129.25, 119.76, 113.67, 111.86, 78.82, 71.71, 55.24, 46.03, 45.30, 25.96, 10.14, 0.69, -4.31, -4.80.

ESI-HRMS: calculated for $C_{28}H_{58}NaO_4Si_5^+$ ($[M+Na]^+$): 621.3074; found: 621.3073.

R_f = 0.42 (hexanes/CH_2Cl_2 1:1)

Determination of stereochemistry of dialdolate **16**: A small amount of dialdolate **16** was converted to acetonide **S16** via a (low yielding) sequence of Wittig reaction/desilylation/acetonide formation in analogy to Brady and Yamamoto.[8] ^{13}C NMR analysis of the mixture of acetonides allowed to assign the configuration of the major product according to the following diagnostic chemical shifts in analogy to the literature.[9]

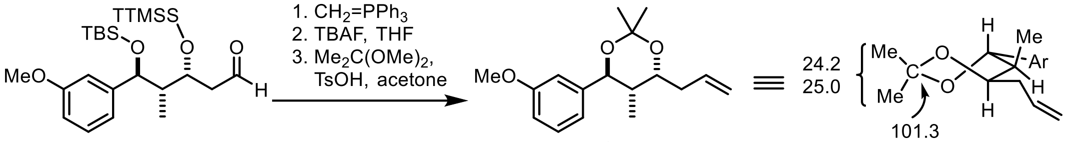

Ref. 11:

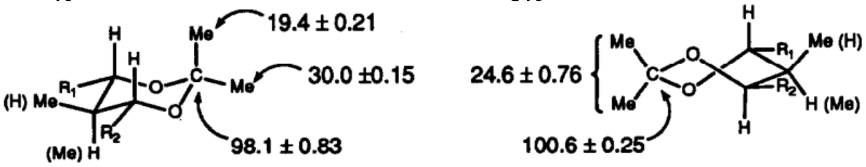

Figure. Average Values for ^{13}C NMR Resonances of *Syn* and *Anti* Polypropionate Polyols (ave ± σ).

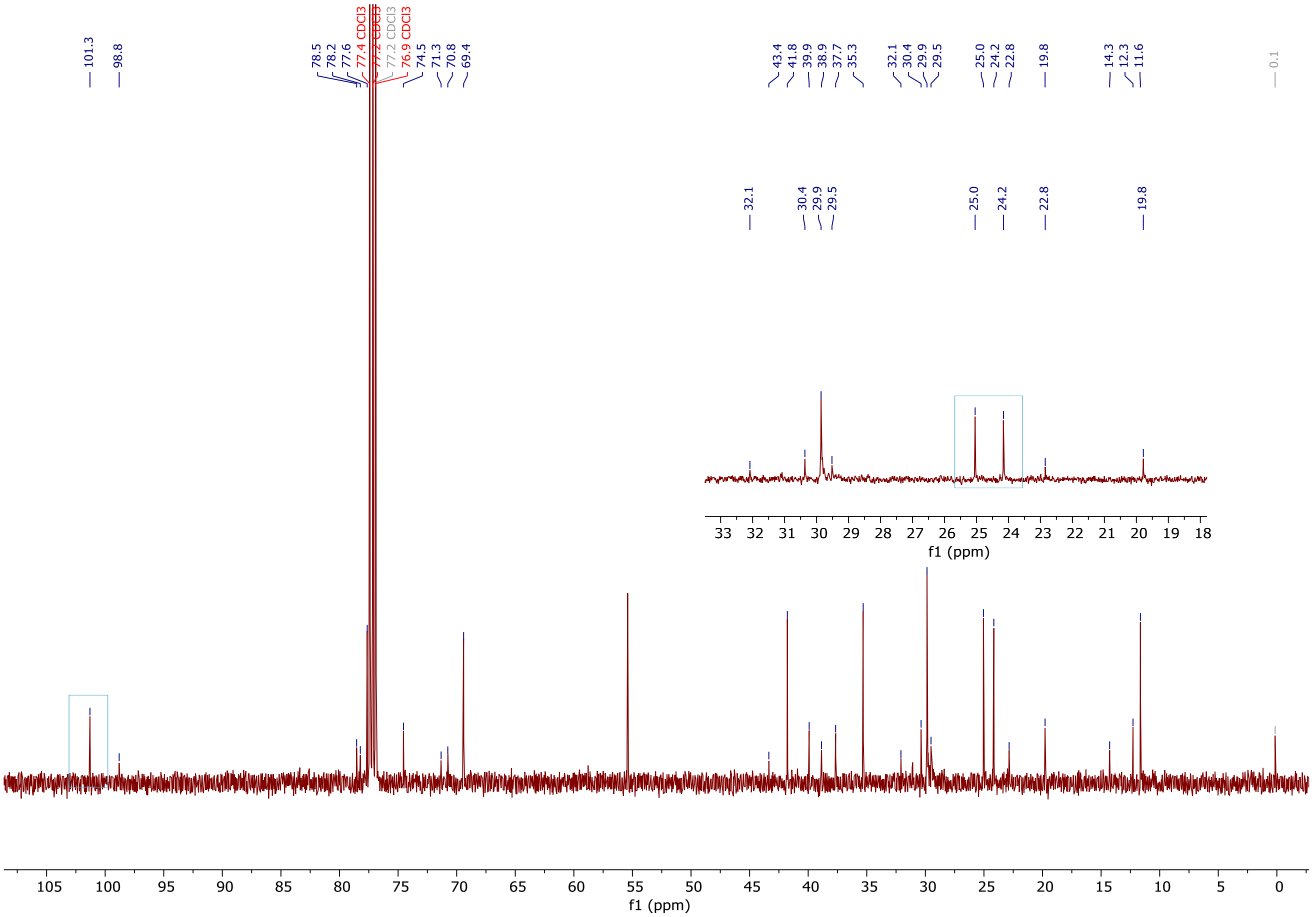

Catalyst synthesis

(*S*,*S*)-Imidodiphosphorimidate 7c

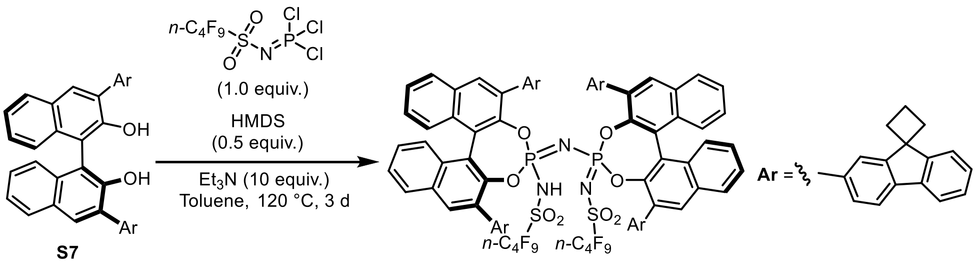

A flame dried Young tube was charged with the BINOL **S7** (270 mg, 0.39 mmol) and dry toluene (1.5 mL) was added under Ar. $P(NSO_2{}^nC_4F_9)Cl_3$ (169 mg, 0.39 mmol, 1 equiv.) was added dropwise via microsyringe at room

temperature followed by dry triethylamine (541 μL, 3.9 mmol, 10 equiv.). The heterogeneous reaction mixture was stirred for 30 min at r.t. and neat HMDS (39 μL, 0.19 mmol, 0.48 equiv.) was added dropwise. The reaction mixture was sealed and stirred at r.t. for additional 30 min, then heated to 120 °C for 3 d. It was cooled to r.t. and quenched with aqueous HCl (10%) and extracted three times with CH_2Cl_2. The combined organic layers were dried over Na_2SO_4, filtered and concentrated under reduced pressure. The crude residue was purified by column chromatography on silica gel (hexanes/MTBE 9/1 gradient to 3:1) to provide the desired product in the form of a salt. It was converted to a Brønsted acidic form via the following procedure: To a solution of a salt form of IDPi in CH_2Cl_2 (15 mL) was added aqueous 6M HCl (30 mL) and the biphasic mixture was vigorously stirred for 5-10 min. The layers were separated and the aqueous phase was extracted twice with CH_2Cl_2. The combined organic phases were evaporated under reduced pressure (*do not use a drying agent!*) and the obtained product was dried overnight under high vacuum at 37-40 °C to provide 217 mg (57%) product as an off-white amorphous solid.

^{1}H NMR (600 MHz, $CDCl_3$) δ 8.07 (s, 1H), 8.05 (dd, *J* = 8.5, 1.1 Hz, 1H), 8.02 (dd, *J* = 8.6, 1.2 Hz, 1H), 7.88 (dd, *J* = 8.1, 4.9 Hz, 2H), 7.80 (d, *J* = 1.6 Hz, 1H), 7.76 (d, *J* = 7.6 Hz, 1H), 7.74 – 7.70 (m, 1H), 7.64 (dd, *J* = 7.6, 1.0 Hz, 1H), 7.58 (ddd, *J* = 8.1, 5.4, 2.4 Hz, 1H), 7.55 – 7.52 (m, 2H), 7.40 (d, *J* = 7.9 Hz, 1H), 7.39 – 7.35 (m, 2H), 7.31 – 7.27 (m, 2H), 7.24 (td, *J* = 7.5, 1.2 Hz, 1H), 7.19 (td, *J* = 7.4, 1.1 Hz, 2H), 7.04 (s, 1H), 6.62 – 6.57 (m, 1H), 6.53 (d, *J* = 8.0 Hz, 1H), 6.33 (dfd, *J* = 7.9, 1.6 Hz, 1H), 2.81 – 2.72 (m, 1H), 2.69 – 2.53 (m, 4H), 2.39 – 2.19 (m, 6H), 2.16 – 2.07 (m, 1H).

^{13}C NMR (151 MHz, $CDCl_3$) δ 153.14, 152.92, 152.33, 152.13, 144.26 (t, *J* = 5.1 Hz), 143.07 (t, *J* = 5.0 Hz), 139.74, 139.12, 138.93, 138.62, 135.09, 134.99, 134.37, 133.90, 132.28, 132.16, 131.83, 131.72, 131.27, 129.48, 129.13, 128.90, 128.75, 128.09, 127.45, 127.30, 127.26, 127.09, 127.01, 126.82, 126.61, 126.59, 126.48, 123.76, 123.68, 123.64, 122.42, 121.83, 119.82, 119.27, 118.67, 118.32, 117.95* (t, *J* = 33.0 Hz), 116.03* (t, *J* = 33.3 Hz), 115.35* (t, *J* = 34.2 Hz), 113.36* (t, *J* = 34.1 Hz), 111.92 – 111.05* (m), 109.84* (t, *J* = 31.4 Hz), 108.05* (t, *J* = 34.0 Hz), 52.05, 52.04, 33.21, 33.05, 33.03, 32.68, 17.10, 17.00. * Detectable resonances for perfluorinated C-atoms.

^{19}F NMR (471 MHz, $CDCl_3$) δ -81.0 (t, *J* = 10.1 Hz, 3F), -111.0 – -112.5 (m, 2F), -121.1 (q, *J* = 10.9 Hz, 2F), -125.1 – -127.1 (m, 2F).

^{31}P NMR (203 MHz, $CDCl_3$) δ -16.9.

ESI-HRMS: calculated for $C_{112}H_{72}F_{18}N_3O_8P_2S_2^-$ ([M–H]$^-$): 2054.3954; found: 2054.3971.

$[\alpha]_D^{25}$ = +188.6 (*c* = 0.14, $CHCl_3$).

The enantiomer **(*R*,*R*)-7c** was obtained using BINOL ***ent*-S7** in a similar way: $[\alpha]_D^{25}$ = –176.1 (*c* = 0.16, $CHCl_3$).

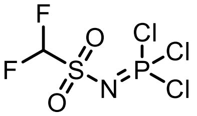

((Difluoromethyl)sulfonyl)phosphorimidoyl trichloride

A flame dried Schlenk tube was charged with the difluoromethanesulfonamide (1.27 g, 9.72 mmol) and phosphorous pentachloride (2.63 g, 12.63 mmol) under Ar. The Schlenck tube was connected to an oil bubbler and a two-neck flask, filled with solid NaOH in this order. The Ar line was closed and the reaction mixture was heated in an oil bath to 80 °C. At around 65 °C vigorous HCl bubbles start evolving and at 80 °C the initially solid mixture becomes a heterogeneous (turbid) liquid. The reaction mixture foams and vigorous HCl evolution is observed. The reaction mixture was heated at 80 °C for 3 h, after which crude ^{19}F and ^{31}P NMR of an aliquot indicated only the presence of the product and excess phosphorous pentachloride. The reaction mixture was further heated to 120 °C for 1 h to achieve ublimation of majority of the excess phosphorous pentachloride, and cooled to room temperature under Ar. Product was distilled into a separate flame-dried Schlenck tube under high vacuum (5 mbar, 120-150 °C) to give a colorless clear oil.

^{1}H NMR (501 MHz, $CDCl_3$) δ 6.2 (td, *J* = 53.8, 2.4 Hz, 1H).

^{13}C NMR (126 MHz, $CDCl_3$) δ 113.3 (td, *J* = 282.3, 7.7 Hz).

^{19}F{^{1}H} NMR (471 MHz, $CDCl_3$) δ -121.63 (d, *J* = 54.3 Hz, 2F).

^{31}P NMR (203 MHz, $CDCl_3$) δ 13.2.

ESI-HRMS: calculated for $CH_2Cl_3F_2NO_2PS^+$ ([M+H]$^+$): 265.8572; found: 265.8572.

(*S,S*)-Imidodiphosphorimidate 7d

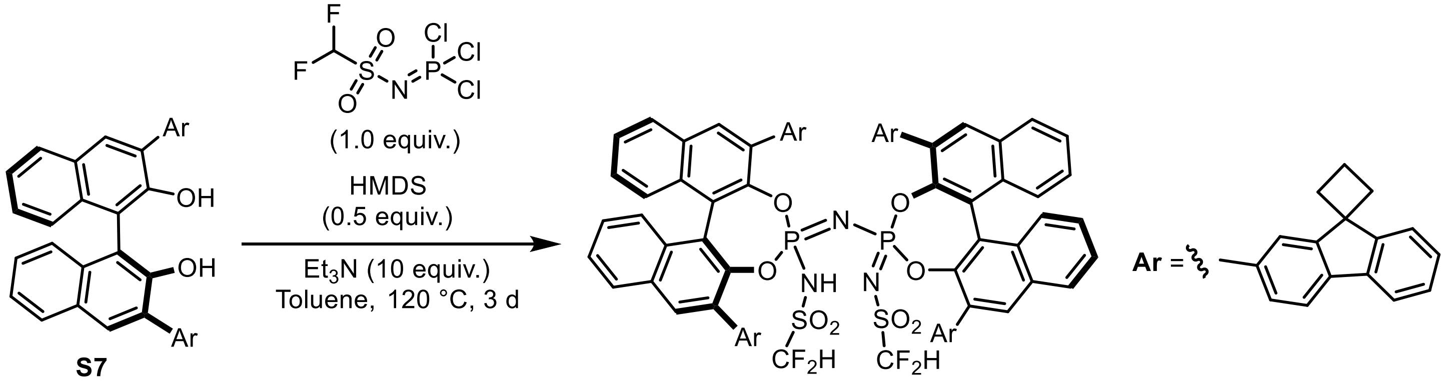

A flame dried Young tube was charged with the BINOL **S7** (180 mg, 0.26 mmol) and dry toluene (1 mL) was added under Ar. ((Difluoromethyl)sulfonyl)phosphorimidoyl trichloride (69 mg, 0.26 mmol, 1 equiv.) was added dropwise via microsyringe at room temperature followed by dry triethylamine (360 μL, 2.6 mmol, 10 equiv.). The heterogeneous reaction mixture was stirred for 30 min at r.t. and neat HMDS (26 μL, 0.12 mmol, 0.48 equiv.) was added dropwise. The reaction mixture was sealed and stirred at r.t. for additional 30 min, then heated to 120 °C for 3 d. It was cooled to r.t. and quenched with aqueous HCl (10%) and extracted three times with CH_2Cl_2. The combined organic layers were dried over Na_2SO_4, filtered and concentrated under reduced pressure. The crude residue was purified by column chromatography on silica gel (hexanes/AcOEt 10/1 gradient to 3:1) to provide the desired product in the form of a salt. It was converted to a Brønsted acidic form via the following procedure: To a solution of a salt form of IDPi in CH_2Cl_2 (10 mL) was added aqueous 6M HCl (20 mL) and the biphasic mixture was vigorously stirred for 5-10 min. The layers were separated and the aqueous phase was extracted twice with CH_2Cl_2. The combined organic phases were evaporated under reduced pressure (*do not use a drying agent!*) and the obtained product was dried overnight under high vacuum at 37-40 °C to provide 150 mg (70%) product as an off-white amorphous solid.

^{1}H NMR (600 MHz, $CDCl_3$) δ 8.13 – 8.03 (m, 3H), 7.87 – 7.79 (m, 4H), 7.67 (ddd, *J* = 8.4, 6.8, 1.3 Hz, 1H), 7.65 – 7.63 (m, 2H), 7.58 (ddd, *J* = 8.2, 6.7, 1.2 Hz, 1H), 7.51 – 7.45 (m, 2H), 7.39 (ddd, *J* = 8.6, 6.8, 1.3 Hz, 1H), 7.34 (ddd, *J* = 7.3, 1.3, 0.7 Hz, 1H), 7.29 (td, *J* = 7.5, 1.1 Hz, 1H), 7.26 – 7.19 (m, 4H), 7.17 (td, *J* = 7.5, 1.1 Hz, 1H), 6.56 (d, *J* = 7.9 Hz, 1H), 6.48 (d, *J* = 7.9 Hz, 1H), 6.09 (dd, *J* = 7.8, 1.6 Hz, 1H), 5.16 (t, *J* = 54.0 Hz, 1H), 2.82 – 2.71 (m, 1H), 2.68 – 2.50 (m, 4H), 2.41 – 2.32 (m, 4H), 2.27 – 2.15 (m, 3H).

^{13}C NMR (151 MHz, $CDCl_3$) δ 153.03, 152.90, 152.35, 151.87, 144.15 (t, *J* = 5.0 Hz), 143.31 (t, *J* = 5.0 Hz), 139.37, 138.96, 138.75, 138.59, 135.23, 134.99, 134.96, 134.46, 132.19, 132.07, 131.95, 131.85, 131.18, 129.18, 129.09, 128.88, 128.59, 128.05, 127.50, 127.46, 127.29, 127.08, 127.03, 126.92, 126.88, 126.77, 126.49, 124.18, 123.74, 123.62, 122.71, 122.50, 122.10, 119.80, 119.21, 118.76, 118.27, 112.72 (t, *J* = 282.4 Hz), 52.09, 52.04, 33.67, 33.10, 32.90, 32.84, 17.10, 17.05.

^{19}F{^{1}H} NMR (565 MHz, $CDCl_3$) δ -120.9 (dd, *J* = 266.7, 54.0 Hz, 2F), -121.5 (dd, *J* = 266.2, 54.1 Hz, 2F).

^{31}P NMR (243 MHz, $CDCl_3$) δ -15.3.

ESI-HRMS: calculated for $C_{106}H_{74}F_4N_3O_8P_2S_2^-$ ([M–H]$^-$): 1718.4334; found: 1718.4342.

$[\alpha]_D^{25}$ = +234.3 (*c* = 0.14, $CHCl_3$).

The enantiomer **(*R,R*)-7d** was obtained using BINOL ***ent*-S7** in a similar way: $[\alpha]_D^{25}$ = –242.8 (*c* = 0.15, $CHCl_3$).

Synthesis of enolsilanes

***General synthesis of E-enolsilanes*:**

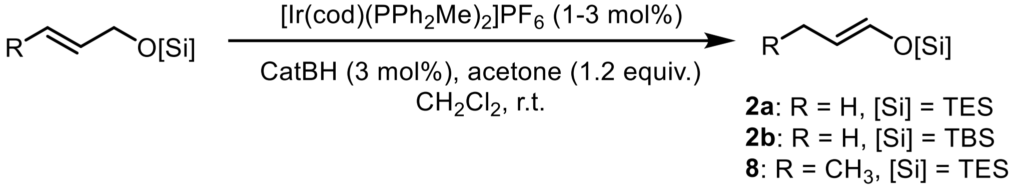

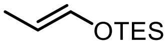

(*E*)-Triethyl(prop-1-en-1-yloxy)silane (2a)

A solution of allyloxytriethylsilane (7.6 g, 44.1 mmol) and dry acetone (660 µL) in dry dichloromethane (44 mL) was added to a stirring solution of (1,5-cyclooctadiene)bis(methyldiphenylphosphine)iridium(I) hexafluorophosphate (373 mg, 0.441 mmol) and acetone (660 µL) in dry dichloromethane (26 mL) under Ar. A solution of catecholborane in tetrahydrofuran (441 µL, 1 M, 0.441 mmol) was immediately added to the stirring reaction mixture. The resulting red solution was stirred for 15 min at 23 °C, then ethylenediamine (177 µL, 2.6 mmol) was added. The yellow solution was concentrated by rotary evaporation (50-150 mbar, 40 °C). The residue was purified by flash-column chromatography (SiO_2, pentane) to provide 6.6 g (87%) (*E*)-enolate as a colorless oil and 543 mg (7%) (*Z*)-enolate. *The products were dried on a rotary evaporator for 40-60 min at 150 mbar at 30 °C.* ^{1}H NMR (501 MHz, $CDCl_3$) δ 6.23 (dq, J = 11.9, 1.7 Hz, 1H), 4.99 (dq, J = 11.9, 6.8 Hz, 1H), 1.51 (dd, J = 6.8, 1.7 Hz, 3H), 0.97 (t, J = 8.0 Hz, 9H), 0.65 (q, J = 7.9 Hz, 6H). ^{13}C NMR (126 MHz, $CDCl_3$) δ 140.3, 105.7, 12.2, 6.5, 4.4.

EI-HRMS: calculated for $C_9H_{20}OSi^{\bullet+}$ ([M]$^{\bullet+}$): 172.1278; found: 172.1277.

R$_f$ = 0.39 (silica, hexanes),

ρ = 0.83 g/mL

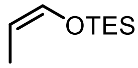

4a

(*Z*)-Triethyl(prop-1-en-1-yloxy)silane (4a)

^{1}H NMR (501 MHz, $CDCl_3$) δ 6.21 (dq, J = 5.6, 1.8 Hz, 1H), 4.50 (p, J = 6.7 Hz, 1H), 1.58 (dd, J = 6.9, 1.5 Hz, 3H), 0.98 (t, J = 8.0 Hz, 9H), 0.65 (q, J = 7.9 Hz, 6H).

^{13}C NMR (126 MHz, $CDCl_3$) δ 139.0, 104.9, 8.9, 6.5, 4.5.

R$_f$ = 0.57 (silica, hexanes),

ρ = 0.82 g/mL.

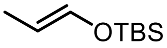

2b

(*E*)-*Tert*-butyldimethyl(prop-1-en-1-yloxy)silane (2b)

A solution of (allyloxy)(*tert*-butyl)dimethylsilane (1.034 g, 6.0 mmol) and acetone (287 µL, 3.9 mmol, 0.6 equiv.) in dry dichloromethane (23 mL) was added to a stirring solution of (1,5-cyclooctadiene)bis(methyldiphenylphosphine)iridium(I) hexafluorophosphate (152 mg, 0.18 mmol, 3 mol%) and acetone (287 µL, 3.9 mmol, 0.6 equiv.) in dry dichloromethane (13 mL). A solution of catecholborane in tetrahydrofuran (180 µL, 1 M, 0.18 mmol, 3 mol%) was immediately added to the stirring reaction mixture. The resulting red solution was stirred for 10 min at 23 °C, then ethylenediamine was added. The yellow product mixture was concentrated by rotary evaporation (150 mbar, 30 C). The residue was purified by flash-column chromatography (SiO_2, *n*-pentane) to provide 650 mg (63%) (*E*)-enolate as a colorless oil (*E*/Z>99:1, contaminated with ca 2.5 mol% toluene) and 267 mg (26%) mixture of isomers (*E/Z* 96:4, contaminated with ca 30 mol% toluene). Overall yield 89%. *Because of volatility, both fractions were dried for 40 min on a rotary evaporator at 30 °C at 150 mbar.*

^{1}H NMR (501 MHz, $CDCl_3$) δ 6.22 (dq, *J* = 11.9, 1.6 Hz, 1H), 4.98 (dq, *J* = 11.9, 6.8 Hz, 1H), 1.51 (dd, *J* = 6.8, 1.7 Hz, 3H), 0.91 (s, 9H), 0.12 (s, 6H).
^{13}C NMR (126 MHz, $CDCl_3$) δ 140.7, 105.9, 25.9, 18.5, 12.3, -5.1.
R_f(*E*-isomer) = 0.39 (*n*-pentane). R_f(*Z*-isomer) = 0.54 (*n*-pentane) *-forms in traces.*

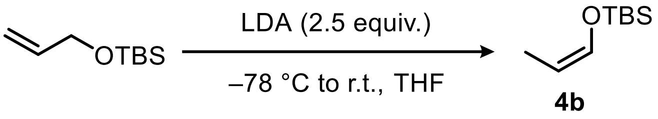

(*Z*)-*Tert*-butyldimethyl(prop-1-en-1-yloxy)silane (4b)
Slight modification of a literature procedure:[10] Under an argon atmosphere, a pentane solution of *n*-BuLi (14 mL, 34.8 mmol, 2.5 M in hexanes, 2.5 equiv.) was added dropwise to a solution of diisopropylamine (4.9 mL, 34.8 mmol, 2.5 equiv.) in dry THF (4 mL) at -78 °C. After stirring for 5 min, the solution was allowed to warm to 0 °C for 2 min and was then re-cooled to -78 °C. Allyloxy-tert-butyldimethylsilane (3 mL, 14.1 mmol) was added dropwise to the mixture at -78 °C and the mixture was allowed to stir for 5 min at -78 °C before warming up to room temperature. After 3 h, the reaction mixture was quenched by saturated ammonium chloride solution. The mixture was then extracted with diethyl ether three times and the organic phase was washed with water, brine and dried over Na_2SO_4. After filtration the filtrate was concentrated on a rotary evaporator at at 30 °C at 150 mbar. The residue was purified by column chromatography by flash-column chromatography (SiO_2, *n*-pentane) to provide 2.06 g (85%) (*Z*)-enolate after drying for *2 h on a rotary evaporator at 30 °C at 200 mbar*.
^{1}H NMR (501 MHz, $CDCl_3$) δ 6.19 (dq, *J* = 5.6, 1.8 Hz, 1H), 4.50 (qd, *J* = 6.7, 5.8 Hz, 1H), 1.57 (dd, *J* = 6.7, 1.8 Hz, 3H), 0.93 (s, 9H), 0.13 (s, 6H).
^{13}C NMR (126 MHz, $CDCl_3$) δ 139.4, 105.0, 25.8, 18.5, 9.1, -5.2.
R_f = 0.54 (*n*-pentane).

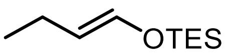

(*E*)-(But-1-en-1-yloxy)triethylsilane (8)
Using the same procedure for **2a** and **2b**: (*E*)-(but-2-en-1-yloxy)triethylsilane (1 g, 5.4 mmol, 94:6 E/Z mixture) was isomerized using (1,5- cyclooctadiene)bis(methyldiphenylphosphine)iridium(I) hexafluorophosphate (91 mg, 0.11 mmol, 2 mol%) to provide 587 mg (59%) pure (*E*)-enolsilane enolsilane **6** and 175 mg (17%) mixture of isomers (E/Z 90:10).
^{1}H NMR (501 MHz, $CDCl_3$) δ 6.24 (dt, *J* = 11.9, 1.4 Hz, 1H), 5.03 (dt, *J* = 11.9, 7.2 Hz, 1H), 1.94 – 1.86 (m, 2H), 0.98 (t, *J* = 7.9 Hz, 9H), 0.95 (t, *J* = 7.4 Hz, 4H), 0.66 (q, *J* = 8.0 Hz, 6H).
^{13}C NMR (126 MHz, $CDCl_3$) δ 139.5, 113.6, 20.9, 15.3, 6.7, 4.6.
R_f = 0.35 (silica, hexanes).
ρ = ca 0.94 g/mL.

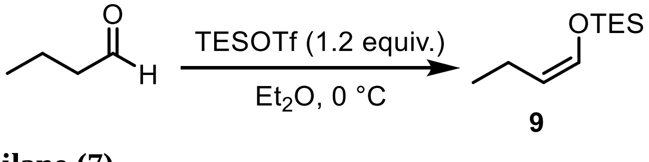

(*Z*)-(But-1-en-1-yloxy)triethylsilane (7)
To a solution of triethylsilyl trifluoromethanesulfonate (1.81 mL, 8.0 mmol, 1.2 equiv.) in dry Et_2O (12 mL) was added Et_3N (1.1 mL, 8.0 mmol, 1.2 equiv.) at 0 °C under Ar in a flame dried Schlenk flask. After stirring for 30 min, a solution of distilled *n*-butyraldehyde (0.6 mL, 6.7 mmol) in Et_2O (4mL) was added to the mixture and stirred for 2 h at 0 °C. The reaction mixture was then diluted with excess *n*-pentane and filtered through a short silica pad. The silica pad was washed with *n*-pentane and the reaction mixture was concentrated on a rotary evaporator (40 °C water bath, pressure 300 mbar). The residue was purified by column chromatography (silicagel, *n*-pentane) to provide 655 mg pure (*Z*)-enolsilane **7** (53%) and 65 mg pure (*E*)-enolsilane **6** (5.2%) as colorless oils. Overall yield 720 mg (58*%). The fractions of the pure products were dried on a rotary evaporator at 30 °C, 200 mbar for 1 h.*

^{1}H NMR (501 MHz, $CDCl_3$) δ 6.16 (dt, *J* = 5.8, 1.5 Hz, 1H), 4.44 (td, *J* = 7.1, 5.8 Hz, 1H), 2.14 – 2.06 (m, 2H), 1.03-0.92 (m, 12H), 0.66 (q, *J* = 7.9 Hz, 6H).
^{13}C NMR (126 MHz, $CDCl_3$) δ 137.9, 113.0, 17.2, 14.5, 6.7, 4.6.
APCI-HRMS: calculated for $C_{10}H_{23}OSi^+$ ($[M+H]^+$): 187.1513; found: 187.1513.
R_f = 0.50 (hexanes).
ρ = ca 0.87 g/mL

Reaction investigations

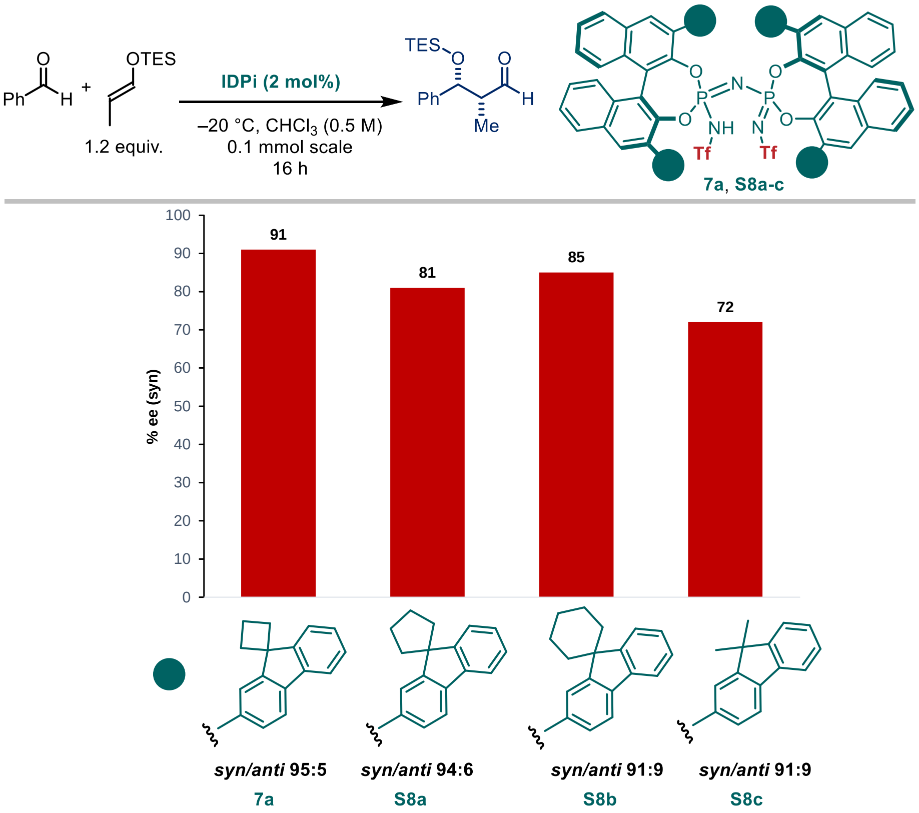

Figure S1. Identification of the spirocyclobutane-2-fluorenyl-goup as the most privileged substituent.

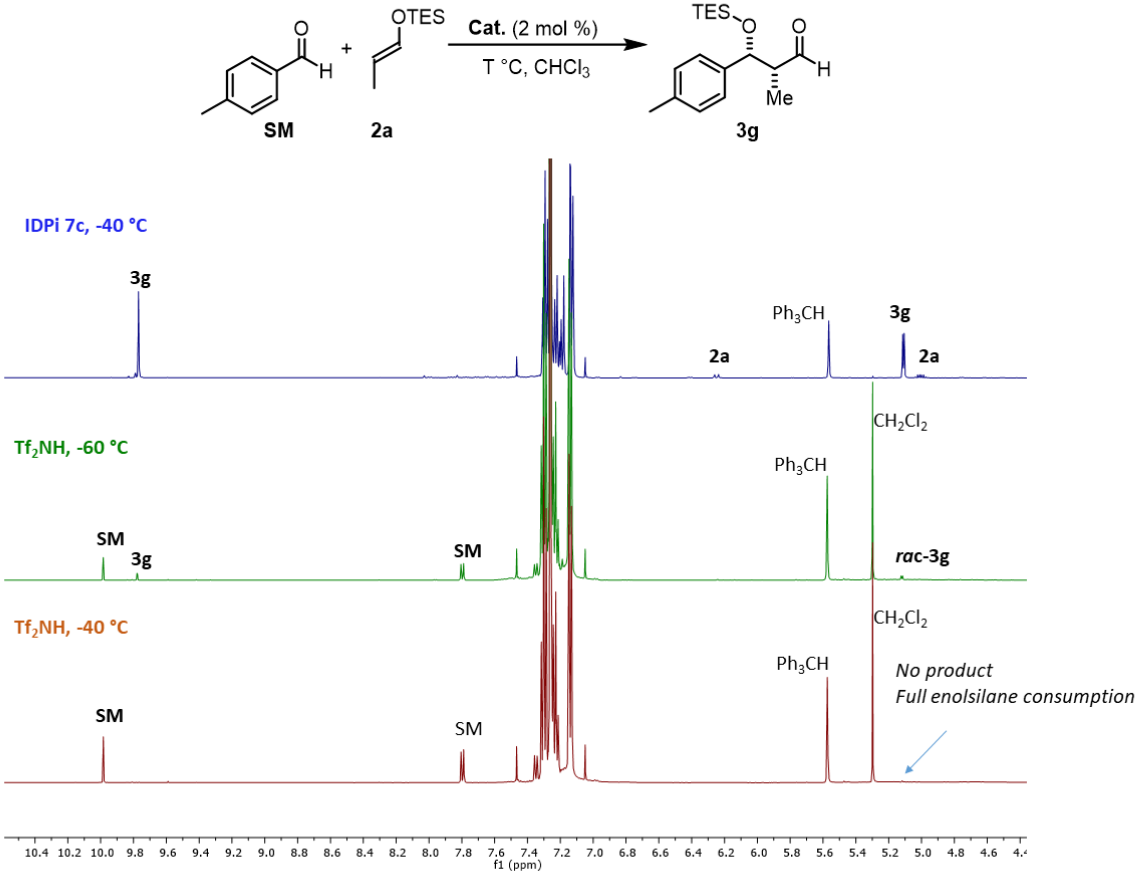

Figure S2. Comparison with Tf_2NH, which showed traces of desired product formation only at –60 °C.

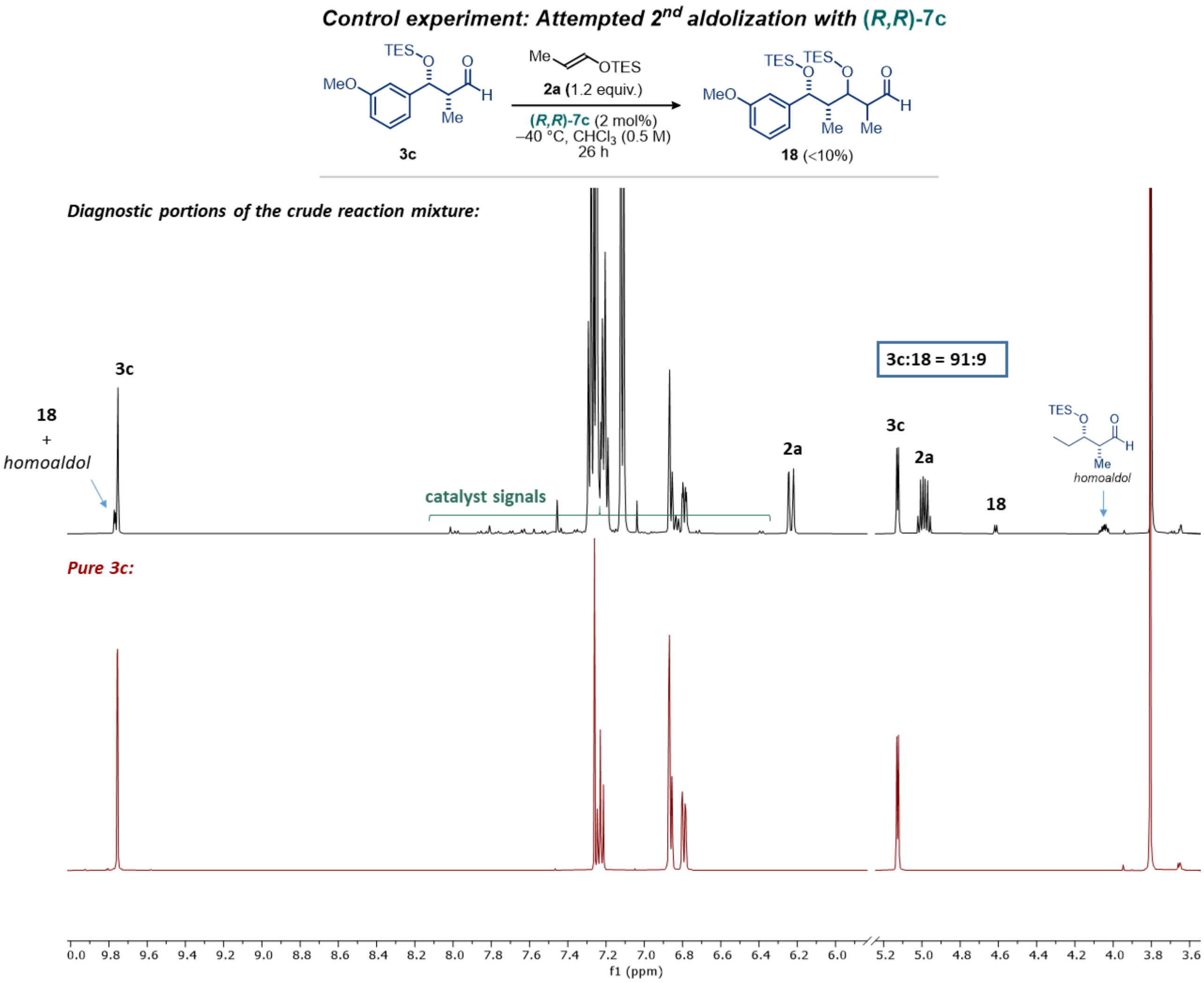

Figure S3. Excerpt of a crude ^{1}H NMR of the attempted 2nd aldolization experiment using ***(R,R)*-7c** and its overlay with the ^{1}H NMR spectrum of pure starting material **3c**, which was obtained using ***(S,S)*-7c**.

Analysis of the crude reaction mixture by mass-spectroscopy (ESI+) confirms the formation of dialdolate **18** in a small amout:

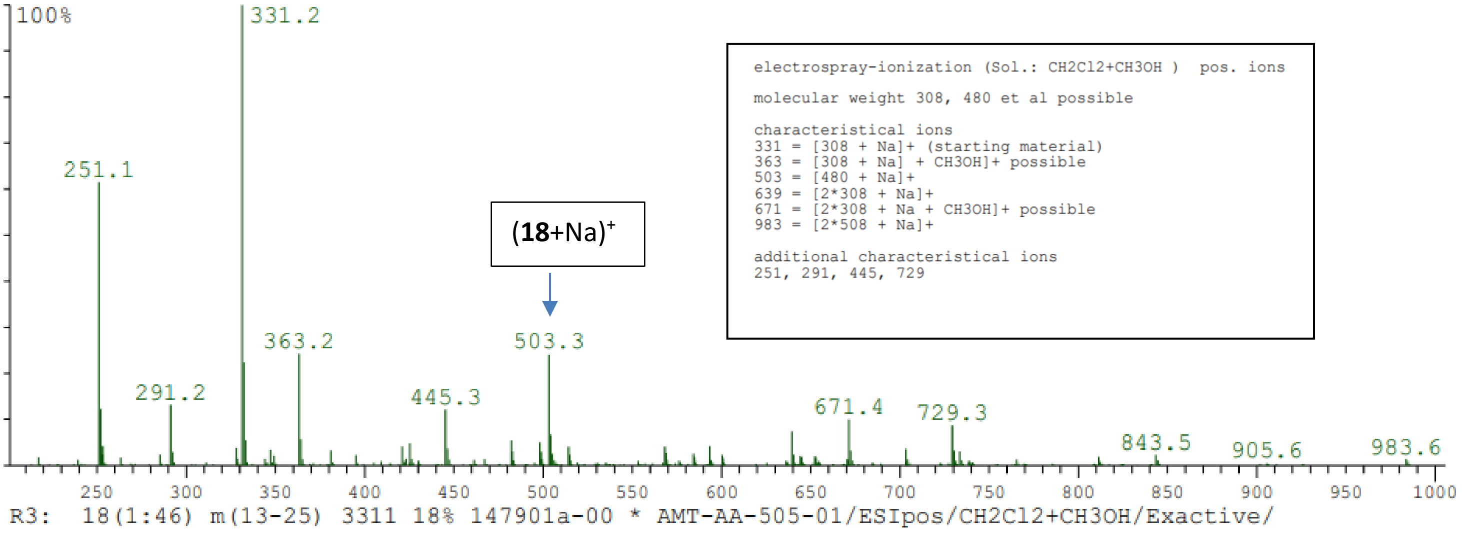

Mechanistic investigations

General procedure for the sample preparation

Stock solution A (166.67 mM benzaldehyde in dry $CDCl_3$): In a flame-dried Young tube under Ar dissolve of benzaldehyde (102 µL, 1.00 mmol) in 6 mL $CDCl_3$. **Stock solution B** (9.3 mM IDPi **7d** in dry $CDCl_3$): Dissolve 8 mg IDPi **7d** (4.65 µmol) in dry $CDCl_3$ (0.5 mL) in a dry Schlenk flask.

A flame-dried NMR tube was charged with 215 µL stock solution B and evaporated to dryness under high vacuum to achieve 2 mol% loading. The NMR tube was refilled with Ar and stock solution A (0.6 mL, 0.1 mmol) was added. The NMR tube was cooled to -78 °C with dry ice and neat enolsilane **4b** (45 µL, 2 equiv. was added, the NMR tube was quickly turned upside down and vortexed. After quick transfer to the precooled NMR probe (–60 °C) of a 500 MHz Bruker spectrometer, ^{1}H NMR spectra were acquired every 3 min after quick shimming of the sample until full conversion was observed. The NMR data was generally imported with the Reaction Monitoring Plugin of MNOVA 14.1.2 and processed there. All the data was referenced to the initial concentration of benzaldehyde (c=166.67 mM) at t=0.

Reaction Progress Analysis

Figure S4 shows the reaction progress for the *anti*-selective Mukaiyama aldol addition of benzaldehyde **1** with enolsilane **4b** under standard conditions following the general procedure:

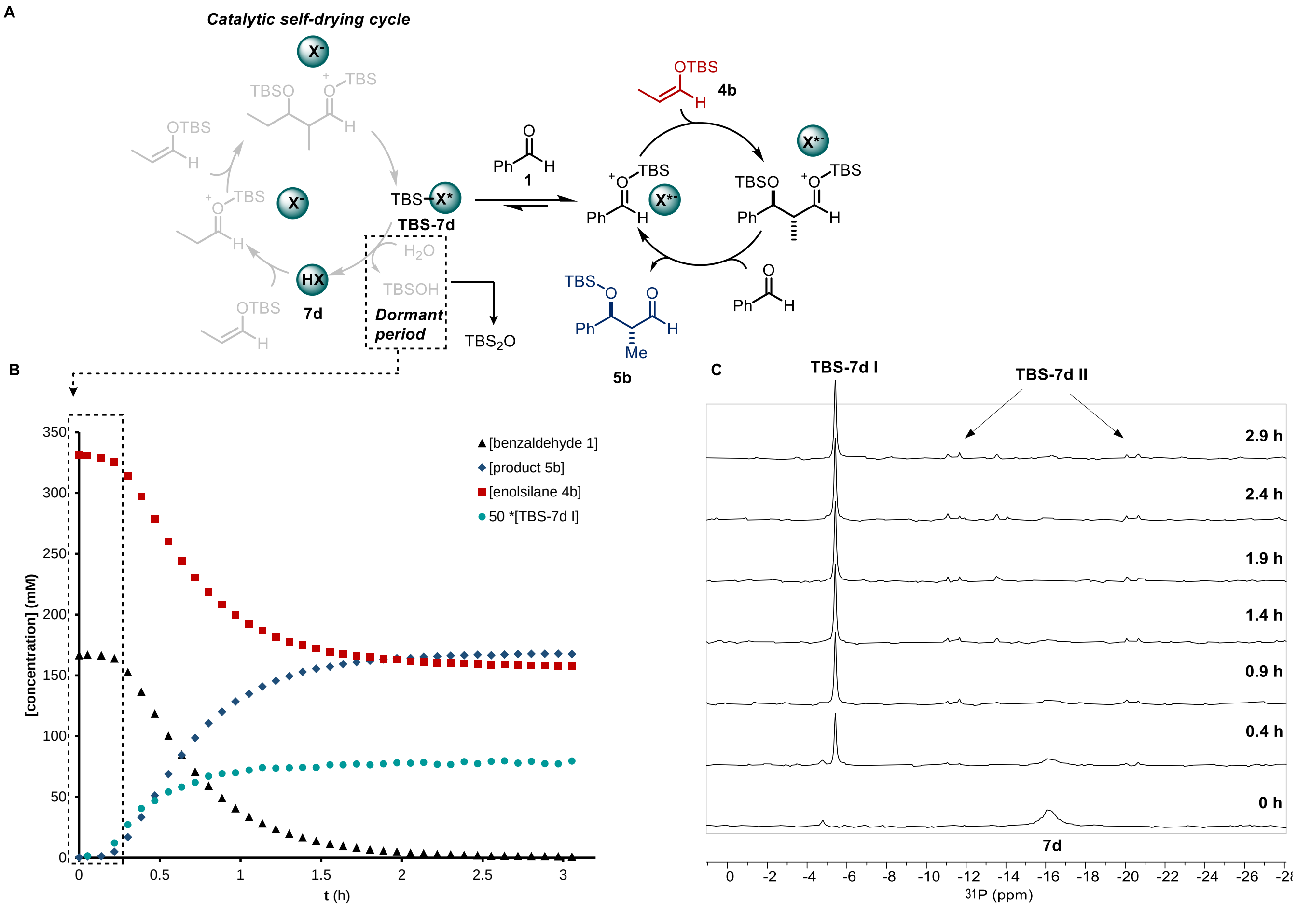

Figure S4. A: proposed reaction mechanism; B: Concentration profile obtained from NMR reaction monitoring of the *anti*-aldolization of benzaldehyde **1** with catalyst **7d** (2 mol%) in the presence of enolsilane **4b** (2 equiv.) at –60 °C in $CDCl_3$; C: ^{31}P NMR spectra taken at different time points during the reaction.

After a dormant period of approximately 15 min, where H_2O in the reaction mixture is consumed to form TBSOH, the product formation starts. During the dormant period silylated IDPi species are not detected by ^{31}P NMR. After the reaction has started two different silylated catalyst species **TBS-7d** are observed as well as residual **7d**. When the reaction is completed (after ~2.5 h), only silylated catalyst species can be observed in the ^{31}P NMR.

Determination of the reaction order of IDPI catalyst 7d for the anti-selective Mukaiyama aldol addition

The catalyst order of IDPi **7d** was investigated by Variable Time Normalization Analysis (VTNA).[11,12,13,14] Samples were prepared according to the procedure above using the required amounts of stock solution B. The time point of the reaction after the initial dormant period was used as t=0 for all the reactions. The best overlap of the reactions profiles (Figure ***S***S5) was obtained when a first order dependence in catalyst concentration is assumed, in agreement with the kinetics of acetaldehyde enolsilane additions.[3] The reaction was conducted with 0.5 mol%, 1.0 mol% and 2 mol% of catalyst **7d**.

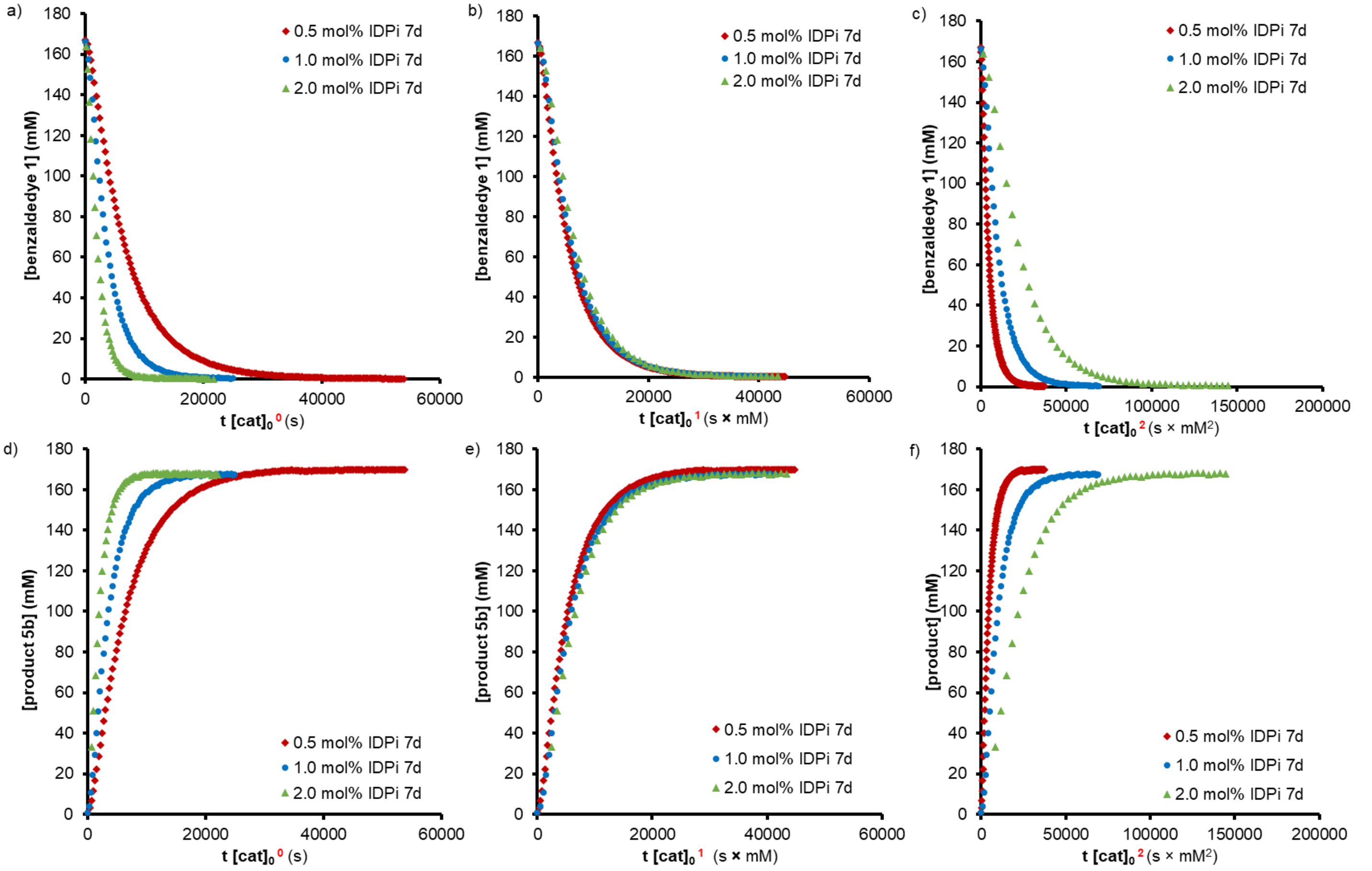

Figure S5: *Reaction progress profiles for the reaction of benzaldehyde* ***1*** *(a-c) with 2 equiv. enolsilane* ***4b*** *and IDPi* ***7d*** *and the formation of Mukaiyama aldol product* ***5b*** *(d-f) in $CDCl_3$ at –60 °C. The time scales were normalized to different catalyst orders: zeroth order (a;d), first order (b;e) and 2nd order (c;f).*

Silylation of IDPi 7d

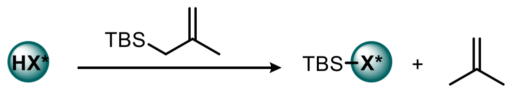

7d **TBS-7d**

A sample of IDPi **7d** (6.74 mg, 3.92 µmol) was mixed with *tert*-butyldimethyl(2-methylallyl)silane (7 mg, 0.04 mmol, 10 equiv.) in 0.5 mL $CDCl_3$ at r.t. and characterized by NMR at Bruker AVNeo 600 MHz NMR spectrometer 298K equipped with a BBO cryoprobe. VT NMR data was acquired at a AVIII 500 MHz NMR spectrometer.

^{1}H NMR spectrum of the activated IDPi mixture in CDCl$_3$ (600 MHz, 298K):

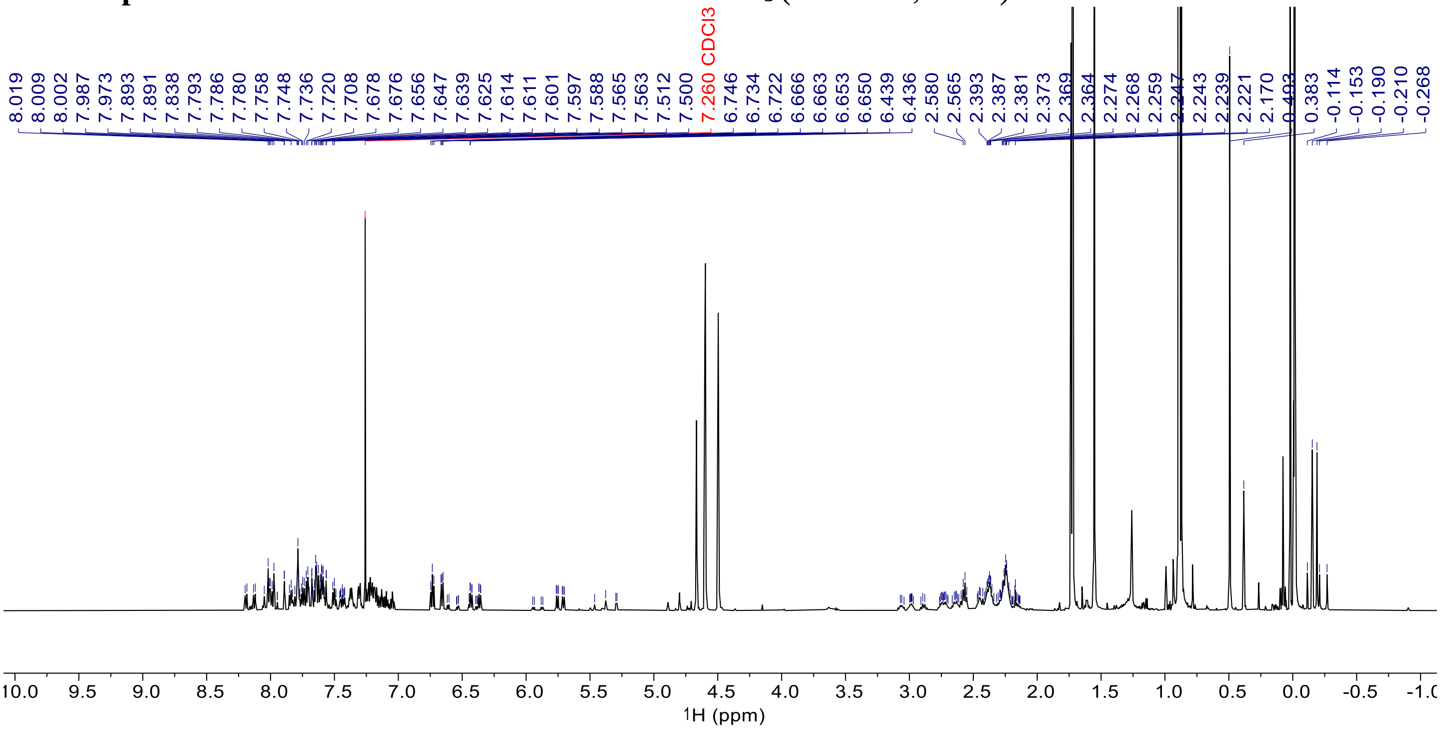

^{31}P NMR spectrum of the silylated IDPi 7d mixture in CDCl$_3$ (243 MHz, 298 K):

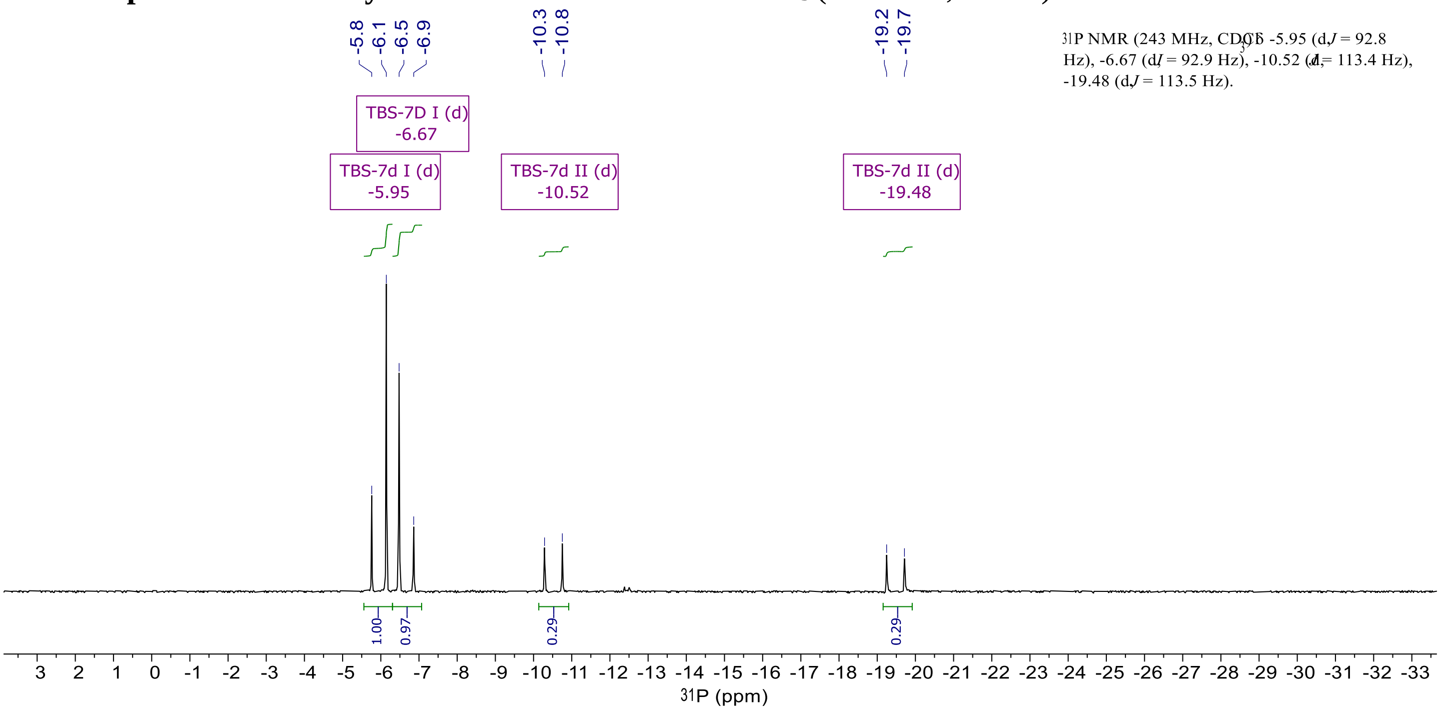

In agreement to the data from the reaction monitoring, two silylated species are observed by ^{31}P NMR at 25°C. The major species shows two ^{31}P doublets with similar chemical shifts of -6.7 ppm and -6.0 ppm and a $^2J_{31P\text{-}31P}$=92.8 Hz. The minor component shows signals at -10.5 ppm and -19.5 ppm with a significantly different $^2J_{31P\text{-}31P}$ coupling (113.4 Hz).

^{31}P NMR spectra of the silylated IDPi mixture 7d in $CDCl_3$ at different temperatures (202.3 MHz, 298-213 K):

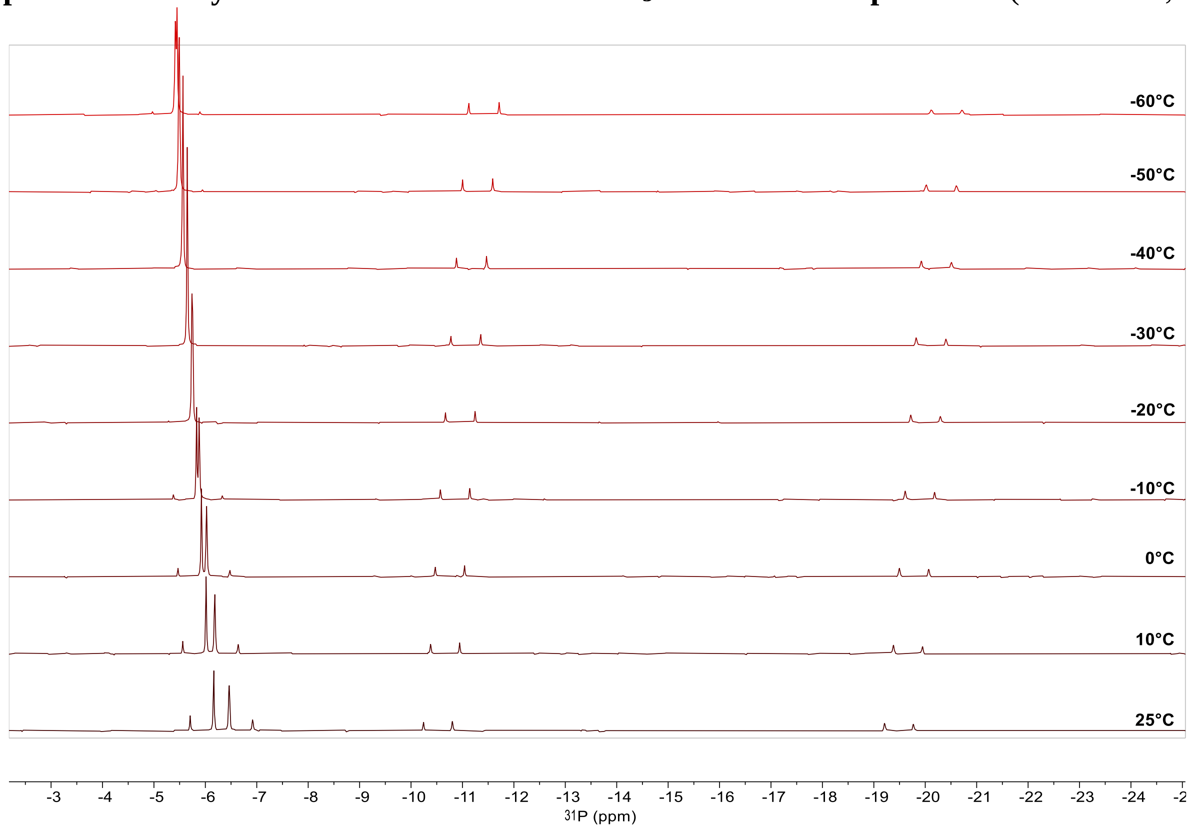

The variable temperature data shows a temperature dependence of the ^{31}P NMR shift. At lower temperatures, a deshielding of the ^{31}P signals of the major species is observed, whereas the minor species are more shielded at towards lower temperatures. At temperatures < -20 °C the ^{31}P NMR signals of the major species forms a strongly coupled AB spin system, so that only the major central line is observed. This matches the spectra acquired during the reaction monitoring.

^{1}H-^{31}P HMBC spectrum of the silylated IDPi 7d mixture in $CDCl_3$:

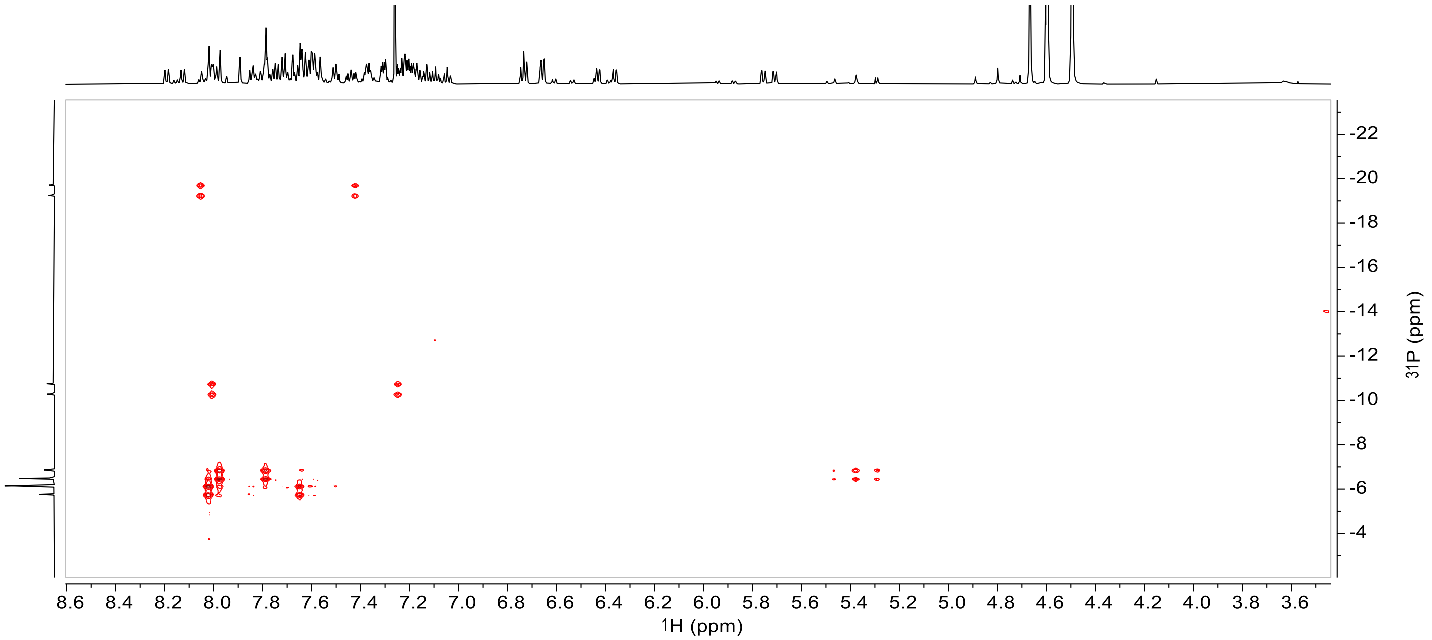

The ^{1}H-^{31}P HMBC spectrum shows long-range correlations of the IDPi core to the BINOL backbone. The major species shows a cross peak of the CF_2H-group at 5.4 ppm to the ^{31}P signal at -6.7 ppm.

^{29}Si INEPT NMR spectrum of the silylated IDPi 7d mixture in $CDCl_3$ (119.24 MHz, 298K):

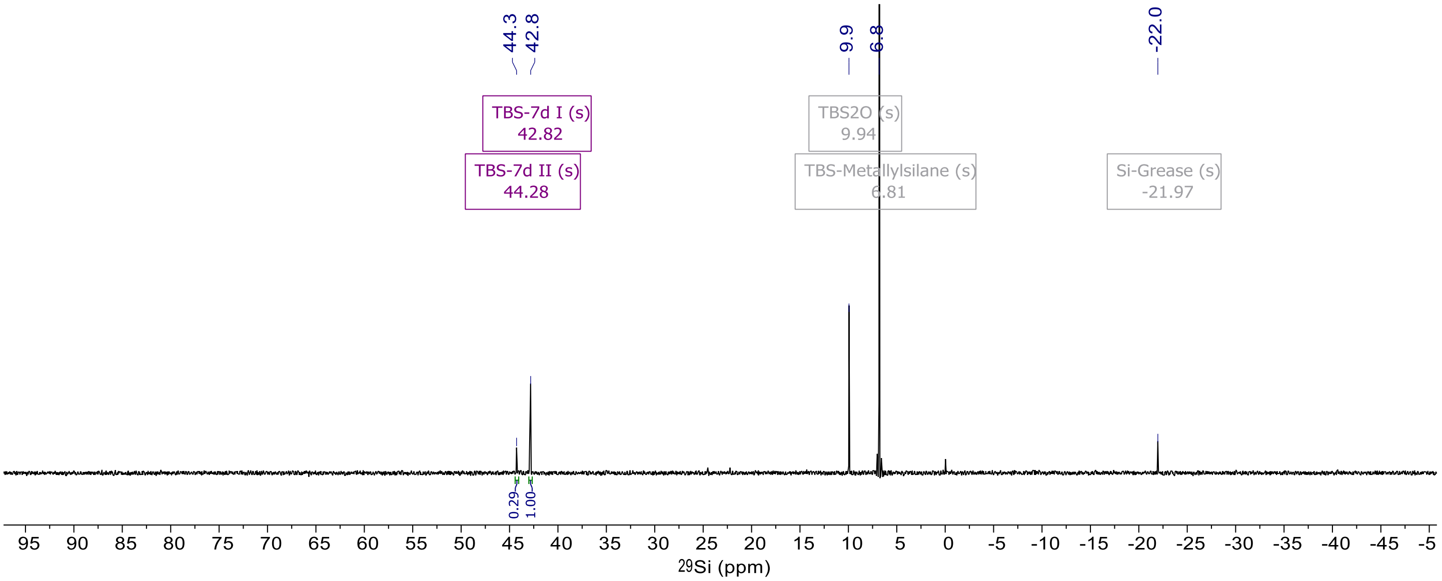

The ^{29}Si NMR data shows two singlets of the silylated IDI species at 42.8 ppm (major) and 44.3 ppm (minor). The relative integrals match the data obtained from ^{31}P NMR.

^{1}H-^{29}Si HMBC of the silylated IDPi 7d mixture in $CDCl_3$:

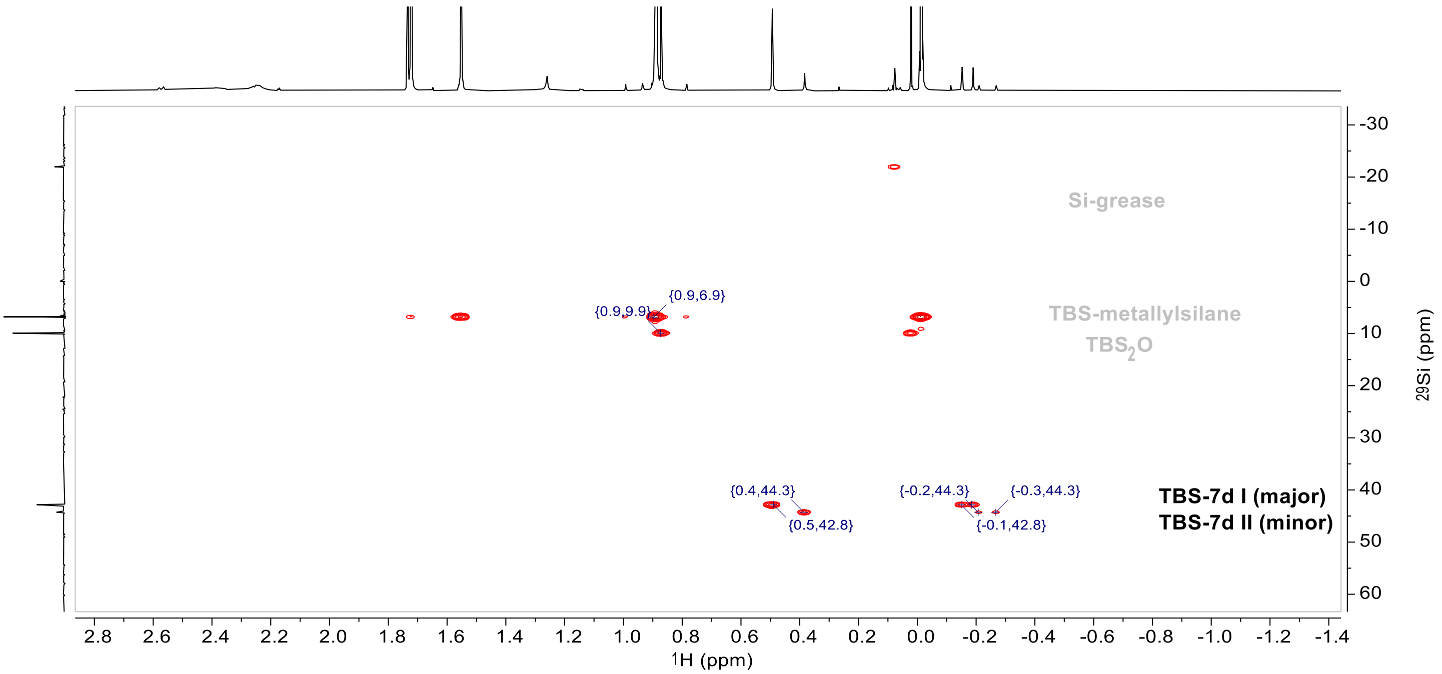

^{19}F NMR spectrum of the silylated IDPi 7d mixture in $CDCl_3$ (564.72 MHz, 298K):

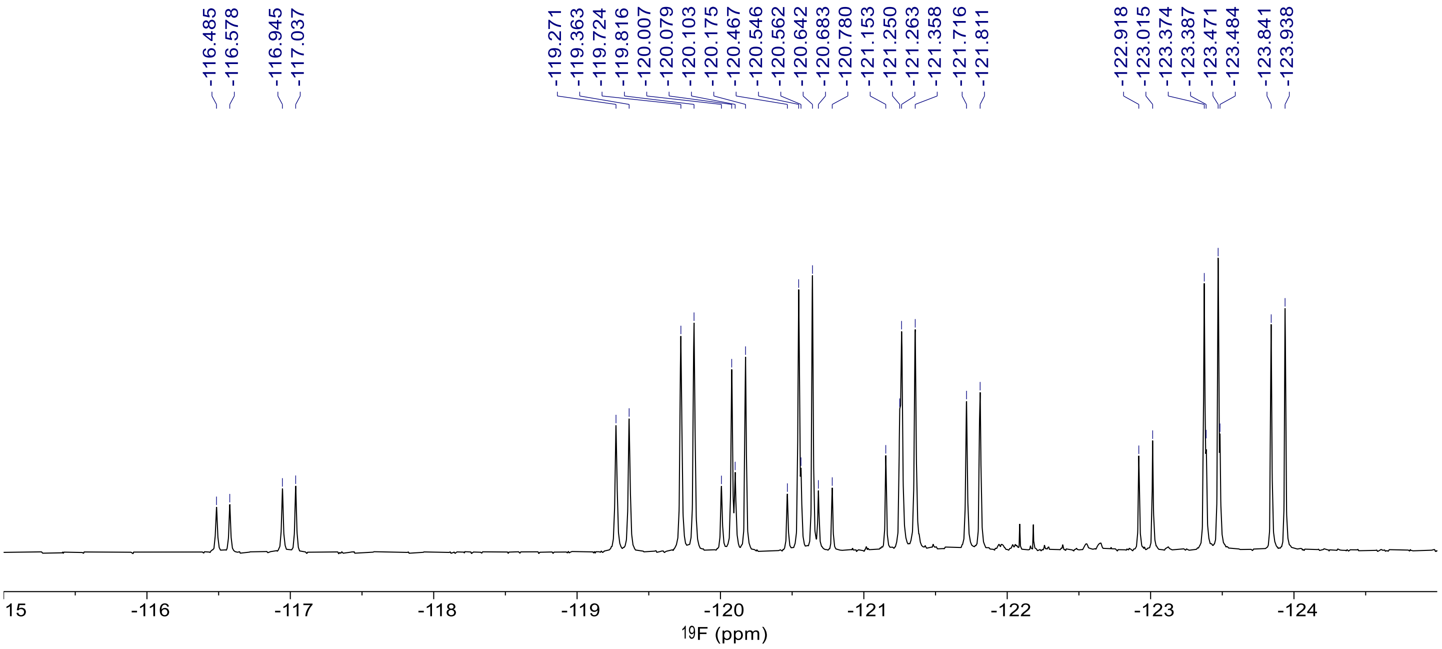

^{19}F{^{1}H} NMR spectrum of the silylated IDPi 7d mixture in $CDCl_3$ (470.31 MHz, 298K):

major species:
^{19}F{^{1}H} NMR (470 MHz, $CDCl_3$) δ -119.54 (d, J = 255.3 Hz), -120.35 (d, J = 263.7 Hz), -121.55 (d, J = 255.5 Hz), -123.67 (d, J = 263.7 Hz).

minor species:
^{19}F{^{1}H} NMR (470 MHz, $CDCl_3$) δ -116.76 (d, J = 259.7 Hz), -120.29 (d, J = 259.7 Hz), -120.96 (d, J = 265.4 Hz), -123.22 (d, J = 265.4 Hz).

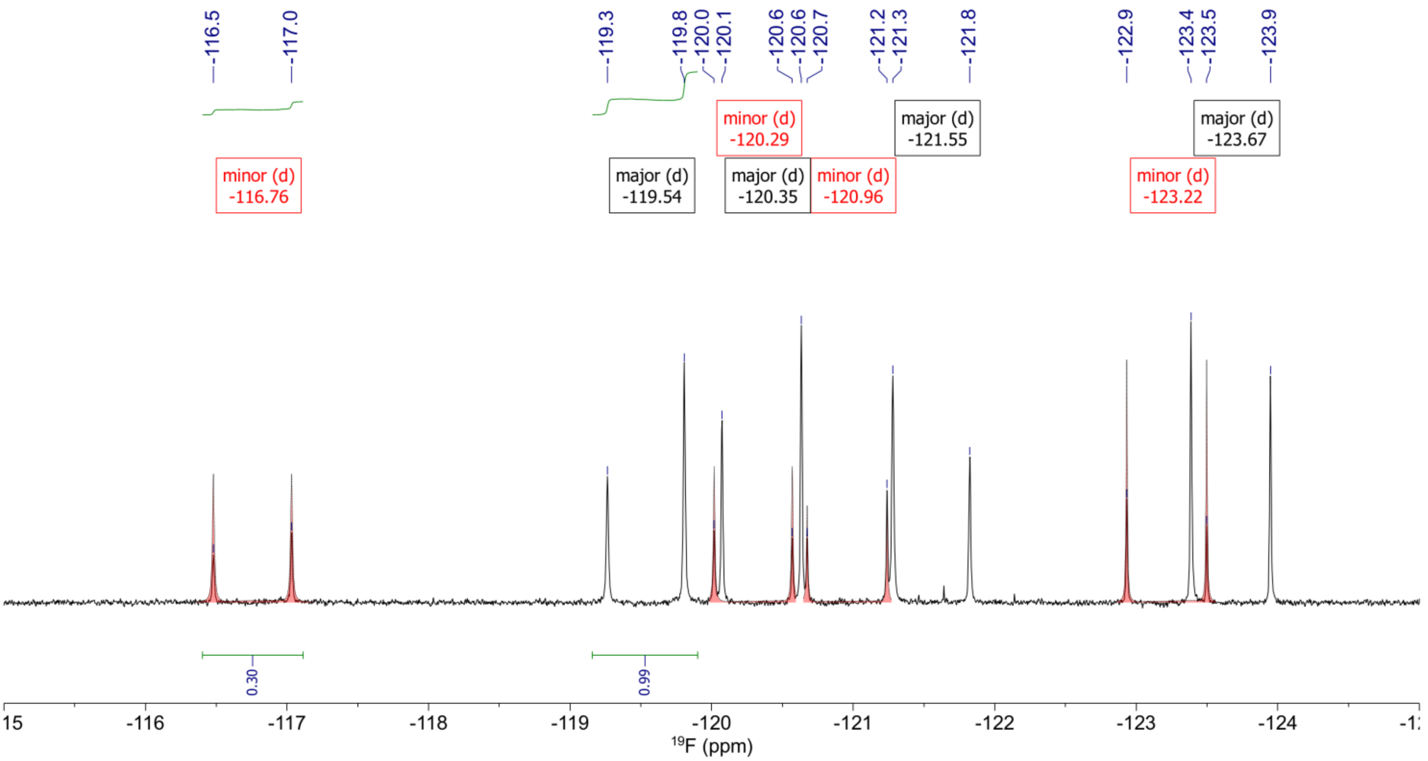

Excerpts of the ^1H-^1H ROESY NMR of the silylated IDPi 7d mixture in $CDCl_3$ (600 MHz, 200ms spinlock 298K)

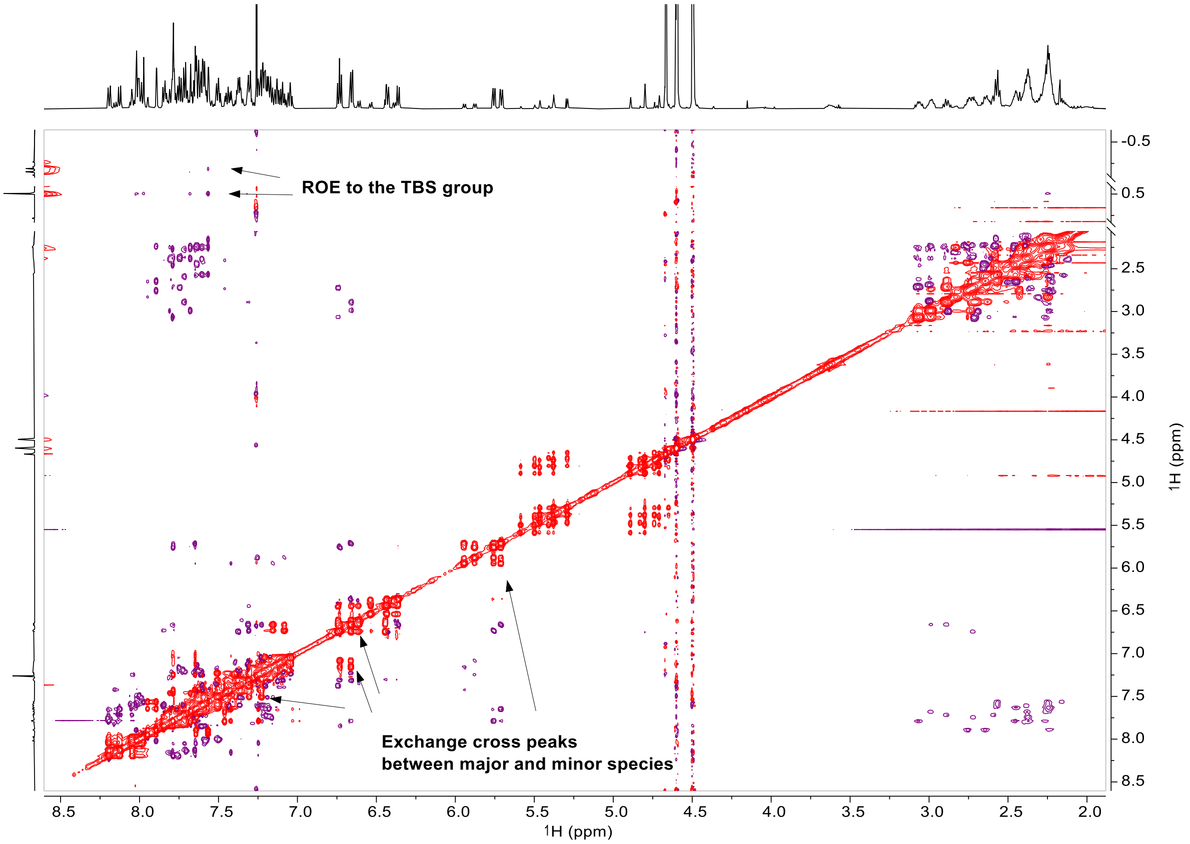

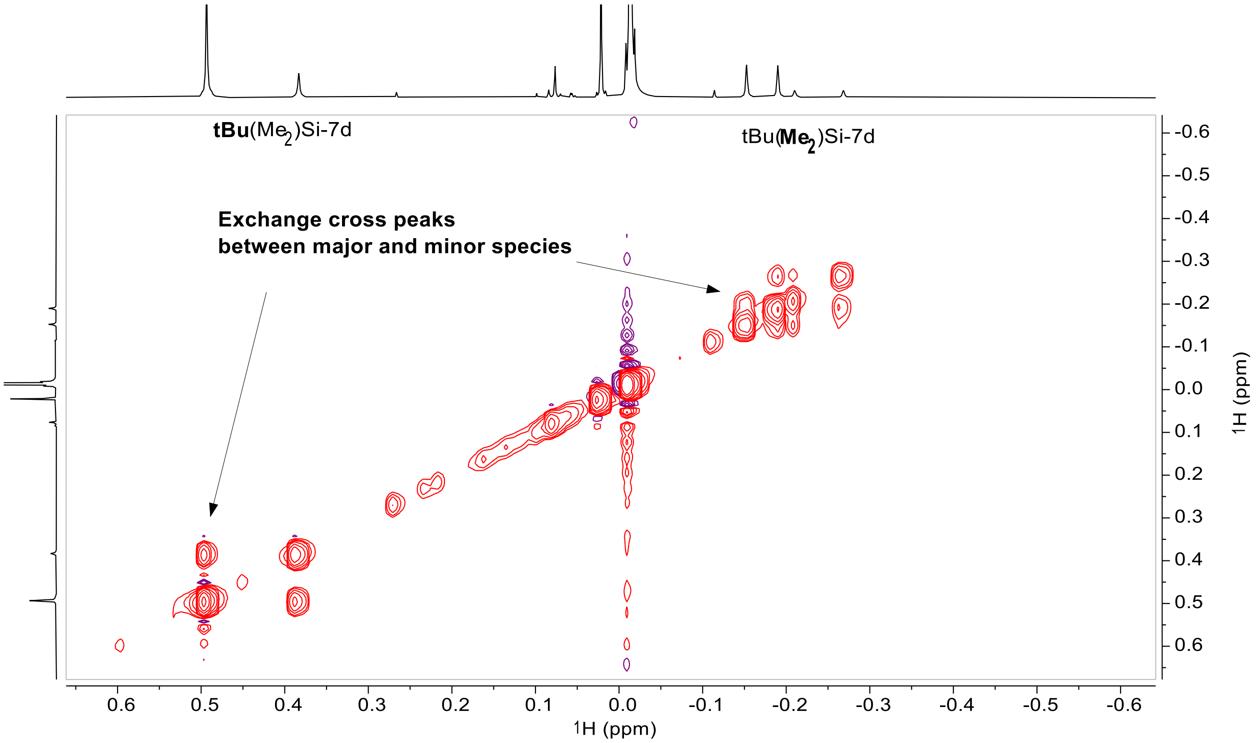

The 2D ROESY data shows ROE cross peaks of aromatic NMR signals to TBS groups (blue cross peaks), which shows, that these signals indeed arise from a silylated catalyst species. Additionally, the chemical interconversion of the minor and major species can also be shown as exchange (EXSY) cross peaks (red cross peaks) are observed in the dataset.

Determination of the absolute configuration

Method 1. Correlation with the known values of optical rotations reported in the literature (Table S1):

Table S1. Measured values of optical rotation vs values documented in the literature

Compound/ Structure	Measured $[\alpha]_D^T$	Literature
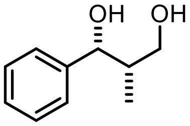 **S1a**	$[\alpha]_D^{25}$ = +42.4 (*c* = 0.75, $CHCl_3$) **1*R*, 2*S***	$[\alpha]_D^{27}$ = +56.1 (*c* = 0.75, $CHCl_3$)[15] **1*R*, 2S**
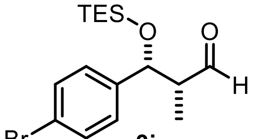 **3j**	$[\alpha]_D^{25}$ = +62.0 (*c* = 0.10, CH_2Cl_2) **2*R*,3*R***	$[\alpha]_D^{25}$ = −27.01 (*c* = 1.00, CH_2Cl_2)[16] **2*S*,3*S***
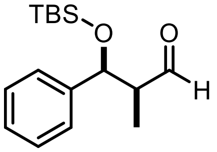 ***ent*-3b**	$[\alpha]_D^{20}$ = −29.8 (*c* = 1, $CHCl_3$). **2*S*,3*S***	$[\alpha]_D^{20}$ = −34.4 (*c* = 1, $CHCl_3$).[5] **2*S*,3*S***
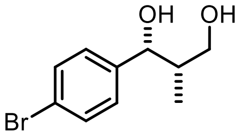 **S1j**	$[\alpha]_D^{25}$ =+39.6 (*c* = 0.45, $CHCl_3$) **1*R*, 2*S***	$[\alpha]_D^{23.9}$ = −44.4 (c = 0.50, $CHCl_3$)[17] **1*S*, 2*R***
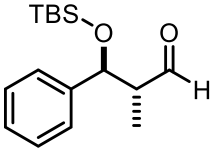 **5b**	$[\alpha]_D^{25}$ = −102.6 (*c* = 0.28, $CHCl_3$). **2*R*,3*S***	$[\alpha]_D^{20}$= +111 (*c* = 0.3, $CHCl_3$)[18] **2*S*,3*R***
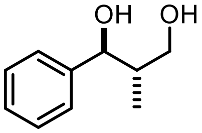 **S5b**	$[\alpha]_D^{25}$ = −38.1 (*c* = 0.31, $CHCl_3$) **1*S*,2*S***	$[\alpha]_D^{25}$= –37.5 (*c* = 0.40, $CHCl_3$)[19] **1*S*,2*S***

Method 2: X-ray crystallography of 1,3-diol derivatives (see Crystallographic data).

X-ray crystallographic analysis of *syn*-1,3-diols, derived from both aromatic and heteroaromatic aldehydes (**S1j** and **S1i**, respectively) shows the same absolute configuration, allowing safe generalization of the assigned absolute configuration to other *syn*-aldols and their derivatives. X-ray crystallographic analysis of *anti*-1,3-diol **S5k**, which is a diastereomer of *syn*-1,3-diol **S1i** confirms that, inversion of enantiofacial selectivity of aldehyde attack has taken place. Additionally, X-ray crystallographic data is in agreement with the assignment of absolute configuration via correlation to literature values of optical rotations according to Method 1.

Crystallographic data

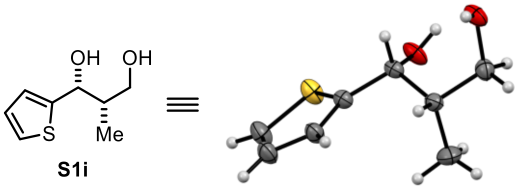

S1i

Table S2. Crystal data and structure refinement.

Identification code	13453	
Empirical formula	$C_8 H_{12} O_2 S$	
Color	colourless	
Formula weight	172.24 g · mol^{-1}	
Temperature	100(2) K	
Wavelength	0.71073 Å	
Crystal system	ORTHORHOMBIC	
Space group	**$P2_12_12_1$, (no. 19)**	
Unit cell dimensions	a = 5.5449(4) Å	α= 90°.
	b = 9.2585(9) Å	β= 90°.
	c = 16.6934(9) Å	γ = 90°.
Volume	857.00(11) Å^3	
Z	4	
Density (calculated)	1.335 Mg · m^{-3}	
Absorption coefficient	0.325 mm^{-1}	
F(000)	368 e	
Crystal size	0.345 x 0.17 x 0.125 mm^3	
θ range for data collection	3.286 to 35.972°.	
Index ranges	$-9 \le h \le 9$, $-15 \le k \le 15$, $-27 \le l \le 27$	
Reflections collected	23090	
Independent reflections	4043 [R_{int} = 0.0330]	
Reflections with I>2σ(I)	3358	
Completeness to θ = 25.242°	99.5 %	
Absorption correction	Gaussian	
Max. and min. transmission	0.97 and 0.91	
Refinement method	Full-matrix least-squares on F^2	
Data / restraints / parameters	4043 / 0 / 125	
Goodness-of-fit on F^2	1.025	
Final R indices [I>2σ(I)]	R_1 = 0.0312	wR^2 = 0.0822
R indices (all data)	R_1 = 0.0429	wR^2 = 0.0861
Absolute structure parameter	0.051(17)	
Largest diff. peak and hole	0.3 and -0.3 e · Å^{-3}	

Table S3. Bond lengths [Å] and angles [°].

S(1)-C(5)	1.7226(13)	S(1)-C(8)	1.7135(14)
O(1)-H(1)	0.80(2)	O(1)-C(1)	1.4280(15)
O(2)-H(2)	0.77(2)	O(2)-C(3)	1.4264(18)
C(1)-H(1A)	0.97(2)	C(1)-C(2)	1.5385(18)
C(1)-C(5)	1.5092(16)	C(2)-H(2A)	0.984(19)
C(2)-C(3)	1.5324(18)	C(2)-C(4)	1.528(2)
C(3)-H(3A)	1.06(2)	C(3)-H(3B)	0.97(2)
C(5)-C(6)	1.3817(17)	C(6)-H(6)	0.9500
C(7)-C(8)	1.359(2)		
C(8)-S(1)-C(5)	92.05(7)	C(1)-O(1)-H(1)	113.6(16)
C(3)-O(2)-H(2)	108.2(15)	O(1)-C(1)-H(1A)	109.0(11)
O(1)-C(1)-C(2)	111.91(10)	O(1)-C(1)-C(5)	106.89(10)
C(2)-C(1)-H(1A)	108.7(11)	C(5)-C(1)-H(1A)	107.4(10)
C(5)-C(1)-C(2)	112.84(10)	C(1)-C(2)-H(2A)	109.3(11)
C(3)-C(2)-C(1)	110.56(10)	C(3)-C(2)-H(2A)	108.0(11)
C(4)-C(2)-C(1)	112.95(12)	C(4)-C(2)-H(2A)	107.3(11)
C(4)-C(2)-C(3)	108.62(11)	O(2)-C(3)-C(2)	114.29(11)
O(2)-C(3)-H(3A)	102.2(11)	O(2)-C(3)-H(3B)	108.0(13)
C(2)-C(3)-H(3A)	112.3(12)	C(2)-C(3)-H(3B)	112.2(12)
H(3A)-C(3)-H(3B)	107.2(15)	C(1)-C(5)-S(1)	119.34(9)
C(6)-C(5)-S(1)	111.28(10)	C(6)-C(5)-C(1)	129.35(12)
C(5)-C(6)-C(7)	111.83(13)	C(8)-C(7)-C(6)	112.95(12)
C(7)-C(8)-S(1)	111.88(11)		

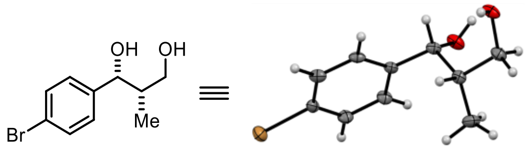

S1j

Table S4. Crystal data and structure refinement.

Identification code	12694	
Empirical formula	$C_{10} H_{13} Br O_2$	
Color	colourless	
Formula weight	245.11 g · mol^{-1}	
Temperature	100(2) K	
Wavelength	0.71073 Å	
Crystal system	ORTHORHOMBIC	
Space group	**$P2_12_12_1$, (no. 19)**	
Unit cell dimensions	a = 16.8808(8) Å	α= 90°.
	b = 6.0030(4) Å	β= 90°.
	c = 10.0455(6) Å	γ = 90°.
Volume	1017.97(10) Å^3	
Z	4	
Density (calculated)	1.599 Mg · m^{-3}	
Absorption coefficient	4.003 mm^{-1}	
F(000)	496 e	
Crystal size	0.11 x 0.10 x 0.09 mm^3	
θ range for data collection	3.152 to 30.504°.	
Index ranges	$-23 \le h \le 24$, $-8 \le k \le 8$, $-14 \le l \le 14$	
Reflections collected	17851	
Independent reflections	3106 [R_{int} = 0.0462]	
Reflections with I>2σ(I)	3026	
Completeness to θ = 25.242°	99.5 %	
Absorption correction	Gaussian	
Max. and min. transmission	0.73 and 0.68	
Refinement method	Full-matrix least-squares on F^2	
Data / restraints / parameters	3106 / 0 / 123	
Goodness-of-fit on F^2	1.402	
Final R indices [I>2σ(I)]	R_1 = 0.0359	wR^2 = 0.0967
R indices (all data)	R_1 = 0.0375	wR^2 = 0.0974
Absolute structure parameter	0.04(2)	
Extinction coefficient	0.0086(16)	
Largest diff. peak and hole	0.7 and -1.2 e · Å^{-3}	

Table S5. Bond lengths [Å] and angles [°].

Br(1)-C(8)	1.906(5)	O(1)-C(1)	1.432(6)
O(2)-C(3)	1.430(6)	C(1)-C(2)	1.524(7)
C(2)-C(3)	1.545(6)	C(2)-C(4)	1.530(6)
C(3)-C(5)	1.510(6)	C(5)-C(6)	1.395(7)
C(5)-C(10)	1.395(6)	C(6)-C(7)	1.393(7)
C(7)-C(8)	1.394(7)	C(8)-C(9)	1.371(7)
C(9)-C(10)	1.401(6)		
O(1)-C(1)-C(2)	113.4(4)	C(1)-C(2)-C(3)	111.1(4)
C(1)-C(2)-C(4)	108.9(4)	C(4)-C(2)-C(3)	112.5(4)
O(2)-C(3)-C(2)	111.3(4)	O(2)-C(3)-C(5)	109.4(4)
C(5)-C(3)-C(2)	110.9(4)	C(6)-C(5)-C(3)	119.6(4)
C(6)-C(5)-C(10)	118.4(4)	C(10)-C(5)-C(3)	122.0(4)
C(7)-C(6)-C(5)	121.9(4)	CC(6)-C(7)-C(8)	117.6(5)
C(7)-C(8)-Br(1)	118.6(4)	C(9)-C(8)-Br(1)	119.0(4)
C(9)-C(8)-C(7)	122.4(4)	C(8)-C(9)-C(10)	118.9(4)
C(5)-C(10)-C(9)	120.8(4)		

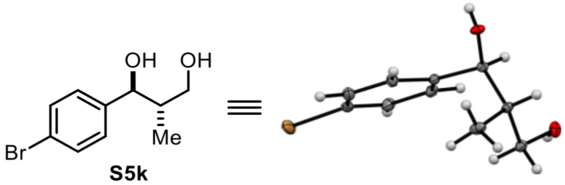

S5k

Table S6. Crystal data and structure refinement.

Identification code	13244	
Empirical formula	$C_{10}H_{13}BrO_2$	
Color	colourless	
Formula weight	245.11 g·mol^{-1}	
Temperature	100(2) K	
Wavelength	0.71073 Å	
Crystal system	monoclinic	
Space group	*P*2$_1$, (no. 4)	
Unit cell dimensions	a = 5.9023(3) Å	α= 90°.
	b = 8.1019(4) Å	β= 101.840(2)°.
	c = 10.9807(5) Å	γ = 90°.
Volume	513.92(4) Å^3	
Z	2	
Density (calculated)	1.584 Mg·m^{-3}	
Absorption coefficient	3.965 mm^{-1}	
F(000)	248 e	
Crystal size	0.080 x 0.058 x 0.022 mm^3	
θ range for data collection	1.895 to 34.335°.	
Index ranges	$-9 \le h \le 9$, $-12 \le k \le 12$, $-17 \le l \le 17$	
Reflections collected	26463	
Independent reflections	4297 [R_{int} = 0.0224]	
Reflections with I>2σ(I)	4134	
Completeness to θ = 25.242°	100.0 %	
Absorption correction	Gaussian	
Max. and min. transmission	0.93707 and 0.82376	
Refinement method	Full-matrix least-squares on F^2	
Data / restraints / parameters	4297 / 1 / 124	
Goodness-of-fit on F^2	1.037	
Final R indices [I>2σ(I)]	R_1 = 0.0182	wR^2 = 0.0432
R indices (all data)	R_1 = 0.0197	wR^2 = 0.0438
Absolute structure parameter	0.008(6)	
Extinction coefficient	0	
Largest diff. peak and hole	0.960 and -0.865 e·Å^{-3}	

Table S7. Bond lengths [Å] and angles [°].

Br(1)-C(8)	1.8987(15)	O(1)-C(1)	1.4359(16)
O(1)-H(1A)	0.77(3)	O(2)-C(3)	1.438(2)
O(2)-H(2A)	0.73(3)	C(1)-C(5)	1.5136(19)
C(1)-C(2)	1.5380(19)	C(1)-H(1)	1.0000
C(2)-C(3)	1.527(2)	C(2)-C(4)	1.529(2)
C(2)-H(2)	1.0000	C(3)-H(3A)	0.9900
C(3)-H(3B)	0.9900	C(4)-H(4A)	0.9800
C(4)-H(4B)	0.9800	C(4)-H(4C)	0.9800
C(5)-C(6)	1.394(2)	C(5)-C(10)	1.401(2)
C(6)-C(7)	1.396(2)	C(6)-H(6)	0.9500
C(7)-C(8)	1.389(2)	C(7)-H(7)	0.9500
C(8)-C(9)	1.393(2)	C(9)-C(10)	1.390(2)
C(9)-H(9)	0.9500	C(10)-H(10)	0.9500
C(1)-O(1)-H(1A)	109.5	C(3)-O(2)-H(2A)	109.5
O(1)-C(1)-C(5)	111.25(11)	O(1)-C(1)-C(2)	107.39(11)
C(5)-C(1)-C(2)	113.98(11)	O(1)-C(1)-H(1)	108.0
C(5)-C(1)-H(1)	108.0	C(2)-C(1)-H(1)	108.0
C(3)-C(2)-C(4)	109.02(13)	C(3)-C(2)-C(1)	111.48(12)
C(4)-C(2)-C(1)	113.78(12)	C(3)-C(2)-H(2)	107.4
C(4)-C(2)-H(2)	107.4	C(1)-C(2)-H(2)	107.4
O(2)-C(3)-C(2)	111.06(13)	O(2)-C(3)-H(3A)	109.4
C(2)-C(3)-H(3A)	109.4	O(2)-C(3)-H(3B)	109.4
C(2)-C(3)-H(3B)	109.4	H(3A)-C(3)-H(3B)	108.0
C(2)-C(4)-H(4A)	109.5	C(2)-C(4)-H(4B)	109.5
H(4A)-C(4)-H(4B)	109.5	C(2)-C(4)-H(4C)	109.5
H(4A)-C(4)-H(4C)	109.5	H(4B)-C(4)-H(4C)	109.5
C(6)-C(5)-C(10)	118.41(14)	C(6)-C(5)-C(1)	119.99(13)
C(10)-C(5)-C(1)	121.59(13)	C(5)-C(6)-C(7)	121.50(15)
C(5)-C(6)-H(6)	119.3	C(7)-C(6)-H(6)	119.3
C(8)-C(7)-C(6)	118.64(15)	C(8)-C(7)-H(7)	120.7
C(6)-C(7)-H(7)	120.7	C(7)-C(8)-C(9)	121.28(15)
C(7)-C(8)-Br(1)	118.99(12)	C(9)-C(8)-Br(1)	119.73(12)
C(10)-C(9)-C(8)	119.11(15)	C(10)-C(9)-H(9)	120.4
C(8)-C(9)-H(9)	120.4	C(9)-C(10)-C(5)	121.02(14)
C(9)-C(10)-H(10)	119.5	C(5)-C(10)-H(10)	119.5

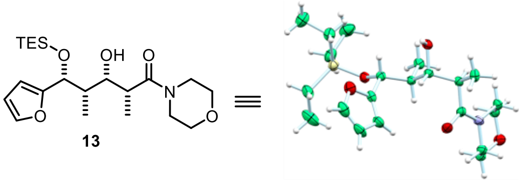

Table S8. Crystal data and structure refinement.

Identification code	13887	
Empirical formula	C_{21} H_{37} N O_5 Si	
Color	colourless	
Formula weight	411.60 g·mol^{-1}	
Temperature	100(2) K	
Wavelength	0.71073 Å	
Crystal system	Monoclinic	
Space group	$P2_1$, (No. 4)	
Unit cell dimensions	a = 9.8303(6) Å	α= 90°.
	b = 9.6564(6) Å	β= 108.913(3)°.
	c = 13.0039(8) Å	γ = 90°.
Volume	1167.76(13) Å^3	
Z	2	
Density (calculated)	1.171 Mg·m^{-3}	
Absorption coefficient	0.130 mm^{-1}	
F(000)	448 e	
Crystal size	0.077 x 0.072 x 0.033 mm^3	
θ range for data collection	1.655 to 29.129°.	
Index ranges	$-13 \le h \le 13$, $-13\le k \le 13$, $-17 \le l \le 17$	
Reflections collected	32428	
Independent reflections	6284 [R_{int} = 0.0563]	
Reflections with I>2σ(I)	4785	
Completeness to θ = 25.242°	100.0 %	
Absorption correction	Gaussian	
Max. and min. transmission	0.99663 and 0.99352	
Refinement method	Full-matrix least-squares on F^2	
Data / restraints / parameters	6284 / 1 / 271	
Goodness-of-fit on F^2	1.036	
Final R indices [I>2σ(I)]	R_1 = 0.0524	wR^2 = 0.1219
R indices (all data)	R_1 = 0.0836	wR^2 = 0.1364
Absolute structure parameter	-0.01(7)	
Extinction coefficient	n/a	
Largest diff. peak and hole	0.373 and -0.343 e·Å^{-3}	

Table 9. Bond lengths [Å] and angles [°].

Si(1)-O(1)	1.646(3)	Si(1)-C(20)	1.868(4)
Si(1)-C(18)	1.866(4)	Si(1)-C(16)	1.877(4)
O(2)-H(2)	0.8400	O(2)-C(4)	1.430(4)
O(1)-C(1)	1.434(4)	O(4)-C(10)	1.411(5)
O(4)-C(9)	1.431(4)	O(3)-C(7)	1.238(4)
O(5)-C(12)	1.364(4)	O(5)-C(15)	1.360(5)
N(1)-C(7)	1.341(4)	N(1)-C(11)	1.473(4)
N(1)-C(8)	1.470(5)	C(7)-C(5)	1.519(5)
C(3)-H(3A)	0.9800	C(3)-H(3B)	0.9800
C(3)-H(3C)	0.9800	C(3)-C(2)	1.527(4)
C(1)-H(1)	1.0000	C(1)-C(2)	1.541(5)
C(1)-C(12)	1.503(5)	C(4)-H(4)	1.0000
C(4)-C(5)	1.540(5)	C(4)-C(2)	1.537(4)
C(5)-C(6)	1.532(5)	C(5)-H(5)	0.94(4)
C(2)-H(2A)	1.0000	C(11)-C(10)	1.500(6)
C(11)-H(11A)	0.92(5)	C(11)-H(11B)	0.98(4)
C(8)-H(8A)	0.9900	C(8)-H(8B)	0.9900
C(8)-C(9)	1.512(5)	C(10)-H(10A)	0.9900
C(10)-H(10B)	0.9900	C(9)-H(9A)	0.9900
C(9)-H(9B)	0.9900	C(12)-C(13)	1.331(5)
C(13)-H(13)	0.9500	C(13)-C(14)	1.436(6)
C(6)-H(6A)	0.9800	C(6)-H(6B)	0.9800
C(6)-H(6C)	0.9800	C(15)-H(15)	0.9500
C(15)-C(14)	1.322(7)	C(20)-H(20A)	0.9900
C(20)-H(20B)	0.9900	C(20)-C(21)	1.530(6)
C(14)-H(14)	0.9500	C(18)-H(18A)	0.9900
C(18)-H(18B)	0.9900	C(18)-C(19)	1.512(8)
C(16)-H(16A)	0.9900	C(16)-H(16B)	0.9900
C(16)-C(17)	1.522(6)	C(21)-H(21A)	0.9800
C(21)-H(21B)	0.9800	C(21)-H(21C)	0.9800
C(19)-H(19A)	0.9800	C(19)-H(19B)	0.9800
C(19)-H(19C)	0.9800	C(17)-H(17A)	0.9800
C(17)-H(17B)	0.9800	C(17)-H(17C)	0.9800

O(1)-Si(1)-C(20)	105.00(17)	O(1)-Si(1)-C(18)	110.23(18)
O(1)-Si(1)-C(16)	109.50(16)	C(20)-Si(1)-C(16)	110.3(2)
C(18)-Si(1)-C(20)	110.3(2)	C(18)-Si(1)-C(16)	111.4(2)
C(4)-O(2)-H(2)	109.5	C(1)-O(1)-Si(1)	125.7(2)
C(10)-O(4)-C(9)	110.0(3)	C(15)-O(5)-C(12)	106.9(3)
C(7)-N(1)-C(11)	127.5(3)	C(7)-N(1)-C(8)	120.6(3)
C(8)-N(1)-C(11)	111.7(3)	O(3)-C(7)-N(1)	120.7(3)
O(3)-C(7)-C(5)	119.2(3)	N(1)-C(7)-C(5)	120.1(3)
H(3A)-C(3)-H(3B)	109.5	H(3A)-C(3)-H(3C)	109.5
H(3B)-C(3)-H(3C)	109.5	C(2)-C(3)-H(3A)	109.5
C(2)-C(3)-H(3B)	109.5	C(2)-C(3)-H(3C)	109.5
O(1)-C(1)-H(1)	108.4	O(1)-C(1)-C(2)	109.3(2)
O(1)-C(1)-C(12)	110.4(3)	C(2)-C(1)-H(1)	108.4
C(12)-C(1)-H(1)	108.4	C(12)-C(1)-C(2)	111.7(3)
O(2)-C(4)-H(4)	108.8	O(2)-C(4)-C(5)	108.3(3)
O(2)-C(4)-C(2)	108.6(2)	C(5)-C(4)-H(4)	108.8
C(2)-C(4)-H(4)	108.8	C(2)-C(4)-C(5)	113.3(3)
C(7)-C(5)-C(4)	110.1(3)	C(7)-C(5)-C(6)	108.1(3)
C(7)-C(5)-H(5)	110(2)	C(4)-C(5)-H(5)	109(2)
C(6)-C(5)-C(4)	110.8(3)	C(6)-C(5)-H(5)	109(2)
C(3)-C(2)-C(1)	111.2(3)	C(3)-C(2)-C(4)	112.4(3)
C(3)-C(2)-H(2A)	108.4	C(1)-C(2)-H(2A)	108.4
C(4)-C(2)-C(1)	107.9(2)	C(4)-C(2)-H(2A)	108.4
N(1)-C(11)-C(10)	109.0(3)	N(1)-C(11)-H(11A)	112(3)
N(1)-C(11)-H(11B)	105(2)	C(10)-C(11)-H(11A)	110(3)
C(10)-C(11)-H(11B)	113(3)	H(11A)-C(11)-H(11B)	108(4)
N(1)-C(8)-H(8A)	109.7	N(1)-C(8)-H(8B)	109.7
N(1)-C(8)-C(9)	109.8(3)	H(8A)-C(8)-H(8B)	108.2
C(9)-C(8)-H(8A)	109.7	C(9)-C(8)-H(8B)	109.7
O(4)-C(10)-C(11)	111.4(3)	O(4)-C(10)-H(10A)	109.4
O(4)-C(10)-H(10B)	109.4	C(11)-C(10)-H(10A)	109.4
C(11)-C(10)-H(10B)	109.4	H(10A)-C(10)-H(10B)	108.0
O(4)-C(9)-C(8)	111.4(3)	O(4)-C(9)-H(9A)	109.3
O(4)-C(9)-H(9B)	109.3	C(8)-C(9)-H(9A)	109.3
C(8)-C(9)-H(9B)	109.3	H(9A)-C(9)-H(9B)	108.0
O(5)-C(12)-C(1)	114.8(3)	C(13)-C(12)-O(5)	109.7(3)

C(13)-C(12)-C(1)	135.4(3)	C(12)-C(13)-H(13)	126.7
C(12)-C(13)-C(14)	106.6(4)	C(14)-C(13)-H(13)	126.7
C(5)-C(6)-H(6A)	109.5	C(5)-C(6)-H(6B)	109.5
C(5)-C(6)-H(6C)	109.5	H(6A)-C(6)-H(6B)	109.5
H(6A)-C(6)-H(6C)	109.5	H(6B)-C(6)-H(6C)	109.5
O(5)-C(15)-H(15)	124.7	C(14)-C(15)-O(5)	110.5(4)
C(14)-C(15)-H(15)	124.7	Si(1)-C(20)-H(20A)	108.7
Si(1)-C(20)-H(20B)	108.7	H(20A)-C(20)-H(20B)	107.6
C(21)-C(20)-Si(1)	114.0(3)	C(21)-C(20)-H(20A)	108.7
C(21)-C(20)-H(20B)	108.7	C(13)-C(14)-H(14)	126.8
C(15)-C(14)-C(13)	106.3(4)	C(15)-C(14)-H(14)	126.8
Si(1)-C(18)-H(18A)	108.7	Si(1)-C(18)-H(18B)	108.7
H(18A)-C(18)-H(18B)	107.6	C(19)-C(18)-Si(1)	114.2(3)
C(19)-C(18)-H(18A)	108.7	C(19)-C(18)-H(18B)	108.7
Si(1)-C(16)-H(16A)	109.0	Si(1)-C(16)-H(16B)	109.0
H(16A)-C(16)-H(16B)	107.8	C(17)-C(16)-Si(1)	113.1(3)
C(17)-C(16)-H(16A)	109.0	C(17)-C(16)-H(16B)	109.0
C(20)-C(21)-H(21A)	109.5	C(20)-C(21)-H(21B)	109.5
C(20)-C(21)-H(21C)	109.5	H(21A)-C(21)-H(21B)	109.5
H(21A)-C(21)-H(21C)	109.5	H(21B)-C(21)-H(21C)	109.5
C(18)-C(19)-H(19A)	109.5	C(18)-C(19)-H(19B)	109.5
C(18)-C(19)-H(19C)	109.5	H(19A)-C(19)-H(19B)	109.5
H(19A)-C(19)-H(19C)	109.5	H(19B)-C(19)-H(19C)	109.5
C(16)-C(17)-H(17A)	109.5	C(16)-C(17)-H(17B)	109.5
C(16)-C(17)-H(17C)	109.5	H(17A)-C(17)-H(17B)	109.5
H(17A)-C(17)-H(17C)	109.5	H(17B)-C(17)-H(17C)	109.5

Copies of NMR Spectra

syn-Aldols and their derivatives

(2*R*,3*R*)-2-Methyl-3-phenyl-3-((triethylsilyl)oxy)propanal

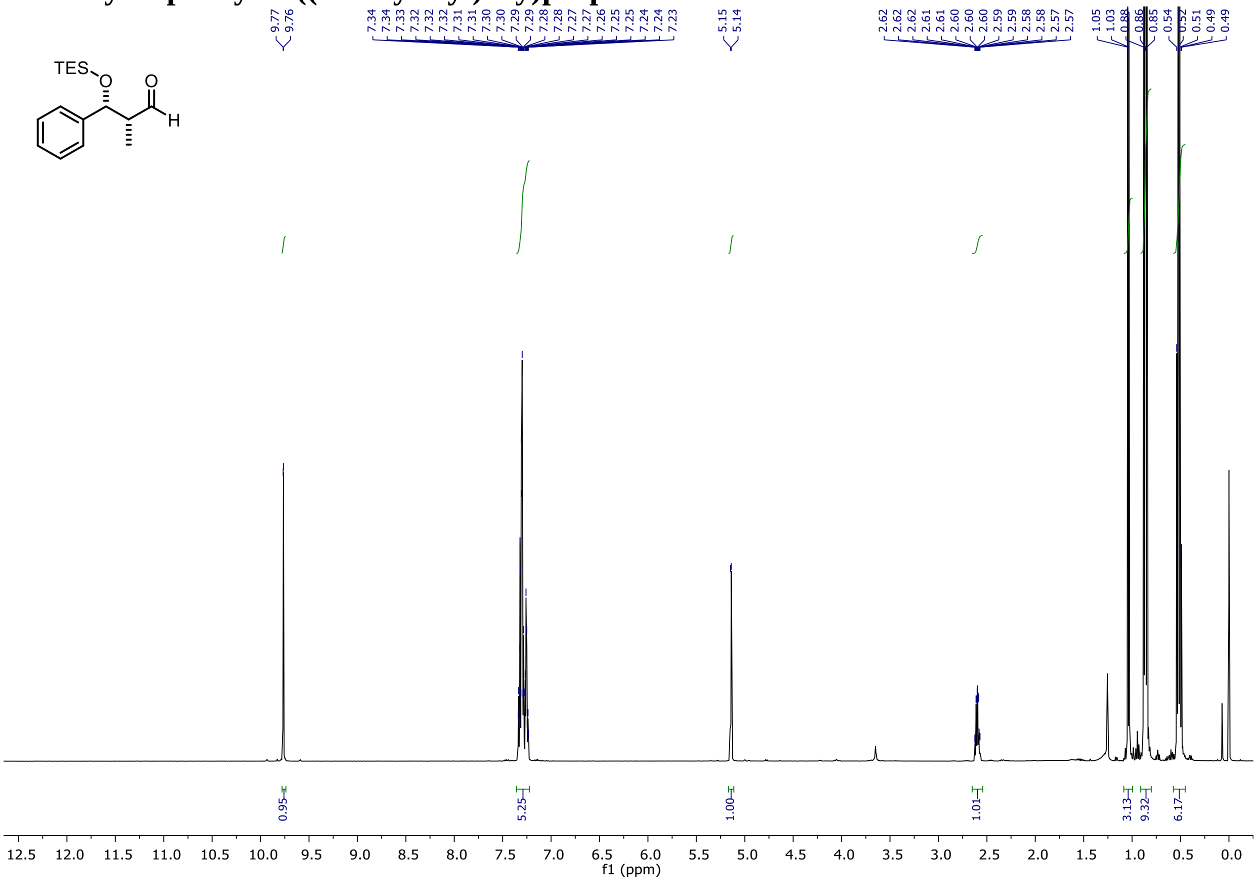

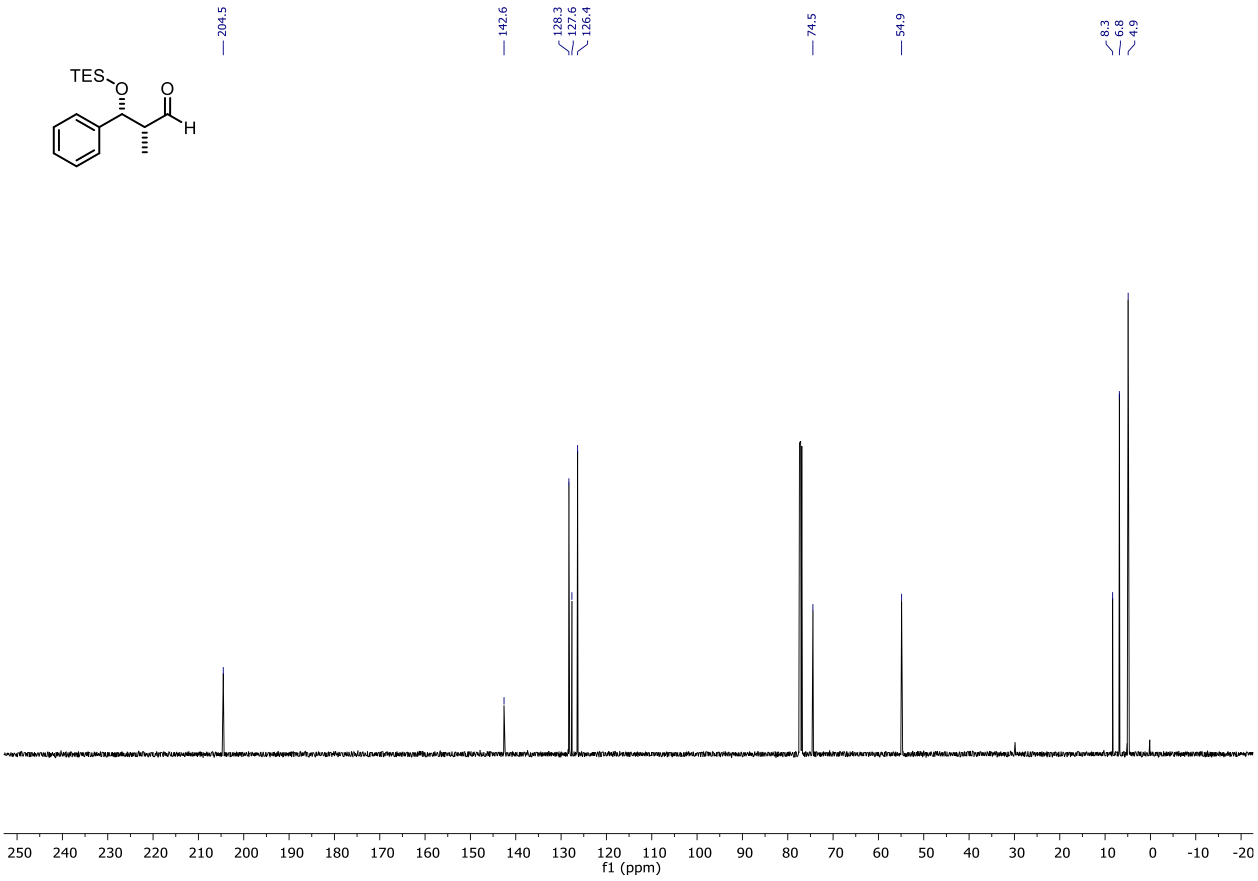

(2*R*,3*R*)-3-(3-Methoxyphenyl)-2-methyl-3-((triethylsilyl)oxy)propanal

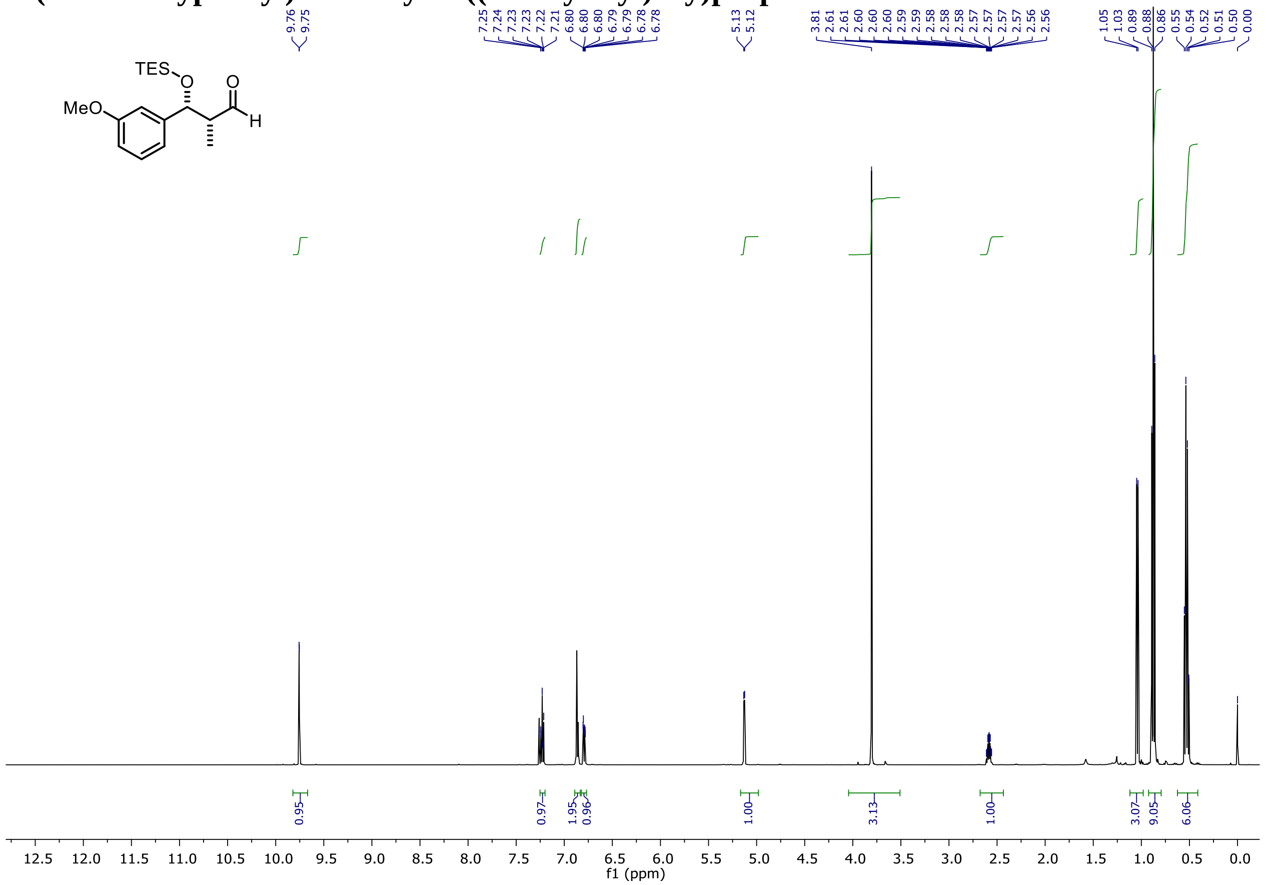

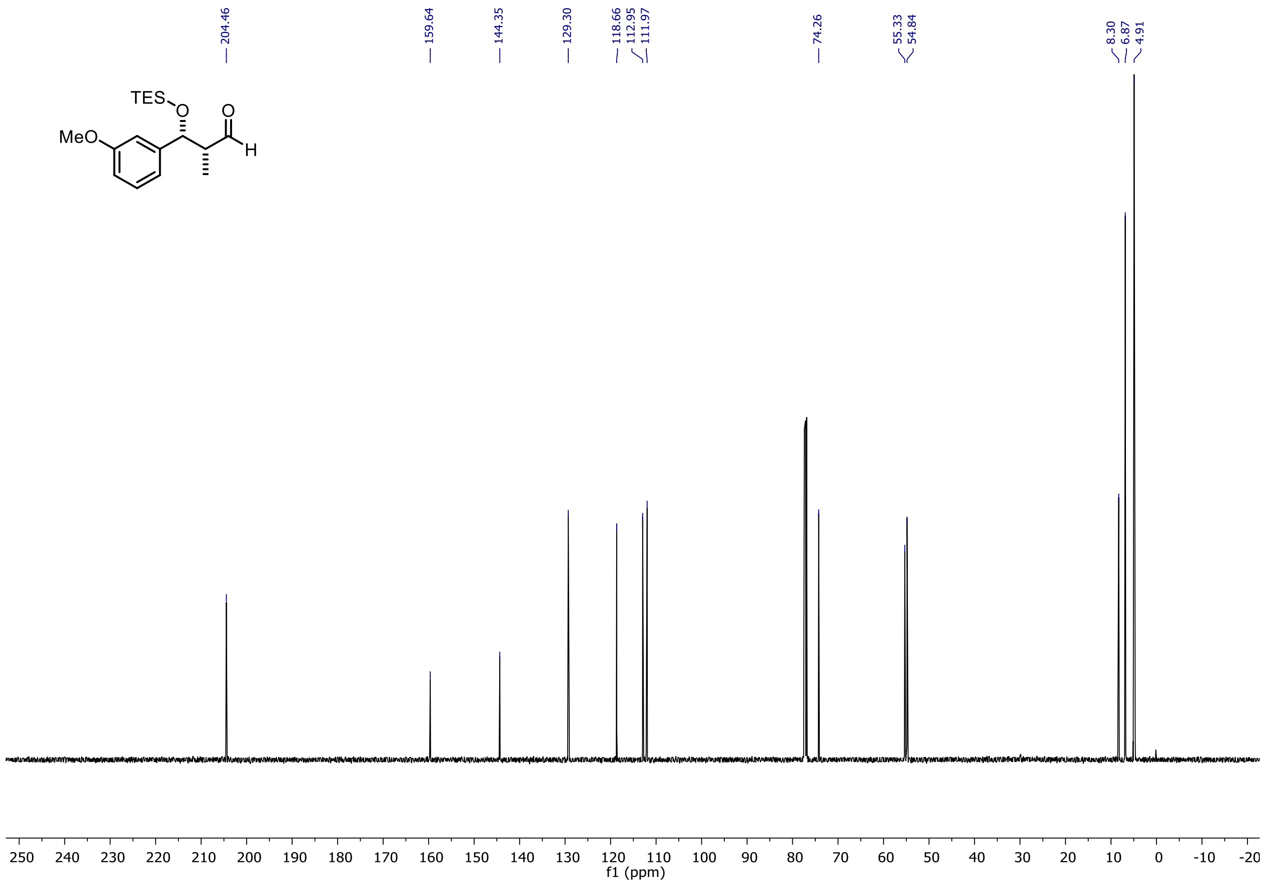

(1*R*, 2*S*)-1-(3-Methoxyphenyl)-2-methylpropane-1,3-diol

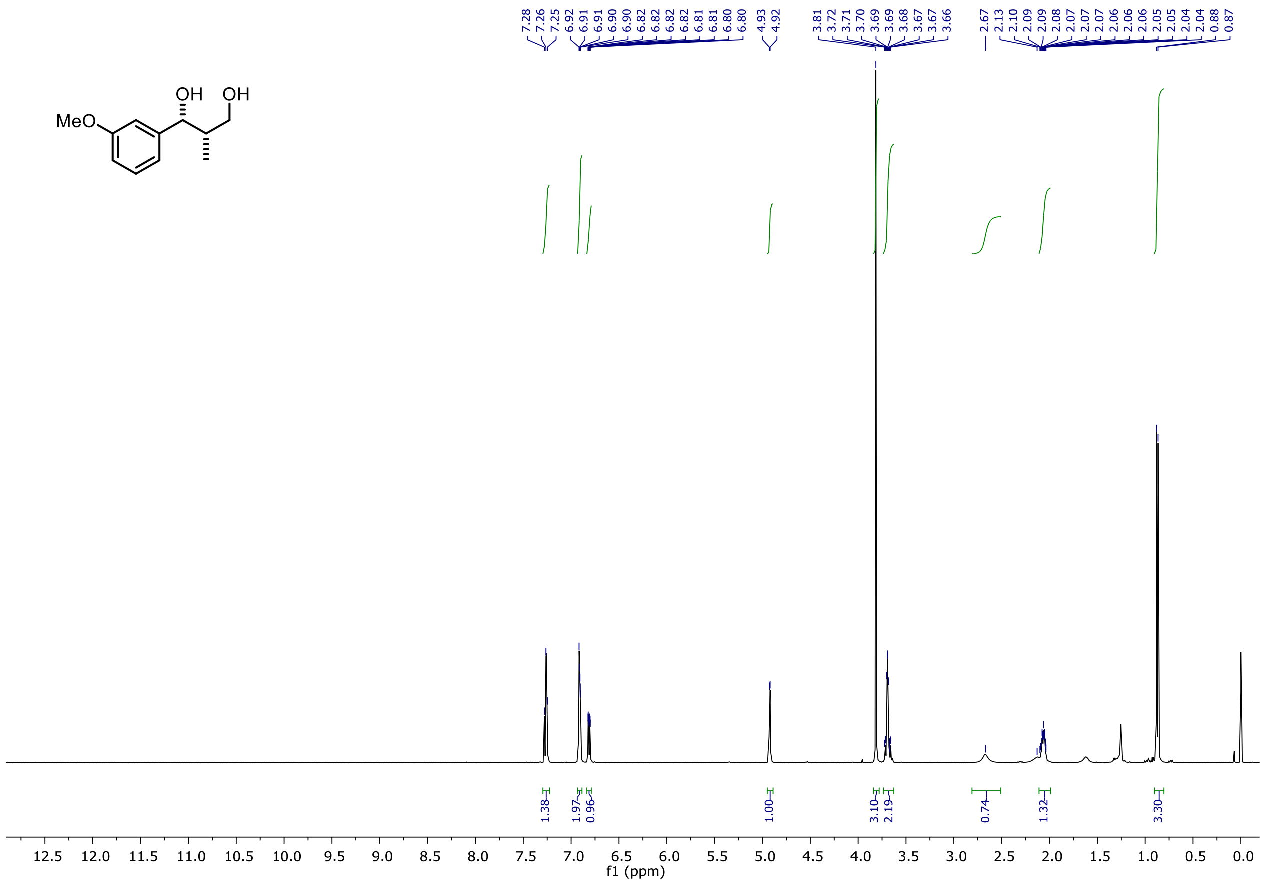

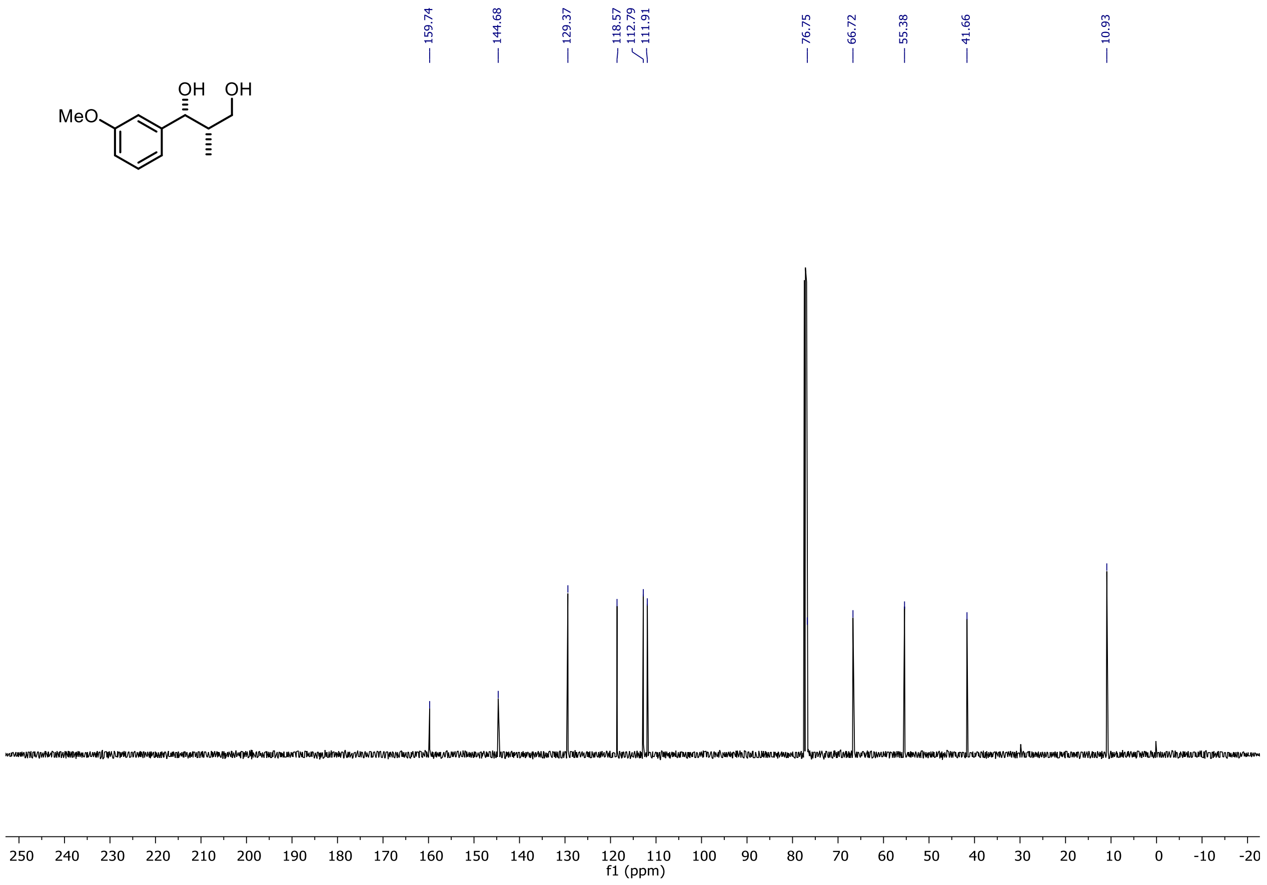

(2*R*,3*R*)-3-(4-Methoxyphenyl)-2-methyl-3-((triethylsilyl)oxy)propanal

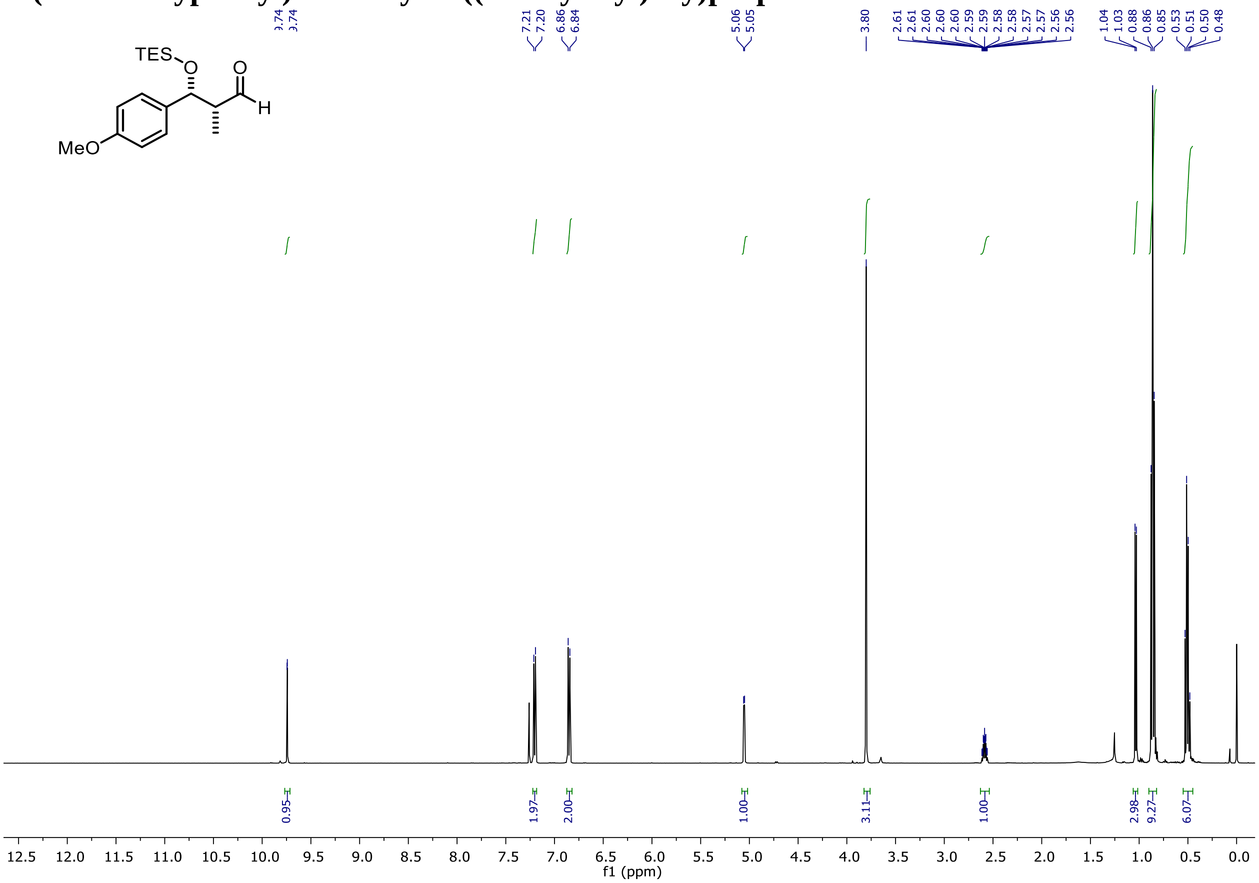

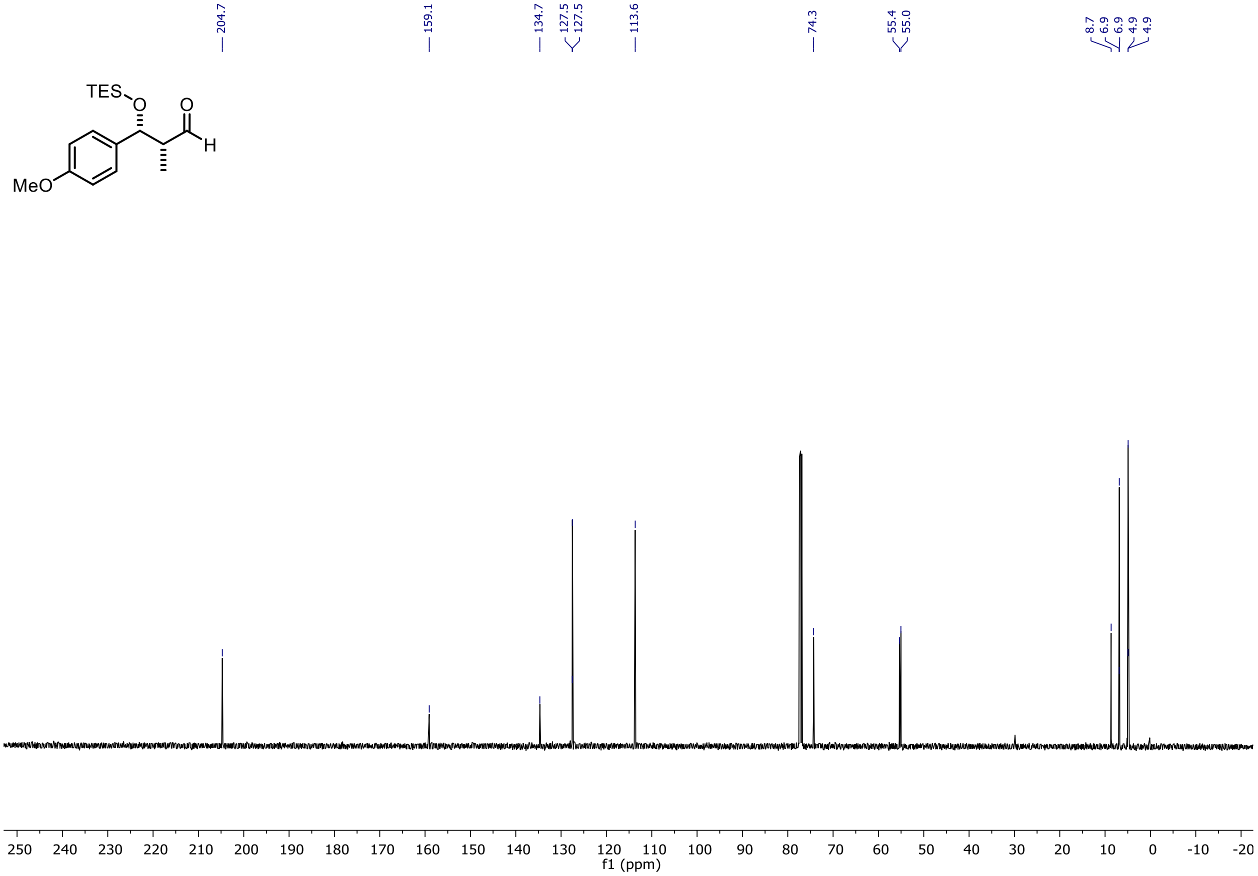

(1*R*,2*S*)-1-(4-Methoxyphenyl)-2-methylpropane-1,3-diol

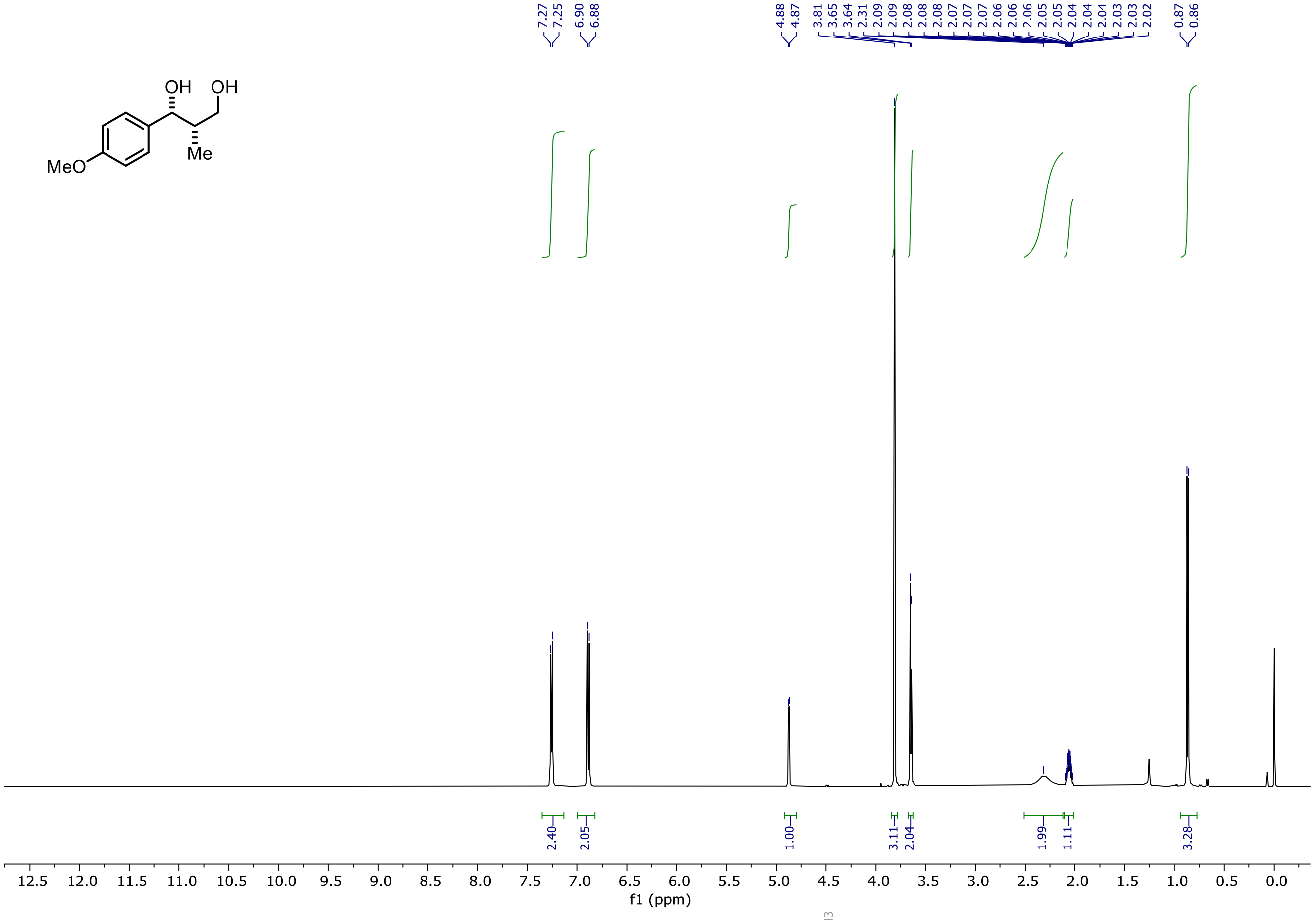

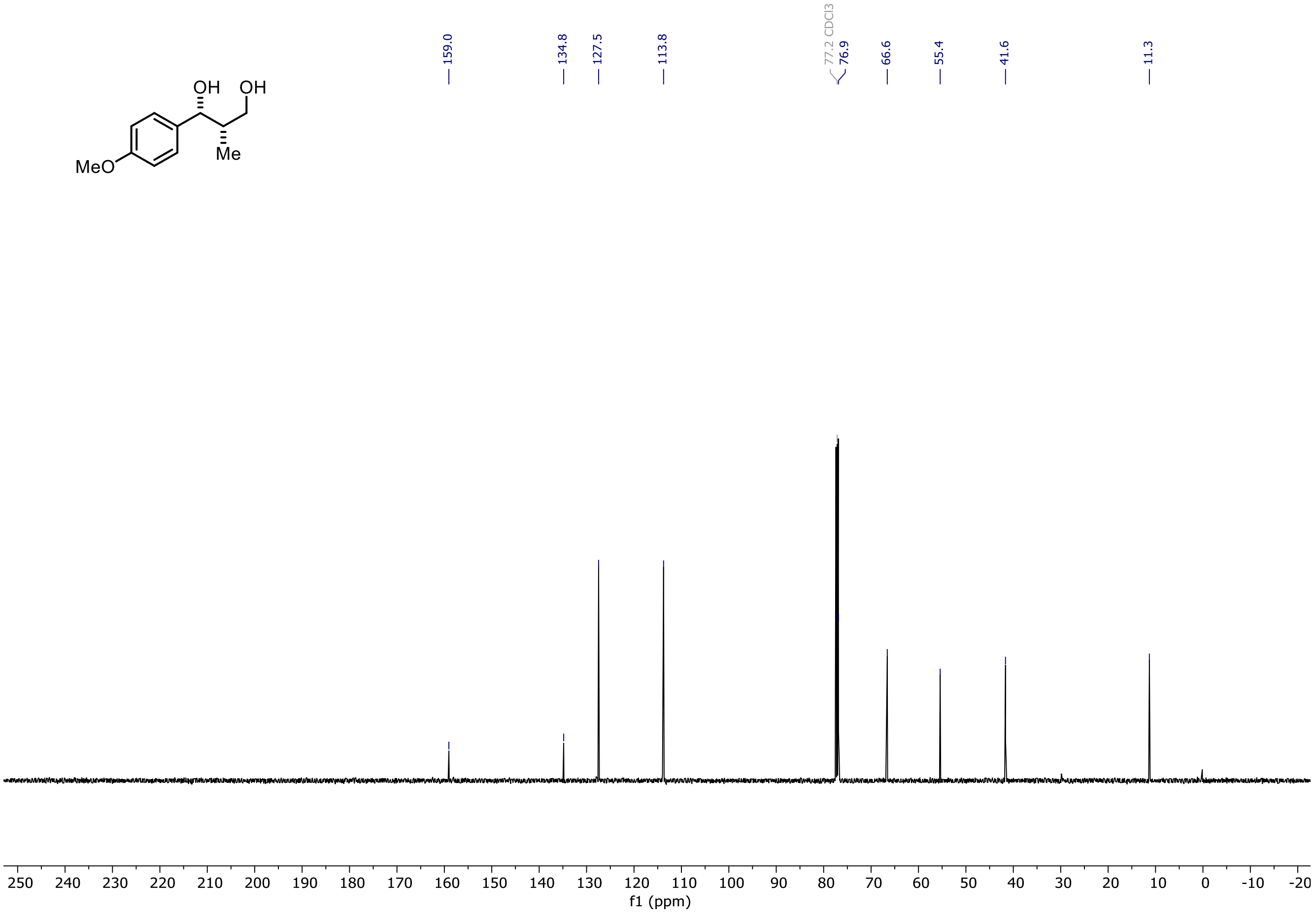

(2*R*,3*R*)-2-Methyl-3-(*o*-tolyl)-3-((triethylsilyl)oxy)propanal

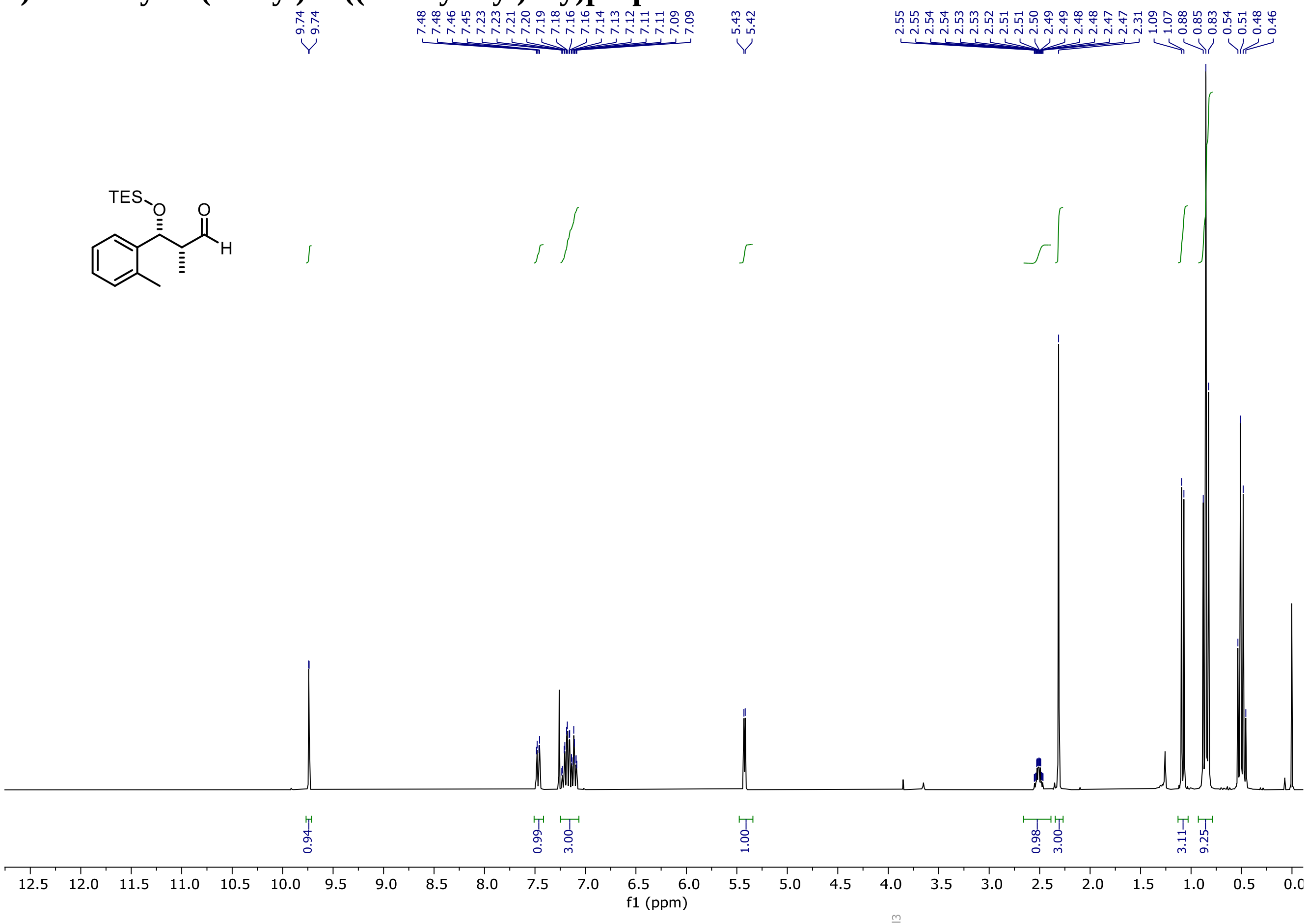

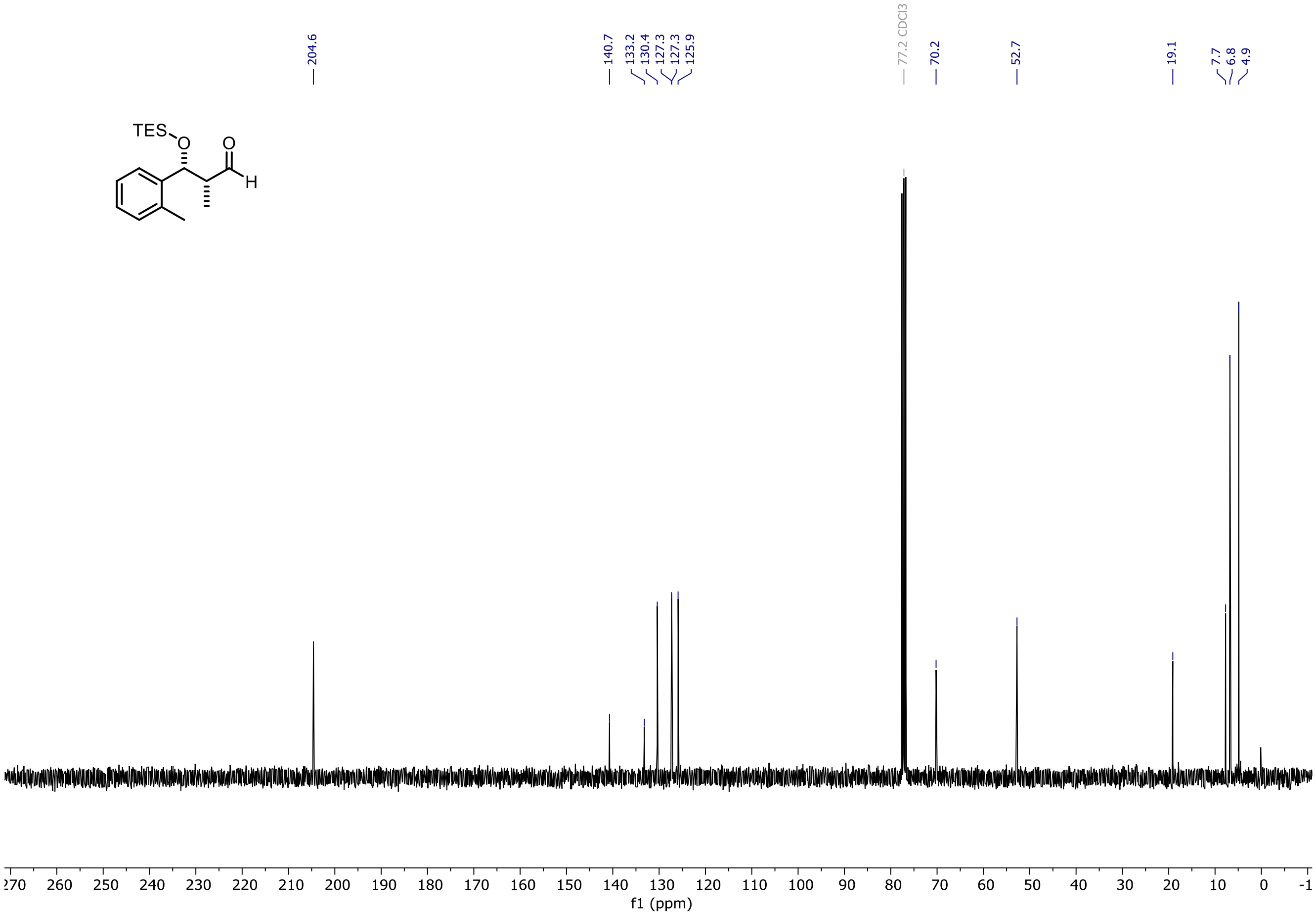

(1*R*,2*S*)-2-Methyl-1-(*o*-tolyl)propane-1,3-diol

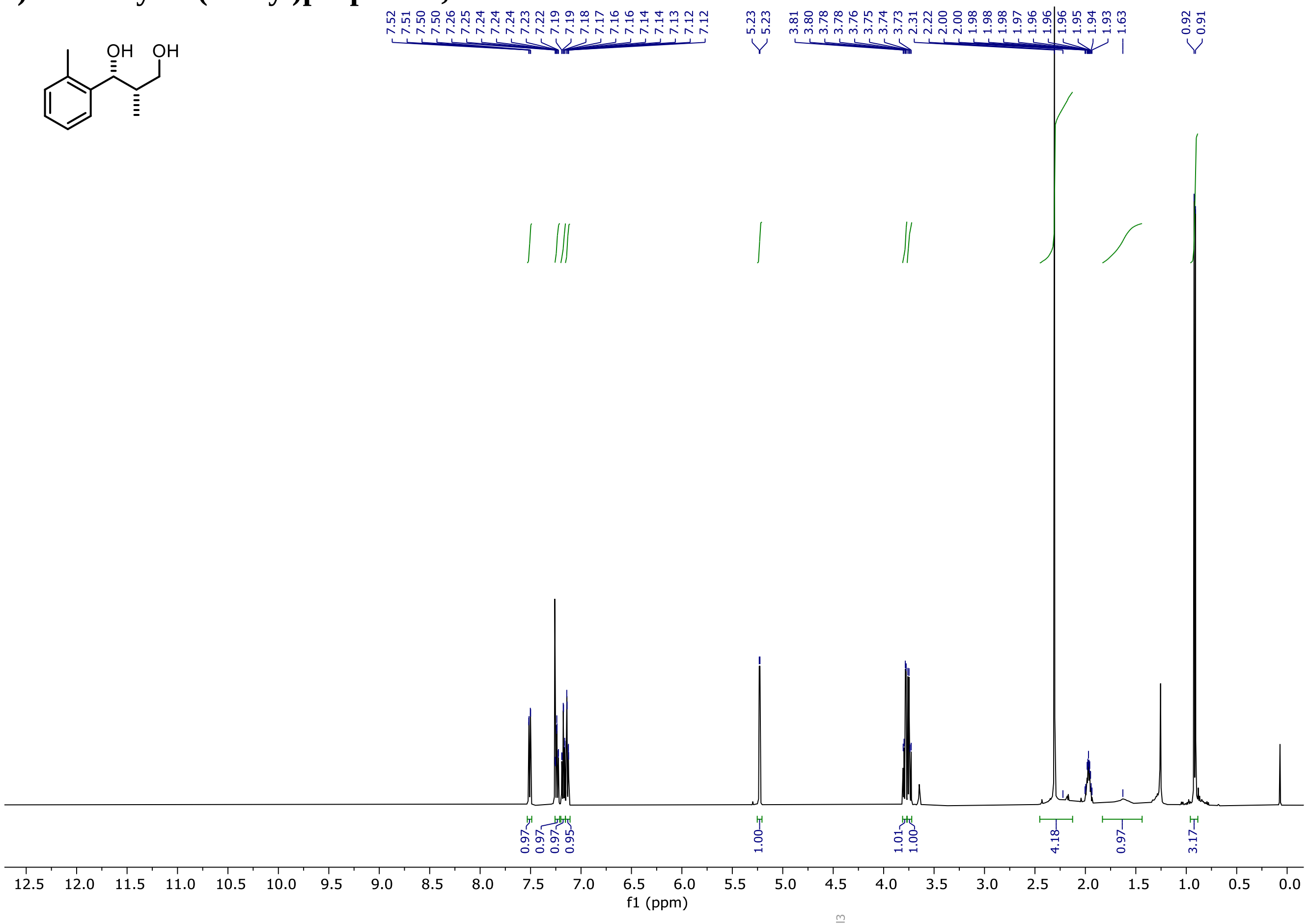

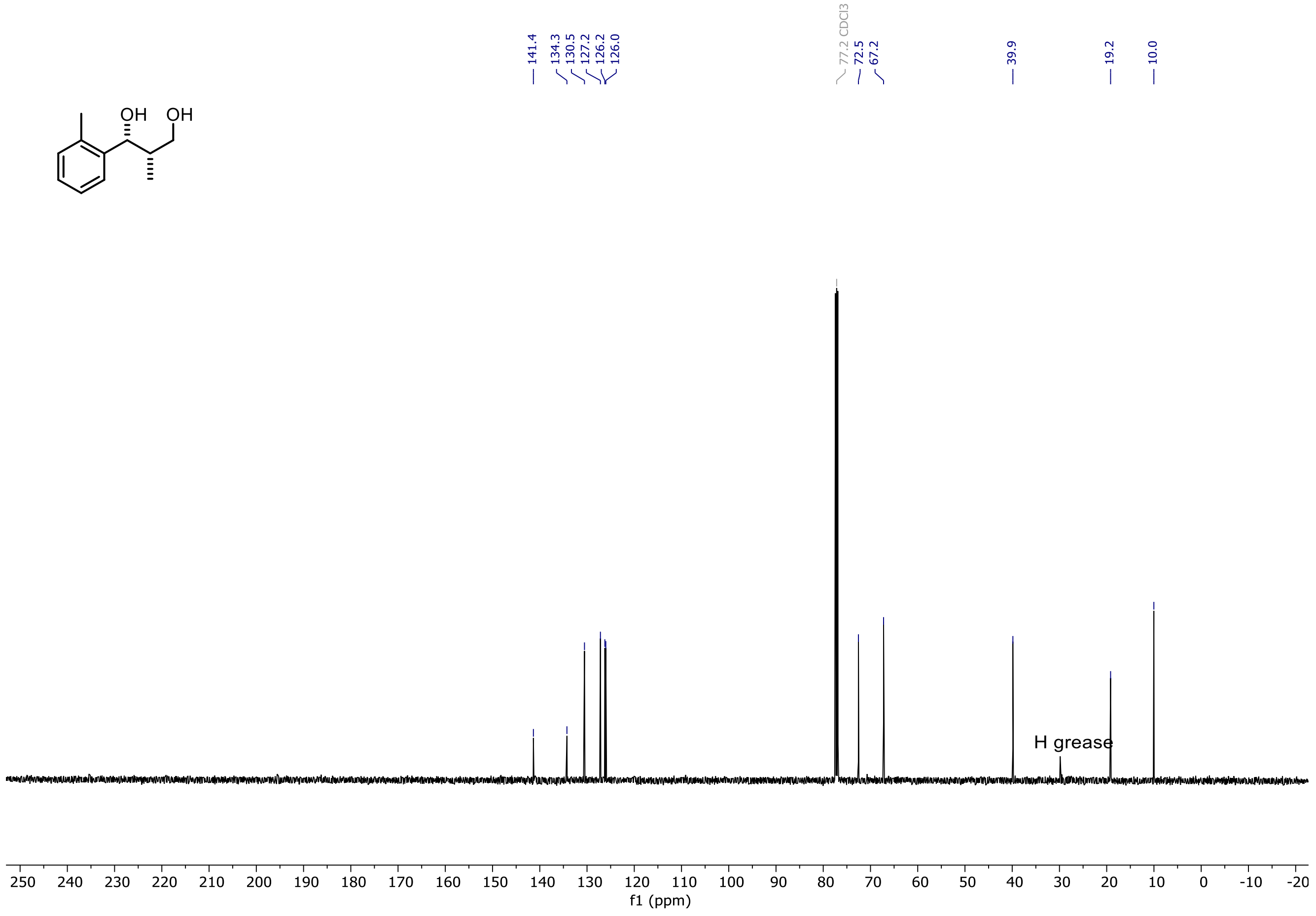

(2*R*,3*R*)-2-Methyl-3-(*m*-tolyl)-3-((triethylsilyl)oxy)propanal

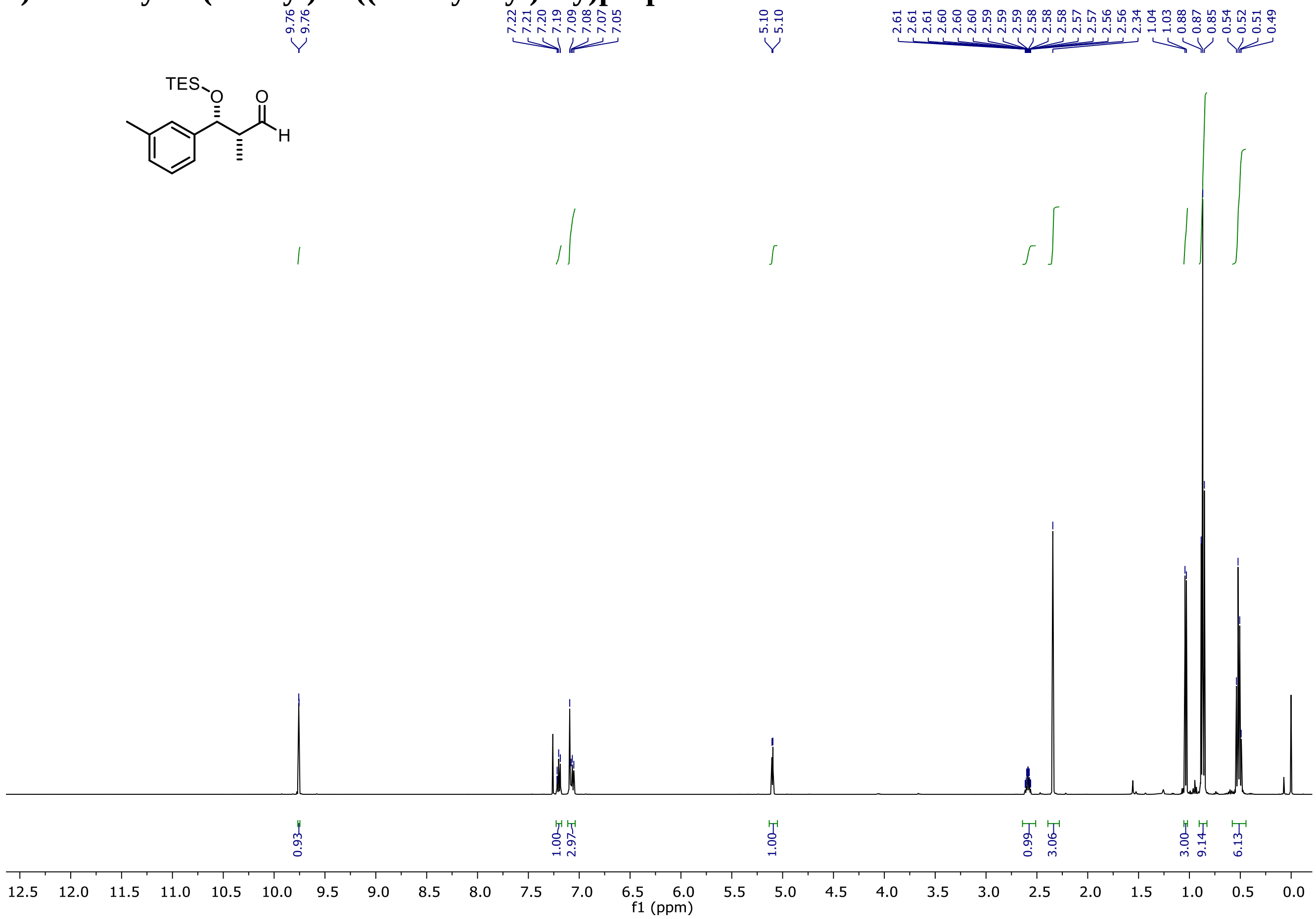

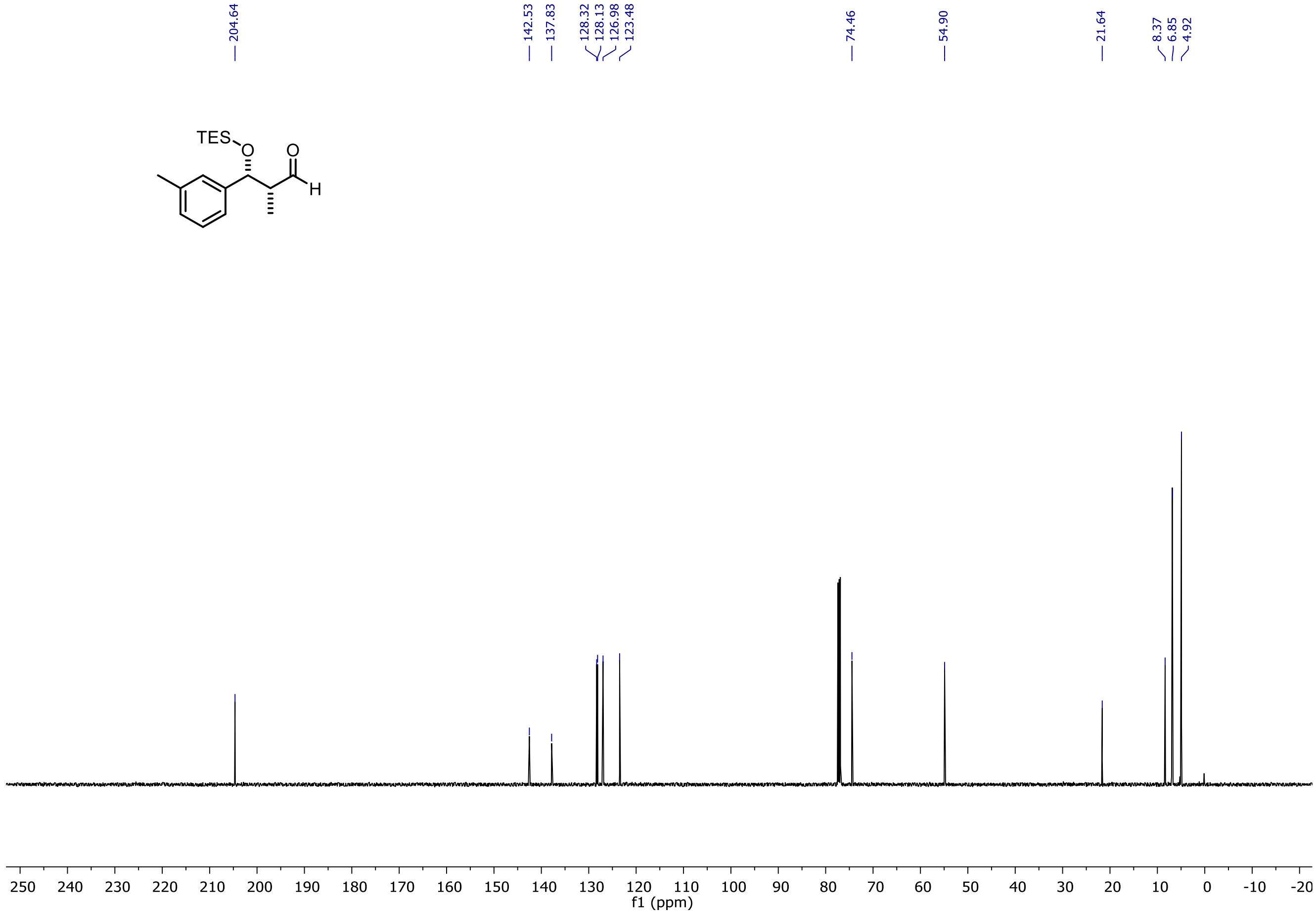

(2*R*,3*R*)-2-Methyl-3-(*p*-tolyl)-3-((triethylsilyl)oxy)propanal

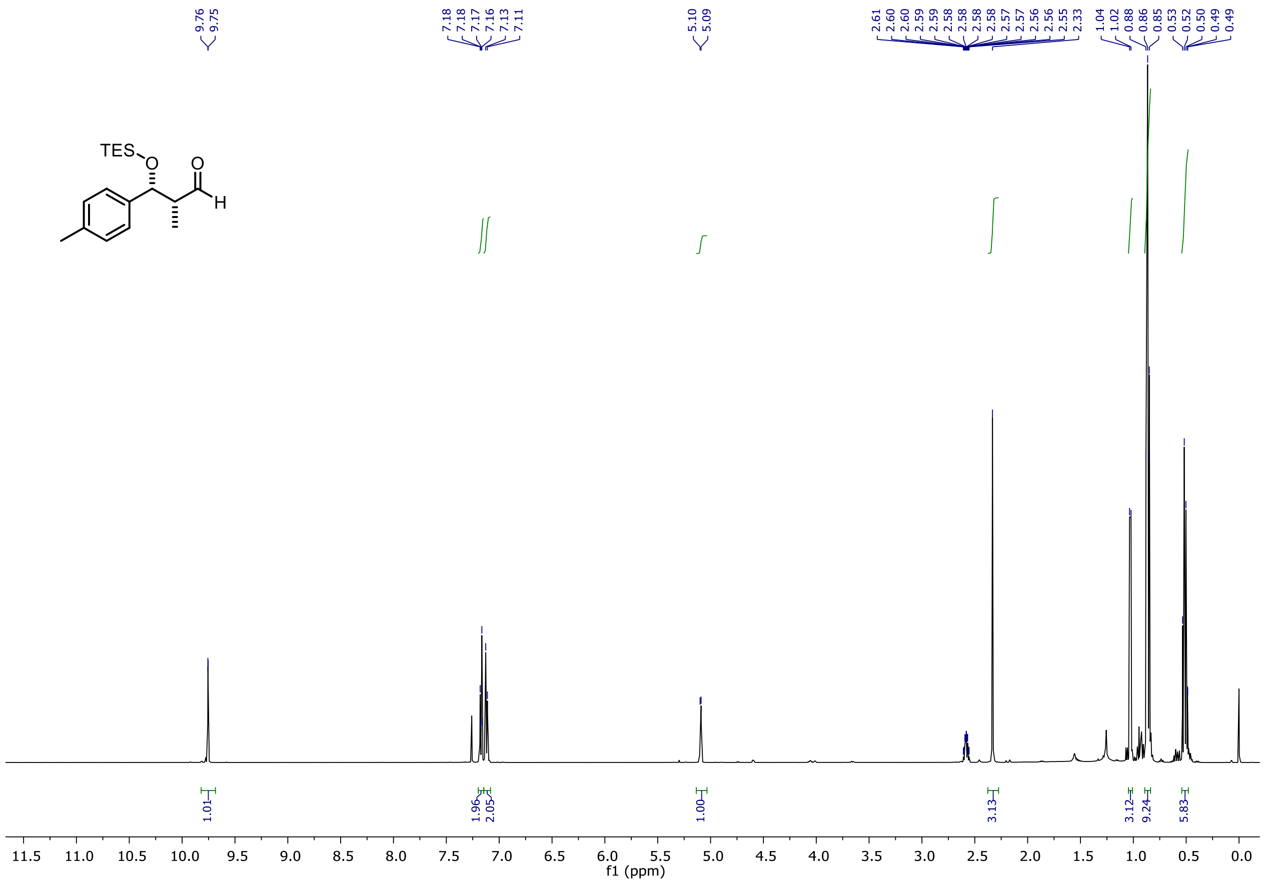

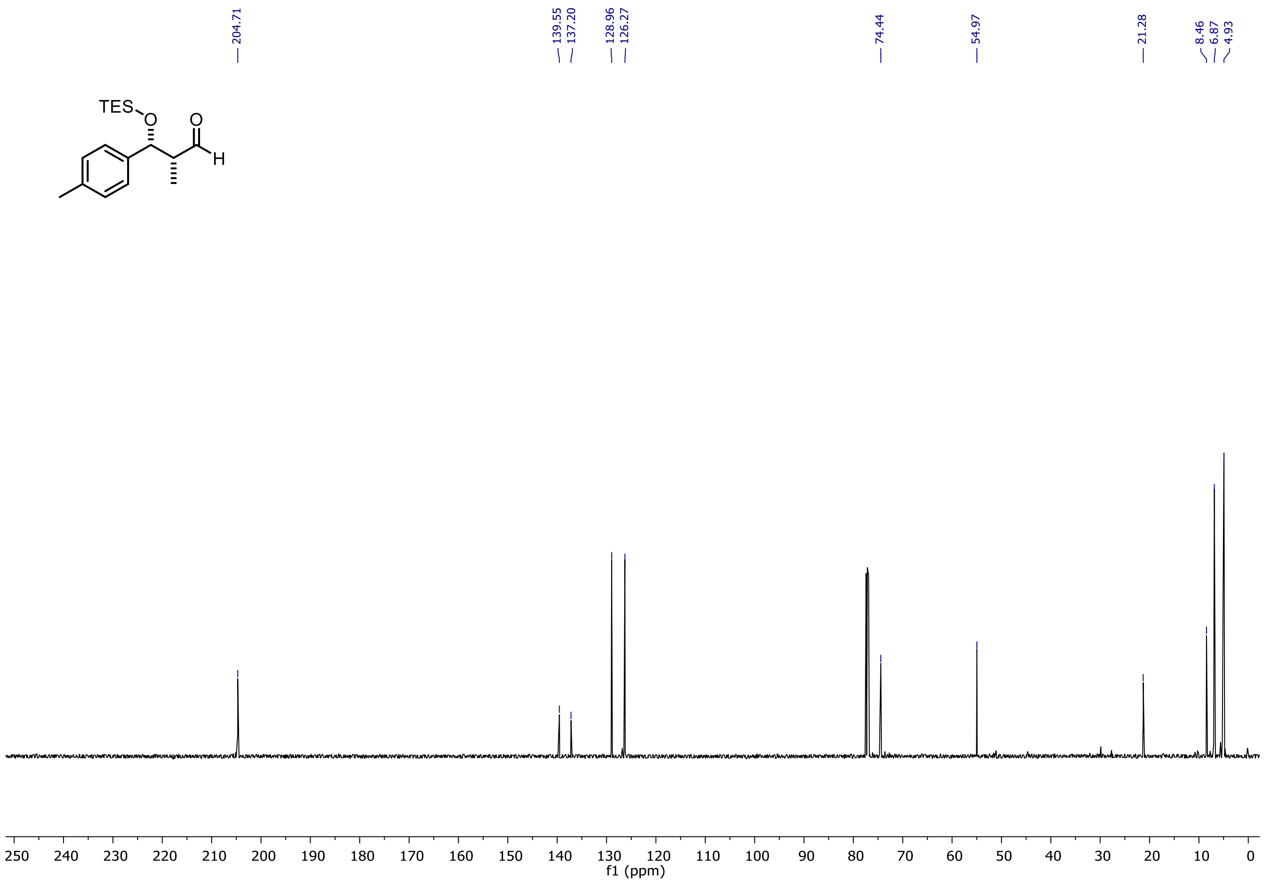

(1*R*,2*S*)-2-Methyl-1-(*p*-tolyl)propane-1,3-diol

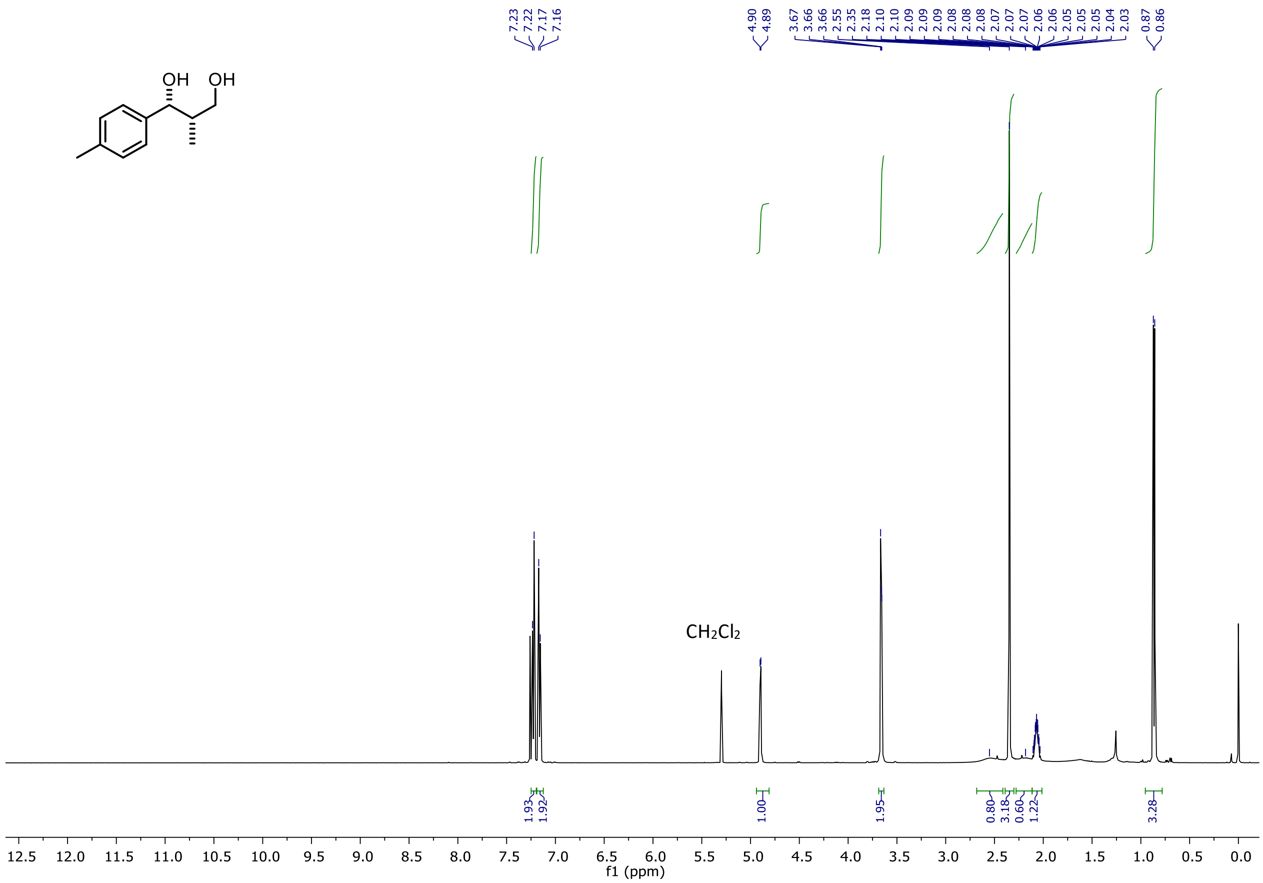

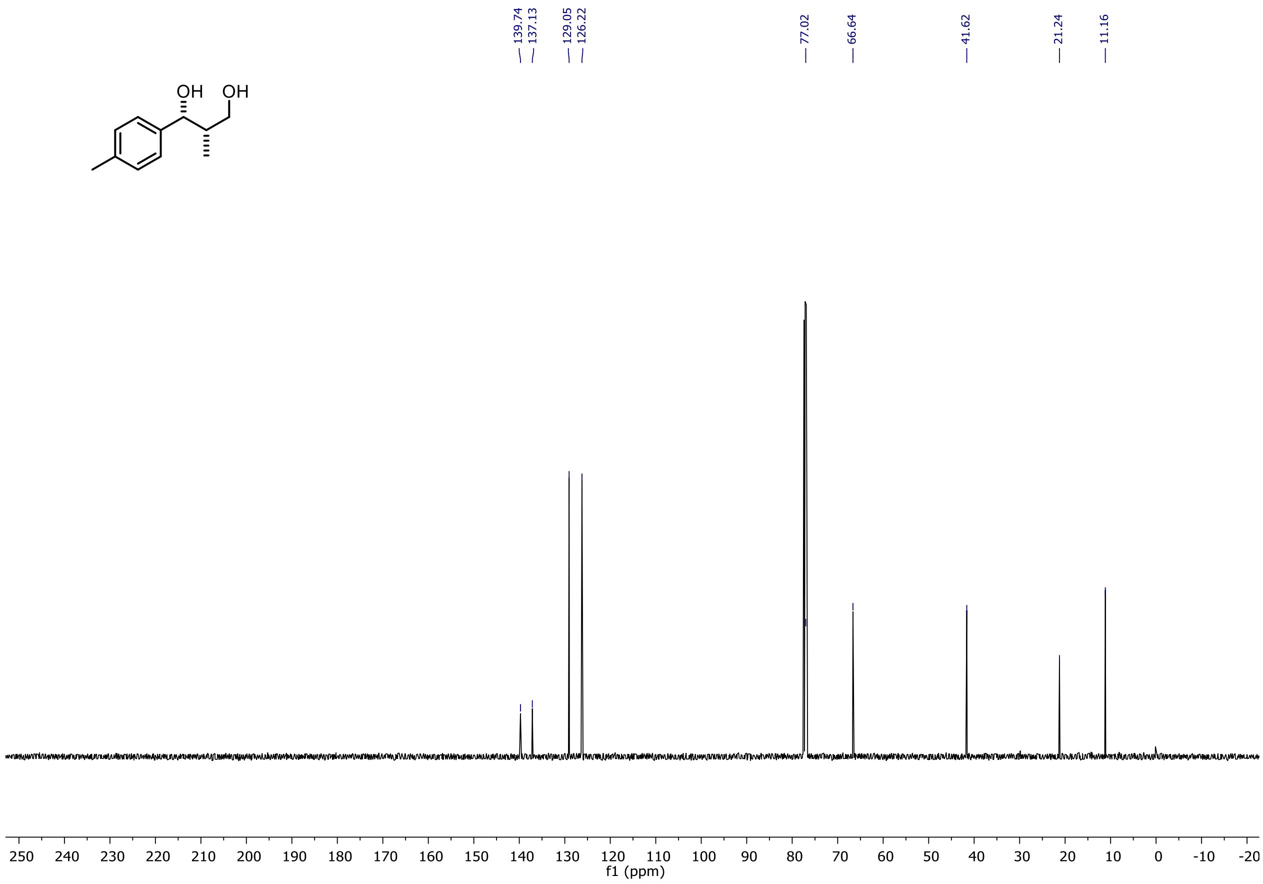

(2*R*,3*R*)-3-(Furan-2-yl)-2-methyl-3-((triethylsilyl)oxy)propanal

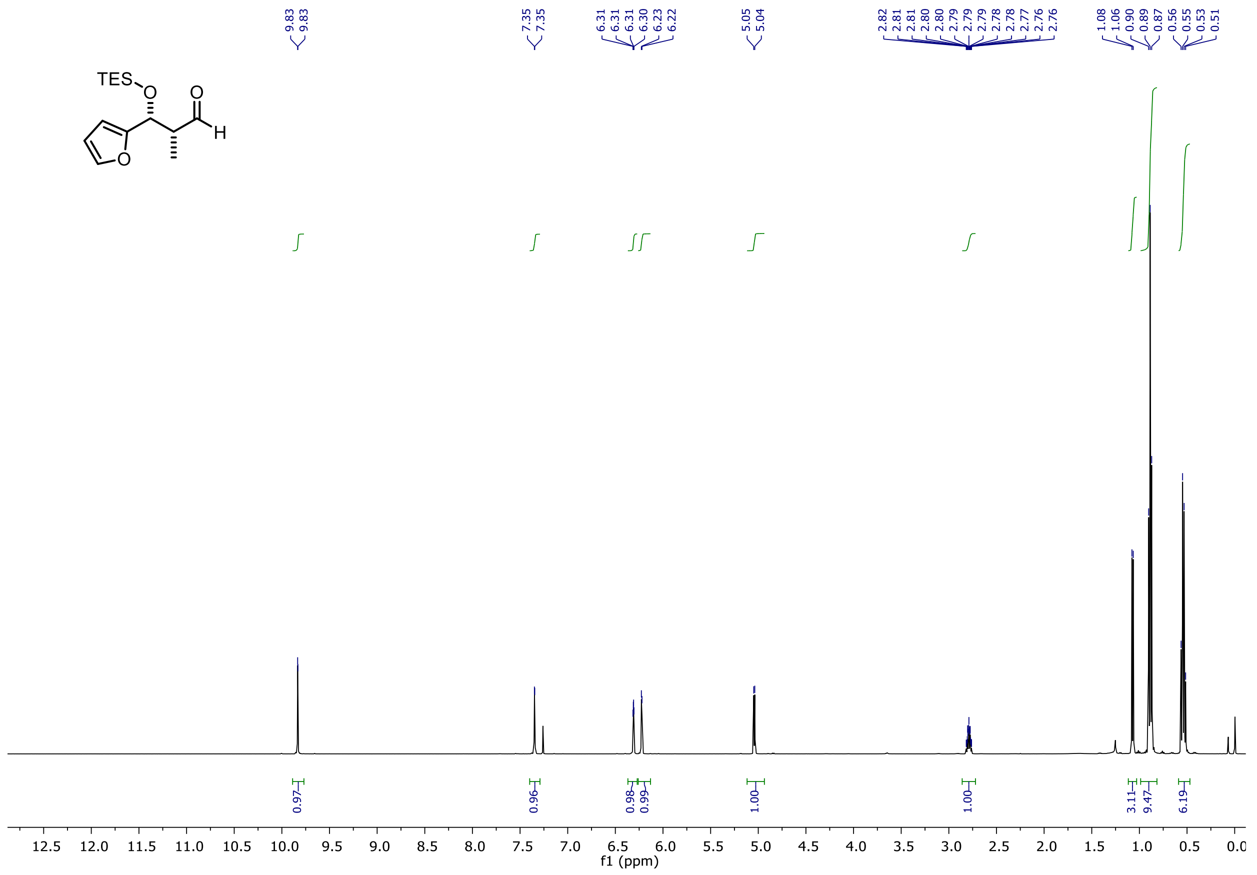

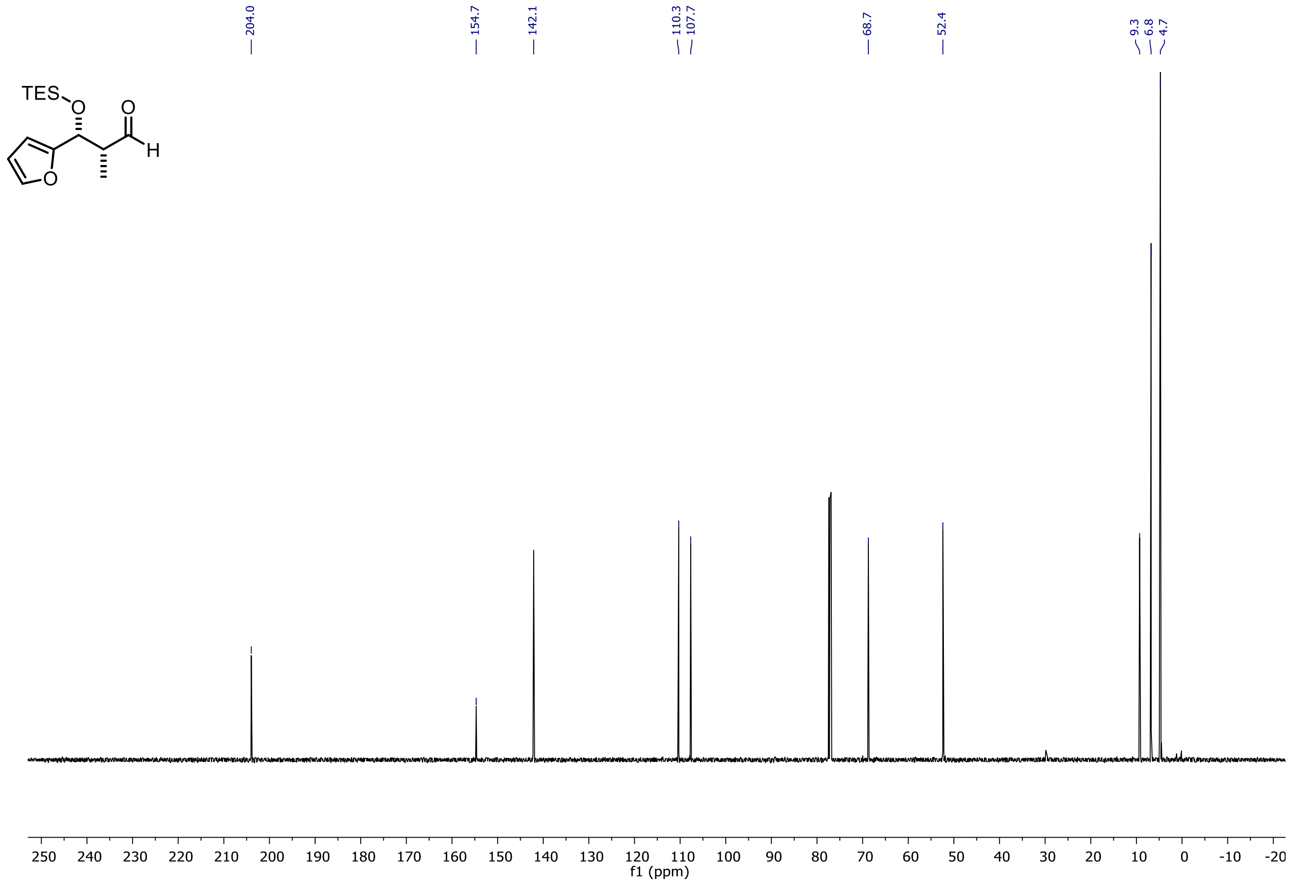

(1*R*,2*S*)-1-(Furan-2-yl)-2-methylpropane-1,3-diol

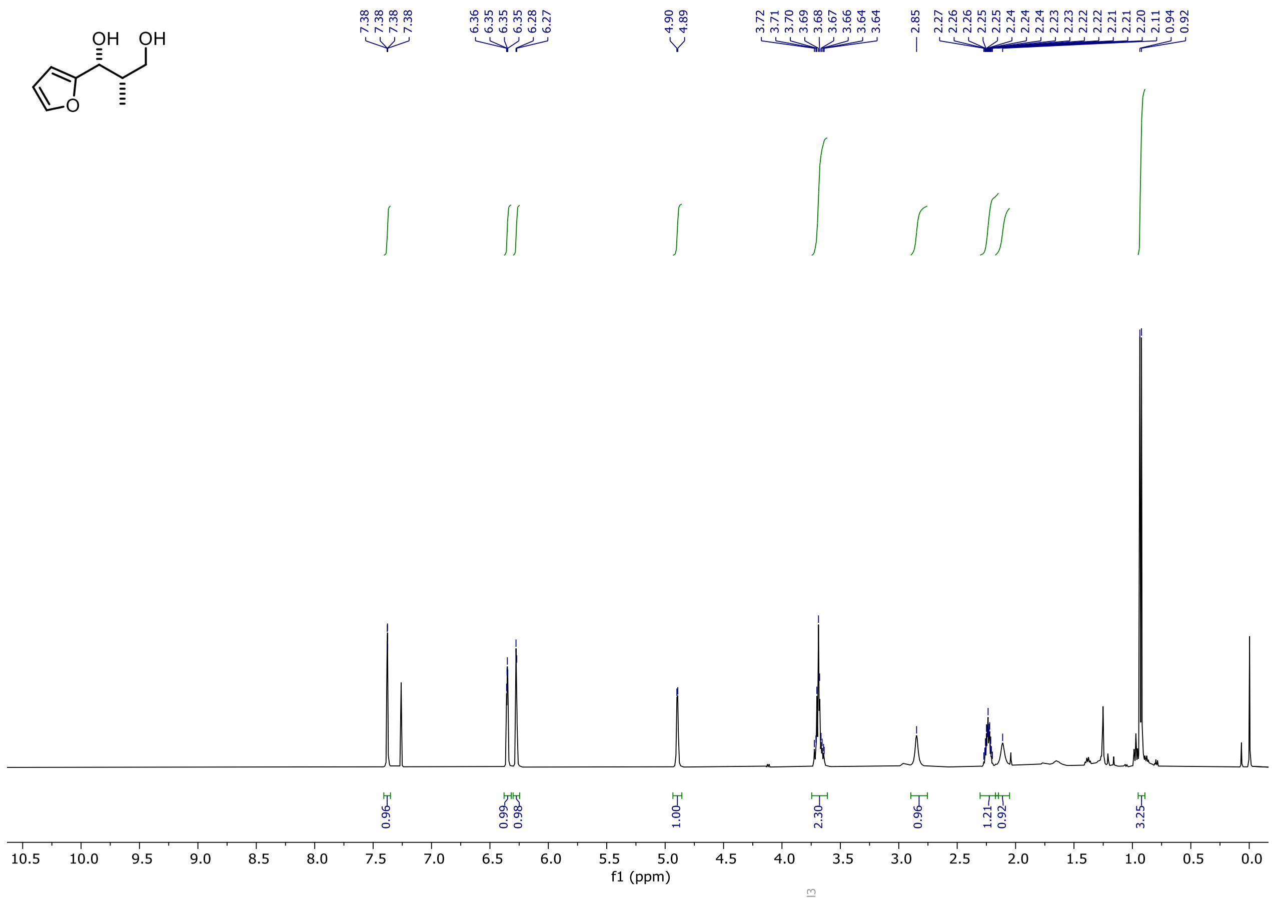

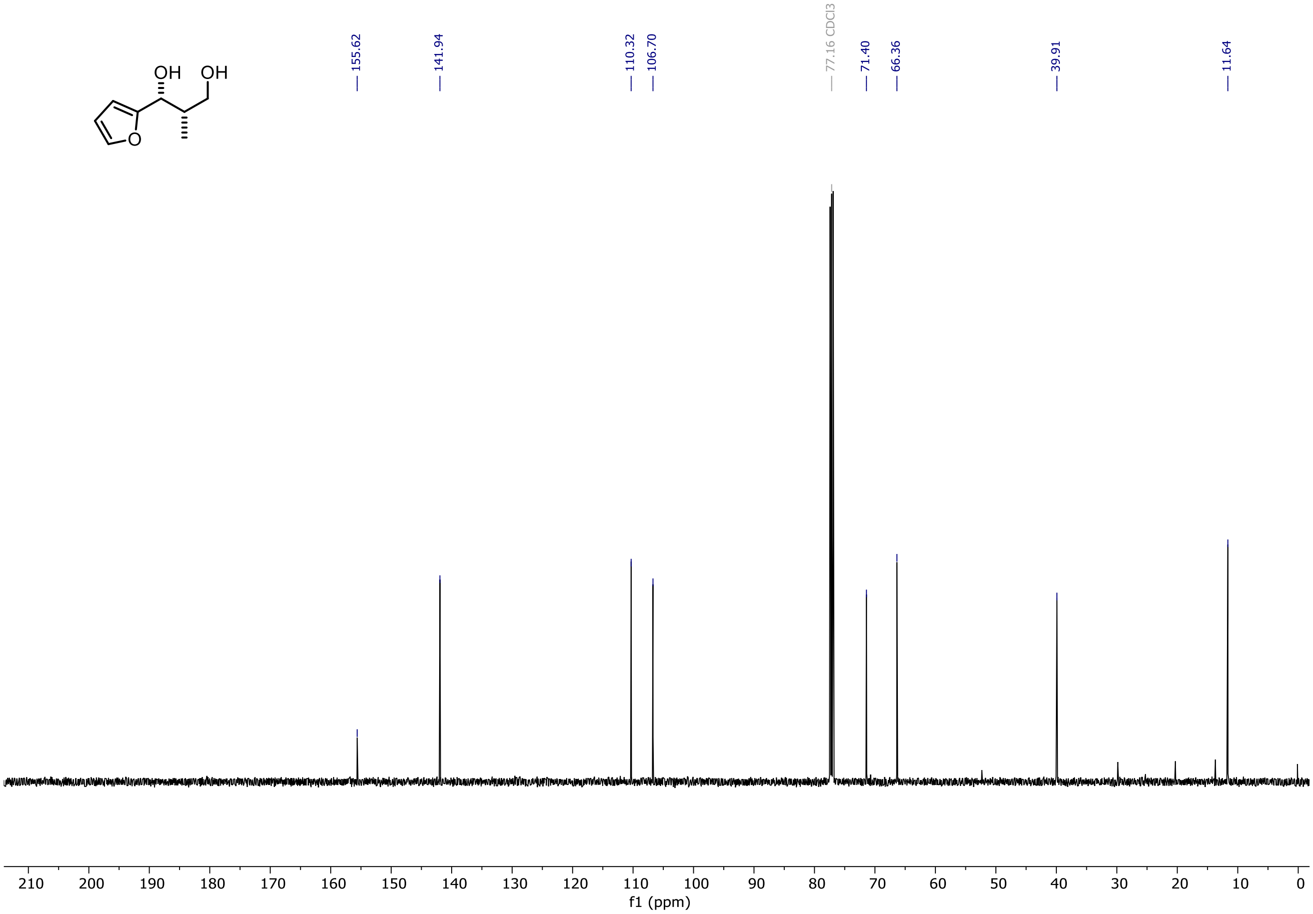

(2*R*,3*S*,4*S*,5*R*)-5-(Furan-2-yl)-3-hydroxy-2,4-dimethyl-1-morpholino-5-((triethylsilyl)oxy)pentan-1-one

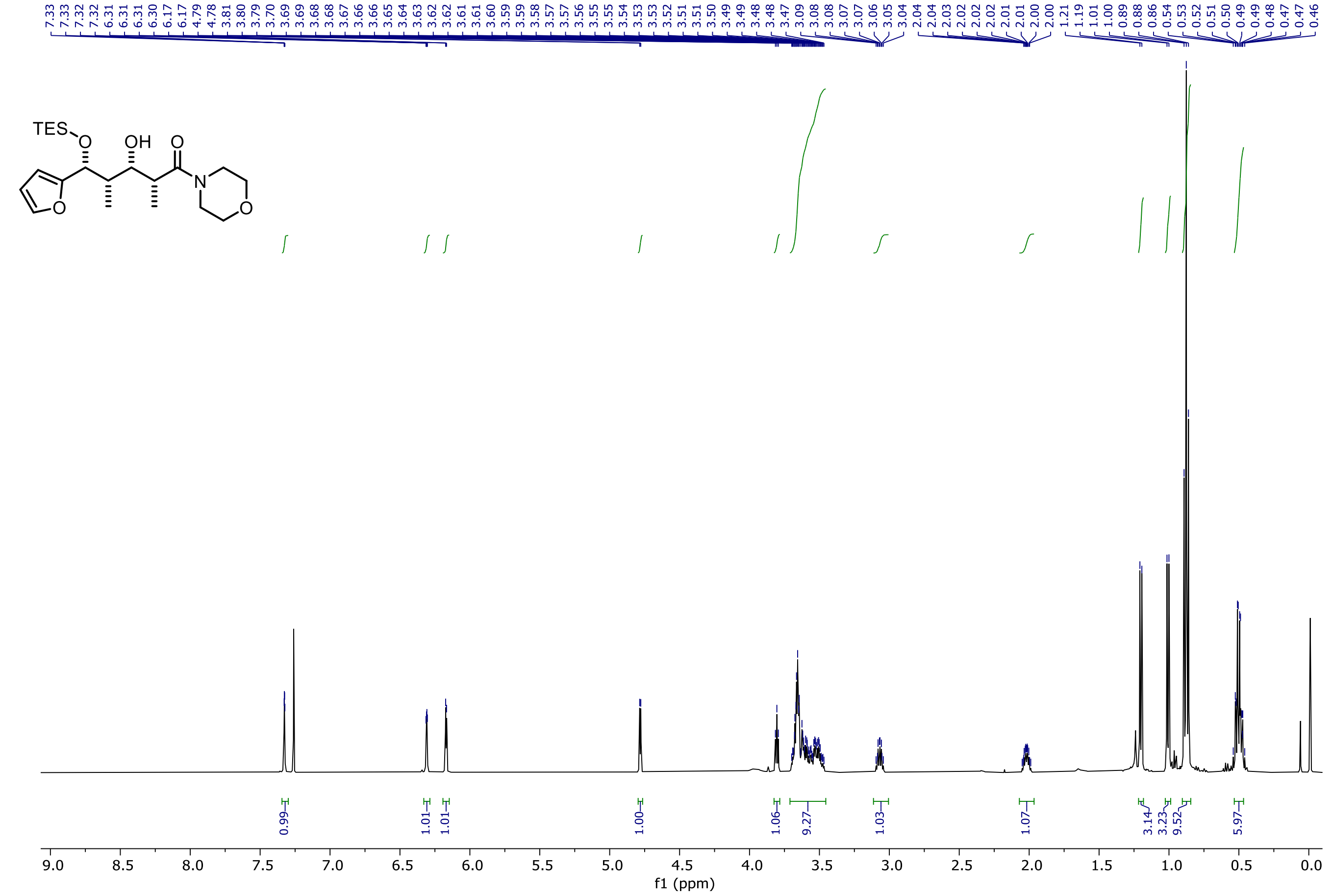

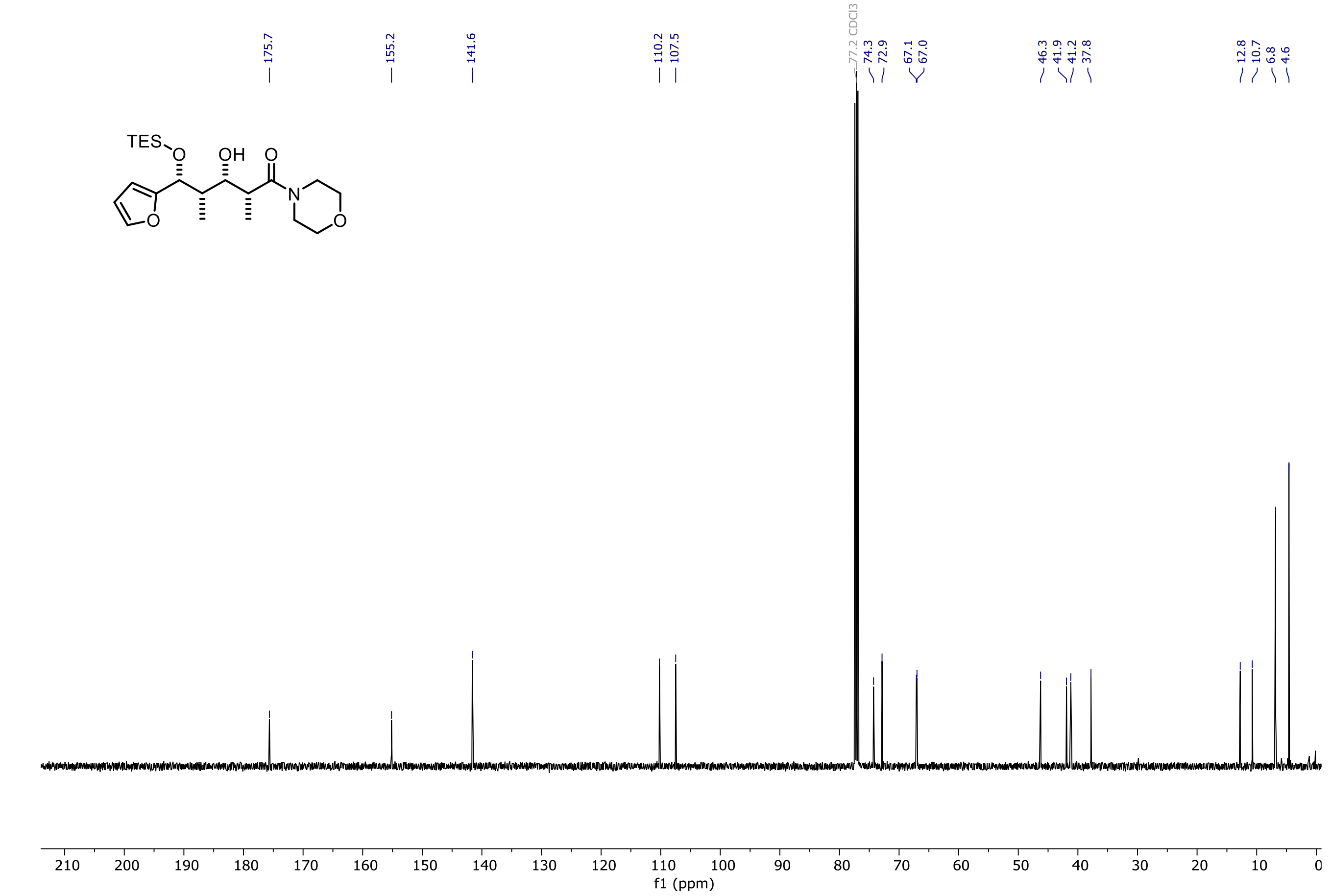

(2*S*,3*R*,4*S*,5*R*)-5-(Furan-2-yl)-3-hydroxy-2,4-dimethyl-1-morpholino-5-((triethylsilyl)oxy)pentan-1-one

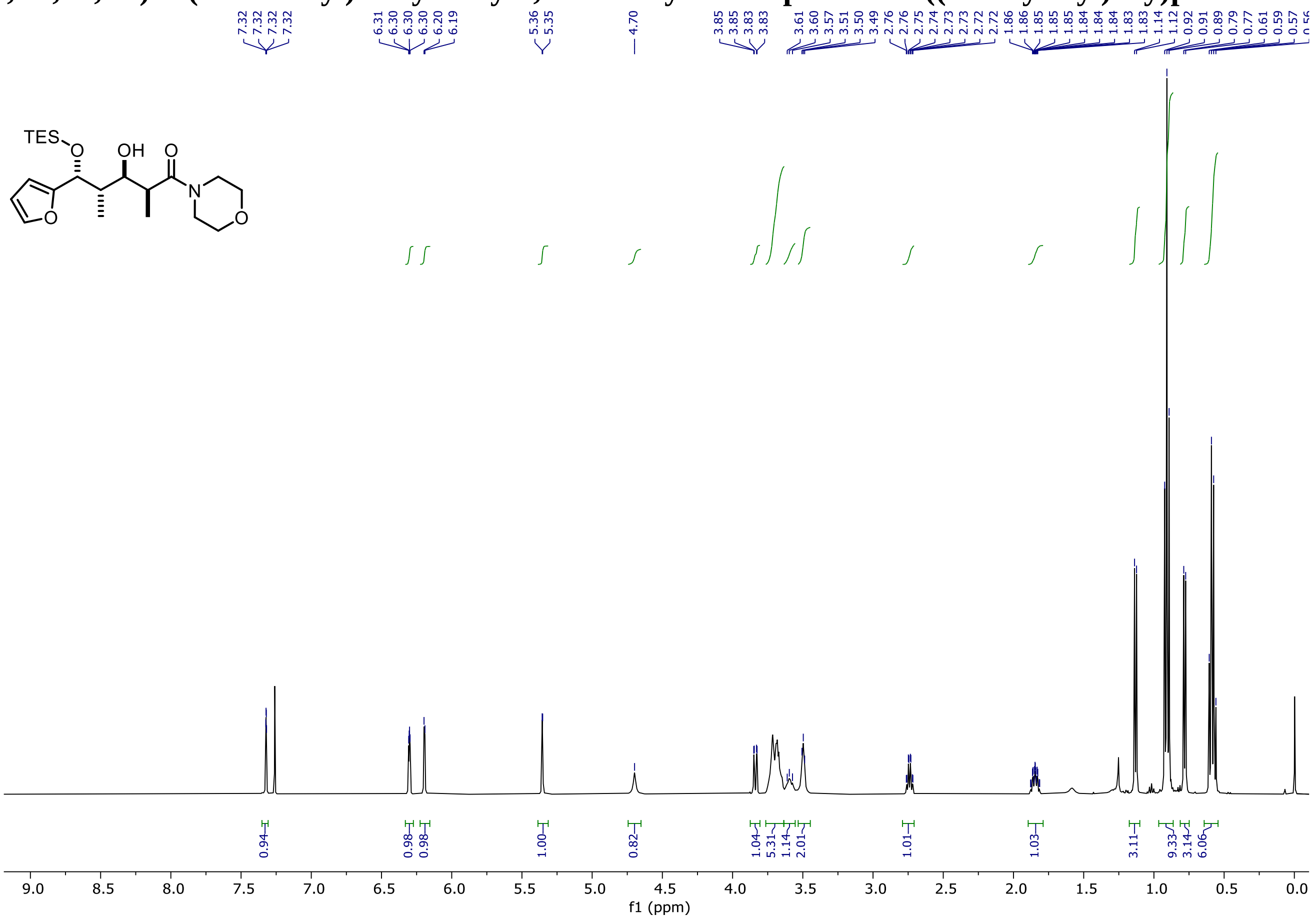

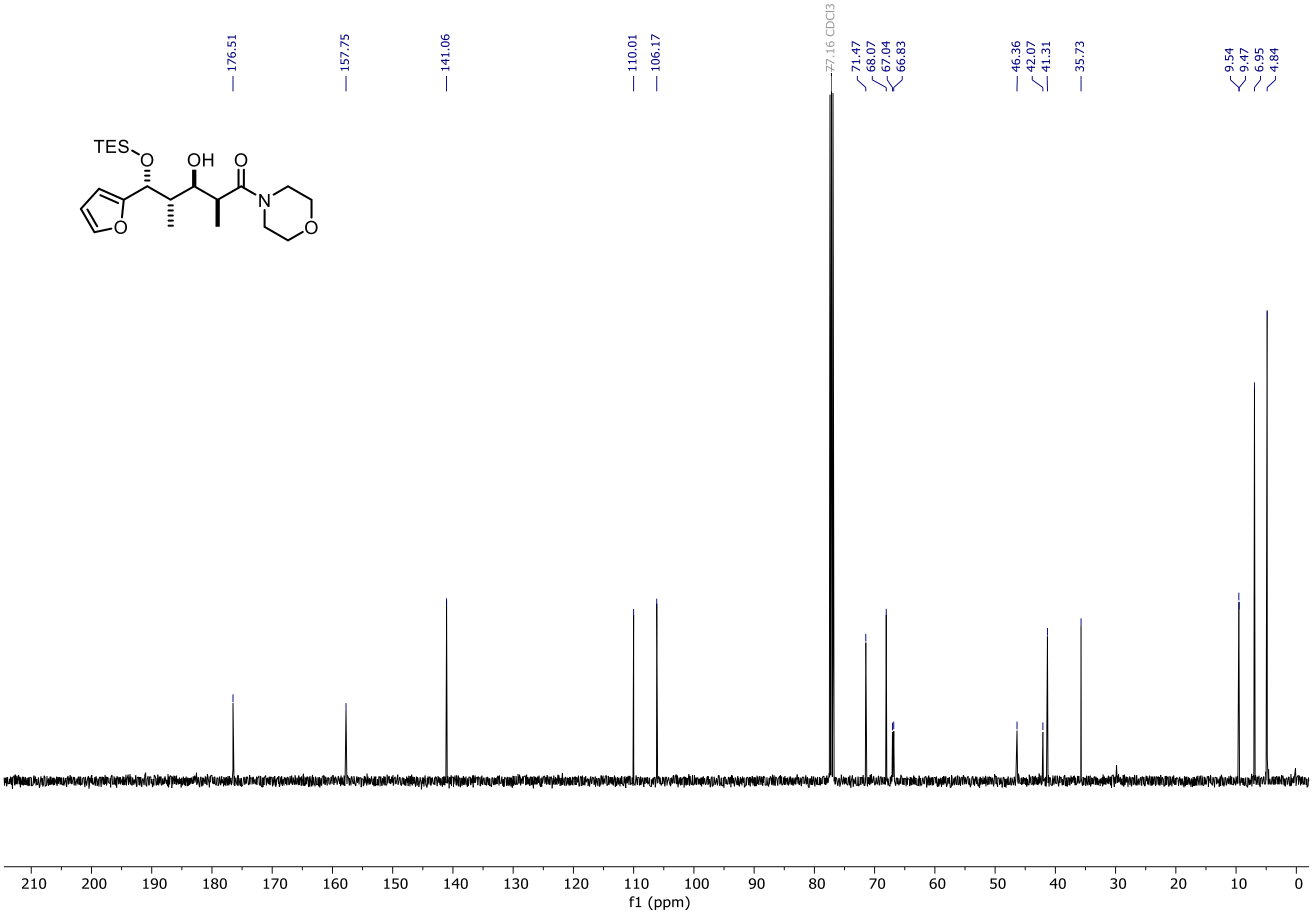

(2*S*,3*S*,4*S*,5*R*)-5-(Furan-2-yl)-3-hydroxy-2,4-dimethyl-5-((triethylsilyl)oxy)pentanal

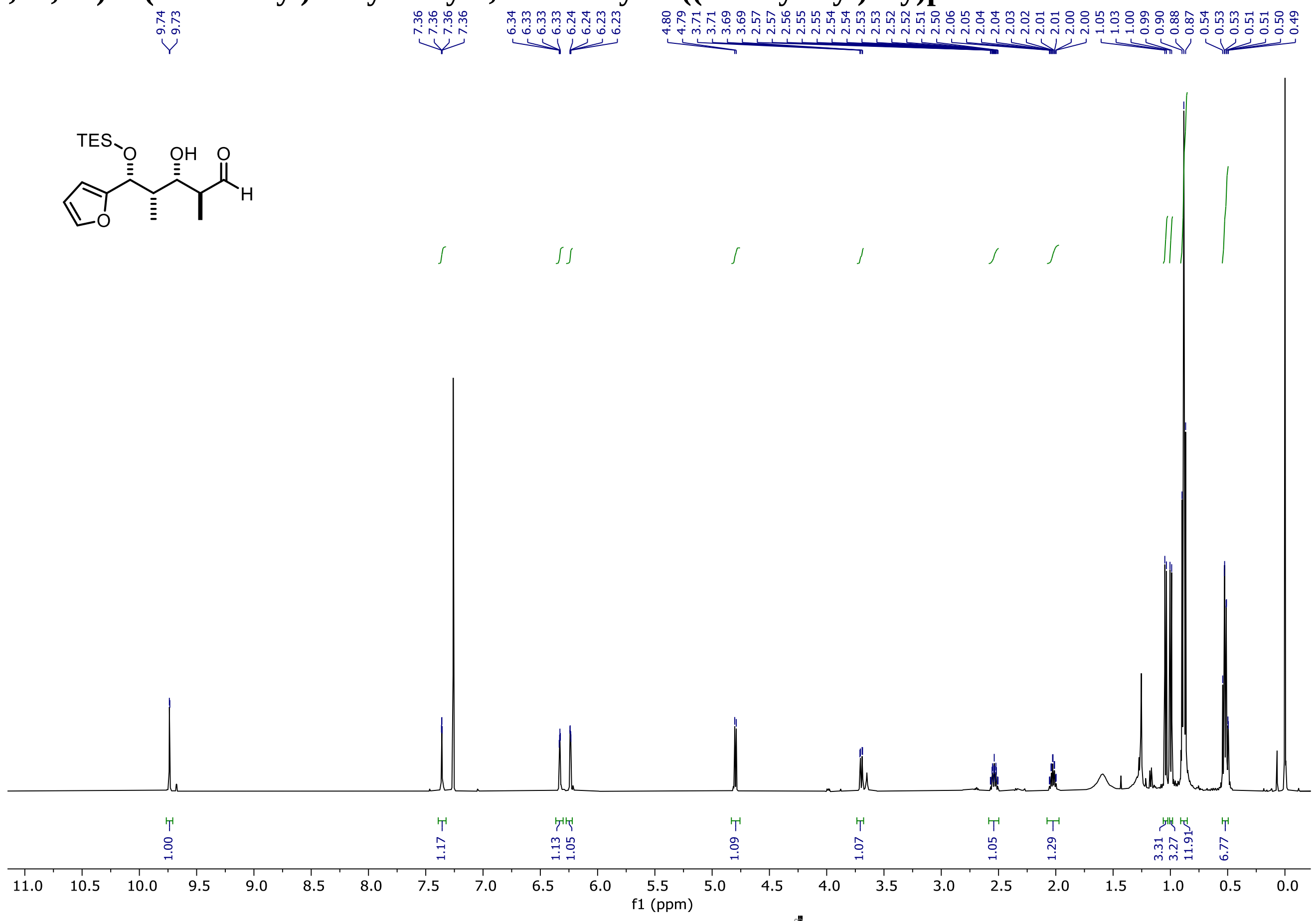

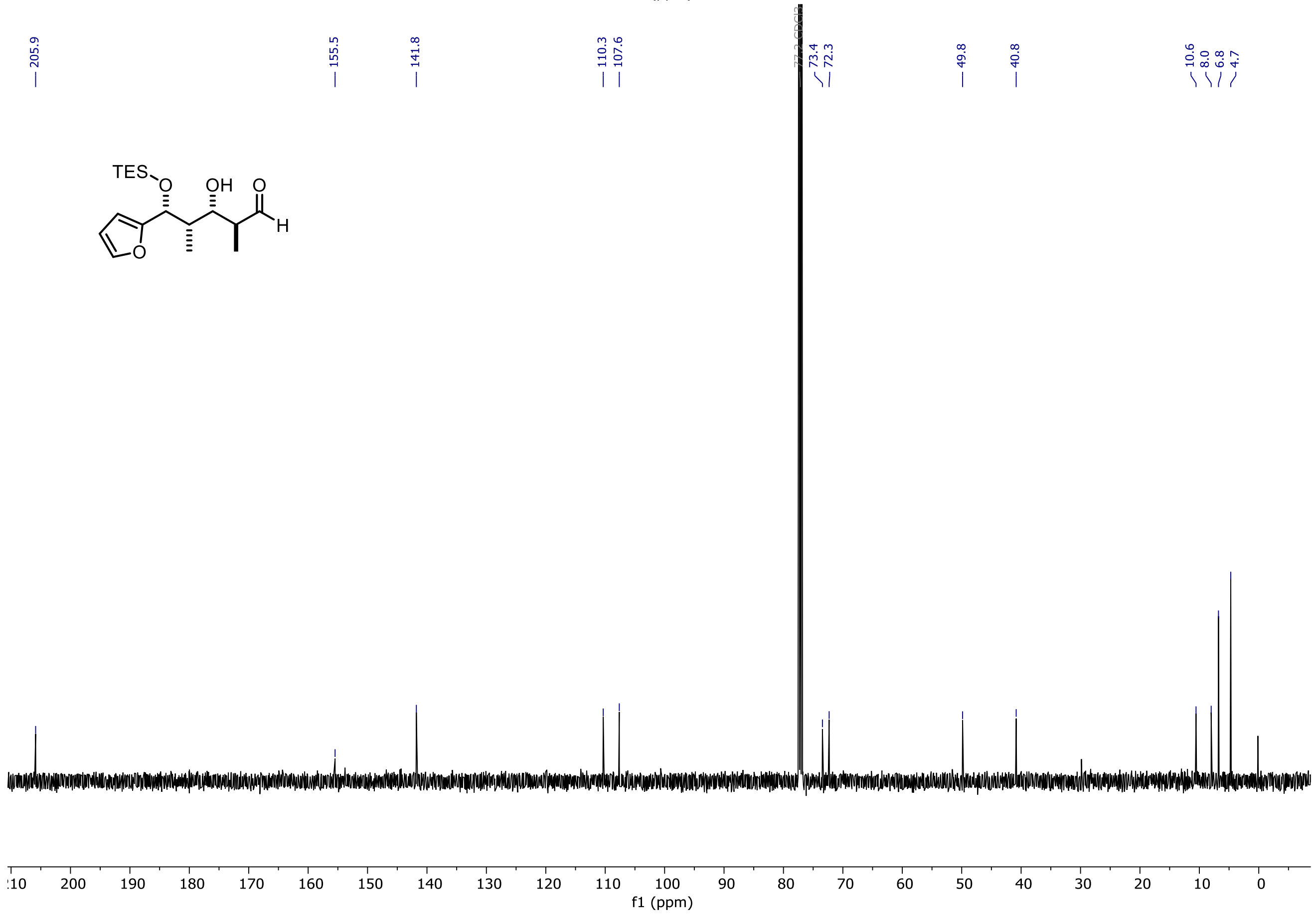

(2*R*, 3*R*)-2-Methyl-3-(thiophen-2-yl)-3-((triethylsilyl)oxy)propanal

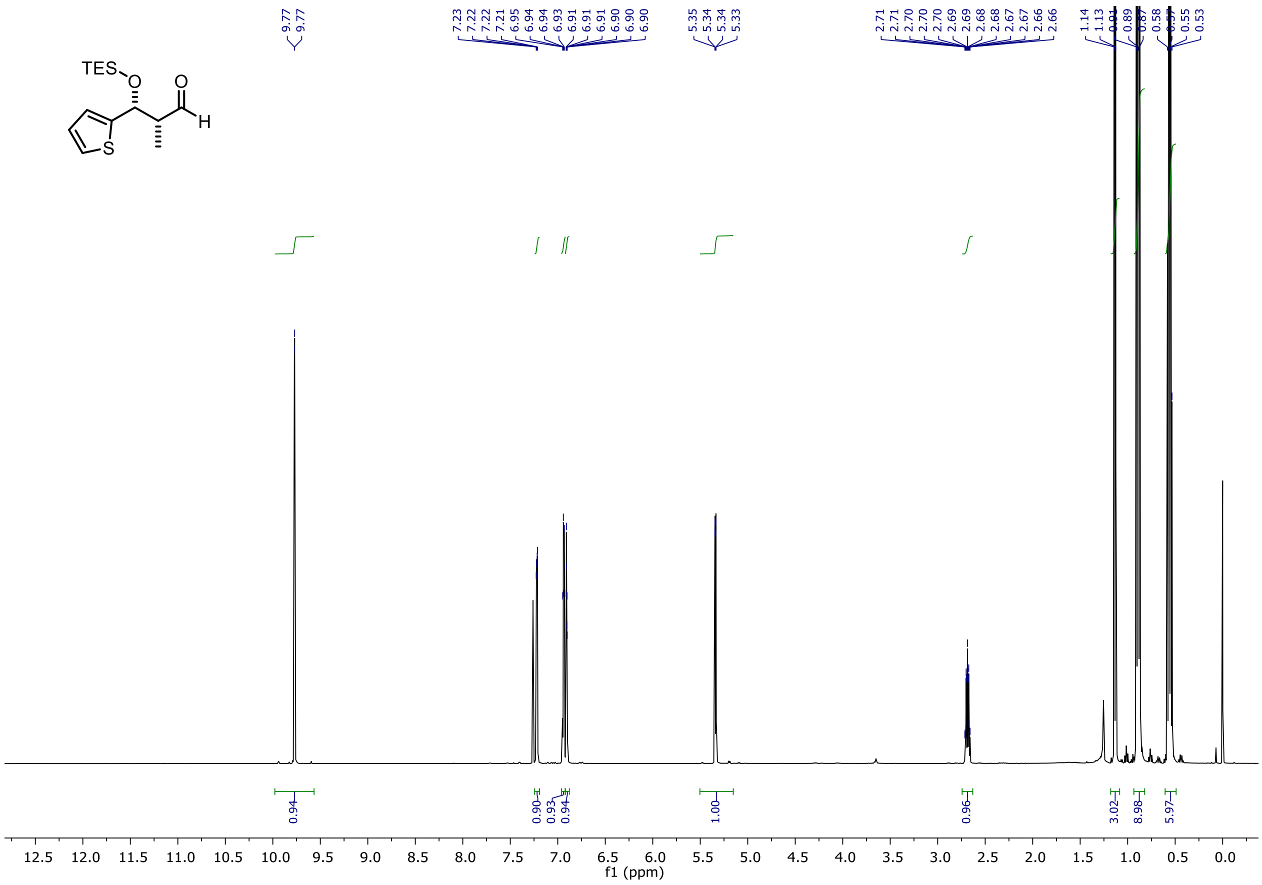

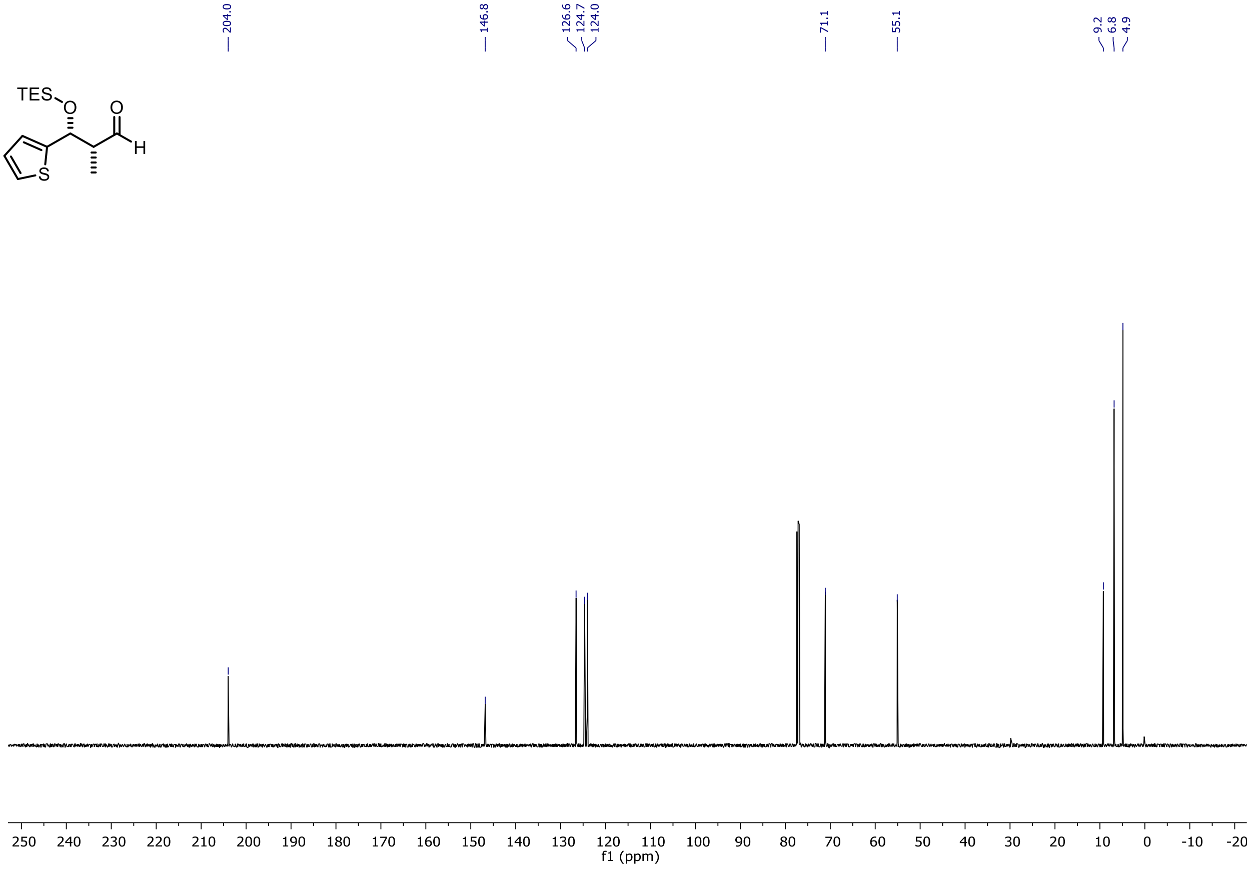

(1*R*, 2*S*)-2-Methyl-1-(thiophen-2-yl)propane-1,3-diol

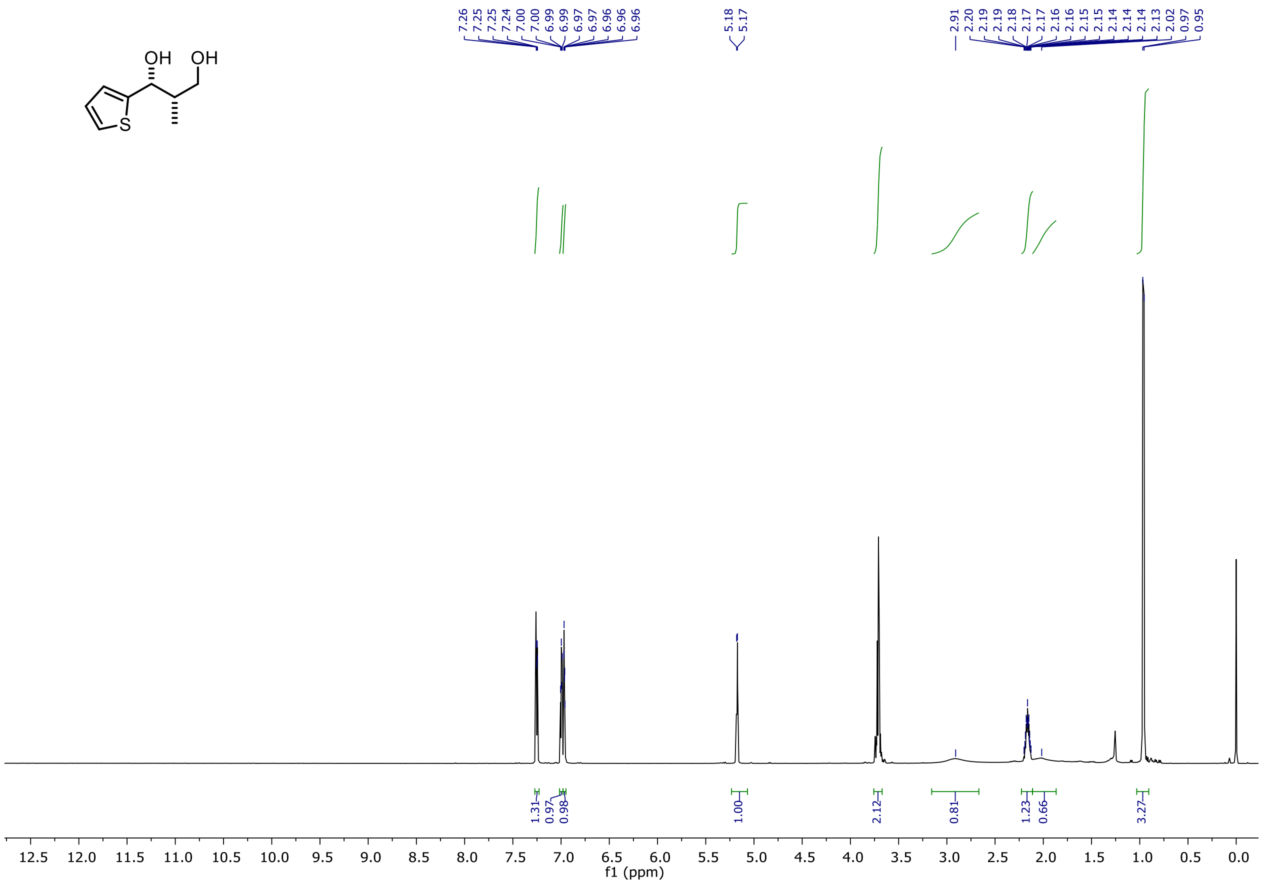

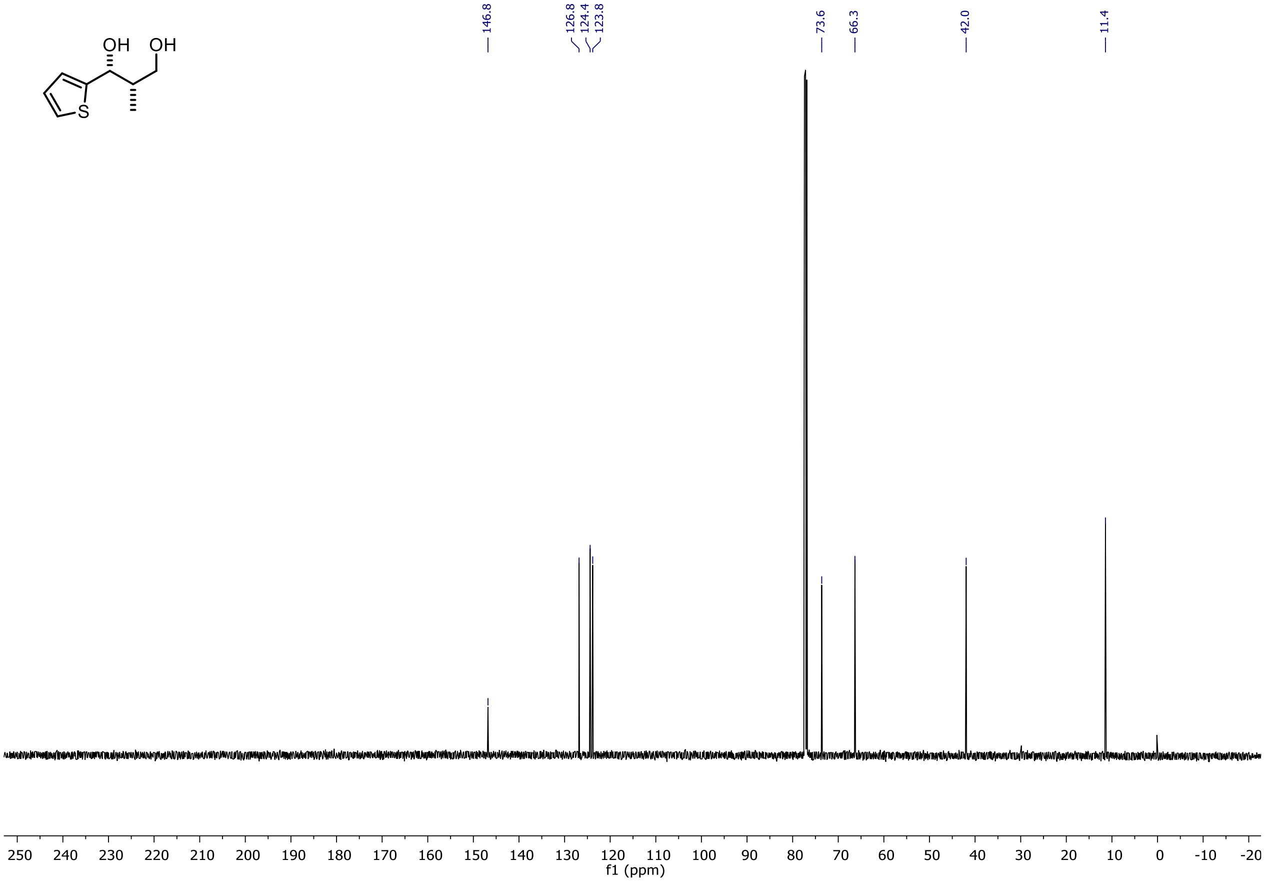

(2*R*,3*R*)-3-(4-Bromophenyl)-2-methyl-3-((triethylsilyl)oxy)propanal

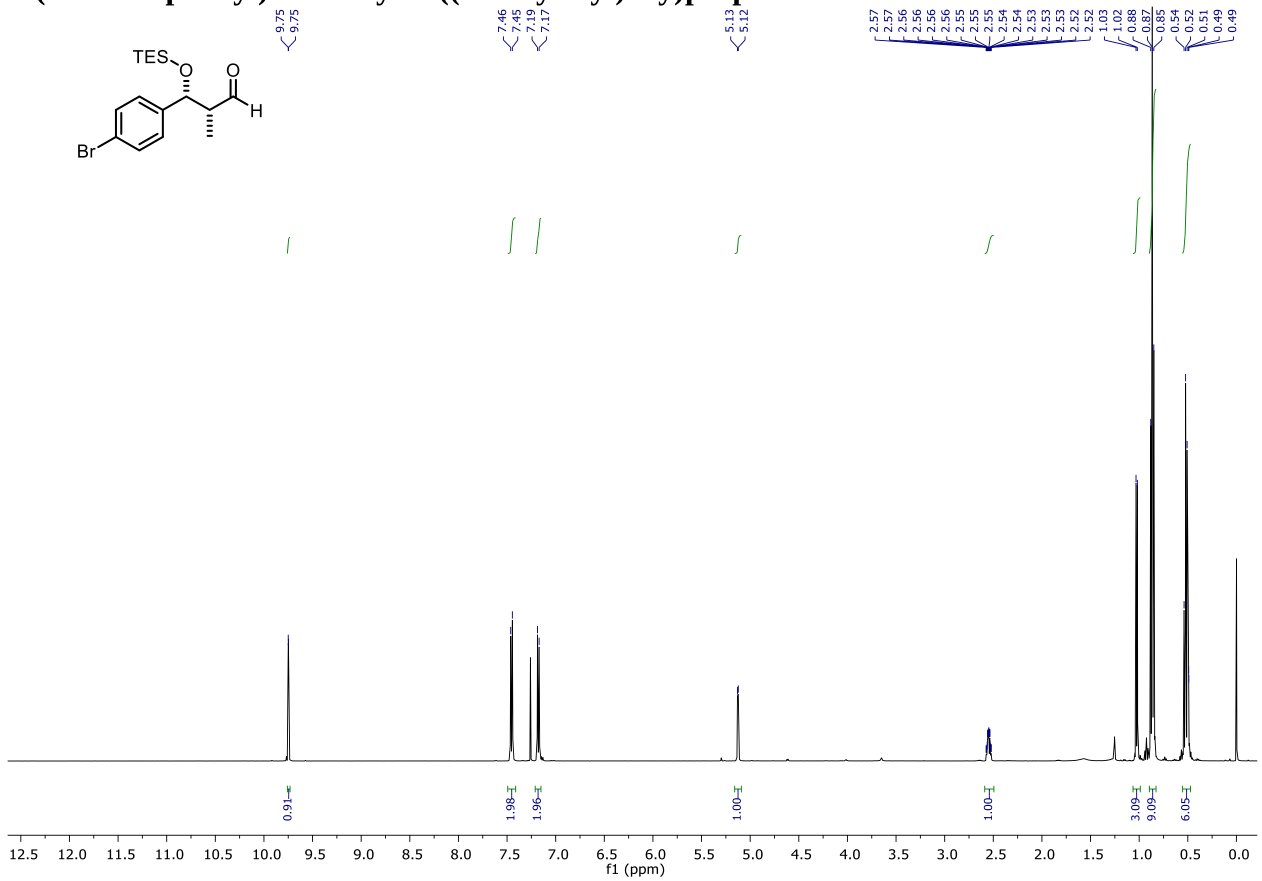

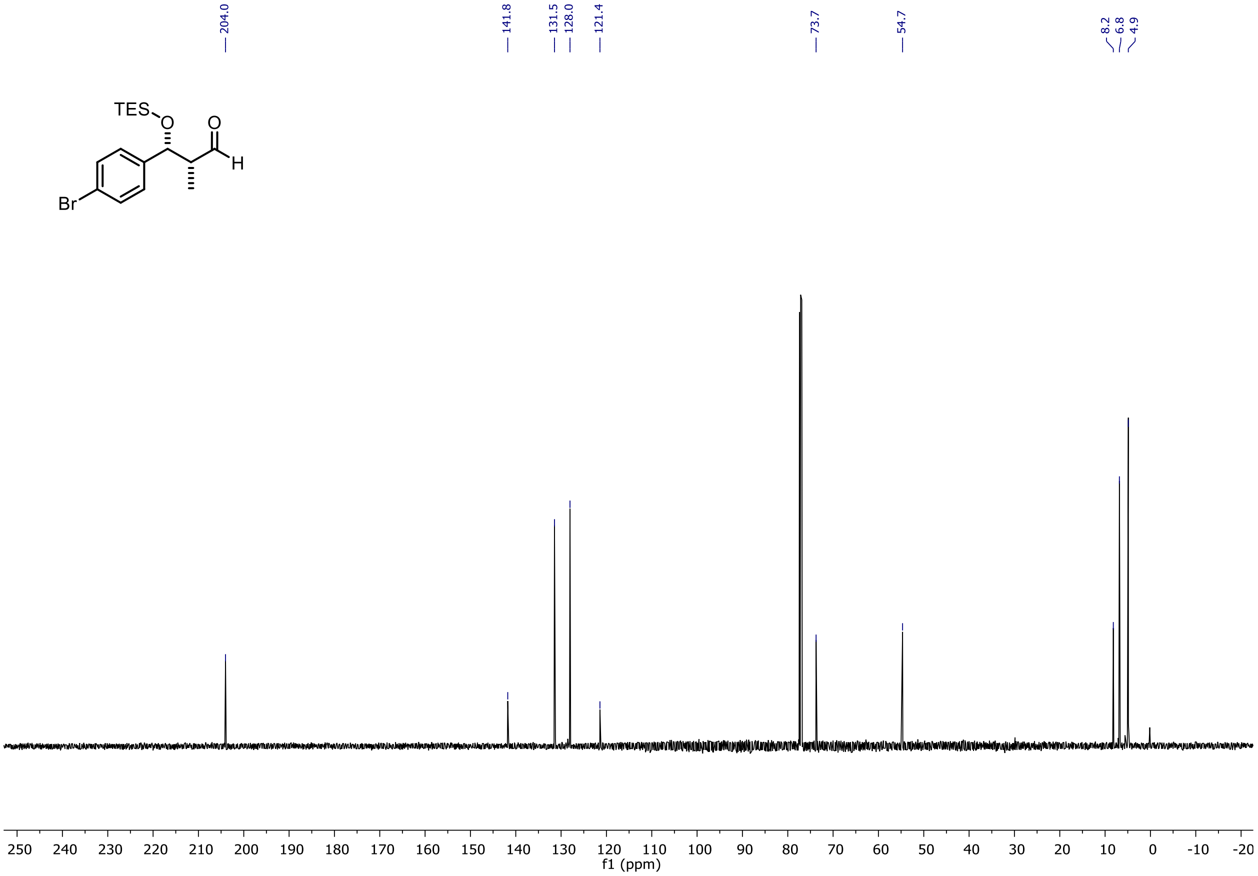

(1*R*,2*S*)-1-(4-Bromophenyl)-2-methylpropane-1,3-diol

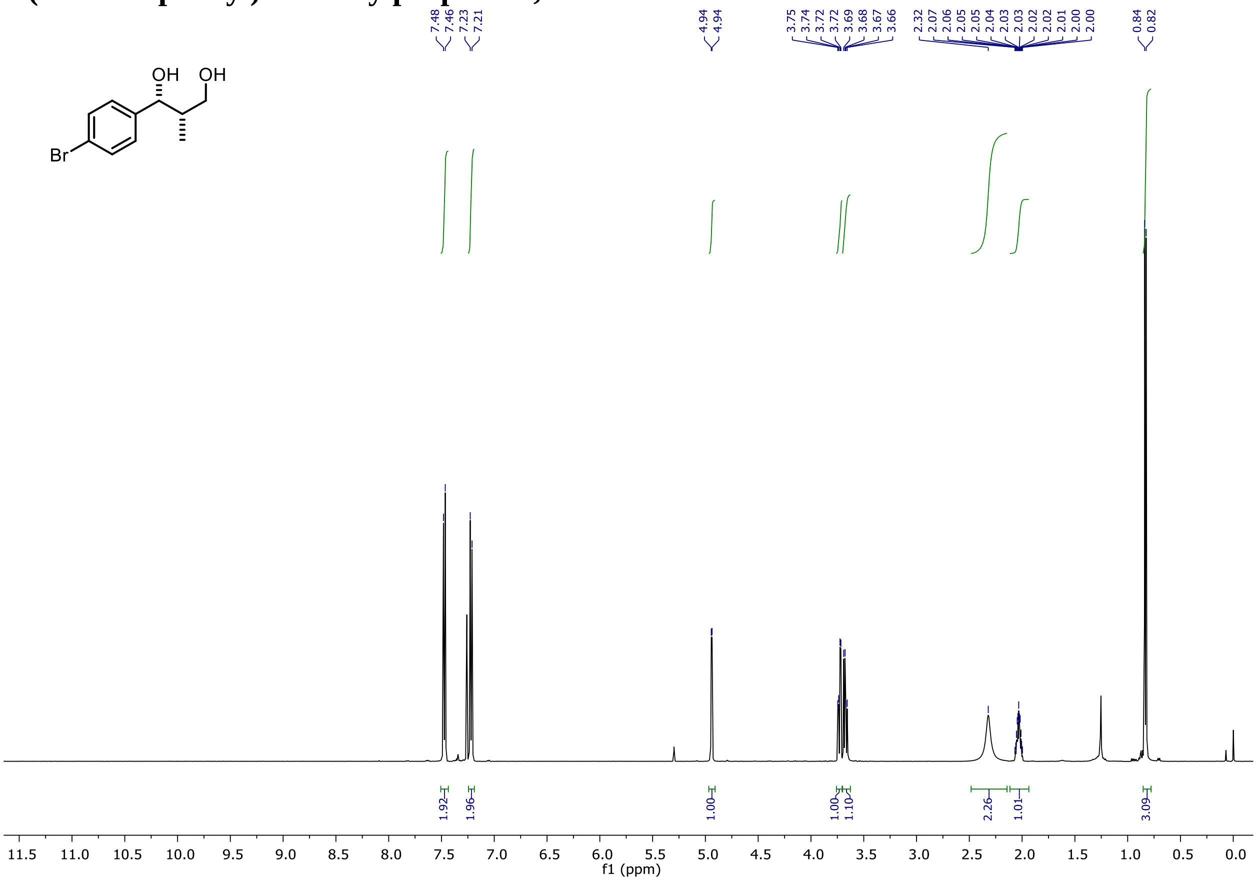

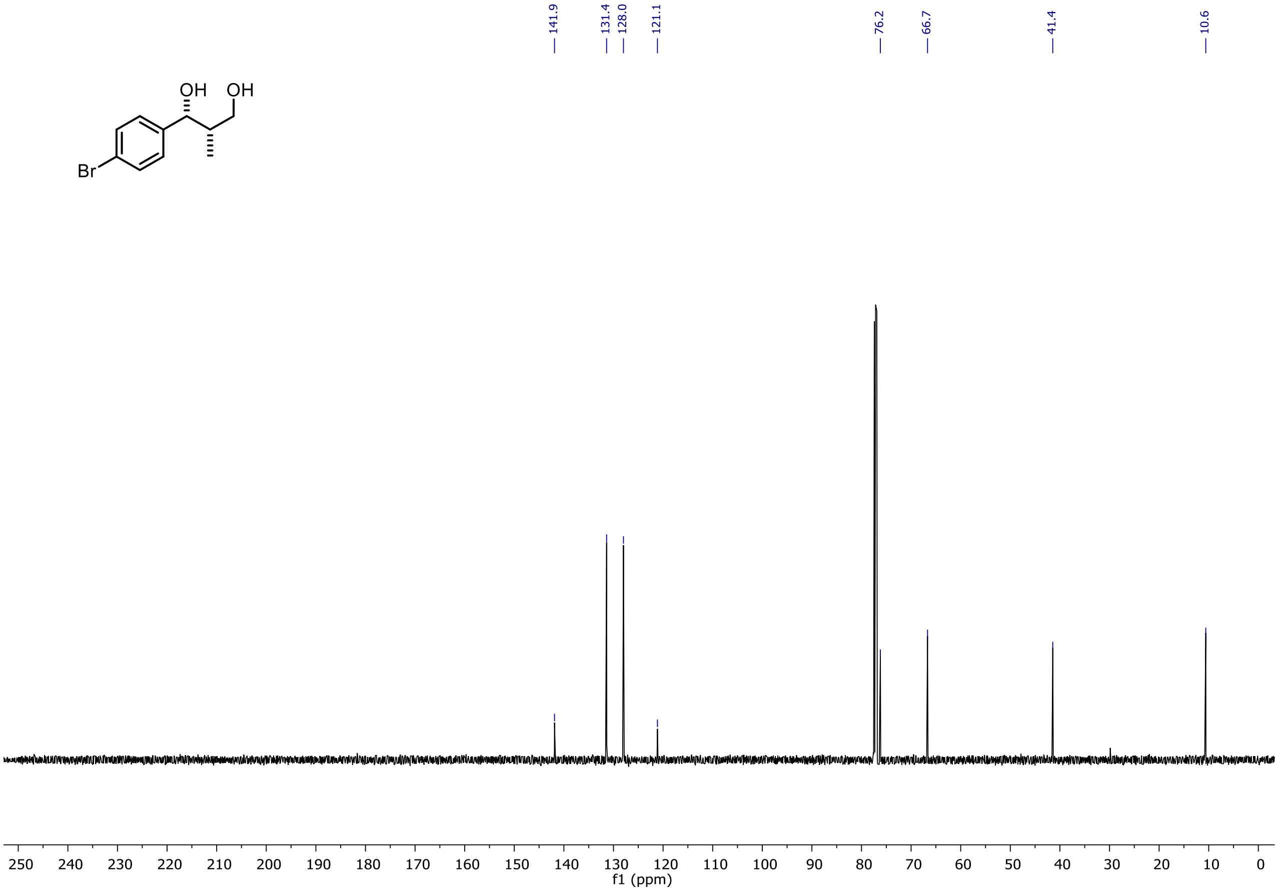

(2*R*,3*R*)-3-(4-Bromo-3,5-dimethoxyphenyl)-2-methyl-3-((triethylsilyl)oxy)propanal

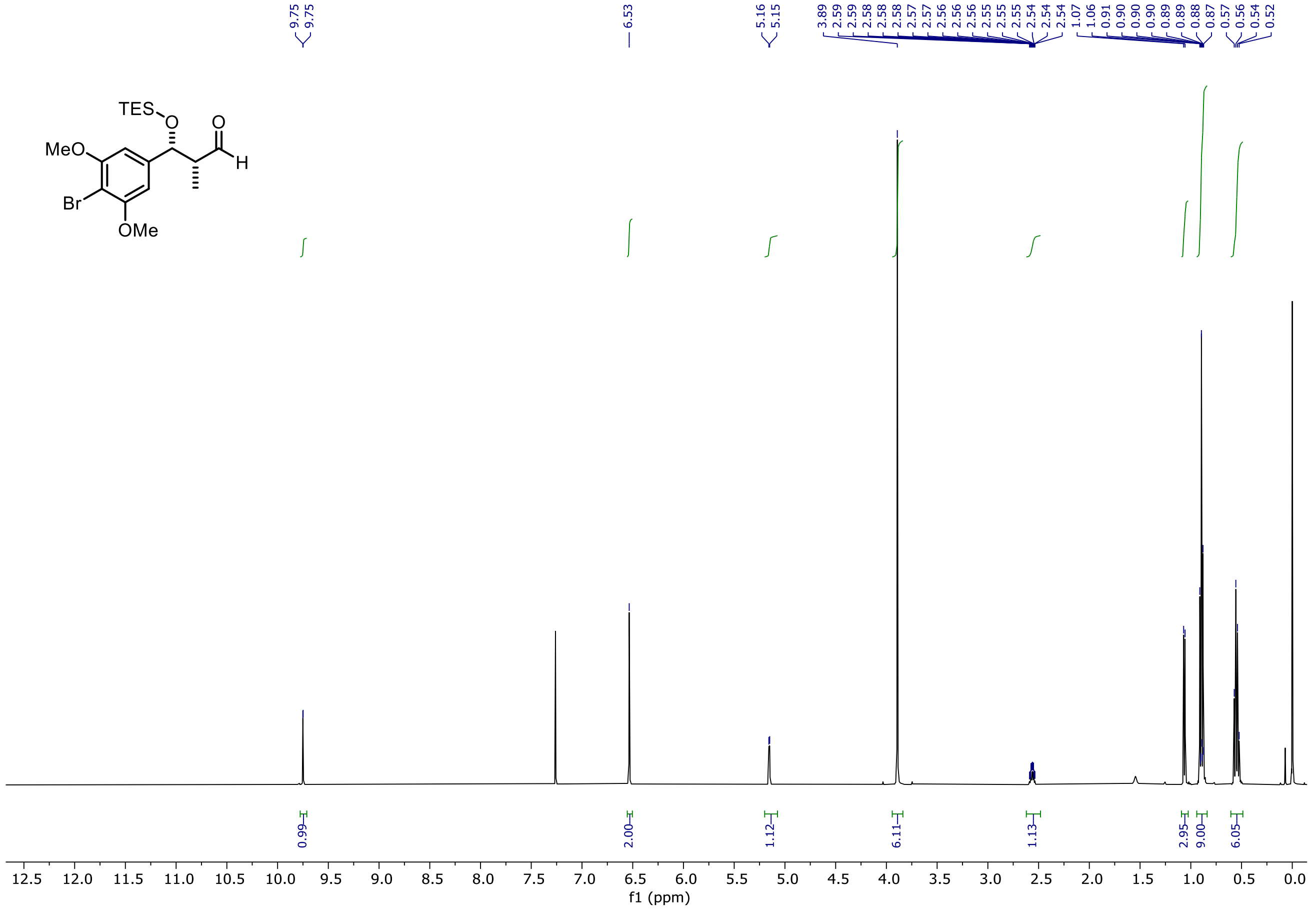

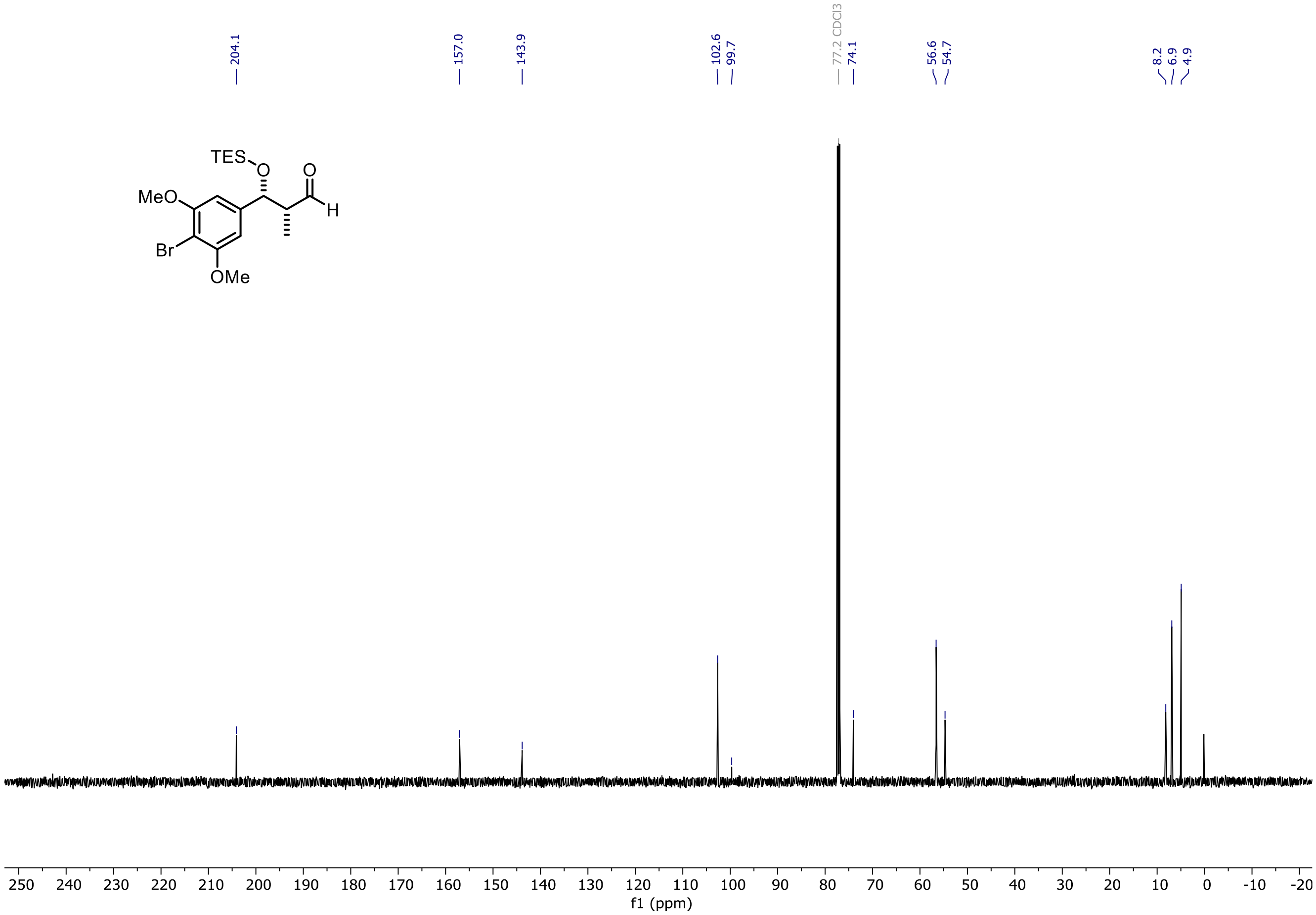

(1*R*,2*S*)-1-(4-Bromo-3,5-dimethoxyphenyl)-2-methylpropane-1,3-diol

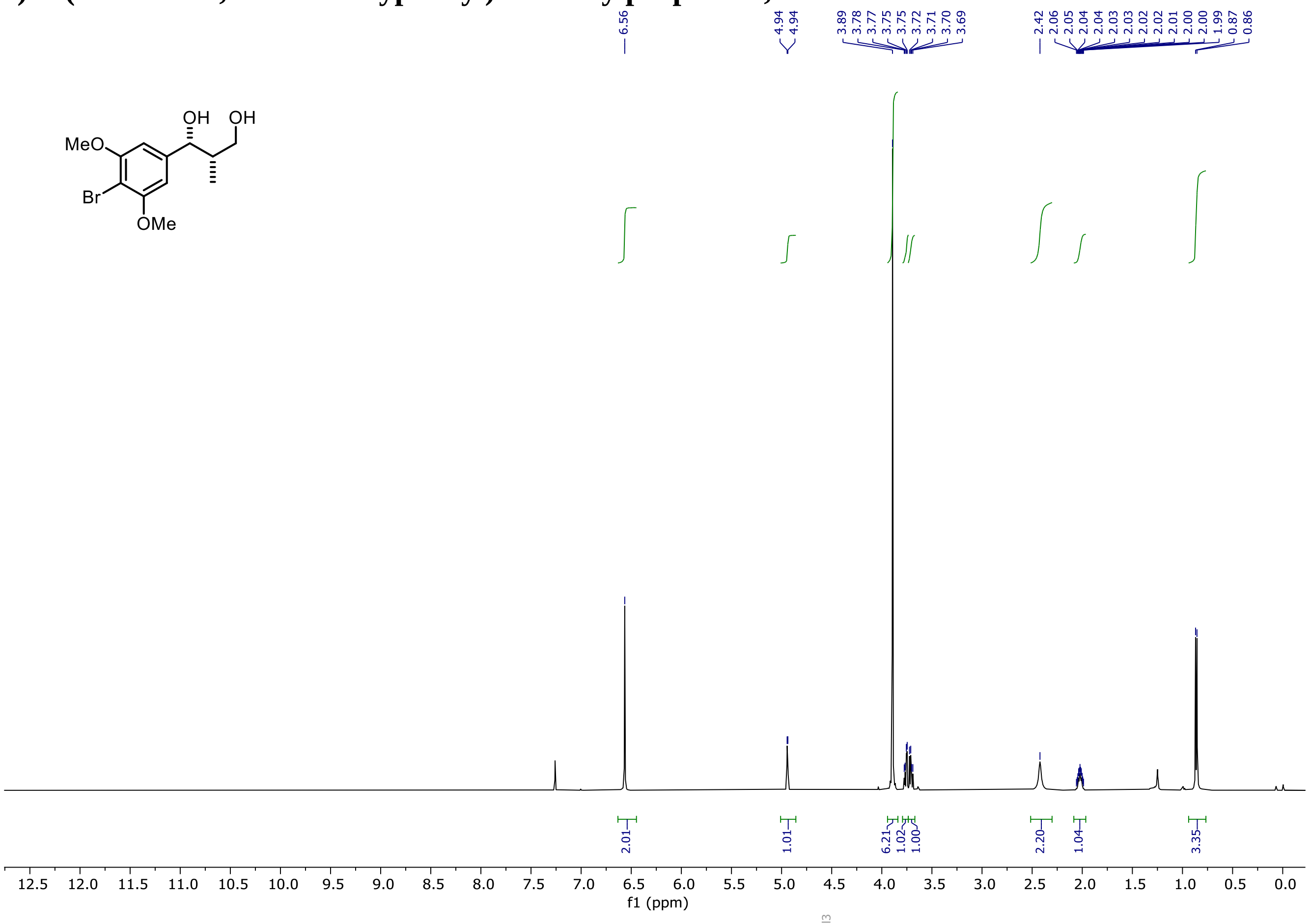

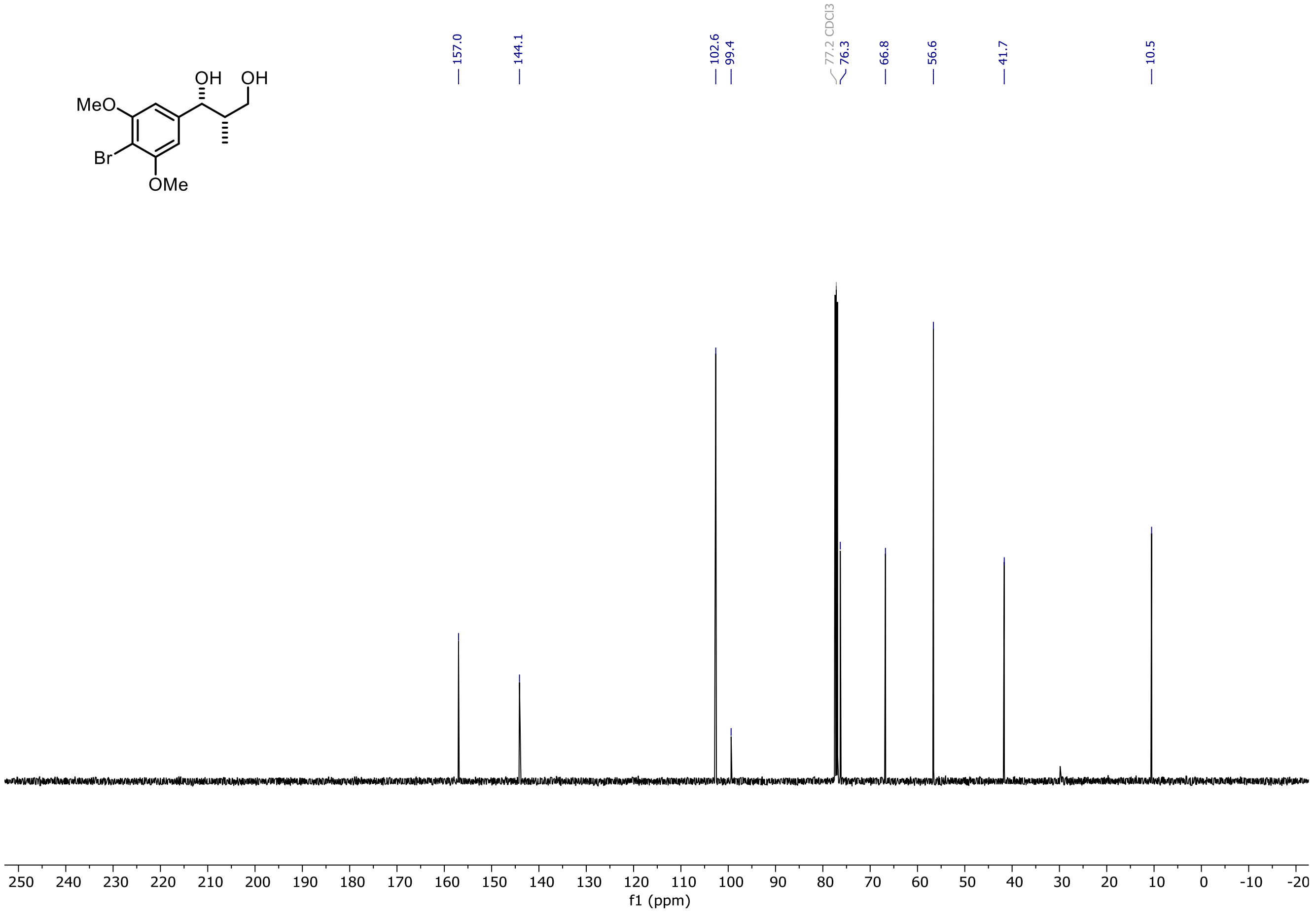

(2*R*,3*R*)-3-(2,5-Dimethoxy-3-nitrophenyl)-2-methyl-3-((triethylsilyl)oxy)propanal

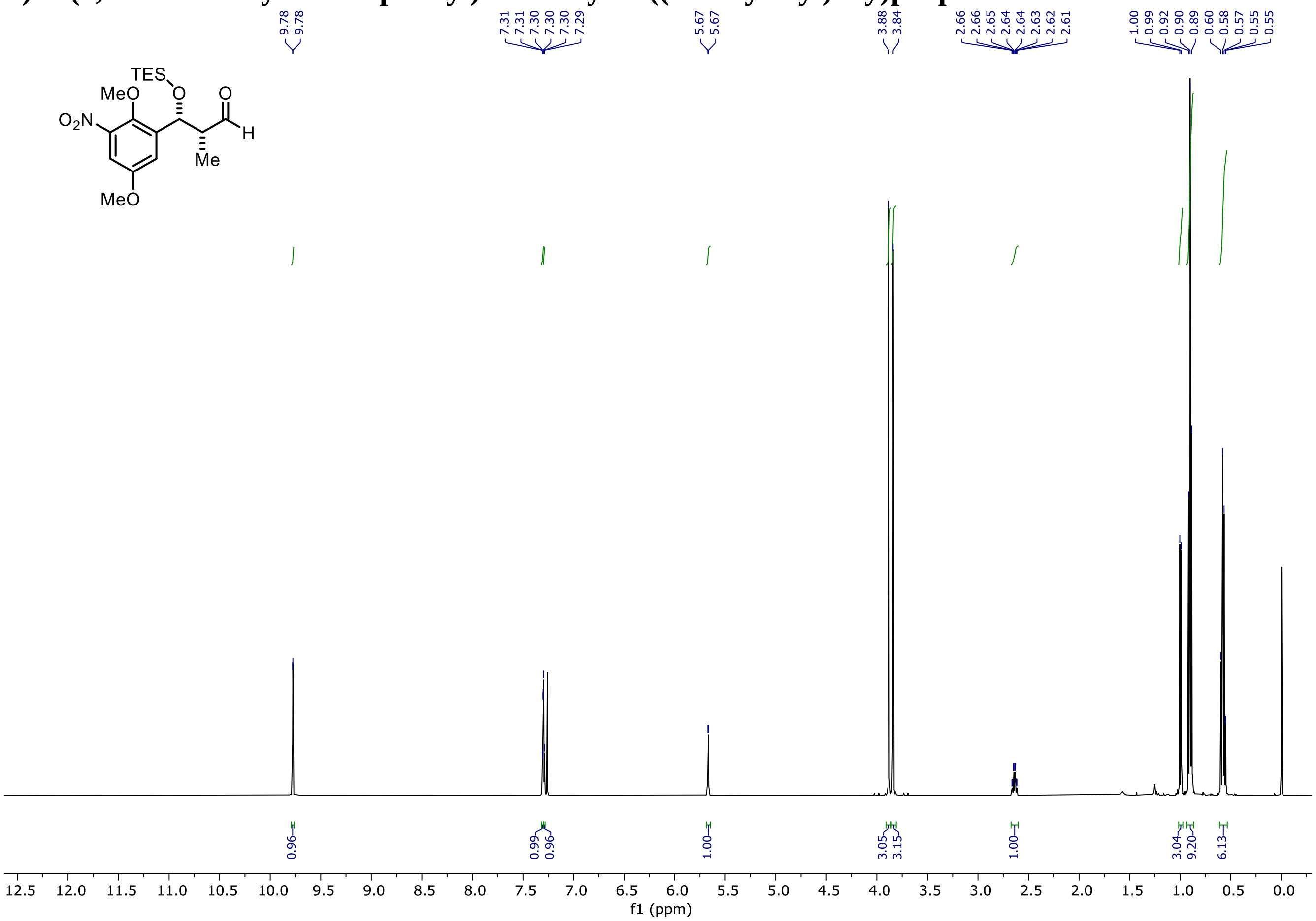

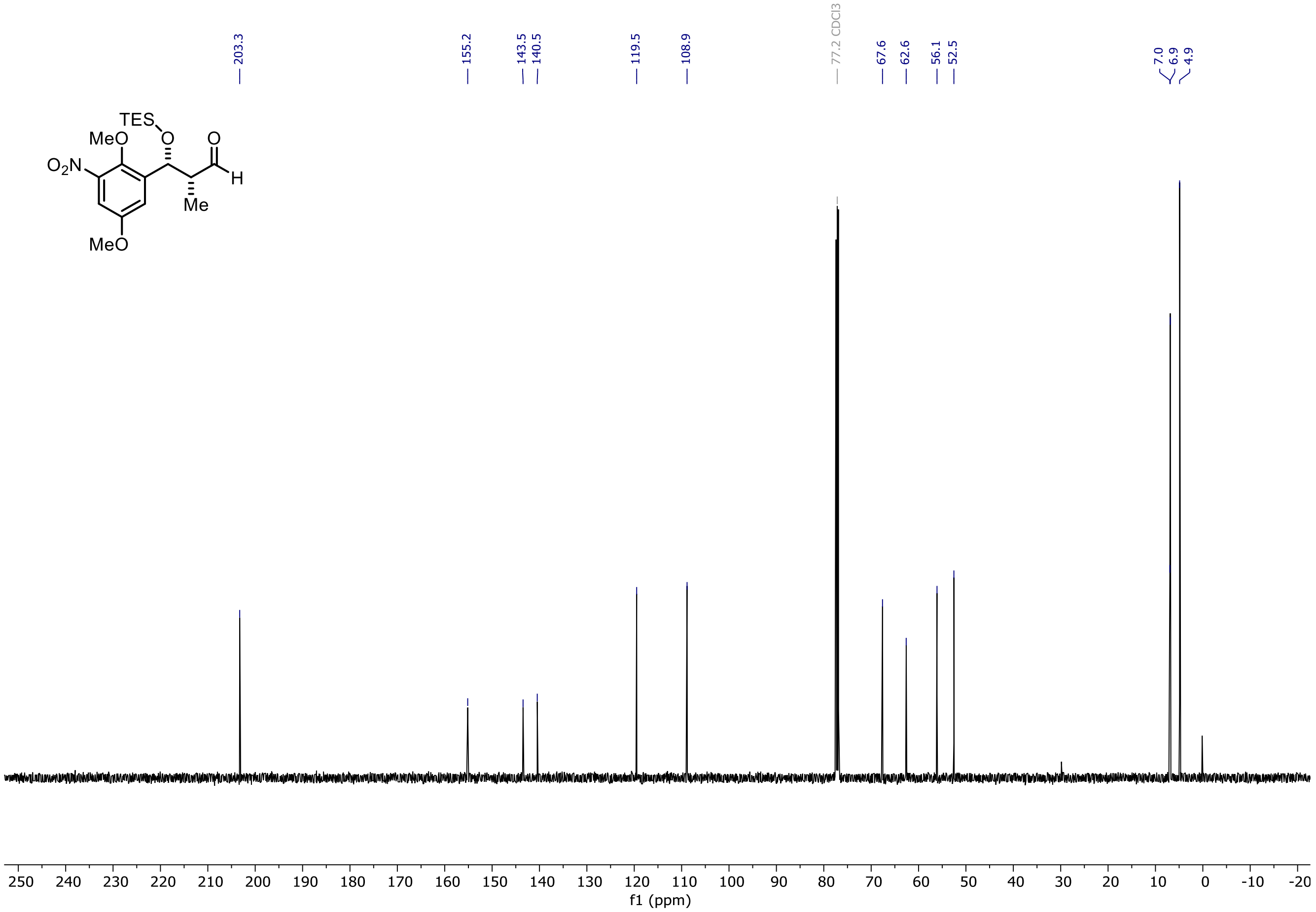

(1*R*,2*S*)-1-(2,5-Dimethoxy-3-nitrophenyl)-2-methylpropane-1,3-diol

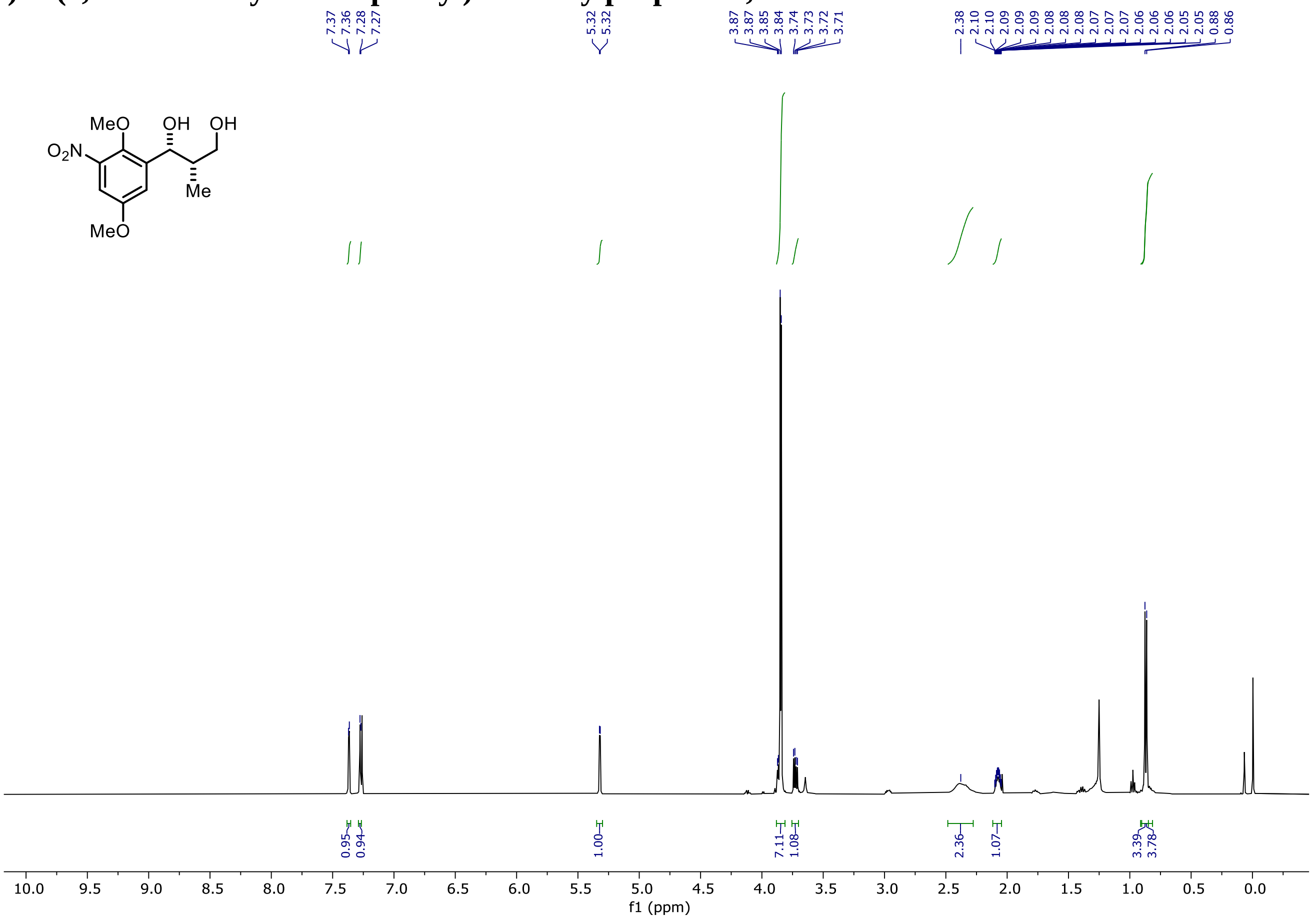

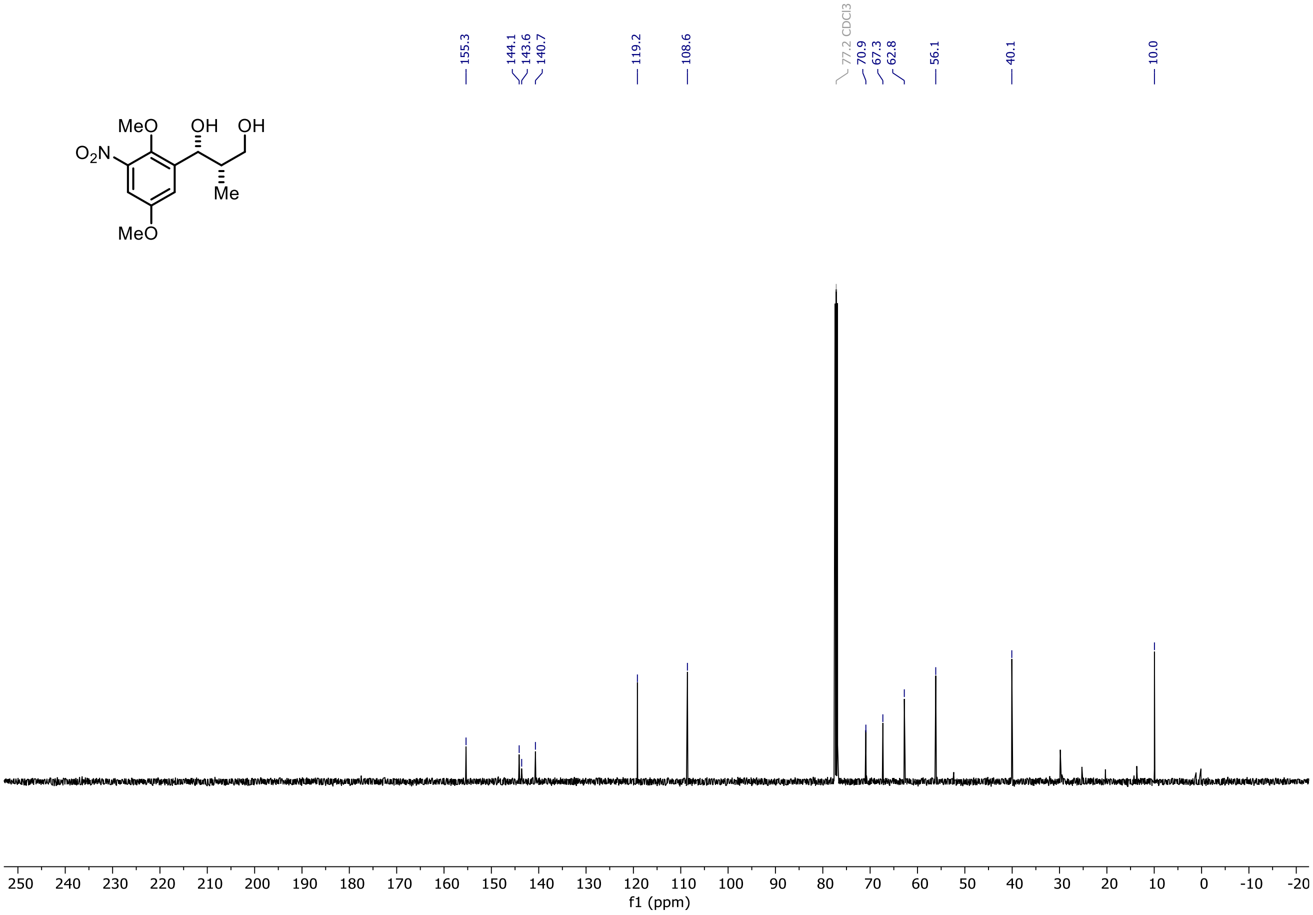

3-((1*R*,2*R*)-2-Methyl-3-oxo-1-((triethylsilyl)oxy)propyl)benzaldehyde

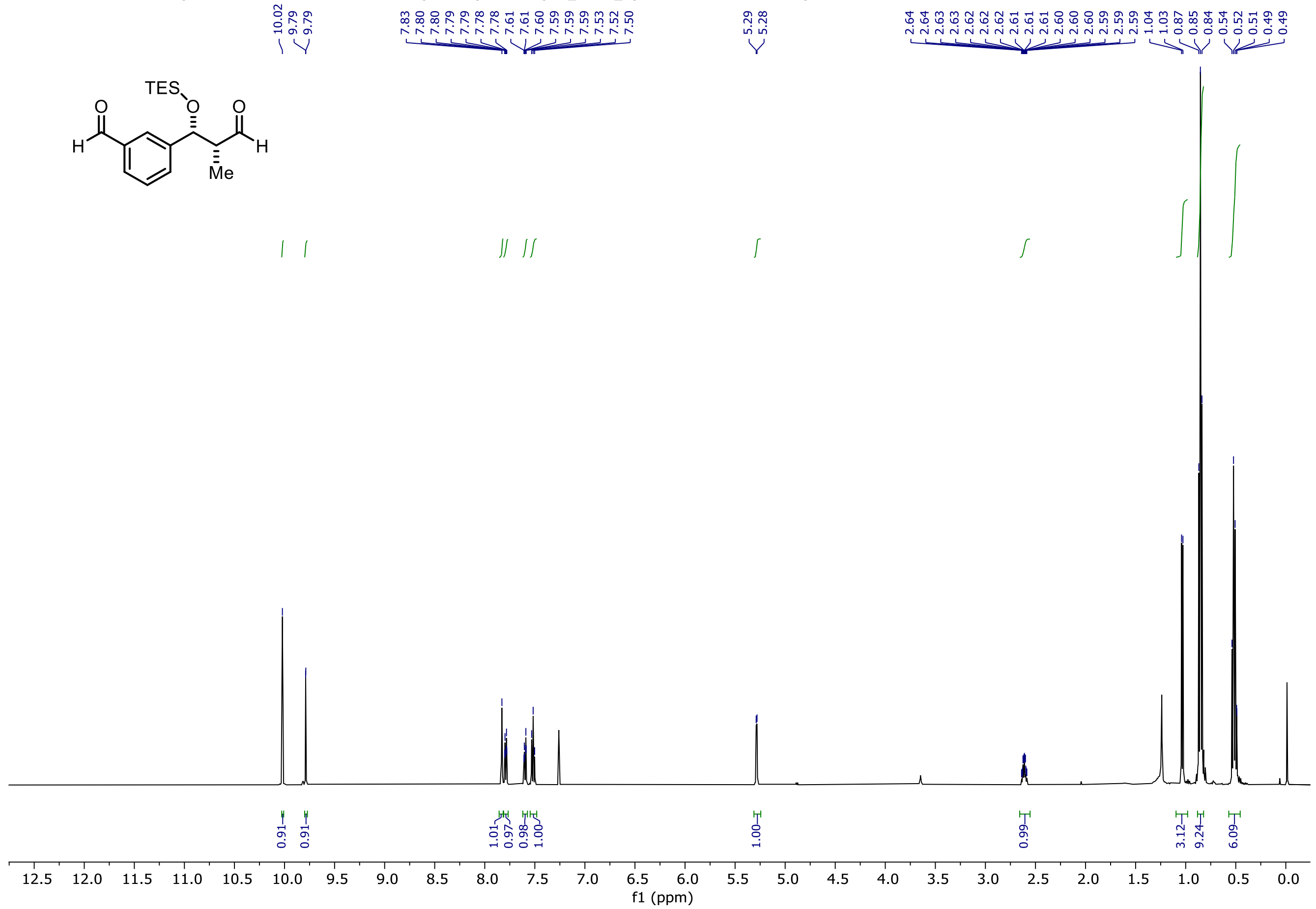

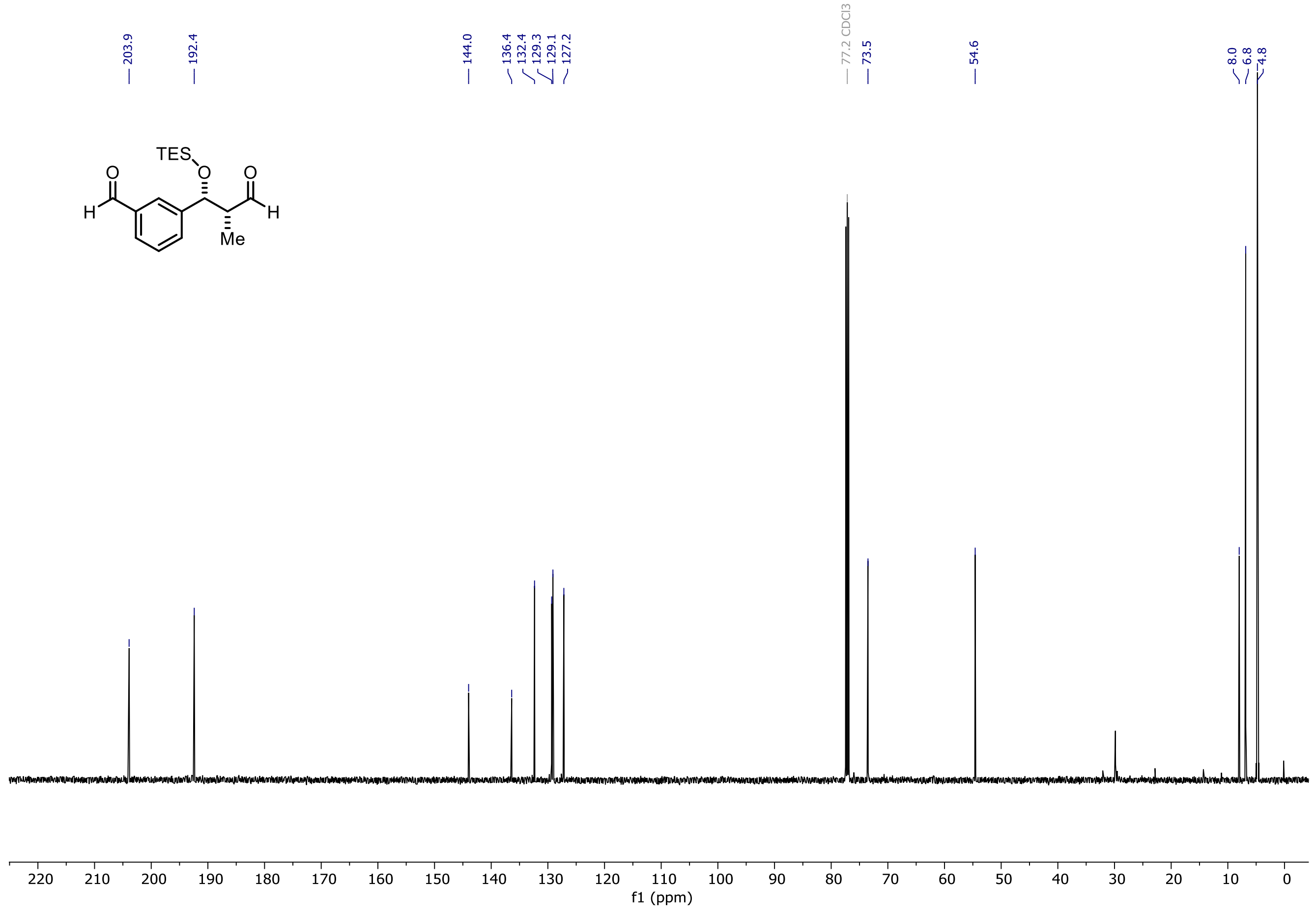

(1*R*,2*S*)-1-(3-(Hydroxymethyl)phenyl)-2-methylpropane-1,3-diol

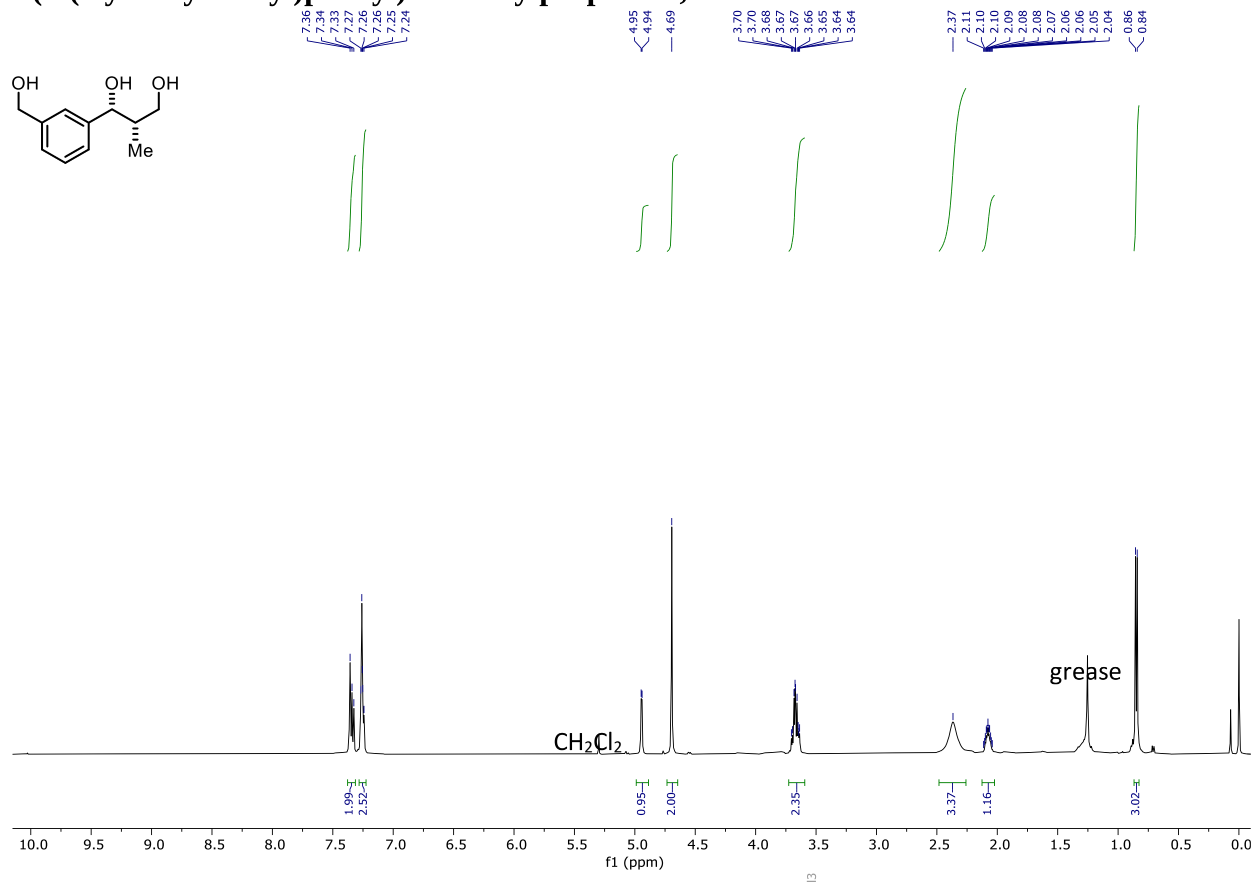

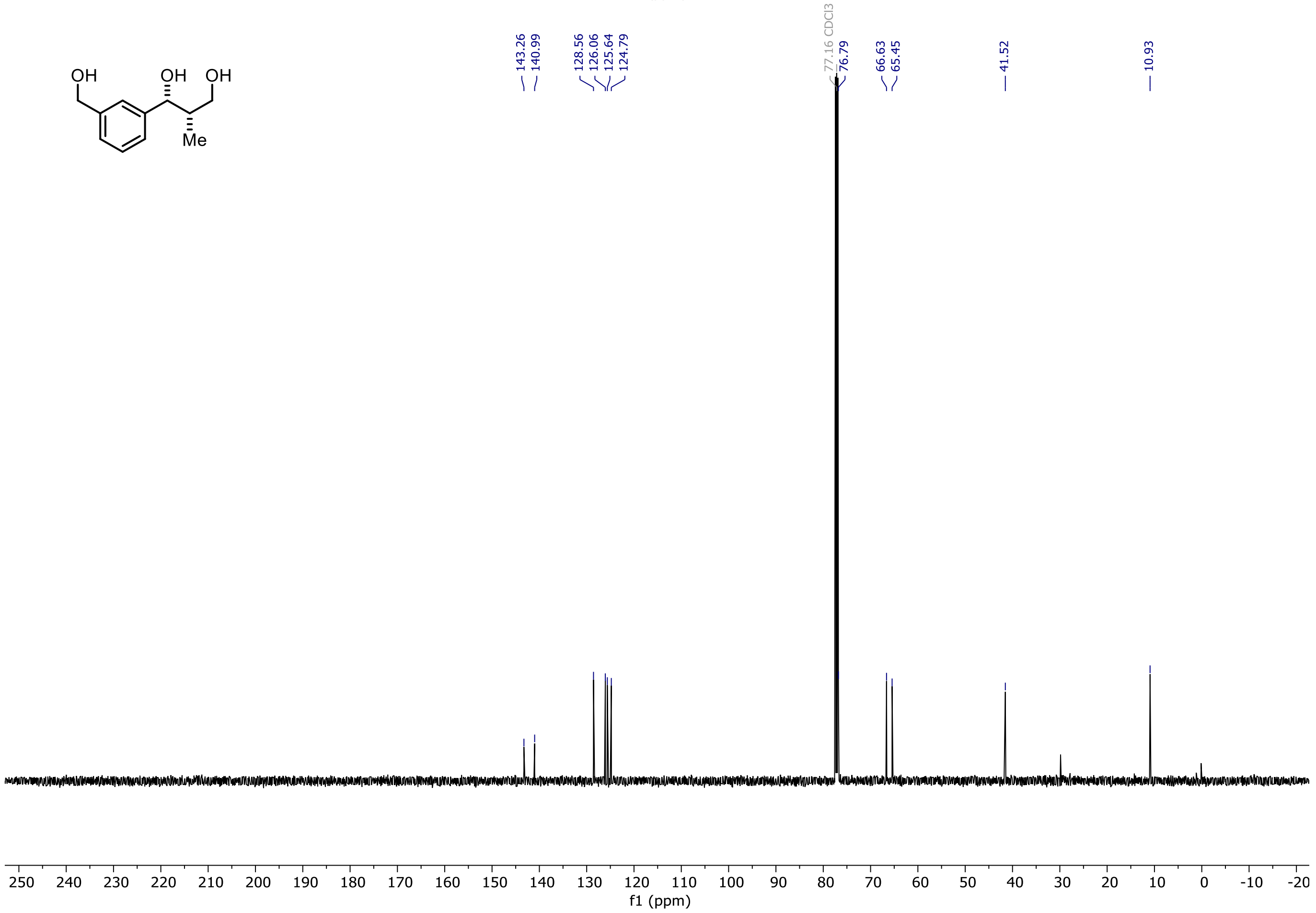

(*R*)-2-((*R*)-Phenyl((triethylsilyl)oxy)methyl)butanal

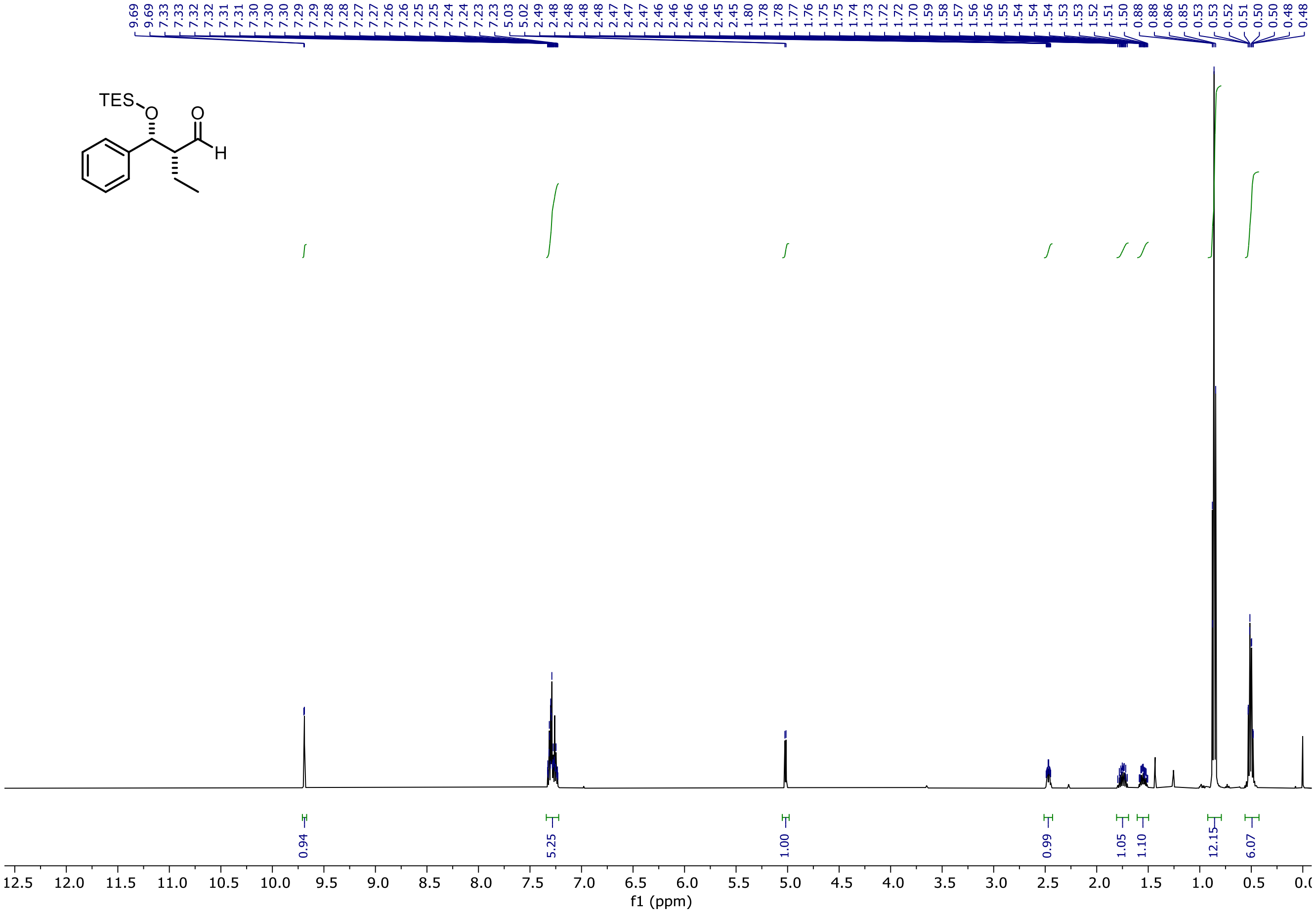

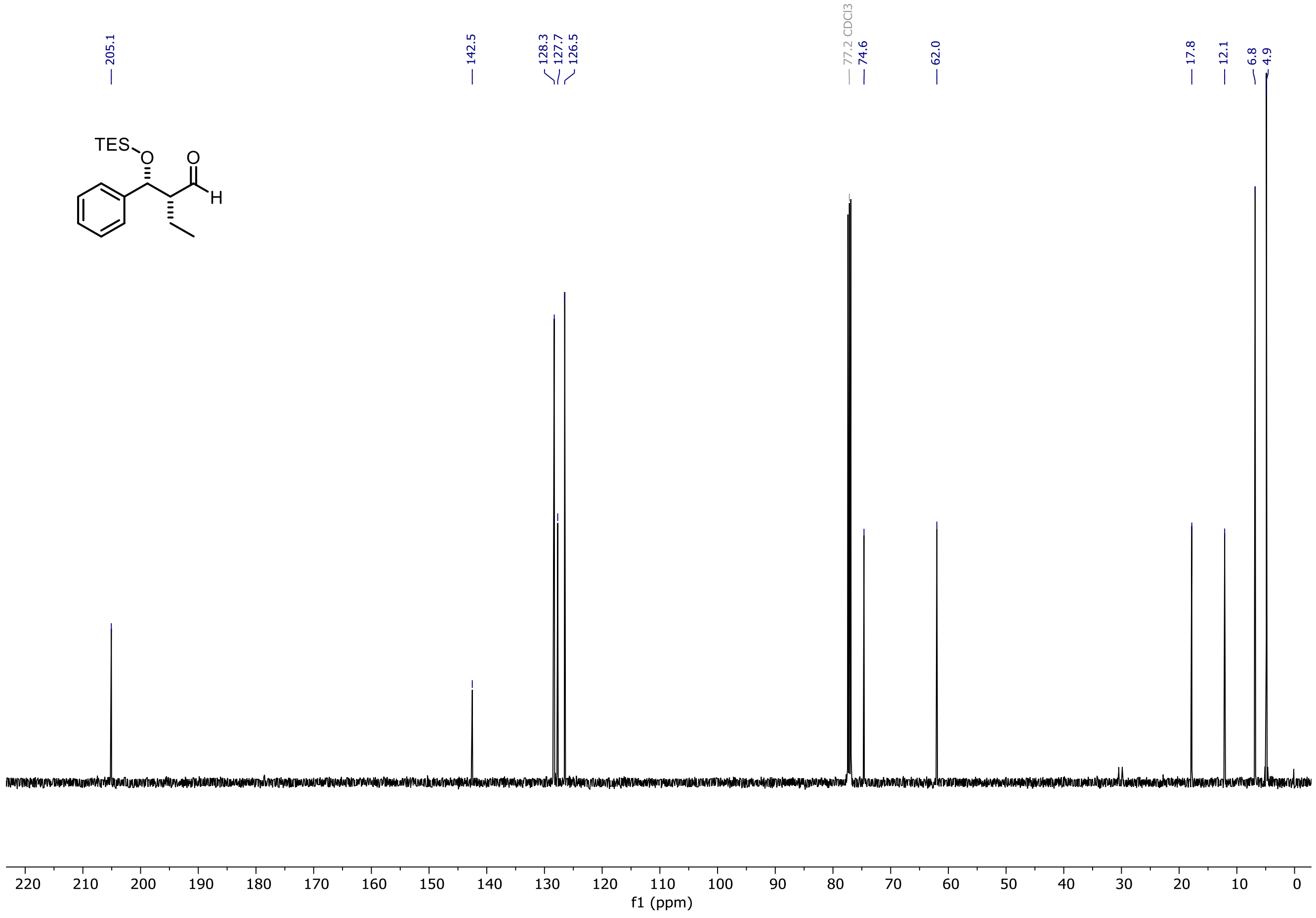

(1*R*,2*S*)-2-Ethyl-1-phenylpropane-1,3-diol

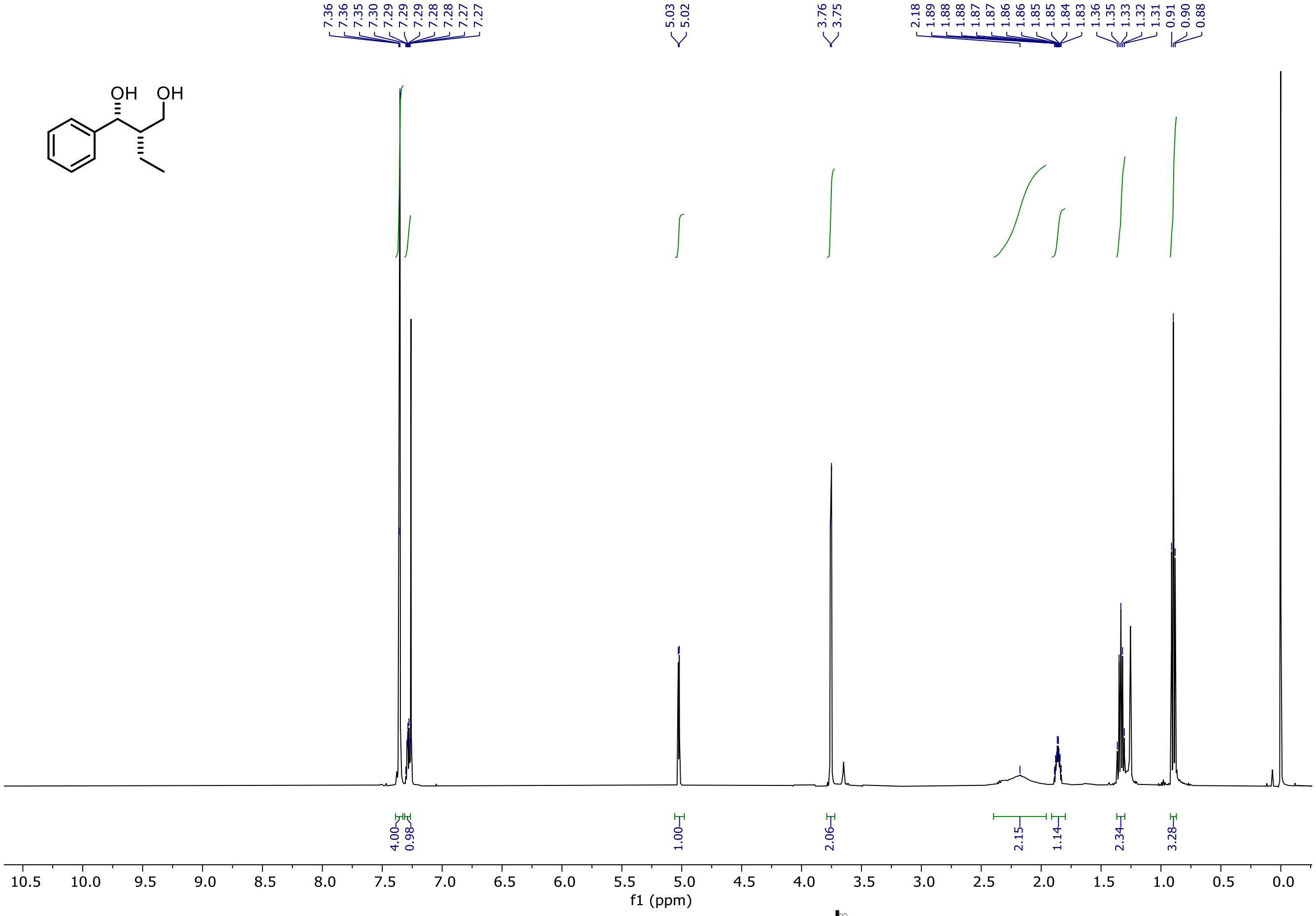

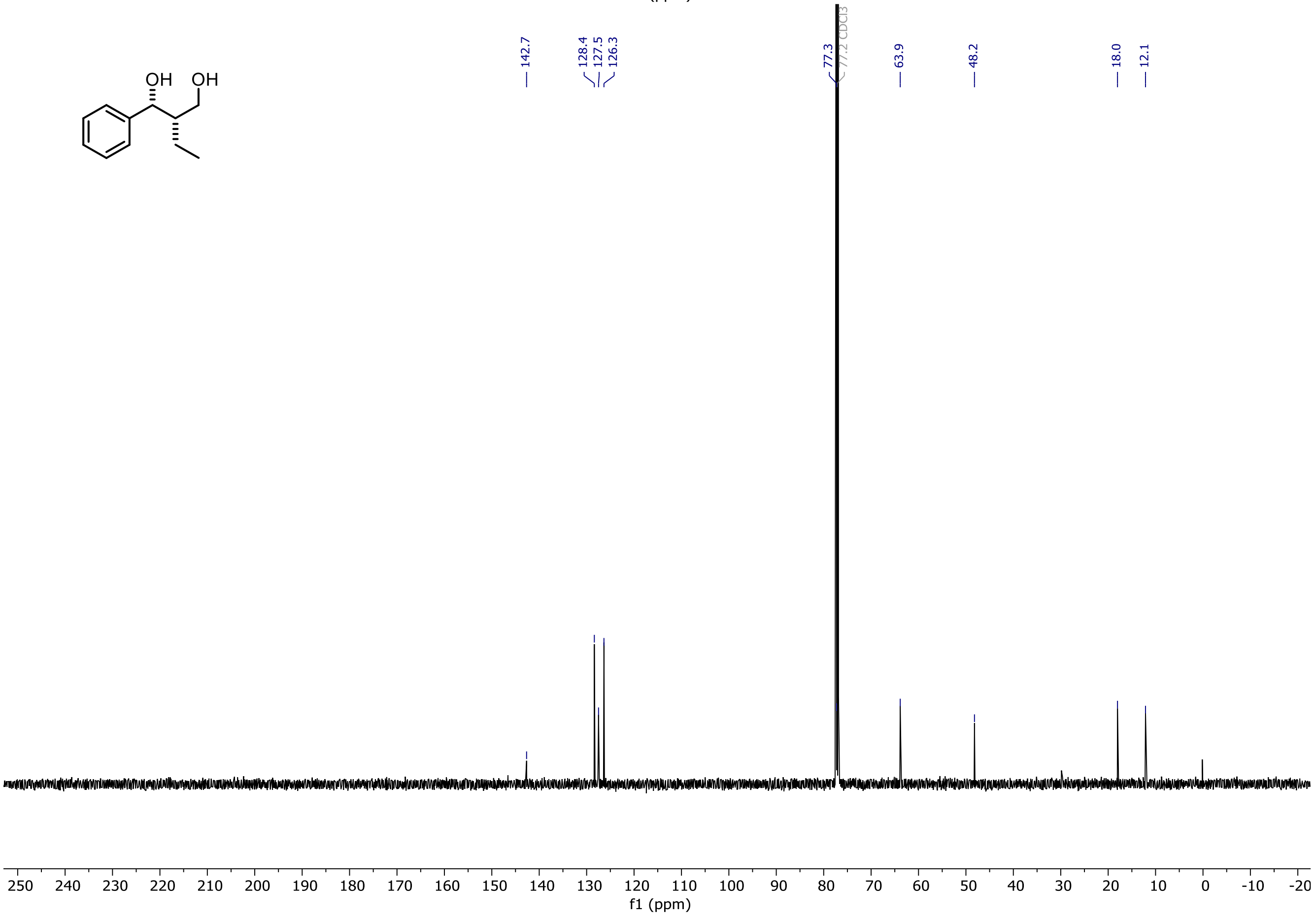

(2*S*,3*S*)-3-((*tert*-Butyldimethylsilyl)oxy)-2-methyl-3-phenylpropanal

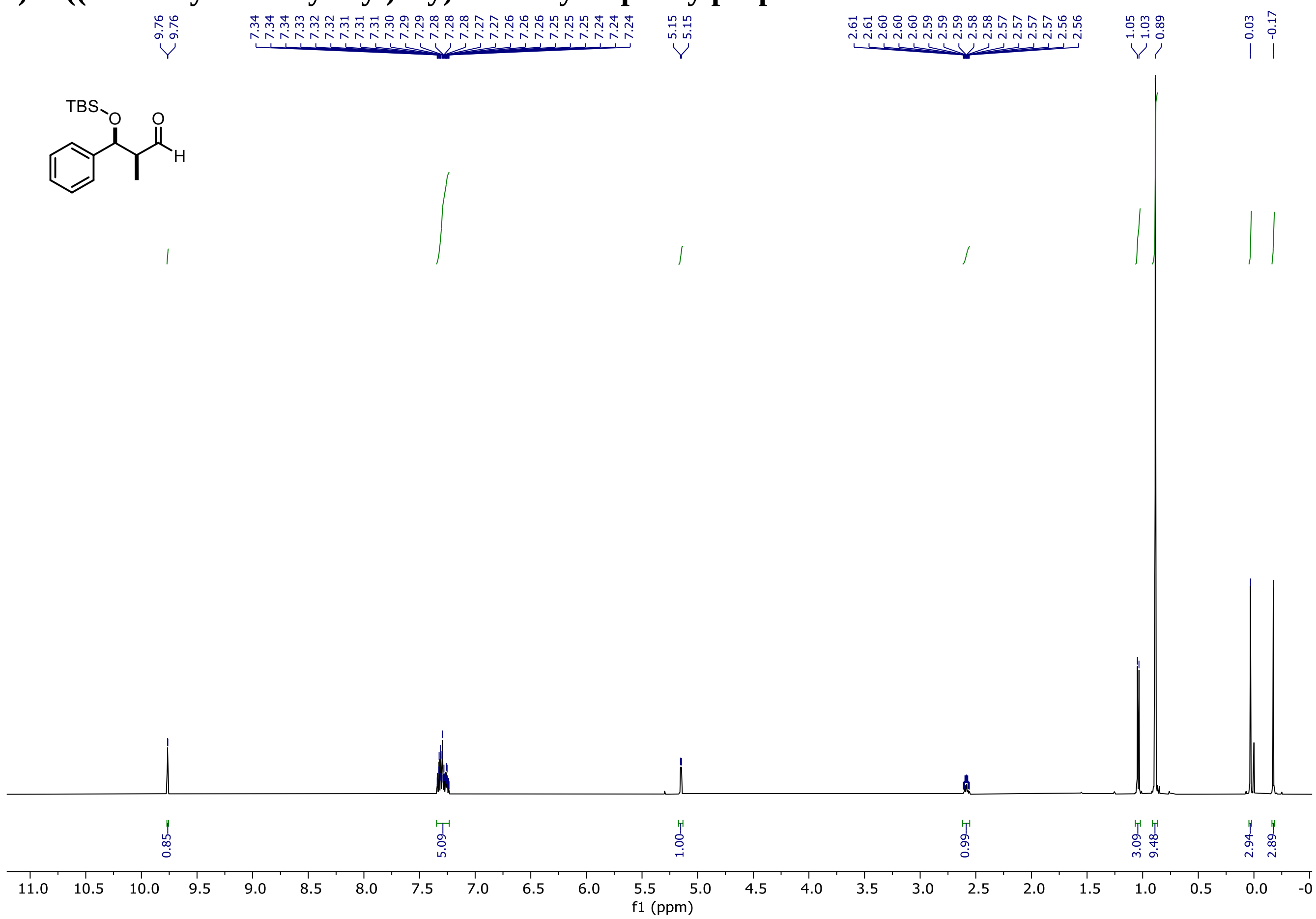

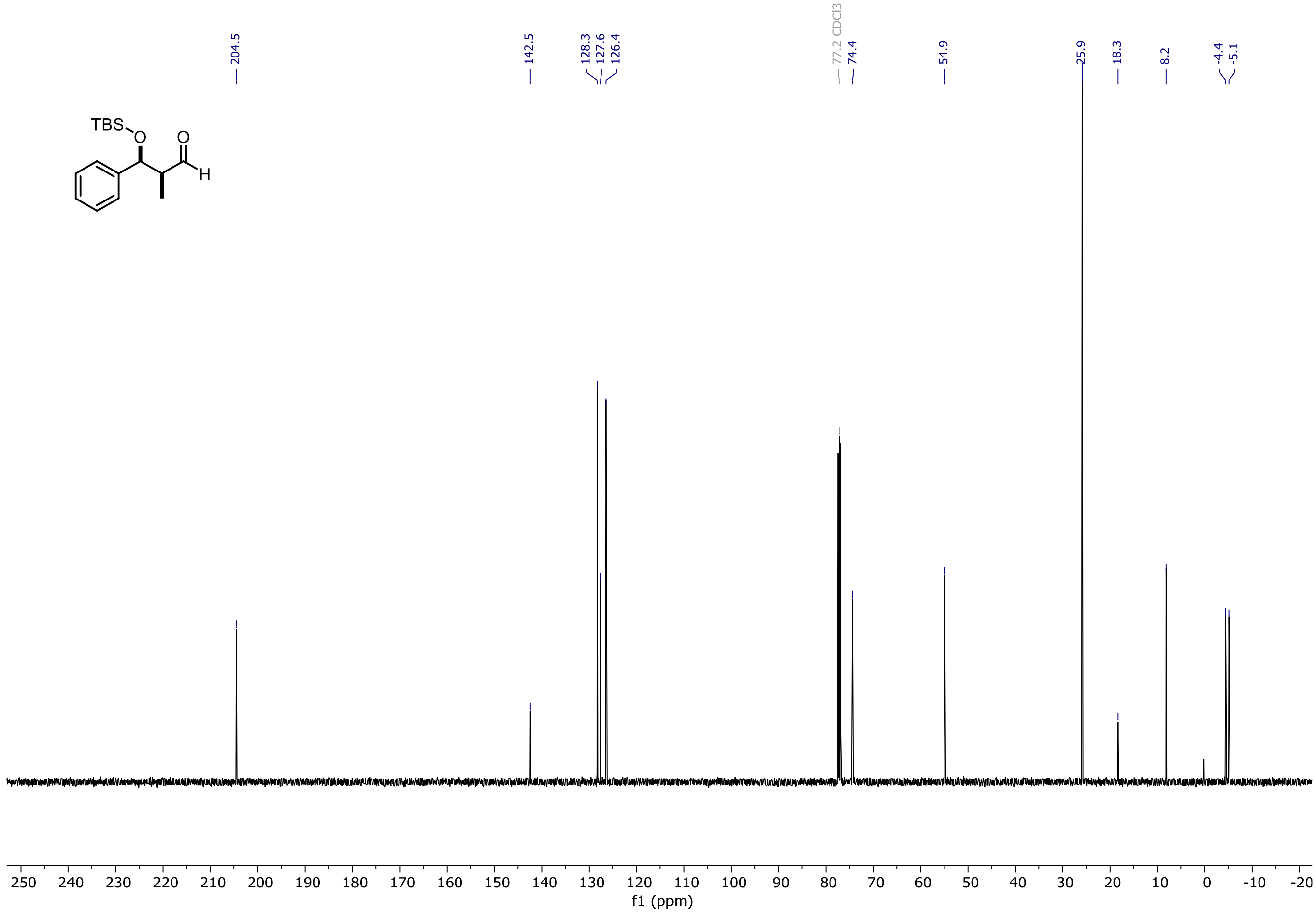

Methyl (2*E*,4*E*,6*R*,7*S*)-7-((tert-butyldimethylsilyl)oxy)-2,6-dimethyl-7-phenylhepta-2,4-dienoate

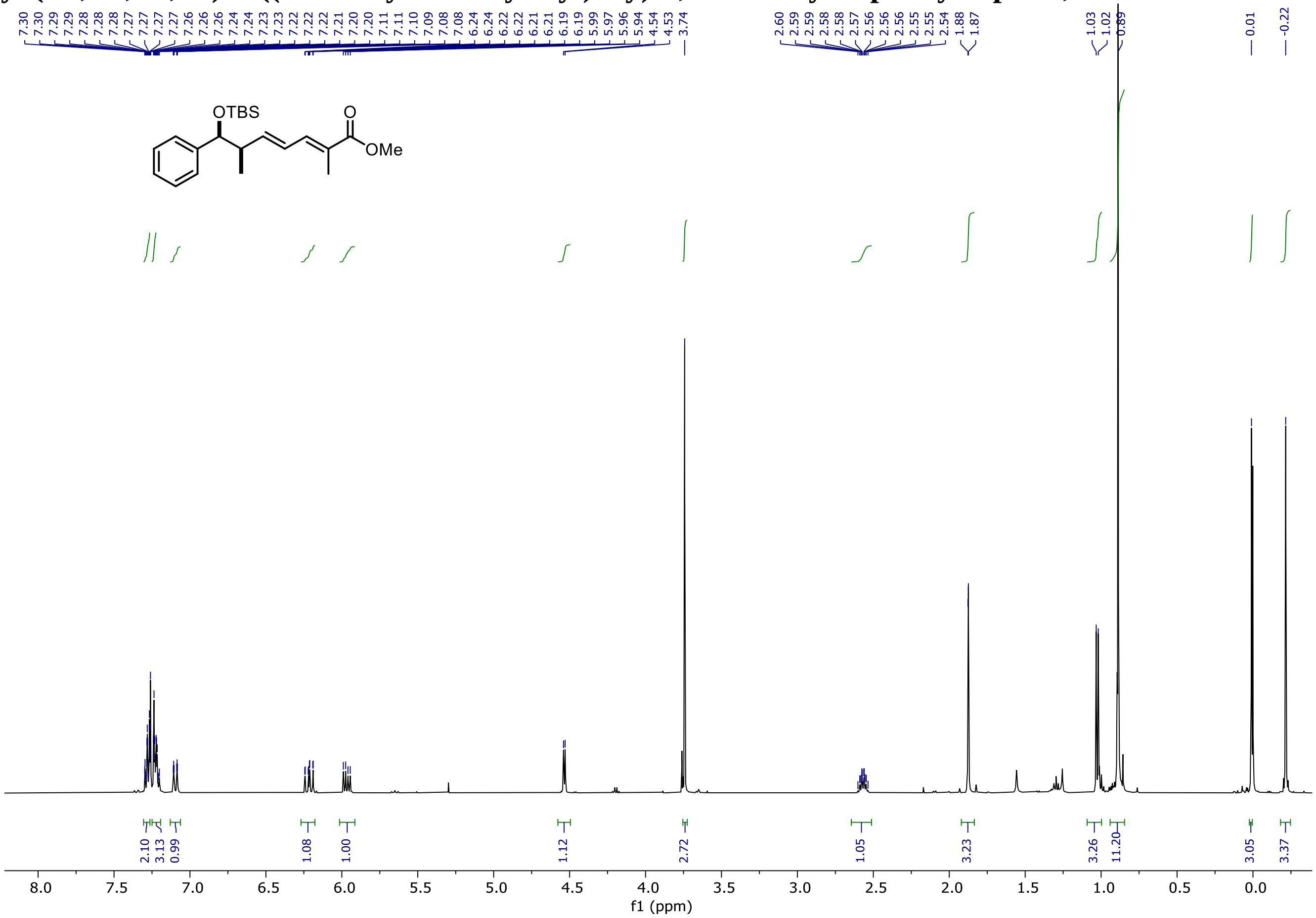

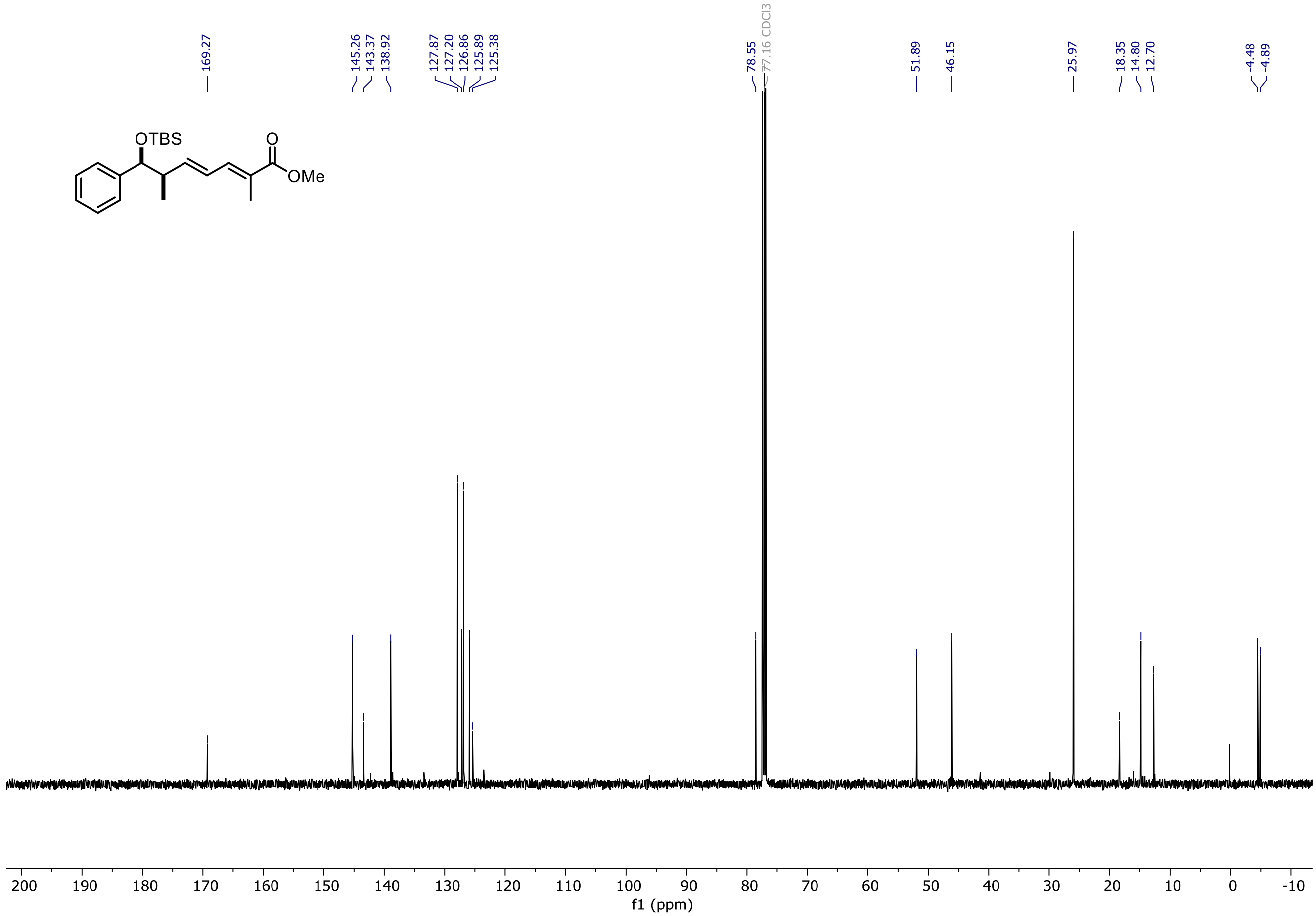

(2*R*,3*S*)-3-((*tert*-Butyldimethylsilyl)oxy)-2-methyl-3-phenylpropanal

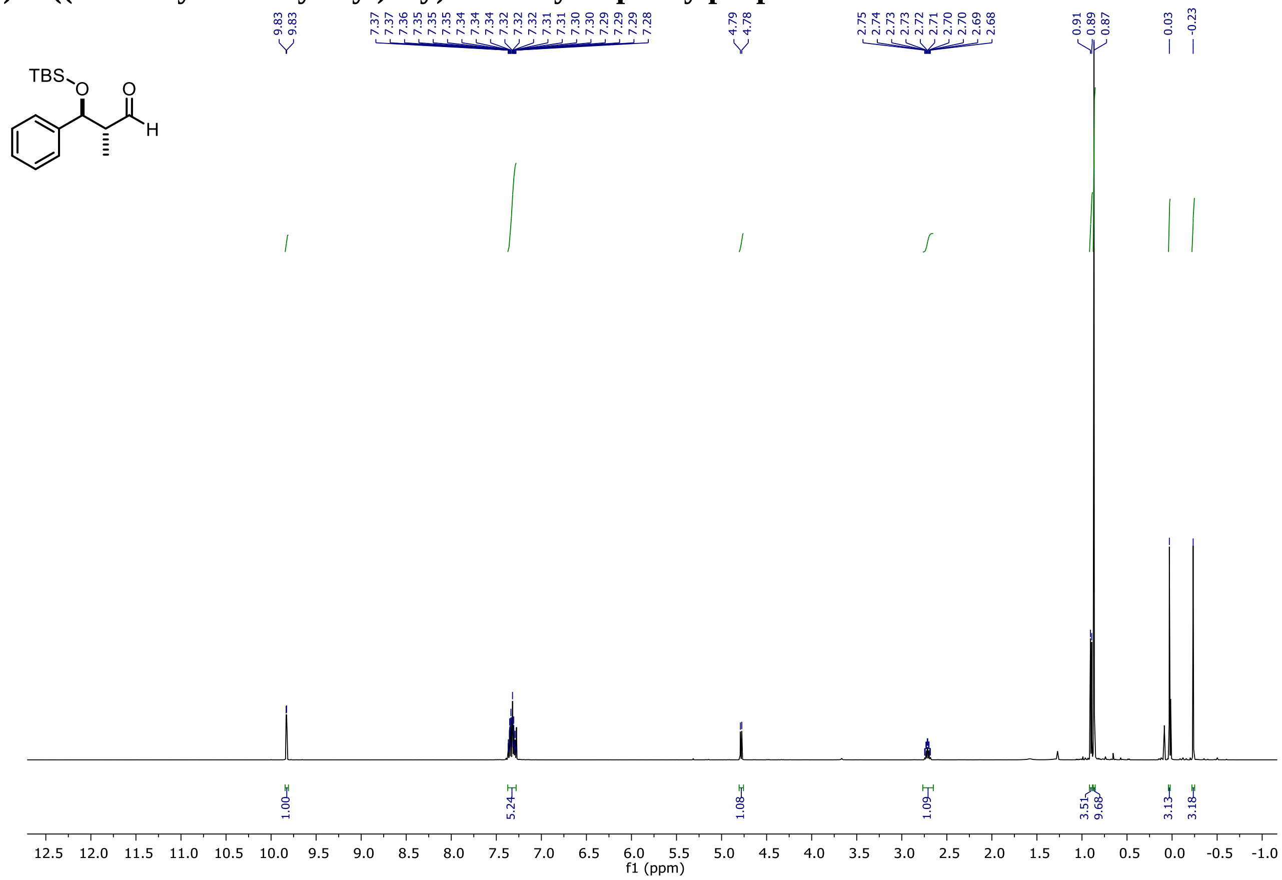

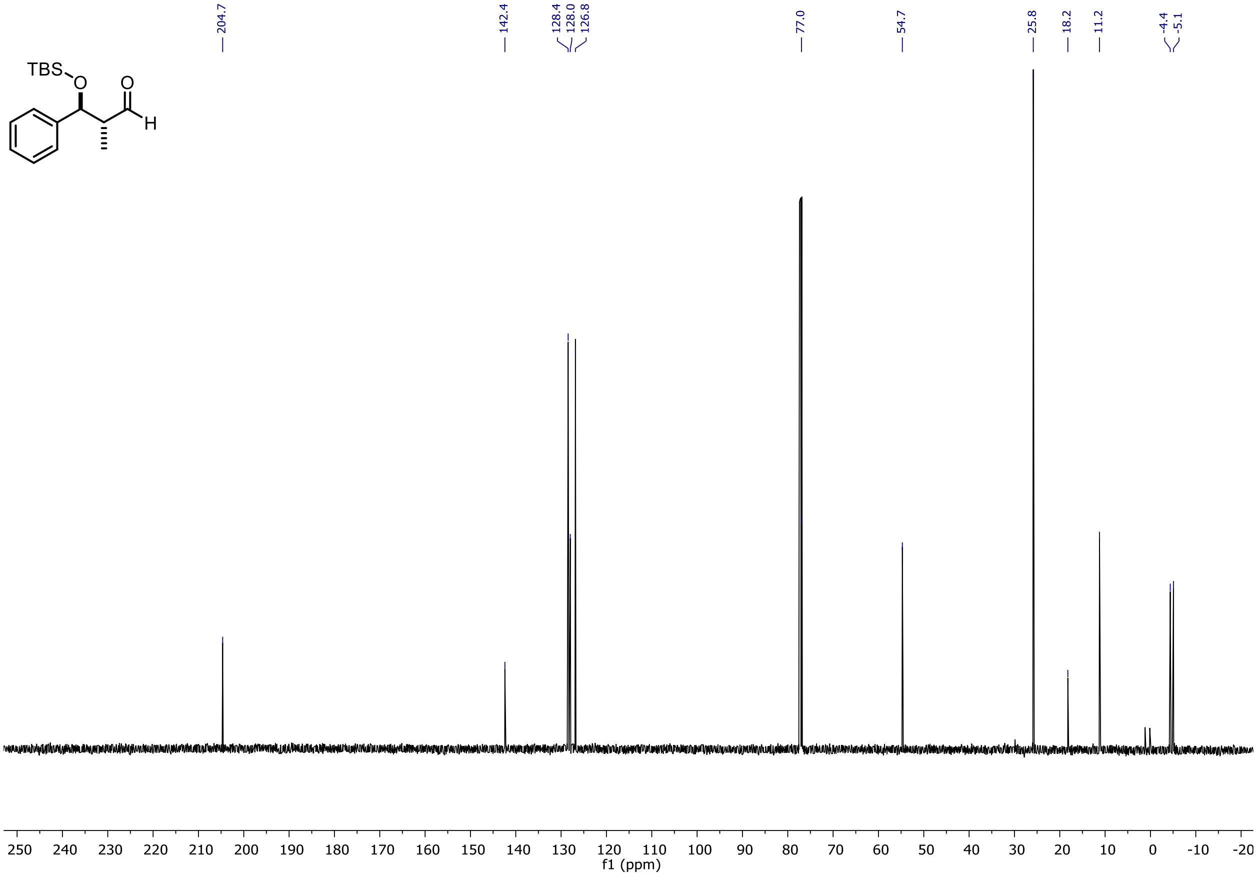

(1*S*,2*S*)-2-Methyl-1-phenylpropane-1,3-diol

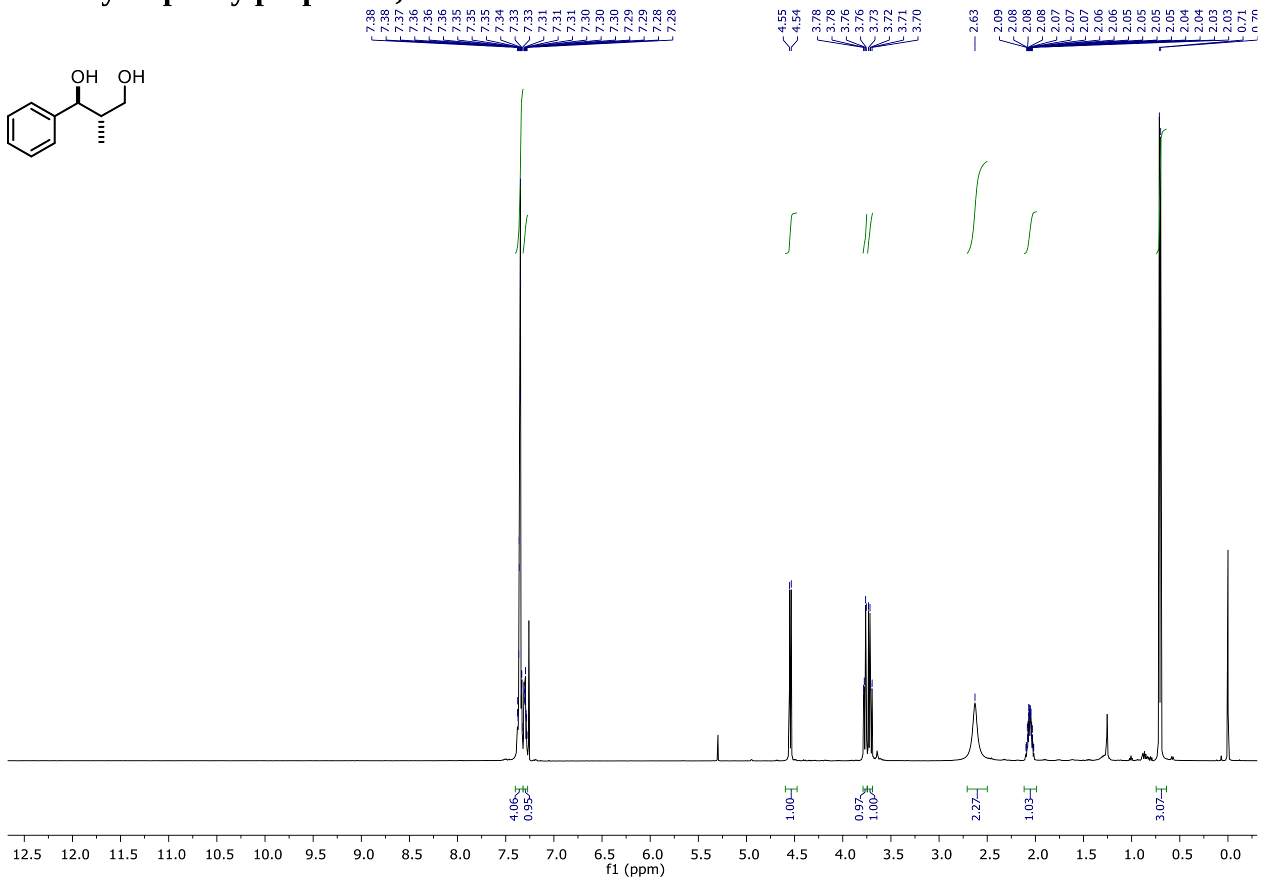

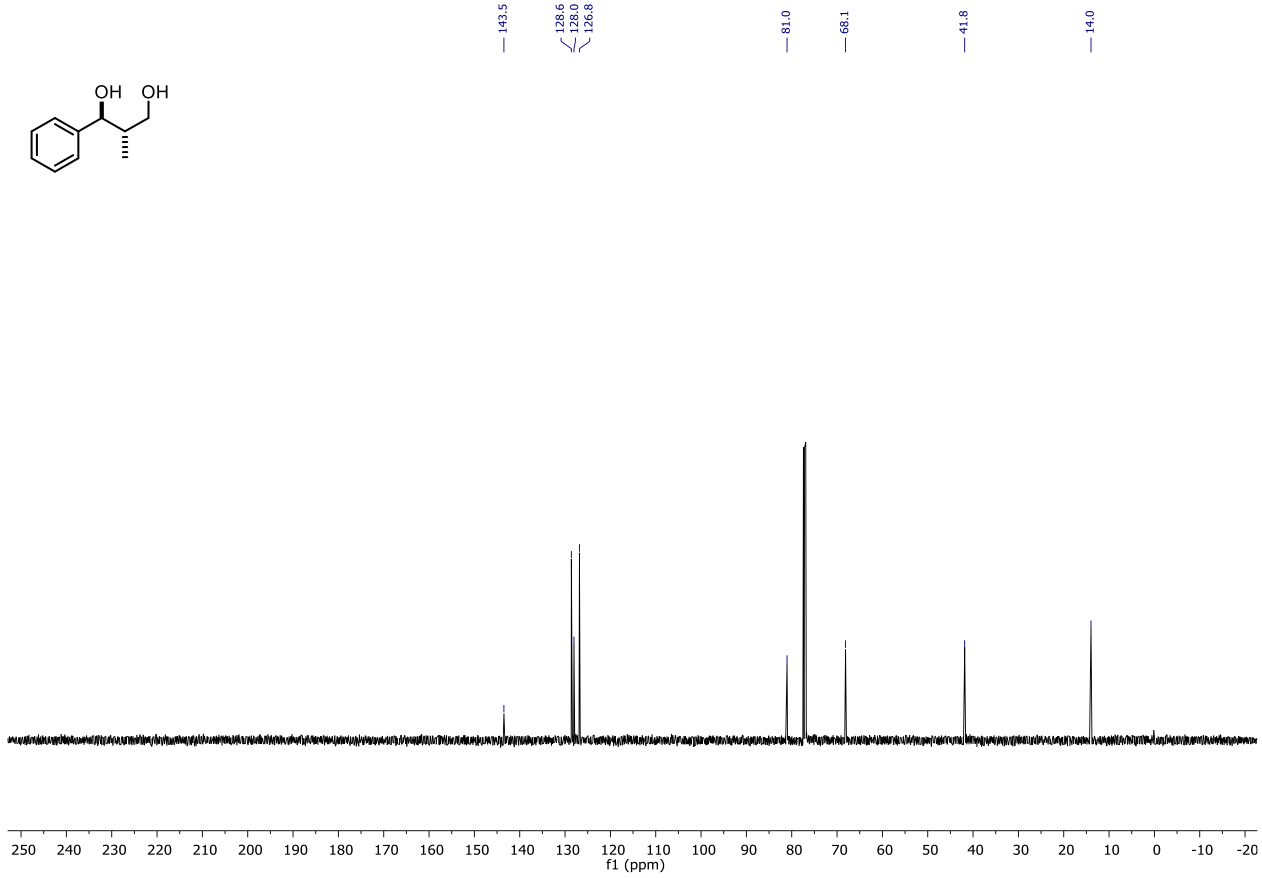

(2*R*,3*S*)-3-((*tert*-Butyldimethylsilyl)oxy)-3-(3-methoxyphenyl)-2-methylpropanal.

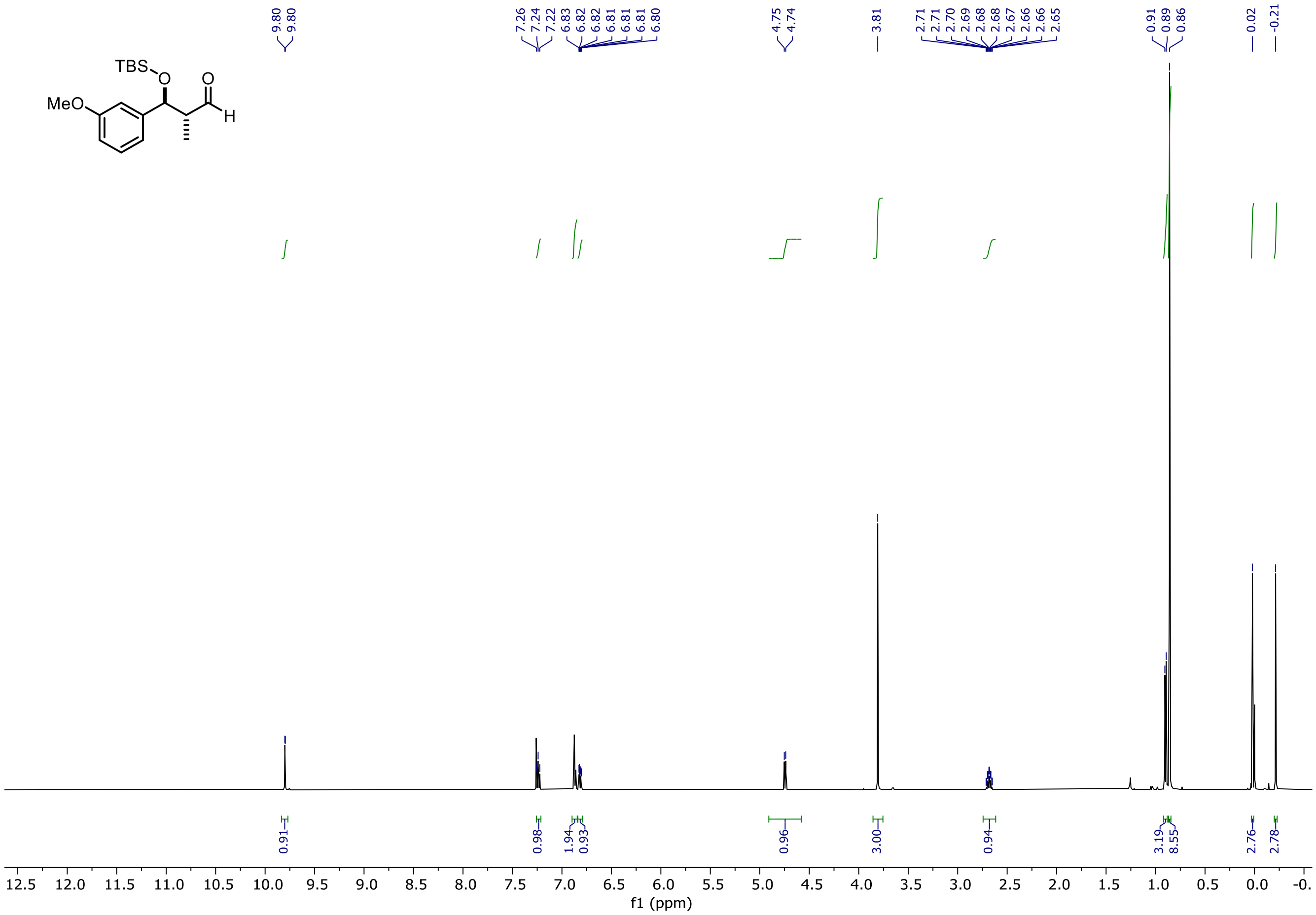

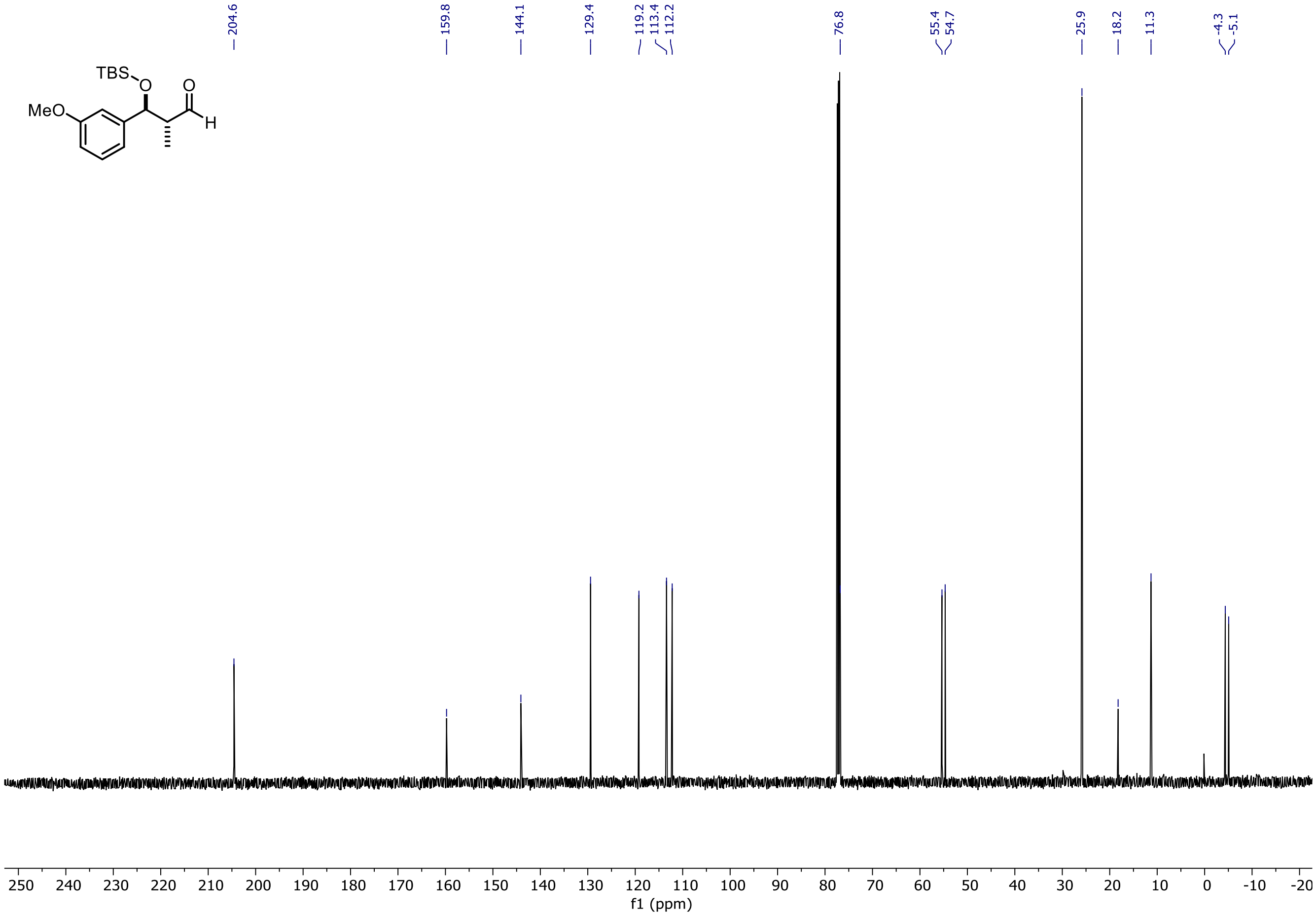

(3*R*,4*S*,5*S*)-5-((*tert*-Butyldimethylsilyl)oxy)-3-((1,1,1,3,3,3-hexamethyl-2-(trimethylsilyl)trisilan-2-yl)oxy)-5-(3-methoxyphenyl)-4-methylpentanal

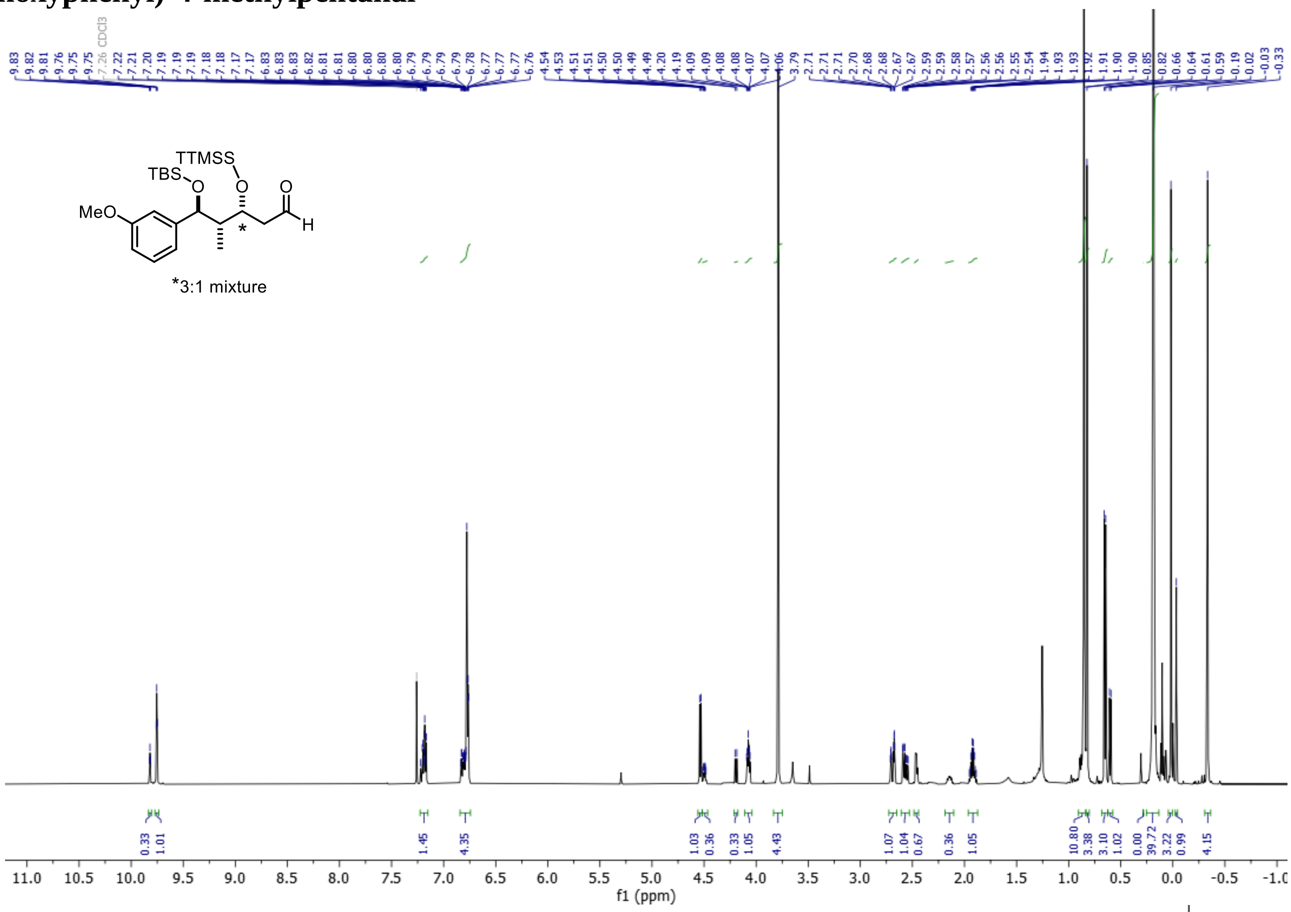

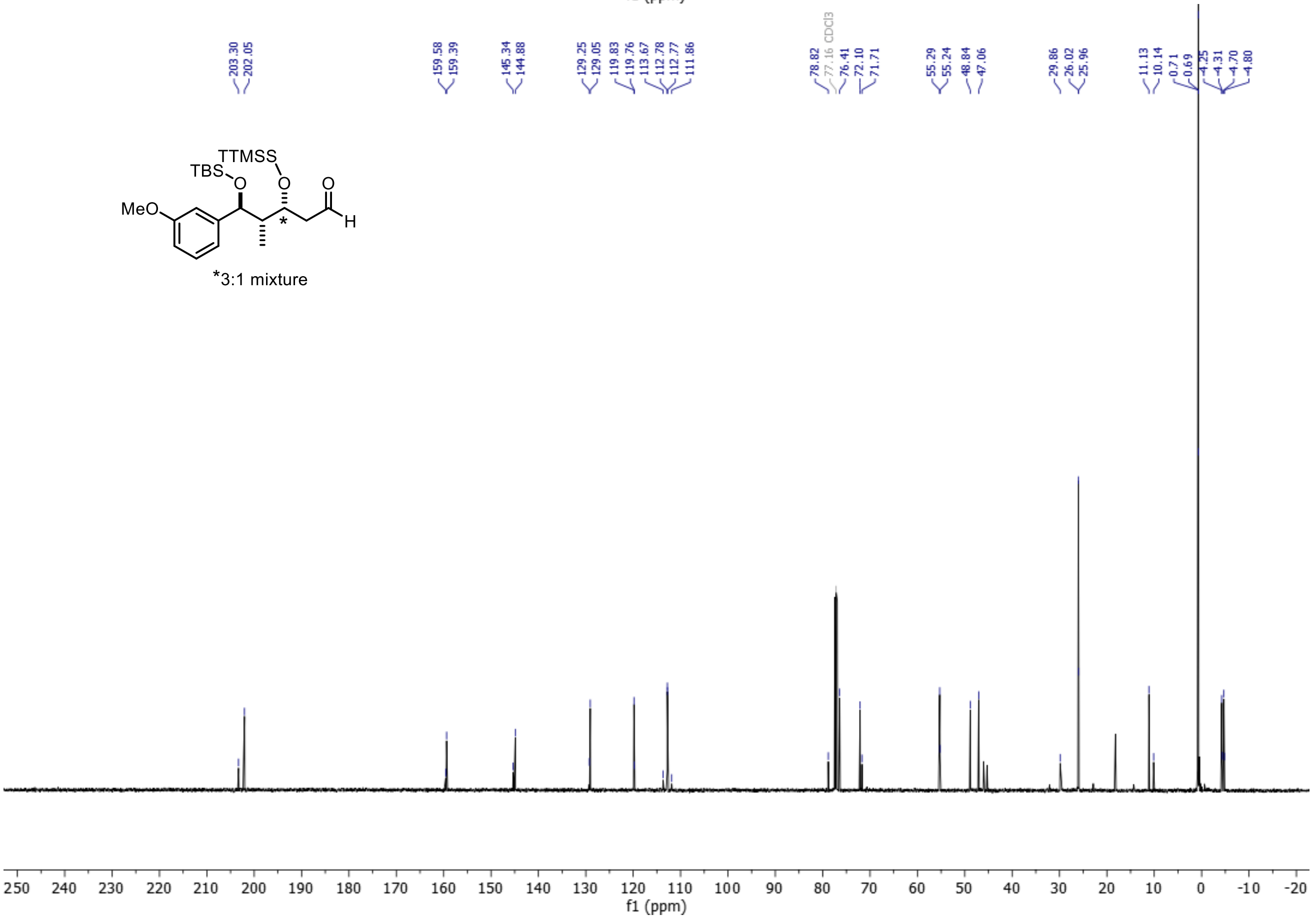

(2*R*,3*S*)-3-((*tert*-Butyldimethylsilyl)oxy)-3-(4-methoxyphenyl)-2-methylpropanal

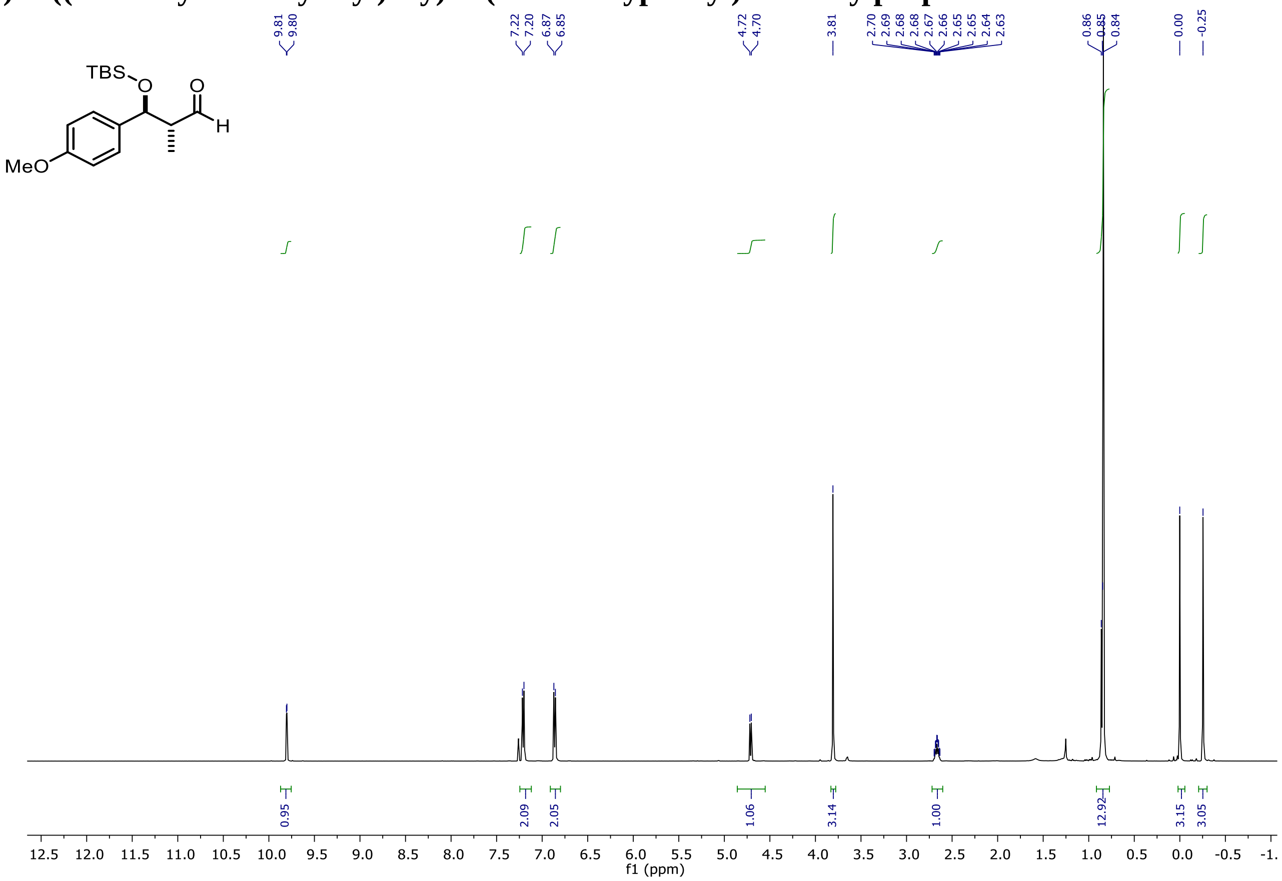

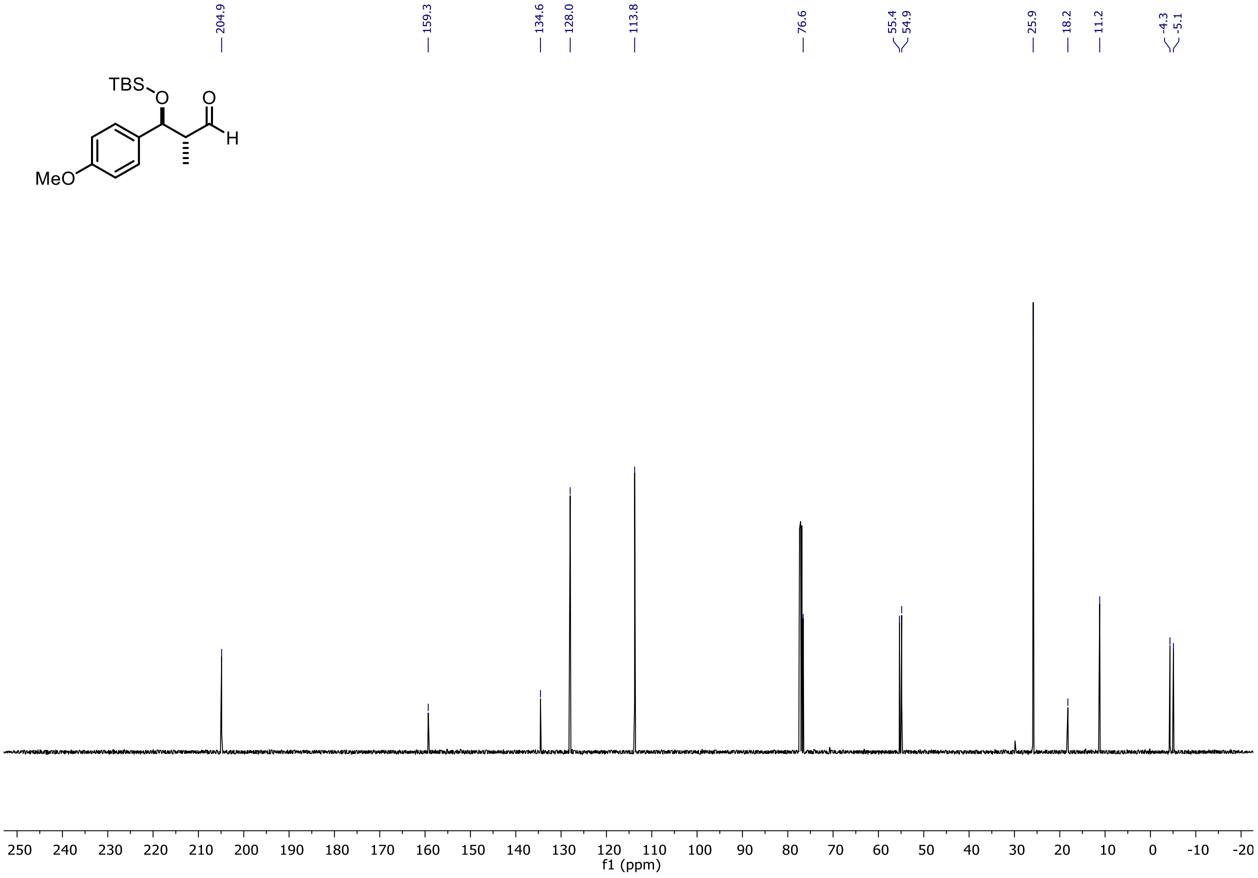

(1*S*,2*S*)-1-(4-Methoxyphenyl)-2-methylpropane-1,3-diol

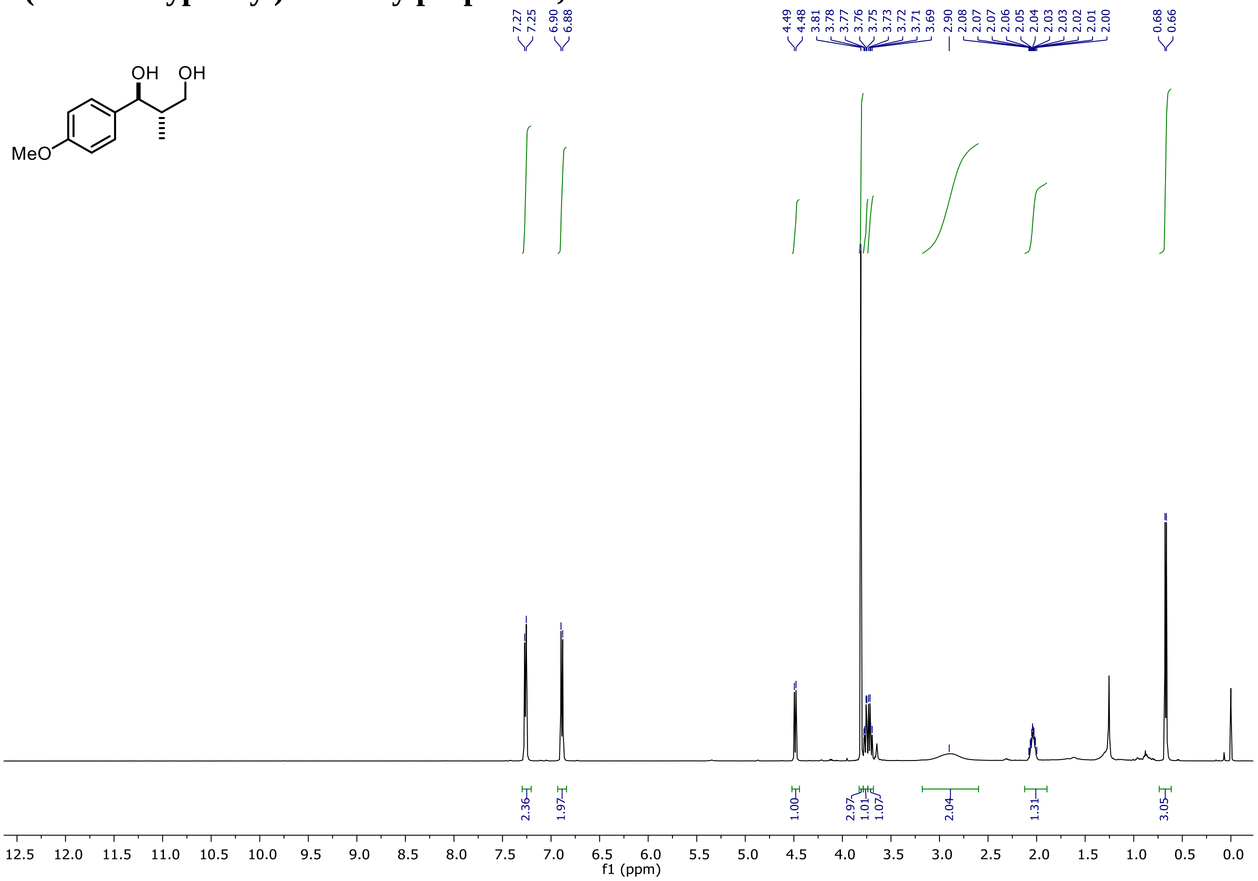

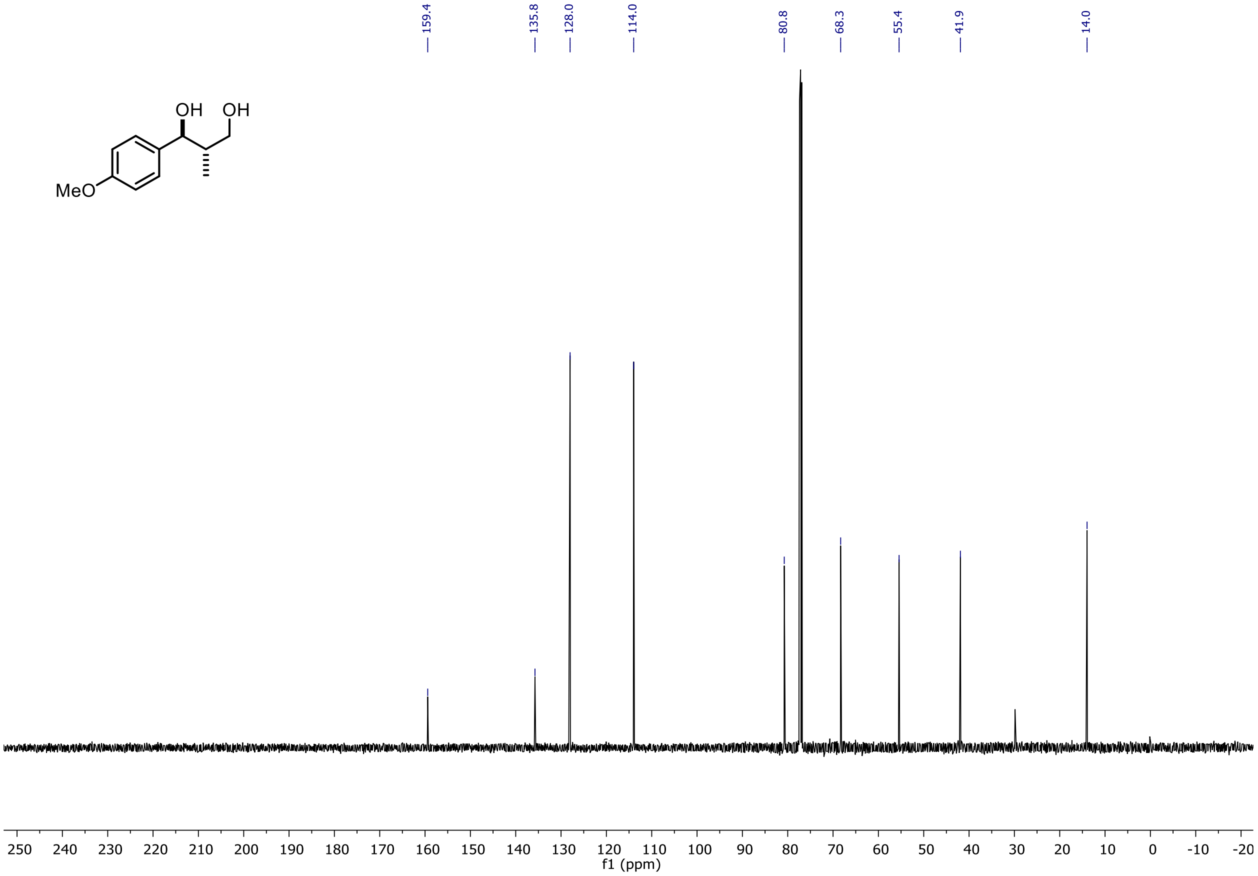

(2*R*,3*S*)-3-((*tert*-Butyldimethylsilyl)oxy)-2-methyl-3-(*o*-tolyl)propanal

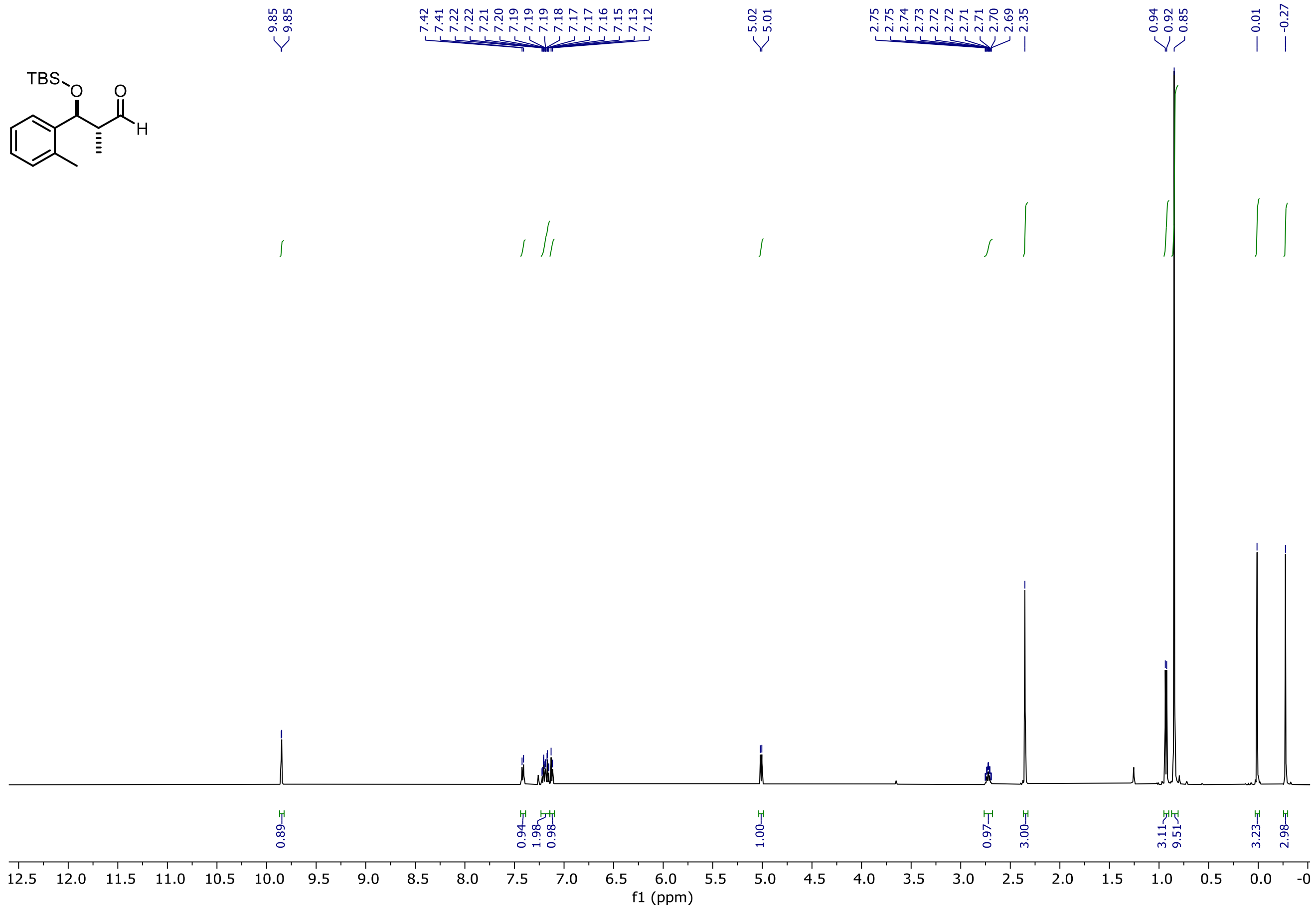

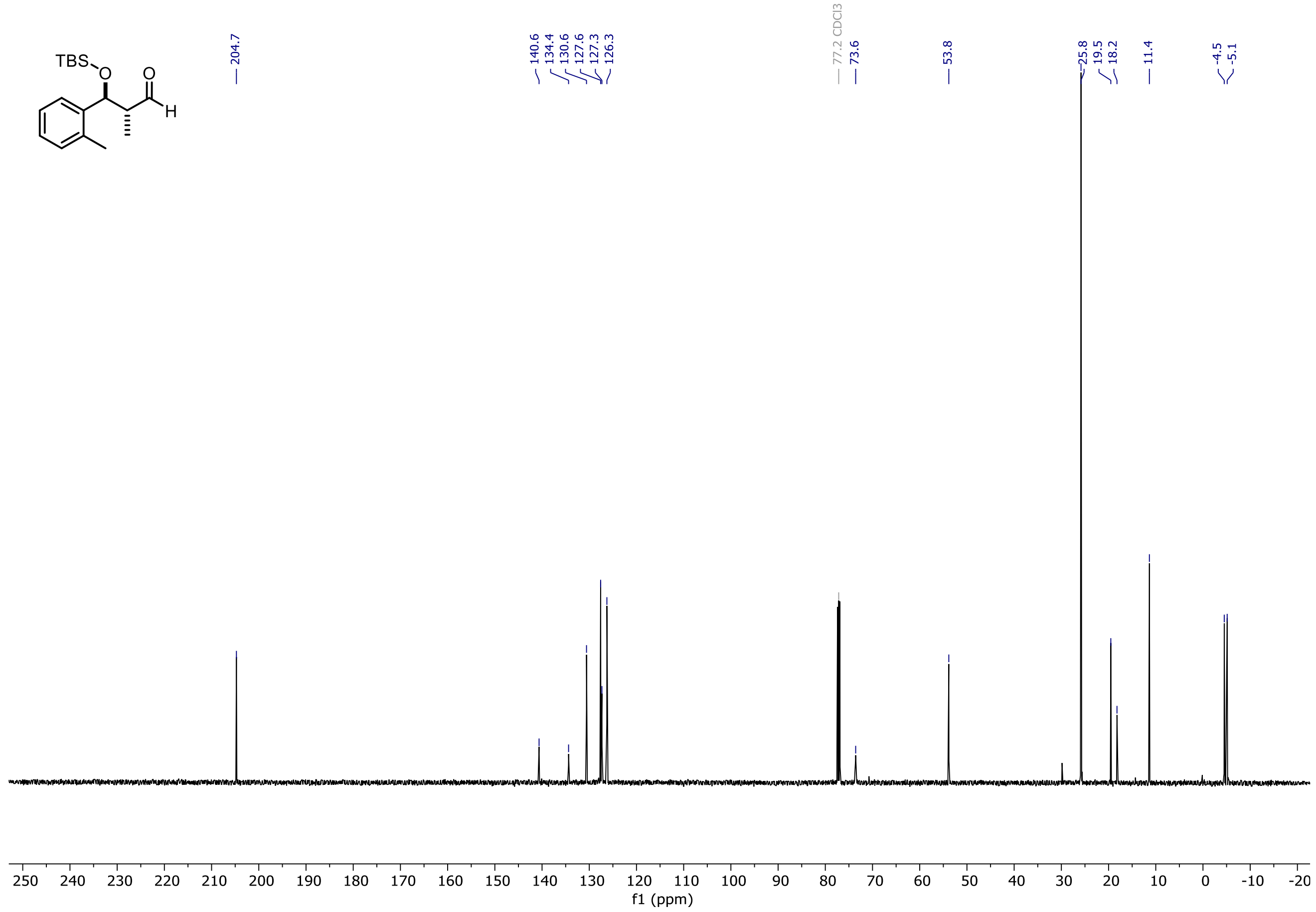

(1*S*,2*S*)-2-Methyl-1-(*o*-tolyl)propane-1,3-diol

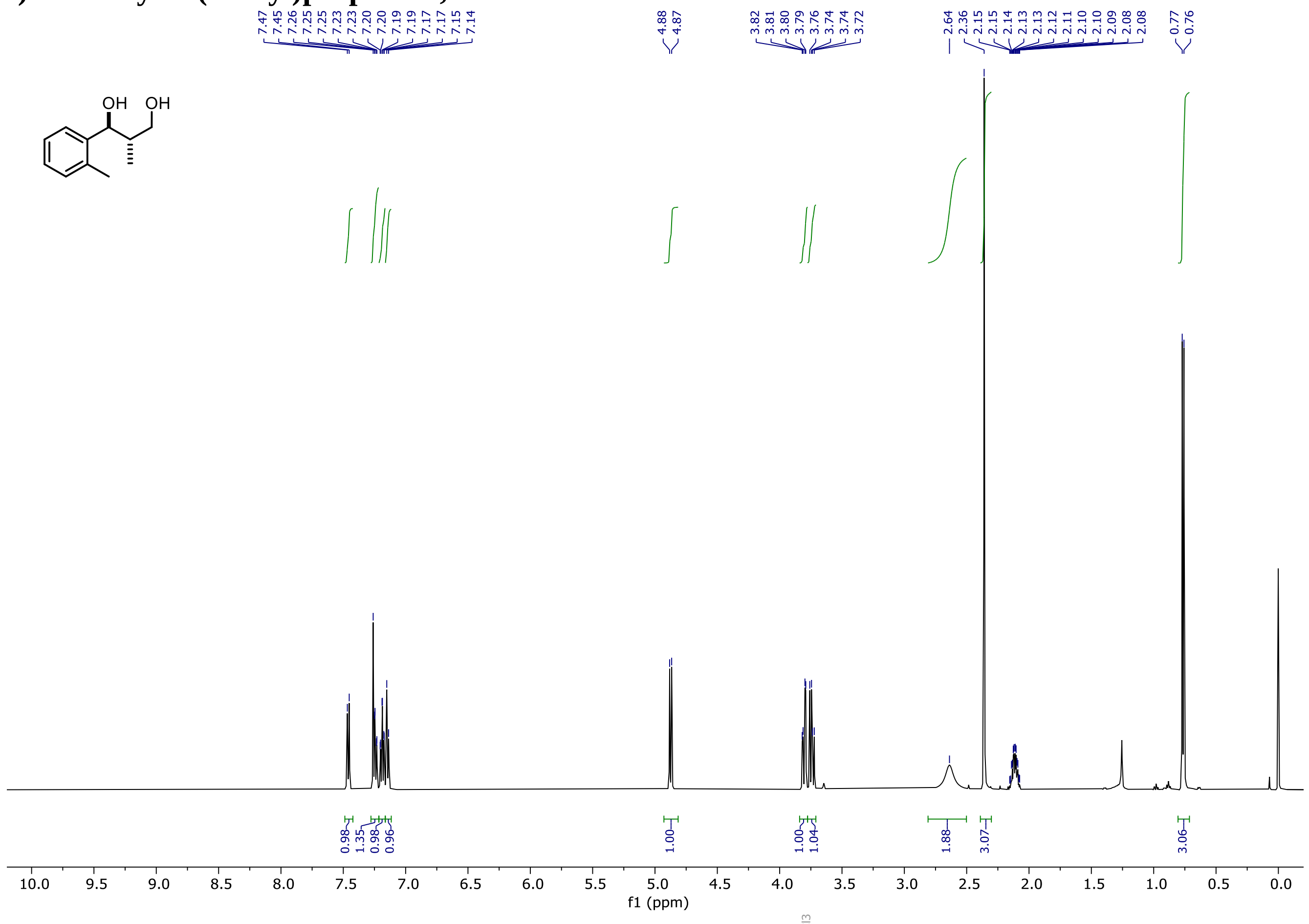

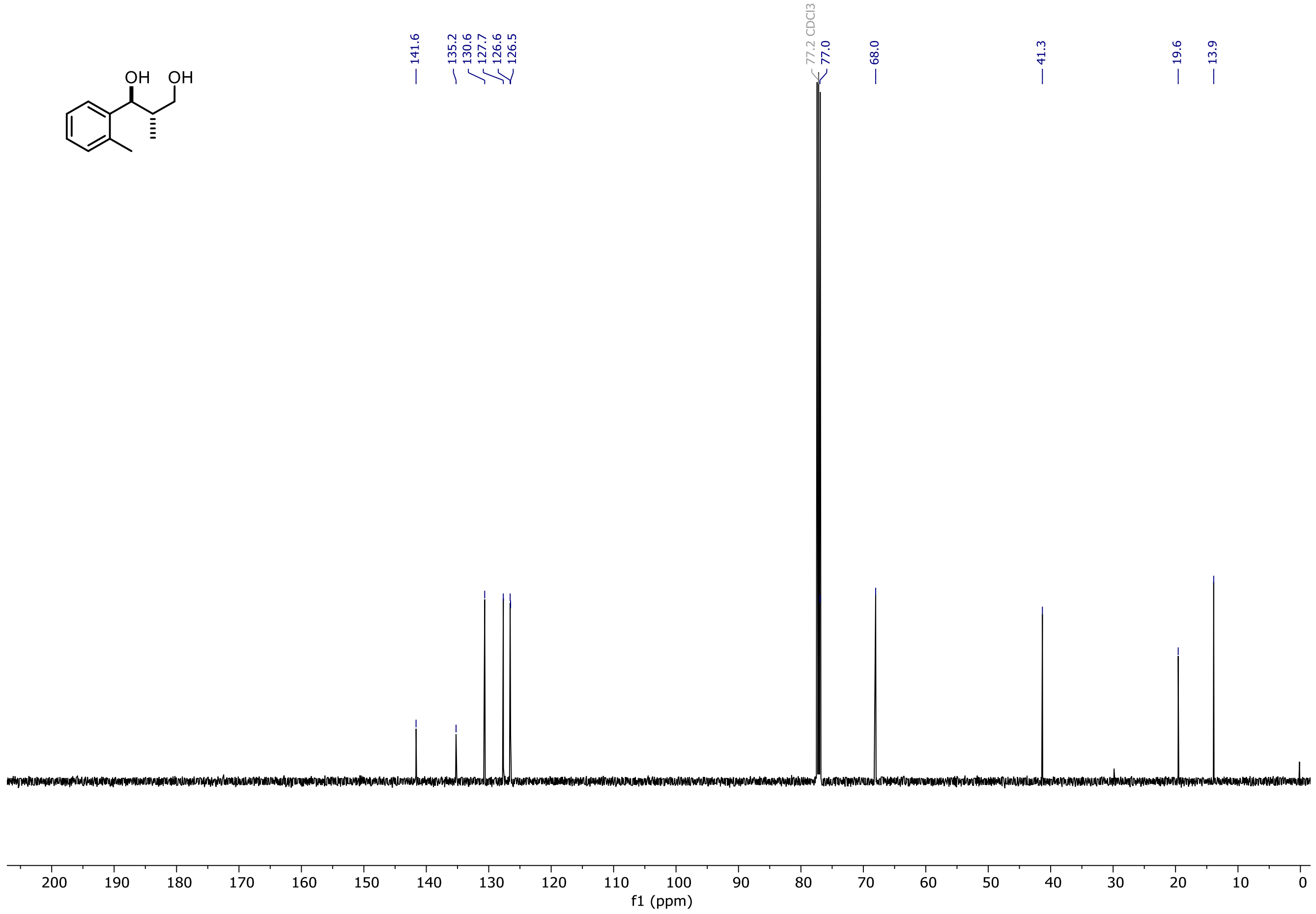

(2*R*,3*S*)-3-((*tert*-Butyldimethylsilyl)oxy)-2-methyl-3-(*m*-tolyl)propanal

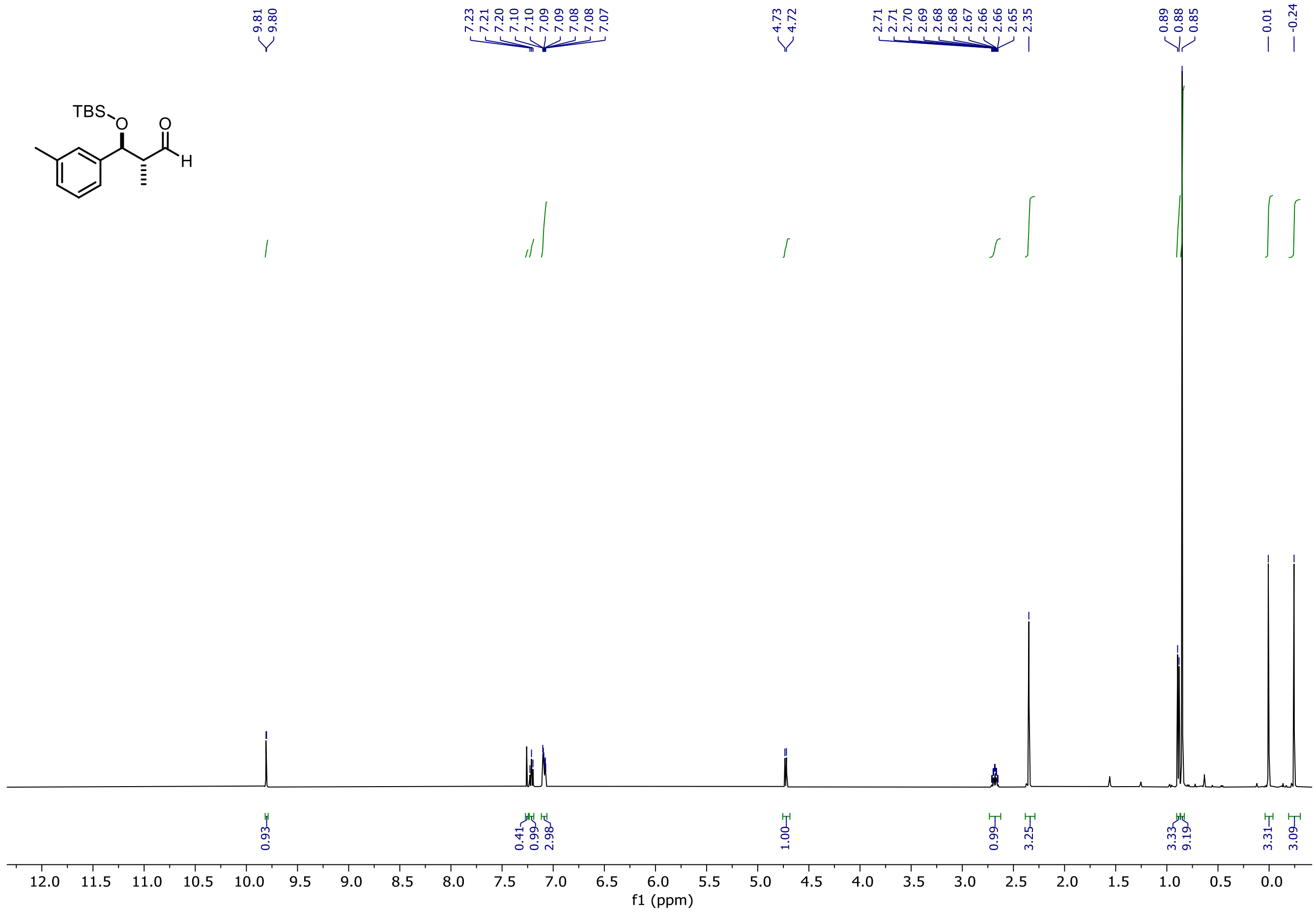

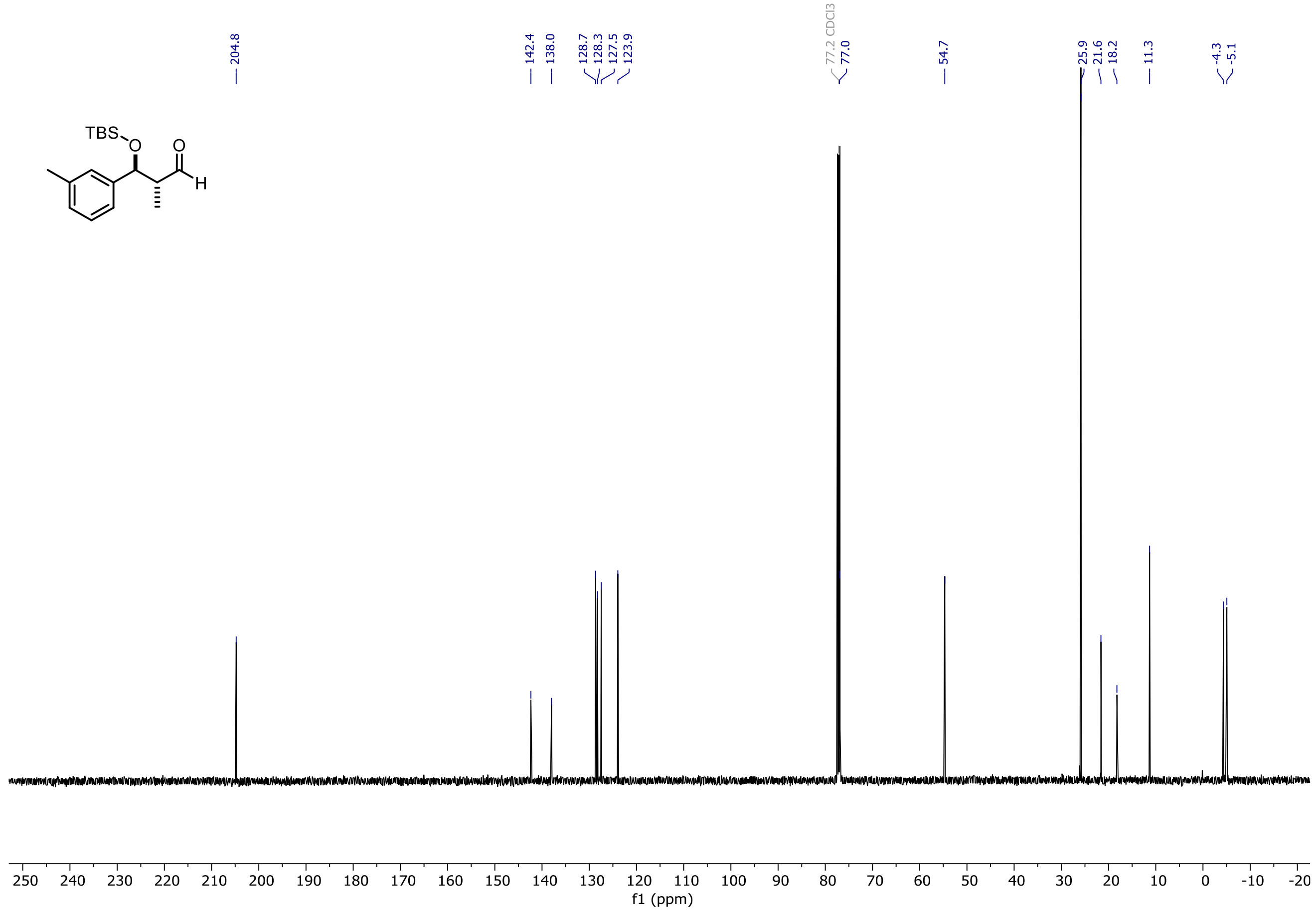

(1*S*,2*S*)-2-Methyl-1-(*m*-tolyl)propane-1,3-diol

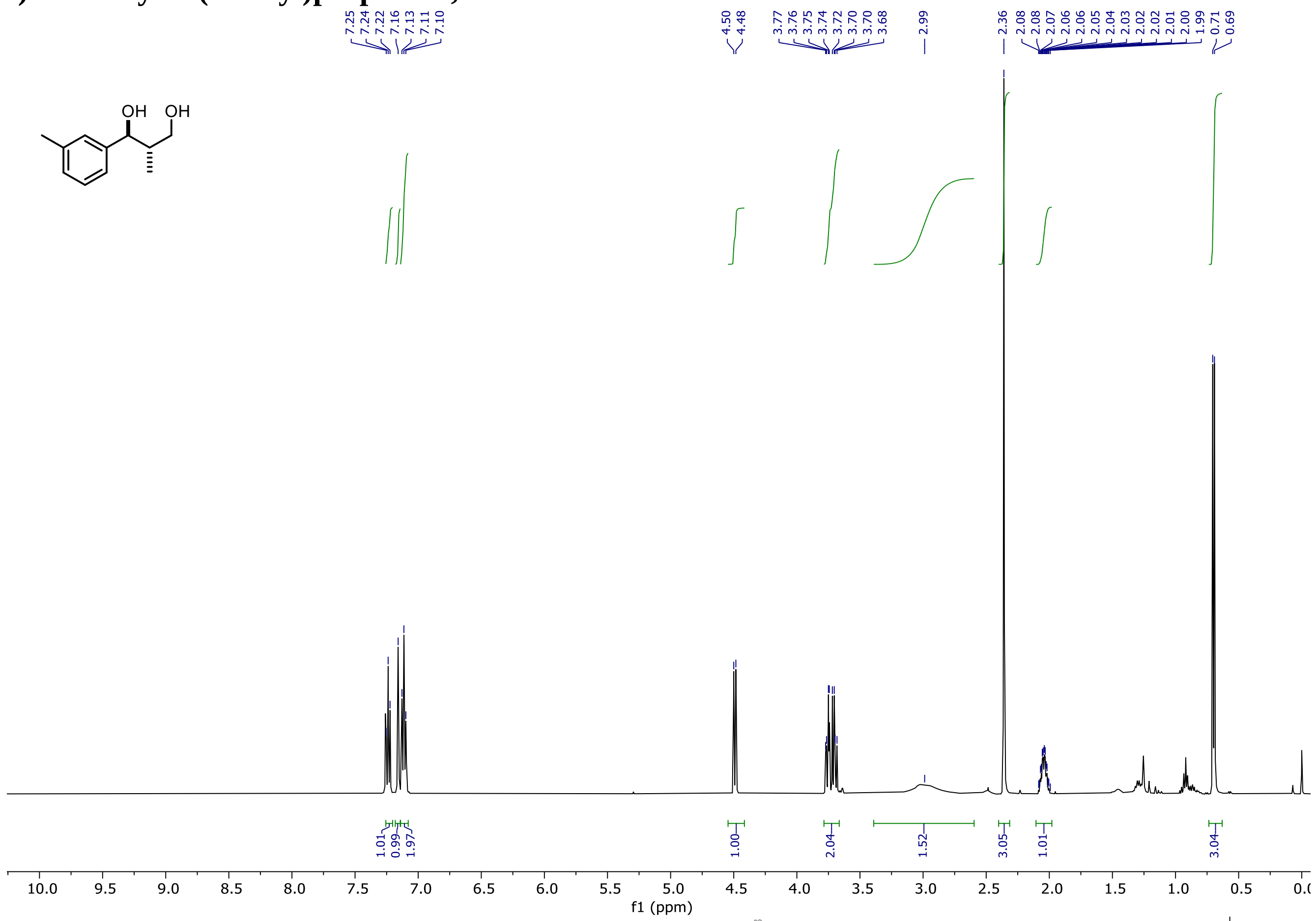

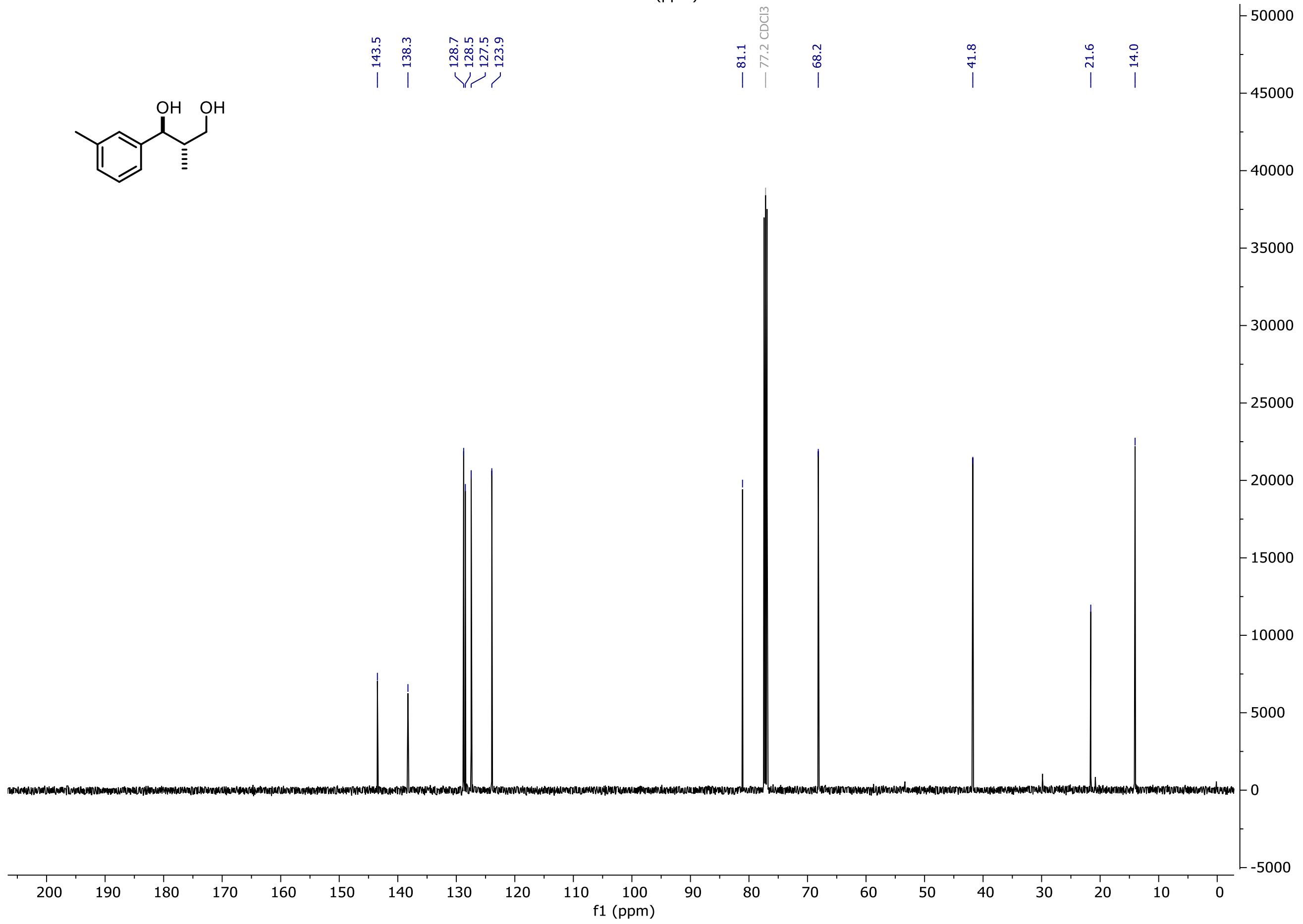

(2*R*,3*S*)-3-((*tert*-Butyldimethylsilyl)oxy)-2-methyl-3-(*p*-tolyl)propanal

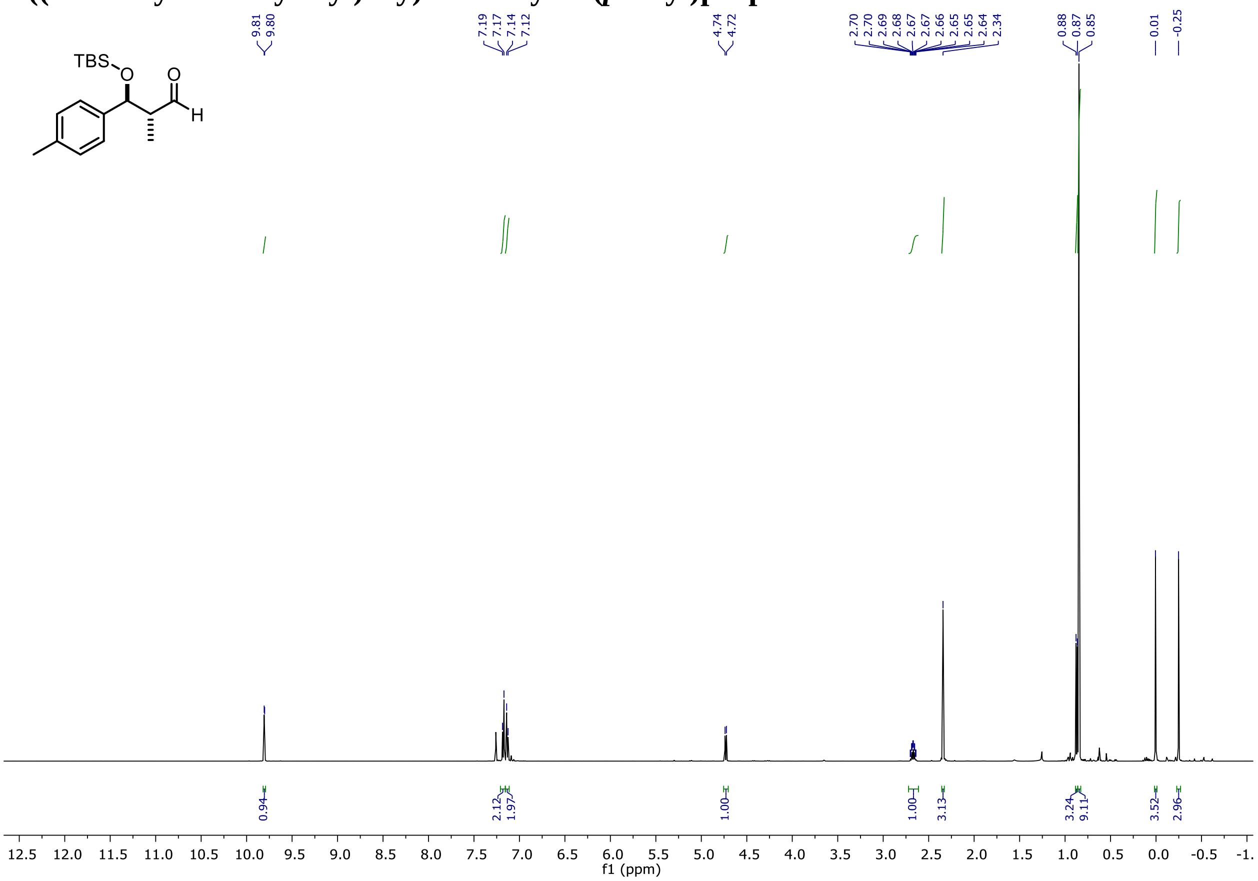

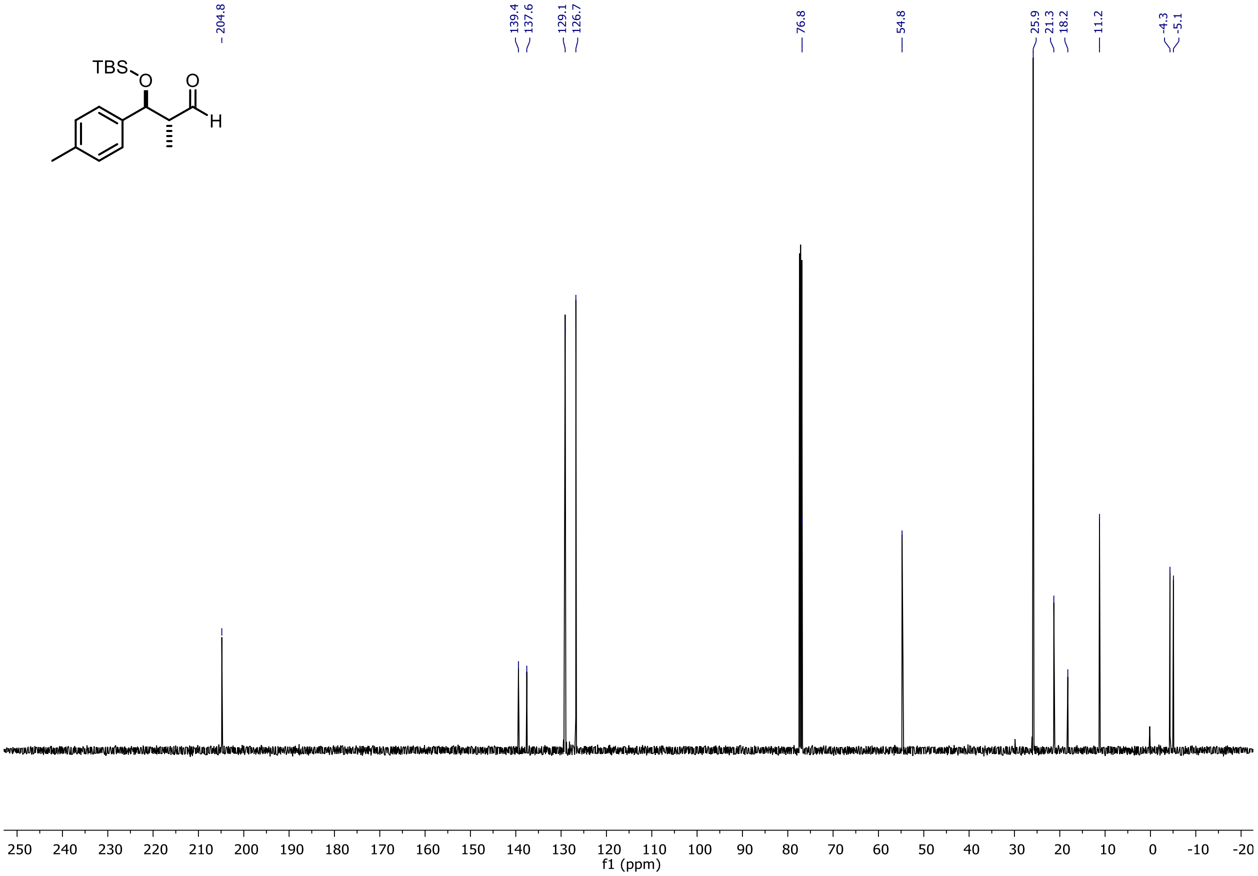

(1*S*,2*S*)-2-Methyl-1-(*p*-tolyl)propane-1,3-diol

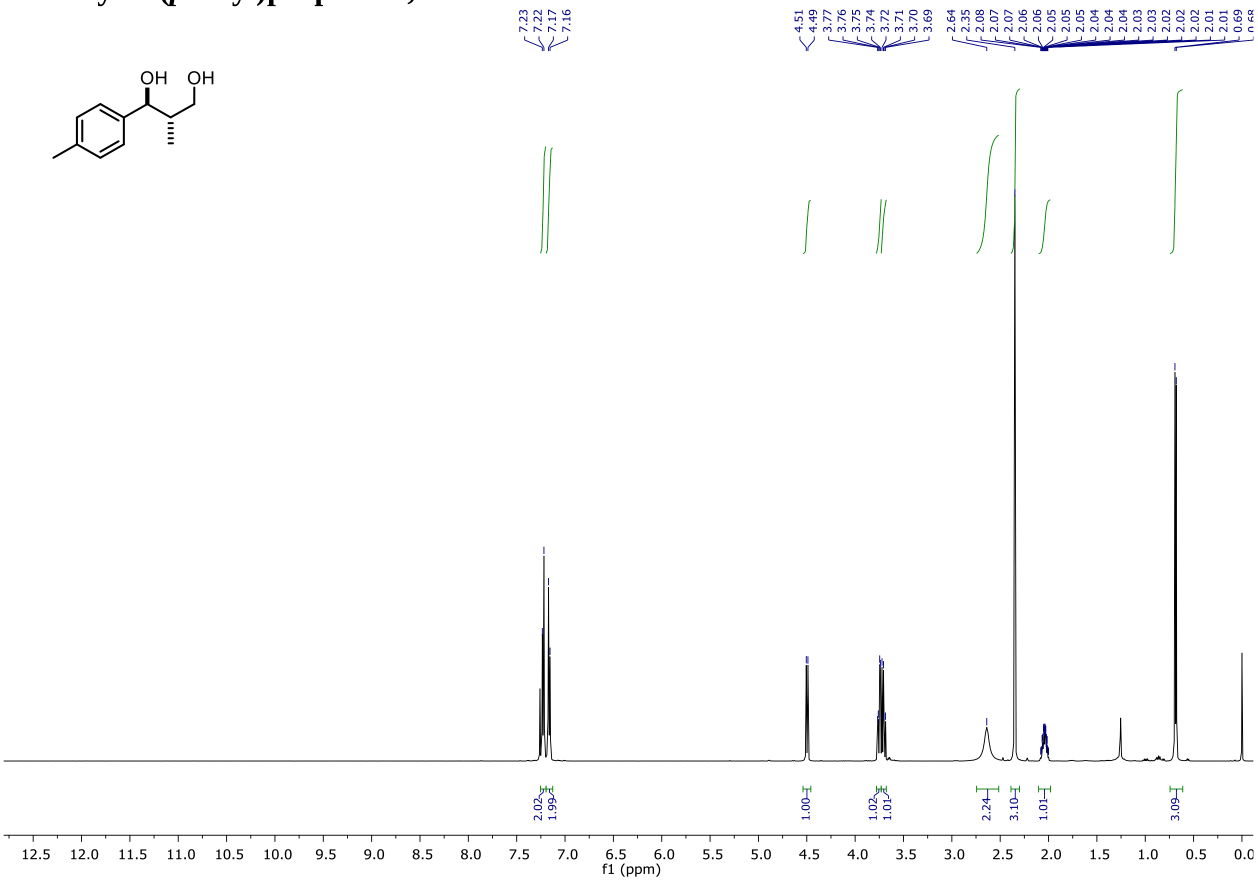

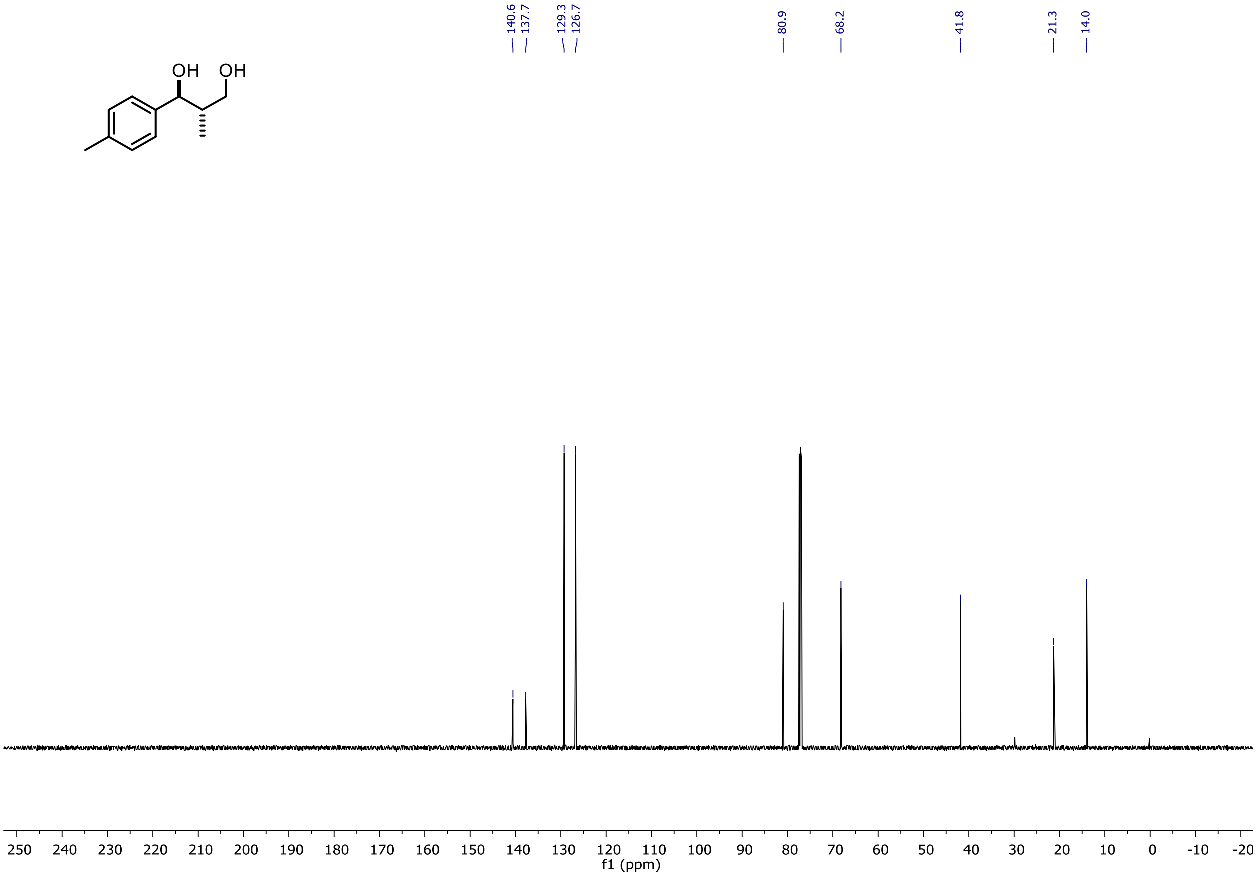

(2*R*,3*S*)-3-((*tert*-Butyldimethylsilyl)oxy)-3-(furan-2-yl)-2-methylpropanal

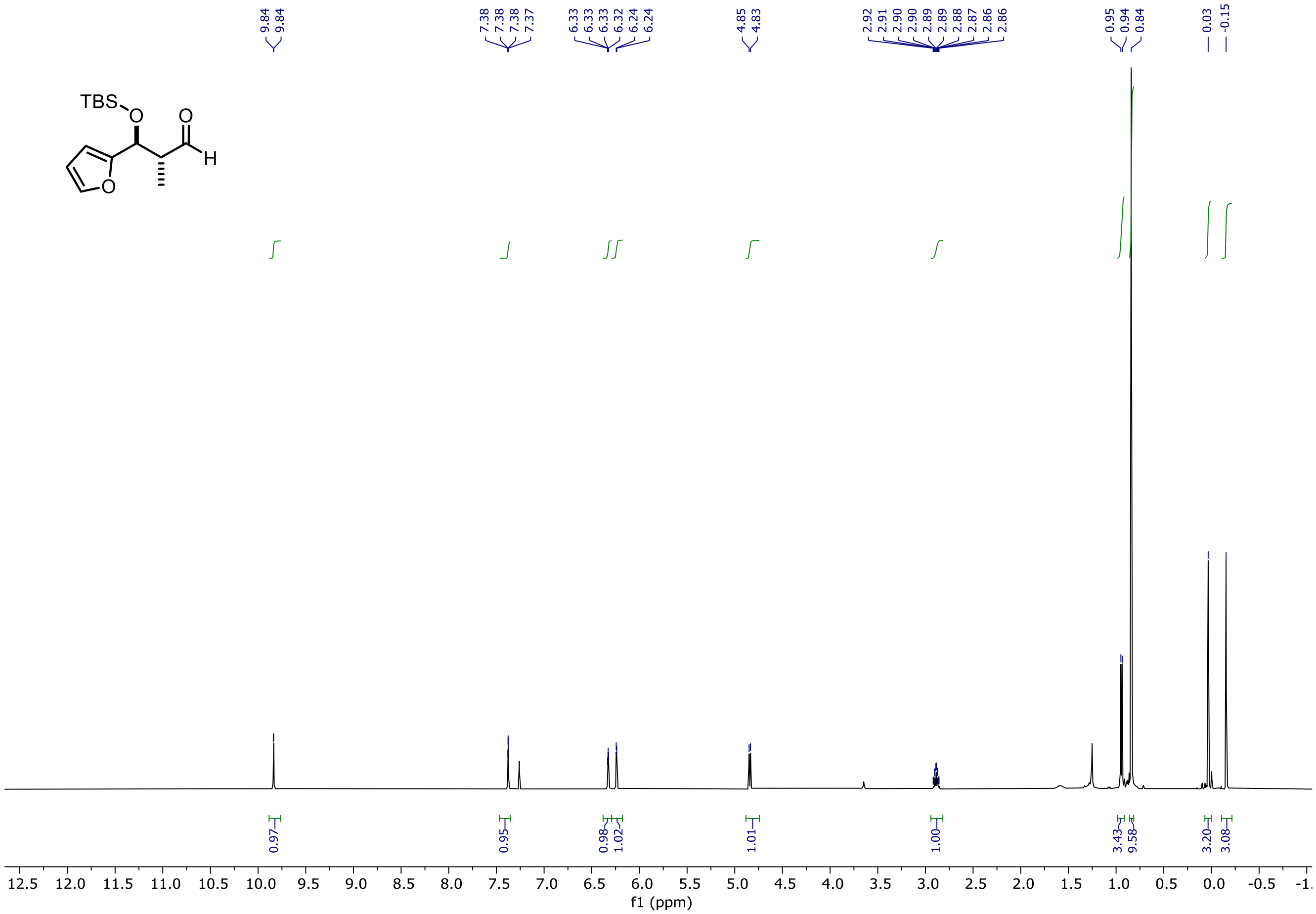

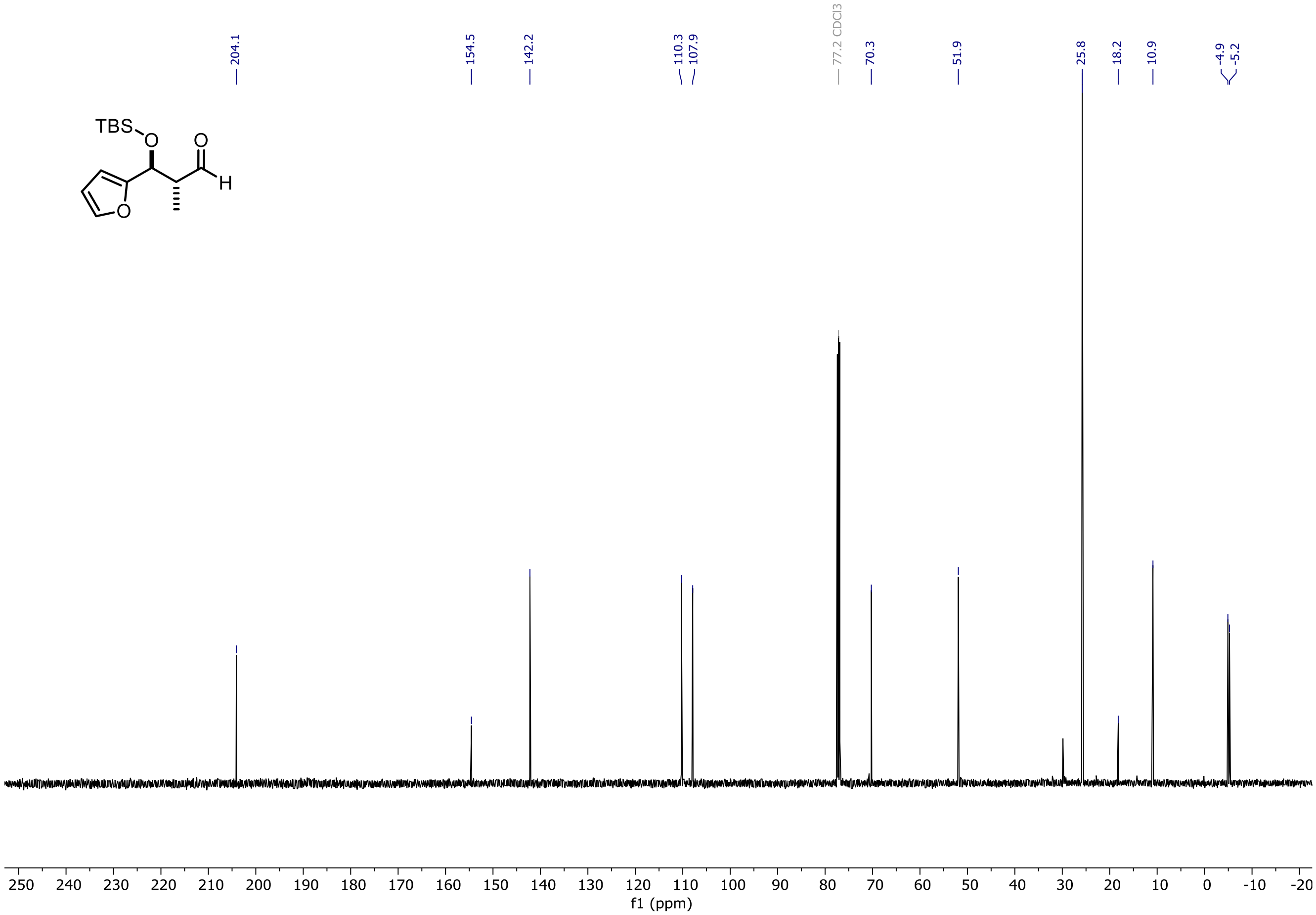

(1*S*,2*S*)-1-(Furan-2-yl)-2-methylpropane-1,3-diol

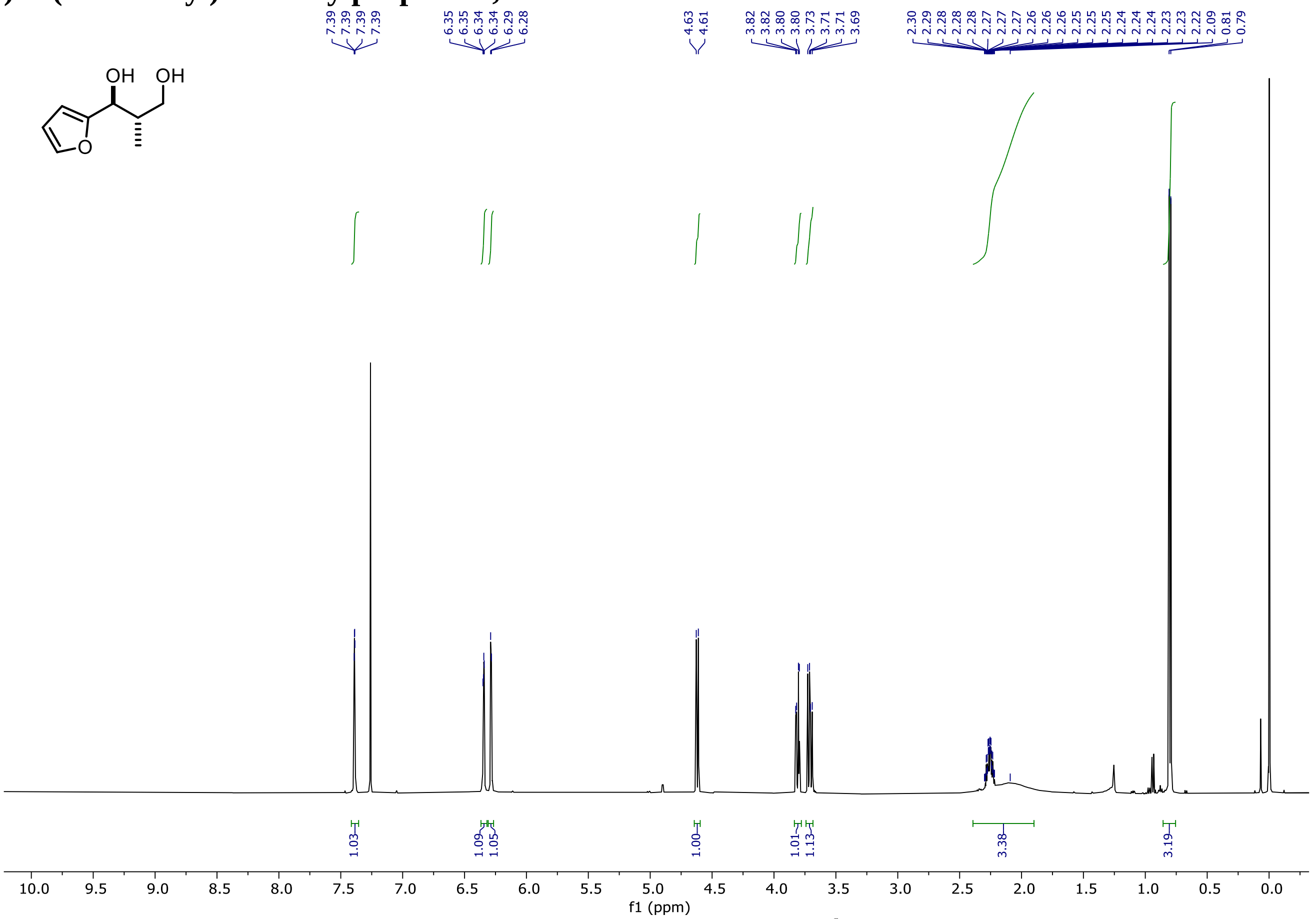

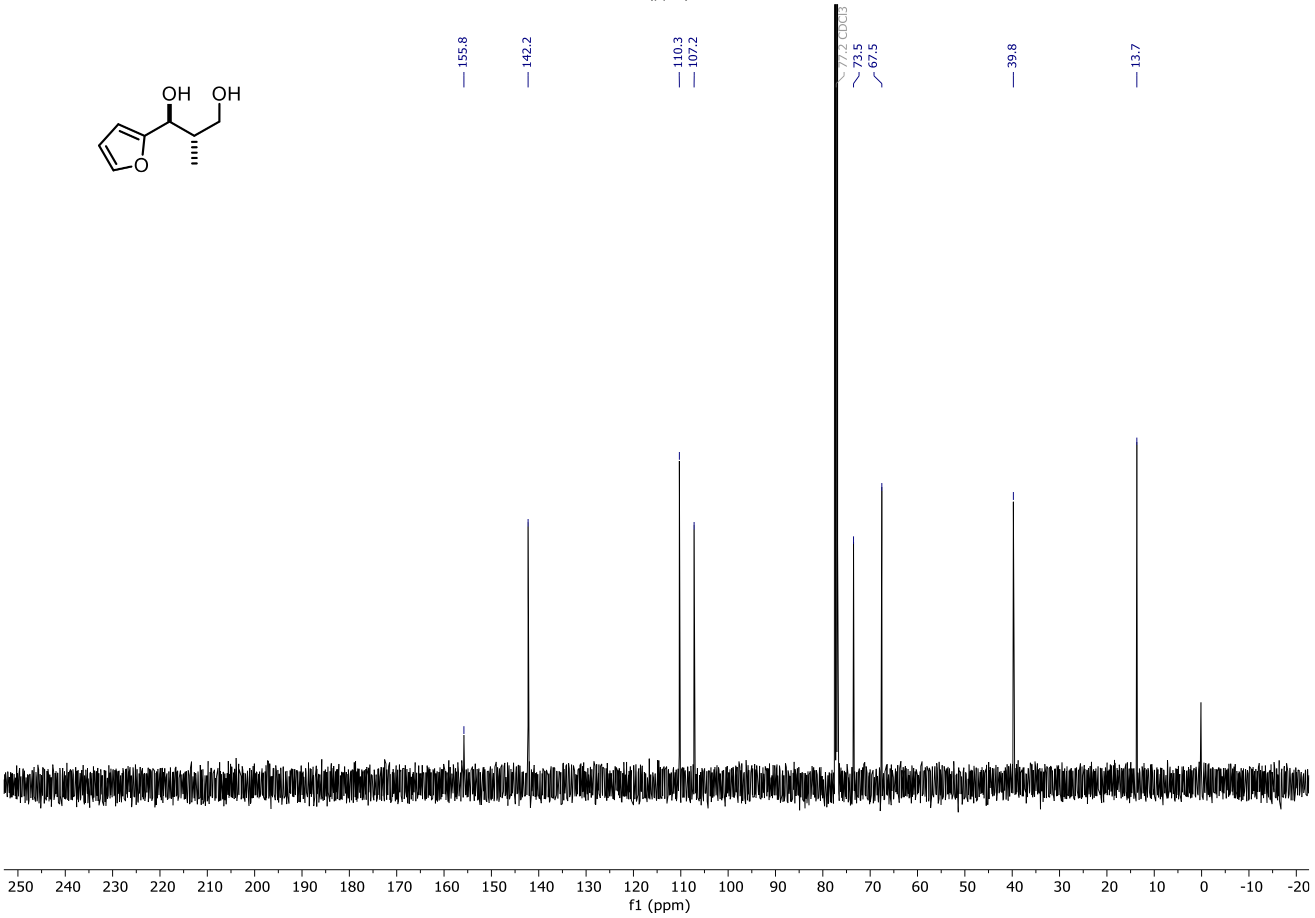

(2*R*,3*S*)-3-((*tert*-Butyldimethylsilyl)oxy)-2-methyl-3-(thiophen-2-yl)propanal

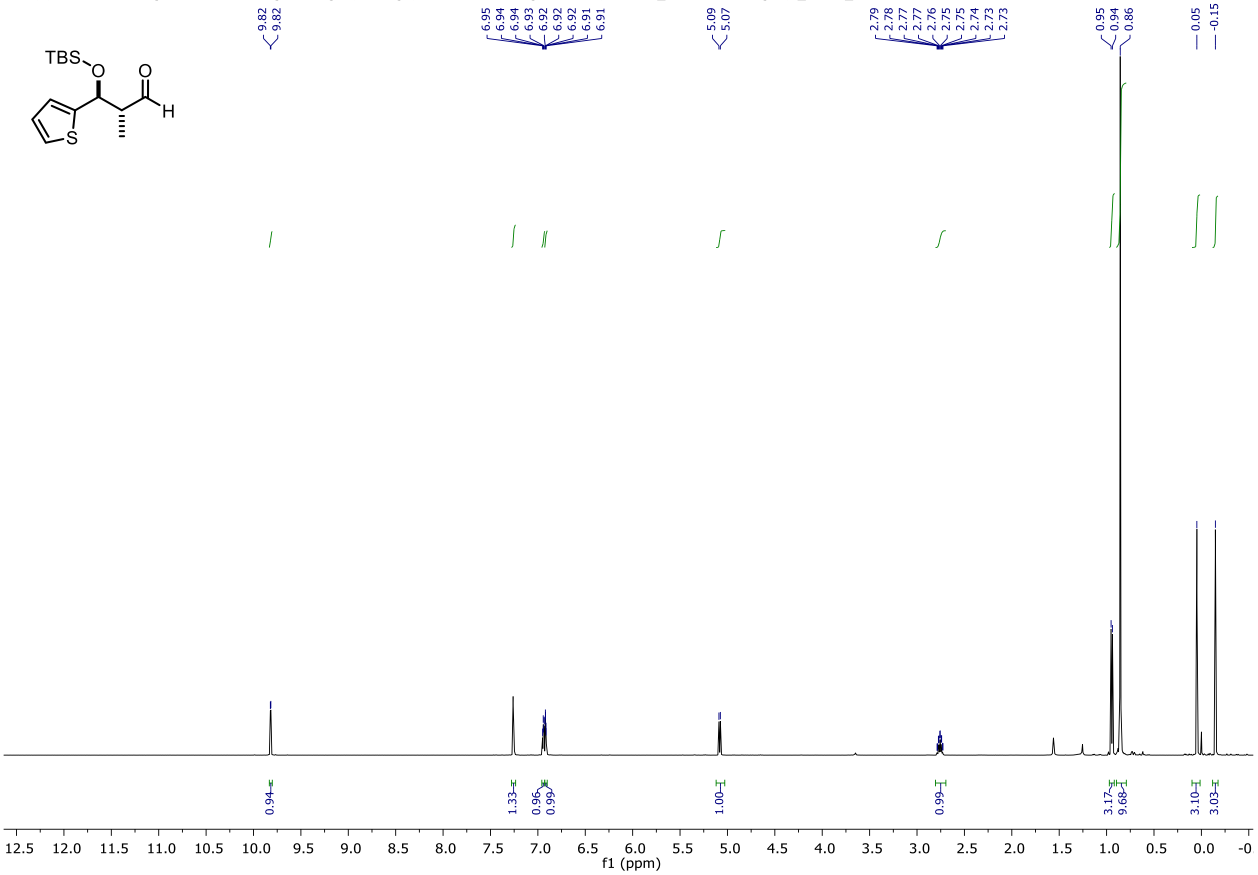

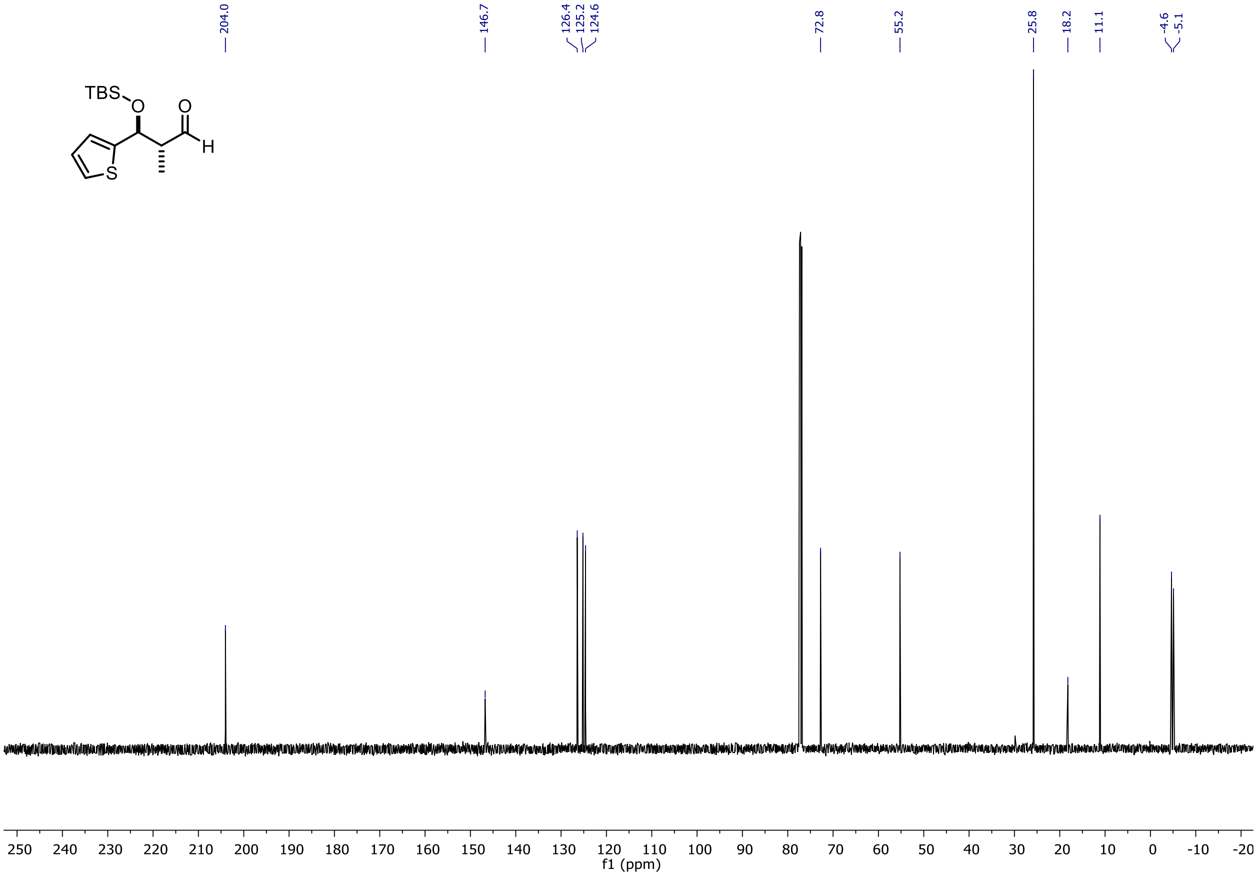

(1*S*,2*S*)-2-Methyl-1-(thiophen-2-yl)propane-1,3-diol

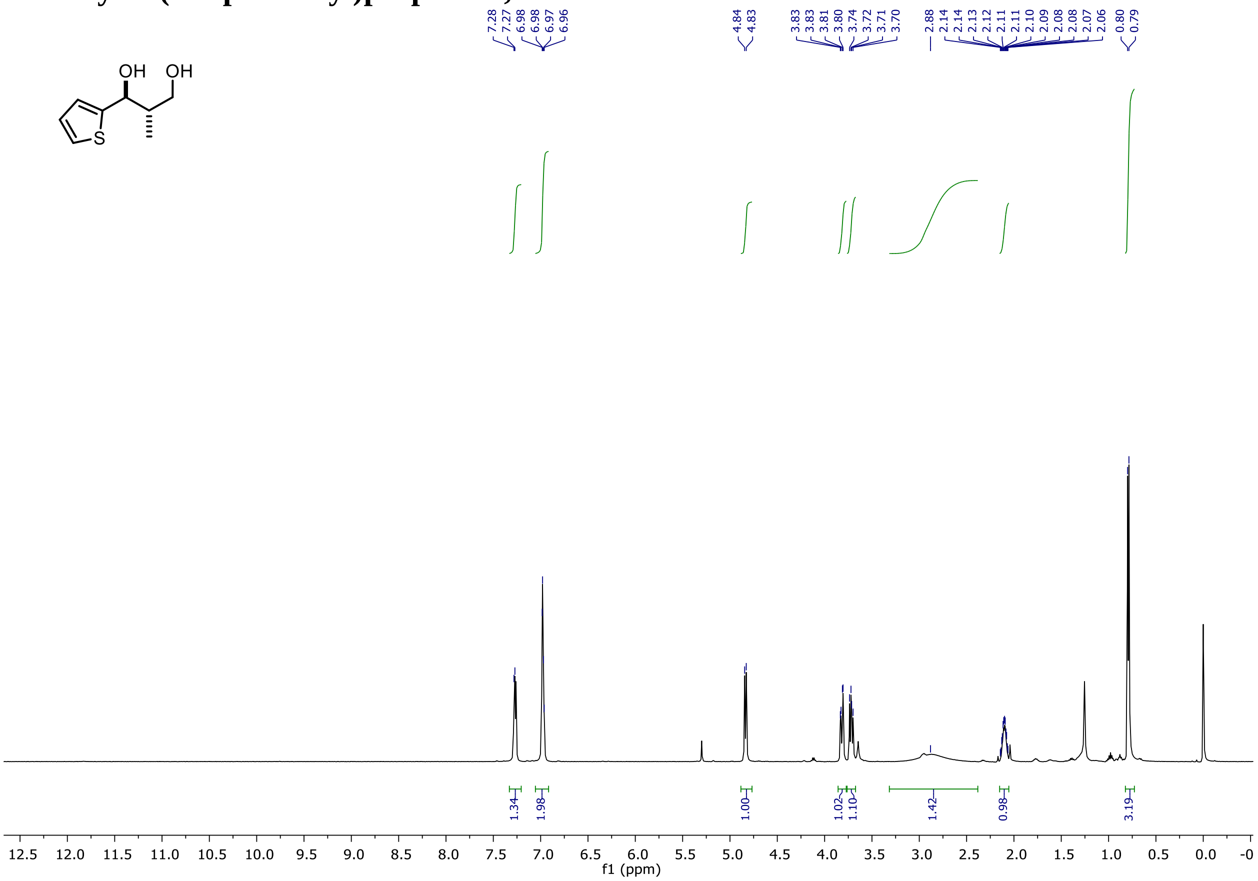

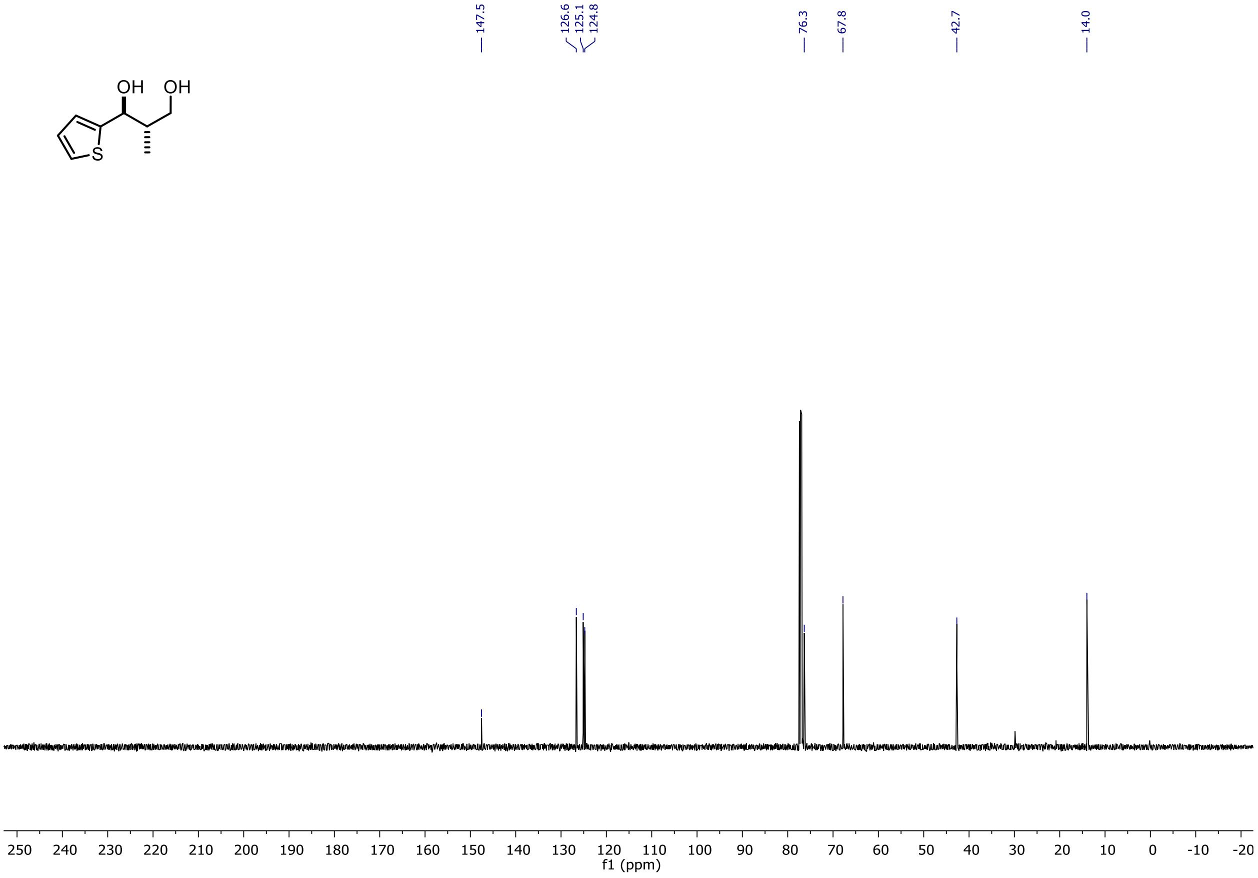

(2*R*,3*S*)-3-(4-Bromophenyl)-3-((*tert*-butyldimethylsilyl)oxy)-2-methylpropanal

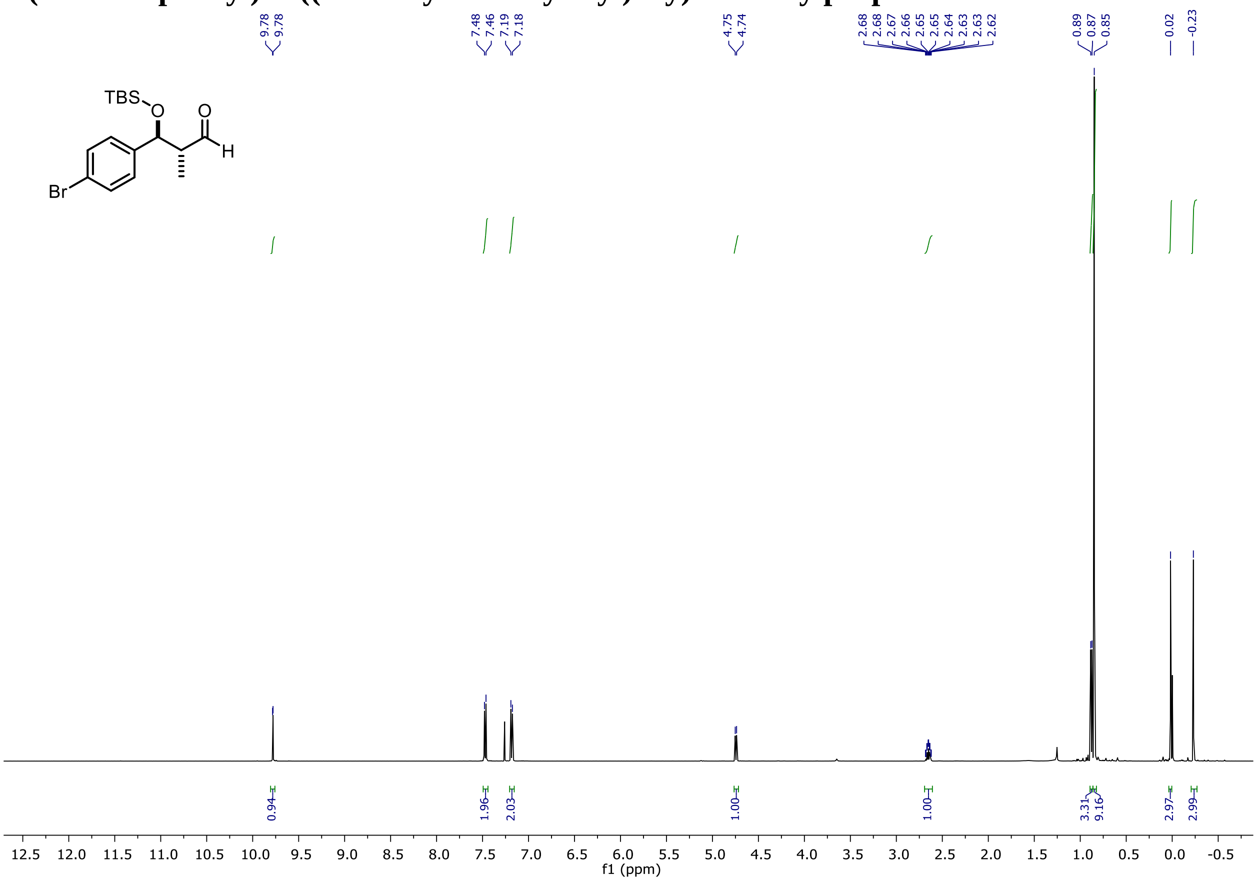

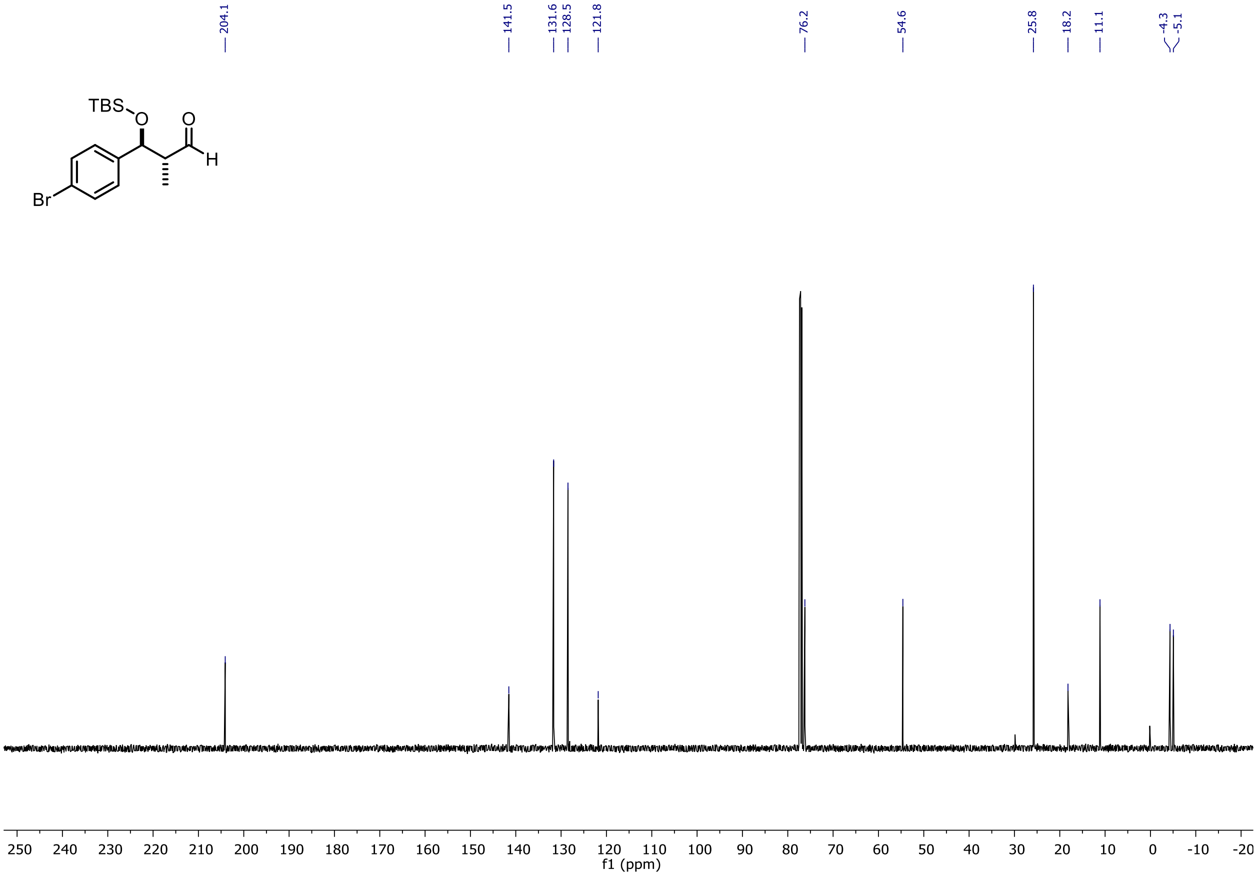

(1*S*,2*S*)-1-(4-Bromophenyl)-2-methylpropane-1,3-diol

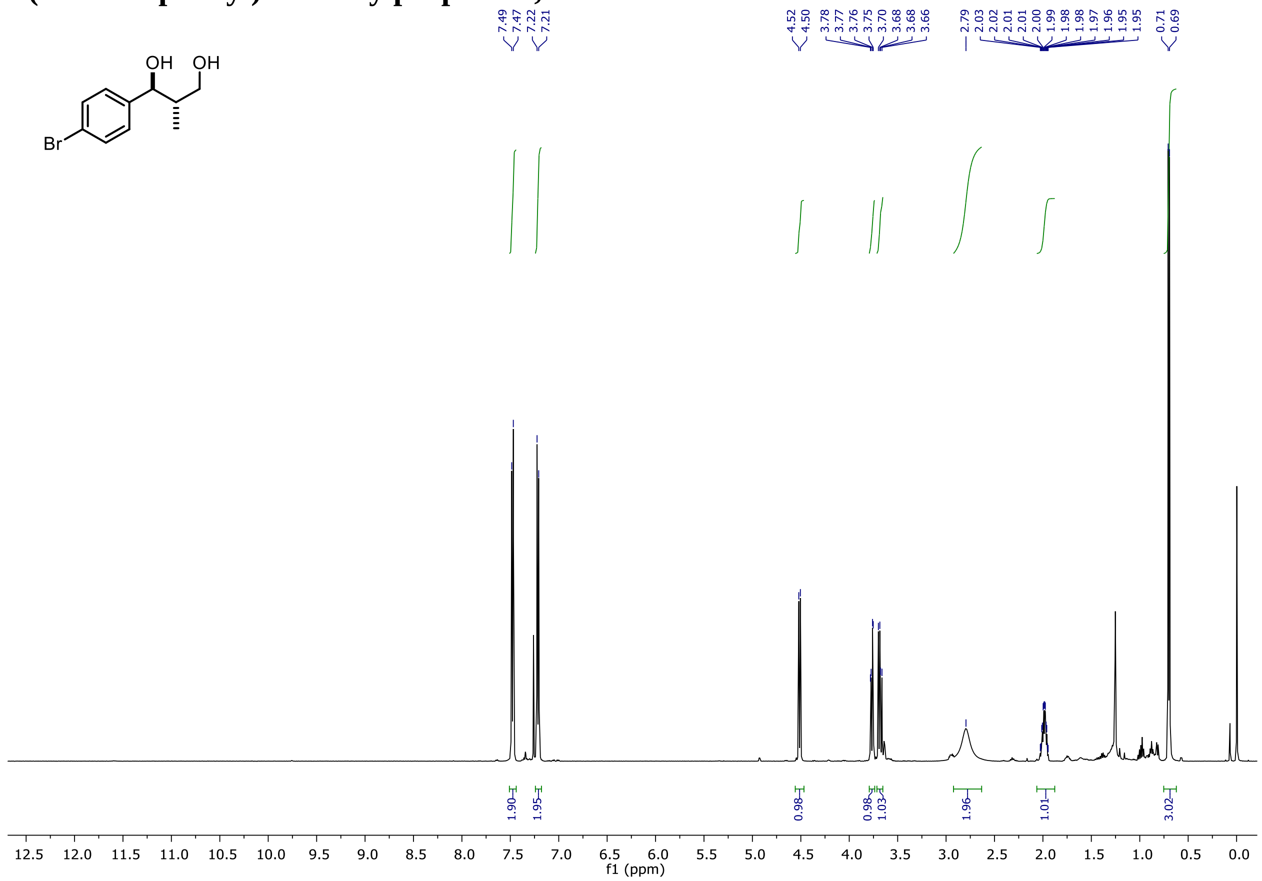

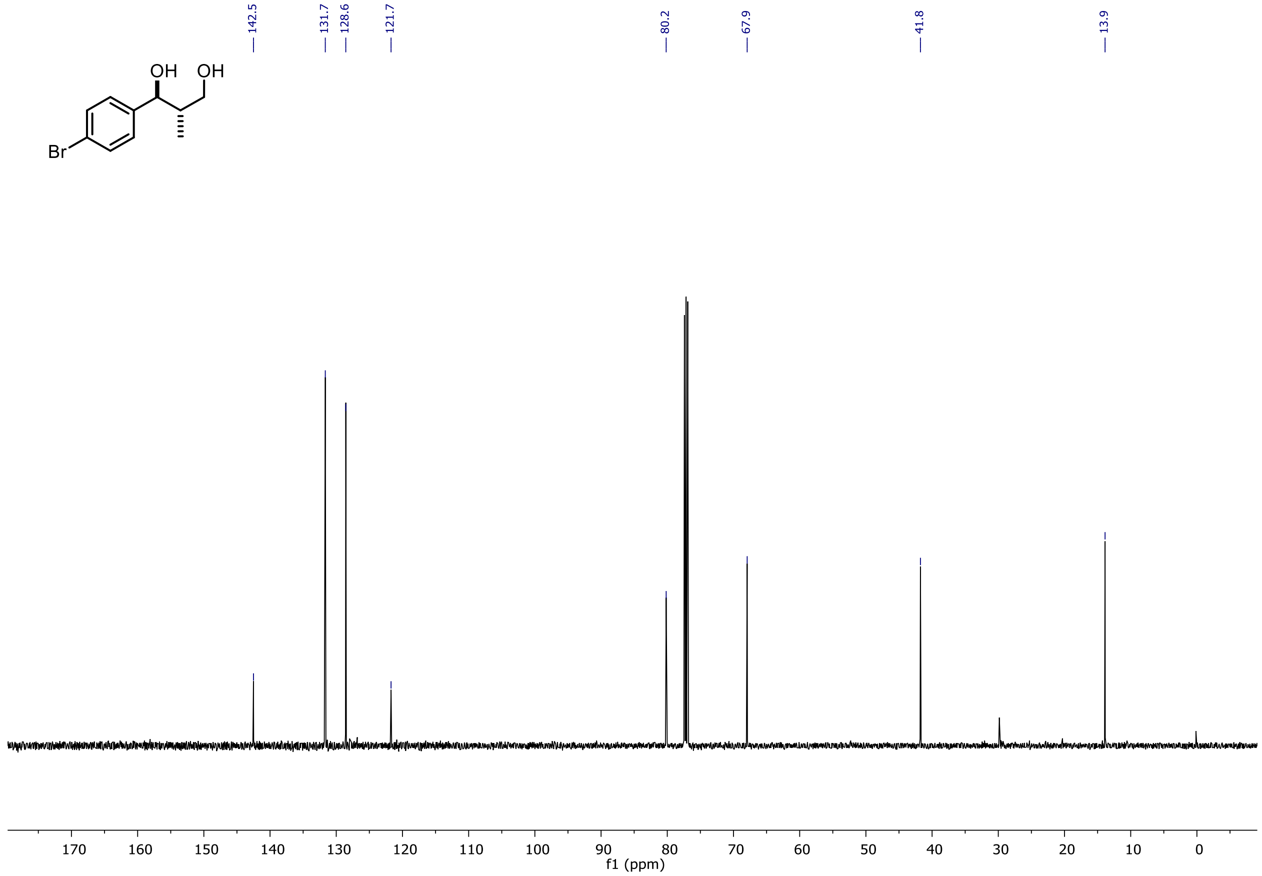

(2*R*,3*S*)-3-((*tert*-Butyldimethylsilyl)oxy)-3-(3,5-dimethoxyphenyl)-2-methylpropanal

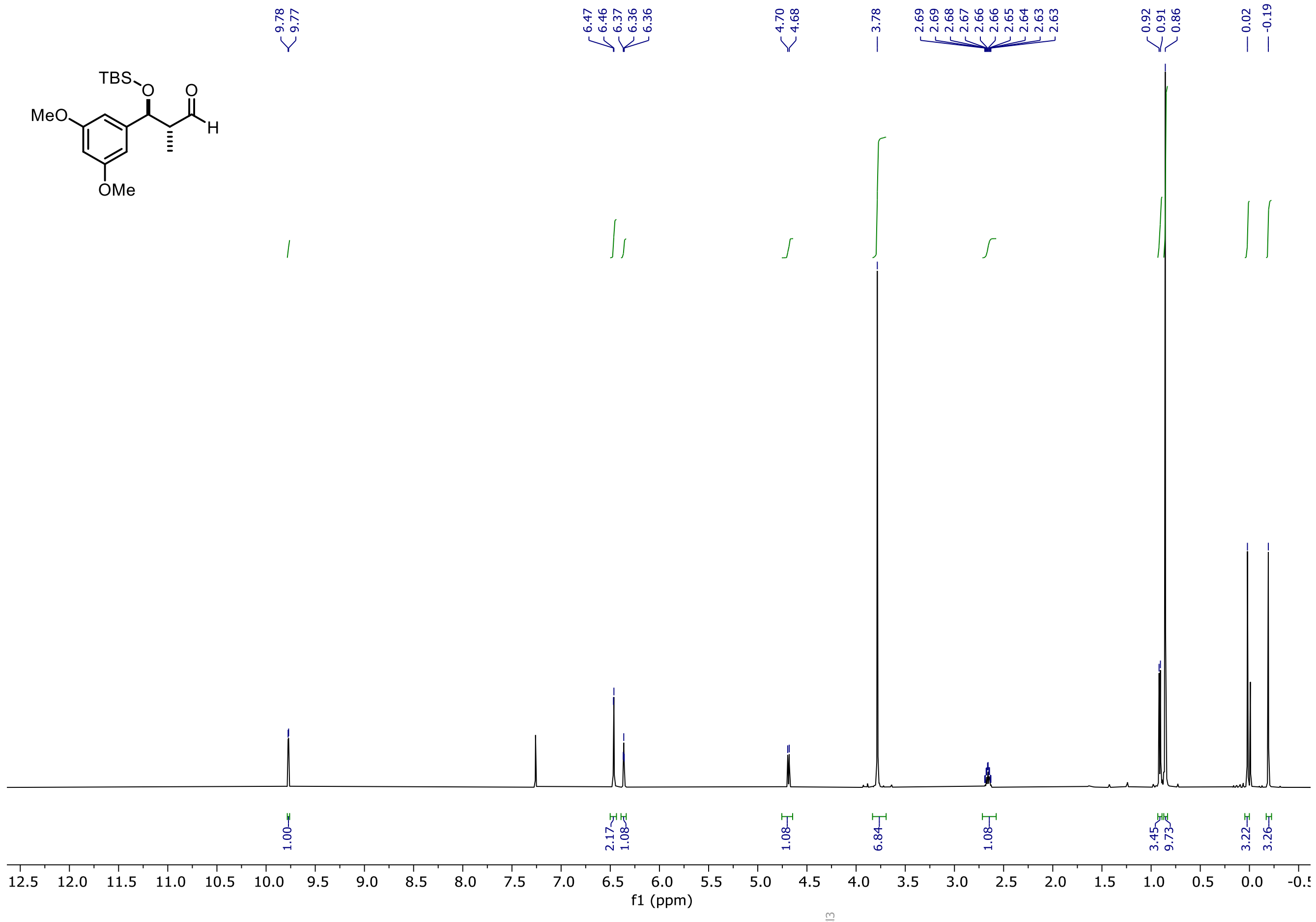

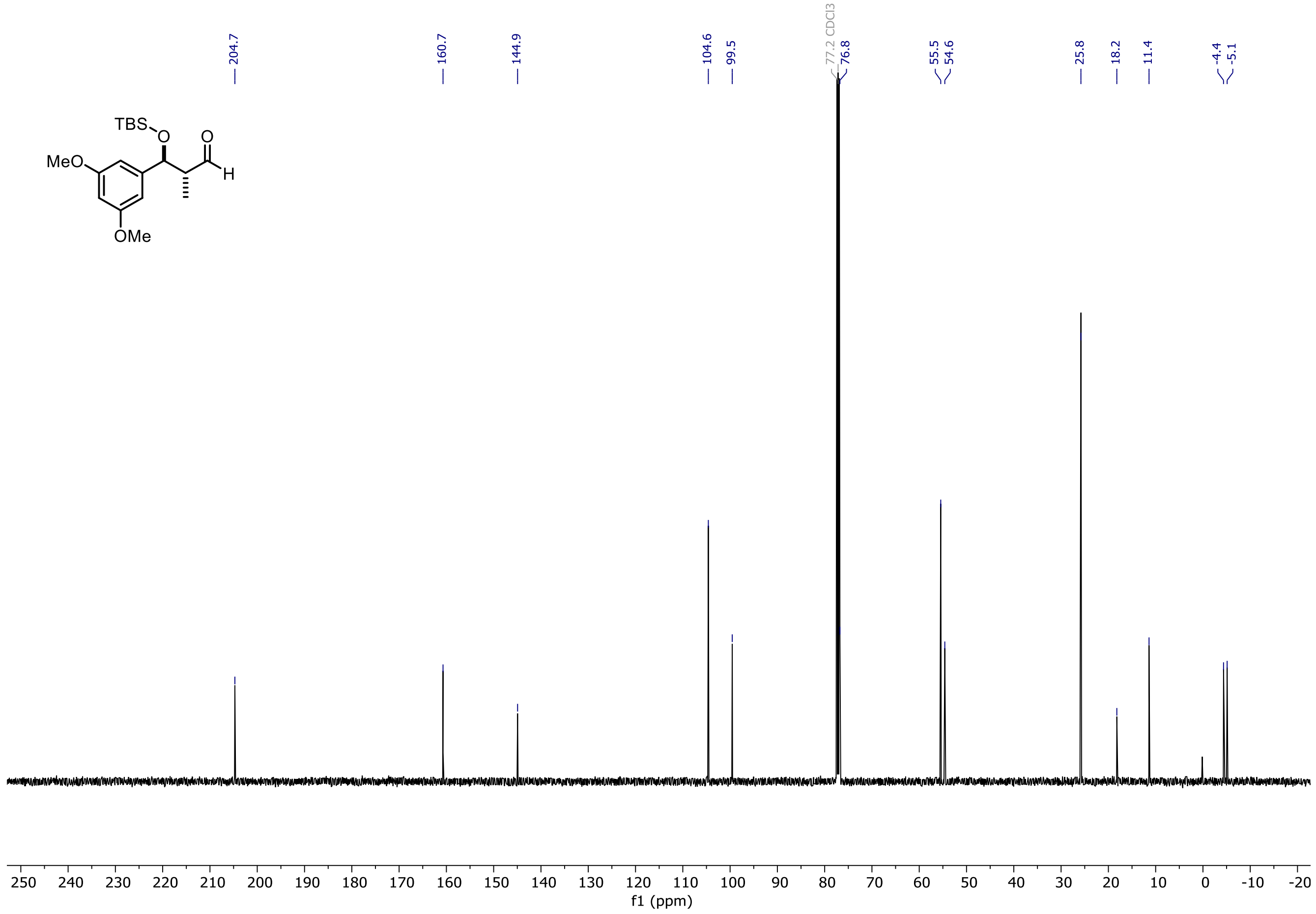

(1*S*,2*S*)-1-(3,5-Dimethoxyphenyl)-2-methylpropane-1,3-diol

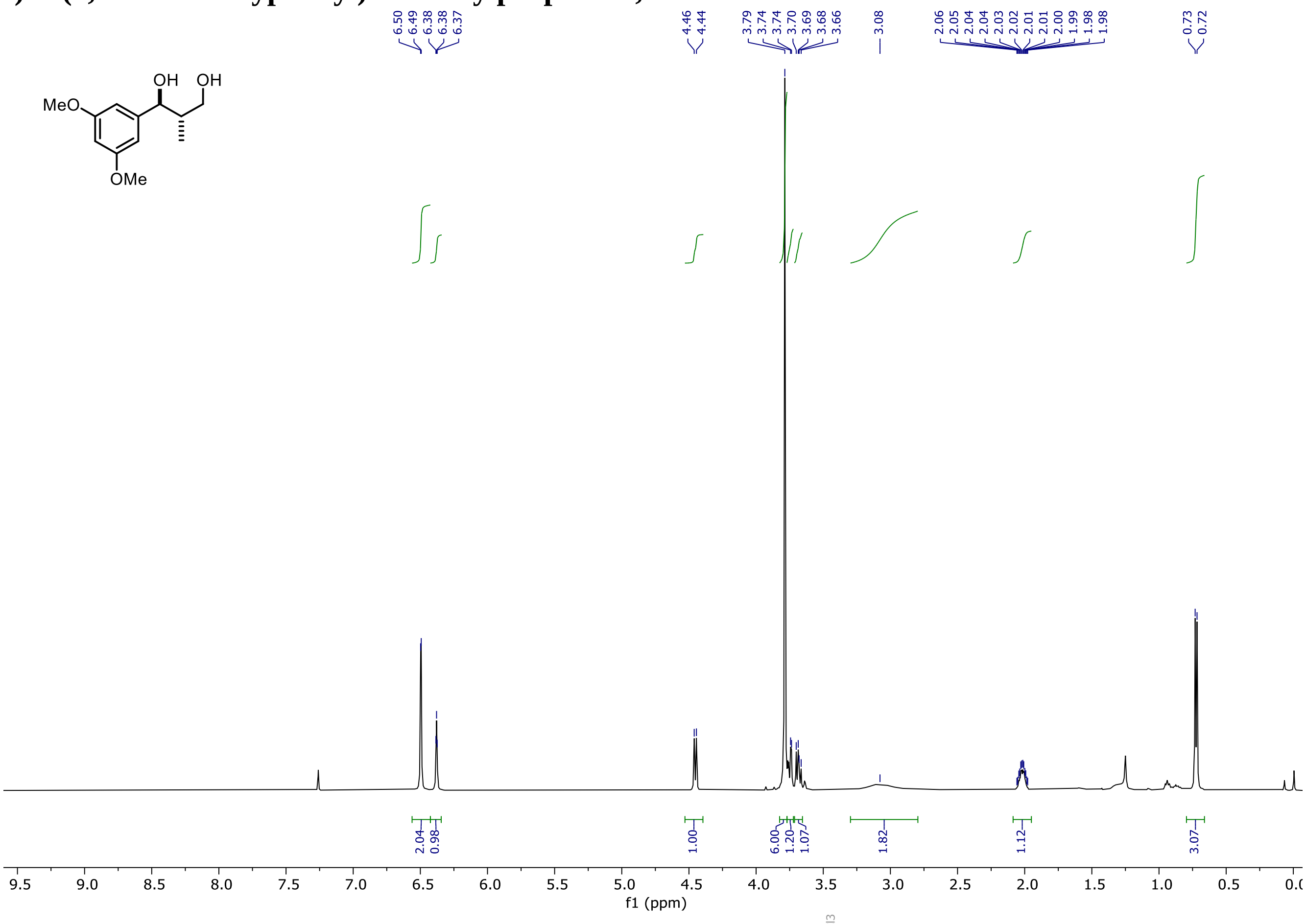

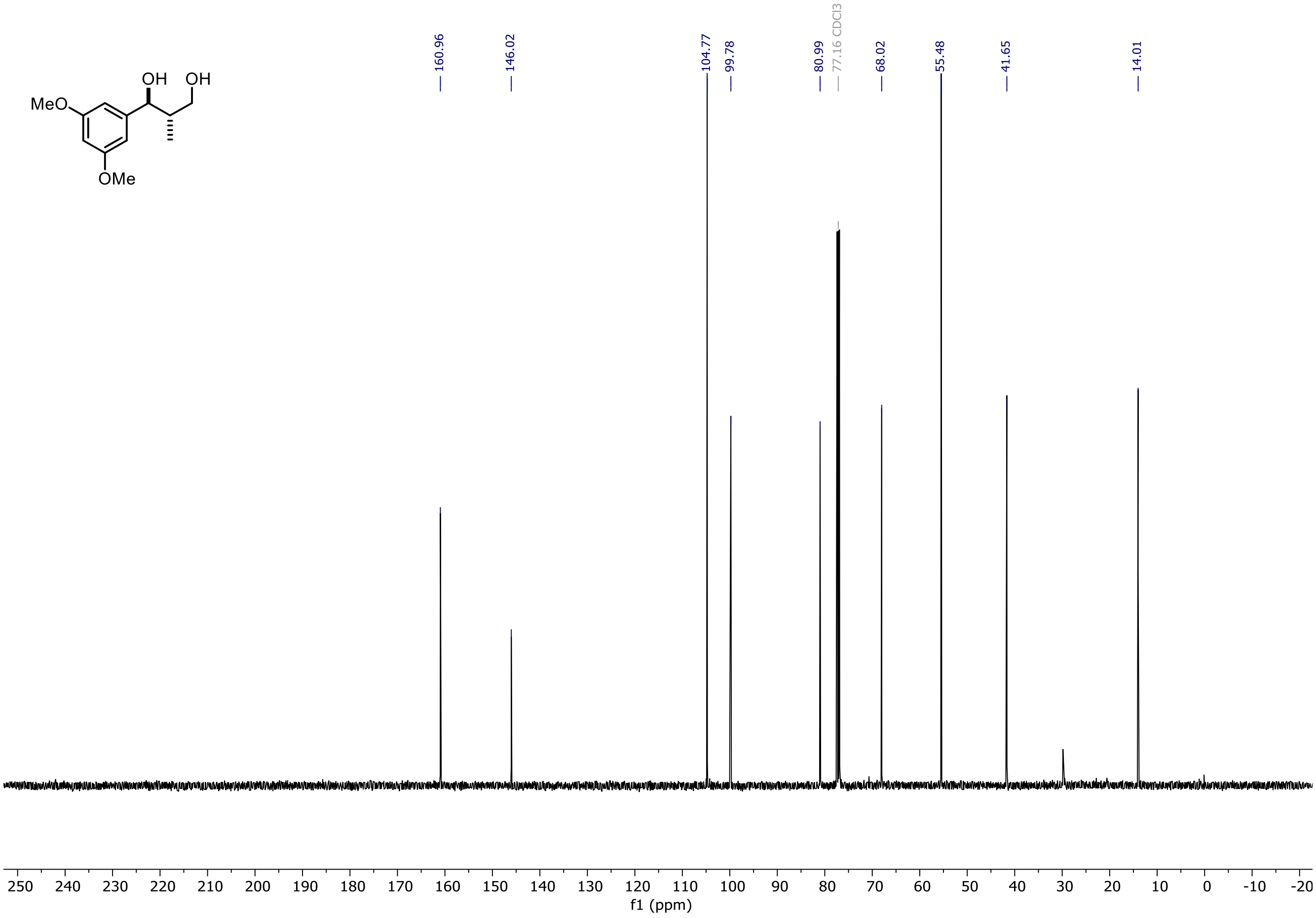

(2*R*,3*S*)-3-((*tert*-Butyldimethylsilyl)oxy)-3-(2,5-dimethoxy-3-nitrophenyl)-2-methylpropanal

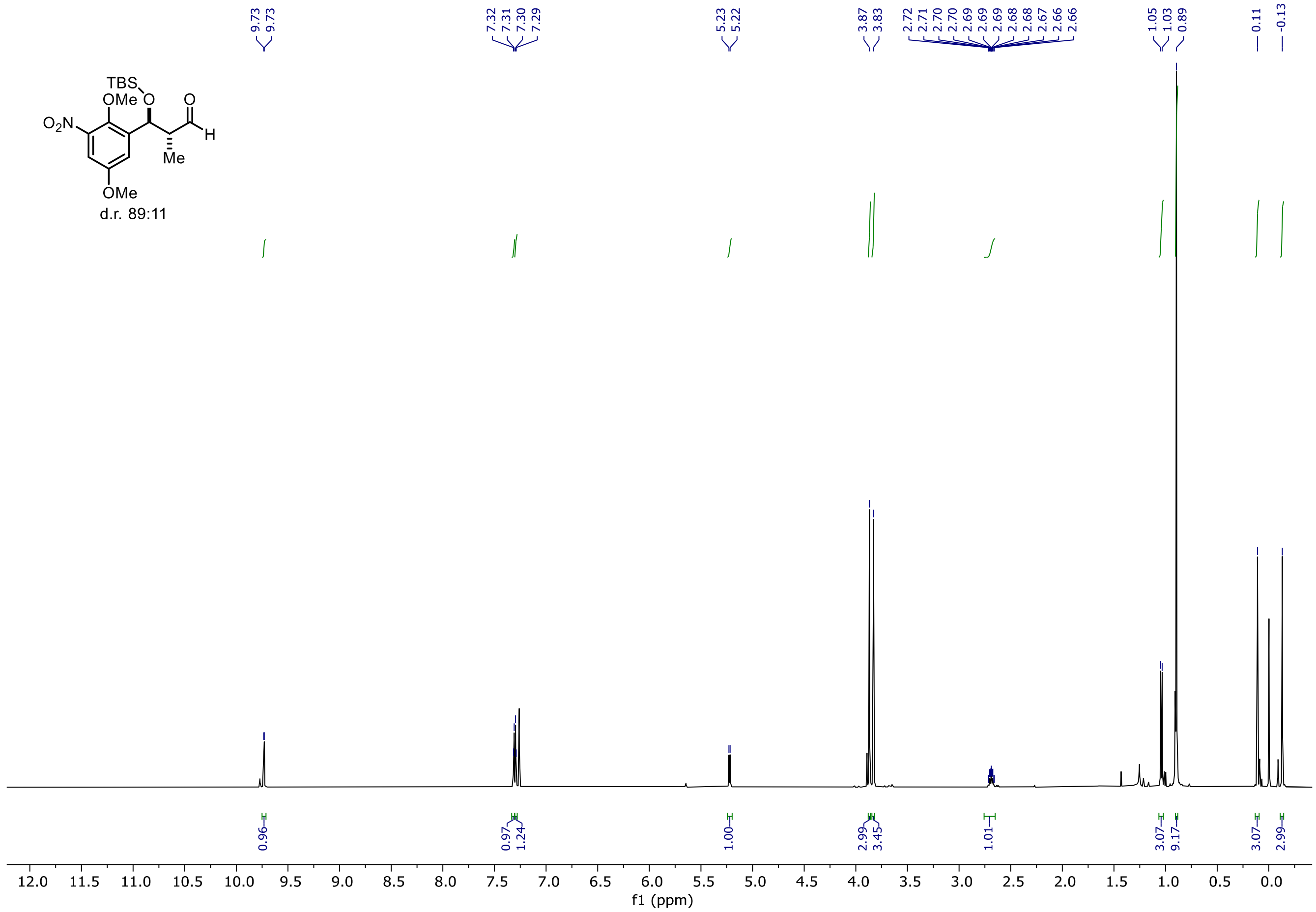

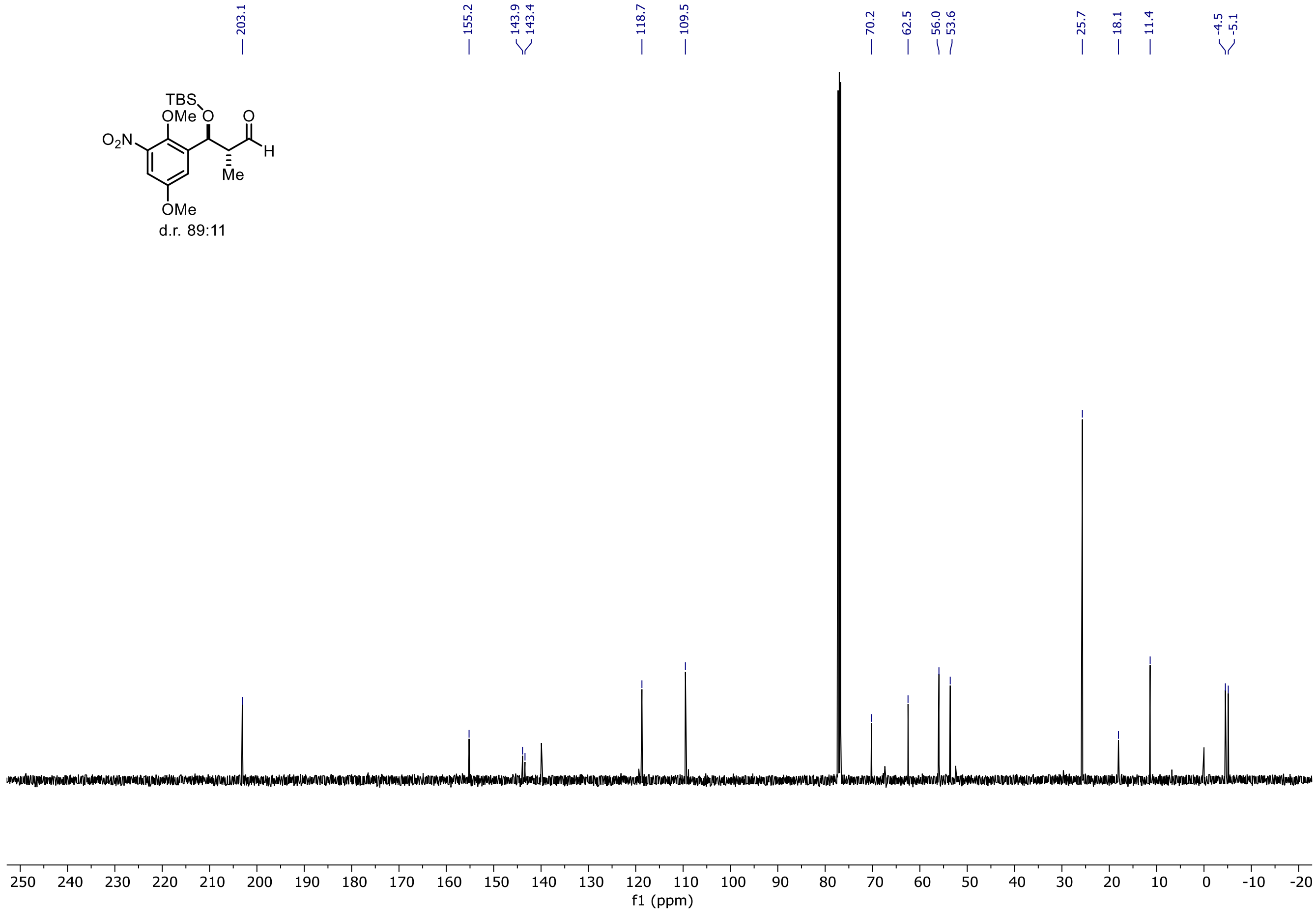

(1*S*,2*S*)-1-(2,5-Dimethoxy-3-nitrophenyl)-2-methylpropane-1,3-diol

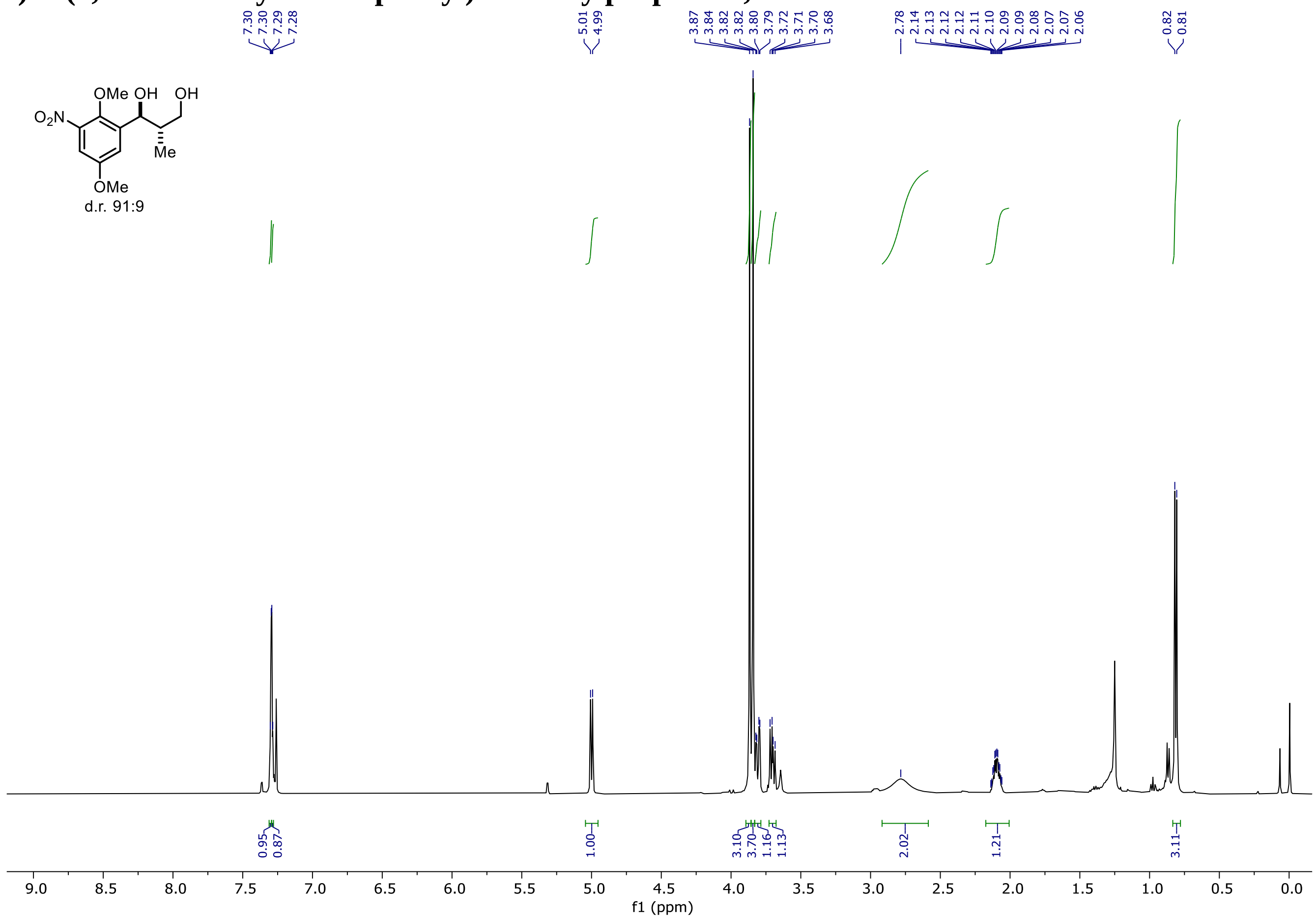

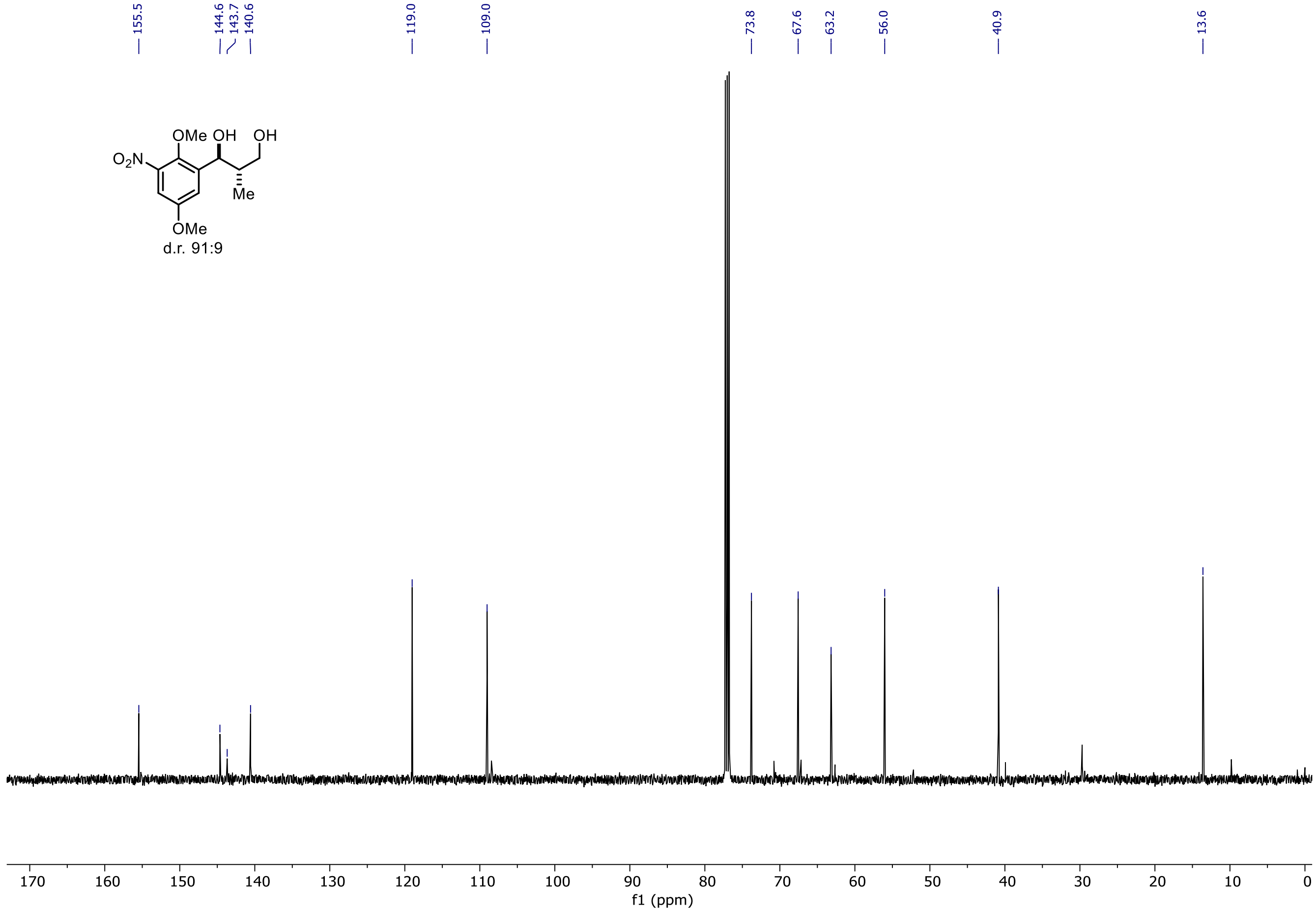

(2*R*,3*S*)-3-(Benzo[d][1,3]dioxol-5-yl)-3-((*tert*-butyldimethylsilyl)oxy)-2-methylpropanal

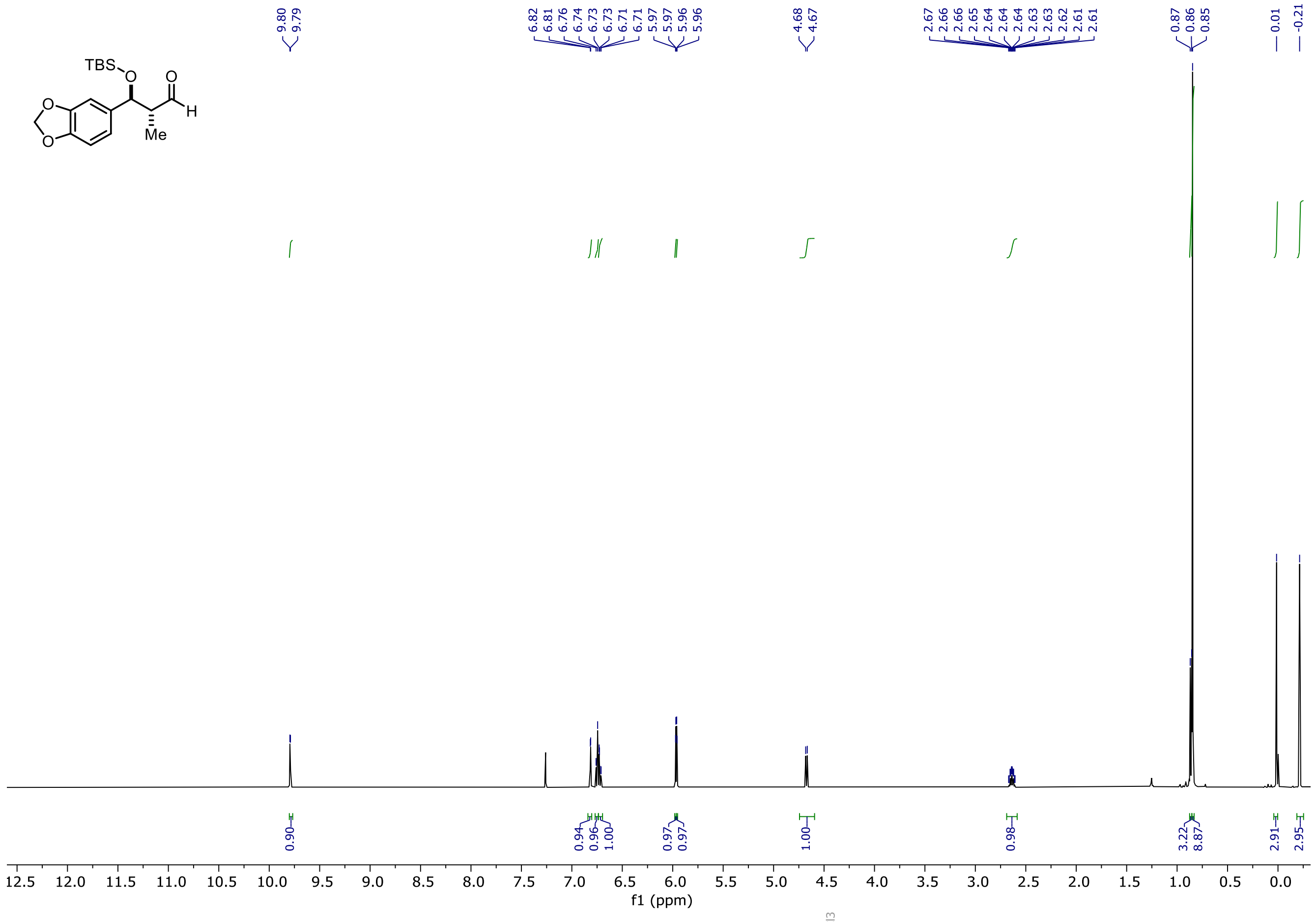

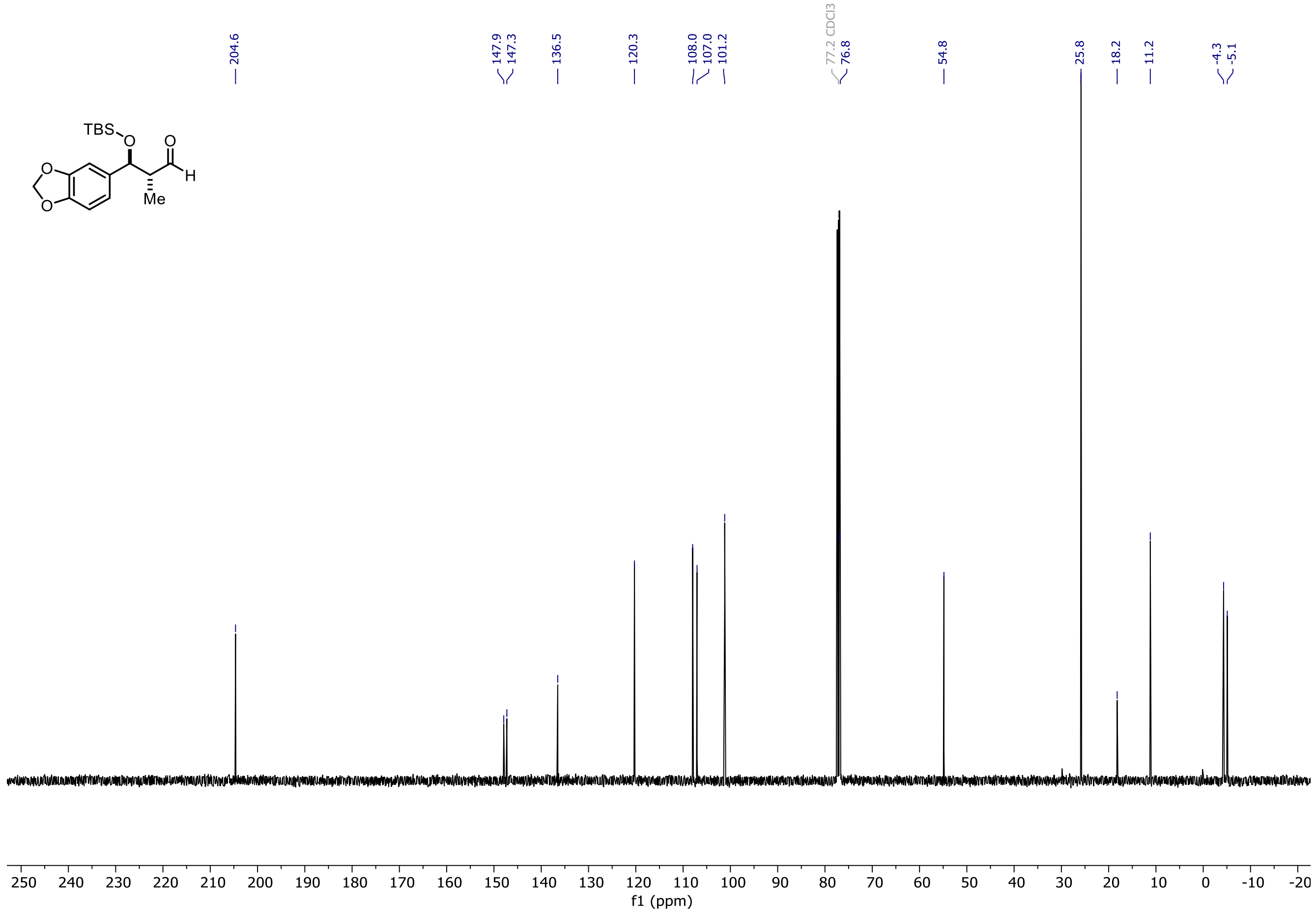

(1*S*,2*S*)-1-(Benzo[d][1,3]dioxol-5-yl)-2-methylpropane-1,3-diol

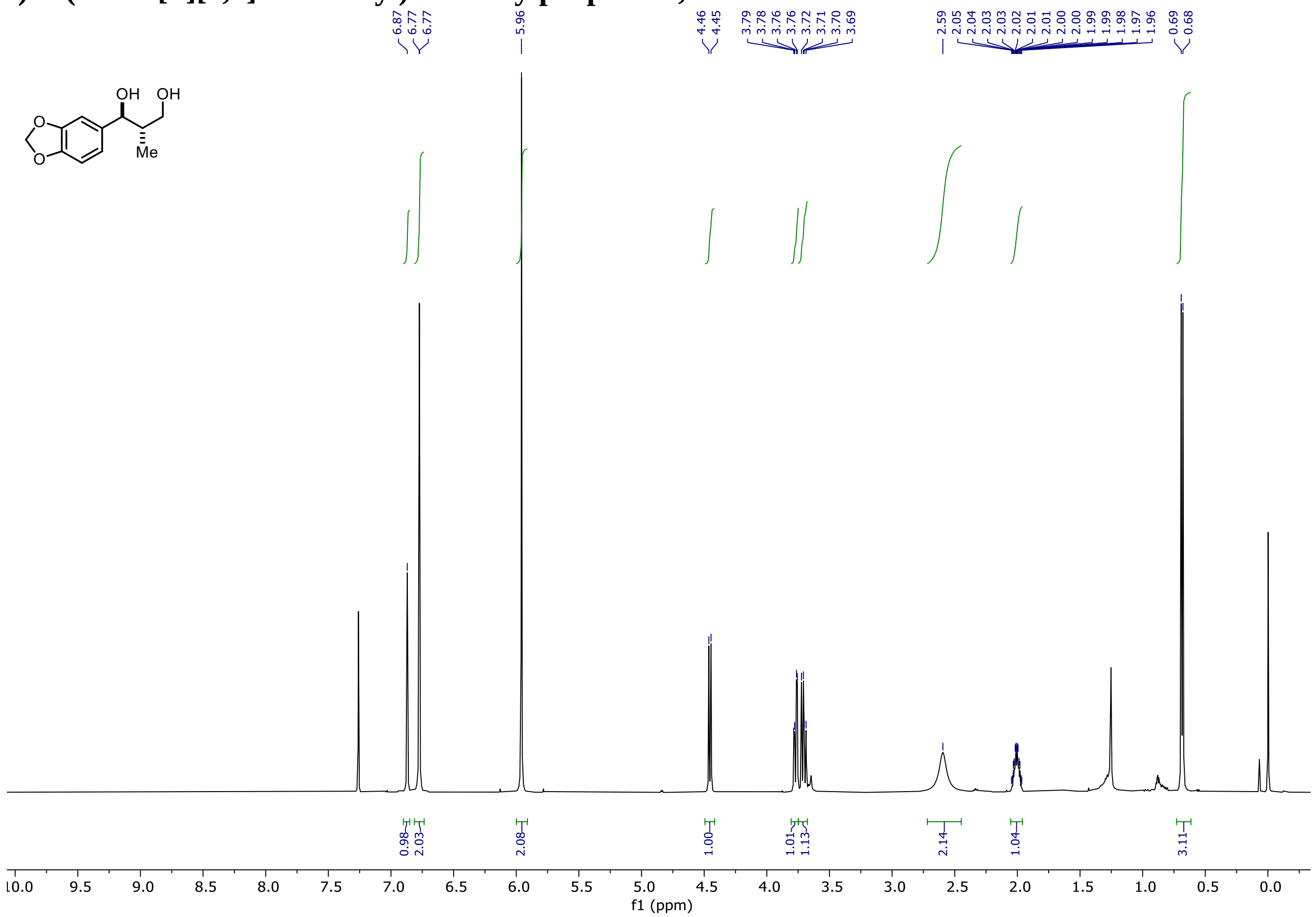

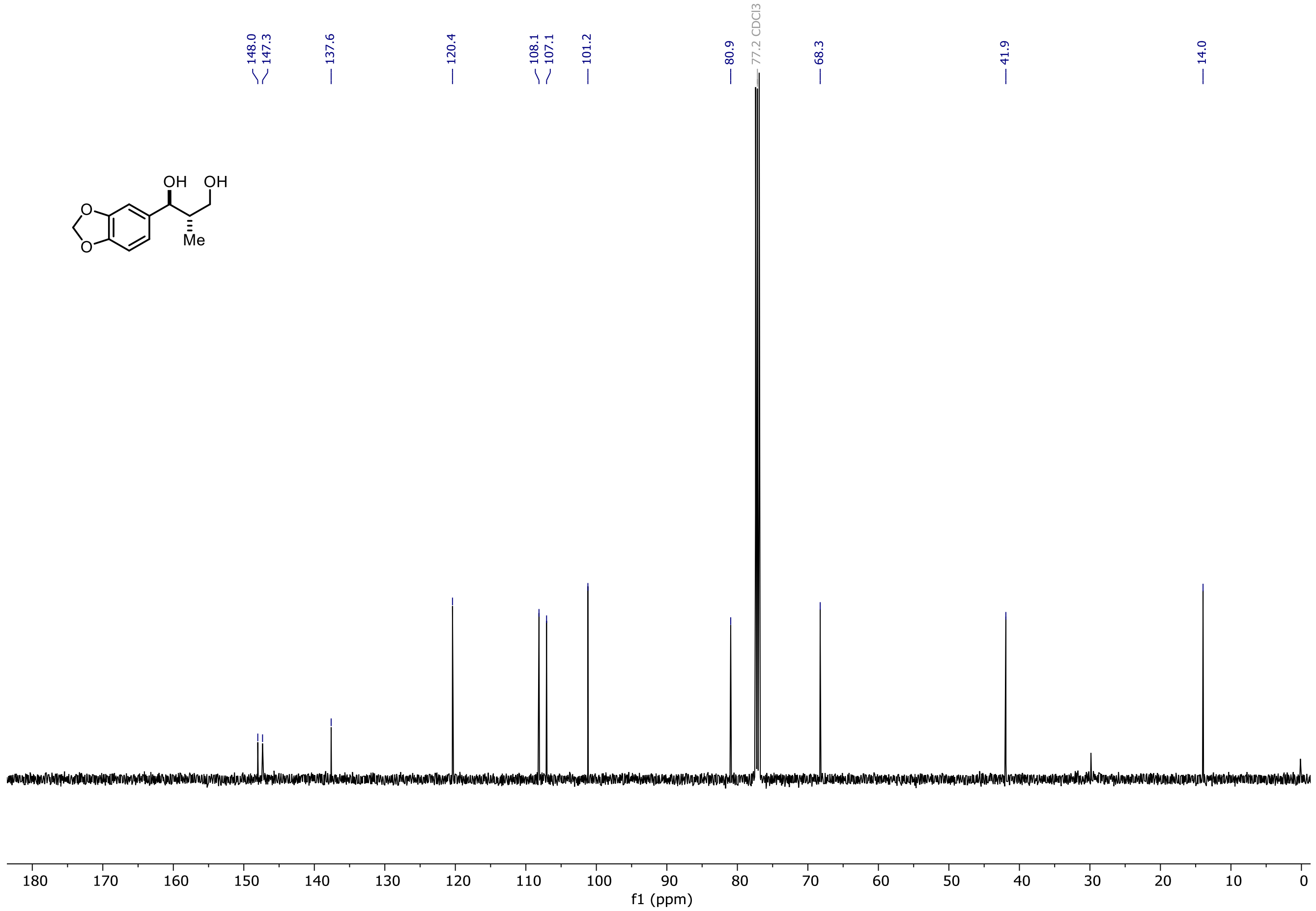

(*R*)-2-((*S*)-Phenyl((triethylsilyl)oxy)methyl)butanal

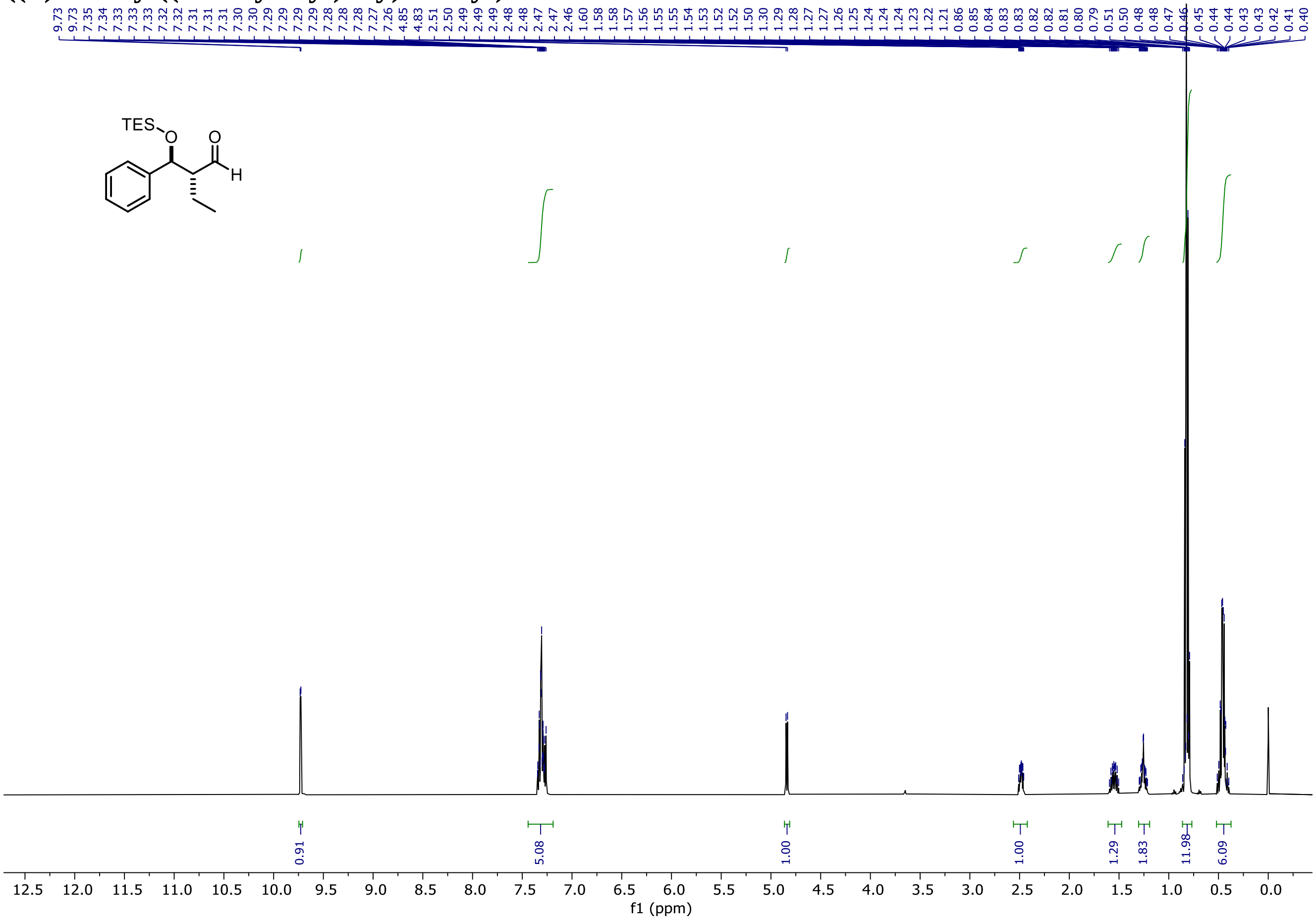

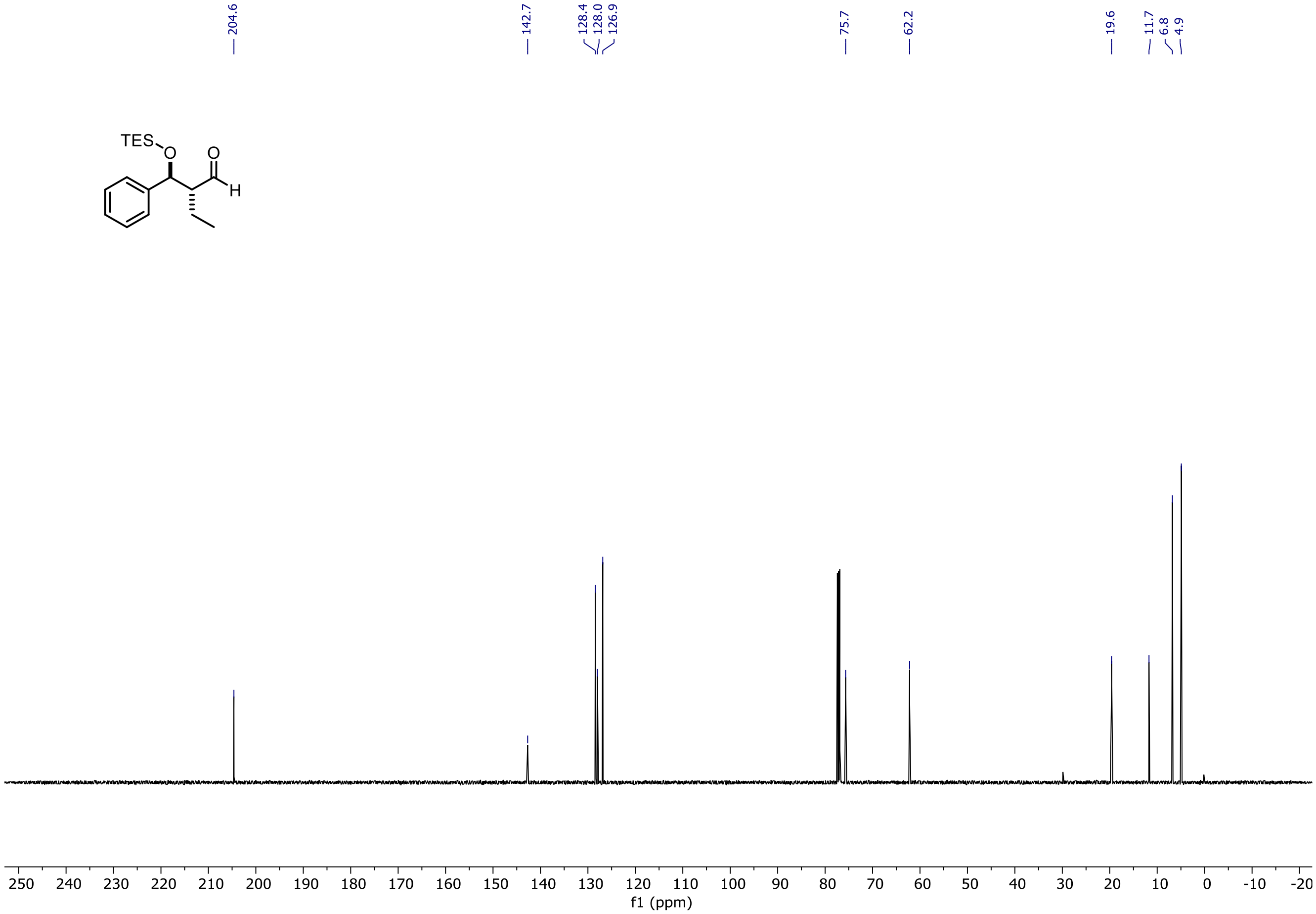

(1*S*,2*S*)-2-Ethyl-1-phenylpropane-1,3-diol

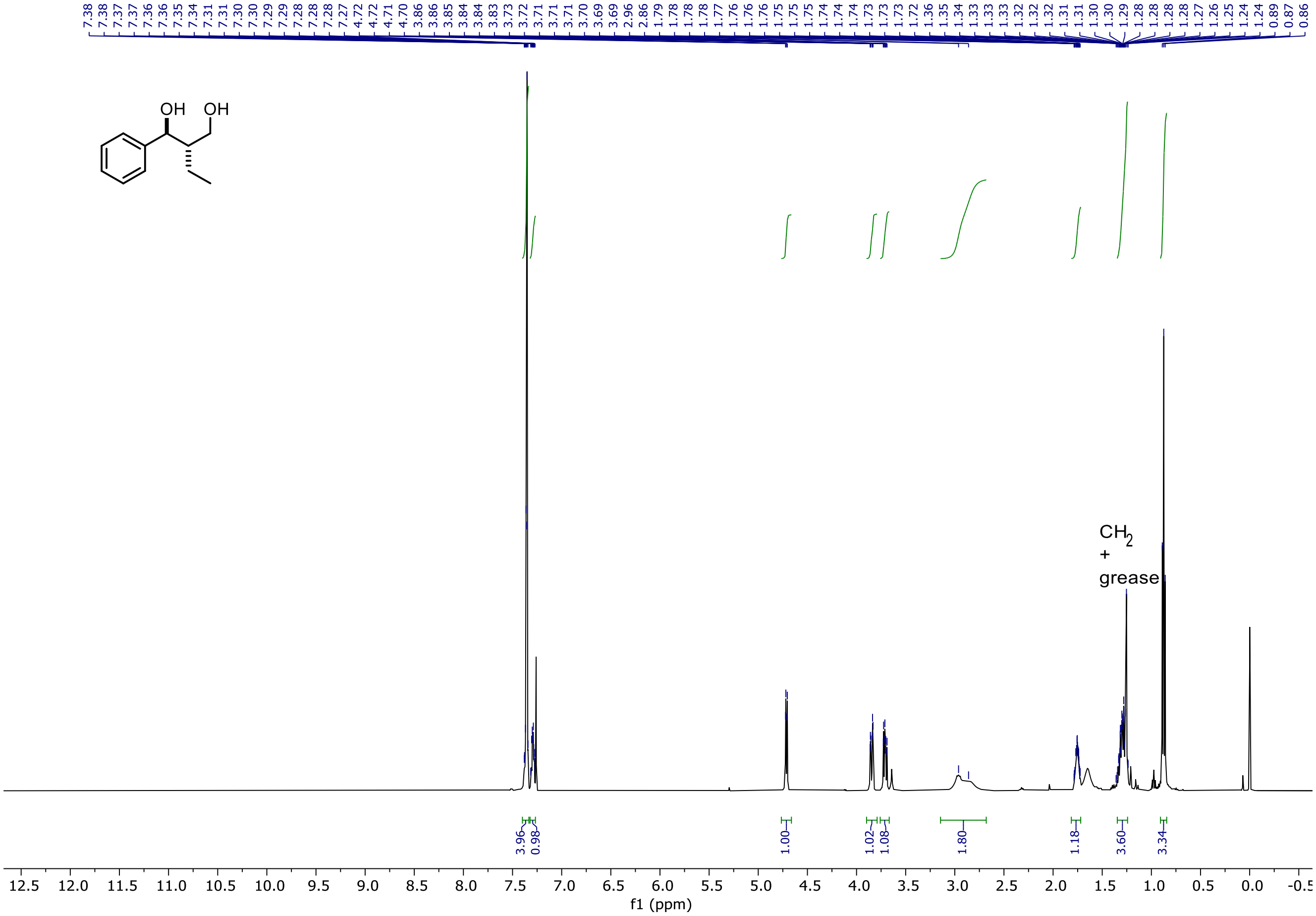

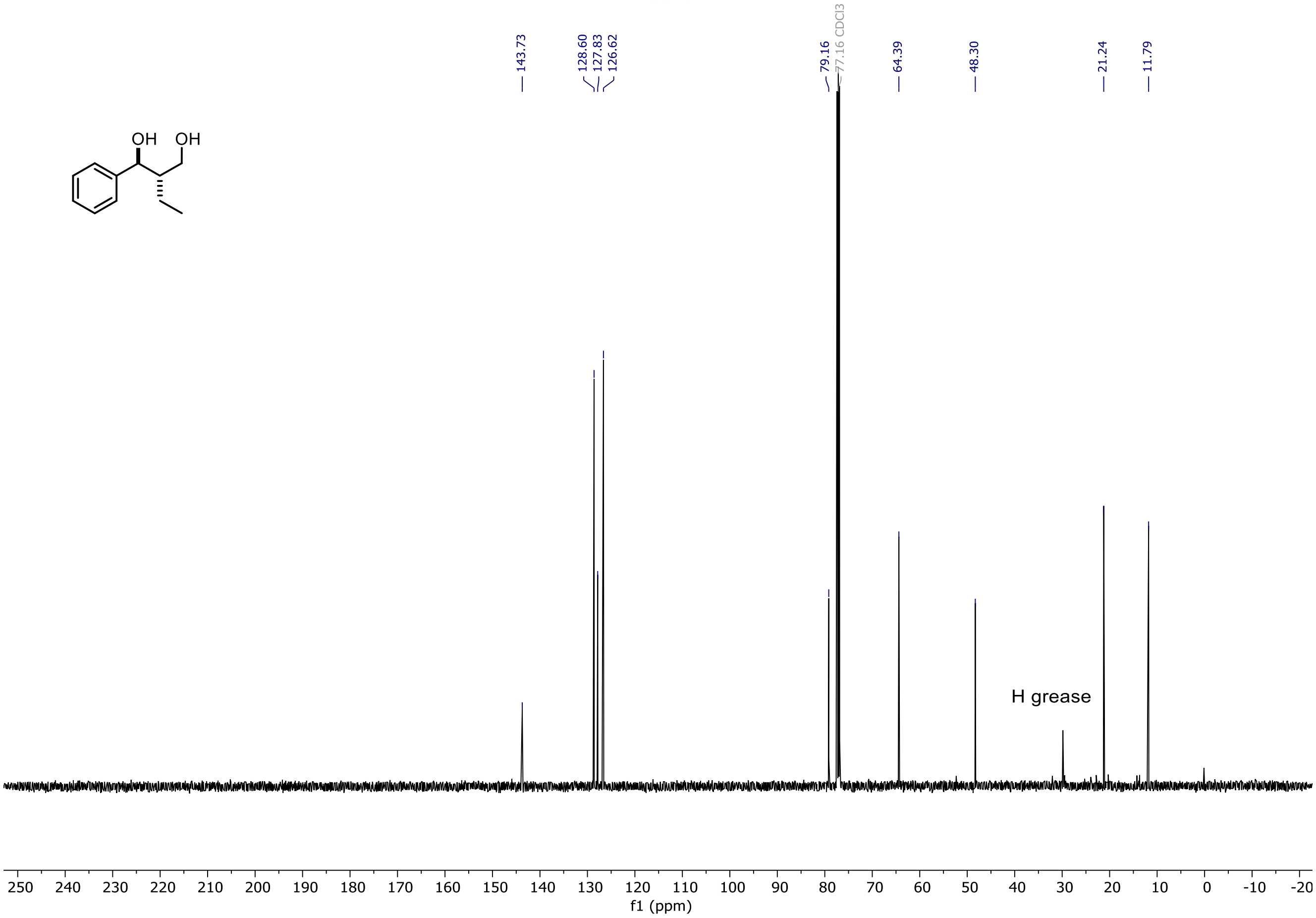

(*S*, *S*)-Imidodiphosphorimidate (7c)

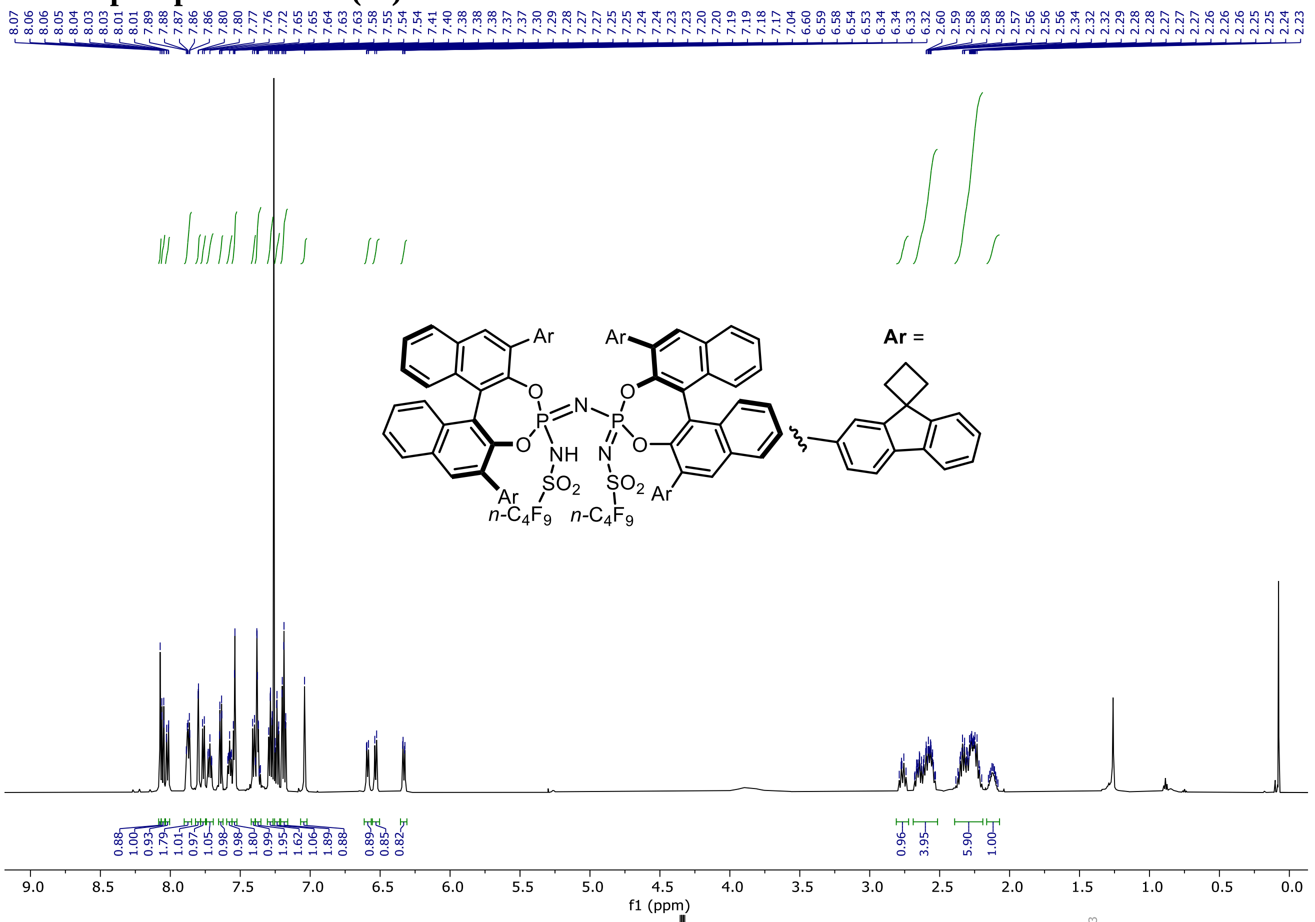

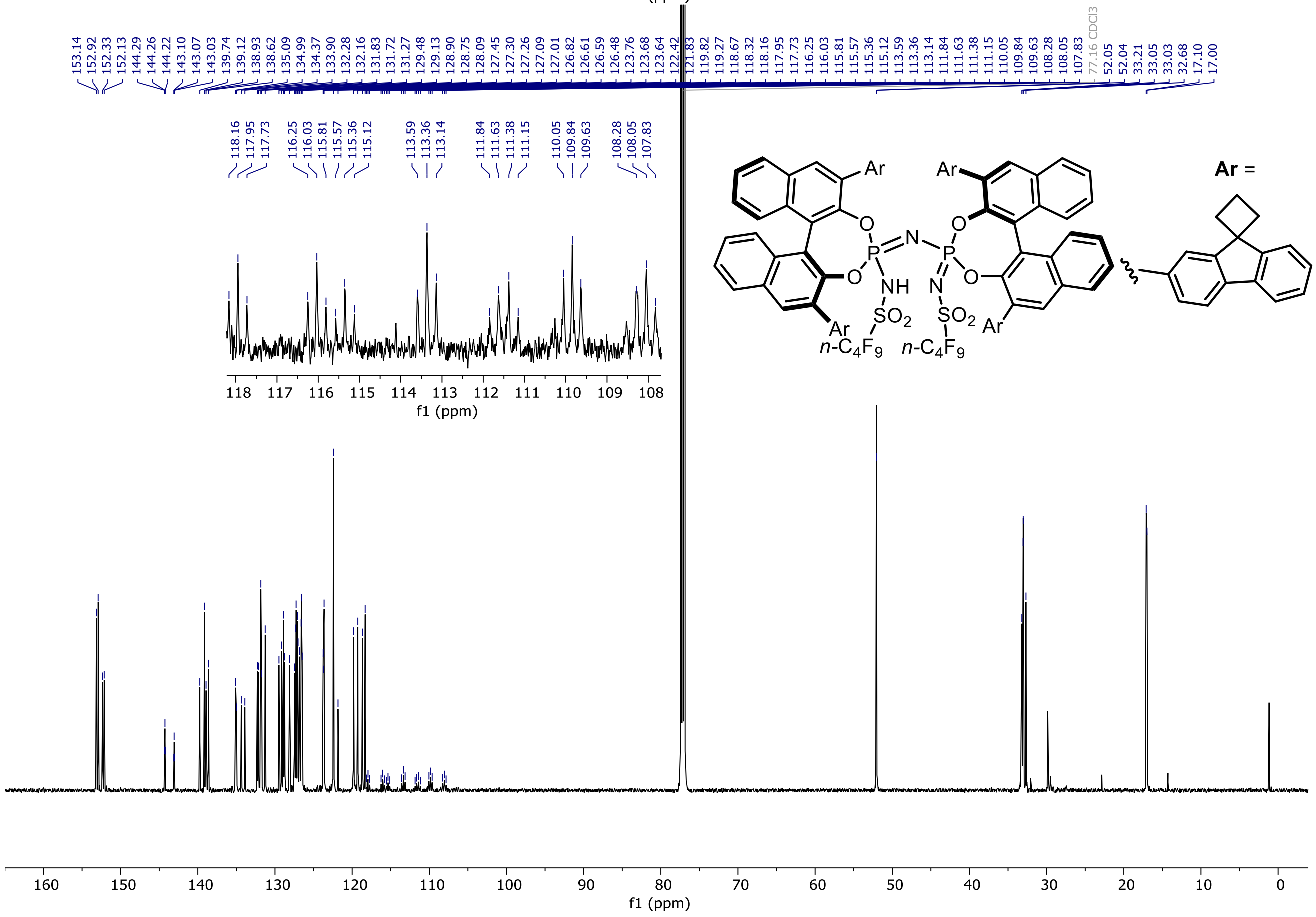

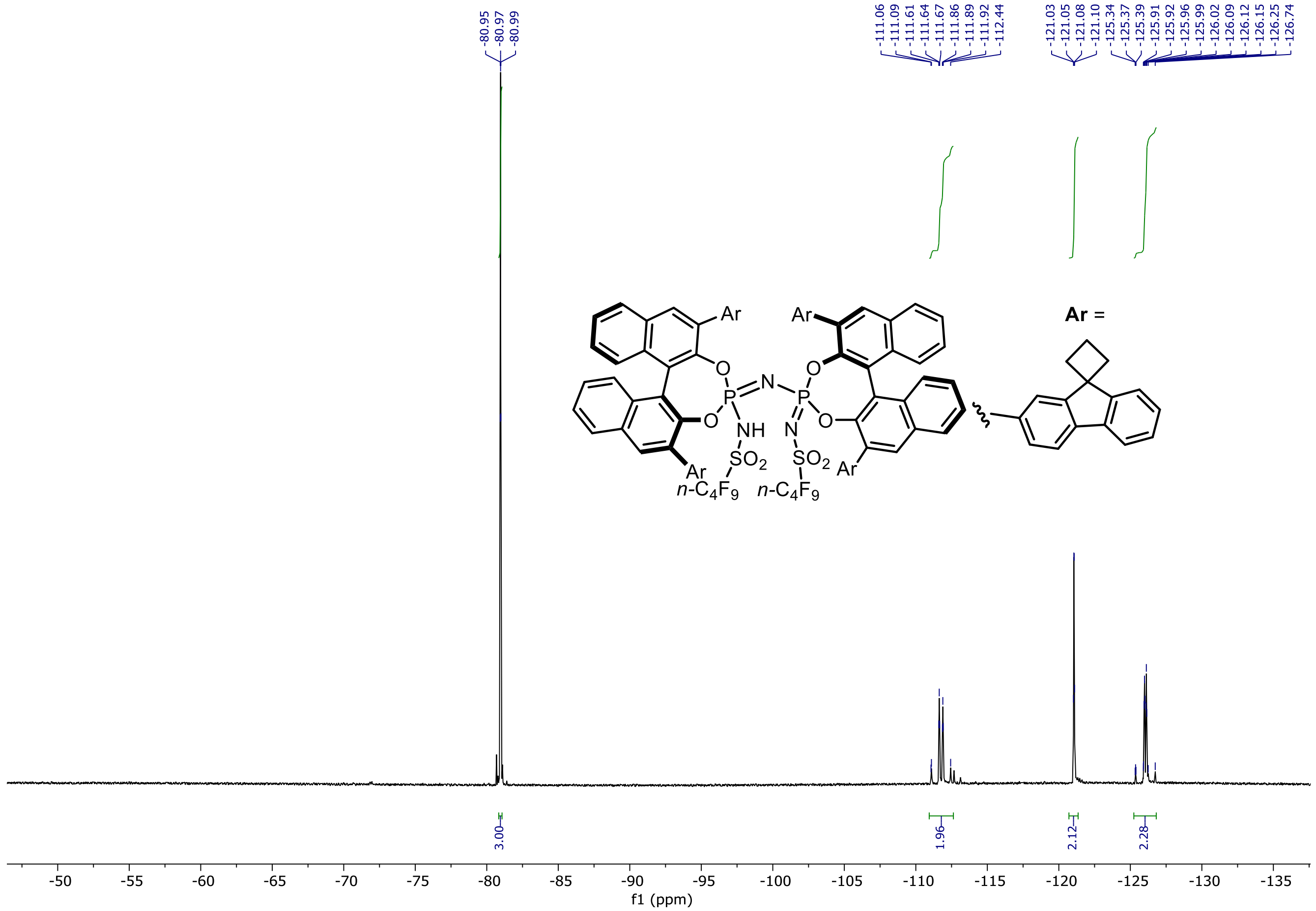
-80.95
-80.97
-80.99
-111.06
-111.09
-111.61
-111.64
-111.67
-111.86
-111.89
-111.92
-112.44
-121.03
-121.05
-121.08
-121.10
-125.34
-125.37
-125.39
-125.91
-125.92
-125.96
-125.99
-126.02
-126.09
-126.12
-126.15
-126.25
-126.74
Ar =
Ar
O
P
N
NH
SO2
n-C4F9
3.00
1.96
2.12
2.28
-50 -55 -60 -65 -70 -75 -80 -85 -90 -95 -100 -105 -110 -115 -120 -125 -130 -135
f1 (ppm)

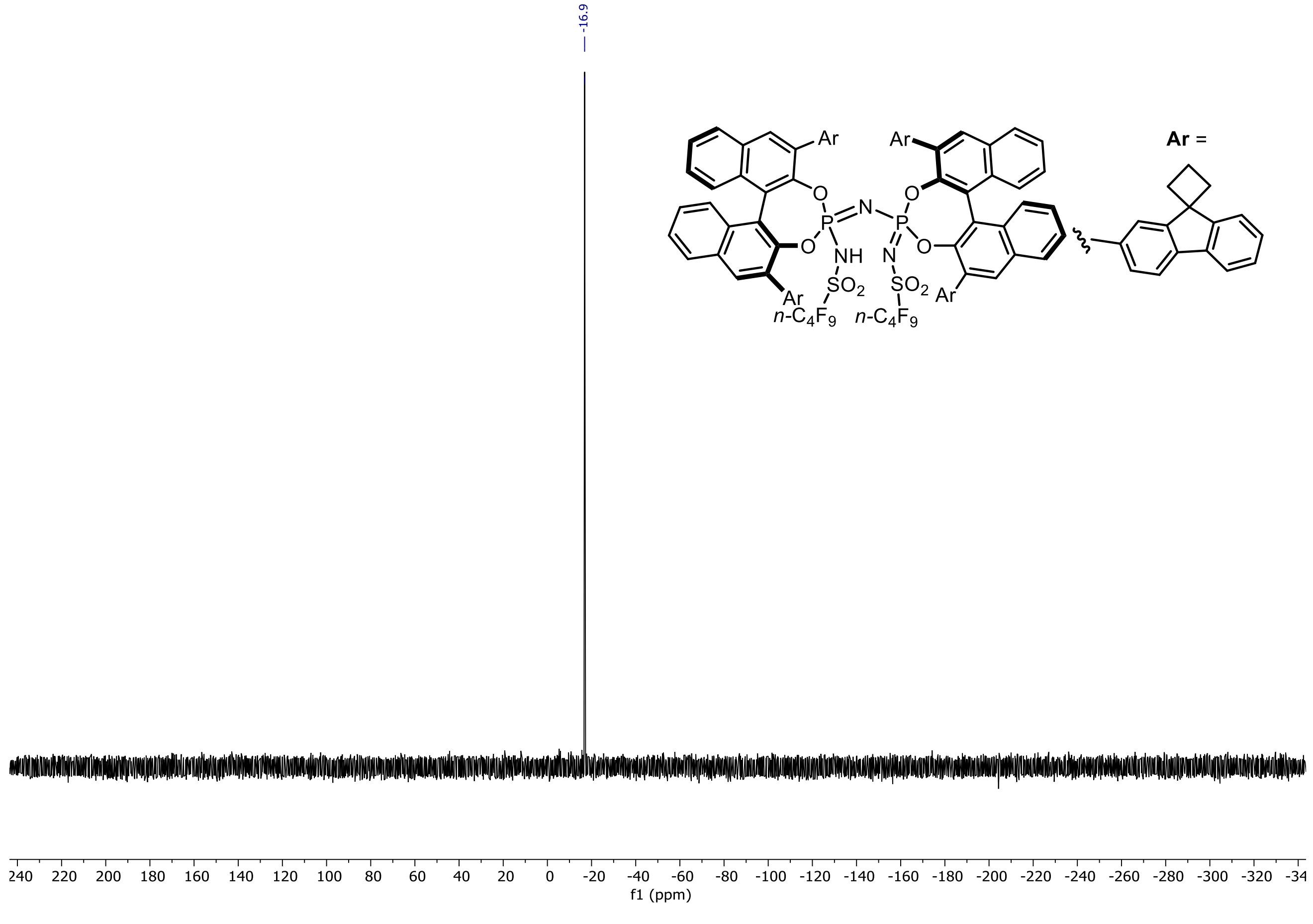
-16.9
Ar =
Ar
O
P
N
NH
SO2
n-C4F9
240 220 200 180 160 140 120 100 80 60 40 20 0 -20 -40 -60 -80 -100 -120 -140 -160 -180 -200 -220 -240 -260 -280 -300 -320 -34
f1 (ppm)

((Difluoromethyl)sulfonyl)phosphorimidoyl trichloride

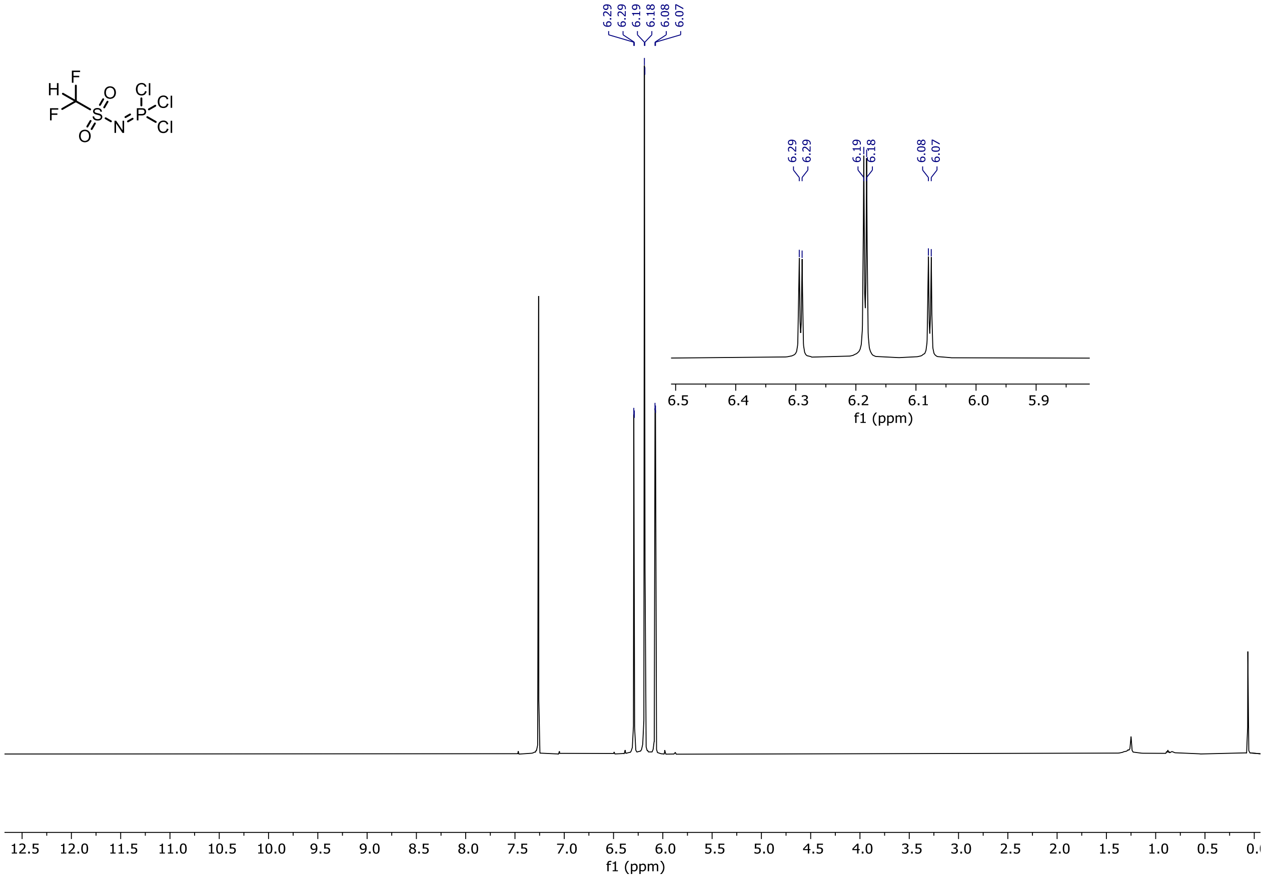

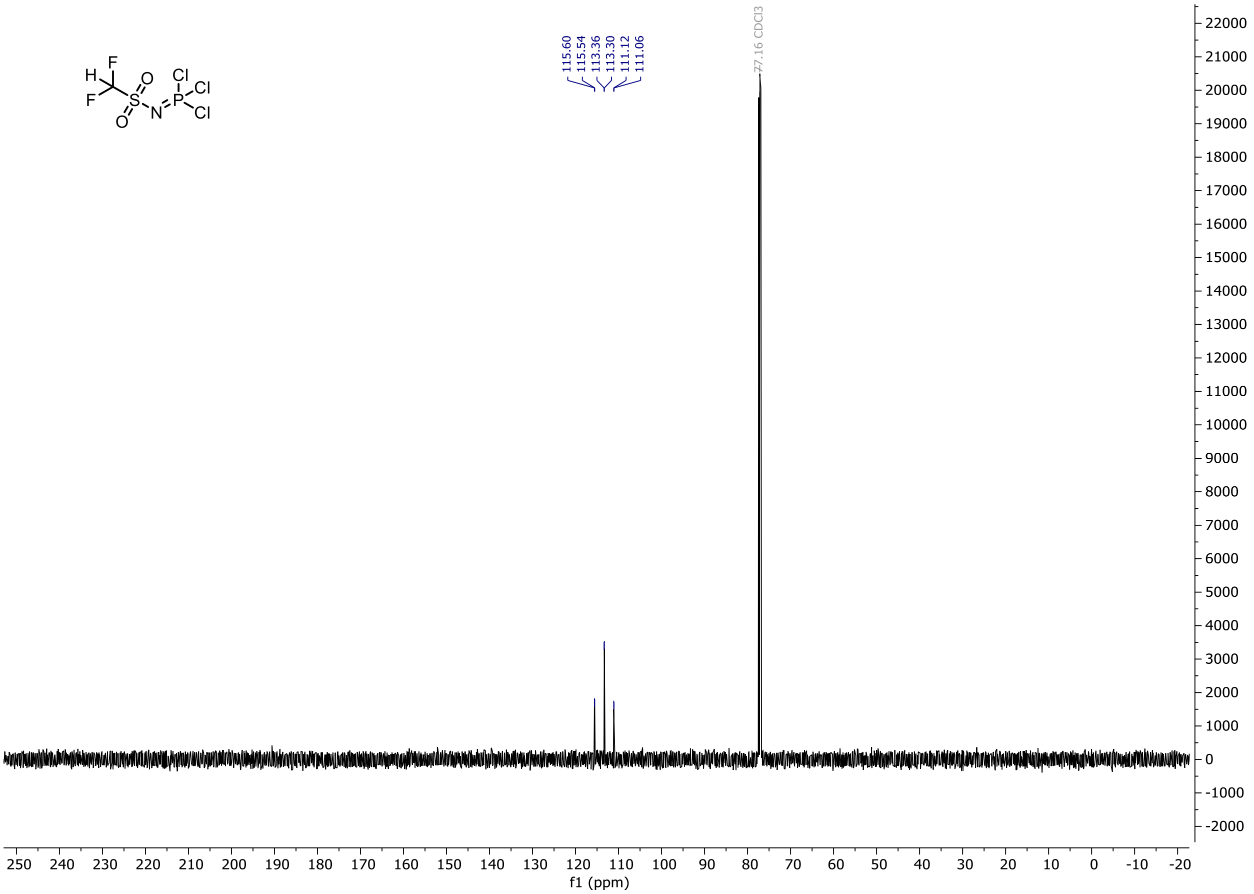

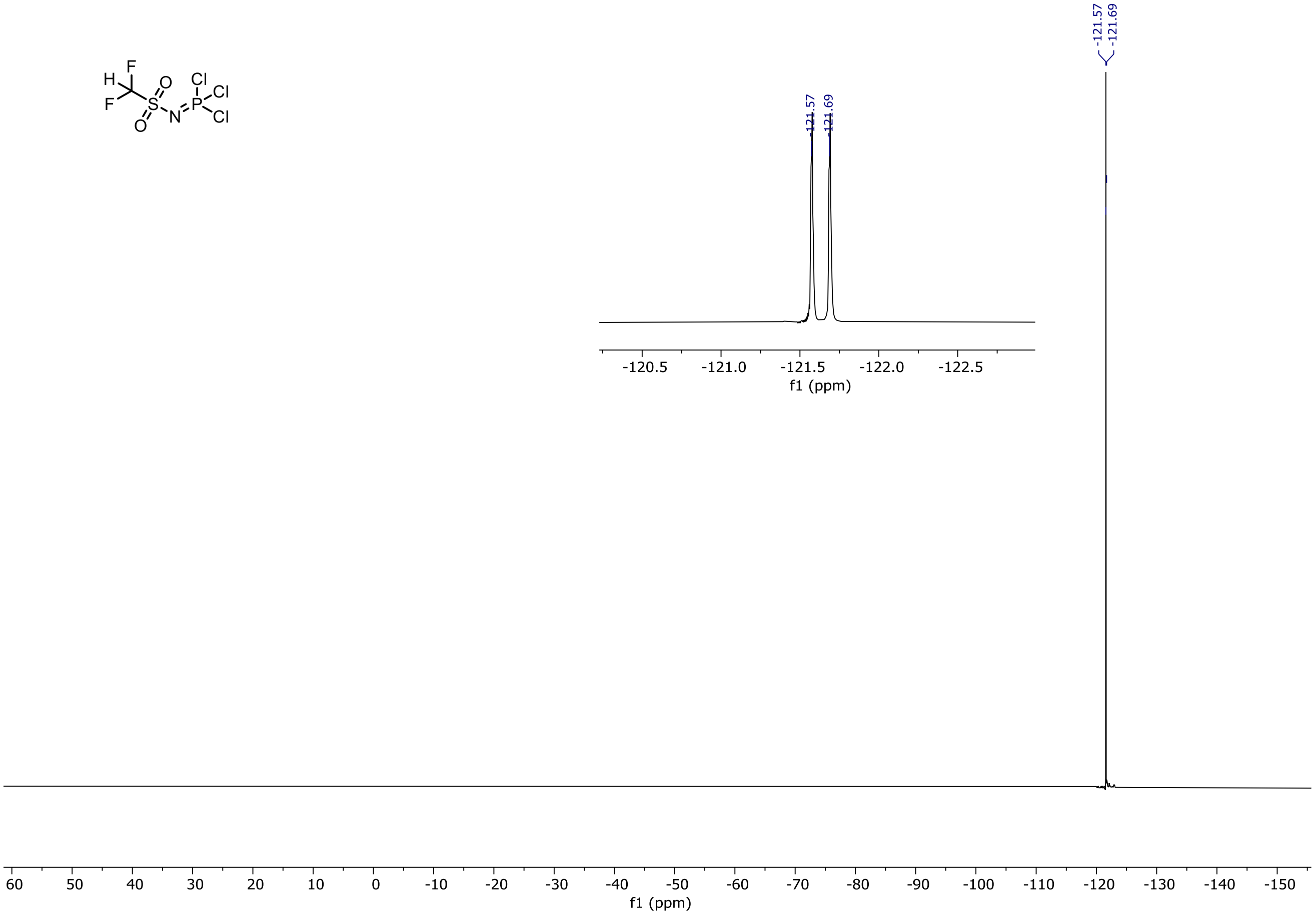
-121.57
-121.69
-120.5
-121.0
-121.5
-122.0
-122.5
f1 (ppm)
60
50
40
30
20
10
0
-10
-20
-30
-40
-50
-60
-70
-80
-90
-100
-110
-120
-130
-140
-150
f1 (ppm)

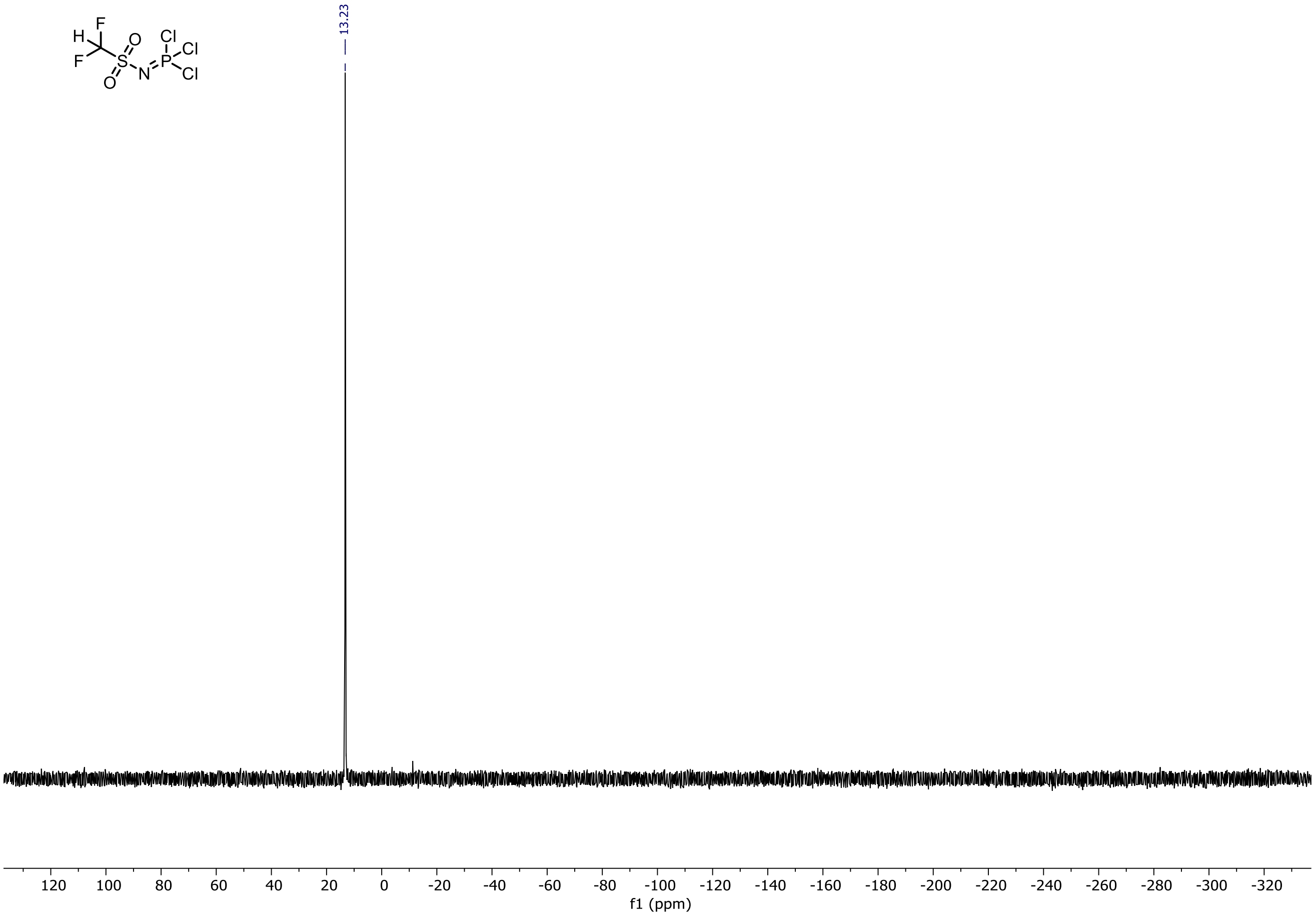
13.23
120
100
80
60
40
20
0
-20
-40
-60
-80
-100
-120
-140
-160
-180
-200
-220
-240
-260
-280
-300
-320
f1 (ppm)

(*S*, *S*)-Imidodiphosphorimidate (7d)

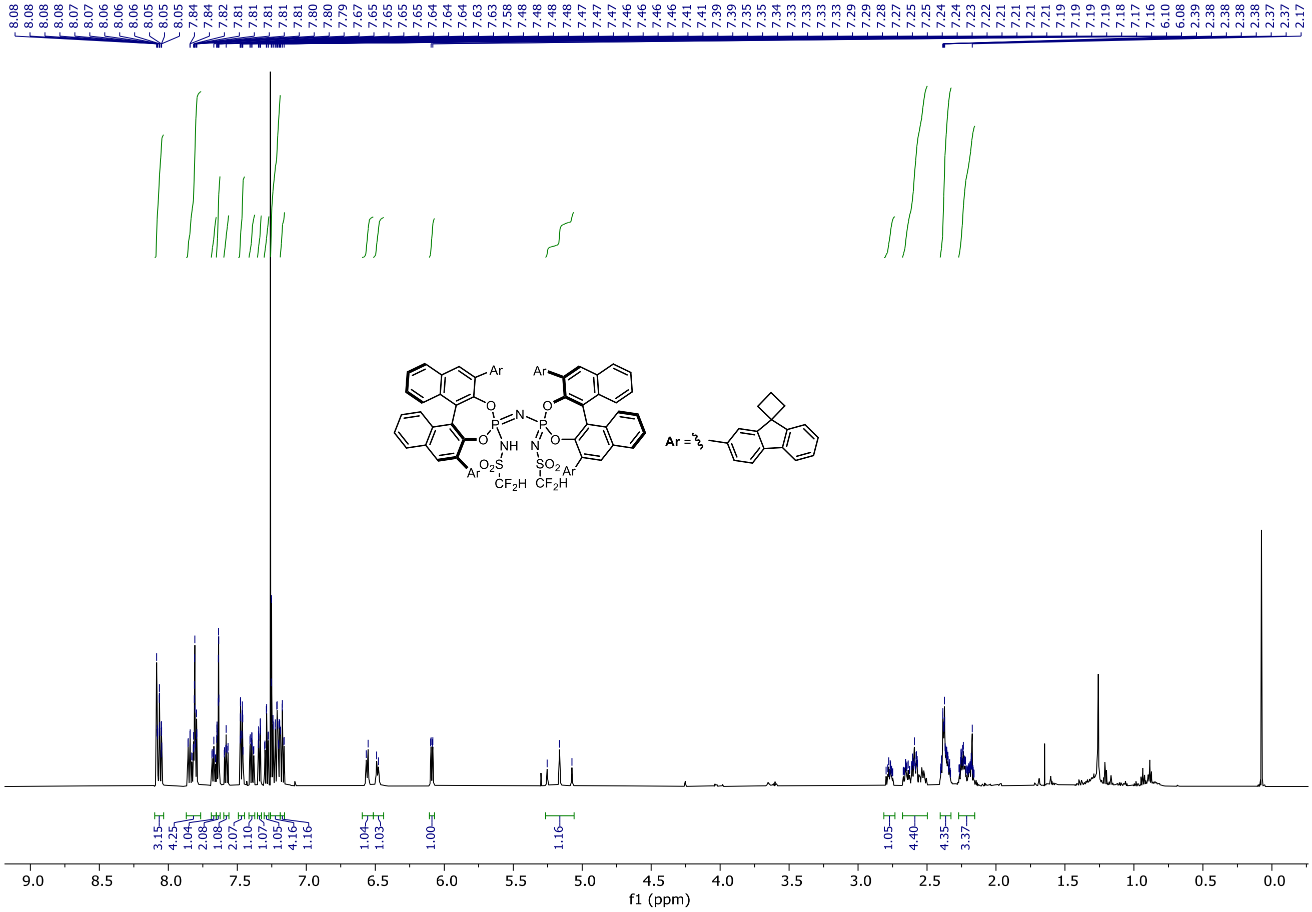

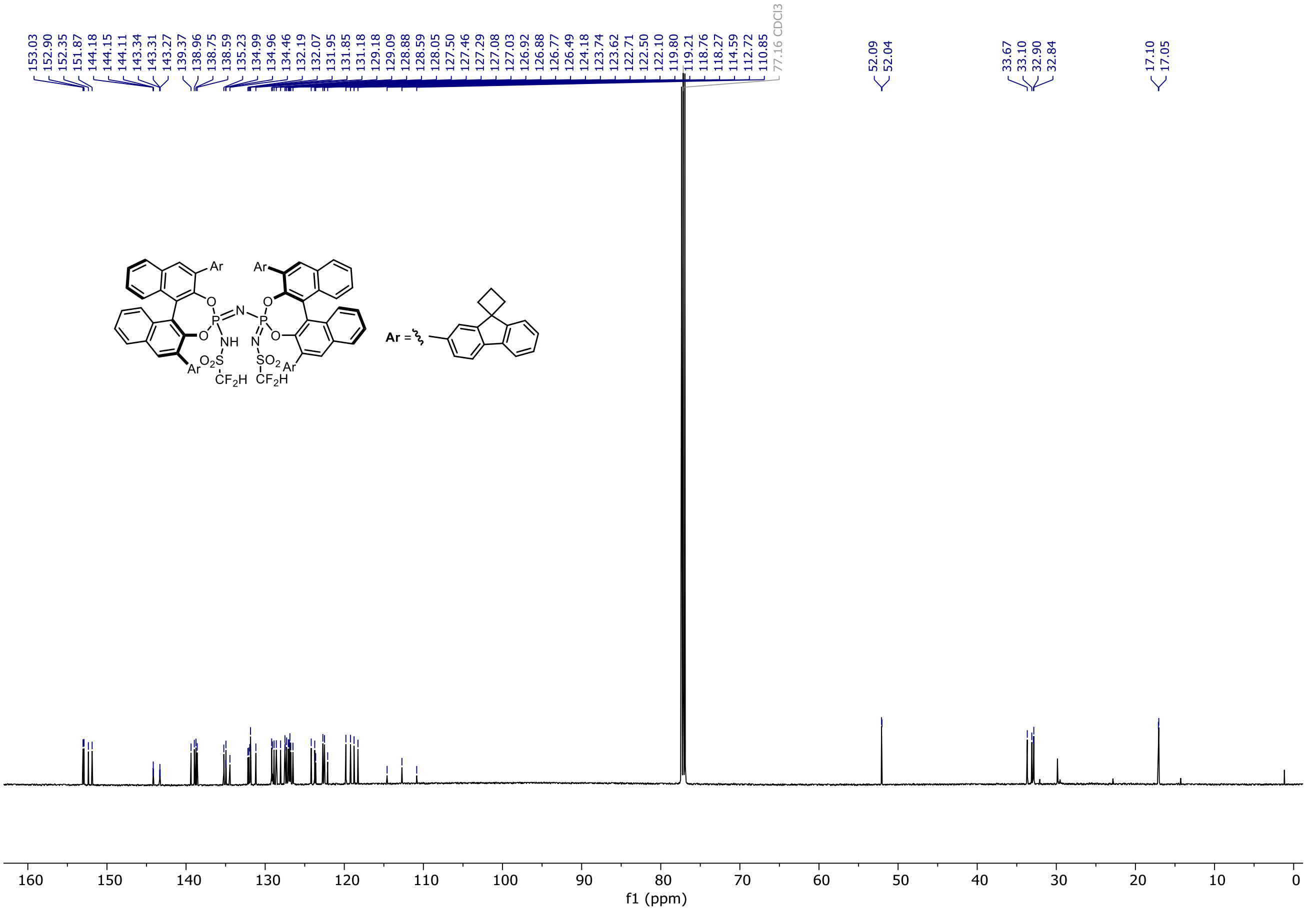

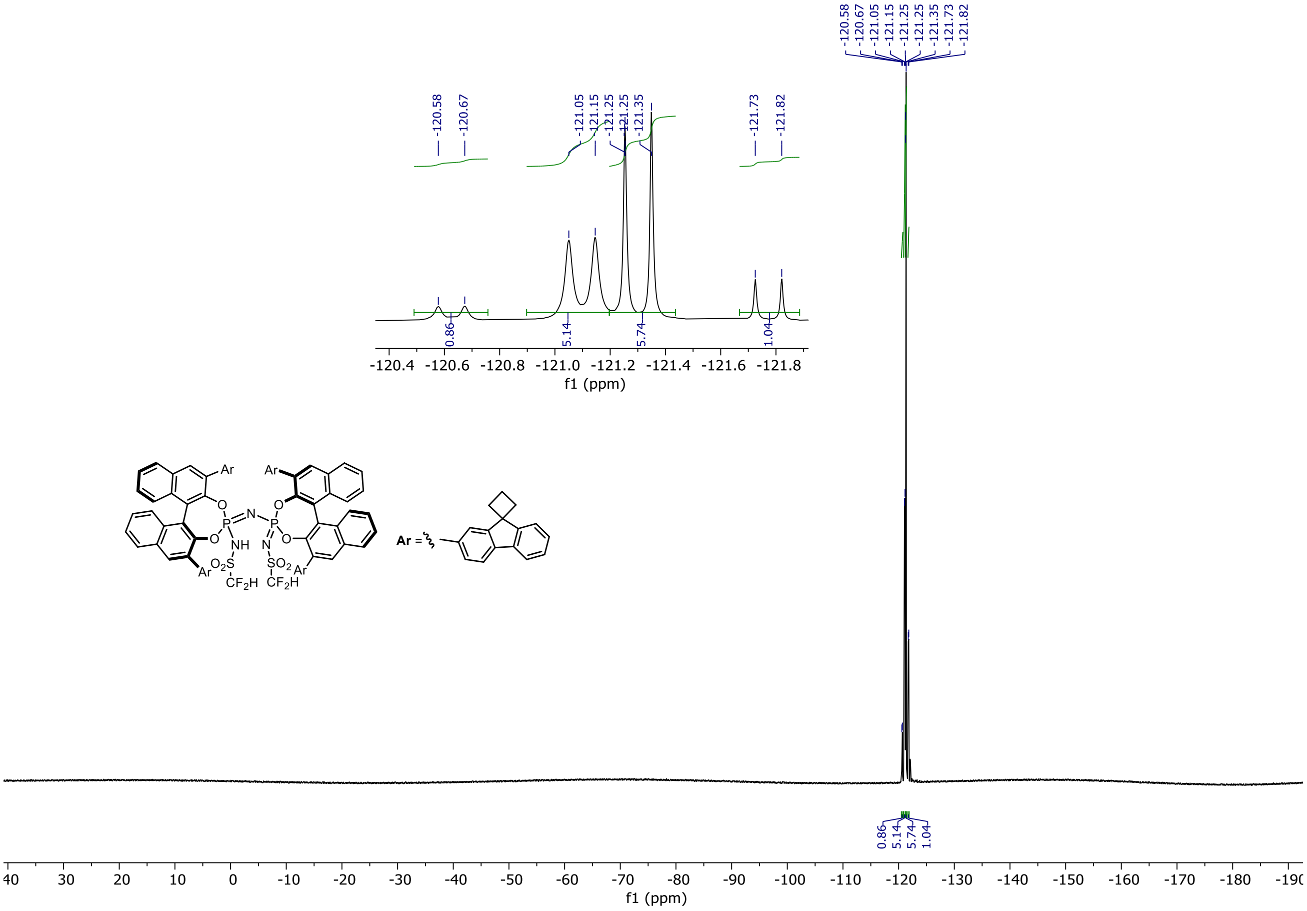
-120.58
-120.67
-121.05
-121.15
-121.25
-121.25
-121.35
-121.73
-121.82
0.86
5.14
5.74
1.04
f1 (ppm)
Ar
O
N
P
NH
O_2S
SO_2
CF_2H
Ar =

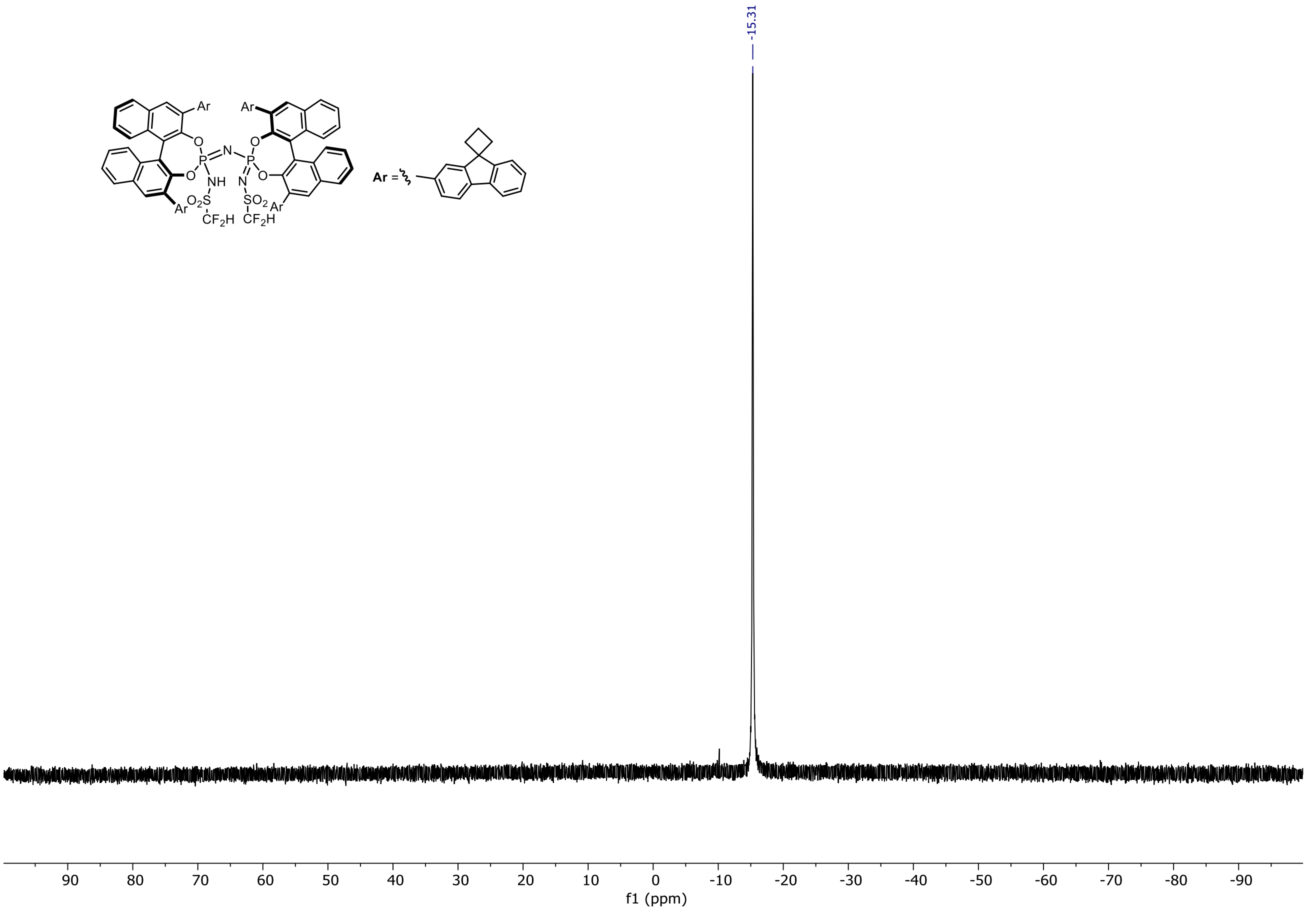
-15.31
Ar
O
N
P
NH
O_2S
SO_2
CF_2H
Ar =
f1 (ppm)

HPLC traces

HPLC (IC-3, *n*-heptane/*i*-PrOH=95:5, 0.5 mL/min, 298 K, 209 nm):

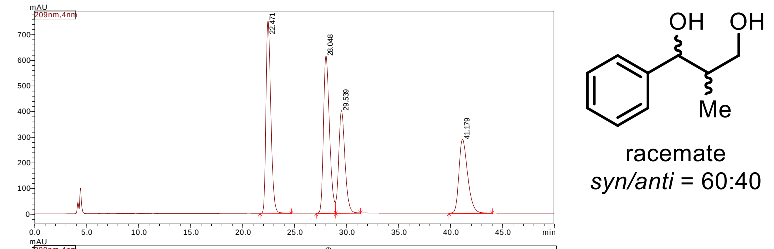

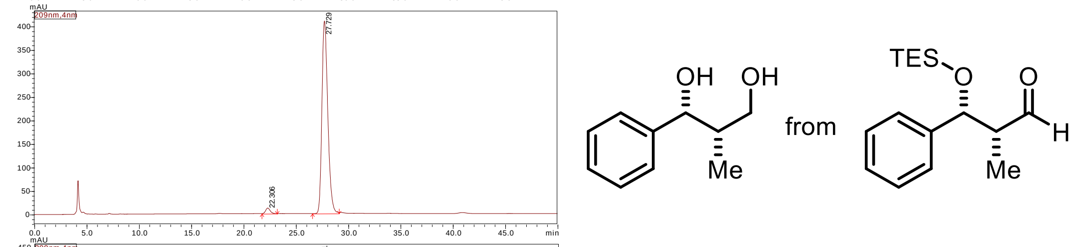

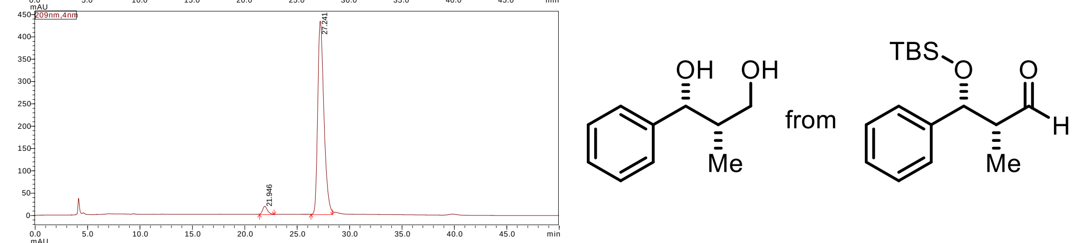

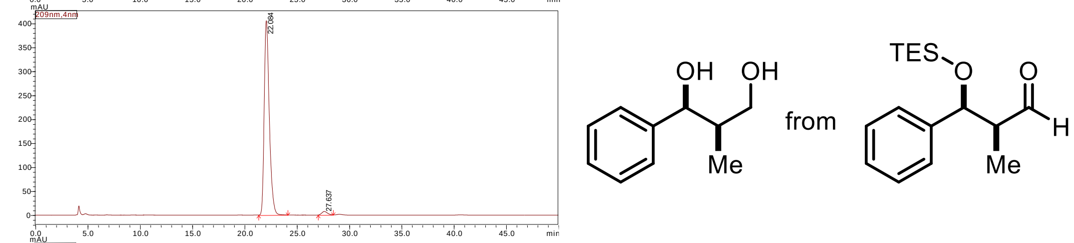

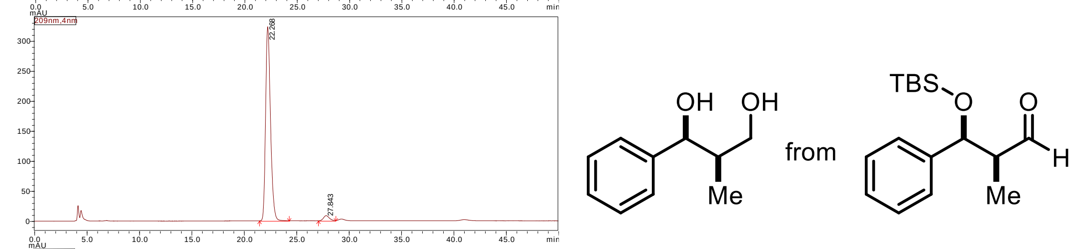

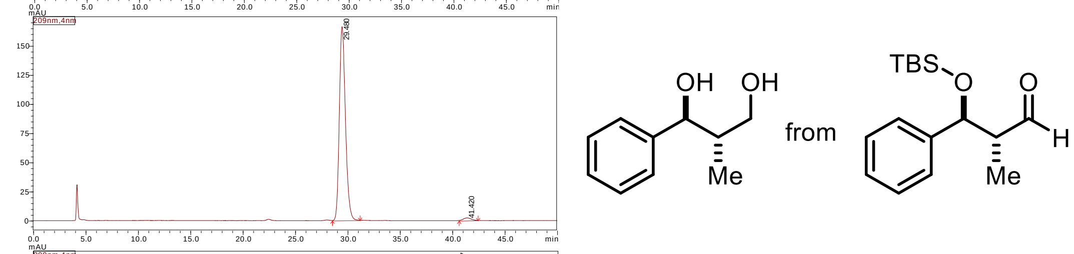

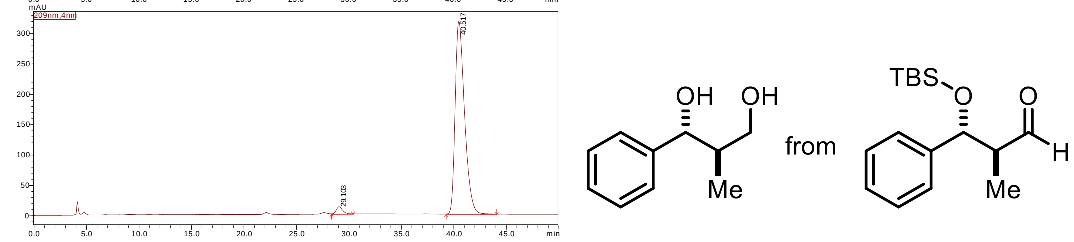

HPLC (IC-3, *n*-heptane/*i*-PrOH=90:10, 0.5 mL/min, 298 K, 215 nm):

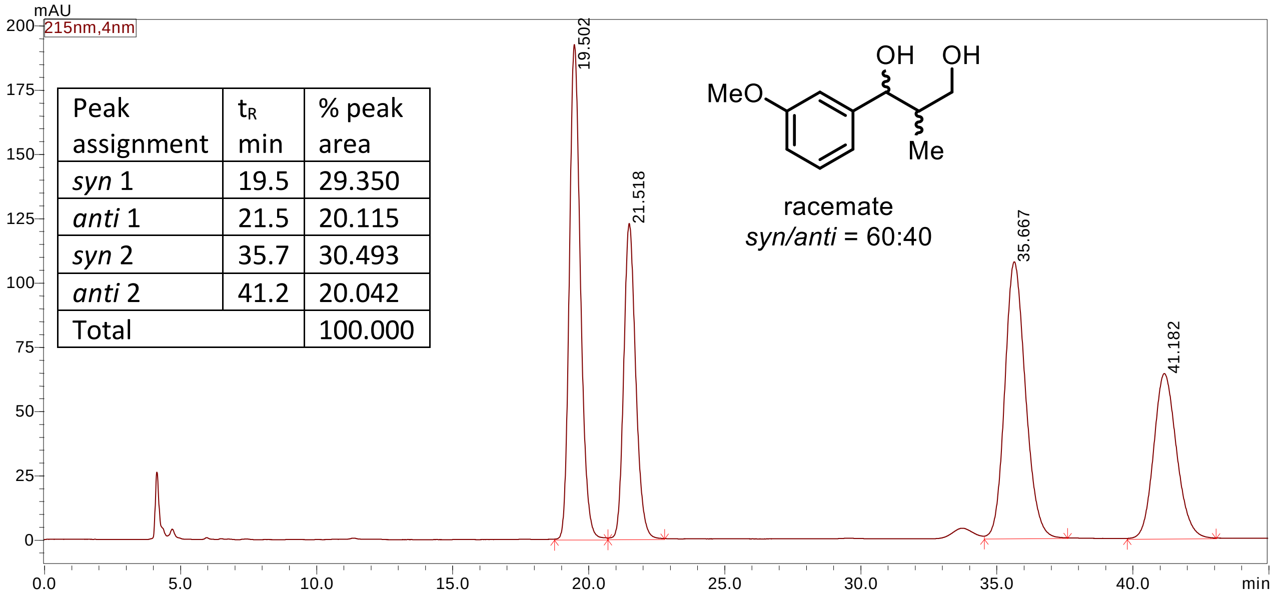

Peak assignment	t_R min	% peak area
syn 1	19.5	29.350
anti 1	21.5	20.115
syn 2	35.7	30.493
anti 2	41.2	20.042
Total		100.000

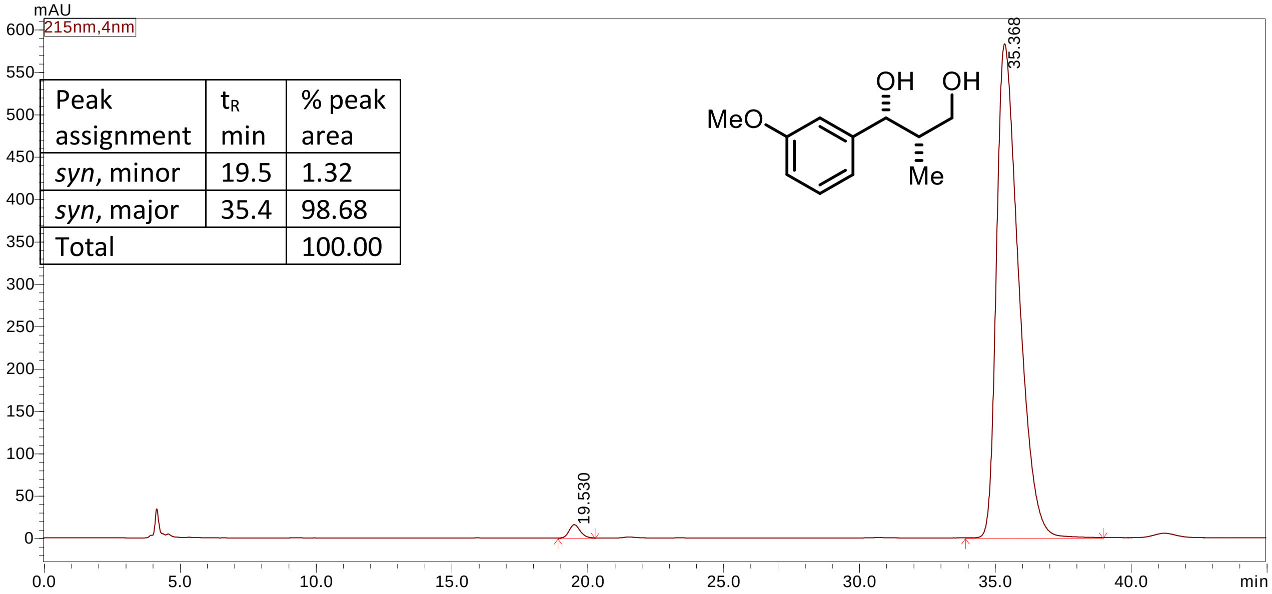

Peak assignment	t_R min	% peak area
syn, minor	19.5	1.32
syn, major	35.4	98.68
Total		100.00

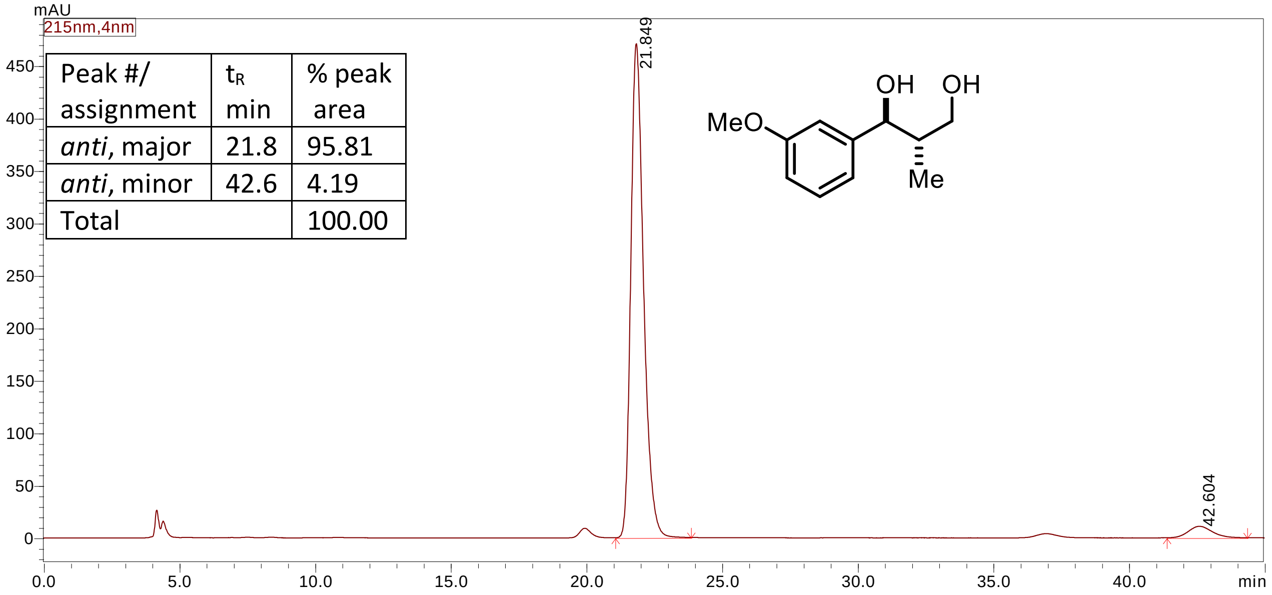

Peak #/ assignment	t_R min	% peak area
anti, major	21.8	95.81
anti, minor	42.6	4.19
Total		100.00

HPLC (IC-3, *n*-heptane/*i*-PrOH=90:10, 1 mL/min, 298 K, 225 nm):

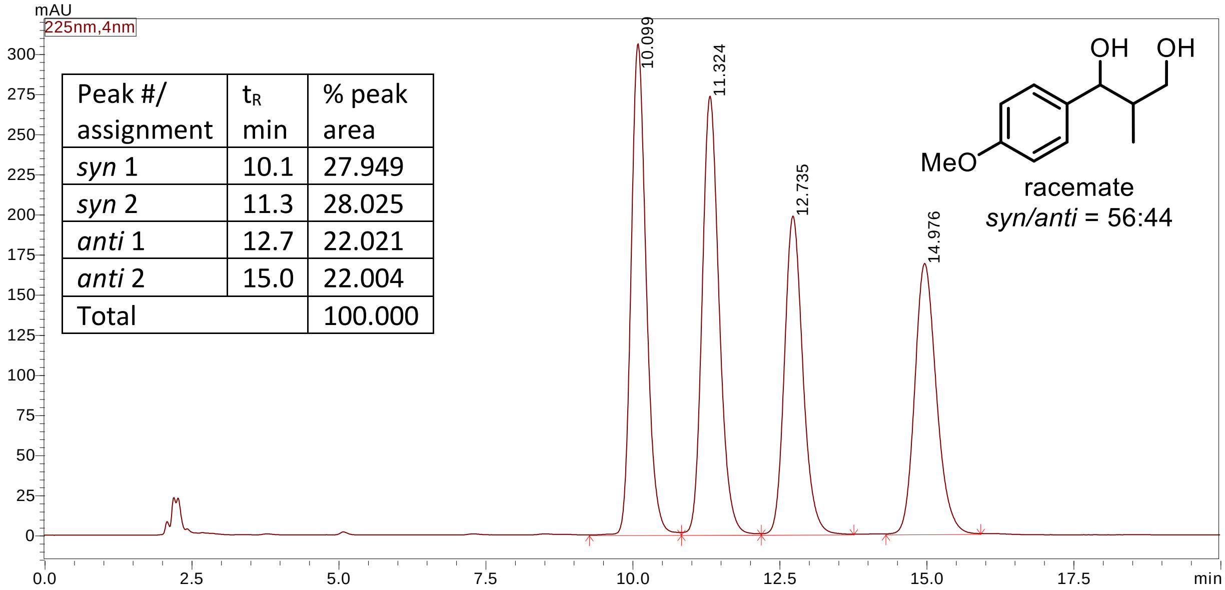

Peak #/ assignment	t_R min	% peak area
syn 1	10.1	27.949
syn 2	11.3	28.025
anti 1	12.7	22.021
anti 2	15.0	22.004
Total		100.000

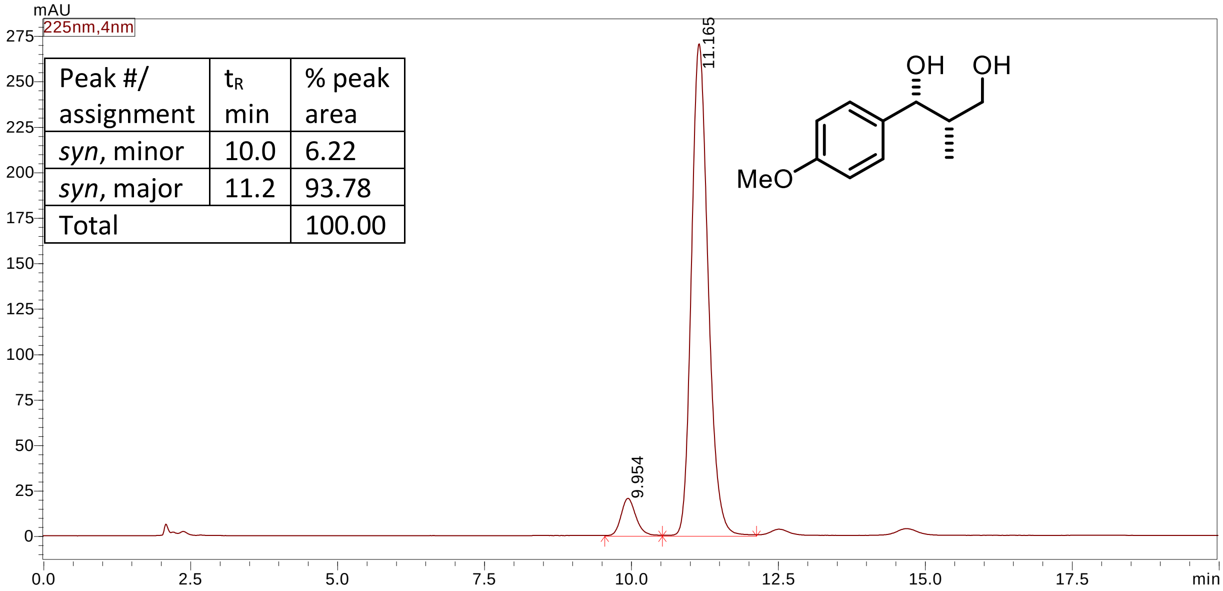

Peak #/ assignment	t_R min	% peak area
syn, minor	10.0	6.22
syn, major	11.2	93.78
Total		100.00

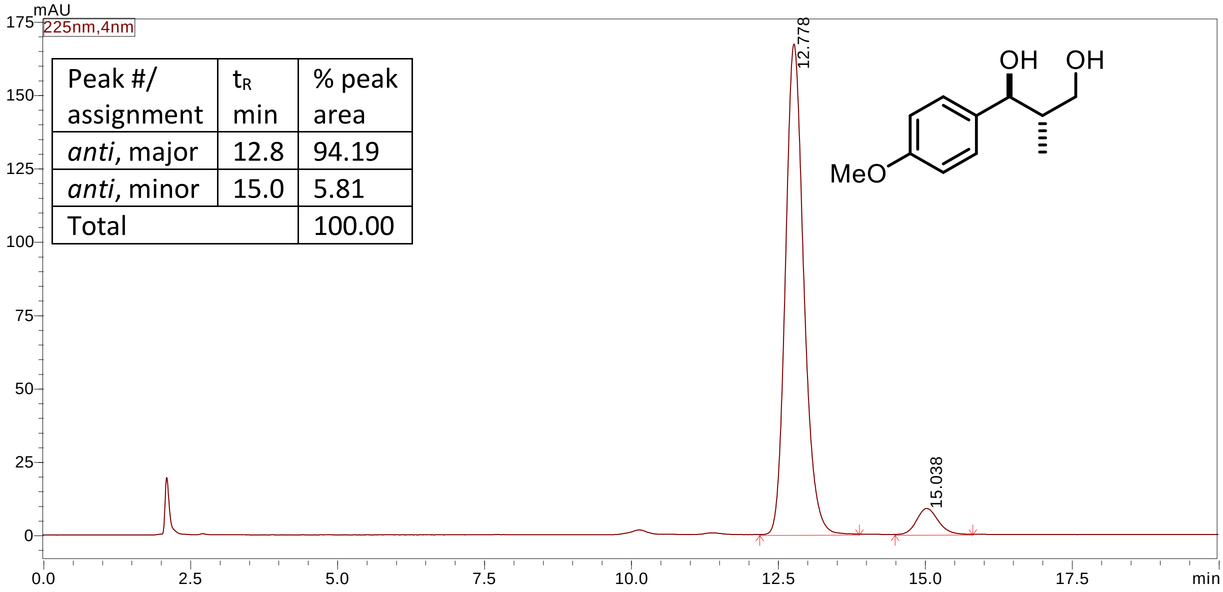

Peak #/ assignment	t_R min	% peak area
anti, major	12.8	94.19
anti, minor	15.0	5.81
Total		100.00

HPLC (IC-3, *n*-heptane/*i*-PrOH=95:5, 0.5 mL/min, 298 K, 211 nm):

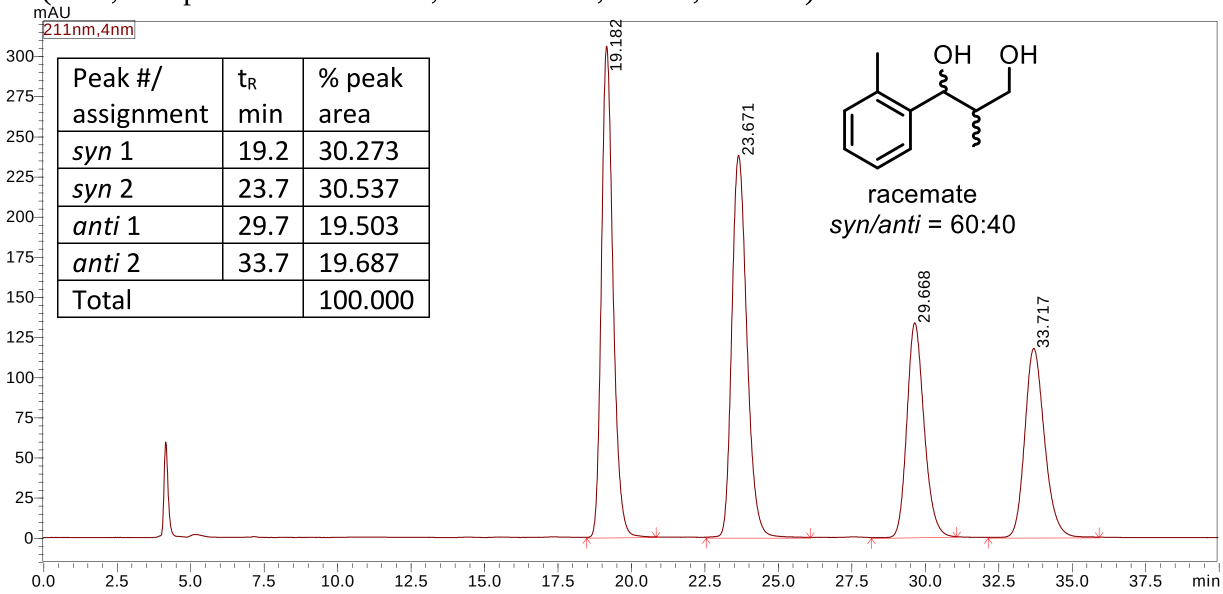

Peak #/ assignment	t_R min	% peak area
syn 1	19.2	30.273
syn 2	23.7	30.537
anti 1	29.7	19.503
anti 2	33.7	19.687
Total		100.000

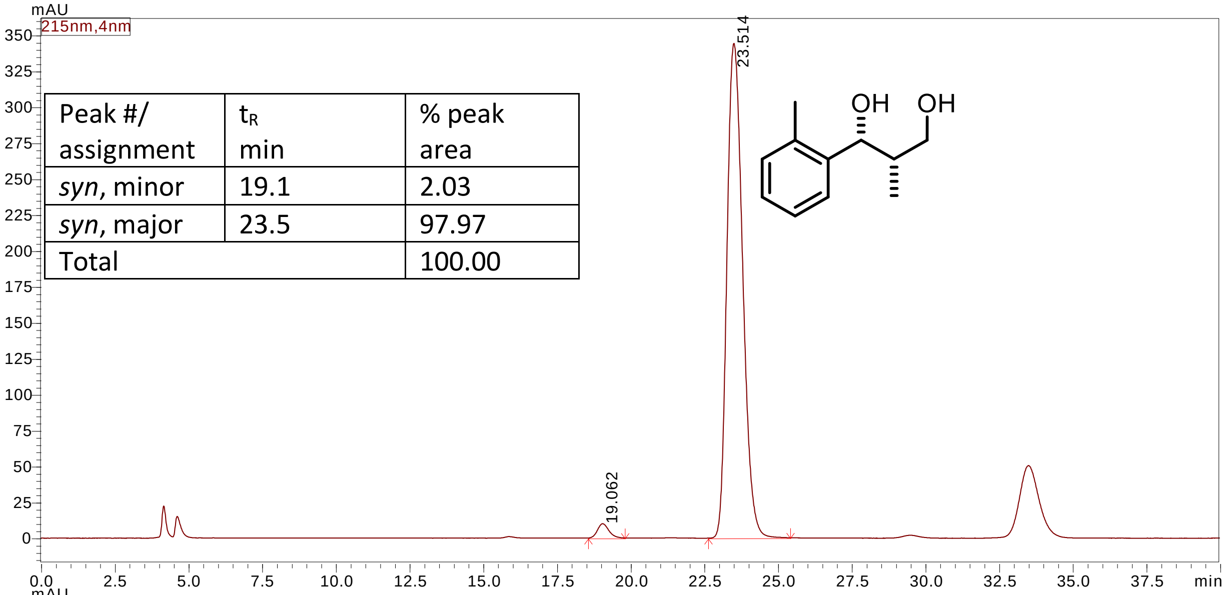

Peak #/ assignment	t_R min	% peak area
syn, minor	19.1	2.03
syn, major	23.5	97.97
Total		100.00

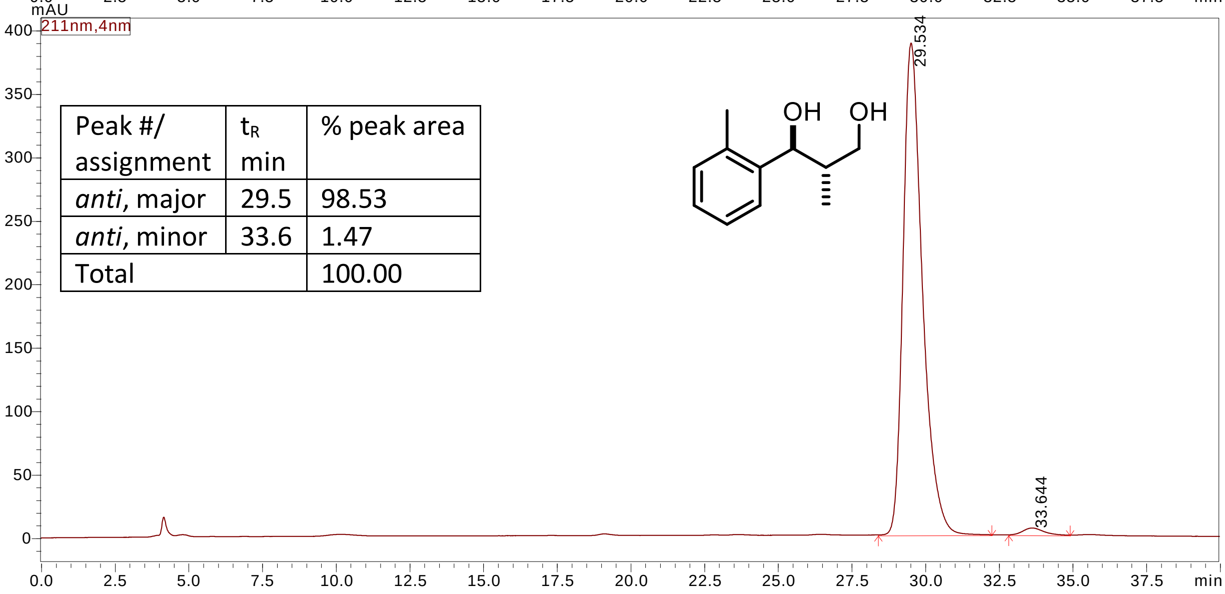

Peak #/ assignment	t_R min	% peak area
anti, major	29.5	98.53
anti, minor	33.6	1.47
Total		100.00

HPLC (IC-3, *n*-heptane/*i*-PrOH=95:5, 1.0 mL/min, 298 K, 215 nm):

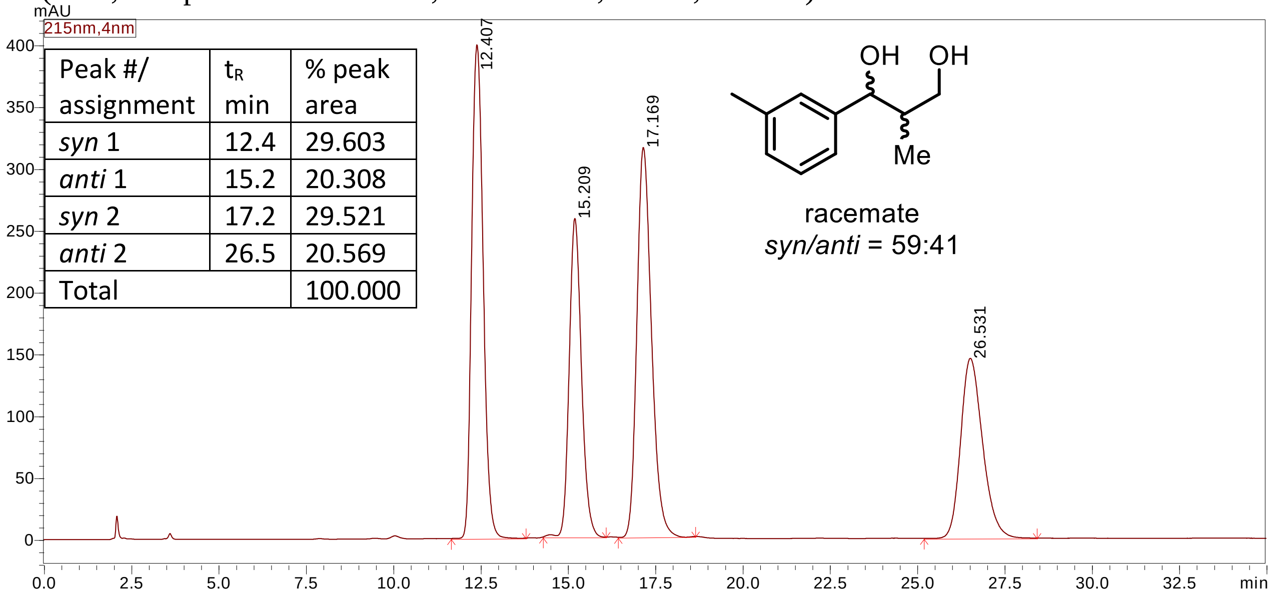

Peak #/ assignment	t_R min	% peak area
syn 1	12.4	29.603
anti 1	15.2	20.308
syn 2	17.2	29.521
anti 2	26.5	20.569
Total		100.000

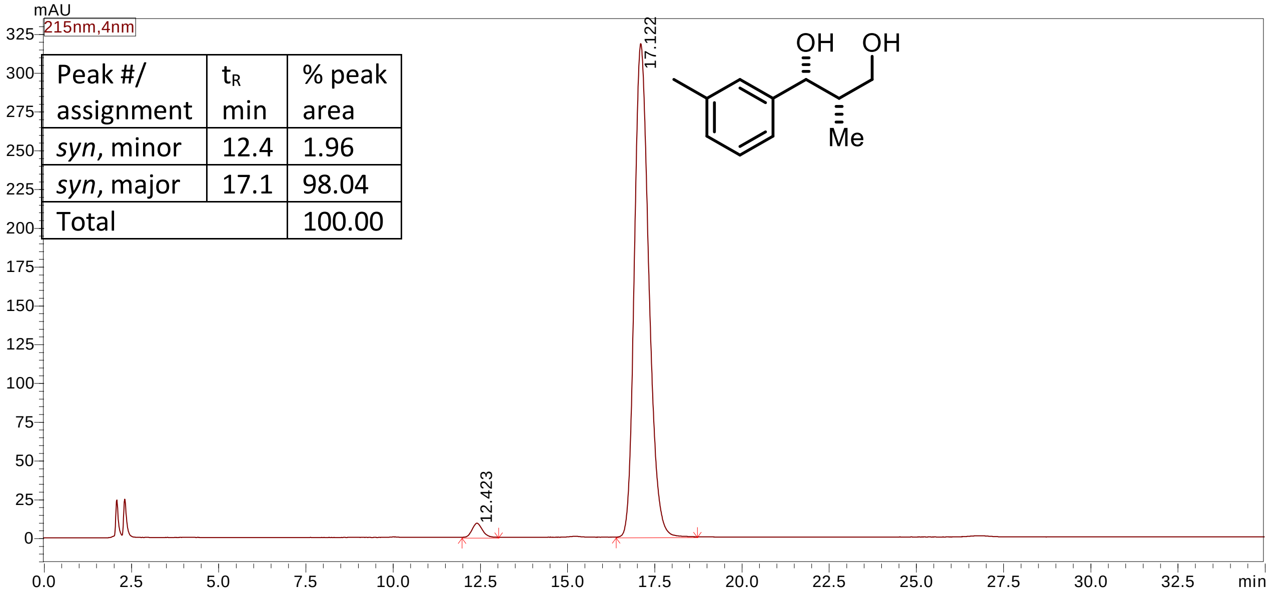

Peak #/ assignment	t_R min	% peak area
syn, minor	12.4	1.96
syn, major	17.1	98.04
Total		100.00

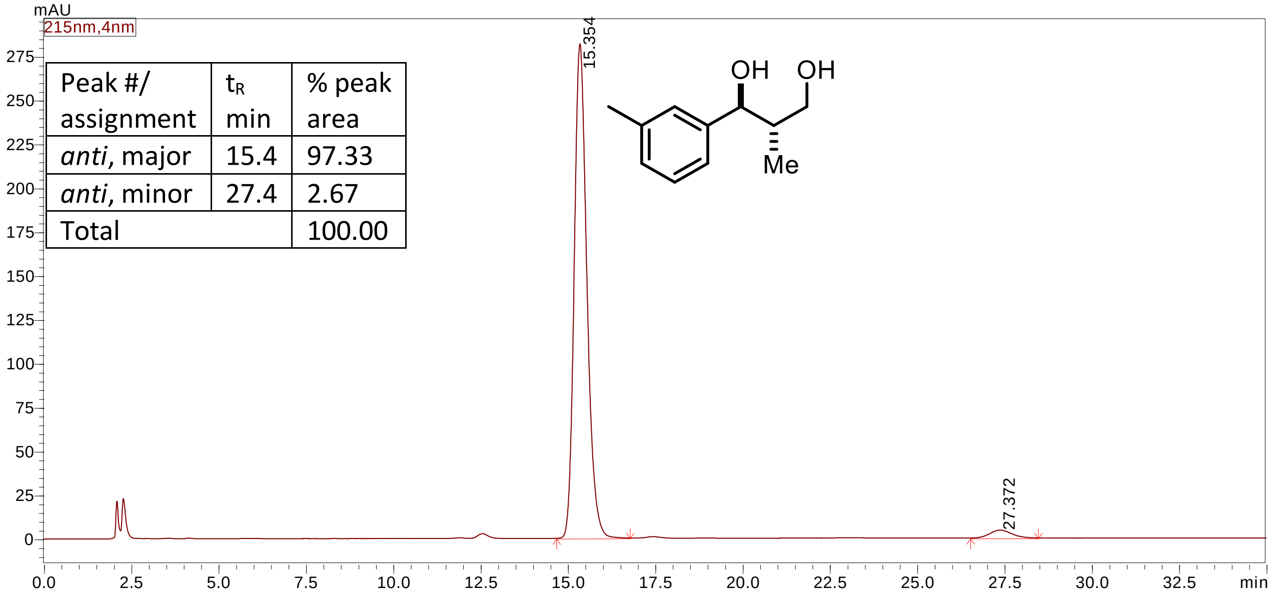

Peak #/ assignment	t_R min	% peak area
anti, major	15.4	97.33
anti, minor	27.4	2.67
Total		100.00

HPLC (IC-3, *n*-heptane/*i*-PrOH=95:5, 1 mL/min, 298 K, 213 nm):

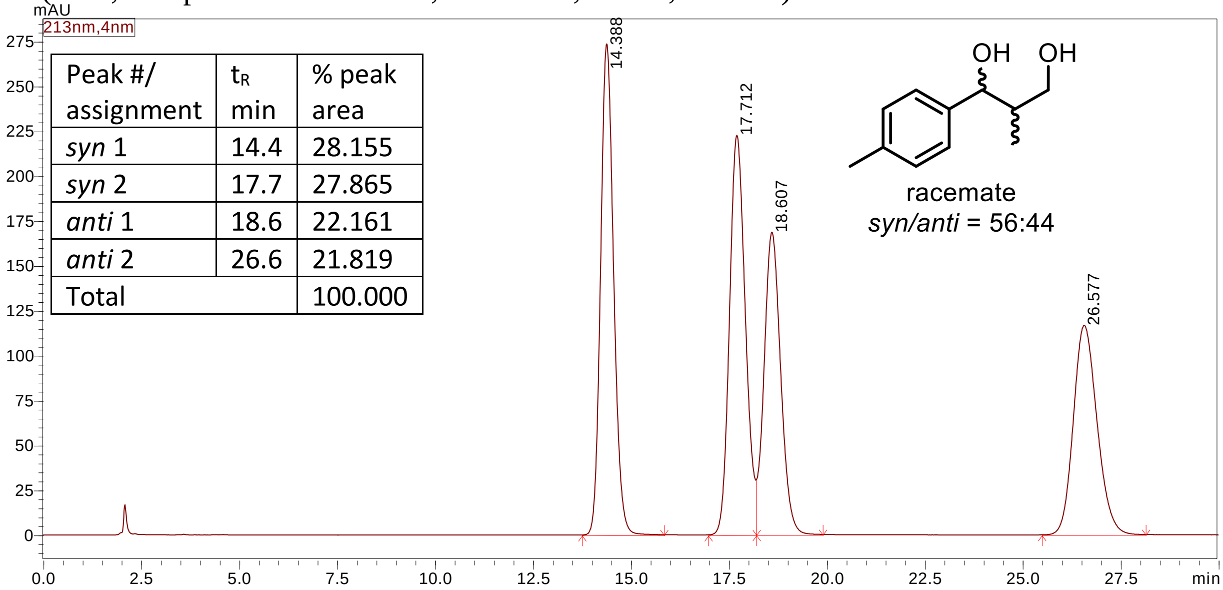

Peak #/ assignment	t_R min	% peak area
syn 1	14.4	28.155
syn 2	17.7	27.865
anti 1	18.6	22.161
anti 2	26.6	21.819
Total		100.000

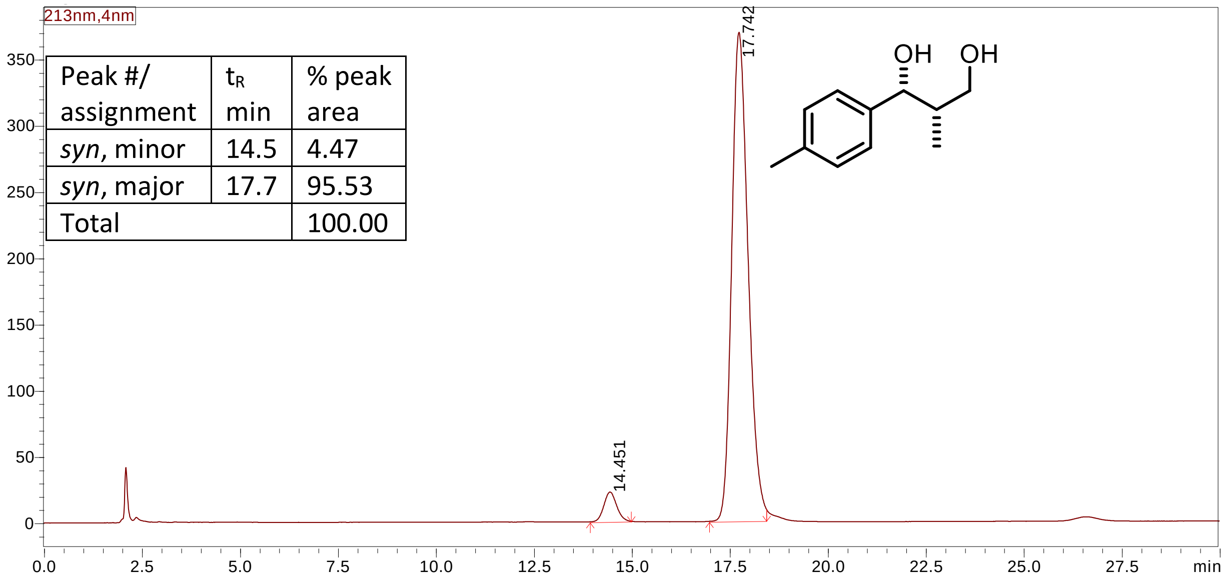

Peak #/ assignment	t_R min	% peak area
syn, minor	14.5	4.47
syn, major	17.7	95.53
Total		100.00

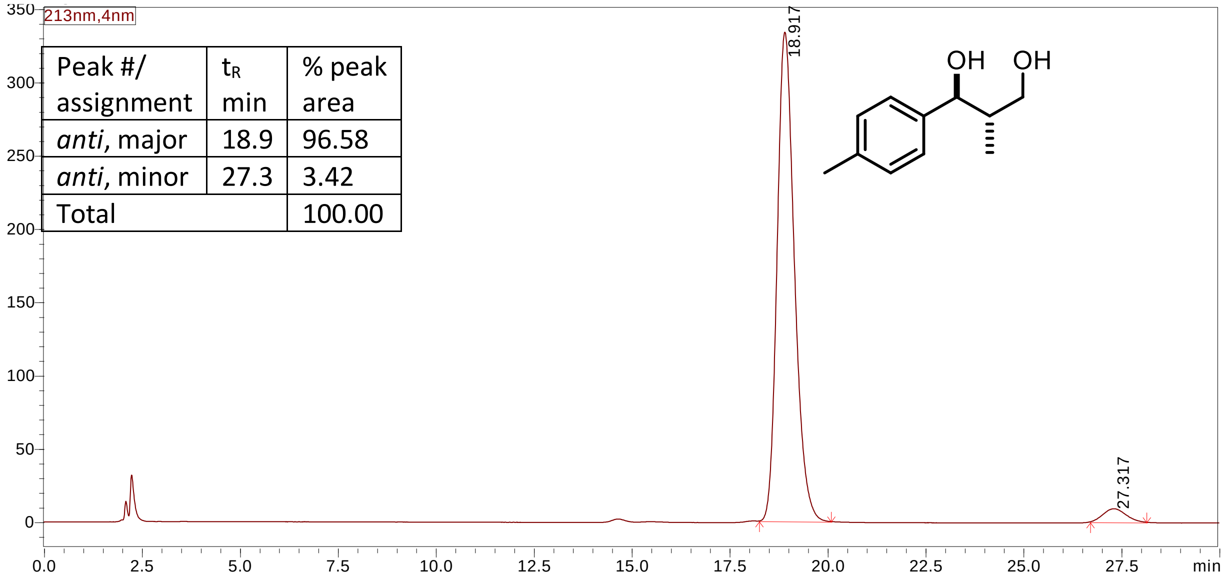

Peak #/ assignment	t_R min	% peak area
anti, major	18.9	96.58
anti, minor	27.3	3.42
Total		100.00

HPLC (IC-3, *n*-heptane/*i*-PrOH = 90:10, 1.0 ml/min 298 K, 215 nm):

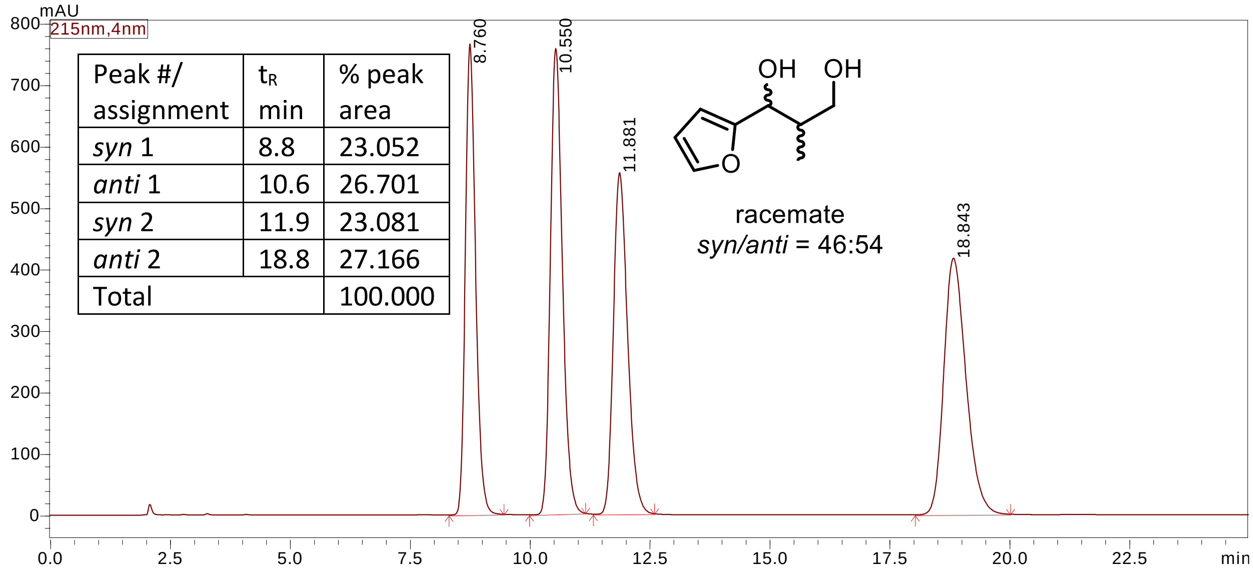

Peak #/ assignment	t_R min	% peak area
syn 1	8.8	23.052
anti 1	10.6	26.701
syn 2	11.9	23.081
anti 2	18.8	27.166
Total		100.000

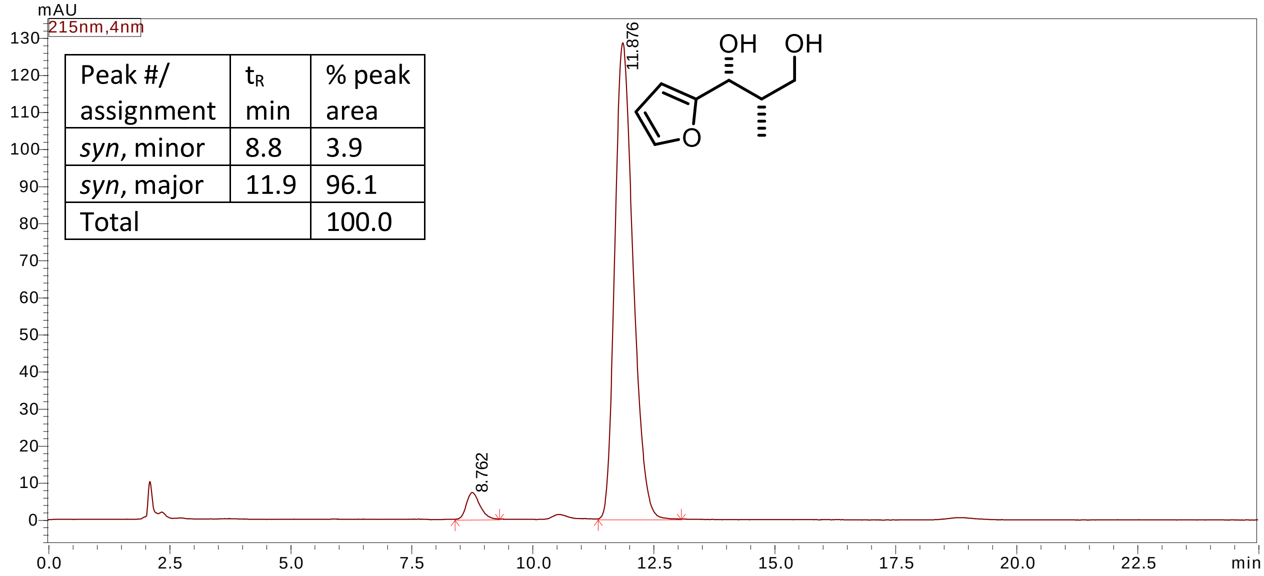

Peak #/ assignment	t_R min	% peak area
syn, minor	8.8	3.9
syn, major	11.9	96.1
Total		100.0

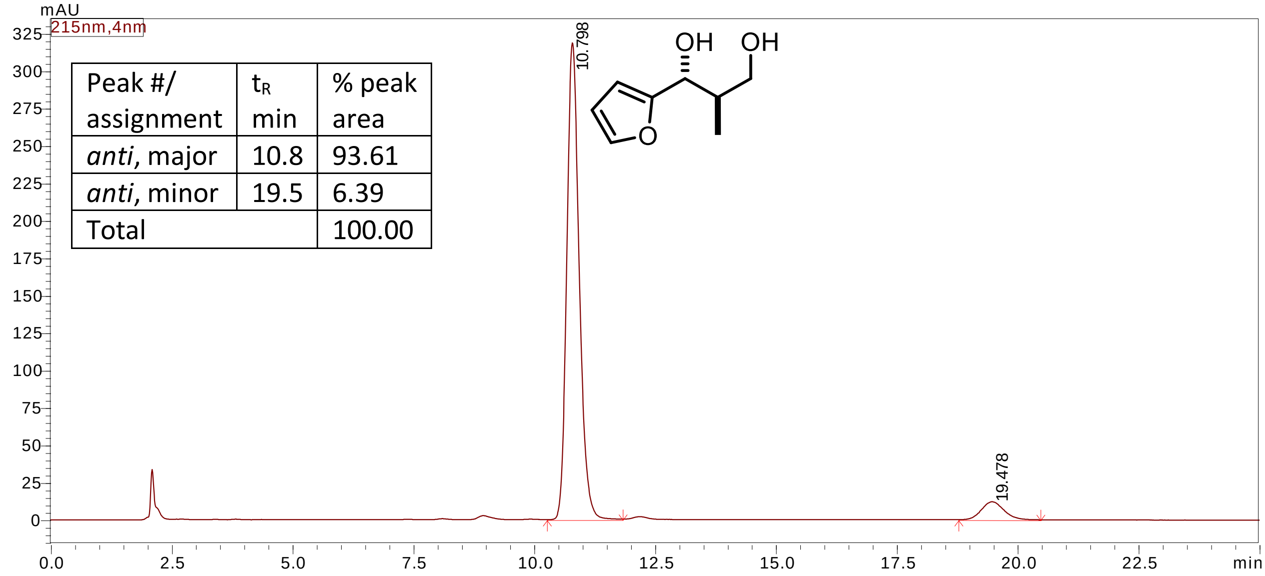

Peak #/ assignment	t_R min	% peak area
anti, major	10.8	93.61
anti, minor	19.5	6.39
Total		100.00

HPLC (IC-3, *n*-heptane/*i*-PrOH=90:10, 0.5 ml/min 298 K, 234 nm):

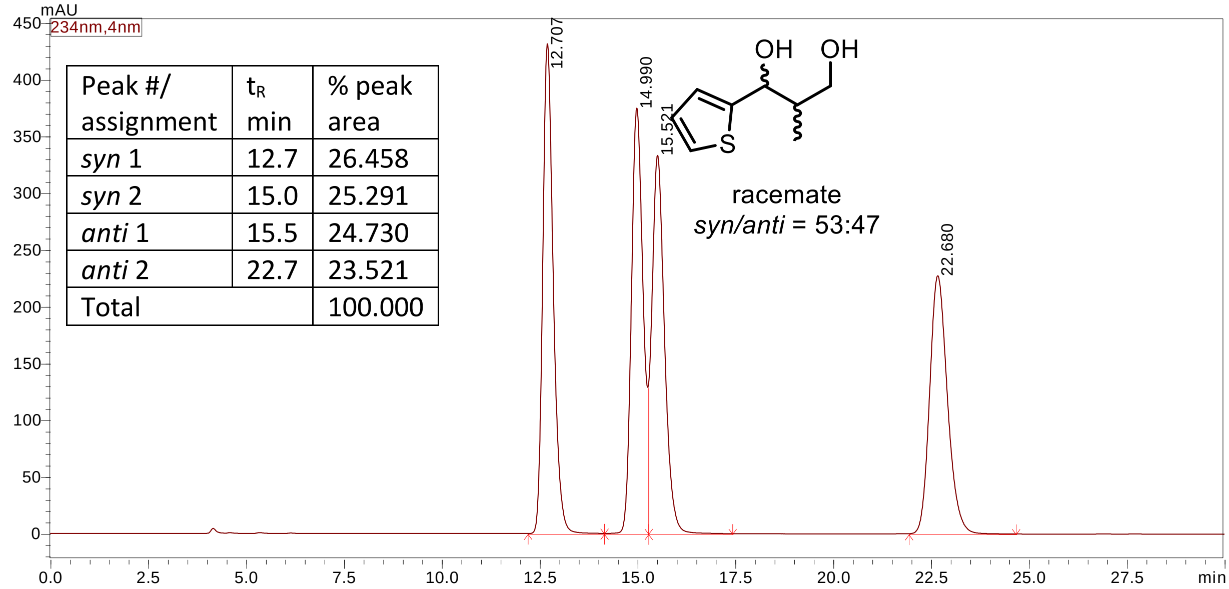

Peak #/ assignment	t_R min	% peak area
syn 1	12.7	26.458
syn 2	15.0	25.291
anti 1	15.5	24.730
anti 2	22.7	23.521
Total		100.000

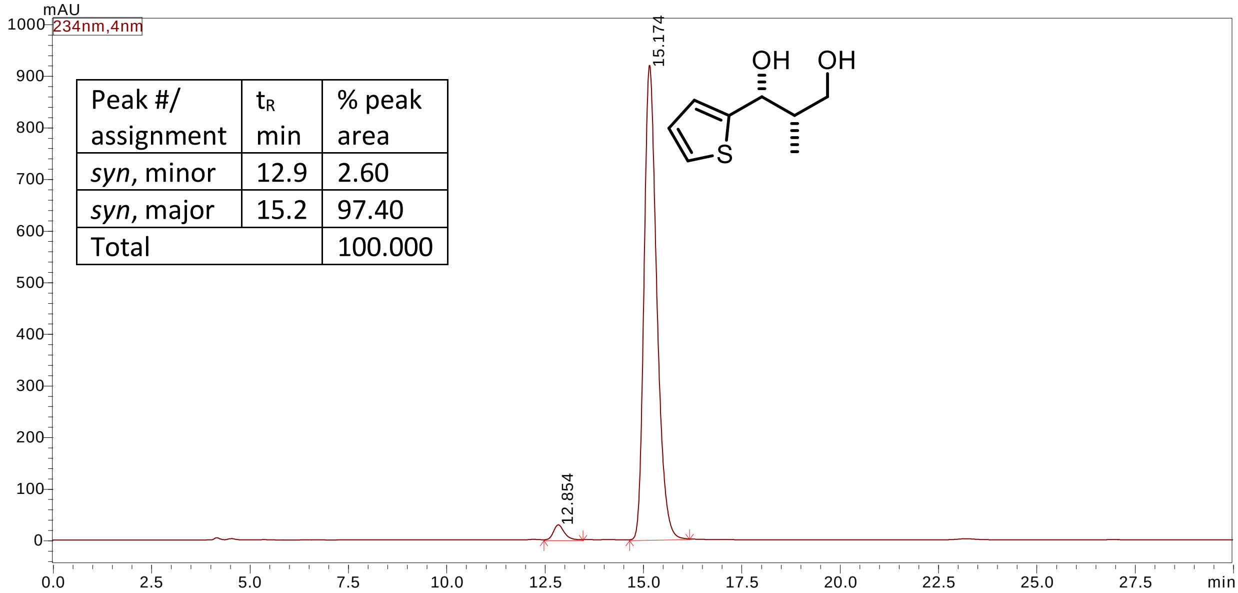

Peak #/ assignment	t_R min	% peak area
syn, minor	12.9	2.60
syn, major	15.2	97.40
Total		100.000

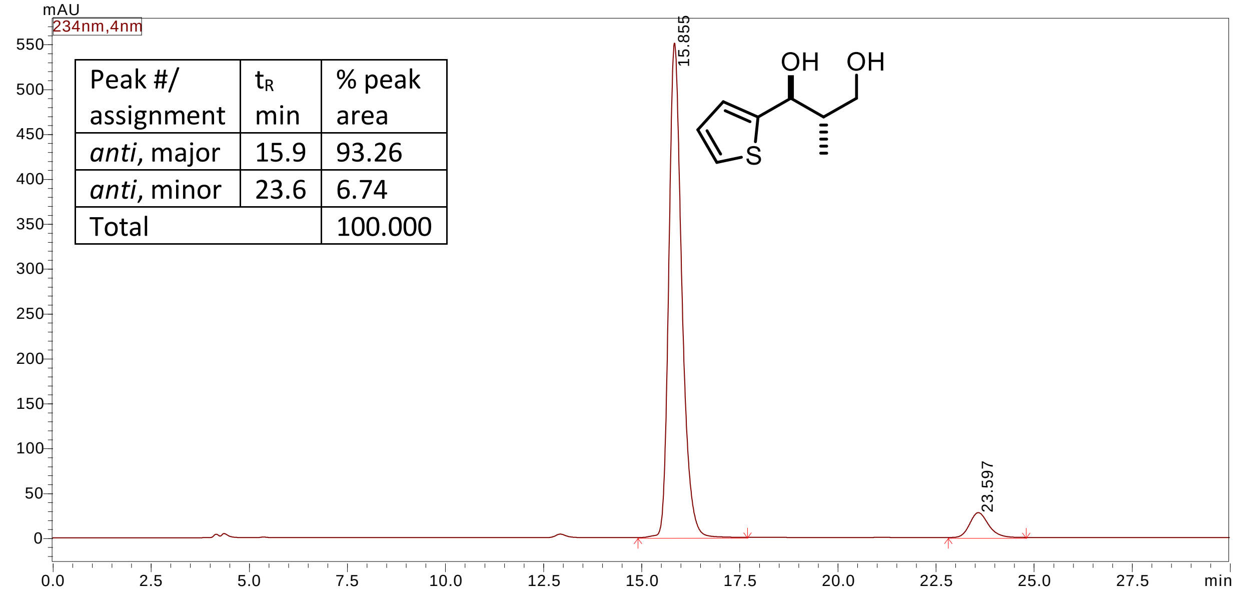

Peak #/ assignment	t_R min	% peak area
anti, major	15.9	93.26
anti, minor	23.6	6.74
Total		100.000

HPLC (IC-3, *n*-heptane/*i*-PrOH=95:5, 0.5 mL/min, 298 K, 220 nm):

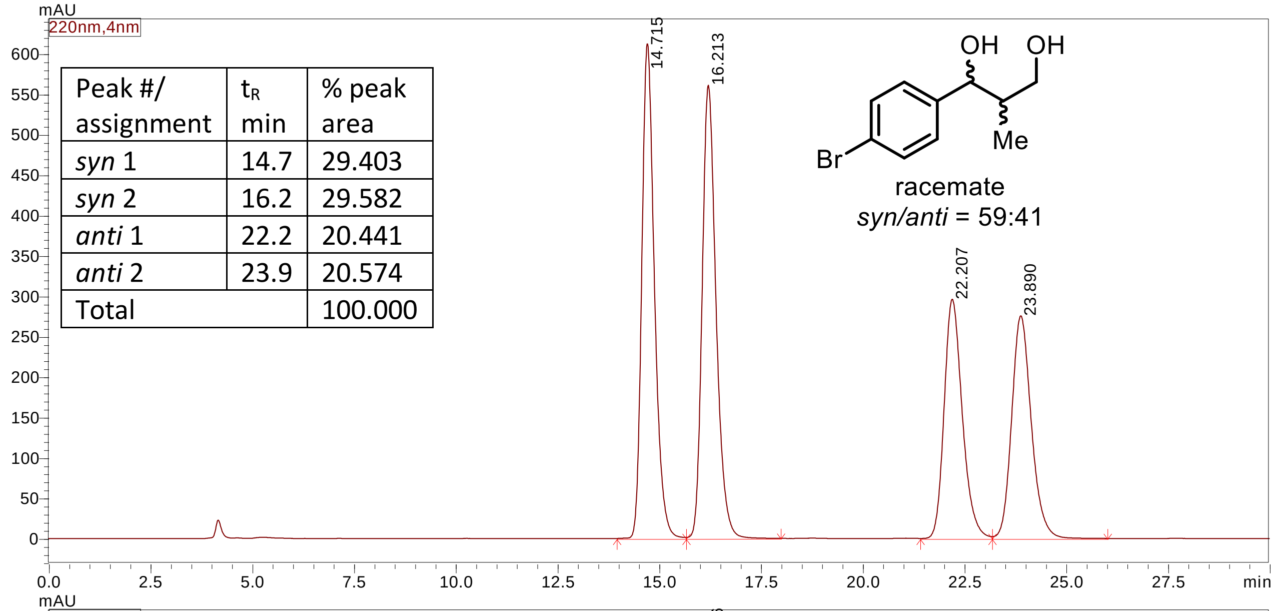

Peak #/ assignment	t_R min	% peak area
syn 1	14.7	29.403
syn 2	16.2	29.582
anti 1	22.2	20.441
anti 2	23.9	20.574
Total		100.000

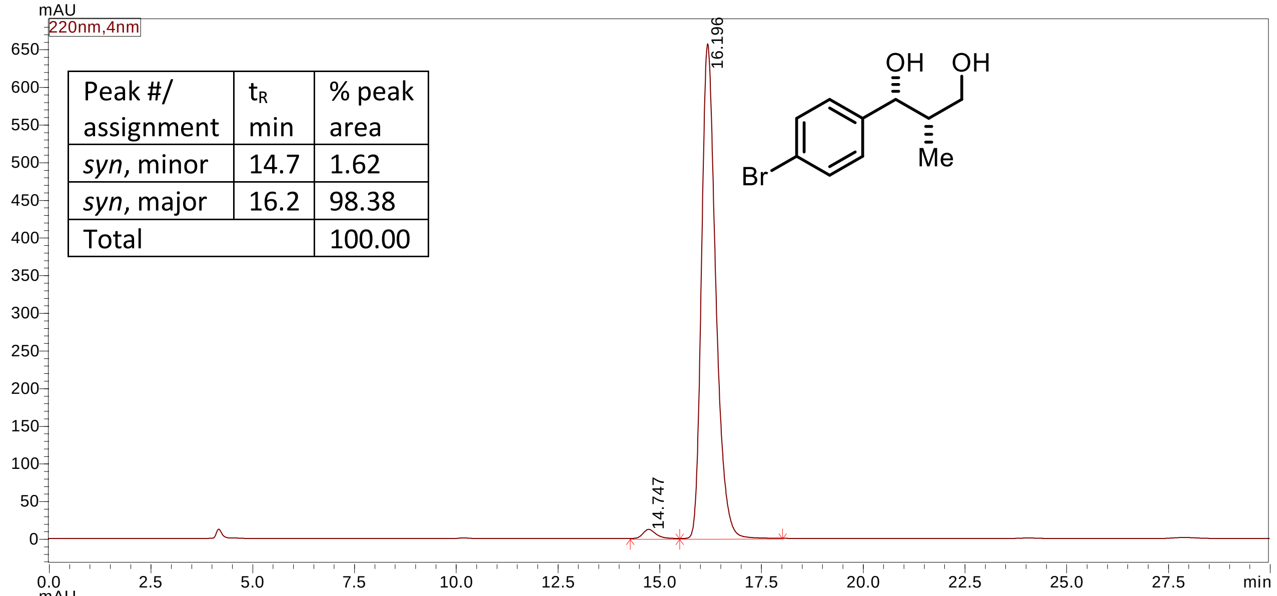

Peak #/ assignment	t_R min	% peak area
syn, minor	14.7	1.62
syn, major	16.2	98.38
Total		100.00

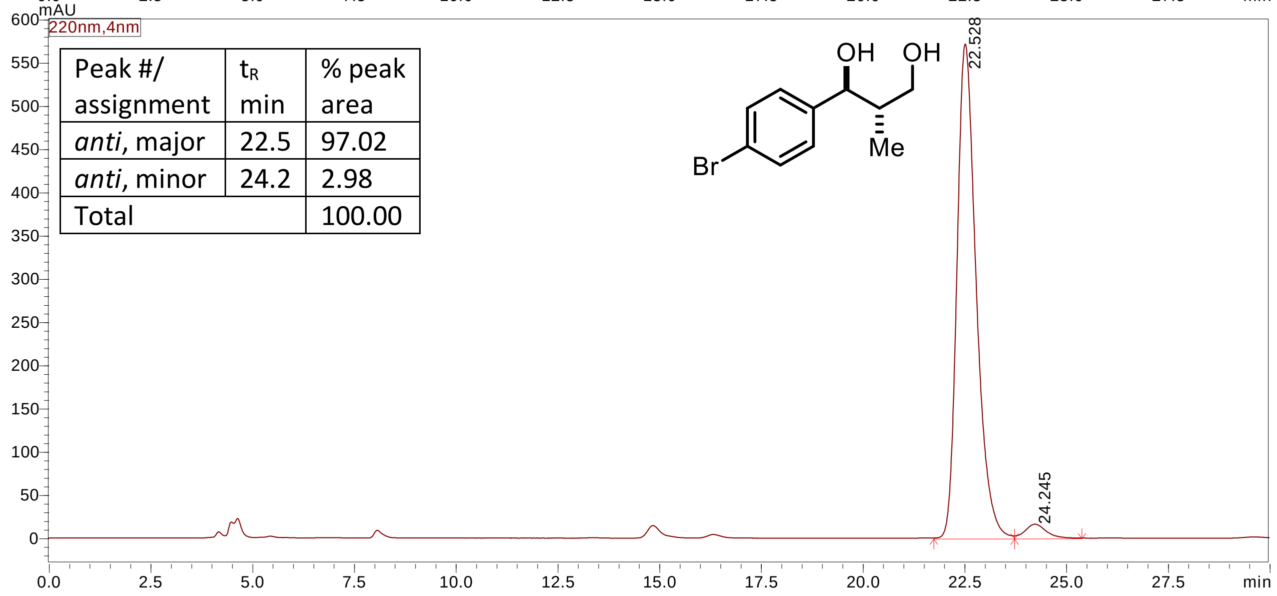

Peak #/ assignment	t_R min	% peak area
anti, major	22.5	97.02
anti, minor	24.2	2.98
Total		100.00

HPLC (IA-3, *n*-heptane/*i*-PrOH=90:10, 1.0 mL/min, 220 nm):

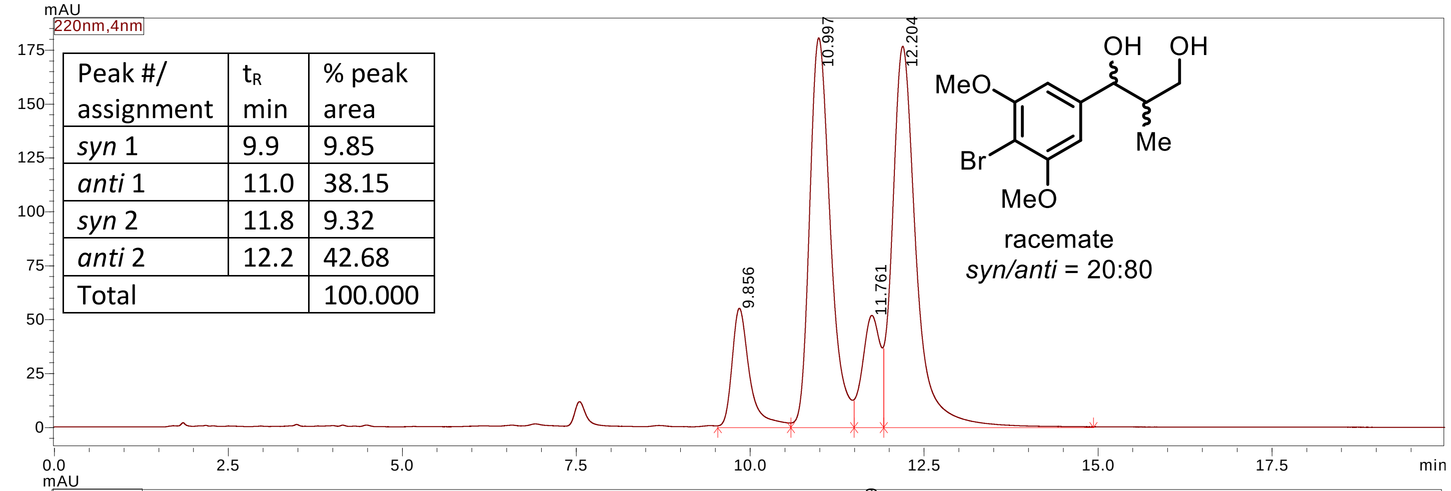

Peak #/ assignment	t_R min	% peak area
syn 1	9.9	9.85
anti 1	11.0	38.15
syn 2	11.8	9.32
anti 2	12.2	42.68
Total		100.000

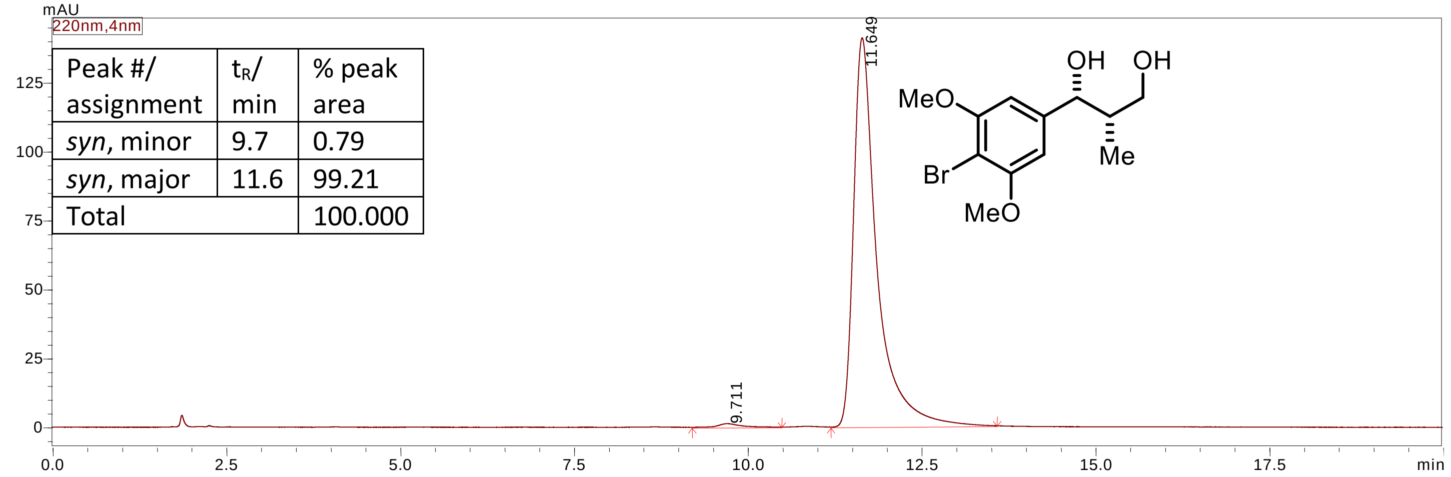

Peak #/ assignment	t_R/ min	% peak area
syn, minor	9.7	0.79
syn, major	11.6	99.21
Total		100.000

Achiral HPLC (RX SIL, *n*-heptane/*i*-PrOH=95:5, 1.0 mL/min, 220 nm) shows that, the syn/anti ratio is 99.64:0.36 in accordance with crude ^{1}H NMR, which also showed d.r.>99:1

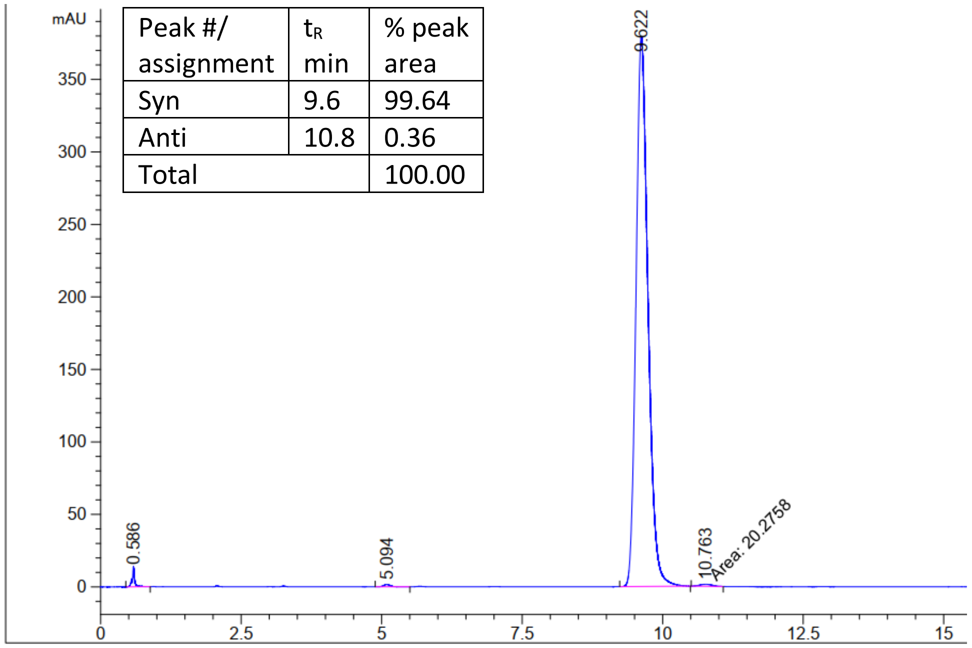

Peak #/ assignment	t_R min	% peak area
Syn	9.6	99.64
Anti	10.8	0.36
Total		100.00

HPLC (IB-N3, *n*-heptane/*i*-PrOH=95:5, 1.0 mL/min, 220 nm):

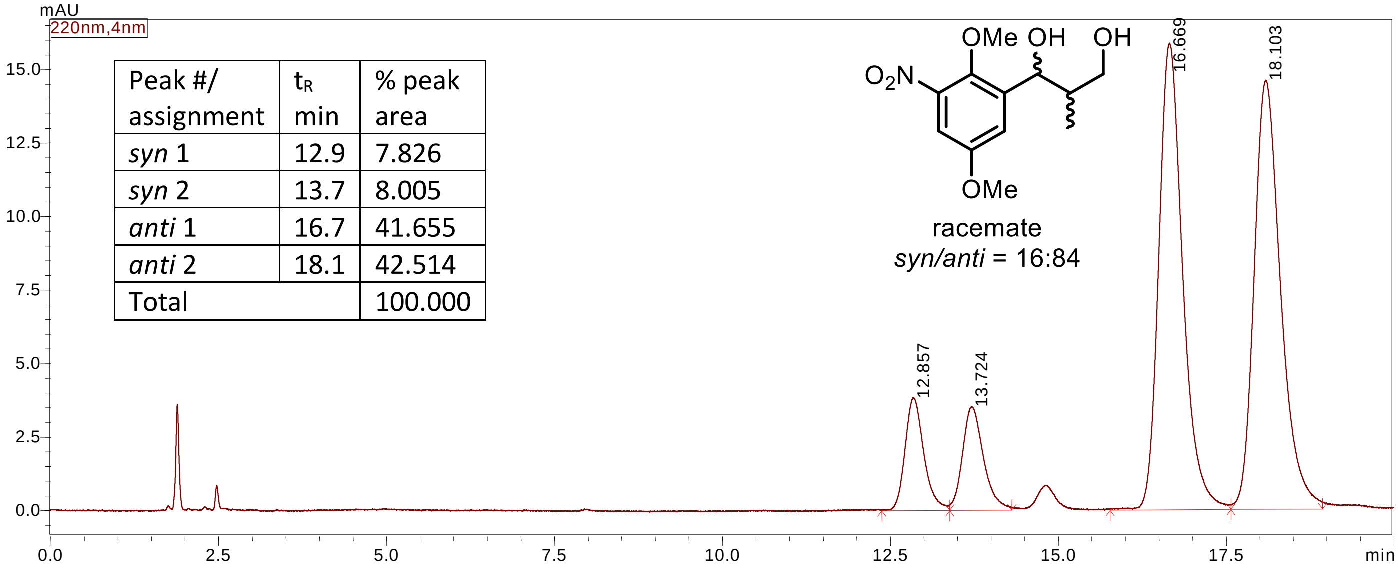

Peak #/ assignment	t_R min	% peak area
syn 1	12.9	7.826
syn 2	13.7	8.005
anti 1	16.7	41.655
anti 2	18.1	42.514
Total		100.000

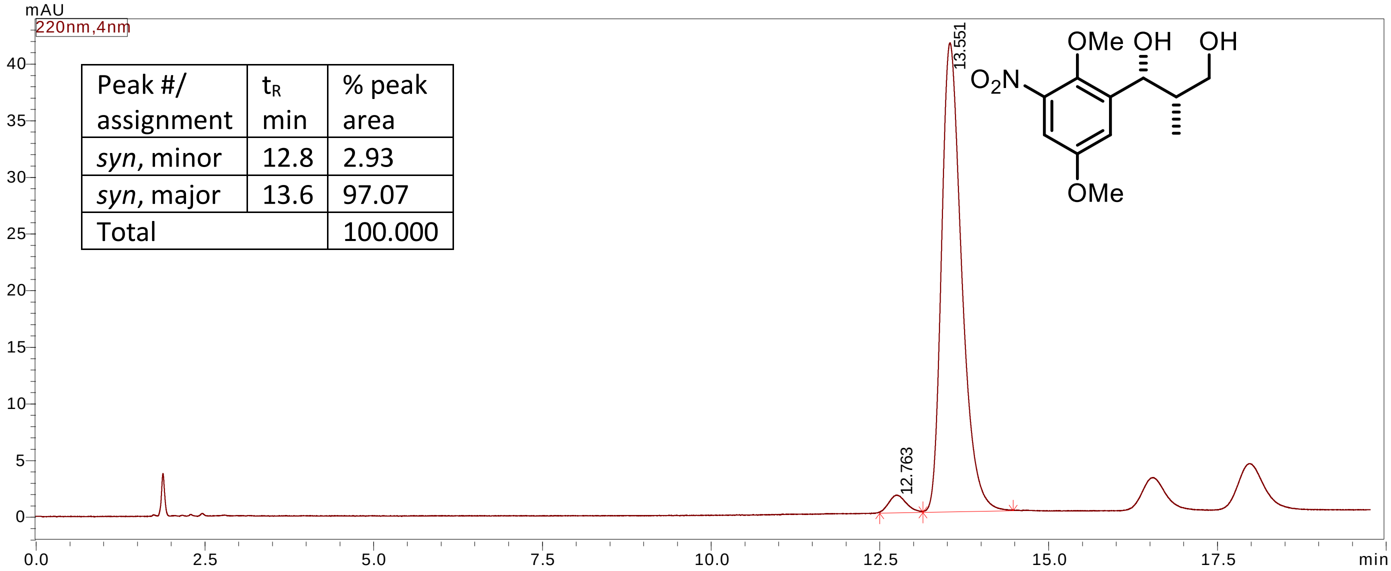

Peak #/ assignment	t_R min	% peak area
syn, minor	12.8	2.93
syn, major	13.6	97.07
Total		100.000

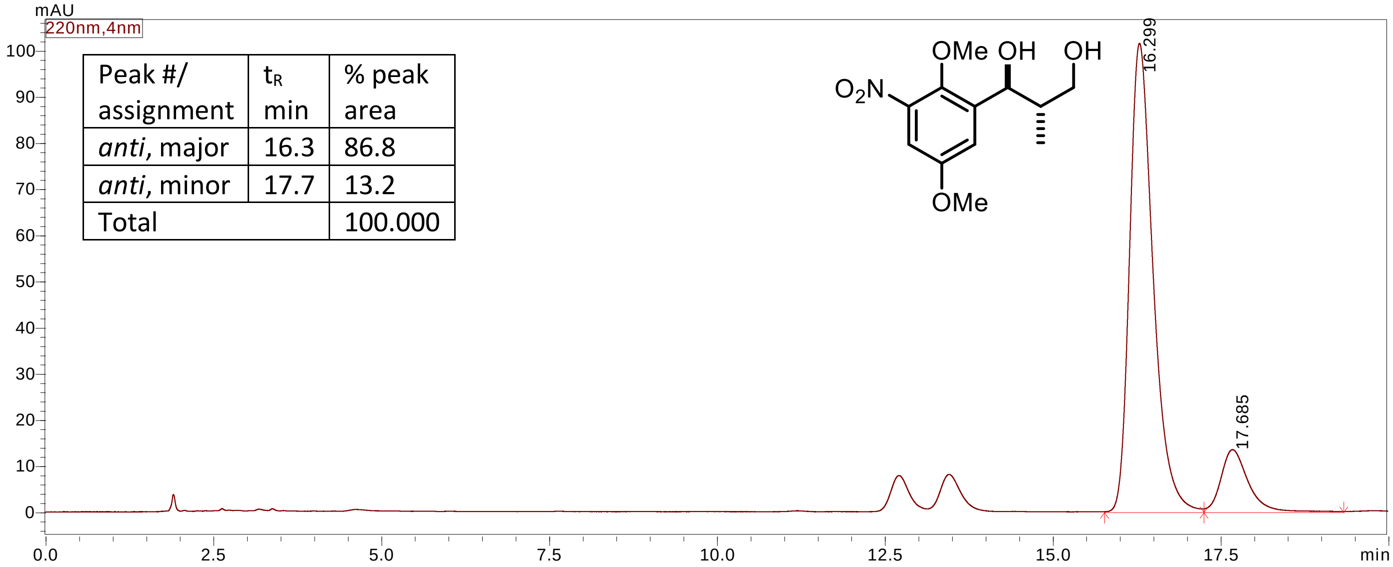

Peak #/ assignment	t_R min	% peak area
anti, major	16.3	86.8
anti, minor	17.7	13.2
Total		100.000

HPLC (IC-3, *n*-heptane/*i*-PrOH=90:10, 1.0 mL/min, 298 K, 220 nm):

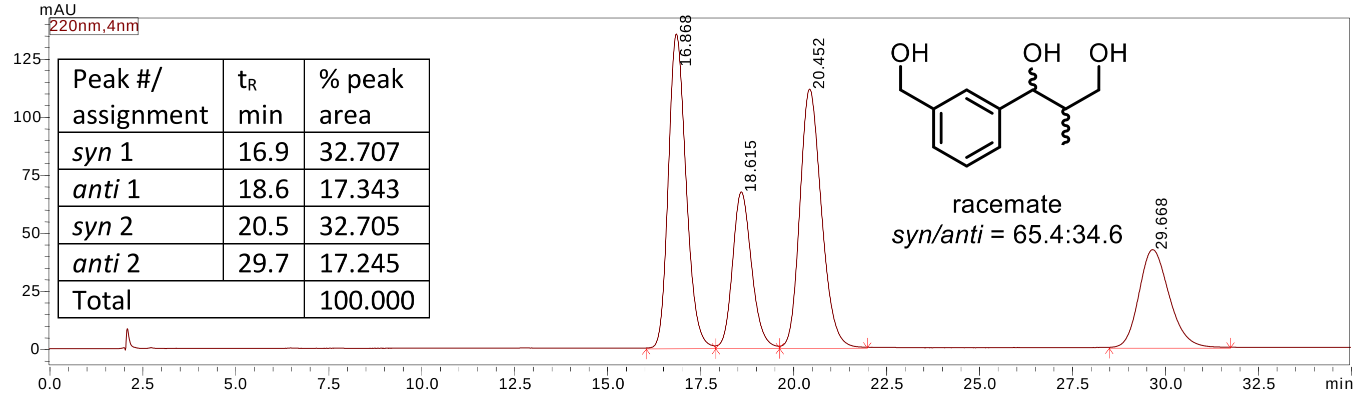

Peak #/ assignment	t_R min	% peak area
syn 1	16.9	32.707
anti 1	18.6	17.343
syn 2	20.5	32.705
anti 2	29.7	17.245
Total		100.000

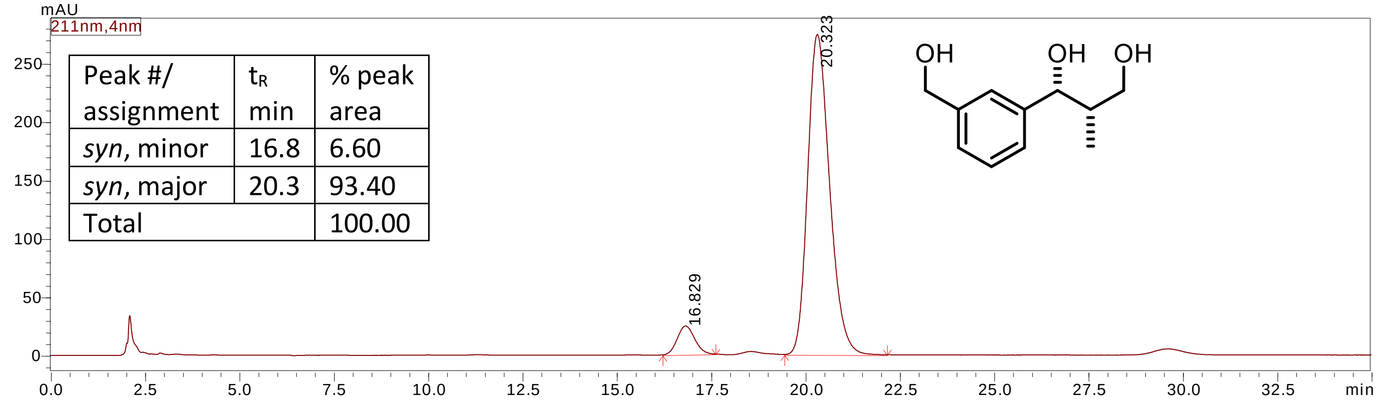

Peak #/ assignment	t_R min	% peak area
syn, minor	16.8	6.60
syn, major	20.3	93.40
Total		100.00

HPLC (Amycoat RP, Acetonitrile/Water = 40:60, 1.0 mL/min, 298 K, 220 nm):

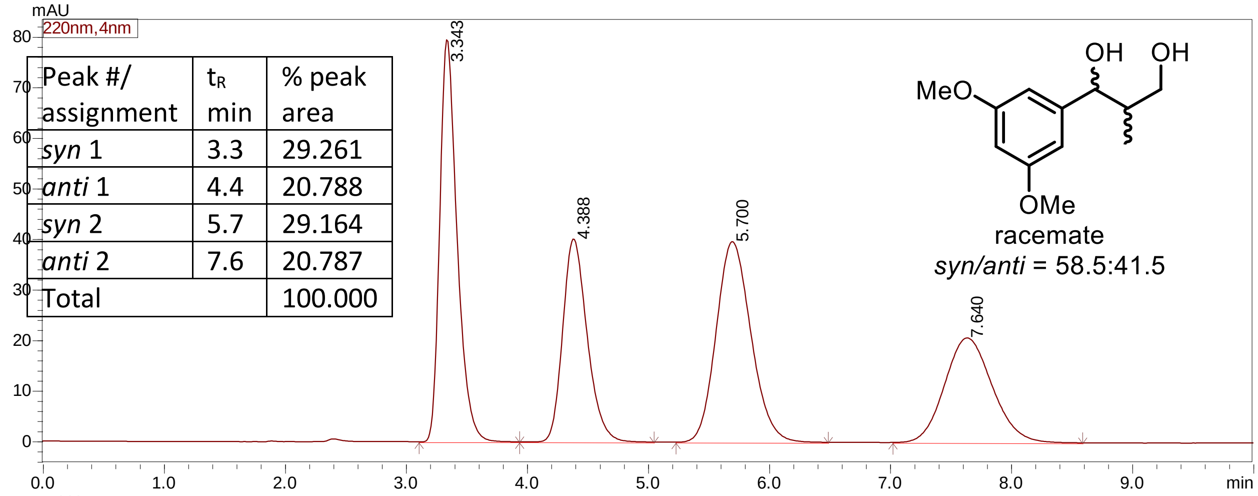

Peak #/ assignment	t_R min	% peak area
syn 1	3.3	29.261
anti 1	4.4	20.788
syn 2	5.7	29.164
anti 2	7.6	20.787
Total		100.000

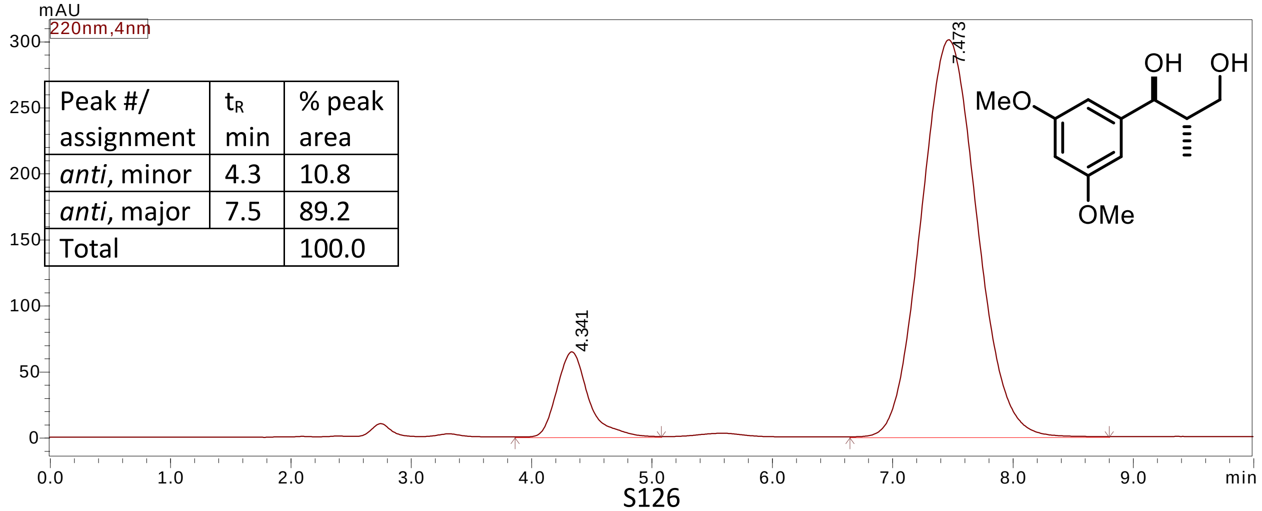

Peak #/ assignment	t_R min	% peak area
anti, minor	4.3	10.8
anti, major	7.5	89.2
Total		100.0

HPLC (IC-3, *n*-heptane/*i*-PrOH = 90:10, 1.0 mL/min, 298 K, 220 nm):

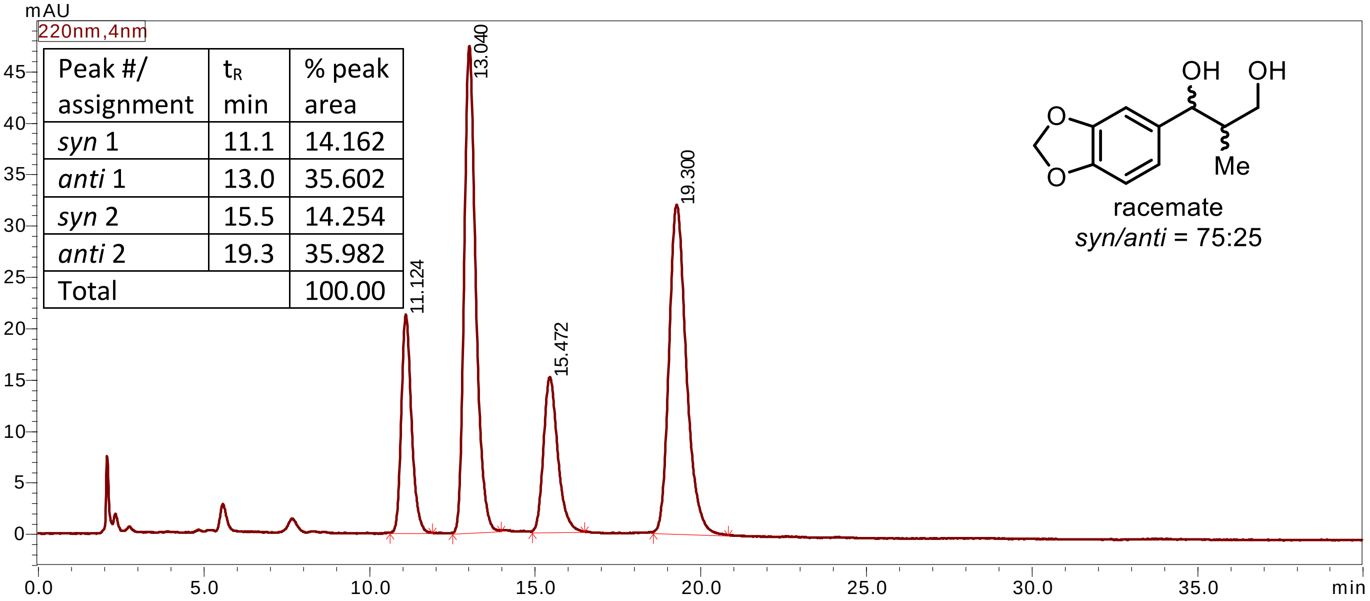

Peak #/ assignment	t_R min	% peak area
syn 1	11.1	14.162
anti 1	13.0	35.602
syn 2	15.5	14.254
anti 2	19.3	35.982
Total		100.00

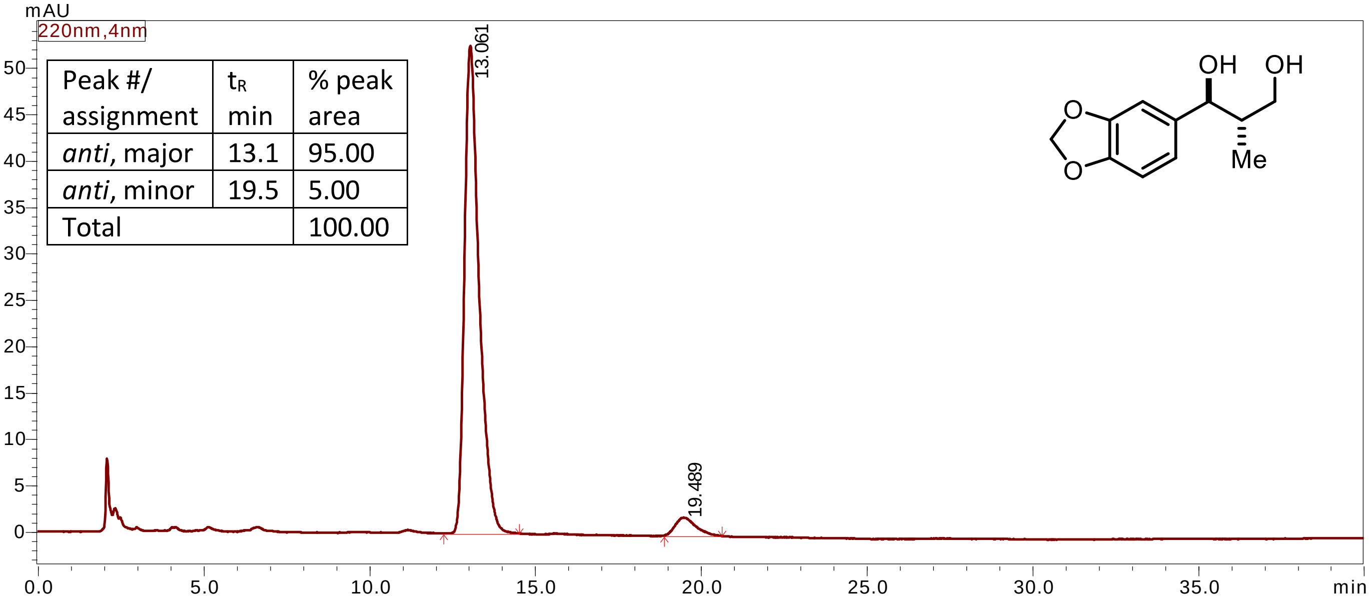

Peak #/ assignment	t_R min	% peak area
anti, major	13.1	95.00
anti, minor	19.5	5.00
Total		100.00

HPLC (IC-3, *n*-heptane/*i*-PrOH = 90:10, 0.3 mL/min, 298 K, 209 nm):

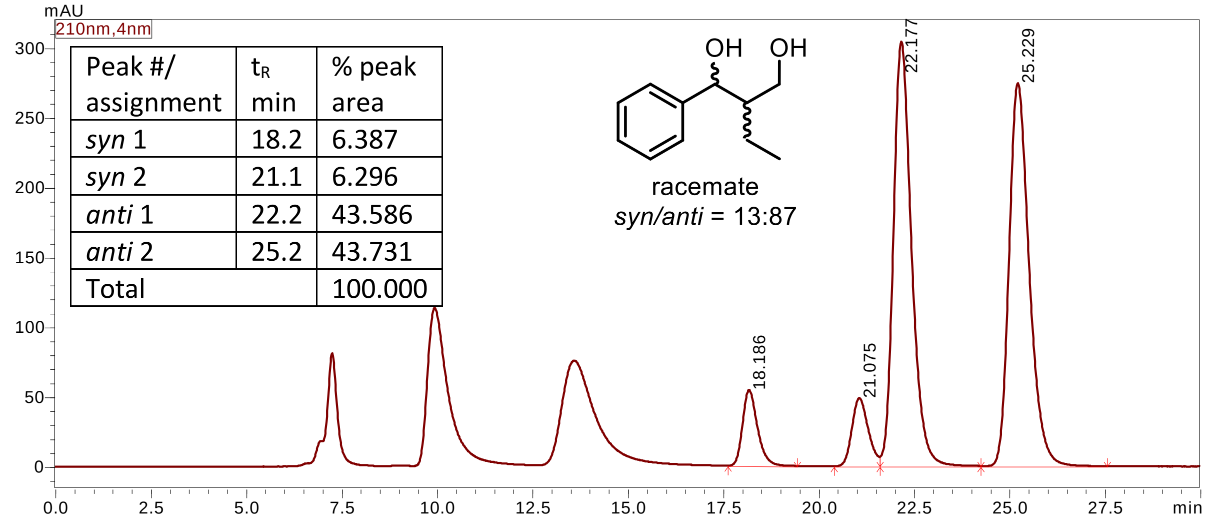

Peak #/ assignment	t_R min	% peak area
syn 1	18.2	6.387
syn 2	21.1	6.296
anti 1	22.2	43.586
anti 2	25.2	43.731
Total		100.000

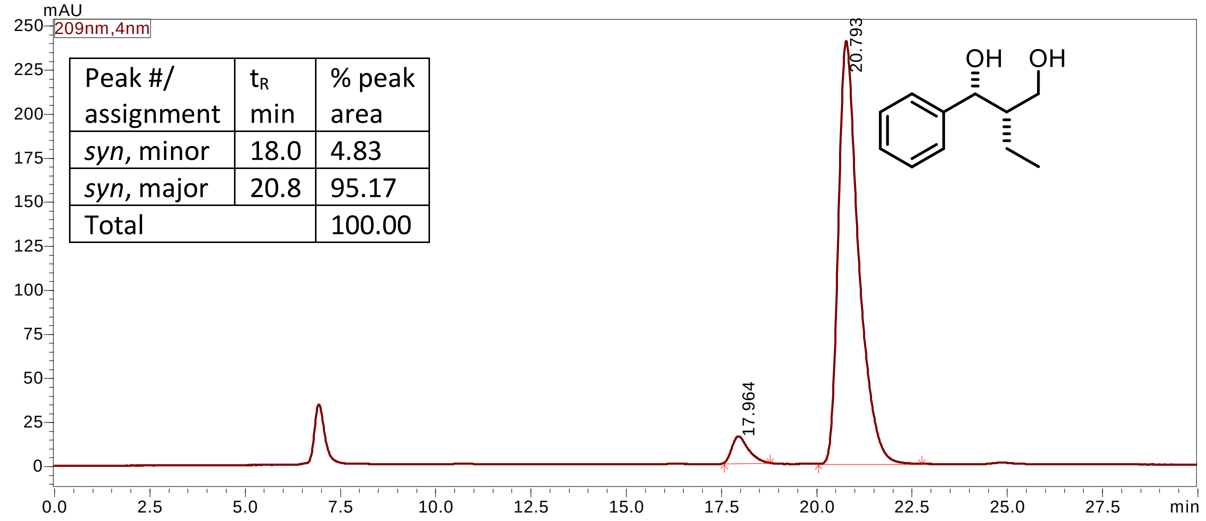

Peak #/ assignment	t_R min	% peak area
syn, minor	18.0	4.83
syn, major	20.8	95.17
Total		100.00

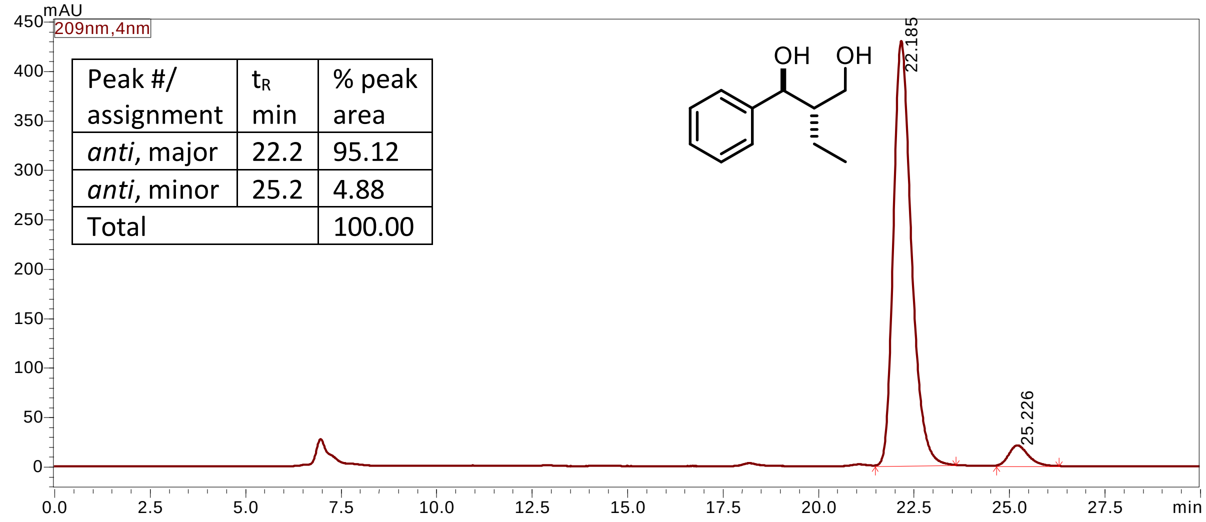

Peak #/ assignment	t_R min	% peak area
anti, major	22.2	95.12
anti, minor	25.2	4.88
Total		100.00

HPLC (IC-3, *n*-heptane/*i*-PrOH 90:10, 1.0 mL/min, 298 K, 209 nm):

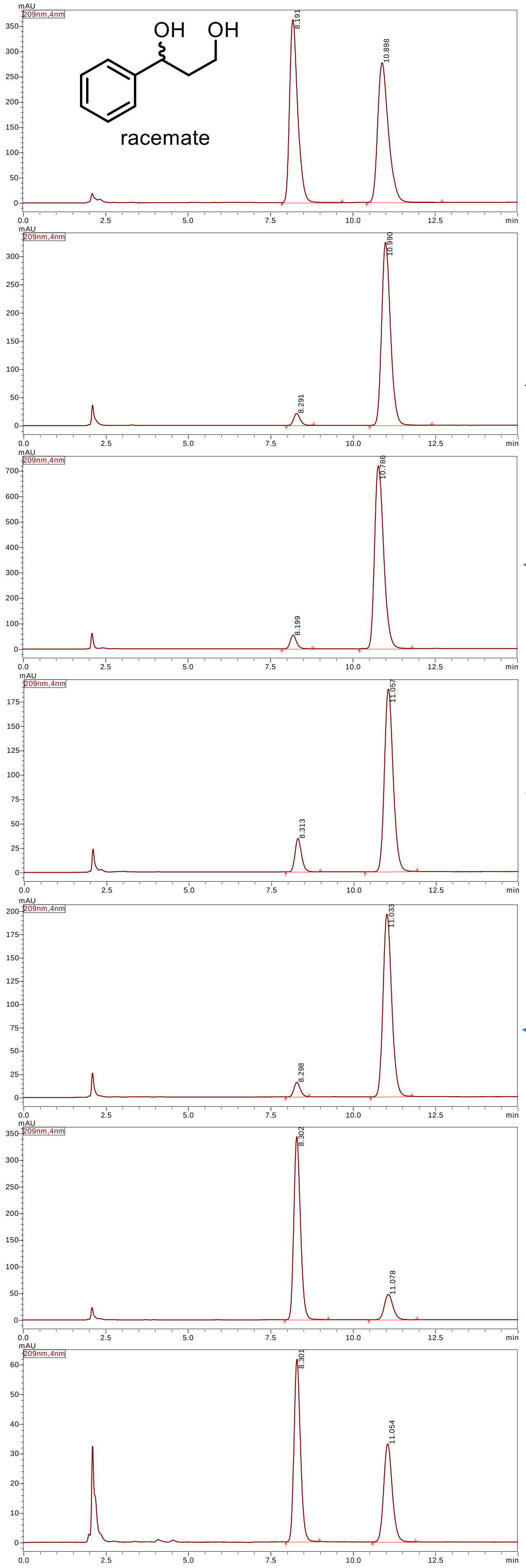

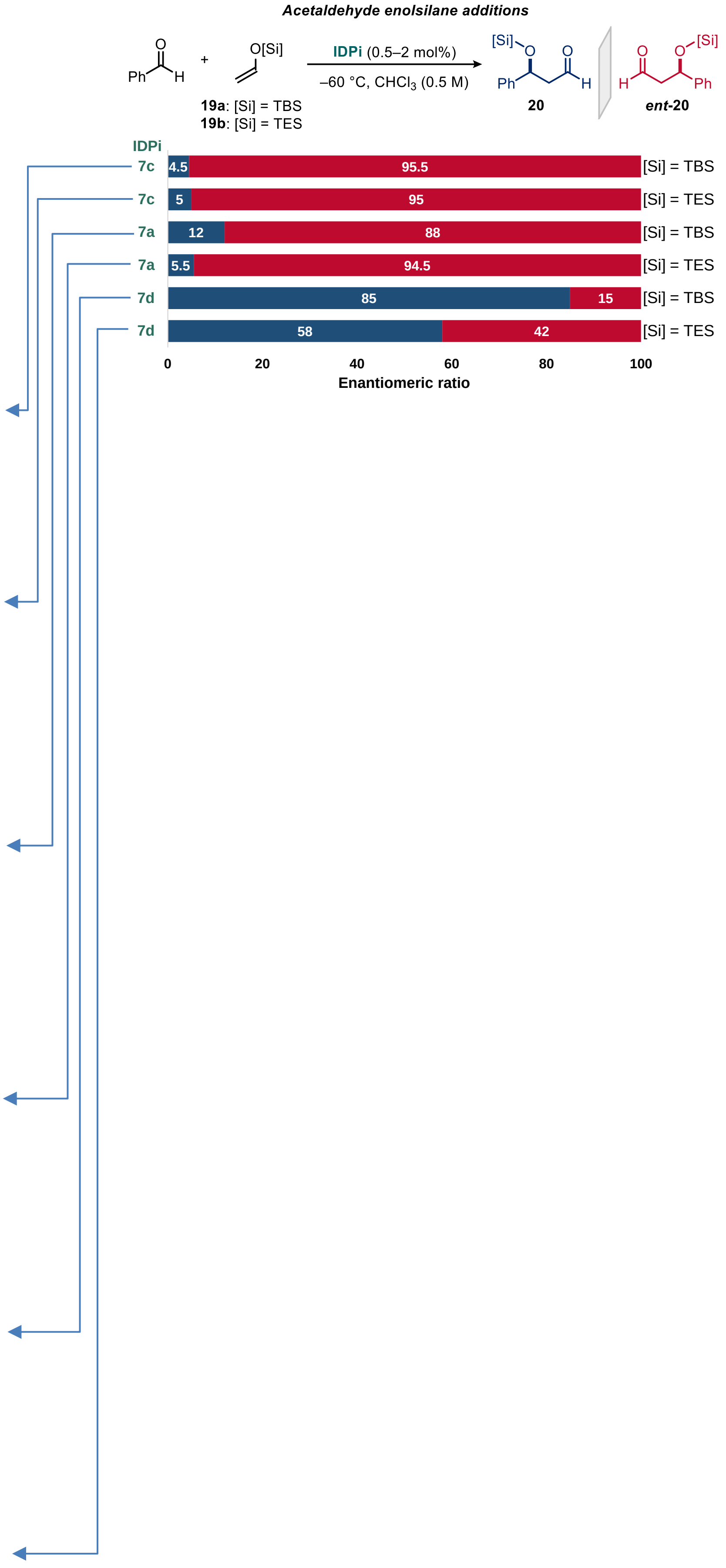

Computational Studies

Method

Possible TS conformations were explored by the artificial force induced reaction (AFIR) method[20] implemented in the global route reaction mapping (GRRM) program.[21] An exhaustive manual conformational search has been performed on possible catalyst substrate orientations at GFN2-xTB level of theory.[22] Molecular geometries were optimized using ONIOM(PBE-D3/6-31G(d): PBE-D3/3-21G) level of theory.[23] Thermal free energy corrections have been performed using Gaussian16 program.[24] Transition state structures were verified by the presence of a single imaginary vibrational frequency. All Single point calculations presented in this paper were carried out with a development version of the ORCA suite of programs base on version 4.2,[25] at B3LYP/def2-TZVP,[26,27] and B3LYP-D3(BJ)/def2-TZVP[28,29] level respectively. The RIJCOSX approximation[30] was used as well as the def2/J auxiliary basis set for all atoms. Solvation effect has been accounted by using CPCM (Chloroform) solvation model,[31] as implemented in ORCA. Distortion-interaction study has been undertaken to qualitatively understand the reason behind stereoinduction.[32] In order to qualitatively understand the outcome of analogous acetaldehyde derived enol silane mediated aldol reaction, fragmentation based analysis has been performed.[33] The molecular structures were visualized and overlay of different structures has been performed with the Chimera program.[34] At the obtained TS, non-covalent interactions and repulsive surfaces were visualized with NCIPlot[35] and VMD programs.[36]

Results and Discussion

The goal of our computational analysis is to understand the underlying reason behind the observed stereoselectivity of our newly developed enantio- and diastereoselective Mukaiyama aldol additions of propionaldehyde derived enolsilanes, and to decipher the origin of the observed intriguing reversal of the face selectivity in the presence of two similar catalysts. We computationally analyzed the two reactions defined in Scheme S1. In the computational study, **7a** and **7d** were used for simplicity and better comparison. The C–C bond formation step is considered as the enantio- and diastereo-determining step, and four possible conformers for each stereoisomer were investigated.

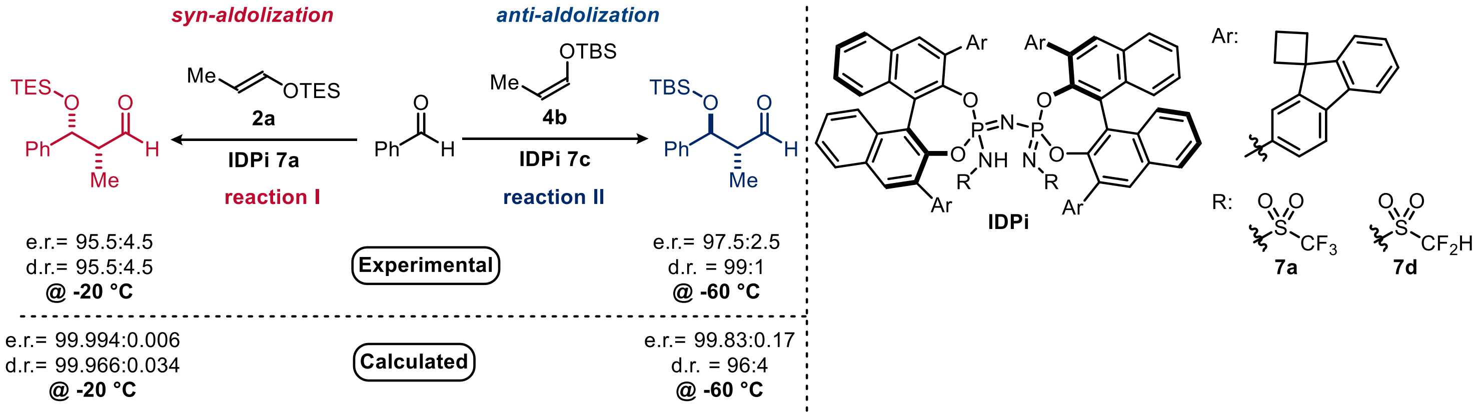

Scheme S1. Summary of substrates and catalysts used in the computational studies.

A good qualitative agreement to that of the experimental results were obtained for both reactions. In both *syn*- and *anti*-selective aldol reactions, there is a significant contribution of London-dispersion for the stabilization of conformers, according to the visualization by NCI plot. Visual depictions of some key non-covalent interactions are summarized in the figures below (Fig S6 for *syn*-aldolization, Fig S7 for *anti*-aldolization, respectively).

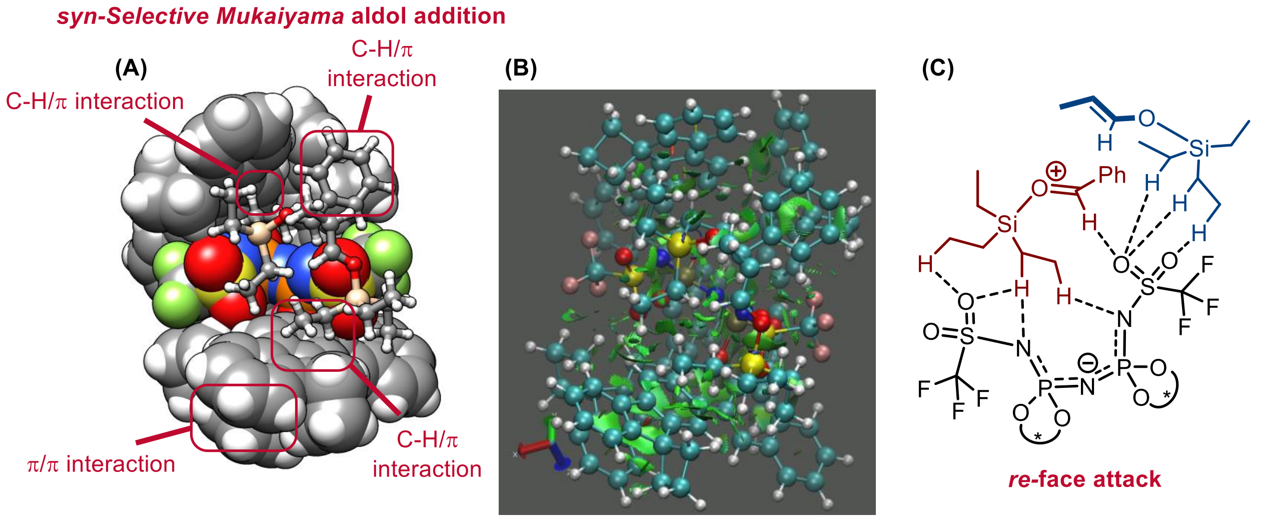

Figure S6. (A) Overview of key non-covalent interactions in the transition state corresponding to the major enantiomer in the *syn*-selective aldol reaction. **(B)** Plot of noncovalent interactions in the transition state structure leading to the major enantiomer. The isosurface value is 0.3. The electron density was computed at the PBE-D3/def2SVP level of theory. (**C**) Representative non-covalent interactions are depicted based on the visualization by NCI plot.

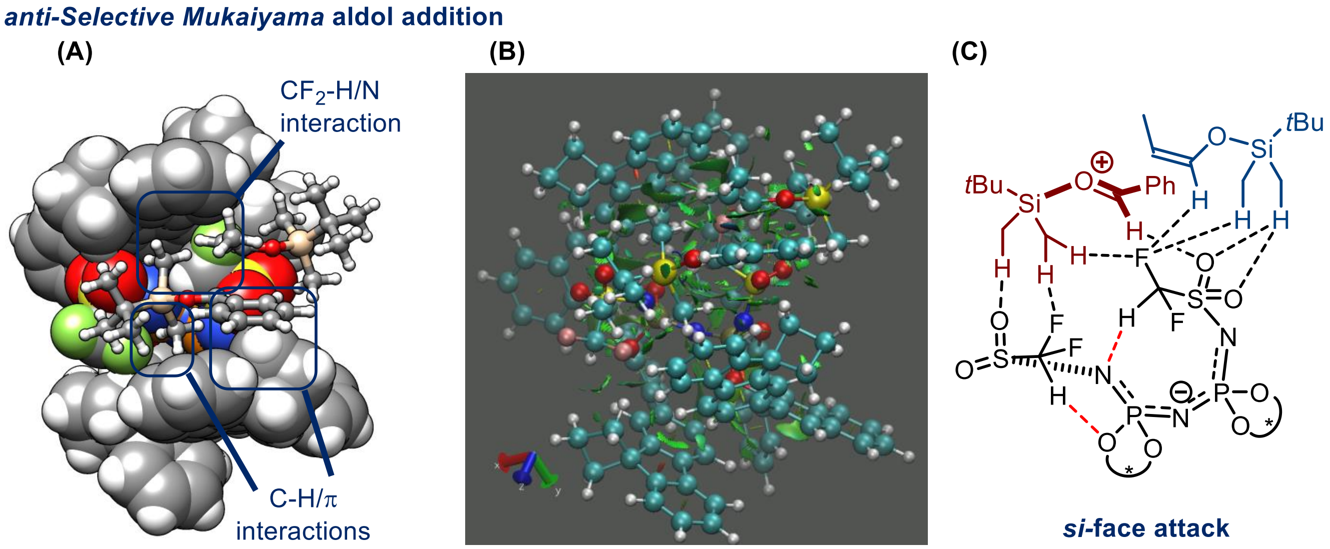

Figure S7. (A) Overview of key non-covalent interactions in the transition state corresponding to the major enantiomer in the *anti*-selective aldol reaction. **(B)** Plot of noncovalent interactions in the transition state structure leading to the major enantiomer. The isosurface value is 0.3. The electron density was computed at the PBE-D3/def2SVP level of theory. (**C**) Representative non-covalent interactions are depicted based on the visualization by NCI plot.

For a better comparison between two major transition states of *syn*- and *anti*-selective additions, the cavity sizes of catalysts are provided by calculating the distances and angles as depicted in Figure S8. The average positions of each highlighted fluorenyl groups were used to represent the substituents.

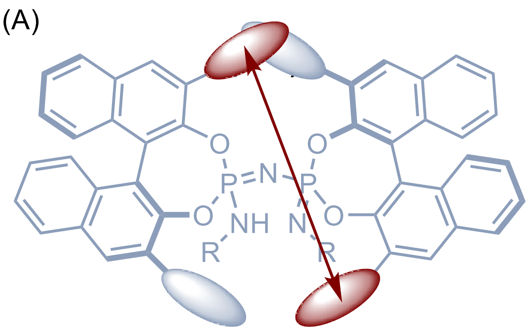

Figure S8. (A) Distance represents the catalyst pocket size for comparison. In the TS of *syn*-selective addition, catalyst counteranion had a larger pocket with 11.9 Å than that of the *anti*-selective addition with 9.9 Å. **(B)** Angles represent catalyst pocket size for comparison. In the TS of *syn*-selective Mukaiyama aldol addition counteranion of catalyst **7a** had a more open, wider pocket with 103.7° compared to that of the *anti*-selective addition.

Next, we investigated the origin of enantio- and diastereoselectivities (Figure S9 for *syn*- and S10 for *anti*-transition states). In the *syn*-selective Mukaiyama-aldol reaction, *re*-face attack is preferred; enolsilane **2a** with bulky TES-group approaches from the less sterically demanding side and the C-H/π interactions between the aryl moiety of benzaldehyde and the spirocyclic methylene groups of the fluorenyl substituent stabilize the transition state, in addition to blocking the *si*-face (Figure S9A). In contrast, in the TS leading to the minor enantiomer, the nucleophile has to approach from the crowded side, and the C-H/π interactions between the electrophile and the catalyst are missing (Figure S9B). In the TS leading to diastereomers, sterically demanding silicon groups are located close to each other, resulting in the loss of multiple non-covalent interactions, increasing the reaction barrier (Figure S9C).

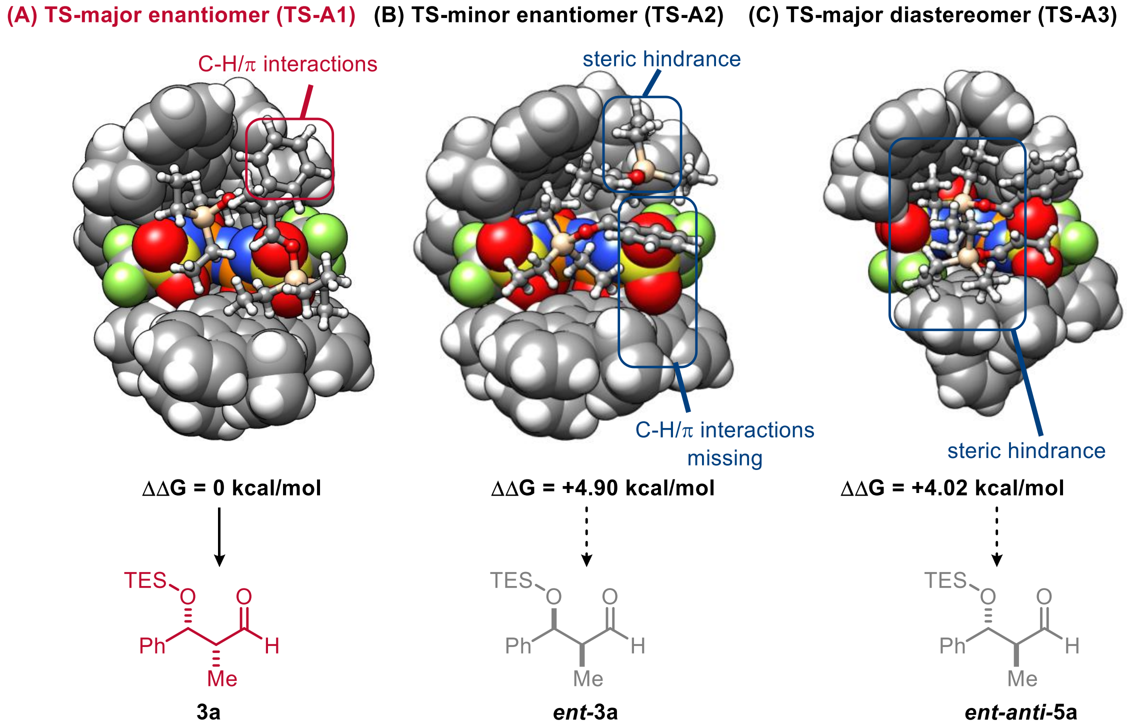

Figure S9. Origin of enantioselectivity and diastereoselectivity for the *syn*-selective aldol reaction (Reaction I), computed at the B3LYP-D3(BJ)/def2-TZVP + CPCM(chloroform)//ONIOM(PBE-D3/6-31G(d): PBE-D3/3-21G) level of theory. **TS-A4**, leading to ***anti*-5a** not shown.

In the *anti*-selective Mukaiyama-aldol addition, *si*-face attack is preferred; The CF_2-H/N intramolecular hydrogen bonding lead to a narrower catalyst pocket. Hence the smaller (*Z*)-enolsilane nucleophile with a bulky TBS-group approaches preferentially from the less sterically hindered side, which is opposite from the case of the *syn*-selective reaction (Figure S10A). When the nucleophile approaches from the opposite side, the fluorenyl group

has to be rotated to expand the pocket, and the C-H/π interactions between the phenyl group of benzaldehyde and the catalyst would be lost (Figure S10B). In the TS leading to diastereomers, in addition to severe steric hindrance between the alkyl groups of the silane and the catalyst, CF_2-H/N interaction and C-H/O interaction between benzaldehyde and the catalyst would be lost, resulting in increasing the barrier (Figure S10C).

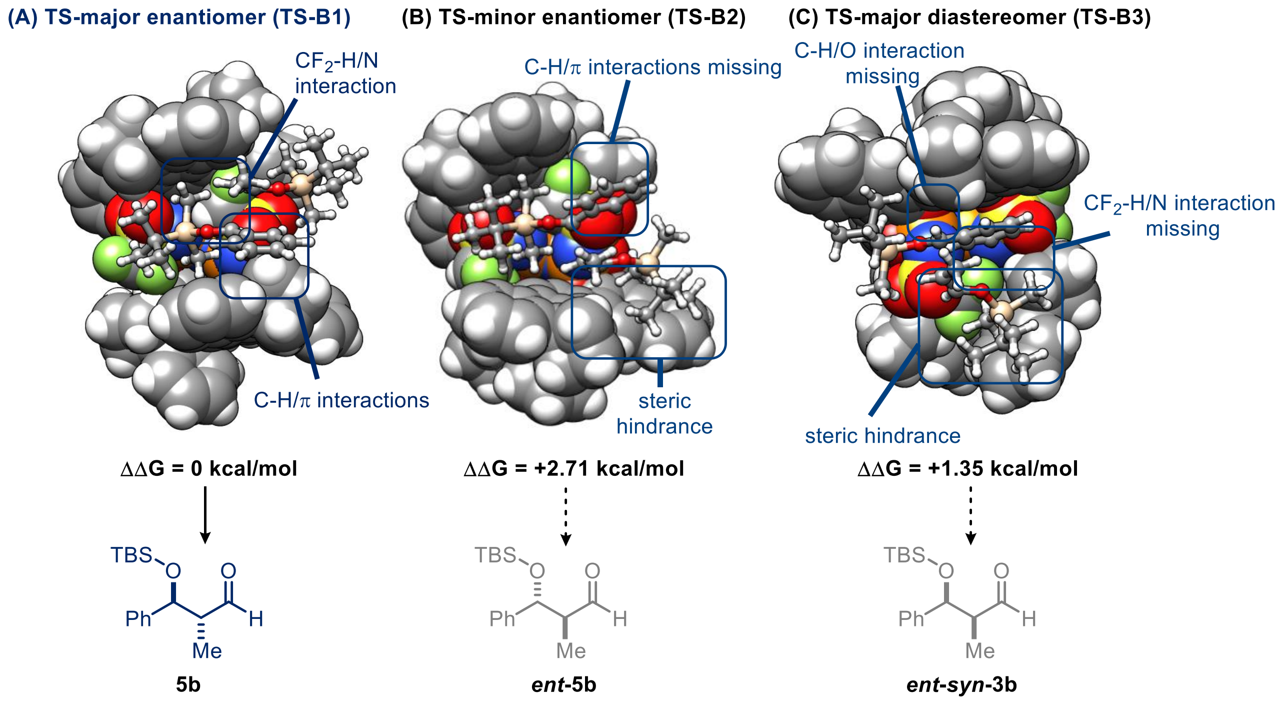

Figure S10. Origin of enantioselectivity and diastereoselectivity for the *anti*-selective aldol reaction (Reaction II), computed at the B3LYP-D3(BJ)/def2-TZVP + CPCM(chloroform)//ONIOM(PBE-D3/6-31G(d): PBE-D3/3-21G) level of theory. **TS-B4**, leading to **3b** not shown.

In addition to studying the origins of stereoselectivity, we investigated the effect of the London-dispersion interactions using DFT-D3 method developed by S. Grimme and co-workers. As expected, DFT-D3 with Becke-Johnson[37] rational damping provided the relative TS energies, which are consistent with the experimental results (Table S10). In contrast, without a dispersion correction, the TS energies can no longer explain the experimental observation (Table S11). These results suggest large contribution of London dispersion in the transition states, which provides a good agreement with the observation by NCI Plot. The trend is similar in *anti*-selective aldol reaction (Table S12, S13).

Table S10. TS corresponding to *syn*-selective aldol addition (reaction I). Energies at the *B3LYP-D3(BJ)/def2-TZVP + CPCM(chloroform)//*ONIOM(PBE-D3/6-31G(d): PBE-D3/3-21G) *level of theory. Thermal energy correction was performed at 253.150 K at the* ONIOM(PBE-D3/6-31G(d): PBE-D3/3-21G) *level.*

TS No	Thermal corrections	*B3LYP-D3(BJ)/def2-TZVP* single point ($CHCl_3$ solv)	*Imaginary freq.*	Δ G(TS) Final (in hartree)	Relative TS Energy (kcal/mol)
TS-A1	1.936260	-8518.7199816	-129.50029	-8516.7837216	0.00
TS-A2	1.935702	-8518.71161370	-204.32262	-8516.7759117	+4.90 (Enant.)
TS-A3	1.934685	-8518.712005790	-128.15021	-8516.77732079	+4.02 (Diast.)
TS-A4	1.936293	-8518.711774135	- 87.9368477	-8516.77548113	+5.17 (Diast.)

Table S11. TS corresponding to *syn*-selective aldol addition (reaction I). Energies at the *B3LYP/def2-TZVP + CPCM(chloroform)//*ONIOM(PBE-D3/6-31G(d): PBE-D3/3-21G) *level of theory. Thermal energy correction was performed at 253.150 K at the* ONIOM(PBE-D3/6-31G(d): PBE-D3/3-21G) *level.*

TS No	Thermal corrections	*B3LYP/def2-TZVP* single point ($CHCl_3$ solv)	*Imaginary freq.*	Δ G(TS) Final (in hartree)	Relative TS Energy (kcal/mol)
TS-A1	1.936260	-8517.78467635	-129.50029	-8515.84841635	+6.17
TS-A2	1.935702	-8517.77676097	-204.32262	-8515.84105897	+10.8
TS-A3	1.934685	-8517.79293760	-128.15021	-8515.8582526	0.00
TS-A4	1.936293	-8517.77828771	-87.9368477	-8515.84199471	+10.2

Table S12. TS corresponding to *anti*-selective aldol addition (reaction II). Energies at the *B3LYP-D3(BJ)/def2-TZVP + CPCM(chloroform)//*ONIOM(PBE-D3/6-31G(d): PBE-D3/3-21G) *level of theory. Thermal energy correction was performed at 213.150 K at the* ONIOM(PBE-D3/6-31G(d): PBE-D3/3-21G) *level.*

TS No	PBE RRHO corrections	*B3LYP-D3(BJ)/def2-TZVP* single point ($CHCl_3$ solv)	*Imaginary freq.*	Δ G(TS) Final (in hartree)	Relative TS Energy (kcal/mol)
TS-B1	1.986691	-8320.22171489	-26.30508329	-8318.23502389	0.00
TS-B2	1.986898	-8320.217600630	-89.15791803	-8318.23070263	2.71 (Enant.)
TS-B3	1.983390	-8320.21626716	-117.26757528	-8318.23287716	1.35 (Diast.)
TS-B4	1.982234	-8320.21371198	-61.25643207	-8318.23147798	2.22 (Diast.)

Table S13. TS corresponding to *anti*-selective aldol addition (reaction II). Energies at the *B3LYP/def2-TZVP + CPCM(chloroform)//* ONIOM(PBE-D3/6-31G(d): PBE-D3/3-21G) *level of theory. Thermal energy correction was performed at 213.150 K at the* ONIOM(PBE-D3/6-31G(d): PBE-D3/3-21G) *level.*

TS No	Thermal corrections	*B3LYP/def2-TZVP* single point ($CHCl_3$ solv)	*Imaginary Freq.*	Δ G(TS) Final (in hartree)	Relative TS Energy (kcal/mol)
TS-A1	1.986691	-8319.305540733	-26.30508329	-8317.31884973	+2.62
TS-A2	1.986898	-8319.283387386	-89.15791803	-8317.29648939	+16.6
TS-A3	1.983390	-8319.306411705	-117.26757528	-8317.32302171	0.00
TS-A4	1.982234	-8319.275610245	-61.25643207	-8317.29337625	+18.6

In order to further understand the reason of high stereoselectivities, we next performed the distortion-interaction analysis following Houk-Bickelhaupt protocol. Gas phase electronic energy of the optimized TS structures has been decomposed into the catalyst counteranion and both substrates (aldehyde and enol silane) at the *B3LYP-D3(BJ)/def2-TZVP level*. We observed that the distortion effect originating from the catalyst counteranion plays a crucial role in the enantioselectivity for both *syn*- and *anti*-selective aldol additions (Table S14.1 and Table S14.3). This is also evident from the overlay of the two counteranions derived from both enantiomers compared to the optimized catalyst counteranions (Fig S11). Notably, the extent of distortion is more prominent for the *anti*-aldol TS, presumably due to its more confined structure. On the other hand, diastereoselectivity (Table S14.2 and Table S14.4) is predominantly dependent on the interactions (C-H/π and C-H/O interactions for *syn*, while CF_2-H/N and C-H/O interactions for *anti*). Together, this analysis complements our previous analysis and offers a semi-quantitative measure of different factors controlling the stereoselectivity in this transformation.

Table S14.1. Distortion-interaction analysis (B3LYP-D3(BJ)/def2-TZVP) to identify reason behind stereoinduction in *syn*-selective aldol reaction (between enantiomers)

TS No	*B3LYP-D3(BJ)/def2-TZVP* single point (in hartree)	Relative Energy (kcal/mol) Δ E
TS-A1	-8518.688111	6.40
TS-A2	-8518.6779570	
Both Substrates(benzaldehyde+Nu) Only		
Subst_TS-A1	-1592.12890303	0.43
Subst_TS-A2	-1592.128209486	
Catalyst Only		
Cat_TS-A1	-6926.3942315	3.95
Cat_TS-A2	-6926.3880279	
Total Distortion	(predominant factor)	4.38
Total Interaction		2.02

Table S14.2. Distortion-interaction analysis (B3LYP-D3(BJ)/def2-TZVP) to identify reason behind stereoinduction in *syn*-selective aldol reaction (between diastereomers)

TS No	*B3LYP-D3(BJ)/def2-TZVP* single point (in hartree)	Relative Energy (kcal/mol) Δ E
TS-A1	-8518.688111	7.09
TS-A3	-8518.676849140829	
Substrates Only		
Subst_TS-A1	-1592.12890303	-4.26
Subst_TS-A3	-1592.135726166349	
Catalyst Only		
Cat_TS-A1	-6926.3942315	4.14
Cat_TS-A3	-6926.387666608739	
Total Distortion		-0.12
Total Interaction	(predominant factor)	7.21

Table S14.3. Distortion-interaction analysis (B3LYP-D3(BJ)/def2-TZVP) to identify reason behind stereoinduction in *anti*-selective aldol reaction (between enantiomers)

TS No	*B3LYP-D3(BJ)/def2-TZVP* single point (in hartree)	Relative Energy (kcal/mol) Δ E
TS-B1	-8320.1831266	1.31
TS-B2	-8320.181055543	
Both Substrates(benzaldehyde+Nu) Only		
Subst_TS-B1	-1592.1503915	0.22
Subst_TS-B2	-1592.15004008	
Catalyst Only		
Cat_TS-B1	-6727.87908673675	7.14
Cat_TS-B2	-6727.867702779	
Total Distortion	(predominant factor)	7.36
Total Interaction		-6.06

Table S14.4. Distortion-interaction analysis (B3LYP-D3(BJ)/def2-TZVP) to identify reason behind stereoinduction in *anti*-selective aldol reaction (between diastereomers)

TS No	*B3LYP-D3(BJ)/def2-TZVP* single point (in hartree)	Relative Energy (kcal/mol) Δ E
TS-B1	-8320.1834266	2.84
TS-B3	-8320.17889674	
Substrates Only		
Subst_ TS-B1	-1592.1503915	-2.62
Subst_ TS-B3	-1592.15458187	
Catalyst Only		
Cat_ TS-B1	-6727.87908673675	3.42
Cat_ TS-B3	-6727.87363891	
Total Distortion		0.80
Total Interaction	(predominant factor)	2.04

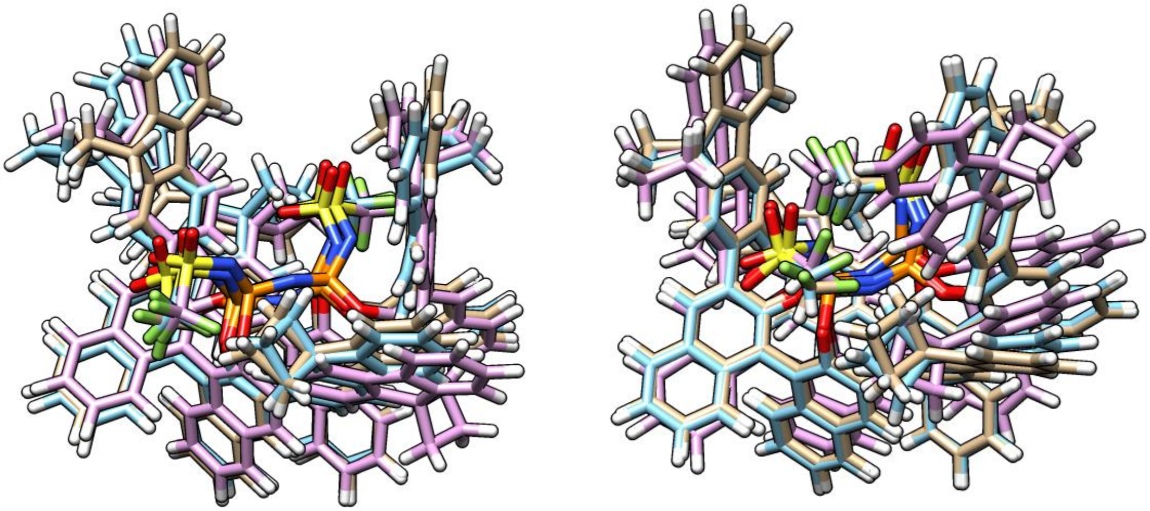

Figure S11. Comparative overlay picture of the counteranions to show the origin of the distortion. Right, *syn*-selective aldol reaction with catalyst **7a**- enantiomers. Left, *anti*-selective aldol reaction with catalyst **7d** -enantiomers. Catalyst counteranion without nucleophile (khaki); TS-major (blue); TS-minor (purple)

Extention to the aldol addition of acetaldehyde-derived enolsilanes

Finally, we have extended our computational model to predict the outcome of an analogous acetaldehyde enolsilane addition (Figure 2C, main text). In order to *qualitatively* understand the outcome of analogous acetaldehyde derived enolsilane mediated aldol reaction, we performed fragmentation based analysis. Gratifyingly, in both cases, our computed model can be successfully extended to qualitatively predict the experimental outcome of the acetaldehyde enolsilane addition (Figure S12 for catalyst **7a** and enolsilane **19b**; (Figure S13 for catalyst **7d** and enolsilane **19a**).

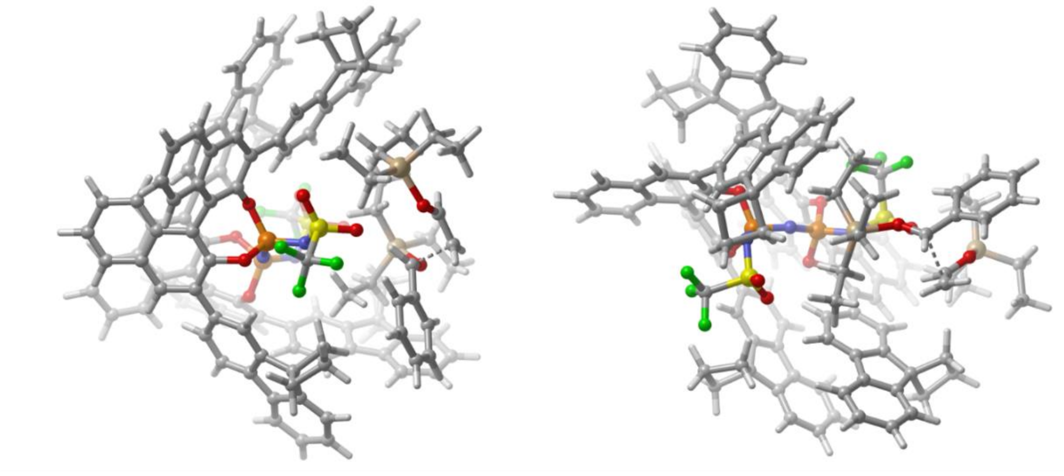

0.0 3.5

Figure S12. Transition state models for acetaldehyde aldol addition to benzaldehyde with catalyst **7a** and enolsilane **19b**.

Table S15.1. Extension of the computed stereochemical model to the acetaldehyde aldol (with catalyst **7a**) reaction:

TS No	*B3LYP-D3(BJ)/def2-TZVP* single point Δ E($CHCl_3$ solv)	Relative TS Energy (Δ E) (kcal/mol)
Major Enantiomer	-8479.40245903518	0.0
Minor Enantiomer	-8479.396857245965	+3.51

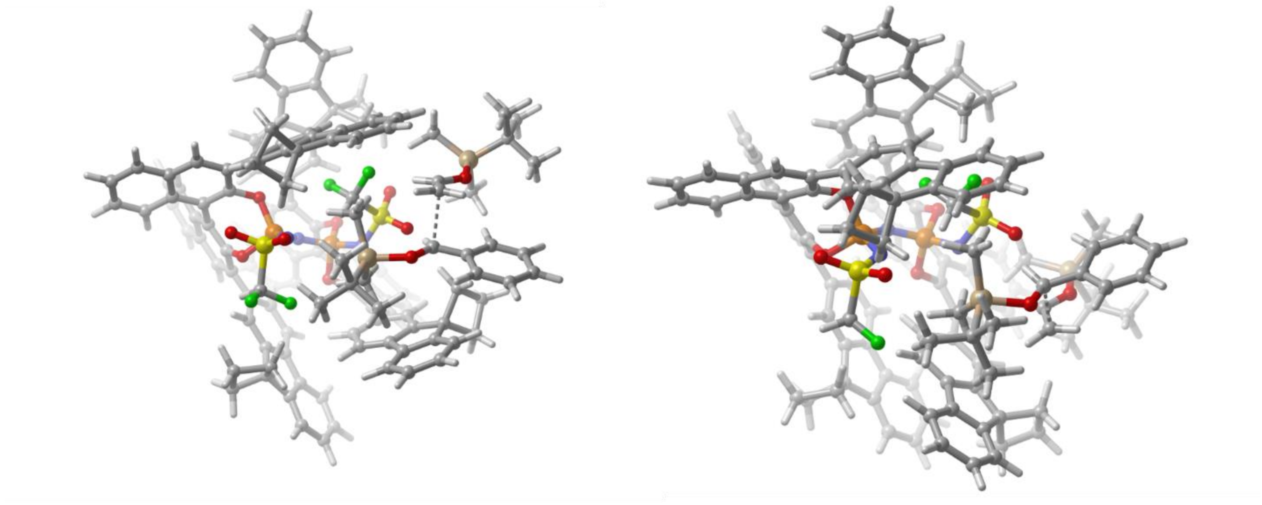

0.0 4.5

Figure S13. Key structures for the model acetaldehyde aldol addition to benzaldehyde (with catalyst **7d** and enolsilane **19a**).

Table S15.2. Extension of the computed stereochemical model to the acetaldehyde aldol reaction (with Catalyst **7d**):

TS No	*B3LYP-D3(BJ)/def2-TZVP* single point Δ E($CHCl_3$ solv)	Relative TS Energy (Δ E) (kcal/mol)
Major Enantiomer	-8280.90798962	0.0
Minor Enantiomer	-8280.90073913	+4.55

Optimized Cartesian Coordinates of the Key Transition States

Syn Selective Aldol Reaction:

TS-A1:

B3LYP-D3(BJ) Energy: -8518.7199816 hartree; Imaginary frequency = -129.50029

16	-12.767944000	-11.232250000	-5.598031000
16	-7.831344000	-8.312025000	-8.008442000
8	-12.289508000	-8.634748000	-3.596745000
8	-10.015100000	-9.837048000	-3.803366000
8	-12.190649000	-12.153218000	-4.583139000
8	-13.157521000	-11.789021000	-6.926258000
8	-7.894802000	-6.826885000	-5.144839000
8	-9.916609000	-5.384357000	-5.948948000
8	-7.711938000	-9.448478000	-7.044524000
8	-8.008785000	-8.634196000	-9.453760000
6	-13.436177000	-10.985495000	-9.862086000
1	-13.044338000	-11.276465000	-8.877535000
8	-12.587552000	-10.556354000	-10.776711000
14	-10.903500000	-10.039591000	-10.442720000
15	-11.145008000	-9.036771000	-4.740330000
15	-9.314786000	-6.924947000	-6.026858000
7	-10.415631000	-7.795998000	-5.333949000
7	-11.944467000	-9.877576000	-5.868617000
7	-8.875893000	-7.173173000	-7.562790000
6	-13.402910000	-13.615666000	-9.728921000
1	-12.338323000	-13.809115000	-9.945586000
6	-14.148138000	-12.814404000	-10.598451000
1	-15.208554000	-12.720795000	-10.338698000
8	-13.900672000	-14.078336000	-8.612313000
14	-13.004233000	-14.972692000	-7.348695000
6	-14.459058000	-10.733395000	-4.853458000

6	-6.153479000	-7.436863000	-8.030611000
6	-11.195274000	-8.245046000	-9.996442000
6	-10.114074000	-10.343156000	-12.129734000
6	-10.224981000	-11.162908000	-9.078126000
6	-13.793139000	-12.710098000	-12.059084000
6	-13.076019000	-16.754932000	-7.988843000
6	-14.083277000	-14.769618000	-5.818586000
6	-11.227204000	-14.364978000	-7.261462000
6	-11.814763000	-7.951191000	-2.468353000
6	-10.366626000	-10.687861000	-2.754833000
6	-7.891916000	-6.177416000	-3.912563000
6	-8.963948000	-4.380130000	-6.173497000
6	-14.769507000	-10.330817000	-9.830686000
9	-15.432648000	-11.482241000	-5.502398000
9	-14.738607000	-9.394353000	-5.050620000
9	-14.470989000	-11.017683000	-3.522486000
9	-5.756825000	-6.995872000	-6.784400000
9	-5.224577000	-8.347294000	-8.490505000
9	-6.234457000	-6.367314000	-8.893100000
1	-11.625154000	-7.735263000	-10.875006000
1	-10.231165000	-7.756484000	-9.790567000
1	-9.898797000	-11.422062000	-12.244023000
1	-9.135006000	-9.829482000	-12.137169000
1	-10.140402000	-10.594224000	-8.135756000
1	-10.954589000	-11.967282000	-8.876198000
6	-12.129508000	-8.061542000	-8.777086000
6	-8.849467000	-11.794890000	-9.425515000
6	-10.996242000	-9.841325000	-13.301890000
1	-12.703274000	-12.741895000	-12.220199000
1	-14.253381000	-13.540373000	-12.623845000

1	-14.171183000	-11.764020000	-12.481109000
1	-12.485359000	-16.823299000	-8.921562000
1	-12.554346000	-17.398243000	-7.253061000
6	-14.515223000	-17.275792000	-8.236048000
1	-14.480073000	-13.742607000	-5.752612000
1	-14.954858000	-15.440820000	-5.933431000
6	-13.294807000	-15.125854000	-4.523520000
1	-11.205216000	-13.264235000	-7.251769000
1	-10.828656000	-14.648394000	-6.270674000
6	-10.278686000	-14.919006000	-8.357909000
6	-11.958753000	-6.527152000	-2.414384000
6	-11.531644000	-5.890030000	-1.250028000
1	-11.665684000	-4.810110000	-1.148627000
6	-10.906197000	-6.598796000	-0.187545000
6	-10.466028000	-5.923433000	0.991680000
1	-10.654874000	-4.850313000	1.074564000
6	-9.779178000	-6.603430000	1.985301000
1	-9.442787000	-6.075870000	2.881722000
6	-9.496747000	-7.989684000	1.834902000
1	-8.932073000	-8.514382000	2.609395000
6	-9.947126000	-8.685530000	0.721710000
1	-9.756857000	-9.755201000	0.622825000
6	-10.684173000	-8.019657000	-0.305513000
6	-11.194774000	-8.696797000	-1.471582000
6	-9.958157000	-12.065891000	-2.836374000
6	-10.228883000	-12.869982000	-1.727637000
1	-9.849124000	-13.893703000	-1.702012000
6	-11.004495000	-12.425672000	-0.624251000
6	-11.366478000	-13.303594000	0.443560000
1	-11.012675000	-14.338449000	0.411998000

6	-12.168227000	-12.859881000	1.483374000
1	-12.448279000	-13.540494000	2.291145000
6	-12.646173000	-11.519307000	1.494262000
1	-13.300594000	-11.185894000	2.303281000
6	-12.292739000	-10.636154000	0.484299000
1	-12.655294000	-9.606671000	0.490041000
6	-11.449301000	-11.057725000	-0.586544000
6	-11.020176000	-10.167877000	-1.633711000
6	-12.517328000	-5.745303000	-3.549314000
6	-11.983822000	-4.457418000	-3.815130000
1	-11.130896000	-4.105928000	-3.228492000
6	-12.485442000	-3.655535000	-4.850798000
1	-12.047023000	-2.671761000	-5.041763000
6	-13.516953000	-4.158534000	-5.658964000
6	-14.193801000	-3.604787000	-6.849667000
6	-14.006940000	-2.398600000	-7.542392000
1	-13.292674000	-1.652803000	-7.183679000
6	-14.729688000	-2.180325000	-8.729289000
1	-14.581620000	-1.254075000	-9.289712000
6	-15.623050000	-3.152973000	-9.218273000
1	-16.161745000	-2.970872000	-10.151791000
6	-15.820344000	-4.362820000	-8.519193000
1	-16.508150000	-5.123009000	-8.902601000
6	-15.112205000	-4.582078000	-7.334907000
6	-15.119009000	-5.796454000	-6.425652000
6	-14.030995000	-5.464886000	-5.422243000
6	-13.558496000	-6.249087000	-4.374617000
1	-13.957480000	-7.250980000	-4.217181000
6	-15.104676000	-7.208537000	-7.150398000
1	-14.983068000	-7.142758000	-8.241336000

1	-14.355442000	-7.890057000	-6.726061000
6	-16.567335000	-7.494323000	-6.643392000
1	-17.314387000	-7.485742000	-7.450379000
1	-16.651132000	-8.416543000	-6.051119000
6	-16.539041000	-6.165978000	-5.800616000
1	-16.463177000	-6.330934000	-4.714530000
1	-17.324636000	-5.429425000	-6.026485000
6	-9.245871000	-12.648255000	-4.007496000
6	-8.494728000	-11.870712000	-4.927212000
1	-8.451286000	-10.790915000	-4.831118000
6	-7.760318000	-12.468487000	-5.961347000
1	-7.217394000	-11.824588000	-6.653342000
6	-7.736220000	-13.862435000	-6.068840000
6	-7.040120000	-14.757332000	-7.007047000
6	-6.180405000	-14.465624000	-8.076018000
1	-5.920017000	-13.429386000	-8.309088000
6	-5.663953000	-15.531268000	-8.835506000
1	-4.993822000	-15.325001000	-9.674093000
6	-6.002404000	-16.862311000	-8.526640000
1	-5.593732000	-17.677967000	-9.128798000
6	-6.863074000	-17.155982000	-7.447151000
1	-7.117511000	-18.194025000	-7.214805000
6	-7.381507000	-16.105045000	-6.688002000
6	-8.309502000	-16.133722000	-5.477368000
6	-8.500487000	-14.658380000	-5.171828000
6	-9.265082000	-14.063562000	-4.176364000
1	-9.897793000	-14.683937000	-3.535667000
6	-7.785452000	-17.069773000	-4.302804000
1	-8.042337000	-16.626773000	-3.326794000
1	-6.714861000	-17.314082000	-4.350773000

6	-8.812770000	-18.177406000	-4.744790000
1	-9.392490000	-18.660795000	-3.943890000
1	-8.338104000	-18.943144000	-5.379555000
6	-9.566323000	-17.095304000	-5.604894000
1	-9.847102000	-17.366772000	-6.633303000
1	-10.430657000	-16.672106000	-5.064791000
6	-7.592440000	-6.949186000	-2.746915000
6	-7.637471000	-6.299609000	-1.513310000
1	-7.390305000	-6.855675000	-0.605313000
6	-7.995982000	-4.926514000	-1.399299000
6	-8.074544000	-4.271395000	-0.132426000
1	-7.854217000	-4.854115000	0.764699000
6	-8.450195000	-2.939286000	-0.042784000
1	-8.512734000	-2.451240000	0.933361000
6	-8.756418000	-2.198687000	-1.219939000
1	-9.067718000	-1.154549000	-1.135950000
6	-8.648152000	-2.790615000	-2.470146000
1	-8.856353000	-2.226683000	-3.382548000
6	-8.253525000	-4.157547000	-2.592449000
6	-8.124966000	-4.802380000	-3.866360000
6	-8.882704000	-3.801823000	-7.473052000
6	-7.918661000	-2.829000000	-7.705367000
1	-7.856317000	-2.366939000	-8.694643000
6	-7.006357000	-2.426494000	-6.687903000
6	-5.999071000	-1.445231000	-6.937691000
1	-5.962898000	-0.976115000	-7.925356000
6	-5.080622000	-1.104598000	-5.957597000
1	-4.311967000	-0.354795000	-6.160359000
6	-5.124552000	-1.743950000	-4.687799000
1	-4.381219000	-1.488529000	-3.928626000

6	-6.095236000	-2.694206000	-4.407123000
1	-6.123638000	-3.188180000	-3.435275000
6	-7.076309000	-3.045158000	-5.383252000
6	-8.098167000	-4.034308000	-5.143192000
6	-7.164235000	-8.369748000	-2.852177000
6	-7.585150000	-9.285185000	-1.859841000
1	-8.288126000	-8.942980000	-1.098201000
6	-7.164603000	-10.622062000	-1.878712000
1	-7.550613000	-11.329723000	-1.140859000
6	-6.278053000	-11.038557000	-2.881757000
6	-5.720512000	-12.367393000	-3.183089000
6	-5.956124000	-13.617315000	-2.594309000
1	-6.628176000	-13.711254000	-1.736942000
6	-5.337537000	-14.751735000	-3.150244000
1	-5.517786000	-15.736331000	-2.711232000
6	-4.506500000	-14.637680000	-4.280758000
1	-4.061279000	-15.534606000	-4.718708000
6	-4.265482000	-13.380036000	-4.870226000
1	-3.635691000	-13.300418000	-5.761032000
6	-4.871800000	-12.249159000	-4.320880000
6	-4.799822000	-10.804283000	-4.778622000
6	-5.815156000	-10.116550000	-3.867772000
6	-6.278076000	-8.801662000	-3.879559000
1	-5.992378000	-8.117179000	-4.677670000
6	-4.856513000	-10.466834000	-6.322488000
1	-4.580536000	-11.355155000	-6.916765000
1	-5.806492000	-10.043586000	-6.665714000
6	-3.653234000	-9.468225000	-6.150385000
1	-2.842168000	-9.544375000	-6.890663000
1	-4.010305000	-8.430127000	-6.093563000

6	-3.367201000	-10.094609000	-4.735681000
1	-3.218306000	-9.406292000	-3.890824000
1	-2.569820000	-10.854974000	-4.769658000
6	-9.797916000	-4.254981000	-8.547147000
6	-11.193347000	-4.230279000	-8.340619000
1	-11.584187000	-3.944266000	-7.365450000
6	-12.075842000	-4.622268000	-9.356085000
1	-13.153600000	-4.601026000	-9.180354000
6	-11.539649000	-5.048381000	-10.578635000
6	-12.209961000	-5.584010000	-11.774524000
6	-13.567420000	-5.834368000	-12.026092000
1	-14.323976000	-5.591180000	-11.274316000
6	-13.926471000	-6.415557000	-13.257211000
1	-14.979091000	-6.621212000	-13.473161000
6	-12.946406000	-6.728373000	-14.219291000
1	-13.244893000	-7.174577000	-15.171464000
6	-11.580137000	-6.483047000	-13.960682000
1	-10.821563000	-6.742454000	-14.705785000
6	-11.212385000	-5.927653000	-12.733266000
6	-9.827007000	-5.639314000	-12.179282000
6	-10.130675000	-5.097564000	-10.788530000
6	-9.257602000	-4.714117000	-9.771495000
1	-8.175432000	-4.849845000	-9.850886000
6	-8.840448000	-4.720834000	-13.021065000
1	-8.770389000	-3.678222000	-12.677540000
1	-9.100687000	-4.763845000	-14.091917000
6	-7.656912000	-5.696320000	-12.666499000
1	-6.983734000	-5.974245000	-13.491721000
1	-7.068538000	-5.335098000	-11.807059000
6	-8.712447000	-6.775143000	-12.217472000

1	-8.522064000	-7.296908000	-11.268624000
1	-8.933264000	-7.493149000	-13.025175000
6	-16.392937000	-8.828459000	-10.835739000
6	-15.134696000	-9.446981000	-10.866489000
6	-15.642517000	-10.570371000	-8.744583000
6	-16.905853000	-9.967322000	-8.740203000
6	-17.288101000	-9.103957000	-9.786736000
1	-16.673039000	-8.129244000	-11.628270000
1	-14.408976000	-9.222361000	-11.651476000
1	-15.311715000	-11.189143000	-7.905872000
1	-17.587189000	-10.145763000	-7.905023000
1	-18.274413000	-8.632484000	-9.770233000
1	-11.760250000	-8.589535000	-7.881420000
1	-12.196006000	-6.987178000	-8.533483000
1	-13.148382000	-8.426035000	-8.994898000
1	-8.516025000	-12.438620000	-8.594820000
1	-8.904932000	-12.412666000	-10.339797000
1	-8.103156000	-10.998264000	-9.573952000
1	-10.504509000	-10.023109000	-14.275311000
1	-11.972023000	-10.357337000	-13.298568000
1	-11.193999000	-8.760005000	-13.213461000
1	-15.038163000	-16.635710000	-8.969018000
1	-14.502734000	-18.309360000	-8.624931000
1	-15.102784000	-17.270095000	-7.300859000
1	-12.762544000	-16.092532000	-4.617718000
1	-12.567870000	-14.324447000	-4.313177000
1	-13.979965000	-15.203116000	-3.660769000
1	-10.514867000	-14.502192000	-9.354332000
1	-9.237549000	-14.650864000	-8.122002000
1	-10.327339000	-16.019841000	-8.426665000

TS-A2:

B3LYP-D3(BJ) Energy: -8518.71161370 *hartree; Imaginary frequency = -204.32262*

16	-13.157476000	-11.197961000	-6.088932000
16	-7.521121000	-7.971432000	-8.168667000
8	-12.524947000	-8.354455000	-4.387844000
8	-10.223062000	-9.536010000	-4.360683000
8	-12.596398000	-12.311677000	-5.286709000
8	-13.806852000	-11.508107000	-7.397018000
8	-8.301434000	-6.739936000	-5.352950000
8	-10.220443000	-5.338843000	-6.459751000
8	-7.449448000	-9.045289000	-7.133015000
8	-7.460826000	-8.368058000	-9.603918000
6	-12.661208000	-12.196161000	-10.617635000
1	-12.752778000	-11.562309000	-9.721876000
8	-11.452485000	-12.293782000	-11.170166000
14	-10.057281000	-11.413279000	-10.478981000
15	-11.364535000	-8.954332000	-5.429907000
15	-9.488975000	-6.825228000	-6.532948000
7	-10.635329000	-7.876972000	-6.313602000
7	-12.162778000	-9.971002000	-6.369077000
7	-8.697415000	-6.893681000	-7.942443000
6	-14.194611000	-10.158027000	-11.145620000
1	-13.571295000	-9.245638000	-11.133237000
6	-13.835266000	-11.245485000	-11.961323000
1	-14.605273000	-12.021143000	-12.031211000
8	-15.204858000	-10.234287000	-10.324490000
14	-15.722838000	-8.962513000	-9.167904000
6	-14.607367000	-10.540658000	-5.028636000
6	-5.969236000	-6.923703000	-7.957866000
6	-10.146508000	-9.666114000	-11.182040000

6	-8.545323000	-12.342425000	-11.102387000
6	-10.267016000	-11.517050000	-8.620090000
6	-13.008666000	-11.003078000	-13.201964000
6	-16.550329000	-7.704934000	-10.316539000
6	-14.193145000	-8.303264000	-8.306183000
6	-16.908511000	-9.963027000	-8.097631000
6	-12.181882000	-7.767568000	-3.175313000
6	-10.664685000	-10.472278000	-3.413560000
6	-8.439323000	-6.054948000	-4.154411000
6	-9.321326000	-4.264787000	-6.535602000
6	-13.438945000	-13.467647000	-10.543124000
9	-15.717493000	-11.307195000	-5.318199000
9	-14.927140000	-9.215283000	-5.339266000
9	-14.294468000	-10.633943000	-3.704076000
9	-5.834795000	-6.471706000	-6.658459000
9	-4.874350000	-7.700842000	-8.270341000
9	-6.047090000	-5.849989000	-8.816006000
1	-10.690279000	-9.698344000	-12.143351000
1	-9.120076000	-9.349371000	-11.432040000
1	-8.411649000	-12.160516000	-12.184811000
1	-8.665279000	-13.431644000	-10.963921000
1	-9.329855000	-11.156040000	-8.157406000
1	-11.041306000	-10.809159000	-8.280537000
6	-10.790966000	-8.627180000	-10.230313000
6	-10.585503000	-12.938651000	-8.083221000
6	-7.300535000	-11.841477000	-10.304763000
1	-12.521026000	-10.015452000	-13.183260000
1	-12.228447000	-11.774208000	-13.317383000
1	-13.659837000	-11.035889000	-14.092727000
1	-15.781088000	-7.361747000	-11.035207000

1	-17.335898000	-8.209720000	-10.907888000
6	-17.149904000	-6.477865000	-9.576539000
1	-14.434447000	-8.191352000	-7.237265000
1	-13.447117000	-9.117625000	-8.317101000
6	-13.552467000	-7.007624000	-8.852679000
1	-17.645859000	-10.443863000	-8.766771000
1	-16.332017000	-10.770203000	-7.612529000
6	-17.632546000	-9.153796000	-6.984651000
6	-12.398515000	-6.359288000	-3.029748000
6	-12.118511000	-5.786313000	-1.792803000
1	-12.296852000	-4.718388000	-1.641720000
6	-11.608132000	-6.555626000	-0.709754000
6	-11.335803000	-5.956581000	0.557682000
1	-11.557981000	-4.894219000	0.685049000
6	-10.772873000	-6.695005000	1.587162000
1	-10.563453000	-6.225142000	2.551624000
6	-10.460426000	-8.068876000	1.389226000
1	-9.998011000	-8.640667000	2.197405000
6	-10.755520000	-8.694099000	0.186043000
1	-10.547912000	-9.756316000	0.047122000
6	-11.353016000	-7.964899000	-0.887951000
6	-11.689993000	-8.571127000	-2.151479000
6	-10.227194000	-11.834236000	-3.536383000
6	-10.662296000	-12.720737000	-2.547803000
1	-10.284619000	-13.745604000	-2.546507000
6	-11.603961000	-12.356637000	-1.549135000
6	-12.123080000	-13.311753000	-0.620958000
1	-11.767431000	-14.344537000	-0.680759000
6	-13.074473000	-12.942972000	0.316683000
1	-13.472842000	-13.682830000	1.015318000

6	-13.552101000	-11.602656000	0.363047000
1	-14.322171000	-11.328698000	1.088223000
6	-13.052623000	-10.646370000	-0.508791000
1	-13.414067000	-9.616821000	-0.478440000
6	-12.056262000	-10.991613000	-1.469198000
6	-11.486172000	-10.030532000	-2.374240000
6	-12.940127000	-5.545934000	-4.151298000
6	-13.997095000	-6.041849000	-4.957933000
1	-14.376223000	-7.047180000	-4.784314000
6	-14.542363000	-5.264032000	-5.987647000
1	-15.359339000	-5.661394000	-6.596542000
6	-14.032784000	-3.977871000	-6.215645000
6	-14.388653000	-2.959156000	-7.218867000
6	-15.286690000	-3.005173000	-8.296734000
1	-15.896349000	-3.894559000	-8.477430000
6	-15.387020000	-1.886085000	-9.143308000
1	-16.077540000	-1.906503000	-9.990405000
6	-14.601676000	-0.740778000	-8.912478000
1	-14.686276000	0.117039000	-9.584266000
6	-13.700390000	-0.692591000	-7.829288000
1	-13.085651000	0.196333000	-7.663794000
6	-13.594352000	-1.799257000	-6.983244000
6	-12.679885000	-2.024537000	-5.787290000
6	-12.984510000	-3.463485000	-5.400350000
6	-12.433739000	-4.240758000	-4.381355000
1	-11.600823000	-3.870901000	-3.776102000
6	-12.823859000	-0.932544000	-4.635732000
1	-13.758025000	-0.352767000	-4.655826000
1	-12.675011000	-1.408929000	-3.652501000
6	-11.510448000	-0.234806000	-5.150807000

1	-11.726756000	0.643531000	-5.780138000
1	-10.766313000	0.030431000	-4.383292000
6	-11.194825000	-1.514714000	-6.008371000
1	-10.480516000	-2.187915000	-5.517099000
1	-10.902838000	-1.365369000	-7.057210000
6	-9.308420000	-12.311611000	-4.604352000
6	-8.404672000	-11.456130000	-5.286358000
1	-8.407929000	-10.386408000	-5.103034000
6	-7.466824000	-11.966763000	-6.196476000
1	-6.808066000	-11.273234000	-6.716433000
6	-7.405460000	-13.344512000	-6.417962000
6	-6.512401000	-14.157098000	-7.259098000
6	-5.395638000	-13.790900000	-8.025109000
1	-5.072523000	-12.747338000	-8.066470000
6	-4.697862000	-14.792587000	-8.722873000
1	-3.825875000	-14.526818000	-9.326296000
6	-5.108497000	-16.137590000	-8.647413000
1	-4.554112000	-16.903897000	-9.195621000
6	-6.224041000	-16.507892000	-7.867135000
1	-6.529265000	-17.556453000	-7.807650000
6	-6.924951000	-15.518397000	-7.173133000
6	-8.130281000	-15.638580000	-6.247046000
6	-8.343137000	-14.209045000	-5.788765000
6	-9.294806000	-13.703161000	-4.914404000
1	-10.057833000	-14.362764000	-4.491876000
6	-8.007479000	-16.783732000	-5.157966000
1	-8.494070000	-16.467878000	-4.220170000
1	-6.982384000	-17.134158000	-4.965826000
6	-8.945639000	-17.698073000	-6.034324000
1	-9.753970000	-18.225248000	-5.505108000

1	-8.366742000	-18.412439000	-6.642088000
6	-9.361927000	-16.426514000	-6.867506000
1	-9.357775000	-16.509143000	-7.964239000
1	-10.310199000	-15.988323000	-6.515127000
6	-8.174906000	-6.791121000	-2.955133000
6	-8.358655000	-6.144067000	-1.736524000
1	-8.150705000	-6.682392000	-0.808144000
6	-8.798544000	-4.791807000	-1.666601000
6	-9.021690000	-4.140790000	-0.415239000
1	-8.858340000	-4.714485000	0.499862000
6	-9.462668000	-2.826763000	-0.363532000
1	-9.639681000	-2.343448000	0.600753000
6	-9.677923000	-2.098432000	-1.567359000
1	-10.031513000	-1.065092000	-1.522048000
6	-9.420701000	-2.684909000	-2.798292000
1	-9.544270000	-2.114234000	-3.719945000
6	-8.974993000	-4.039434000	-2.884588000
6	-8.716558000	-4.685882000	-4.143143000
6	-9.176057000	-3.600845000	-7.786241000
6	-8.283858000	-2.544498000	-7.883153000
1	-8.177217000	-2.020015000	-8.836730000
6	-7.493468000	-2.138585000	-6.768812000
6	-6.550442000	-1.071806000	-6.877630000
1	-6.471410000	-0.542510000	-7.831751000
6	-5.743406000	-0.725533000	-5.806057000
1	-5.022289000	0.090001000	-5.900474000
6	-5.836524000	-1.447325000	-4.584137000
1	-5.174936000	-1.190189000	-3.753344000
6	-6.749803000	-2.481011000	-4.439855000
1	-6.811805000	-3.038226000	-3.504769000

6	-7.622897000	-2.839799000	-5.511791000
6	-8.589732000	-3.907753000	-5.407690000
6	-7.610968000	-8.165611000	-3.019178000
6	-8.076226000	-9.148544000	-2.117803000
1	-8.897482000	-8.893106000	-1.446023000
6	-7.546964000	-10.447270000	-2.127943000
1	-7.974025000	-11.219433000	-1.483229000
6	-6.498996000	-10.743898000	-3.010296000
6	-5.828301000	-12.018881000	-3.314936000
6	-6.115148000	-13.325377000	-2.897115000
1	-6.917034000	-13.516726000	-2.179114000
6	-5.391081000	-14.389674000	-3.464889000
1	-5.624609000	-15.418243000	-3.177163000
6	-4.392190000	-14.147999000	-4.426158000
1	-3.865894000	-14.988933000	-4.884138000
6	-4.098649000	-12.833141000	-4.841703000
1	-3.343126000	-12.656339000	-5.612595000
6	-4.825079000	-11.773436000	-4.296270000
6	-4.777544000	-10.297139000	-4.646414000
6	-5.970124000	-9.737271000	-3.873014000
6	-6.549003000	-8.470677000	-3.915839000
1	-6.225712000	-7.731878000	-4.649067000
6	-4.620420000	-9.855817000	-6.155599000
1	-4.166085000	-10.670492000	-6.745732000
1	-5.540742000	-9.493687000	-6.623605000
6	-3.563484000	-8.772597000	-5.729423000
1	-2.647466000	-8.711866000	-6.336673000
1	-4.031130000	-7.779011000	-5.658381000
6	-3.432553000	-9.490117000	-4.334499000
1	-3.464000000	-8.867094000	-3.428680000

1	-2.577063000	-10.184954000	-4.306343000
6	-9.978171000	-4.058294000	-8.951998000
6	-11.377675000	-3.881688000	-8.954686000
1	-11.868612000	-3.392114000	-8.113235000
6	-12.153258000	-4.369017000	-10.016347000
1	-13.238004000	-4.240235000	-9.995026000
6	-11.511415000	-5.045037000	-11.063456000
6	-12.069165000	-5.828602000	-12.180452000
6	-13.396872000	-6.152309000	-12.502597000
1	-14.227915000	-5.720139000	-11.937264000
6	-13.638372000	-7.048894000	-13.562149000
1	-14.666672000	-7.313576000	-13.824337000
6	-12.568448000	-7.602096000	-14.293600000
1	-12.775419000	-8.290548000	-15.117341000
6	-11.232978000	-7.273251000	-13.973583000
1	-10.404149000	-7.710938000	-14.537649000
6	-10.986075000	-6.396229000	-12.915462000
6	-9.665518000	-5.893718000	-12.363496000
6	-10.091325000	-5.177048000	-11.089463000
6	-9.322889000	-4.703555000	-10.025718000
1	-8.251586000	-4.903420000	-9.944769000
6	-8.828676000	-4.917368000	-13.316555000
1	-8.964740000	-3.843550000	-13.122480000
1	-9.036687000	-5.158939000	-14.372108000
6	-7.511034000	-5.608901000	-12.808282000
1	-6.751060000	-5.844196000	-13.568458000
1	-7.054173000	-5.047067000	-11.977328000
6	-8.381508000	-6.806389000	-12.270743000
1	-8.140669000	-7.211011000	-11.278208000
1	-8.443817000	-7.618180000	-13.015758000

6	-13.900017000	-15.693037000	-11.400286000
6	-13.155007000	-14.508729000	-11.450036000
6	-14.444663000	-13.619402000	-9.565840000
6	-15.181884000	-14.812651000	-9.522441000
6	-14.922100000	-15.843076000	-10.443031000
1	-13.681373000	-16.505630000	-12.097820000
1	-12.340050000	-14.376126000	-12.164707000
1	-14.582220000	-12.839175000	-8.812711000
1	-15.950113000	-14.944648000	-8.756416000
1	-15.500529000	-16.769764000	-10.404080000
1	-10.086482000	-8.327827000	-9.440309000
1	-11.064381000	-7.714075000	-10.780879000
1	-11.696458000	-9.019844000	-9.733747000
1	-10.743720000	-12.900511000	-6.992570000
1	-11.502427000	-13.352455000	-8.540307000
1	-9.748793000	-13.631446000	-8.281244000
1	-6.370296000	-12.250992000	-10.736366000
1	-7.228840000	-10.737345000	-10.286053000
1	-7.363043000	-12.192427000	-9.260305000
1	-16.377237000	-5.964132000	-8.977945000
1	-17.562064000	-5.747235000	-10.295565000
1	-17.962532000	-6.782118000	-8.895243000
1	-14.233675000	-6.144635000	-8.765020000
1	-12.642986000	-6.769974000	-8.275148000
1	-13.259241000	-7.105664000	-9.911600000
1	-16.901256000	-8.800426000	-6.242298000
1	-18.185176000	-8.288707000	-7.389207000
1	-18.355552000	-9.800963000	-6.456675000

TS-A3:

B3LYP-D3(BJ) Energy: - 8518.712005790   hartree; Imaginary frequency =   -128.15021

16	-13.402439000	-10.084338000	-4.337535000
16	-8.743028000	-7.044752000	-9.114312000
8	-11.336458000	-7.761780000	-3.018457000
8	-9.775839000	-9.827575000	-3.407326000
8	-13.435599000	-10.330713000	-2.873033000
8	-13.871113000	-11.160427000	-5.250050000
8	-7.737451000	-6.550694000	-6.026952000
8	-10.050691000	-5.551646000	-5.567306000
8	-8.196310000	-8.402975000	-9.446261000
8	-9.517293000	-6.356715000	-10.185794000
6	-9.681104000	-10.857077000	-10.755981000
1	-9.434766000	-10.034973000	-10.067635000
8	-10.590210000	-11.741172000	-10.393350000
14	-11.106667000	-11.808675000	-8.662170000
15	-10.780109000	-8.777055000	-4.235316000
15	-9.353223000	-6.937312000	-6.196876000
7	-9.795242000	-8.127215000	-5.283468000
7	-12.056158000	-9.432084000	-4.942348000
7	-9.584265000	-7.069683000	-7.768302000
6	-11.563189000	-9.258182000	-11.420151000
1	-11.421283000	-8.432661000	-10.707862000
6	-10.552699000	-9.610453000	-12.310093000
1	-10.764832000	-10.441789000	-12.995788000
8	-12.679327000	-9.928141000	-11.229042000
14	-13.649216000	-10.877607000	-12.356632000
6	-14.653190000	-8.682755000	-4.662897000
6	-7.161232000	-6.006784000	-8.958425000
6	-11.460731000	-10.080551000	-8.032025000

6	-9.573349000	-12.488687000	-7.814291000
6	-12.589587000	-12.959297000	-8.739040000
6	-9.521946000	-8.609955000	-12.755718000
6	-15.196315000	-11.178653000	-11.327579000
6	-12.741739000	-12.472977000	-12.825545000
6	-13.920903000	-9.732280000	-13.837517000
6	-10.437321000	-7.179484000	-2.121476000
6	-10.212042000	-10.277643000	-2.156102000
6	-7.300967000	-5.862649000	-4.898075000
6	-9.620394000	-4.377416000	-6.196071000
6	-8.558486000	-11.327840000	-11.590221000
9	-14.098281000	-7.437173000	-4.407671000
9	-15.746558000	-8.865751000	-3.858572000
9	-15.050605000	-8.697551000	-5.989968000
9	-7.445560000	-4.812047000	-8.357462000
9	-6.173169000	-6.675318000	-8.259044000
9	-6.718369000	-5.796122000	-10.242270000
1	-10.791027000	-9.346264000	-8.515570000
1	-11.117689000	-10.108246000	-6.981198000
1	-9.894987000	-12.898457000	-6.841448000
1	-8.927317000	-11.627004000	-7.565598000
1	-12.955392000	-13.070031000	-7.702232000
1	-13.393833000	-12.438933000	-9.290626000
6	-12.923615000	-9.582353000	-8.041810000
6	-12.313486000	-14.358270000	-9.345348000
6	-8.753135000	-13.547967000	-8.591810000
1	-9.304405000	-7.867254000	-11.973152000
1	-8.585367000	-9.117422000	-13.038215000
1	-9.895006000	-8.071746000	-13.646775000
1	-15.027821000	-12.028012000	-10.640813000

1	-15.343684000	-10.280443000	-10.701238000
6	-16.473244000	-11.423941000	-12.175684000
1	-12.050910000	-12.284593000	-13.669281000
1	-12.120439000	-12.767031000	-11.962775000
6	-13.713357000	-13.632208000	-13.181562000
1	-14.637046000	-8.948951000	-13.532984000
1	-12.969245000	-9.213799000	-14.061486000
6	-14.419786000	-10.478902000	-15.103445000
6	-10.323517000	-5.747739000	-2.084701000
6	-9.395295000	-5.202741000	-1.194642000
1	-9.314818000	-4.117217000	-1.100831000
6	-8.557295000	-6.011603000	-0.379573000
6	-7.585941000	-5.444067000	0.500742000
1	-7.490968000	-4.356540000	0.532877000
6	-6.764250000	-6.255943000	1.267809000
1	-6.016947000	-5.811518000	1.930413000
6	-6.890861000	-7.673133000	1.201410000
1	-6.227404000	-8.303648000	1.798312000
6	-7.861707000	-8.254667000	0.398815000
1	-7.989801000	-9.339010000	0.361727000
6	-8.722657000	-7.441656000	-0.399021000
6	-9.742708000	-8.002845000	-1.236429000
6	-10.696635000	-11.612413000	-2.046225000
6	-11.147686000	-12.060832000	-0.811810000
1	-11.500802000	-13.091590000	-0.720118000
6	-11.171991000	-11.211002000	0.328139000
6	-11.676245000	-11.664062000	1.585016000
1	-12.025579000	-12.697728000	1.663538000
6	-11.738396000	-10.809516000	2.673677000
1	-12.131195000	-11.163130000	3.630116000

6	-11.309279000	-9.459761000	2.542648000
1	-11.388164000	-8.782866000	3.396922000
6	-10.802990000	-8.990729000	1.339612000
1	-10.485115000	-7.952432000	1.241335000
6	-10.695551000	-9.853722000	0.206694000
6	-10.180739000	-9.413785000	-1.067387000
6	-11.256275000	-4.856368000	-2.826037000
6	-12.589700000	-5.258682000	-3.095055000
1	-12.888666000	-6.282550000	-2.893684000
6	-13.524447000	-4.371057000	-3.645935000
1	-14.538226000	-4.722574000	-3.855754000
6	-13.136329000	-3.051916000	-3.917591000
6	-13.889980000	-1.894494000	-4.431938000
6	-15.231312000	-1.771745000	-4.823461000
1	-15.902242000	-2.634220000	-4.791213000
6	-15.695574000	-0.518554000	-5.261089000
1	-16.737791000	-0.404492000	-5.570088000
6	-14.831177000	0.591927000	-5.305653000
1	-15.210437000	1.559060000	-5.645877000
6	-13.480808000	0.468435000	-4.918730000
1	-12.812938000	1.333544000	-4.962211000
6	-13.011805000	-0.772932000	-4.483189000
6	-11.610460000	-1.187201000	-4.059335000
6	-11.792200000	-2.644964000	-3.680049000
6	-10.858817000	-3.533158000	-3.153208000
1	-9.821849000	-3.224502000	-2.997873000
6	-10.873428000	-0.219966000	-3.041928000
1	-11.533478000	0.464395000	-2.488758000
1	-10.247073000	-0.805238000	-2.347963000
6	-10.032130000	0.378593000	-4.233098000

1	-10.433021000	1.344735000	-4.579920000
1	-8.945430000	0.463032000	-4.072440000
6	-10.506089000	-0.817962000	-5.138477000
1	-9.756571000	-1.616482000	-5.213763000
1	-10.897450000	-0.578253000	-6.136169000
6	-10.692738000	-12.541421000	-3.203564000
6	-9.479653000	-12.850588000	-3.859402000
1	-8.582027000	-12.279848000	-3.617852000
6	-9.421259000	-13.885092000	-4.803254000
1	-8.465237000	-14.143577000	-5.265749000
6	-10.603984000	-14.573637000	-5.122114000
6	-10.850536000	-15.666045000	-6.081175000
6	-9.972077000	-16.373182000	-6.916790000
1	-8.902212000	-16.147440000	-6.915415000
6	-10.499146000	-17.372774000	-7.756428000
1	-9.831530000	-17.931270000	-8.417911000
6	-11.877136000	-17.663877000	-7.752600000
1	-12.266604000	-18.442860000	-8.413183000
6	-12.758014000	-16.959579000	-6.905982000
1	-13.828070000	-17.188207000	-6.905654000
6	-12.244567000	-15.962364000	-6.074149000
6	-12.969406000	-15.069532000	-5.084957000
6	-11.844154000	-14.214629000	-4.520337000
6	-11.890176000	-13.209617000	-3.553135000
1	-12.823609000	-12.892437000	-3.082014000
6	-13.892830000	-15.747893000	-3.985170000
1	-13.450041000	-15.802784000	-2.979410000
1	-14.217583000	-16.746158000	-4.322871000
6	-14.988335000	-14.648204000	-4.246204000
1	-14.944490000	-13.833613000	-3.505136000

1	-16.024117000	-15.004451000	-4.353635000
6	-14.237288000	-14.237694000	-5.568348000
1	-14.678705000	-14.694668000	-6.469469000
1	-14.057534000	-13.160266000	-5.689640000
6	-6.566251000	-6.604913000	-3.920753000
6	-6.073938000	-5.914821000	-2.817027000
1	-5.495453000	-6.451173000	-2.060276000
6	-6.324801000	-4.524899000	-2.633814000
6	-5.817088000	-3.822835000	-1.497761000
1	-5.186431000	-4.370097000	-0.792387000
6	-6.141854000	-2.492779000	-1.275822000
1	-5.753019000	-1.970087000	-0.398172000
6	-6.985432000	-1.803517000	-2.191677000
1	-7.252649000	-0.759653000	-2.007153000
6	-7.454951000	-2.441049000	-3.331359000
1	-8.065815000	-1.897305000	-4.053174000
6	-7.129871000	-3.807324000	-3.594328000
6	-7.593958000	-4.504760000	-4.763822000
6	-10.419885000	-3.852169000	-7.260386000
6	-9.959838000	-2.710472000	-7.911524000
1	-10.570446000	-2.262950000	-8.700728000
6	-8.705520000	-2.117650000	-7.594755000
6	-8.232510000	-0.964187000	-8.291395000
1	-8.871155000	-0.523710000	-9.062741000
6	-6.988861000	-0.422634000	-8.007440000
1	-6.636146000	0.458870000	-8.548333000
6	-6.157897000	-1.024409000	-7.022145000
1	-5.165898000	-0.610354000	-6.825804000
6	-6.592573000	-2.136489000	-6.315451000
1	-5.952733000	-2.603268000	-5.564817000

6	-7.884577000	-2.692717000	-6.554472000
6	-8.387090000	-3.833865000	-5.834537000
6	-6.387152000	-8.073100000	-4.079894000
6	-6.579010000	-8.909864000	-2.954704000
1	-6.877322000	-8.460719000	-2.004559000
6	-6.453227000	-10.303506000	-3.059746000
1	-6.627808000	-10.935344000	-2.184138000
6	-6.139215000	-10.864997000	-4.306282000
6	-5.975881000	-12.270358000	-4.727329000
6	-6.077607000	-13.470086000	-4.005947000
1	-6.294484000	-13.462997000	-2.934425000
6	-5.919262000	-14.688988000	-4.692446000
1	-6.011329000	-15.631776000	-4.147114000
6	-5.651563000	-14.707047000	-6.075157000
1	-5.530249000	-15.663567000	-6.590610000
6	-5.531895000	-13.500867000	-6.798520000
1	-5.320323000	-13.516554000	-7.872010000
6	-5.696910000	-12.288231000	-6.125202000
6	-5.647187000	-10.875860000	-6.675869000
6	-5.955384000	-10.029821000	-5.447557000
6	-6.069124000	-8.646387000	-5.341316000
1	-5.954139000	-8.005602000	-6.215622000
6	-6.510726000	-10.553774000	-7.969112000
1	-6.777614000	-11.480617000	-8.501578000
1	-7.398221000	-9.929723000	-7.805380000
6	-5.277202000	-9.827158000	-8.617665000
1	-5.030555000	-10.141134000	-9.644025000
1	-5.389984000	-8.736089000	-8.573927000
6	-4.354573000	-10.419712000	-7.486228000
1	-3.706682000	-9.715348000	-6.943636000

1	-3.778904000	-11.296710000	-7.824455000
6	-11.634372000	-4.571982000	-7.719820000
6	-12.514980000	-5.219663000	-6.816919000
1	-12.359962000	-5.120682000	-5.744492000
6	-13.583450000	-5.999596000	-7.282336000
1	-14.200870000	-6.540276000	-6.566862000
6	-13.801779000	-6.098268000	-8.665519000
6	-14.821699000	-6.822870000	-9.448782000
6	-15.847731000	-7.690926000	-9.041488000
1	-15.946054000	-7.975142000	-7.991595000
6	-16.717921000	-8.207931000	-10.019538000
1	-17.527001000	-8.881111000	-9.722879000
6	-16.565748000	-7.862199000	-11.377454000
1	-17.268019000	-8.255946000	-12.117211000
6	-15.511556000	-7.019851000	-11.790245000
1	-15.392148000	-6.757533000	-12.846309000
6	-14.633536000	-6.513467000	-10.828541000
6	-13.419941000	-5.612792000	-11.002685000
6	-12.955897000	-5.404556000	-9.573989000
6	-11.864385000	-4.675502000	-9.113879000
1	-11.140620000	-4.252024000	-9.813593000
6	-13.649511000	-4.316441000	-11.893413000
1	-13.128351000	-3.452253000	-11.451230000
1	-14.705074000	-4.074862000	-12.087649000
6	-12.834222000	-4.978682000	-13.067352000
1	-13.492741000	-5.373327000	-13.858857000
1	-12.041194000	-4.360070000	-13.514255000
6	-12.357649000	-6.107201000	-12.079081000
1	-11.332101000	-5.980448000	-11.697289000
1	-12.518497000	-7.141207000	-12.421213000

6	-7.559533000	-12.994745000	-13.047619000
6	-8.656717000	-12.538656000	-12.309072000
6	-7.354967000	-10.585057000	-11.594033000
6	-6.258688000	-11.056652000	-12.332598000
6	-6.360536000	-12.253404000	-13.063264000
1	-7.626573000	-13.933194000	-13.603566000
1	-9.585939000	-13.110206000	-12.256215000
1	-7.298597000	-9.671838000	-10.992655000
1	-5.322112000	-10.493263000	-12.329747000
1	-5.504178000	-12.616991000	-13.636917000
1	-13.554868000	-10.238566000	-7.422007000
1	-12.957688000	-8.575471000	-7.596652000
1	-13.332041000	-9.525300000	-9.061921000
1	-13.241884000	-14.955190000	-9.393734000
1	-11.900070000	-14.275985000	-10.366851000
1	-11.597721000	-14.919770000	-8.723048000
1	-7.880916000	-13.858943000	-7.988696000
1	-9.363136000	-14.442207000	-8.803287000
1	-8.382991000	-13.146035000	-9.551439000
1	-16.382185000	-12.328393000	-12.803414000
1	-17.352432000	-11.553530000	-11.519462000
1	-16.672189000	-10.561150000	-12.836423000
1	-14.349258000	-13.379950000	-14.049023000
1	-13.149184000	-14.549088000	-13.428383000
1	-14.371217000	-13.861120000	-12.324414000
1	-13.697066000	-11.254682000	-15.414864000
1	-15.391252000	-10.971265000	-14.919728000
1	-14.549955000	-9.777774000	-15.946884000

TS-A4:

B3LYP-D3(BJ) Energy: -8518.711774135 hartree; Imaginary frequency =   -87.9368477

16	-12.870157000	-10.846969000	-5.839491000
16	-6.934862000	-7.969903000	-7.973900000
8	-11.988147000	-8.421887000	-4.251963000
8	-9.710831000	-9.636203000	-4.076653000
8	-13.211174000	-11.174873000	-4.429007000
8	-13.072951000	-11.893812000	-6.876822000
8	-7.692166000	-6.752671000	-5.113818000
8	-9.698021000	-5.452941000	-6.154177000
8	-6.912888000	-9.105320000	-7.002756000
8	-6.832035000	-8.266666000	-9.429060000
6	-12.472204000	-12.230529000	-10.463791000
1	-11.812761000	-12.462856000	-11.317392000
8	-12.211463000	-11.174537000	-9.746710000
14	-10.843804000	-10.021172000	-9.923224000
15	-10.769008000	-9.034332000	-5.211718000
15	-8.883630000	-6.885621000	-6.285517000
7	-9.953785000	-8.014096000	-6.071920000
7	-11.453910000	-10.156793000	-6.142501000
7	-8.105533000	-6.894636000	-7.703766000
6	-12.196117000	-14.038856000	-8.363170000
1	-11.634644000	-13.558296000	-7.548312000
6	-11.628986000	-14.155870000	-9.625773000
1	-12.204805000	-14.736031000	-10.356272000
8	-13.441405000	-14.401318000	-8.128852000
14	-14.231669000	-14.603339000	-6.549813000
6	-14.139078000	-9.488477000	-6.332135000
6	-5.356120000	-6.986431000	-7.691515000
6	-11.613995000	-8.406903000	-9.339485000

6	-10.438272000	-10.062706000	-11.770023000
6	-9.451166000	-10.678281000	-8.840061000
6	-10.135539000	-14.077136000	-9.807629000
6	-15.671969000	-13.396286000	-6.314817000
6	-12.889395000	-14.628578000	-5.217246000
6	-14.878648000	-16.364418000	-6.812249000
6	-11.723438000	-7.888158000	-2.992082000
6	-10.277941000	-10.618304000	-3.250298000
6	-7.895736000	-6.130501000	-3.888658000
6	-8.858605000	-4.330599000	-6.211378000
6	-13.869773000	-12.667404000	-10.562943000
9	-14.982510000	-9.974194000	-7.324362000
9	-13.497187000	-8.362693000	-6.807974000
9	-14.893384000	-9.134178000	-5.240052000
9	-5.378664000	-5.896219000	-8.539315000
9	-5.227304000	-6.562545000	-6.383614000
9	-4.285186000	-7.799286000	-7.996133000
1	-11.002536000	-7.577573000	-9.744667000
1	-11.503026000	-8.363994000	-8.244396000
1	-10.136719000	-11.085660000	-12.068680000
1	-9.533033000	-9.442682000	-11.902428000
1	-9.165782000	-9.881079000	-8.133749000
1	-9.864365000	-11.476381000	-8.198415000
6	-13.105232000	-8.174466000	-9.700332000
6	-8.198733000	-11.163913000	-9.616177000
6	-11.567701000	-9.531259000	-12.689593000
1	-9.641211000	-13.544007000	-8.980888000
1	-9.692499000	-15.089512000	-9.842388000
1	-9.851095000	-13.574287000	-10.748846000
1	-15.464470000	-12.436574000	-6.811369000

1	-16.565285000	-13.834470000	-6.797727000
6	-15.944044000	-13.132238000	-4.804261000
1	-12.005098000	-15.105446000	-5.677739000
1	-12.587279000	-13.610344000	-4.927930000
6	-13.285027000	-15.434076000	-3.949918000
1	-15.441394000	-16.683892000	-5.914417000
1	-15.604007000	-16.345854000	-7.646906000
6	-13.738470000	-17.369473000	-7.125910000
6	-11.954512000	-6.484803000	-2.808609000
6	-11.714667000	-5.951444000	-1.545237000
1	-11.899202000	-4.888911000	-1.366394000
6	-11.231943000	-6.753115000	-0.472725000
6	-10.984035000	-6.194574000	0.818244000
1	-11.198119000	-5.134097000	0.972118000
6	-10.454636000	-6.969446000	1.838961000
1	-10.263436000	-6.529623000	2.821190000
6	-10.153546000	-8.341590000	1.610791000
1	-9.718746000	-8.940925000	2.414339000
6	-10.423234000	-8.927528000	0.382313000
1	-10.221622000	-9.987096000	0.213043000
6	-10.983154000	-8.158927000	-0.684042000
6	-11.280781000	-8.727710000	-1.972022000
6	-10.014169000	-12.001398000	-3.528390000
6	-10.593064000	-12.929239000	-2.657067000
1	-10.354976000	-13.990030000	-2.764953000
6	-11.507259000	-12.555974000	-1.635783000
6	-12.159542000	-13.535326000	-0.824086000
1	-11.918783000	-14.590430000	-0.984121000
6	-13.095641000	-13.159101000	0.125323000
1	-13.596336000	-13.916215000	0.733815000

6	-13.422386000	-11.784879000	0.301499000
1	-14.181276000	-11.500306000	1.034276000
6	-12.792603000	-10.807430000	-0.454490000
1	-13.044268000	-9.753385000	-0.326578000
6	-11.808457000	-11.161929000	-1.424138000
6	-11.115620000	-10.189645000	-2.222995000
6	-12.455804000	-5.645867000	-3.930120000
6	-13.435299000	-6.154476000	-4.820213000
1	-13.824267000	-7.157720000	-4.678939000
6	-13.908727000	-5.387093000	-5.890746000
1	-14.631911000	-5.819165000	-6.587343000
6	-13.442902000	-4.075350000	-6.045221000
6	-13.779003000	-3.042357000	-7.039693000
6	-14.609888000	-3.094248000	-8.169806000
1	-15.159196000	-4.007660000	-8.415866000
6	-14.717973000	-1.950169000	-8.980995000
1	-15.355388000	-1.971908000	-9.868593000
6	-14.007842000	-0.776831000	-8.663135000
1	-14.098660000	0.100763000	-9.308118000
6	-13.173466000	-0.724645000	-7.527832000
1	-12.616515000	0.187170000	-7.295380000
6	-13.059515000	-1.856086000	-6.716399000
6	-12.197943000	-2.085172000	-5.482279000
6	-12.481646000	-3.539863000	-5.140224000
6	-11.972759000	-4.322159000	-4.102699000
1	-11.190860000	-3.940404000	-3.439249000
6	-12.394517000	-1.014212000	-4.322372000
1	-13.331778000	-0.440896000	-4.370376000
1	-12.281435000	-1.502987000	-3.340382000
6	-11.068067000	-0.296561000	-4.775323000

1	-11.267532000	0.593722000	-5.393373000
1	-10.353912000	-0.042539000	-3.976031000
6	-10.711458000	-1.556050000	-5.647005000
1	-10.005319000	-2.231635000	-5.147604000
1	-10.392103000	-1.386126000	-6.684138000
6	-9.166312000	-12.460404000	-4.665091000
6	-8.180948000	-11.640458000	-5.274653000
1	-8.032460000	-10.616706000	-4.946919000
6	-7.358804000	-12.129991000	-6.298033000
1	-6.635187000	-11.454717000	-6.754059000
6	-7.497539000	-13.456815000	-6.716616000
6	-6.769279000	-14.230888000	-7.734936000
6	-5.714829000	-13.866066000	-8.585132000
1	-5.287175000	-12.860774000	-8.538890000
6	-5.223093000	-14.820362000	-9.494627000
1	-4.405357000	-14.553612000	-10.169132000
6	-5.772122000	-16.116296000	-9.547291000
1	-5.377276000	-16.841884000	-10.263141000
6	-6.822215000	-16.488228000	-8.681184000
1	-7.237664000	-17.500208000	-8.717971000
6	-7.316628000	-15.545497000	-7.777765000
6	-8.404212000	-15.696679000	-6.728901000
6	-8.494883000	-14.292266000	-6.136960000
6	-9.327495000	-13.795272000	-5.139708000
1	-10.113980000	-14.426545000	-4.723393000
6	-8.202703000	-16.811354000	-5.609325000
1	-7.832318000	-16.429488000	-4.646875000
1	-7.557497000	-17.620553000	-5.988939000
6	-9.731693000	-17.174838000	-5.729435000
1	-10.334102000	-16.668613000	-4.956868000

1	-9.986718000	-18.244932000	-5.761636000
6	-9.774662000	-16.395544000	-7.097537000
1	-9.642186000	-17.073459000	-7.957604000
1	-10.621609000	-15.718191000	-7.275870000
6	-7.671735000	-6.915956000	-2.712308000
6	-7.898911000	-6.315548000	-1.476235000
1	-7.720035000	-6.887360000	-0.561920000
6	-8.358338000	-4.973099000	-1.367495000
6	-8.627856000	-4.377071000	-0.097732000
1	-8.473361000	-4.982086000	0.798668000
6	-9.105206000	-3.078248000	-0.006856000
1	-9.317439000	-2.637120000	0.970489000
6	-9.314871000	-2.311027000	-1.187420000
1	-9.698661000	-1.290337000	-1.111698000
6	-9.016058000	-2.843195000	-2.433489000
1	-9.138144000	-2.241195000	-3.335098000
6	-8.528195000	-4.180101000	-2.560661000
6	-8.223976000	-4.772921000	-3.834915000
6	-8.745059000	-3.658816000	-7.458992000
6	-7.906647000	-2.557478000	-7.545859000
1	-7.819440000	-2.024869000	-8.496906000
6	-7.144049000	-2.118210000	-6.424173000
6	-6.262256000	-0.998939000	-6.520146000
1	-6.213106000	-0.455512000	-7.468288000
6	-5.476436000	-0.618900000	-5.444197000
1	-4.803129000	0.237488000	-5.529471000
6	-5.528101000	-1.357569000	-4.229850000
1	-4.882493000	-1.071592000	-3.395897000
6	-6.380950000	-2.443199000	-4.097605000
1	-6.411859000	-3.013634000	-3.168721000

6	-7.232975000	-2.838267000	-5.173445000
6	-8.139104000	-3.957932000	-5.080771000
6	-7.156617000	-8.308430000	-2.805258000
6	-7.688654000	-9.286197000	-1.933534000
1	-8.499221000	-9.001004000	-1.260029000
6	-7.256455000	-10.618089000	-1.988930000
1	-7.735351000	-11.378566000	-1.366800000
6	-6.237057000	-10.960754000	-2.887999000
6	-5.668538000	-12.272525000	-3.241248000
6	-6.058963000	-13.567540000	-2.872022000
1	-6.873469000	-13.721138000	-2.158956000
6	-5.418002000	-14.663299000	-3.477950000
1	-5.723252000	-15.681278000	-3.219803000
6	-4.403026000	-14.464646000	-4.433560000
1	-3.934331000	-15.327999000	-4.912377000
6	-4.008087000	-13.162344000	-4.801738000
1	-3.237635000	-13.014627000	-5.564142000
6	-4.645369000	-12.069596000	-4.210696000
6	-4.475536000	-10.589077000	-4.497770000
6	-5.634105000	-9.966776000	-3.716923000
6	-6.117718000	-8.658285000	-3.713161000
1	-5.741047000	-7.920553000	-4.421597000
6	-4.238082000	-10.104013000	-5.981174000
1	-3.804803000	-10.923498000	-6.580637000
1	-5.117689000	-9.676037000	-6.470443000
6	-3.134927000	-9.104109000	-5.475713000
1	-2.200925000	-9.062238000	-6.056493000
1	-3.549724000	-8.090638000	-5.362329000
6	-3.082485000	-9.898437000	-4.117602000
1	-3.086929000	-9.324276000	-3.179545000

1	-2.279950000	-10.654436000	-4.115142000
6	-9.489424000	-4.175229000	-8.638900000
6	-10.898351000	-4.126527000	-8.680892000
1	-11.455807000	-3.722617000	-7.835905000
6	-11.591170000	-4.585972000	-9.811862000
1	-12.680986000	-4.509610000	-9.845778000
6	-10.859244000	-5.109461000	-10.888835000
6	-11.298847000	-5.610990000	-12.205834000
6	-12.581788000	-5.763498000	-12.754546000
1	-13.470301000	-5.503227000	-12.172884000
6	-12.701971000	-6.256512000	-14.067480000
1	-13.693624000	-6.379855000	-14.510660000
6	-11.558728000	-6.594760000	-14.816815000
1	-11.672792000	-6.972667000	-15.836187000
6	-10.270153000	-6.460077000	-14.260057000
1	-9.384271000	-6.738984000	-14.838630000
6	-10.142065000	-5.968229000	-12.959303000
6	-8.895184000	-5.813879000	-12.110249000
6	-9.437246000	-5.198515000	-10.828501000
6	-8.751782000	-4.733052000	-9.707286000
1	-7.670862000	-4.842235000	-9.597097000
6	-7.569554000	-5.130628000	-12.644800000
1	-7.402503000	-4.102284000	-12.290192000
1	-7.529545000	-5.174911000	-13.746382000
6	-6.727488000	-6.291724000	-11.985863000
1	-5.921816000	-6.720076000	-12.601444000
1	-6.340776000	-6.048103000	-10.983200000
6	-8.046709000	-7.137362000	-11.873453000
1	-8.203243000	-7.647593000	-10.916834000
1	-8.173981000	-7.818117000	-12.732490000

6	-16.148999000	-12.605446000	-9.726548000
6	-14.810469000	-12.203642000	-9.620553000
6	-14.270707000	-13.538356000	-11.600887000
6	-15.607495000	-13.937928000	-11.696827000
6	-16.549470000	-13.471942000	-10.757639000
1	-16.878318000	-12.246876000	-8.996517000
1	-14.472403000	-11.546939000	-8.820768000
1	-13.533814000	-13.887531000	-12.331861000
1	-15.923011000	-14.604501000	-12.502981000
1	-17.594051000	-13.784086000	-10.835771000
1	-13.744029000	-8.972142000	-9.288287000
1	-13.436830000	-7.214354000	-9.263914000
1	-13.254151000	-8.125779000	-10.791948000
1	-7.559898000	-11.767167000	-8.951634000
1	-8.455960000	-11.793006000	-10.486339000
1	-7.611099000	-10.295711000	-9.954004000
1	-11.283334000	-9.614682000	-13.753207000
1	-12.511293000	-10.089412000	-12.539852000
1	-11.762490000	-8.464847000	-12.488633000
1	-15.090373000	-12.593630000	-4.356686000
1	-16.846561000	-12.506764000	-4.681017000
1	-16.109716000	-14.071194000	-4.244775000
1	-13.625522000	-16.454016000	-4.203095000
1	-12.416824000	-15.523537000	-3.271148000
1	-14.084675000	-14.920097000	-3.390655000
1	-13.195354000	-17.054863000	-8.034879000
1	-13.010890000	-17.418890000	-6.294515000
1	-14.133106000	-18.387389000	-7.294408000

Anti Selective Aldol Reaction:

TS-B1:

B3LYP-D3(BJ) Energy: -8320.22171489 hartree; Imaginary frequency =   -26.30508329

16	-7.156240000	30.956368000	5.876006000
16	-12.307861000	33.202281000	4.050932000
15	-7.688441000	33.401728000	4.460997000
15	-10.201189000	35.131181000	4.514748000
7	-8.751629000	34.547627000	4.364227000
7	-8.164387000	32.059896000	5.215841000
7	-11.465636000	34.237529000	4.948334000
8	-6.292940000	33.990682000	5.215428000
8	-7.149331000	33.174928000	2.895537000
8	-5.800668000	30.834076000	5.280325000
8	-7.938126000	29.707401000	6.113817000
8	-10.033572000	36.308435000	5.681156000
8	-10.589690000	36.045830000	3.162967000
8	-13.289734000	32.494374000	4.934404000
8	-12.818323000	33.742219000	2.760951000
6	-14.002237000	29.712507000	6.827916000
1	-13.819441000	30.615054000	6.225918000
8	-13.011201000	29.117355000	7.416752000
14	-11.259560000	29.548980000	7.195554000
6	-14.307335000	28.214903000	4.925730000
1	-13.286828000	27.846805000	5.051805000
6	-14.433645000	29.332295000	4.135097000
1	-13.552524000	29.874076000	3.771288000
8	-15.631388000	29.829637000	3.815907000
14	-15.999851000	31.008565000	2.566979000
6	-6.865624000	31.684675000	7.584932000
6	-11.043976000	31.895655000	3.628153000

6	-11.193155000	31.390701000	7.485821000
6	-10.882323000	28.932295000	5.467544000
6	-10.454022000	28.533038000	8.597210000
6	-15.432751000	27.305799000	5.305184000
6	-14.631628000	30.935672000	1.283970000
6	-16.207381000	32.695514000	3.365303000
6	-17.650410000	30.285317000	1.961684000
6	-5.658796000	35.045048000	4.539915000
6	-5.893487000	32.591645000	2.710651000
6	-10.938042000	37.365728000	5.692266000
6	-9.702968000	37.099102000	2.901368000
6	-15.349802000	29.477649000	7.341780000
9	-6.425516000	30.640071000	8.388552000
9	-8.100045000	32.155206000	8.073394000
9	-11.729602000	30.862065000	2.934777000
1	-6.131775000	32.502866000	7.528846000
9	-10.168938000	32.539901000	2.738065000
1	-10.500199000	31.503513000	4.499297000
1	-10.146280000	31.731866000	7.490495000
1	-11.624653000	31.621967000	8.474246000
1	-11.467767000	29.405277000	4.666543000
1	-9.822499000	29.138232000	5.246454000
6	-10.041110000	27.130792000	8.085186000
1	-11.749605000	31.985244000	6.744936000
1	-11.022158000	27.842864000	5.376510000
6	-9.186676000	29.272486000	9.107752000
6	-11.447940000	28.376250000	9.775820000
1	-15.388823000	26.388017000	4.687812000
1	-15.359780000	26.992817000	6.362044000
1	-16.413922000	27.781256000	5.157175000

1	-14.781924000	31.723823000	0.526627000
1	-13.641996000	31.113953000	1.732296000
1	-14.612755000	29.958763000	0.769818000
1	-16.869926000	33.323797000	2.743018000
1	-15.235100000	33.202628000	3.459786000
6	-18.210903000	31.145241000	0.801777000
6	-17.431583000	28.830303000	1.475892000
6	-18.654848000	30.279129000	3.143901000
1	-16.656223000	32.624590000	4.371209000
6	-4.925557000	34.768708000	3.384613000
6	-4.393069000	35.853291000	2.605290000
6	-3.770758000	35.648841000	1.335208000
1	-3.675090000	34.627847000	0.960650000
6	-3.305862000	36.725707000	0.594959000
1	-2.843400000	36.555539000	-0.380277000
6	-3.423113000	38.053692000	1.095676000
1	-3.033300000	38.888974000	0.508423000
6	-4.032725000	38.284190000	2.319126000
1	-4.142762000	39.298447000	2.712031000
6	-4.558904000	37.201184000	3.088692000
6	-5.261573000	37.423759000	4.303862000
1	-5.375603000	38.448353000	4.667340000
6	-5.840376000	36.379996000	5.023217000
6	-4.756060000	33.357438000	2.944813000
6	-3.455725000	32.765045000	2.784424000
6	-2.248505000	33.463472000	3.088363000
1	-2.316077000	34.484290000	3.468820000
6	-1.014864000	32.855971000	2.906273000
1	-0.099460000	33.402649000	3.145907000
6	-0.929467000	31.522168000	2.418149000

1	0.050384000	31.059167000	2.277761000
6	-2.084482000	30.809152000	2.137890000
1	-2.030971000	29.776092000	1.781841000
6	-3.371899000	31.399293000	2.322282000
6	-4.569561000	30.665350000	2.090599000
1	-4.501407000	29.626029000	1.757196000
6	-5.823504000	31.224850000	2.304209000
6	-6.610171000	36.688579000	6.256097000
6	-6.434396000	35.950436000	7.454768000
1	-5.768648000	35.085585000	7.457141000
6	-7.091593000	36.363510000	8.613951000
6	-7.020999000	35.804860000	10.032794000
6	-5.533443000	35.657886000	10.578789000
1	-4.779501000	36.255688000	10.046555000
1	-5.505542000	35.862070000	11.661835000
6	-5.598523000	34.114023000	10.275776000
1	-5.141047000	33.875847000	9.300684000
1	-5.203662000	33.437589000	11.048478000
6	-7.166558000	34.234828000	10.166370000
1	-7.650744000	33.987086000	11.126885000
1	-7.664627000	33.717247000	9.339527000
6	-7.989879000	36.691817000	10.791550000
6	-8.396716000	36.613242000	12.125031000
1	-7.991328000	35.843543000	12.788951000
6	-9.348596000	37.537298000	12.602102000
1	-9.682038000	37.484340000	13.641573000
6	-9.883901000	38.521092000	11.748514000
1	-10.630854000	39.219942000	12.133442000
6	-9.477828000	38.606112000	10.404809000
1	-9.910168000	39.356424000	9.737418000

6	-8.524918000	37.691965000	9.932406000
6	-7.949834000	37.503168000	8.588204000
6	-8.146480000	38.220153000	7.400548000
1	-8.829560000	39.072707000	7.362425000
6	-7.473601000	37.808560000	6.243781000
1	-7.648130000	38.332948000	5.304467000
6	-7.057072000	30.408847000	2.185274000
6	-7.101992000	29.149079000	2.836115000
1	-6.255746000	28.848713000	3.458473000
6	-8.259934000	28.377398000	2.744718000
6	-8.573248000	27.006990000	3.333246000
6	-8.014091000	26.759143000	4.793766000
1	-7.811564000	27.676272000	5.365095000
1	-8.681338000	26.079128000	5.349686000
6	-6.785738000	25.991467000	4.174781000
1	-6.496694000	25.045222000	4.656675000
1	-5.907603000	26.650659000	4.081927000
6	-7.573238000	25.878733000	2.816884000
1	-8.104302000	24.916349000	2.728669000
1	-7.039452000	26.119969000	1.886117000
6	-10.047277000	26.836872000	3.011561000
6	-10.930049000	25.824936000	3.397069000
1	-10.584500000	24.989747000	4.014148000
6	-12.279849000	25.903144000	2.990884000
1	-12.983402000	25.121778000	3.291367000
6	-12.732096000	26.982667000	2.205659000
1	-13.784503000	27.034216000	1.912613000
6	-11.848530000	28.005394000	1.813903000
1	-12.202289000	28.856150000	1.226052000
6	-10.506684000	27.926274000	2.215580000

6	-9.390868000	28.870125000	2.032059000
6	-9.340173000	30.104676000	1.372643000
1	-10.195555000	30.477356000	0.808565000
6	-8.172786000	30.874428000	1.454566000
1	-8.135544000	31.848740000	0.971398000
6	-10.869893000	38.351063000	4.707228000
6	-11.833229000	39.420503000	4.712466000
6	-11.894206000	40.403460000	3.678376000
1	-11.191886000	40.330635000	2.846127000
6	-12.829653000	41.426257000	3.727496000
1	-12.864840000	42.168633000	2.926385000
6	-13.752570000	41.512926000	4.807043000
1	-14.479956000	42.328044000	4.833054000
6	-13.739450000	40.557143000	5.810503000
1	-14.458657000	40.601111000	6.633825000
6	-12.797328000	39.483793000	5.786768000
6	-12.806293000	38.460635000	6.777888000
1	-13.544125000	38.503436000	7.584296000
6	-11.914335000	37.395896000	6.732302000
6	-9.807470000	38.260454000	3.667176000
6	-8.847158000	39.316530000	3.486177000
6	-8.804613000	40.472159000	4.325595000
1	-9.549411000	40.569302000	5.117742000
6	-7.833809000	41.445608000	4.141460000
1	-7.807378000	42.317621000	4.799360000
6	-6.871122000	41.320165000	3.100049000
1	-6.125246000	42.105886000	2.955564000
6	-6.879192000	40.204444000	2.277487000
1	-6.139003000	40.085621000	1.481747000
6	-7.840365000	39.163490000	2.464757000

6	-7.807912000	37.976412000	1.683237000
1	-7.046342000	37.882535000	0.904916000
6	-8.698025000	36.925179000	1.897279000
6	-12.018944000	36.261273000	7.683488000
6	-13.268747000	35.613344000	7.805743000
1	-14.101236000	35.938680000	7.176980000
6	-13.391616000	34.527856000	8.667462000
6	-14.583580000	33.614254000	8.892747000
6	-15.257122000	33.114792000	7.547833000
1	-14.617348000	33.156492000	6.653046000
1	-15.666170000	32.105362000	7.699268000
6	-16.383839000	34.199115000	7.725108000
1	-16.181676000	35.104384000	7.130557000
1	-17.418762000	33.861841000	7.555796000
6	-15.939701000	34.369882000	9.227758000
1	-16.556590000	33.761063000	9.909466000
6	-14.042267000	32.599731000	9.881529000
6	-14.675657000	31.486747000	10.437867000
1	-15.699927000	31.226113000	10.155095000
6	-13.967304000	30.691417000	11.362208000
1	-14.449166000	29.815542000	11.804602000
6	-12.641513000	31.011257000	11.716519000
1	-12.110390000	30.385838000	12.439278000
6	-11.988339000	32.117773000	11.140724000
1	-10.952716000	32.352432000	11.402493000
6	-12.692512000	32.912167000	10.222218000
6	-12.278275000	34.094521000	9.441401000
6	-11.035259000	34.734878000	9.320675000
1	-10.170300000	34.406781000	9.903079000
6	-10.905186000	35.806616000	8.425524000

1	-9.941817000	36.296758000	8.299800000
6	-8.600538000	35.693274000	1.073634000
6	-9.758399000	35.023256000	0.597326000
1	-10.746343000	35.338453000	0.934808000
6	-9.609502000	33.960027000	-0.290904000
6	-10.680277000	33.134905000	-0.994219000
6	-11.812707000	34.009102000	-1.688421000
1	-11.544027000	35.062778000	-1.854164000
1	-12.139180000	33.535330000	-2.629117000
6	-12.780264000	33.678982000	-0.489679000
1	-12.819921000	34.468224000	0.276267000
1	-13.801697000	33.369494000	-0.761991000
6	-11.799072000	32.538442000	-0.040715000
1	-12.114393000	31.541601000	-0.393352000
1	-11.531415000	32.527930000	1.016626000
6	-9.861822000	32.161912000	-1.820459000
6	-10.297479000	31.120450000	-2.642399000
1	-11.366091000	30.937094000	-2.790756000
6	-9.338366000	30.301636000	-3.272606000
1	-9.664695000	29.480137000	-3.915513000
6	-7.962405000	30.525104000	-3.073230000
1	-7.233961000	29.872444000	-3.561132000
6	-7.516093000	31.571229000	-2.245971000
1	-6.447684000	31.733231000	-2.080082000
6	-8.469450000	32.391093000	-1.624629000
6	-8.313569000	33.524020000	-0.693765000
6	-7.165308000	34.147276000	-0.182833000
1	-6.165314000	33.789591000	-0.442805000
6	-7.317160000	35.229185000	0.693221000
1	-6.431809000	35.695832000	1.128353000

1	-15.819277000	35.391823000	9.616512000
6	-15.529543000	28.624737000	8.454463000
6	-16.810104000	28.436491000	8.988014000
6	-17.917852000	29.084834000	8.408991000
6	-17.742863000	29.922930000	7.291046000
6	-16.464165000	30.125569000	6.758038000
1	-14.650226000	28.157613000	8.901721000
1	-16.949123000	27.790639000	9.858316000
1	-18.915922000	28.939248000	8.829678000
1	-18.603459000	30.419987000	6.836911000
1	-16.320811000	30.756648000	5.881557000
1	-9.594321000	26.554782000	8.919345000
1	-10.913455000	26.565473000	7.704046000
1	-9.292205000	27.214045000	7.279239000
1	-9.423176000	30.295181000	9.454204000
1	-8.764971000	28.705053000	9.961387000
1	-8.417344000	29.348208000	8.324338000
1	-10.941748000	27.834992000	10.599972000
1	-11.783796000	29.355334000	10.157818000
1	-12.339876000	27.800222000	9.472347000
1	-17.515974000	31.157331000	-0.059095000
1	-19.175611000	30.723648000	0.457196000
1	-18.390368000	32.188199000	1.124172000
1	-17.024117000	28.207178000	2.292543000
1	-18.396824000	28.396290000	1.149179000
1	-16.732298000	28.794972000	0.619448000
1	-18.850751000	31.305953000	3.506385000
1	-19.616616000	29.840471000	2.812589000
1	-18.262344000	29.673786000	3.981611000

TS-B2:

B3LYP-D3(BJ) Energy: -8320.217600630 hartree; Imaginary frequency = -89.15791803

16	-7.435733000	30.895958000	5.602028000
16	-12.453759000	32.525158000	3.400597000
15	-7.976904000	33.273200000	4.062138000
15	-10.646222000	34.607443000	4.080378000
7	-9.127277000	34.317186000	3.782182000
7	-8.428085000	31.967799000	4.894535000
7	-11.723890000	33.482636000	4.492772000
8	-6.678219000	34.032968000	4.826486000
8	-7.330886000	32.968708000	2.553352000
8	-6.034200000	30.837515000	5.109861000
8	-8.194049000	29.634288000	5.822810000
8	-10.548185000	35.716760000	5.324261000
8	-11.337142000	35.541581000	2.887190000
8	-13.541059000	31.773727000	4.103705000
8	-12.818657000	33.178339000	2.116125000
6	-14.122879000	29.696407000	6.179268000
1	-13.595301000	30.391516000	5.514369000
8	-13.409118000	28.852389000	6.902384000
14	-11.620081000	28.980853000	6.744358000
6	-14.710595000	31.202916000	7.674073000
1	-13.770588000	31.070615000	8.218405000
6	-14.736359000	32.318988000	6.840397000
1	-13.811361000	32.851857000	6.574932000
8	-15.862087000	32.718970000	6.284195000
14	-16.242103000	34.160973000	5.334604000
6	-7.221720000	31.625263000	7.342629000
6	-11.092540000	31.289264000	3.061368000
6	-11.191465000	30.768726000	7.105218000

6	-11.268932000	28.396601000	5.001270000
6	-11.057335000	27.799086000	8.112063000
6	-15.957360000	30.630450000	8.290788000
6	-17.229710000	33.473141000	3.893029000
6	-14.714827000	35.103650000	4.816980000
6	-17.312995000	35.130506000	6.572626000
6	-6.154574000	35.123187000	4.115727000
6	-6.003651000	32.550109000	2.451673000
6	-11.603008000	36.550340000	5.640594000
6	-10.736599000	36.788729000	2.699184000
6	-15.460015000	29.280866000	5.748887000
9	-8.172174000	31.065252000	8.224597000
9	-7.469936000	33.003289000	7.259731000
9	-11.667300000	30.258254000	2.307350000
1	-6.200313000	31.421976000	7.697868000
9	-10.193751000	32.021132000	2.269538000
1	-10.604964000	30.922983000	3.976186000
1	-10.100196000	30.851247000	7.203975000
1	-11.607557000	31.104986000	8.069074000
1	-11.810960000	28.952916000	4.219858000
1	-10.192785000	28.498790000	4.782672000
6	-11.463839000	26.348215000	7.747988000
1	-11.505637000	31.488982000	6.331961000
1	-11.528675000	27.333604000	4.864978000
6	-9.516389000	27.877870000	8.279119000
6	-11.757337000	28.216687000	9.430994000
1	-15.804945000	29.578044000	8.581130000
1	-16.216149000	31.192375000	9.206883000
1	-16.811906000	30.679833000	7.599171000
1	-17.740645000	34.289372000	3.352738000

1	-16.559443000	32.962811000	3.180652000
1	-17.991095000	32.754222000	4.238967000
1	-13.925096000	35.103868000	5.584337000
1	-14.981766000	36.163369000	4.655049000
6	-17.809424000	36.441088000	5.908434000
6	-18.522214000	34.269276000	7.013472000
6	-16.452307000	35.484068000	7.810543000
1	-14.280683000	34.719107000	3.881639000
6	-5.345724000	34.876928000	3.005057000
6	-4.949474000	35.971037000	2.159926000
6	-4.245918000	35.776525000	0.932171000
1	-3.936051000	34.764757000	0.663572000
6	-3.972462000	36.849372000	0.095930000
1	-3.445359000	36.682408000	-0.846477000
6	-4.380860000	38.166608000	0.448546000
1	-4.159135000	38.999039000	-0.223737000
6	-5.062047000	38.388776000	1.635006000
1	-5.390193000	39.394415000	1.915797000
6	-5.372364000	37.304824000	2.513167000
6	-6.119052000	37.507378000	3.705594000
1	-6.398335000	38.526964000	3.984868000
6	-6.533581000	36.446874000	4.509534000
6	-4.982849000	33.471950000	2.667297000
6	-3.612918000	33.043069000	2.583898000
6	-2.516996000	33.909824000	2.876388000
1	-2.729738000	34.934758000	3.186274000
6	-1.209660000	33.458257000	2.770252000
1	-0.381265000	34.133208000	2.999376000
6	-0.936438000	32.119432000	2.373432000
1	0.099115000	31.780117000	2.291357000

6	-1.979675000	31.246508000	2.106807000
1	-1.781030000	30.209808000	1.819197000
6	-3.338156000	31.673984000	2.214127000
6	-4.422706000	30.777962000	1.992142000
1	-4.209418000	29.739485000	1.723722000
6	-5.745213000	31.181135000	2.130518000
6	-7.347824000	36.732959000	5.721885000
6	-7.098941000	36.107924000	6.975607000
1	-6.352828000	35.314669000	7.045616000
6	-7.839556000	36.506409000	8.091209000
6	-7.722292000	36.104353000	9.561076000
6	-6.365775000	36.667693000	10.204095000
1	-5.865258000	37.452480000	9.619110000
1	-6.553633000	37.001906000	11.237230000
6	-5.767417000	35.214890000	10.149601000
1	-5.125674000	35.076104000	9.263869000
1	-5.245621000	34.865598000	11.053359000
6	-7.216969000	34.650871000	9.899565000
1	-7.694735000	34.271326000	10.817689000
1	-7.321989000	33.939239000	9.077757000
6	-8.988049000	36.680953000	10.167593000
6	-9.526768000	36.499698000	11.441710000
1	-9.055422000	35.819634000	12.157034000
6	-10.707355000	37.191686000	11.786666000
1	-11.145007000	37.053498000	12.779276000
6	-11.327204000	38.058432000	10.865055000
1	-12.240787000	38.586640000	11.151795000
6	-10.785915000	38.247693000	9.580093000
1	-11.281041000	38.899654000	8.855862000
6	-9.618073000	37.552288000	9.234339000

6	-8.887406000	37.468117000	7.958371000
6	-9.125710000	38.092519000	6.729439000
1	-9.925865000	38.826436000	6.613938000
6	-8.347542000	37.728656000	5.624161000
1	-8.563596000	38.175542000	4.653825000
6	-6.863267000	30.210025000	2.016335000
6	-6.788816000	28.994363000	2.745261000
1	-5.937198000	28.831449000	3.409185000
6	-7.849203000	28.090501000	2.677080000
6	-8.030229000	26.734290000	3.352660000
6	-7.619268000	26.684917000	4.883616000
1	-7.614612000	27.665537000	5.377350000
1	-8.260877000	25.967997000	5.424888000
6	-6.240336000	26.043586000	4.473775000
1	-5.893107000	25.186312000	5.070540000
1	-5.446428000	26.805745000	4.411919000
6	-6.843778000	25.718004000	3.056003000
1	-7.232819000	24.687583000	3.001861000
1	-6.237237000	25.955232000	2.169607000
6	-9.442947000	26.350436000	2.955975000
6	-10.206680000	25.255929000	3.366655000
1	-9.782219000	24.496962000	4.031291000
6	-11.544419000	25.154937000	2.929123000
1	-12.156799000	24.307080000	3.247369000
6	-12.104073000	26.145938000	2.098561000
1	-13.149299000	26.062599000	1.789452000
6	-11.340094000	27.250670000	1.680634000
1	-11.786273000	28.046070000	1.079558000
6	-10.005100000	27.341363000	2.101681000
6	-9.002171000	28.402684000	1.899404000

6	-9.063086000	29.587570000	1.153791000
1	-9.935203000	29.825634000	0.544708000
6	-7.994976000	30.492117000	1.219130000
1	-8.047029000	31.425916000	0.662988000
6	-11.969387000	37.554972000	4.737261000
6	-13.117946000	38.366447000	5.025514000
6	-13.673317000	39.270556000	4.071298000
1	-13.204542000	39.337928000	3.087036000
6	-14.789778000	40.031698000	4.385800000
1	-15.214345000	40.709144000	3.641111000
6	-15.394511000	39.932293000	5.671336000
1	-16.272135000	40.540218000	5.904227000
6	-14.878377000	39.062029000	6.619807000
1	-15.345965000	38.966986000	7.604564000
6	-13.741839000	38.246937000	6.322251000
6	-13.236402000	37.302447000	7.257818000
1	-13.663847000	37.290417000	8.263442000
6	-12.233116000	36.388105000	6.924960000
6	-11.082588000	37.826084000	3.562775000
6	-10.413487000	39.098549000	3.426158000
6	-10.627052000	40.192918000	4.318876000
1	-11.381441000	40.092241000	5.100110000
6	-9.891095000	41.363583000	4.201447000
1	-10.064822000	42.184451000	4.901358000
6	-8.907021000	41.502759000	3.184567000
1	-8.335178000	42.430571000	3.106693000
6	-8.672098000	40.459873000	2.302801000
1	-7.911925000	40.547064000	1.520303000
6	-9.402098000	39.235064000	2.401045000
6	-9.099743000	38.135215000	1.550082000

1	-8.322642000	38.254306000	0.790157000
6	-9.732752000	36.903222000	1.689269000
6	-11.853452000	35.295532000	7.862632000
6	-12.785378000	34.878279000	8.856116000
1	-13.772250000	35.341888000	8.910051000
6	-12.447412000	33.868654000	9.752351000
6	-13.218267000	33.322883000	10.947454000
6	-14.775760000	33.162425000	10.740736000
1	-15.090061000	33.103598000	9.688365000
1	-15.166010000	32.303835000	11.312201000
6	-15.006268000	34.533443000	11.481323000
1	-15.146493000	35.360884000	10.766439000
1	-15.803265000	34.554999000	12.239776000
6	-13.526706000	34.465126000	12.017404000
1	-13.473955000	34.055917000	13.039520000
6	-12.347741000	32.163729000	11.405370000
6	-12.556291000	31.243858000	12.434427000
1	-13.477871000	31.262315000	13.024176000
6	-11.557192000	30.283640000	12.702729000
1	-11.710459000	29.552275000	13.500320000
6	-10.367934000	30.251037000	11.949100000
1	-9.615093000	29.488011000	12.162877000
6	-10.148997000	31.170253000	10.906196000
1	-9.245439000	31.128486000	10.292407000
6	-11.142659000	32.126351000	10.643984000
6	-11.201417000	33.194404000	9.635470000
6	-10.280981000	33.582745000	8.660186000
1	-9.341691000	33.055971000	8.531552000
6	-10.592713000	34.643000000	7.805781000
1	-9.847056000	34.973454000	7.091850000

6	-9.308943000	35.735832000	0.872004000
6	-10.233367000	34.804151000	0.325942000
1	-11.294729000	34.882315000	0.563796000
6	-9.755169000	33.770814000	-0.482642000
6	-10.521335000	32.723559000	-1.291031000
6	-11.657367000	33.358773000	-2.211298000
1	-11.562203000	34.441324000	-2.381605000
1	-11.718488000	32.813712000	-3.167905000
6	-12.740211000	32.866244000	-1.179061000
1	-13.028504000	33.645269000	-0.456281000
1	-13.637460000	32.386240000	-1.598730000
6	-11.662843000	31.921258000	-0.539318000
1	-11.718976000	30.891999000	-0.933946000
1	-11.595743000	31.929371000	0.546304000
6	-9.408283000	31.925427000	-1.943147000
6	-9.494065000	30.783108000	-2.742148000
1	-10.467477000	30.359167000	-3.006880000
6	-8.304903000	30.174691000	-3.192939000
1	-8.357203000	29.275228000	-3.811700000
6	-7.048810000	30.705589000	-2.841259000
1	-6.138045000	30.210589000	-3.187855000
6	-6.954118000	31.856071000	-2.038413000
1	-5.978455000	32.254415000	-1.746688000
6	-8.137766000	32.465083000	-1.594838000
6	-8.355510000	33.629642000	-0.720415000
6	-7.439598000	34.520632000	-0.147021000
1	-6.363061000	34.398316000	-0.288118000
6	-7.922494000	35.572347000	0.637844000
1	-7.215245000	36.242984000	1.127247000
1	-12.898583000	35.361918000	11.912934000

6	-16.070291000	28.141354000	6.316458000
6	-17.356846000	27.770498000	5.910174000
6	-18.033247000	28.529492000	4.934891000
6	-17.412113000	29.648419000	4.349423000
6	-16.124732000	30.029489000	4.750225000
1	-15.509985000	27.562849000	7.054385000
1	-17.835192000	26.888506000	6.343153000
1	-19.038164000	28.237156000	4.619328000
1	-17.928129000	30.217146000	3.571832000
1	-15.597341000	30.863097000	4.281712000
1	-11.162503000	25.665350000	8.566808000
1	-12.558946000	26.268255000	7.612797000
1	-10.967408000	26.015981000	6.816995000
1	-9.165164000	28.908796000	8.456967000
1	-9.213397000	27.244128000	9.136600000
1	-9.001112000	27.511802000	7.373636000
1	-11.467113000	27.517821000	10.240583000
1	-11.457779000	29.231352000	9.744237000
1	-12.856701000	28.186026000	9.316916000
1	-18.402196000	36.232589000	4.997722000
1	-18.460171000	36.987231000	6.619968000
1	-16.966003000	37.105713000	5.643703000
1	-18.180244000	33.321200000	7.466728000
1	-19.120560000	34.825044000	7.761908000
1	-19.177471000	34.032674000	6.154742000
1	-15.570762000	36.081533000	7.514228000
1	-17.053994000	36.075604000	8.529207000
1	-16.105471000	34.569245000	8.325109000

TS-B3:

B3LYP-D3(BJ) Energy: -8320.21626716 hartree; Imaginary frequency = -117.26757528

16	-9.344028000	33.255315000	7.333088000
16	-11.820187000	33.824641000	2.134696000
15	-7.987628000	33.228784000	4.812139000
15	-9.516927000	35.173852000	3.113030000
7	-8.361936000	34.492341000	3.941921000
7	-9.002838000	32.594767000	5.880400000
7	-11.054191000	34.710525000	3.269766000
8	-6.540842000	33.509032000	5.585914000
8	-7.576566000	32.079062000	3.679868000
8	-8.163151000	33.893720000	7.981940000
8	-10.101156000	32.241894000	8.123816000
8	-9.317517000	36.755732000	3.601467000
8	-9.220113000	35.288739000	1.459123000
8	-10.920791000	32.868133000	1.417526000
8	-13.096986000	33.315671000	2.703452000
6	-10.625459000	28.561444000	6.850950000
1	-10.645622000	27.460158000	6.861066000
8	-9.496964000	29.173360000	7.129947000
14	-8.074872000	28.913007000	8.116297000
6	-11.716810000	28.349150000	8.775882000
1	-10.777841000	28.213157000	9.317995000
6	-12.202475000	29.646700000	8.724854000
1	-11.550544000	30.507746000	8.936911000
8	-13.419338000	29.883809000	8.289698000
14	-14.381080000	31.355626000	8.504312000
6	-10.568790000	34.638625000	6.925359000
6	-12.321330000	35.101050000	0.849052000
6	-8.457045000	29.573258000	9.840194000

6	-6.801511000	29.982901000	7.269914000
6	-7.592451000	27.066057000	8.139007000
6	-12.619030000	27.157486000	8.603244000
6	-15.527284000	31.298950000	7.025349000
6	-13.301568000	32.870192000	8.614473000
6	-15.257760000	30.958735000	10.151871000
6	-5.473779000	33.822662000	4.740976000
6	-6.663478000	31.063720000	3.910497000
6	-9.804612000	37.757217000	2.769657000
6	-8.021779000	35.908003000	1.090482000
6	-11.549409000	29.247860000	5.946071000
9	-11.048762000	35.099976000	8.149286000
9	-11.606859000	34.017646000	6.216476000
9	-12.748940000	34.382827000	-0.264200000
1	-10.104662000	35.445555000	6.337386000
9	-13.396298000	35.828008000	1.385890000
1	-11.475946000	35.766103000	0.615482000
1	-7.497680000	29.720747000	10.368466000
1	-9.085133000	28.920566000	10.468519000
1	-6.777605000	29.773968000	6.190723000
1	-7.037631000	31.051875000	7.398701000
6	-8.628314000	26.196215000	8.893372000
1	-8.940146000	30.561396000	9.749989000
1	-5.789813000	29.801892000	7.671245000
6	-7.410619000	26.521736000	6.702237000
6	-6.228418000	26.973523000	8.880530000
1	-12.043642000	26.245715000	8.366296000
1	-13.188221000	26.957493000	9.530476000
1	-13.354288000	27.335903000	7.802501000
1	-16.170498000	30.403345000	7.047538000

1	-16.170521000	32.195232000	6.999728000
1	-14.930690000	31.269627000	6.098150000
1	-13.938238000	33.738605000	8.861059000
1	-12.802351000	33.094937000	7.664000000
6	-16.292571000	32.074060000	10.447326000
6	-15.977339000	29.590405000	10.068469000
6	-14.212165000	30.919146000	11.294237000
1	-12.522572000	32.798706000	9.390677000
6	-4.832908000	32.773746000	4.087504000
6	-3.764388000	33.068280000	3.163552000
6	-3.140009000	32.074134000	2.349204000
1	-3.461081000	31.036859000	2.450711000
6	-2.154677000	32.419601000	1.436098000
1	-1.700205000	31.646663000	0.811816000
6	-1.734190000	33.771959000	1.299190000
1	-0.949002000	34.025374000	0.582682000
6	-2.327765000	34.761896000	2.066057000
1	-2.025782000	35.808530000	1.964472000
6	-3.367635000	34.446174000	2.995027000
6	-4.056714000	35.470987000	3.699441000
1	-3.755570000	36.509430000	3.539079000
6	-5.138960000	35.196972000	4.534403000
6	-5.335367000	31.373775000	4.227859000
6	-4.433033000	30.312542000	4.584407000
6	-3.139214000	30.562161000	5.131027000
1	-2.831422000	31.598035000	5.287095000
6	-2.301660000	29.509774000	5.471644000
1	-1.319271000	29.714490000	5.903909000
6	-2.713596000	28.162004000	5.269156000
1	-2.039773000	27.344783000	5.538126000

6	-3.965457000	27.888224000	4.740465000
1	-4.296768000	26.856030000	4.590355000
6	-4.860243000	28.949083000	4.398177000
6	-6.165823000	28.694919000	3.898028000
1	-6.435361000	27.661875000	3.665497000
6	-7.111818000	29.708844000	3.714065000
6	-5.943013000	36.319645000	5.090715000
6	-6.498307000	36.328805000	6.400071000
1	-6.415105000	35.450724000	7.042392000
6	-7.188387000	37.467439000	6.829568000
6	-7.775950000	37.786983000	8.199792000
6	-6.762298000	37.794720000	9.430583000
1	-5.940051000	37.087198000	9.235564000
1	-6.374842000	38.784485000	9.713063000
6	-7.883287000	37.149061000	10.326993000
1	-7.572062000	36.313050000	10.971527000
1	-8.415218000	37.907965000	10.923894000
6	-8.660772000	36.760786000	9.015865000
1	-9.744714000	36.933732000	8.981118000
1	-8.432436000	35.729110000	8.711133000
6	-8.423043000	39.148269000	7.980212000
6	-9.216950000	39.915510000	8.838384000
1	-9.454540000	39.561133000	9.845003000
6	-9.718872000	41.153559000	8.386122000
1	-10.349025000	41.754569000	9.046670000
6	-9.424976000	41.619732000	7.090586000
1	-9.829483000	42.578563000	6.756775000
6	-8.629530000	40.855542000	6.219108000
1	-8.418432000	41.204947000	5.204492000
6	-8.136087000	39.619812000	6.666501000

6	-7.360329000	38.586671000	5.962433000
6	-6.850704000	38.562376000	4.659232000
1	-7.012212000	39.393810000	3.968739000
6	-6.146307000	37.432050000	4.235992000
1	-5.799442000	37.378769000	3.203985000
6	-8.497494000	29.364443000	3.294082000
6	-8.980687000	28.050768000	3.562018000
1	-8.381838000	27.358100000	4.157158000
6	-10.218383000	27.653730000	3.077731000
6	-10.896136000	26.298974000	3.177381000
6	-10.955508000	25.520639000	4.554145000
1	-10.145284000	25.866417000	5.218007000
1	-11.924666000	25.544629000	5.075282000
6	-10.559687000	24.190260000	3.810983000
1	-11.444680000	23.575690000	3.580063000
1	-9.785356000	23.570441000	4.288152000
6	-10.112054000	25.052221000	2.571710000
1	-10.446897000	24.732070000	1.574515000
1	-9.028308000	25.254281000	2.576293000
6	-12.239252000	26.547035000	2.497164000
6	-13.337186000	25.698369000	2.338322000
1	-13.325175000	24.682209000	2.743241000
6	-14.472730000	26.170453000	1.645257000
1	-15.339366000	25.516345000	1.518613000
6	-14.498544000	27.473590000	1.111493000
1	-15.384971000	27.818723000	0.572784000
6	-13.397248000	28.334852000	1.268981000
1	-13.413946000	29.351707000	0.867595000
6	-12.275565000	27.870808000	1.970283000
6	-11.027182000	28.556239000	2.333820000

6	-10.588670000	29.860072000	2.096505000
1	-11.190972000	30.592748000	1.559404000
6	-9.328894000	30.254993000	2.566709000
1	-8.995651000	31.261682000	2.338078000
6	-9.140019000	38.065840000	1.581741000
6	-9.697759000	39.069454000	0.713144000
6	-9.131960000	39.379752000	-0.561387000
1	-8.241744000	38.836660000	-0.884051000
6	-9.702083000	40.349364000	-1.372597000
1	-9.256997000	40.573755000	-2.345089000
6	-10.868248000	41.049481000	-0.955236000
1	-11.303176000	41.812518000	-1.605309000
6	-11.457499000	40.753120000	0.263736000
1	-12.364865000	41.272137000	0.586389000
6	-10.902960000	39.756586000	1.123191000
6	-11.527668000	39.407130000	2.357773000
1	-12.445348000	39.919371000	2.659153000
6	-11.002109000	38.415734000	3.172186000
6	-7.920245000	37.292296000	1.203449000
6	-6.642031000	37.912147000	0.981767000
6	-6.423853000	39.307659000	1.188761000
1	-7.278025000	39.933423000	1.456407000
6	-5.153419000	39.851657000	1.064502000
1	-4.999157000	40.919060000	1.239667000
6	-4.045508000	39.028746000	0.717227000
1	-3.051606000	39.473016000	0.622840000
6	-4.226534000	37.671038000	0.501207000
1	-3.383372000	37.027244000	0.234358000
6	-5.516770000	37.072170000	0.635139000
6	-5.709445000	35.673077000	0.439120000

1	-4.861628000	35.056671000	0.127934000
6	-6.948219000	35.076278000	0.646895000
6	-11.665123000	37.969139000	4.430908000
6	-12.855771000	37.213696000	4.347454000
1	-13.227386000	36.918526000	3.365244000
6	-13.470085000	36.794359000	5.528560000
6	-14.714257000	35.944862000	5.723459000
6	-14.522998000	34.492626000	5.088711000
1	-13.486221000	34.250479000	4.842842000
1	-14.973271000	33.726384000	5.737464000
6	-15.449822000	34.911397000	3.891872000
1	-14.855737000	35.144578000	2.998811000
1	-16.248664000	34.199776000	3.631845000
6	-15.899516000	36.184320000	4.708867000
1	-16.873393000	36.039586000	5.206670000
6	-14.908008000	35.974788000	7.225230000
6	-15.945569000	35.449807000	7.998526000
1	-16.816621000	34.987948000	7.523111000
6	-15.861867000	35.540747000	9.404573000
1	-16.683046000	35.167143000	10.021682000
6	-14.727336000	36.106084000	10.020916000
1	-14.669769000	36.145246000	11.111976000
6	-13.674665000	36.629057000	9.246879000
1	-12.792121000	37.063250000	9.724009000
6	-13.782702000	36.589223000	7.849398000
6	-12.898558000	37.115285000	6.792652000
6	-11.698158000	37.837020000	6.871040000
1	-11.247835000	38.099548000	7.830652000
6	-11.080907000	38.258739000	5.682914000
1	-10.147587000	38.817557000	5.736315000

6	-7.186668000	33.631992000	0.392566000
6	-8.317284000	33.223459000	-0.364021000
1	-9.026063000	33.970257000	-0.723922000
6	-8.521657000	31.869810000	-0.612768000
6	-9.600527000	31.173733000	-1.436002000
6	-9.471115000	31.533712000	-2.989931000
1	-8.501369000	31.954510000	-3.293489000
1	-9.726806000	30.650791000	-3.598507000
6	-10.690120000	32.515419000	-2.836130000
1	-10.356091000	33.555941000	-2.688712000
1	-11.457552000	32.475921000	-3.624141000
6	-11.037901000	31.828609000	-1.462460000
1	-11.804624000	31.043644000	-1.585128000
1	-11.274885000	32.476613000	-0.612552000
6	-9.395304000	29.714328000	-1.075033000
6	-10.196773000	28.609712000	-1.367286000
1	-11.130651000	28.728522000	-1.924477000
6	-9.806971000	27.340717000	-0.892699000
1	-10.445197000	26.473159000	-1.079099000
6	-8.620920000	27.186900000	-0.150280000
1	-8.346126000	26.198069000	0.226139000
6	-7.804504000	28.294280000	0.141219000
1	-6.906147000	28.180440000	0.753684000
6	-8.197189000	29.558117000	-0.320244000
6	-7.634612000	30.896521000	-0.062202000
6	-6.502936000	31.296304000	0.662743000
1	-5.824375000	30.557531000	1.096486000
6	-6.278426000	32.665711000	0.876389000
1	-5.426743000	32.986879000	1.475876000
1	-15.873215000	37.156447000	4.192934000

6	-11.286449000	30.597327000	5.632183000
6	-12.144060000	31.296550000	4.784421000
6	-13.263848000	30.656300000	4.221727000
6	-13.530096000	29.307820000	4.529955000
6	-12.681726000	28.603314000	5.395343000
1	-10.387261000	31.079392000	6.010766000
1	-11.925543000	32.335982000	4.550741000
1	-13.879268000	31.218497000	3.516093000
1	-14.370564000	28.792565000	4.060451000
1	-12.871256000	27.547226000	5.602552000
1	-8.267682000	25.149776000	8.942631000
1	-8.779583000	26.553734000	9.928695000
1	-9.606981000	26.188907000	8.377839000
1	-6.702714000	27.146842000	6.128272000
1	-7.017333000	25.486496000	6.742724000
1	-8.372816000	26.499855000	6.159596000
1	-5.921630000	25.910720000	8.942886000
1	-5.440877000	27.525951000	8.336159000
1	-6.298396000	27.369840000	9.911000000
1	-17.063408000	32.123817000	9.655002000
1	-16.799897000	31.863742000	11.409748000
1	-15.802602000	33.061508000	10.518541000
1	-15.258503000	28.781234000	9.846761000
1	-16.471352000	29.371077000	11.035525000
1	-16.751670000	29.594892000	9.279160000
1	-13.689235000	31.888178000	11.392381000
1	-14.719049000	30.700997000	12.255035000
1	-13.458955000	30.126142000	11.122188000

TS-B4:

B3LYP-D3(BJ) Energy: -8320.21371198 hartree; Imaginary frequency = -61.25643207

16	-6.418556000	31.036718000	6.090572000
16	-12.793320000	33.370321000	4.767076000
15	-7.445300000	33.263327000	4.580538000
15	-10.178437000	34.641704000	4.576238000
7	-8.676342000	34.230128000	4.533779000
7	-7.632262000	31.927749000	5.441917000
7	-11.203820000	33.470959000	4.966627000
8	-6.095966000	34.054388000	5.191173000
8	-7.001742000	33.113034000	2.970015000
8	-5.145253000	31.008823000	5.322948000
8	-7.006877000	29.737536000	6.532350000
8	-10.218965000	35.941274000	5.638434000
8	-10.636631000	35.391365000	3.130097000
8	-13.340628000	32.327834000	5.685766000
8	-13.561392000	34.635280000	4.617484000
6	-13.298744000	28.170700000	6.614376000
1	-13.025745000	27.217181000	7.099212000
8	-12.484473000	29.179624000	6.710466000
14	-10.707871000	29.166221000	6.956246000
6	-12.833864000	27.059009000	4.761065000
1	-11.759715000	27.053991000	4.972200000
6	-13.252128000	28.070688000	3.916150000
1	-12.633708000	28.961724000	3.750140000
8	-14.456489000	28.040824000	3.374000000
14	-15.173381000	29.038771000	2.120305000
6	-6.131902000	32.007418000	7.709682000
6	-12.859938000	32.553840000	3.068454000
6	-10.146621000	29.599888000	5.220025000

6	-10.308203000	27.432693000	7.597693000
6	-10.489185000	30.445767000	8.341061000
6	-13.580211000	25.760545000	4.878146000
6	-13.846423000	29.438965000	0.851954000
6	-15.912363000	30.581484000	2.887918000
6	-16.485183000	27.815910000	1.506456000
6	-5.674685000	35.197618000	4.520948000
6	-5.684384000	32.755407000	2.688943000
6	-11.288908000	36.827699000	5.517550000
6	-9.903967000	36.540436000	2.806554000
6	-14.736173000	28.478958000	6.509377000
9	-4.946457000	32.723918000	7.562673000
9	-6.007895000	31.077981000	8.733921000
9	-11.762269000	32.997413000	2.333868000
1	-6.993985000	32.674969000	7.869346000
9	-12.743482000	31.151389000	3.263125000
1	-13.808528000	32.788930000	2.563062000
1	-9.132537000	30.027475000	5.229244000
1	-10.827162000	30.358782000	4.793506000
1	-10.541914000	26.611387000	6.898773000
1	-9.226264000	27.386767000	7.809365000
6	-11.645679000	30.269559000	9.360519000
1	-10.129308000	28.726590000	4.546103000
1	-10.837198000	27.248393000	8.549952000
6	-9.145510000	30.212963000	9.083570000
6	-10.529531000	31.871960000	7.739646000
1	-13.278822000	25.092298000	4.052564000
1	-13.345003000	25.238866000	5.822390000
1	-14.668515000	25.915911000	4.814701000
1	-14.266612000	30.051275000	0.034485000

1	-13.038432000	30.019344000	1.323716000
1	-13.414434000	28.527387000	0.408635000
1	-16.736201000	30.326488000	3.571309000
1	-16.292429000	31.258788000	2.102670000
6	-17.320502000	28.465753000	0.374812000
6	-15.786430000	26.537865000	0.975265000
6	-17.410282000	27.435429000	2.692059000
1	-15.146076000	31.106076000	3.476737000
6	-4.990364000	35.061727000	3.313690000
6	-4.633521000	36.243513000	2.576894000
6	-4.047835000	36.187448000	1.274823000
1	-3.850677000	35.207319000	0.834991000
6	-3.733119000	37.351932000	0.589357000
1	-3.293090000	37.293234000	-0.409137000
6	-3.966852000	38.625000000	1.182258000
1	-3.686595000	39.532961000	0.641926000
6	-4.548490000	38.711116000	2.438262000
1	-4.750154000	39.682072000	2.898159000
6	-4.928771000	37.533974000	3.152683000
6	-5.588106000	37.611451000	4.412240000
1	-5.787293000	38.597028000	4.841306000
6	-5.985358000	36.468784000	5.101711000
6	-4.661706000	33.692244000	2.827494000
6	-3.302777000	33.286501000	2.573220000
6	-2.187277000	34.149351000	2.797264000
1	-2.370672000	35.151132000	3.188847000
6	-0.894636000	33.722033000	2.531850000
1	-0.053061000	34.395150000	2.713368000
6	-0.651778000	32.410150000	2.038753000
1	0.372405000	32.089803000	1.832318000

6	-1.709555000	31.536321000	1.843576000
1	-1.533485000	30.515564000	1.491440000
6	-3.052290000	31.938414000	2.116126000
6	-4.141097000	31.029308000	1.982006000
1	-3.945917000	30.003978000	1.655932000
6	-5.439567000	31.411097000	2.283745000
6	-6.658956000	36.563483000	6.424310000
6	-7.593037000	37.604676000	6.666245000
1	-7.905147000	38.254731000	5.842748000
6	-8.119637000	37.768578000	7.947702000
6	-9.049921000	38.848481000	8.476825000
6	-10.395382000	39.217145000	7.723907000
1	-10.345032000	38.901152000	6.672641000
1	-11.311143000	38.827161000	8.188878000
6	-10.070257000	40.745400000	7.904482000
1	-10.200692000	41.379086000	7.013065000
1	-10.609900000	41.185739000	8.758476000
6	-8.589960000	40.359761000	8.274880000
1	-8.137106000	40.833631000	9.157872000
1	-7.922100000	40.430192000	7.400216000
6	-9.242690000	38.440776000	9.931758000
6	-10.057877000	39.001824000	10.917875000
1	-10.672923000	39.879188000	10.700599000
6	-10.091986000	38.411054000	12.197431000
1	-10.735112000	38.836327000	12.972011000
6	-9.313410000	37.274267000	12.486880000
1	-9.355654000	36.830416000	13.484824000
6	-8.487696000	36.703450000	11.501890000
1	-7.882102000	35.819877000	11.724624000
6	-8.460881000	37.286256000	10.225109000

6	-7.752686000	36.880813000	8.999227000
6	-6.861620000	35.826206000	8.756876000
1	-6.575615000	35.139481000	9.559037000
6	-6.311393000	35.674507000	7.476817000
1	-5.617834000	34.855190000	7.293366000
6	-6.570777000	30.448829000	2.232616000
6	-6.502946000	29.259017000	2.995800000
1	-5.658398000	29.115680000	3.674933000
6	-7.554655000	28.345386000	2.910922000
6	-7.717051000	27.005206000	3.601633000
6	-7.593767000	26.934742000	5.185453000
1	-7.111345000	27.836362000	5.593788000
1	-8.556363000	26.765822000	5.686321000
6	-6.651441000	25.681241000	5.052141000
1	-7.179451000	24.734671000	5.256580000
1	-5.715525000	25.719209000	5.629658000
6	-6.519441000	25.966239000	3.508584000
1	-6.688438000	25.125685000	2.818898000
1	-5.583227000	26.491692000	3.260250000
6	-9.047649000	26.506287000	3.057752000
6	-9.721539000	25.305611000	3.300187000
1	-9.294185000	24.555564000	3.972221000
6	-10.946322000	25.059237000	2.642460000
1	-11.464707000	24.109754000	2.803348000
6	-11.501357000	26.019239000	1.773833000
1	-12.455966000	25.815769000	1.279210000
6	-10.833936000	27.234978000	1.532555000
1	-11.251242000	27.975848000	0.845604000
6	-9.605023000	27.471894000	2.167143000
6	-8.676211000	28.612032000	2.076174000

6	-8.753106000	29.803694000	1.342550000
1	-9.607160000	30.015491000	0.696709000
6	-7.701217000	30.723922000	1.429528000
1	-7.746146000	31.654781000	0.867014000
6	-11.300203000	37.739191000	4.466936000
6	-12.404948000	38.647013000	4.322172000
6	-12.513690000	39.559392000	3.229607000
1	-11.729542000	39.561067000	2.470464000
6	-13.593229000	40.425253000	3.136704000
1	-13.663080000	41.117954000	2.294326000
6	-14.616846000	40.415635000	4.125390000
1	-15.458437000	41.107465000	4.039236000
6	-14.552259000	39.524373000	5.184772000
1	-15.341654000	39.497341000	5.941744000
6	-13.459472000	38.613162000	5.308679000
6	-13.385708000	37.674269000	6.375877000
1	-14.173523000	37.662991000	7.134322000
6	-12.330454000	36.776642000	6.486992000
6	-10.171185000	37.720537000	3.501747000
6	-9.346596000	38.876952000	3.294418000
6	-9.471169000	40.062325000	4.082651000
1	-10.243687000	40.100545000	4.852378000
6	-8.630018000	41.144786000	3.869192000
1	-8.728737000	42.038958000	4.490168000
6	-7.647859000	41.104235000	2.838712000
1	-7.009714000	41.974679000	2.665489000
6	-7.503289000	39.966788000	2.059017000
1	-6.744116000	39.914497000	1.274949000
6	-8.318623000	38.815775000	2.286471000
6	-8.137371000	37.614772000	1.548398000

1	-7.374996000	37.593116000	0.765398000
6	-8.885629000	36.464053000	1.803162000
6	-12.263165000	35.832417000	7.627630000
6	-13.392833000	35.034897000	7.926460000
1	-14.230044000	35.021308000	7.222830000
6	-13.372816000	34.242555000	9.071699000
6	-14.425486000	33.277782000	9.577110000
6	-14.947416000	32.179144000	8.555177000
1	-14.688725000	32.467437000	7.525772000
1	-14.587714000	31.161612000	8.760701000
6	-16.430549000	32.501541000	8.968843000
1	-17.140269000	32.670776000	8.143881000
1	-16.840308000	31.750224000	9.663802000
6	-15.921297000	33.791358000	9.718175000
1	-16.261443000	33.940393000	10.753832000
6	-13.792144000	32.725208000	10.844215000
6	-14.271261000	31.760393000	11.732356000
1	-15.253848000	31.303743000	11.581242000
6	-13.463473000	31.370729000	12.821160000
1	-13.826210000	30.613216000	13.520858000
6	-12.191724000	31.943609000	13.013232000
1	-11.578382000	31.624876000	13.860097000
6	-11.700681000	32.915765000	12.122283000
1	-10.711058000	33.358618000	12.266153000
6	-12.504615000	33.305502000	11.040936000
6	-12.241343000	34.243818000	9.934585000
6	-11.110317000	35.014693000	9.625722000
1	-10.238788000	35.040382000	10.283705000
6	-11.119203000	35.793884000	8.459909000
1	-10.238052000	36.376607000	8.191664000

6	-8.636176000	35.246284000	0.986119000
6	-9.694699000	34.449670000	0.474033000
1	-10.716861000	34.625929000	0.811276000
6	-9.400272000	33.446663000	-0.451419000
6	-10.349436000	32.546072000	-1.231797000
6	-11.519485000	33.250875000	-2.046186000
1	-11.735137000	34.243422000	-1.618836000
1	-11.350413000	33.320341000	-3.130562000
6	-12.526501000	32.151596000	-1.537016000
1	-13.481487000	32.521996000	-1.131266000
1	-12.717177000	31.379504000	-2.300320000
6	-11.454378000	31.688518000	-0.481912000
1	-11.240743000	30.609605000	-0.448733000
1	-11.630504000	32.085179000	0.523569000
6	-9.401442000	31.707904000	-2.077226000
6	-9.685057000	30.667437000	-2.964939000
1	-10.718055000	30.363820000	-3.158469000
6	-8.617816000	30.006008000	-3.606485000
1	-8.826677000	29.188215000	-4.300940000
6	-7.284932000	30.382516000	-3.354514000
1	-6.470446000	29.851090000	-3.853179000
6	-6.991375000	31.428711000	-2.461975000
1	-5.955773000	31.712135000	-2.256361000
6	-8.052960000	32.091353000	-1.828743000
6	-8.054769000	33.191044000	-0.846903000
6	-7.000145000	33.930223000	-0.291651000
1	-5.959838000	33.709882000	-0.545560000
6	-7.300992000	34.955389000	0.612561000
1	-6.492324000	35.529245000	1.066519000
1	-16.068705000	34.707973000	9.124164000

6	-15.127454000	29.800751000	6.204337000
6	-16.490734000	30.124508000	6.181321000
6	-17.463004000	29.136406000	6.423469000
6	-17.070282000	27.815952000	6.711864000
6	-15.708565000	27.488335000	6.770003000
1	-14.362388000	30.561447000	6.012885000
1	-16.790607000	31.156651000	5.983730000
1	-18.524616000	29.396179000	6.404103000
1	-17.824814000	27.052232000	6.914621000
1	-15.395769000	26.476048000	7.040387000
1	-11.530785000	31.004667000	10.177073000
1	-12.622393000	30.449372000	8.882614000
1	-11.643899000	29.255105000	9.806893000
1	-8.271321000	30.227540000	8.412865000
1	-9.027498000	31.015008000	9.839688000
1	-9.150021000	29.245200000	9.621247000
1	-10.435871000	32.618281000	8.551047000
1	-9.712535000	32.021273000	7.011563000
1	-11.480819000	32.068154000	7.216680000
1	-16.684393000	28.739776000	-0.488308000
1	-18.086331000	27.748920000	0.019561000
1	-17.843097000	29.372937000	0.732533000
1	-15.169222000	26.076246000	1.767690000
1	-16.549818000	25.804089000	0.651135000
1	-15.139355000	26.765587000	0.107151000
1	-17.952695000	28.317656000	3.079355000
1	-18.157483000	26.691555000	2.352849000
1	-16.824909000	27.001759000	3.522097000

Acetaldehyde enolsilane addition (with Catalyst 7a)

Major enantiomer

B3LYP-D3(BJ) Energy: -8479.40245903518hartree;

16	-12.767940000	-11.232250000	-5.598030000
16	-7.831340000	-8.312030000	-8.008440000
8	-12.289510000	-8.634750000	-3.596740000
8	-10.015100000	-9.837050000	-3.803370000
8	-12.190650000	-12.153220000	-4.583140000
8	-13.157520000	-11.789020000	-6.926260000
8	-7.894800000	-6.826880000	-5.144840000
8	-9.916610000	-5.384360000	-5.948950000
8	-7.711940000	-9.448480000	-7.044520000
8	-8.008780000	-8.634200000	-9.453760000
6	-13.436180000	-10.985500000	-9.862090000
1	-13.044340000	-11.276460000	-8.877530000
8	-12.587550000	-10.556350000	-10.776710000
14	-10.903500000	-10.039590000	-10.442720000
15	-11.145010000	-9.036770000	-4.740330000
15	-9.314790000	-6.924950000	-6.026860000
7	-10.415630000	-7.796000000	-5.333950000
7	-11.944470000	-9.877580000	-5.868620000
7	-8.875890000	-7.173170000	-7.562790000
6	-13.402910000	-13.615670000	-9.728920000
1	-12.338320000	-13.809120000	-9.945590000
6	-14.148140000	-12.814400000	-10.598450000
1	-15.193263000	-12.664654000	-10.424689000
8	-13.900670000	-14.078340000	-8.612310000
14	-13.004230000	-14.972690000	-7.348700000
6	-14.459060000	-10.733390000	-4.853460000

6	-6.153480000	-7.436860000	-8.030610000
6	-11.195270000	-8.245050000	-9.996440000
6	-10.114070000	-10.343160000	-12.129730000
6	-10.224980000	-11.162910000	-9.078130000
6	-13.076020000	-16.754930000	-7.988840000
6	-14.083280000	-14.769620000	-5.818590000
6	-11.227200000	-14.364980000	-7.261460000
6	-11.814760000	-7.951190000	-2.468350000
6	-10.366630000	-10.687860000	-2.754830000
6	-7.891920000	-6.177420000	-3.912560000
6	-8.963950000	-4.380130000	-6.173500000
6	-14.769510000	-10.330820000	-9.830690000
9	-15.432650000	-11.482240000	-5.502400000
9	-14.738610000	-9.394350000	-5.050620000
9	-14.470990000	-11.017680000	-3.522490000
9	-5.756830000	-6.995870000	-6.784400000
9	-5.224580000	-8.347290000	-8.490510000
9	-6.234460000	-6.367310000	-8.893100000
1	-11.625150000	-7.735260000	-10.875010000
1	-10.231170000	-7.756480000	-9.790570000
1	-9.898800000	-11.422060000	-12.244020000
1	-9.135010000	-9.829480000	-12.137170000
1	-10.140400000	-10.594220000	-8.135760000
1	-10.954590000	-11.967280000	-8.876200000
6	-12.129510000	-8.061540000	-8.777090000
6	-8.849470000	-11.794890000	-9.425520000
6	-10.996240000	-9.841320000	-13.301890000
1	-12.485360000	-16.823300000	-8.921560000
1	-12.554350000	-17.398240000	-7.253060000
6	-14.515220000	-17.275790000	-8.236050000

1	-14.480070000	-13.742610000	-5.752610000
1	-14.954860000	-15.440820000	-5.933430000
6	-13.294810000	-15.125850000	-4.523520000
1	-11.205220000	-13.264230000	-7.251770000
1	-10.828660000	-14.648390000	-6.270670000
6	-10.278690000	-14.919010000	-8.357910000
6	-11.958750000	-6.527150000	-2.414380000
6	-11.531640000	-5.890030000	-1.250030000
1	-11.665680000	-4.810110000	-1.148630000
6	-10.906200000	-6.598800000	-0.187540000
6	-10.466030000	-5.923430000	0.991680000
1	-10.654870000	-4.850310000	1.074560000
6	-9.779180000	-6.603430000	1.985300000
1	-9.442790000	-6.075870000	2.881720000
6	-9.496750000	-7.989680000	1.834900000
1	-8.932070000	-8.514380000	2.609400000
6	-9.947130000	-8.685530000	0.721710000
1	-9.756860000	-9.755200000	0.622820000
6	-10.684170000	-8.019660000	-0.305510000
6	-11.194770000	-8.696800000	-1.471580000
6	-9.958160000	-12.065890000	-2.836370000
6	-10.228880000	-12.869980000	-1.727640000
1	-9.849120000	-13.893700000	-1.702010000
6	-11.004500000	-12.425670000	-0.624250000
6	-11.366480000	-13.303590000	0.443560000
1	-11.012670000	-14.338450000	0.412000000
6	-12.168230000	-12.859880000	1.483370000
1	-12.448280000	-13.540490000	2.291150000
6	-12.646170000	-11.519310000	1.494260000
1	-13.300590000	-11.185890000	2.303280000

6	-12.292740000	-10.636150000	0.484300000
1	-12.655290000	-9.606670000	0.490040000
6	-11.449300000	-11.057720000	-0.586540000
6	-11.020180000	-10.167880000	-1.633710000
6	-12.517330000	-5.745300000	-3.549310000
6	-11.983820000	-4.457420000	-3.815130000
1	-11.130900000	-4.105930000	-3.228490000
6	-12.485440000	-3.655530000	-4.850800000
1	-12.047020000	-2.671760000	-5.041760000
6	-13.516950000	-4.158530000	-5.658960000
6	-14.193800000	-3.604790000	-6.849670000
6	-14.006940000	-2.398600000	-7.542390000
1	-13.292670000	-1.652800000	-7.183680000
6	-14.729690000	-2.180320000	-8.729290000
1	-14.581620000	-1.254080000	-9.289710000
6	-15.623050000	-3.152970000	-9.218270000
1	-16.161740000	-2.970870000	-10.151790000
6	-15.820340000	-4.362820000	-8.519190000
1	-16.508150000	-5.123010000	-8.902600000
6	-15.112200000	-4.582080000	-7.334910000
6	-15.119010000	-5.796450000	-6.425650000
6	-14.031000000	-5.464890000	-5.422240000
6	-13.558500000	-6.249090000	-4.374620000
1	-13.957480000	-7.250980000	-4.217180000
6	-15.104680000	-7.208540000	-7.150400000
1	-14.983070000	-7.142760000	-8.241340000
1	-14.355440000	-7.890060000	-6.726060000
6	-16.567330000	-7.494320000	-6.643390000
1	-17.314390000	-7.485740000	-7.450380000
1	-16.651130000	-8.416540000	-6.051120000

6	-16.539040000	-6.165980000	-5.800620000
1	-16.463180000	-6.330930000	-4.714530000
1	-17.324640000	-5.429430000	-6.026490000
6	-9.245870000	-12.648260000	-4.007500000
6	-8.494730000	-11.870710000	-4.927210000
1	-8.451290000	-10.790920000	-4.831120000
6	-7.760320000	-12.468490000	-5.961350000
1	-7.217390000	-11.824590000	-6.653340000
6	-7.736220000	-13.862430000	-6.068840000
6	-7.040120000	-14.757330000	-7.007050000
6	-6.180410000	-14.465620000	-8.076020000
1	-5.920020000	-13.429390000	-8.309090000
6	-5.663950000	-15.531270000	-8.835510000
1	-4.993820000	-15.325000000	-9.674090000
6	-6.002400000	-16.862310000	-8.526640000
1	-5.593730000	-17.677970000	-9.128800000
6	-6.863070000	-17.155980000	-7.447150000
1	-7.117510000	-18.194020000	-7.214810000
6	-7.381510000	-16.105050000	-6.688000000
6	-8.309500000	-16.133720000	-5.477370000
6	-8.500490000	-14.658380000	-5.171830000
6	-9.265080000	-14.063560000	-4.176360000
1	-9.897790000	-14.683940000	-3.535670000
6	-7.785450000	-17.069770000	-4.302800000
1	-8.042340000	-16.626770000	-3.326790000
1	-6.714860000	-17.314080000	-4.350770000
6	-8.812770000	-18.177410000	-4.744790000
1	-9.392490000	-18.660800000	-3.943890000
1	-8.338100000	-18.943140000	-5.379550000
6	-9.566320000	-17.095300000	-5.604890000

1	-9.847100000	-17.366770000	-6.633300000
1	-10.430660000	-16.672110000	-5.064790000
6	-7.592440000	-6.949190000	-2.746910000
6	-7.637470000	-6.299610000	-1.513310000
1	-7.390300000	-6.855670000	-0.605310000
6	-7.995980000	-4.926510000	-1.399300000
6	-8.074540000	-4.271400000	-0.132430000
1	-7.854220000	-4.854120000	0.764700000
6	-8.450200000	-2.939290000	-0.042780000
1	-8.512730000	-2.451240000	0.933360000
6	-8.756420000	-2.198690000	-1.219940000
1	-9.067720000	-1.154550000	-1.135950000
6	-8.648150000	-2.790610000	-2.470150000
1	-8.856350000	-2.226680000	-3.382550000
6	-8.253520000	-4.157550000	-2.592450000
6	-8.124970000	-4.802380000	-3.866360000
6	-8.882700000	-3.801820000	-7.473050000
6	-7.918660000	-2.829000000	-7.705370000
1	-7.856320000	-2.366940000	-8.694640000
6	-7.006360000	-2.426490000	-6.687900000
6	-5.999070000	-1.445230000	-6.937690000
1	-5.962900000	-0.976110000	-7.925360000
6	-5.080620000	-1.104600000	-5.957600000
1	-4.311970000	-0.354800000	-6.160360000
6	-5.124550000	-1.743950000	-4.687800000
1	-4.381220000	-1.488530000	-3.928630000
6	-6.095240000	-2.694210000	-4.407120000
1	-6.123640000	-3.188180000	-3.435270000
6	-7.076310000	-3.045160000	-5.383250000
6	-8.098170000	-4.034310000	-5.143190000

6	-7.164230000	-8.369750000	-2.852180000
6	-7.585150000	-9.285190000	-1.859840000
1	-8.288130000	-8.942980000	-1.098200000
6	-7.164600000	-10.622060000	-1.878710000
1	-7.550610000	-11.329720000	-1.140860000
6	-6.278050000	-11.038560000	-2.881760000
6	-5.720510000	-12.367390000	-3.183090000
6	-5.956120000	-13.617310000	-2.594310000
1	-6.628180000	-13.711250000	-1.736940000
6	-5.337540000	-14.751740000	-3.150240000
1	-5.517790000	-15.736330000	-2.711230000
6	-4.506500000	-14.637680000	-4.280760000
1	-4.061280000	-15.534610000	-4.718710000
6	-4.265480000	-13.380040000	-4.870230000
1	-3.635690000	-13.300420000	-5.761030000
6	-4.871800000	-12.249160000	-4.320880000
6	-4.799820000	-10.804280000	-4.778620000
6	-5.815160000	-10.116550000	-3.867770000
6	-6.278080000	-8.801660000	-3.879560000
1	-5.992380000	-8.117180000	-4.677670000
6	-4.856510000	-10.466830000	-6.322490000
1	-4.580540000	-11.355150000	-6.916760000
1	-5.806490000	-10.043590000	-6.665710000
6	-3.653230000	-9.468230000	-6.150380000
1	-2.842170000	-9.544380000	-6.890660000
1	-4.010300000	-8.430130000	-6.093560000
6	-3.367200000	-10.094610000	-4.735680000
1	-3.218310000	-9.406290000	-3.890820000
1	-2.569820000	-10.854970000	-4.769660000
6	-9.797920000	-4.254980000	-8.547150000

6	-11.193350000	-4.230280000	-8.340620000
1	-11.584190000	-3.944270000	-7.365450000
6	-12.075840000	-4.622270000	-9.356090000
1	-13.153600000	-4.601030000	-9.180350000
6	-11.539650000	-5.048380000	-10.578640000
6	-12.209960000	-5.584010000	-11.774520000
6	-13.567420000	-5.834370000	-12.026090000
1	-14.323980000	-5.591180000	-11.274320000
6	-13.926470000	-6.415560000	-13.257210000
1	-14.979090000	-6.621210000	-13.473160000
6	-12.946410000	-6.728370000	-14.219290000
1	-13.244890000	-7.174580000	-15.171460000
6	-11.580140000	-6.483050000	-13.960680000
1	-10.821560000	-6.742450000	-14.705790000
6	-11.212380000	-5.927650000	-12.733270000
6	-9.827010000	-5.639310000	-12.179280000
6	-10.130680000	-5.097560000	-10.788530000
6	-9.257600000	-4.714120000	-9.771490000
1	-8.175430000	-4.849850000	-9.850890000
6	-8.840450000	-4.720830000	-13.021070000
1	-8.770390000	-3.678220000	-12.677540000
1	-9.100690000	-4.763840000	-14.091920000
6	-7.656910000	-5.696320000	-12.666500000
1	-6.983730000	-5.974240000	-13.491720000
1	-7.068540000	-5.335100000	-11.807060000
6	-8.712450000	-6.775140000	-12.217470000
1	-8.522060000	-7.296910000	-11.268620000
1	-8.933260000	-7.493150000	-13.025180000
6	-16.392940000	-8.828460000	-10.835740000
6	-15.134700000	-9.446980000	-10.866490000

6	-15.642520000	-10.570370000	-8.744580000
6	-16.905850000	-9.967320000	-8.740200000
6	-17.288100000	-9.103960000	-9.786740000
1	-16.673040000	-8.129240000	-11.628270000
1	-14.408980000	-9.222360000	-11.651480000
1	-15.311710000	-11.189140000	-7.905870000
1	-17.587190000	-10.145760000	-7.905020000
1	-18.274410000	-8.632480000	-9.770230000
1	-11.760250000	-8.589530000	-7.881420000
1	-12.196010000	-6.987180000	-8.533480000
1	-13.148380000	-8.426040000	-8.994900000
1	-8.516030000	-12.438620000	-8.594820000
1	-8.904930000	-12.412670000	-10.339800000
1	-8.103160000	-10.998260000	-9.573950000
1	-10.504510000	-10.023110000	-14.275310000
1	-11.972020000	-10.357340000	-13.298570000
1	-11.194000000	-8.760000000	-13.213460000
1	-15.038160000	-16.635710000	-8.969020000
1	-14.502730000	-18.309360000	-8.624930000
1	-15.102780000	-17.270100000	-7.300860000
1	-12.762540000	-16.092530000	-4.617720000
1	-12.567870000	-14.324450000	-4.313180000
1	-13.979960000	-15.203120000	-3.660770000
1	-10.514870000	-14.502190000	-9.354330000
1	-9.237550000	-14.650860000	-8.122000000
1	-10.327340000	-16.019840000	-8.426660000
1	-13.673534000	-12.350727000	-11.437888000

Minor enantiomer

B3LYP-D3(BJ) Energy: -8479.396857245965 hartree;

16	-12.870160000	-10.846970000	-5.839490000

16	-6.934860000	-7.969900000	-7.973900000
8	-11.988150000	-8.421890000	-4.251960000
8	-9.710830000	-9.636200000	-4.076650000
8	-13.211170000	-11.174870000	-4.429010000
8	-13.072950000	-11.893810000	-6.876820000
8	-7.692170000	-6.752670000	-5.113820000
8	-9.698020000	-5.452940000	-6.154180000
8	-6.912890000	-9.105320000	-7.002760000
8	-6.832040000	-8.266670000	-9.429060000
6	-12.472200000	-12.230530000	-10.463790000
1	-11.812760000	-12.462860000	-11.317390000
8	-12.211460000	-11.174540000	-9.746710000
14	-10.843800000	-10.021170000	-9.923220000
15	-10.769010000	-9.034330000	-5.211720000
15	-8.883630000	-6.885620000	-6.285520000
7	-9.953780000	-8.014100000	-6.071920000
7	-11.453910000	-10.156790000	-6.142500000
7	-8.105530000	-6.894640000	-7.703770000
6	-12.196120000	-14.038860000	-8.363170000
1	-11.634640000	-13.558300000	-7.548310000
6	-11.628990000	-14.155870000	-9.625770000
1	-12.134546000	-14.708005000	-10.390270000
8	-13.441400000	-14.401320000	-8.128850000
14	-14.231670000	-14.603340000	-6.549810000
6	-14.139080000	-9.488480000	-6.332140000
6	-5.356120000	-6.986430000	-7.691510000
6	-11.613990000	-8.406900000	-9.339480000
6	-10.438270000	-10.062710000	-11.770020000
6	-9.451170000	-10.678280000	-8.840060000
6	-15.671970000	-13.396290000	-6.314820000

6	-12.889400000	-14.628580000	-5.217250000
6	-14.878650000	-16.364420000	-6.812250000
6	-11.723440000	-7.888160000	-2.992080000
6	-10.277940000	-10.618300000	-3.250300000
6	-7.895740000	-6.130500000	-3.888660000
6	-8.858610000	-4.330600000	-6.211380000
6	-13.869770000	-12.667400000	-10.562940000
9	-14.982510000	-9.974190000	-7.324360000
9	-13.497190000	-8.362690000	-6.807970000
9	-14.893380000	-9.134180000	-5.240050000
9	-5.378660000	-5.896220000	-8.539320000
9	-5.227300000	-6.562550000	-6.383610000
9	-4.285190000	-7.799290000	-7.996130000
1	-11.002540000	-7.577570000	-9.744670000
1	-11.503030000	-8.363990000	-8.244400000
1	-10.136720000	-11.085660000	-12.068680000
1	-9.533030000	-9.442680000	-11.902430000
1	-9.165780000	-9.881080000	-8.133750000
1	-9.864360000	-11.476380000	-8.198420000
6	-13.105230000	-8.174470000	-9.700330000
6	-8.198730000	-11.163910000	-9.616180000
6	-11.567700000	-9.531260000	-12.689590000
1	-15.464470000	-12.436570000	-6.811370000
1	-16.565280000	-13.834470000	-6.797730000
6	-15.944040000	-13.132240000	-4.804260000
1	-12.005100000	-15.105450000	-5.677740000
1	-12.587280000	-13.610340000	-4.927930000
6	-13.285030000	-15.434080000	-3.949920000
1	-15.441390000	-16.683890000	-5.914420000
1	-15.604010000	-16.345850000	-7.646910000

6	-13.738470000	-17.369470000	-7.125910000
6	-11.954510000	-6.484800000	-2.808610000
6	-11.714670000	-5.951440000	-1.545240000
1	-11.899200000	-4.888910000	-1.366390000
6	-11.231940000	-6.753120000	-0.472730000
6	-10.984040000	-6.194570000	0.818240000
1	-11.198120000	-5.134100000	0.972120000
6	-10.454640000	-6.969450000	1.838960000
1	-10.263440000	-6.529620000	2.821190000
6	-10.153550000	-8.341590000	1.610790000
1	-9.718750000	-8.940930000	2.414340000
6	-10.423230000	-8.927530000	0.382310000
1	-10.221620000	-9.987100000	0.213040000
6	-10.983150000	-8.158930000	-0.684040000
6	-11.280780000	-8.727710000	-1.972020000
6	-10.014170000	-12.001400000	-3.528390000
6	-10.593060000	-12.929240000	-2.657070000
1	-10.354980000	-13.990030000	-2.764950000
6	-11.507260000	-12.555970000	-1.635780000
6	-12.159540000	-13.535330000	-0.824090000
1	-11.918780000	-14.590430000	-0.984120000
6	-13.095640000	-13.159100000	0.125320000
1	-13.596340000	-13.916210000	0.733810000
6	-13.422390000	-11.784880000	0.301500000
1	-14.181280000	-11.500310000	1.034280000
6	-12.792600000	-10.807430000	-0.454490000
1	-13.044270000	-9.753380000	-0.326580000
6	-11.808460000	-11.161930000	-1.424140000
6	-11.115620000	-10.189650000	-2.223000000
6	-12.455800000	-5.645870000	-3.930120000

6	-13.435300000	-6.154480000	-4.820210000
1	-13.824270000	-7.157720000	-4.678940000
6	-13.908730000	-5.387090000	-5.890750000
1	-14.631910000	-5.819160000	-6.587340000
6	-13.442900000	-4.075350000	-6.045220000
6	-13.779000000	-3.042360000	-7.039690000
6	-14.609890000	-3.094250000	-8.169810000
1	-15.159200000	-4.007660000	-8.415870000
6	-14.717970000	-1.950170000	-8.981000000
1	-15.355390000	-1.971910000	-9.868590000
6	-14.007840000	-0.776830000	-8.663140000
1	-14.098660000	0.100760000	-9.308120000
6	-13.173470000	-0.724640000	-7.527830000
1	-12.616510000	0.187170000	-7.295380000
6	-13.059510000	-1.856090000	-6.716400000
6	-12.197940000	-2.085170000	-5.482280000
6	-12.481650000	-3.539860000	-5.140220000
6	-11.972760000	-4.322160000	-4.102700000
1	-11.190860000	-3.940400000	-3.439250000
6	-12.394520000	-1.014210000	-4.322370000
1	-13.331780000	-0.440900000	-4.370380000
1	-12.281440000	-1.502990000	-3.340380000
6	-11.068070000	-0.296560000	-4.775320000
1	-11.267530000	0.593720000	-5.393370000
1	-10.353910000	-0.042540000	-3.976030000
6	-10.711460000	-1.556050000	-5.647010000
1	-10.005320000	-2.231630000	-5.147600000
1	-10.392100000	-1.386130000	-6.684140000
6	-9.166310000	-12.460400000	-4.665090000
6	-8.180950000	-11.640460000	-5.274650000

1	-8.032460000	-10.616710000	-4.946920000
6	-7.358800000	-12.129990000	-6.298030000
1	-6.635190000	-11.454720000	-6.754060000
6	-7.497540000	-13.456820000	-6.716620000
6	-6.769280000	-14.230890000	-7.734940000
6	-5.714830000	-13.866070000	-8.585130000
1	-5.287180000	-12.860770000	-8.538890000
6	-5.223090000	-14.820360000	-9.494630000
1	-4.405360000	-14.553610000	-10.169130000
6	-5.772120000	-16.116300000	-9.547290000
1	-5.377280000	-16.841880000	-10.263140000
6	-6.822210000	-16.488230000	-8.681180000
1	-7.237660000	-17.500210000	-8.717970000
6	-7.316630000	-15.545500000	-7.777760000
6	-8.404210000	-15.696680000	-6.728900000
6	-8.494880000	-14.292270000	-6.136960000
6	-9.327500000	-13.795270000	-5.139710000
1	-10.113980000	-14.426550000	-4.723390000
6	-8.202700000	-16.811350000	-5.609330000
1	-7.832320000	-16.429490000	-4.646870000
1	-7.557500000	-17.620550000	-5.988940000
6	-9.731690000	-17.174840000	-5.729430000
1	-10.334100000	-16.668610000	-4.956870000
1	-9.986720000	-18.244930000	-5.761640000
6	-9.774660000	-16.395540000	-7.097540000
1	-9.642190000	-17.073460000	-7.957600000
1	-10.621610000	-15.718190000	-7.275870000
6	-7.671730000	-6.915960000	-2.712310000
6	-7.898910000	-6.315550000	-1.476230000
1	-7.720040000	-6.887360000	-0.561920000

6	-8.358340000	-4.973100000	-1.367490000
6	-8.627860000	-4.377070000	-0.097730000
1	-8.473360000	-4.982090000	0.798670000
6	-9.105210000	-3.078250000	-0.006860000
1	-9.317440000	-2.637120000	0.970490000
6	-9.314870000	-2.311030000	-1.187420000
1	-9.698660000	-1.290340000	-1.111700000
6	-9.016060000	-2.843200000	-2.433490000
1	-9.138140000	-2.241190000	-3.335100000
6	-8.528200000	-4.180100000	-2.560660000
6	-8.223980000	-4.772920000	-3.834920000
6	-8.745060000	-3.658820000	-7.458990000
6	-7.906650000	-2.557480000	-7.545860000
1	-7.819440000	-2.024870000	-8.496910000
6	-7.144050000	-2.118210000	-6.424170000
6	-6.262260000	-0.998940000	-6.520150000
1	-6.213110000	-0.455510000	-7.468290000
6	-5.476440000	-0.618900000	-5.444200000
1	-4.803130000	0.237490000	-5.529470000
6	-5.528100000	-1.357570000	-4.229850000
1	-4.882490000	-1.071590000	-3.395900000
6	-6.380950000	-2.443200000	-4.097600000
1	-6.411860000	-3.013630000	-3.168720000
6	-7.232970000	-2.838270000	-5.173450000
6	-8.139100000	-3.957930000	-5.080770000
6	-7.156620000	-8.308430000	-2.805260000
6	-7.688650000	-9.286200000	-1.933530000
1	-8.499220000	-9.001000000	-1.260030000
6	-7.256450000	-10.618090000	-1.988930000
1	-7.735350000	-11.378570000	-1.366800000

6	-6.237060000	-10.960750000	-2.888000000
6	-5.668540000	-12.272520000	-3.241250000
6	-6.058960000	-13.567540000	-2.872020000
1	-6.873470000	-13.721140000	-2.158960000
6	-5.418000000	-14.663300000	-3.477950000
1	-5.723250000	-15.681280000	-3.219800000
6	-4.403030000	-14.464650000	-4.433560000
1	-3.934330000	-15.328000000	-4.912380000
6	-4.008090000	-13.162340000	-4.801740000
1	-3.237640000	-13.014630000	-5.564140000
6	-4.645370000	-12.069600000	-4.210700000
6	-4.475540000	-10.589080000	-4.497770000
6	-5.634100000	-9.966780000	-3.716920000
6	-6.117720000	-8.658280000	-3.713160000
1	-5.741050000	-7.920550000	-4.421600000
6	-4.238080000	-10.104010000	-5.981170000
1	-3.804800000	-10.923500000	-6.580640000
1	-5.117690000	-9.676040000	-6.470440000
6	-3.134930000	-9.104110000	-5.475710000
1	-2.200920000	-9.062240000	-6.056490000
1	-3.549720000	-8.090640000	-5.362330000
6	-3.082490000	-9.898440000	-4.117600000
1	-3.086930000	-9.324280000	-3.179550000
1	-2.279950000	-10.654440000	-4.115140000
6	-9.489420000	-4.175230000	-8.638900000
6	-10.898350000	-4.126530000	-8.680890000
1	-11.455810000	-3.722620000	-7.835910000
6	-11.591170000	-4.585970000	-9.811860000
1	-12.680990000	-4.509610000	-9.845780000
6	-10.859240000	-5.109460000	-10.888840000

6	-11.298850000	-5.610990000	-12.205830000
6	-12.581790000	-5.763500000	-12.754550000
1	-13.470300000	-5.503230000	-12.172880000
6	-12.701970000	-6.256510000	-14.067480000
1	-13.693620000	-6.379860000	-14.510660000
6	-11.558730000	-6.594760000	-14.816820000
1	-11.672790000	-6.972670000	-15.836190000
6	-10.270150000	-6.460080000	-14.260060000
1	-9.384270000	-6.738980000	-14.838630000
6	-10.142070000	-5.968230000	-12.959300000
6	-8.895180000	-5.813880000	-12.110250000
6	-9.437250000	-5.198520000	-10.828500000
6	-8.751780000	-4.733050000	-9.707290000
1	-7.670860000	-4.842230000	-9.597100000
6	-7.569550000	-5.130630000	-12.644800000
1	-7.402500000	-4.102280000	-12.290190000
1	-7.529540000	-5.174910000	-13.746380000
6	-6.727490000	-6.291720000	-11.985860000
1	-5.921820000	-6.720080000	-12.601440000
1	-6.340780000	-6.048100000	-10.983200000
6	-8.046710000	-7.137360000	-11.873450000
1	-8.203240000	-7.647590000	-10.916830000
1	-8.173980000	-7.818120000	-12.732490000
6	-16.149000000	-12.605450000	-9.726550000
6	-14.810470000	-12.203640000	-9.620550000
6	-14.270710000	-13.538360000	-11.600890000
6	-15.607500000	-13.937930000	-11.696830000
6	-16.549470000	-13.471940000	-10.757640000
1	-16.878320000	-12.246880000	-8.996520000
1	-14.472400000	-11.546940000	-8.820770000

1	-13.533810000	-13.887530000	-12.331860000
1	-15.923010000	-14.604500000	-12.502980000
1	-17.594050000	-13.784090000	-10.835770000
1	-13.744030000	-8.972140000	-9.288290000
1	-13.436830000	-7.214350000	-9.263910000
1	-13.254150000	-8.125780000	-10.791950000
1	-7.559900000	-11.767170000	-8.951630000
1	-8.455960000	-11.793010000	-10.486340000
1	-7.611100000	-10.295710000	-9.954000000
1	-11.283330000	-9.614680000	-13.753210000
1	-12.511290000	-10.089410000	-12.539850000
1	-11.762490000	-8.464850000	-12.488630000
1	-15.090370000	-12.593630000	-4.356690000
1	-16.846560000	-12.506760000	-4.681020000
1	-16.109720000	-14.071190000	-4.244770000
1	-13.625520000	-16.454020000	-4.203100000
1	-12.416820000	-15.523540000	-3.271150000
1	-14.084680000	-14.920100000	-3.390660000
1	-13.195350000	-17.054860000	-8.034880000
1	-13.010890000	-17.418890000	-6.294510000
1	-14.133110000	-18.387390000	-7.294410000
1	-10.686571000	-13.693868000	-9.833858000

Acetaldehyde enolsilane addition (with Catalyst 7d)

Major enantiomer

B3LYP-D3(BJ) Energy: -8280.90798962 hartree;

16	-7.156240000	30.956370000	5.876010000
16	-12.307860000	33.202280000	4.050930000
15	-7.688440000	33.401730000	4.461000000
15	-10.201190000	35.131180000	4.514750000

7	-8.751630000	34.547630000	4.364230000
7	-8.164390000	32.059900000	5.215840000
7	-11.465640000	34.237530000	4.948330000
8	-6.292940000	33.990680000	5.215430000
8	-7.149330000	33.174930000	2.895540000
8	-5.800670000	30.834080000	5.280330000
8	-7.938130000	29.707400000	6.113820000
8	-10.033570000	36.308440000	5.681160000
8	-10.589690000	36.045830000	3.162970000
8	-13.289730000	32.494370000	4.934400000
8	-12.818320000	33.742220000	2.760950000
6	-14.002240000	29.712510000	6.827920000
1	-13.819440000	30.615050000	6.225920000
8	-13.011200000	29.117350000	7.416750000
14	-11.259560000	29.548980000	7.195550000
6	-14.307340000	28.214900000	4.925730000
1	-13.341159000	27.789665000	5.100572000
6	-14.433650000	29.332290000	4.135100000
1	-13.552520000	29.874080000	3.771290000
8	-15.631390000	29.829640000	3.815910000
14	-15.999850000	31.008570000	2.566980000
6	-6.865620000	31.684670000	7.584930000
6	-11.043980000	31.895660000	3.628150000
6	-11.193160000	31.390700000	7.485820000
6	-10.882320000	28.932290000	5.467540000
6	-10.454020000	28.533040000	8.597210000
6	-14.631630000	30.935670000	1.283970000
6	-16.207380000	32.695510000	3.365300000
6	-17.650410000	30.285320000	1.961680000
6	-5.658800000	35.045050000	4.539910000

6	-5.893490000	32.591640000	2.710650000
6	-10.938040000	37.365730000	5.692270000
6	-9.702970000	37.099100000	2.901370000
6	-15.349800000	29.477650000	7.341780000
9	-6.425520000	30.640070000	8.388550000
9	-8.100040000	32.155210000	8.073390000
9	-11.729600000	30.862070000	2.934780000
1	-6.131780000	32.502870000	7.528850000
9	-10.168940000	32.539900000	2.738070000
1	-10.500200000	31.503510000	4.499300000
1	-10.146280000	31.731870000	7.490500000
1	-11.624650000	31.621970000	8.474250000
1	-11.467770000	29.405280000	4.666540000
1	-9.822500000	29.138230000	5.246450000
6	-10.041110000	27.130790000	8.085190000
1	-11.749610000	31.985240000	6.744940000
1	-11.022160000	27.842860000	5.376510000
6	-9.186680000	29.272490000	9.107750000
6	-11.447940000	28.376250000	9.775820000
1	-14.781920000	31.723820000	0.526630000
1	-13.642000000	31.113950000	1.732300000
1	-14.612750000	29.958760000	0.769820000
1	-16.869930000	33.323800000	2.743020000
1	-15.235100000	33.202630000	3.459790000
6	-18.210900000	31.145240000	0.801780000
6	-17.431580000	28.830300000	1.475890000
6	-18.654850000	30.279130000	3.143900000
1	-16.656220000	32.624590000	4.371210000
6	-4.925560000	34.768710000	3.384610000
6	-4.393070000	35.853290000	2.605290000

6	-3.770760000	35.648840000	1.335210000
1	-3.675090000	34.627850000	0.960650000
6	-3.305860000	36.725710000	0.594960000
1	-2.843400000	36.555540000	-0.380280000
6	-3.423110000	38.053690000	1.095680000
1	-3.033300000	38.888970000	0.508420000
6	-4.032730000	38.284190000	2.319130000
1	-4.142760000	39.298450000	2.712030000
6	-4.558900000	37.201180000	3.088690000
6	-5.261570000	37.423760000	4.303860000
1	-5.375600000	38.448350000	4.667340000
6	-5.840380000	36.380000000	5.023220000
6	-4.756060000	33.357440000	2.944810000
6	-3.455730000	32.765050000	2.784420000
6	-2.248510000	33.463470000	3.088360000
1	-2.316080000	34.484290000	3.468820000
6	-1.014860000	32.855970000	2.906270000
1	-0.099460000	33.402650000	3.145910000
6	-0.929470000	31.522170000	2.418150000
1	0.050380000	31.059170000	2.277760000
6	-2.084480000	30.809150000	2.137890000
1	-2.030970000	29.776090000	1.781840000
6	-3.371900000	31.399290000	2.322280000
6	-4.569560000	30.665350000	2.090600000
1	-4.501410000	29.626030000	1.757200000
6	-5.823500000	31.224850000	2.304210000
6	-6.610170000	36.688580000	6.256100000
6	-6.434400000	35.950440000	7.454770000
1	-5.768650000	35.085590000	7.457140000
6	-7.091590000	36.363510000	8.613950000

6	-7.021000000	35.804860000	10.032790000
6	-5.533440000	35.657890000	10.578790000
1	-4.779500000	36.255690000	10.046550000
1	-5.505540000	35.862070000	11.661830000
6	-5.598520000	34.114020000	10.275780000
1	-5.141050000	33.875850000	9.300680000
1	-5.203660000	33.437590000	11.048480000
6	-7.166560000	34.234830000	10.166370000
1	-7.650740000	33.987090000	11.126880000
1	-7.664630000	33.717250000	9.339530000
6	-7.989880000	36.691820000	10.791550000
6	-8.396720000	36.613240000	12.125030000
1	-7.991330000	35.843540000	12.788950000
6	-9.348600000	37.537300000	12.602100000
1	-9.682040000	37.484340000	13.641570000
6	-9.883900000	38.521090000	11.748510000
1	-10.630850000	39.219940000	12.133440000
6	-9.477830000	38.606110000	10.404810000
1	-9.910170000	39.356420000	9.737420000
6	-8.524920000	37.691970000	9.932410000
6	-7.949830000	37.503170000	8.588200000
6	-8.146480000	38.220150000	7.400550000
1	-8.829560000	39.072710000	7.362420000
6	-7.473600000	37.808560000	6.243780000
1	-7.648130000	38.332950000	5.304470000
6	-7.057070000	30.408850000	2.185270000
6	-7.101990000	29.149080000	2.836110000
1	-6.255750000	28.848710000	3.458470000
6	-8.259930000	28.377400000	2.744720000
6	-8.573250000	27.006990000	3.333250000

6	-8.014090000	26.759140000	4.793770000
1	-7.811560000	27.676270000	5.365100000
1	-8.681340000	26.079130000	5.349690000
6	-6.785740000	25.991470000	4.174780000
1	-6.496690000	25.045220000	4.656670000
1	-5.907600000	26.650660000	4.081930000
6	-7.573240000	25.878730000	2.816880000
1	-8.104300000	24.916350000	2.728670000
1	-7.039450000	26.119970000	1.886120000
6	-10.047280000	26.836870000	3.011560000
6	-10.930050000	25.824940000	3.397070000
1	-10.584500000	24.989750000	4.014150000
6	-12.279850000	25.903140000	2.990880000
1	-12.983400000	25.121780000	3.291370000
6	-12.732100000	26.982670000	2.205660000
1	-13.784500000	27.034220000	1.912610000
6	-11.848530000	28.005390000	1.813900000
1	-12.202290000	28.856150000	1.226050000
6	-10.506680000	27.926270000	2.215580000
6	-9.390870000	28.870130000	2.032060000
6	-9.340170000	30.104680000	1.372640000
1	-10.195560000	30.477360000	0.808560000
6	-8.172790000	30.874430000	1.454570000
1	-8.135540000	31.848740000	0.971400000
6	-10.869890000	38.351060000	4.707230000
6	-11.833230000	39.420500000	4.712470000
6	-11.894210000	40.403460000	3.678380000
1	-11.191890000	40.330640000	2.846130000
6	-12.829650000	41.426260000	3.727500000
1	-12.864840000	42.168630000	2.926380000

6	-13.752570000	41.512930000	4.807040000
1	-14.479960000	42.328040000	4.833050000
6	-13.739450000	40.557140000	5.810500000
1	-14.458660000	40.601110000	6.633820000
6	-12.797330000	39.483790000	5.786770000
6	-12.806290000	38.460640000	6.777890000
1	-13.544120000	38.503440000	7.584300000
6	-11.914330000	37.395900000	6.732300000
6	-9.807470000	38.260450000	3.667180000
6	-8.847160000	39.316530000	3.486180000
6	-8.804610000	40.472160000	4.325590000
1	-9.549410000	40.569300000	5.117740000
6	-7.833810000	41.445610000	4.141460000
1	-7.807380000	42.317620000	4.799360000
6	-6.871120000	41.320170000	3.100050000
1	-6.125250000	42.105890000	2.955560000
6	-6.879190000	40.204440000	2.277490000
1	-6.139000000	40.085620000	1.481750000
6	-7.840370000	39.163490000	2.464760000
6	-7.807910000	37.976410000	1.683240000
1	-7.046340000	37.882530000	0.904920000
6	-8.698020000	36.925180000	1.897280000
6	-12.018940000	36.261270000	7.683490000
6	-13.268750000	35.613340000	7.805740000
1	-14.101240000	35.938680000	7.176980000
6	-13.391620000	34.527860000	8.667460000
6	-14.583580000	33.614250000	8.892750000
6	-15.257120000	33.114790000	7.547830000
1	-14.617350000	33.156490000	6.653050000
1	-15.666170000	32.105360000	7.699270000

6	-16.383840000	34.199110000	7.725110000
1	-16.181680000	35.104380000	7.130560000
1	-17.418760000	33.861840000	7.555800000
6	-15.939700000	34.369880000	9.227760000
1	-16.556590000	33.761060000	9.909470000
6	-14.042270000	32.599730000	9.881530000
6	-14.675660000	31.486750000	10.437870000
1	-15.699930000	31.226110000	10.155090000
6	-13.967300000	30.691420000	11.362210000
1	-14.449170000	29.815540000	11.804600000
6	-12.641510000	31.011260000	11.716520000
1	-12.110390000	30.385840000	12.439280000
6	-11.988340000	32.117770000	11.140720000
1	-10.952720000	32.352430000	11.402490000
6	-12.692510000	32.912170000	10.222220000
6	-12.278280000	34.094520000	9.441400000
6	-11.035260000	34.734880000	9.320670000
1	-10.170300000	34.406780000	9.903080000
6	-10.905190000	35.806620000	8.425520000
1	-9.941820000	36.296760000	8.299800000
6	-8.600540000	35.693270000	1.073630000
6	-9.758400000	35.023260000	0.597330000
1	-10.746340000	35.338450000	0.934810000
6	-9.609500000	33.960030000	-0.290900000
6	-10.680280000	33.134910000	-0.994220000
6	-11.812710000	34.009100000	-1.688420000
1	-11.544030000	35.062780000	-1.854160000
1	-12.139180000	33.535330000	-2.629120000
6	-12.780260000	33.678980000	-0.489680000
1	-12.819920000	34.468220000	0.276270000

1	-13.801700000	33.369490000	-0.761990000
6	-11.799070000	32.538440000	-0.040720000
1	-12.114390000	31.541600000	-0.393350000
1	-11.531420000	32.527930000	1.016630000
6	-9.861820000	32.161910000	-1.820460000
6	-10.297480000	31.120450000	-2.642400000
1	-11.366090000	30.937090000	-2.790760000
6	-9.338370000	30.301640000	-3.272610000
1	-9.664700000	29.480140000	-3.915510000
6	-7.962410000	30.525100000	-3.073230000
1	-7.233960000	29.872440000	-3.561130000
6	-7.516090000	31.571230000	-2.245970000
1	-6.447680000	31.733230000	-2.080080000
6	-8.469450000	32.391090000	-1.624630000
6	-8.313570000	33.524020000	-0.693760000
6	-7.165310000	34.147280000	-0.182830000
1	-6.165310000	33.789590000	-0.442810000
6	-7.317160000	35.229190000	0.693220000
1	-6.431810000	35.695830000	1.128350000
1	-15.819280000	35.391820000	9.616510000
6	-15.529540000	28.624740000	8.454460000
6	-16.810100000	28.436490000	8.988010000
6	-17.917850000	29.084830000	8.408990000
6	-17.742860000	29.922930000	7.291050000
6	-16.464170000	30.125570000	6.758040000
1	-14.650230000	28.157610000	8.901720000
1	-16.949120000	27.790640000	9.858320000
1	-18.915920000	28.939250000	8.829680000
1	-18.603460000	30.419990000	6.836910000
1	-16.320810000	30.756650000	5.881560000

1	-9.594320000	26.554780000	8.919340000
1	-10.913460000	26.565470000	7.704050000
1	-9.292200000	27.214040000	7.279240000
1	-9.423180000	30.295180000	9.454200000
1	-8.764970000	28.705050000	9.961390000
1	-8.417340000	29.348210000	8.324340000
1	-10.941750000	27.834990000	10.599970000
1	-11.783800000	29.355330000	10.157820000
1	-12.339880000	27.800220000	9.472350000
1	-17.515970000	31.157330000	-0.059100000
1	-19.175610000	30.723650000	0.457200000
1	-18.390370000	32.188200000	1.124170000
1	-17.024120000	28.207180000	2.292540000
1	-18.396820000	28.396290000	1.149180000
1	-16.732300000	28.794970000	0.619450000
1	-18.850750000	31.305950000	3.506380000
1	-19.616620000	29.840470000	2.812590000
1	-18.262340000	29.673790000	3.981610000
1	-15.175202000	27.770369000	5.366307000

Minor enantiomer

B3LYP-D3(BJ) Energy: -8280.90073913 hartree;

16	-7.435730000	30.895960000	5.602030000
16	-12.453760000	32.525160000	3.400600000
15	-7.976900000	33.273200000	4.062140000
15	-10.646220000	34.607440000	4.080380000
7	-9.127280000	34.317190000	3.782180000
7	-8.428080000	31.967800000	4.894540000
7	-11.723890000	33.482640000	4.492770000
8	-6.678220000	34.032970000	4.826490000

8	-7.330890000	32.968710000	2.553350000
8	-6.034200000	30.837510000	5.109860000
8	-8.194050000	29.634290000	5.822810000
8	-10.548190000	35.716760000	5.324260000
8	-11.337140000	35.541580000	2.887190000
8	-13.541060000	31.773730000	4.103700000
8	-12.818660000	33.178340000	2.116120000
6	-14.122880000	29.696410000	6.179270000
1	-13.595300000	30.391520000	5.514370000
8	-13.409120000	28.852390000	6.902380000
14	-11.620080000	28.980850000	6.744360000
6	-14.710590000	31.202920000	7.674070000
1	-13.835500000	30.985550000	8.250150000
6	-14.736360000	32.318990000	6.840400000
1	-13.811360000	32.851860000	6.574930000
8	-15.862090000	32.718970000	6.284200000
14	-16.242100000	34.160970000	5.334600000
6	-7.221720000	31.625260000	7.342630000
6	-11.092540000	31.289260000	3.061370000
6	-11.191470000	30.768730000	7.105220000
6	-11.268930000	28.396600000	5.001270000
6	-11.057340000	27.799090000	8.112060000
6	-17.229710000	33.473140000	3.893030000
6	-14.714830000	35.103650000	4.816980000
6	-17.313000000	35.130510000	6.572630000
6	-6.154570000	35.123190000	4.115730000
6	-6.003650000	32.550110000	2.451670000
6	-11.603010000	36.550340000	5.640590000
6	-10.736600000	36.788730000	2.699180000
6	-15.460020000	29.280870000	5.748890000

9	-8.172170000	31.065250000	8.224600000
9	-7.469940000	33.003290000	7.259730000
9	-11.667300000	30.258250000	2.307350000
1	-6.200310000	31.421980000	7.697870000
9	-10.193750000	32.021130000	2.269540000
1	-10.604960000	30.922980000	3.976190000
1	-10.100200000	30.851250000	7.203970000
1	-11.607560000	31.104990000	8.069070000
1	-11.810960000	28.952920000	4.219860000
1	-10.192790000	28.498790000	4.782670000
6	-11.463840000	26.348210000	7.747990000
1	-11.505640000	31.488980000	6.331960000
1	-11.528670000	27.333600000	4.864980000
6	-9.516390000	27.877870000	8.279120000
6	-11.757340000	28.216690000	9.430990000
1	-17.740650000	34.289370000	3.352740000
1	-16.559440000	32.962810000	3.180650000
1	-17.991100000	32.754220000	4.238970000
1	-13.925100000	35.103870000	5.584340000
1	-14.981770000	36.163370000	4.655050000
6	-17.809420000	36.441090000	5.908430000
6	-18.522210000	34.269280000	7.013470000
6	-16.452310000	35.484070000	7.810540000
1	-14.280680000	34.719110000	3.881640000
6	-5.345720000	34.876930000	3.005060000
6	-4.949470000	35.971040000	2.159930000
6	-4.245920000	35.776520000	0.932170000
1	-3.936050000	34.764760000	0.663570000
6	-3.972460000	36.849370000	0.095930000
1	-3.445360000	36.682410000	-0.846480000

6	-4.380860000	38.166610000	0.448550000
1	-4.159140000	38.999040000	-0.223740000
6	-5.062050000	38.388780000	1.635010000
1	-5.390190000	39.394420000	1.915800000
6	-5.372360000	37.304820000	2.513170000
6	-6.119050000	37.507380000	3.705590000
1	-6.398340000	38.526960000	3.984870000
6	-6.533580000	36.446870000	4.509530000
6	-4.982850000	33.471950000	2.667300000
6	-3.612920000	33.043070000	2.583900000
6	-2.517000000	33.909820000	2.876390000
1	-2.729740000	34.934760000	3.186270000
6	-1.209660000	33.458260000	2.770250000
1	-0.381270000	34.133210000	2.999380000
6	-0.936440000	32.119430000	2.373430000
1	0.099110000	31.780120000	2.291360000
6	-1.979680000	31.246510000	2.106810000
1	-1.781030000	30.209810000	1.819200000
6	-3.338160000	31.673980000	2.214130000
6	-4.422710000	30.777960000	1.992140000
1	-4.209420000	29.739480000	1.723720000
6	-5.745210000	31.181140000	2.130520000
6	-7.347820000	36.732960000	5.721890000
6	-7.098940000	36.107920000	6.975610000
1	-6.352830000	35.314670000	7.045620000
6	-7.839560000	36.506410000	8.091210000
6	-7.722290000	36.104350000	9.561080000
6	-6.365780000	36.667690000	10.204100000
1	-5.865260000	37.452480000	9.619110000
1	-6.553630000	37.001910000	11.237230000

6	-5.767420000	35.214890000	10.149600000
1	-5.125670000	35.076100000	9.263870000
1	-5.245620000	34.865600000	11.053360000
6	-7.216970000	34.650870000	9.899570000
1	-7.694730000	34.271330000	10.817690000
1	-7.321990000	33.939240000	9.077760000
6	-8.988050000	36.680950000	10.167590000
6	-9.526770000	36.499700000	11.441710000
1	-9.055420000	35.819630000	12.157030000
6	-10.707350000	37.191690000	11.786670000
1	-11.145010000	37.053500000	12.779280000
6	-11.327200000	38.058430000	10.865050000
1	-12.240790000	38.586640000	11.151790000
6	-10.785910000	38.247690000	9.580090000
1	-11.281040000	38.899650000	8.855860000
6	-9.618070000	37.552290000	9.234340000
6	-8.887410000	37.468120000	7.958370000
6	-9.125710000	38.092520000	6.729440000
1	-9.925860000	38.826440000	6.613940000
6	-8.347540000	37.728660000	5.624160000
1	-8.563600000	38.175540000	4.653830000
6	-6.863270000	30.210030000	2.016340000
6	-6.788820000	28.994360000	2.745260000
1	-5.937200000	28.831450000	3.409180000
6	-7.849200000	28.090500000	2.677080000
6	-8.030230000	26.734290000	3.352660000
6	-7.619270000	26.684920000	4.883620000
1	-7.614610000	27.665540000	5.377350000
1	-8.260880000	25.968000000	5.424890000
6	-6.240340000	26.043590000	4.473770000

1	-5.893110000	25.186310000	5.070540000
1	-5.446430000	26.805750000	4.411920000
6	-6.843780000	25.718000000	3.056000000
1	-7.232820000	24.687580000	3.001860000
1	-6.237240000	25.955230000	2.169610000
6	-9.442950000	26.350440000	2.955980000
6	-10.206680000	25.255930000	3.366660000
1	-9.782220000	24.496960000	4.031290000
6	-11.544420000	25.154940000	2.929120000
1	-12.156800000	24.307080000	3.247370000
6	-12.104070000	26.145940000	2.098560000
1	-13.149300000	26.062600000	1.789450000
6	-11.340090000	27.250670000	1.680630000
1	-11.786270000	28.046070000	1.079560000
6	-10.005100000	27.341360000	2.101680000
6	-9.002170000	28.402680000	1.899400000
6	-9.063090000	29.587570000	1.153790000
1	-9.935200000	29.825630000	0.544710000
6	-7.994980000	30.492120000	1.219130000
1	-8.047030000	31.425920000	0.662990000
6	-11.969390000	37.554970000	4.737260000
6	-13.117950000	38.366450000	5.025510000
6	-13.673320000	39.270560000	4.071300000
1	-13.204540000	39.337930000	3.087040000
6	-14.789780000	40.031700000	4.385800000
1	-15.214340000	40.709140000	3.641110000
6	-15.394510000	39.932290000	5.671340000
1	-16.272130000	40.540220000	5.904230000
6	-14.878380000	39.062030000	6.619810000
1	-15.345960000	38.966990000	7.604560000

6	-13.741840000	38.246940000	6.322250000
6	-13.236400000	37.302450000	7.257820000
1	-13.663850000	37.290420000	8.263440000
6	-12.233120000	36.388110000	6.924960000
6	-11.082590000	37.826080000	3.562770000
6	-10.413490000	39.098550000	3.426160000
6	-10.627050000	40.192920000	4.318880000
1	-11.381440000	40.092240000	5.100110000
6	-9.891090000	41.363580000	4.201450000
1	-10.064820000	42.184450000	4.901360000
6	-8.907020000	41.502760000	3.184570000
1	-8.335180000	42.430570000	3.106690000
6	-8.672100000	40.459870000	2.302800000
1	-7.911930000	40.547060000	1.520300000
6	-9.402100000	39.235060000	2.401040000
6	-9.099740000	38.135220000	1.550080000
1	-8.322640000	38.254310000	0.790160000
6	-9.732750000	36.903220000	1.689270000
6	-11.853450000	35.295530000	7.862630000
6	-12.785380000	34.878280000	8.856120000
1	-13.772250000	35.341890000	8.910050000
6	-12.447410000	33.868650000	9.752350000
6	-13.218270000	33.322880000	10.947450000
6	-14.775760000	33.162420000	10.740740000
1	-15.090060000	33.103600000	9.688360000
1	-15.166010000	32.303830000	11.312200000
6	-15.006270000	34.533440000	11.481320000
1	-15.146490000	35.360880000	10.766440000
1	-15.803260000	34.555000000	12.239780000
6	-13.526710000	34.465130000	12.017400000

1	-13.473960000	34.055920000	13.039520000
6	-12.347740000	32.163730000	11.405370000
6	-12.556290000	31.243860000	12.434430000
1	-13.477870000	31.262320000	13.024180000
6	-11.557190000	30.283640000	12.702730000
1	-11.710460000	29.552280000	13.500320000
6	-10.367930000	30.251040000	11.949100000
1	-9.615090000	29.488010000	12.162880000
6	-10.149000000	31.170250000	10.906200000
1	-9.245440000	31.128490000	10.292410000
6	-11.142660000	32.126350000	10.643980000
6	-11.201420000	33.194400000	9.635470000
6	-10.280980000	33.582750000	8.660190000
1	-9.341690000	33.055970000	8.531550000
6	-10.592710000	34.643000000	7.805780000
1	-9.847060000	34.973450000	7.091850000
6	-9.308940000	35.735830000	0.872000000
6	-10.233370000	34.804150000	0.325940000
1	-11.294730000	34.882310000	0.563800000
6	-9.755170000	33.770810000	-0.482640000
6	-10.521340000	32.723560000	-1.291030000
6	-11.657370000	33.358770000	-2.211300000
1	-11.562200000	34.441320000	-2.381600000
1	-11.718490000	32.813710000	-3.167910000
6	-12.740210000	32.866240000	-1.179060000
1	-13.028500000	33.645270000	-0.456280000
1	-13.637460000	32.386240000	-1.598730000
6	-11.662840000	31.921260000	-0.539320000
1	-11.718980000	30.892000000	-0.933950000
1	-11.595740000	31.929370000	0.546300000

6	-9.408280000	31.925430000	-1.943150000
6	-9.494070000	30.783110000	-2.742150000
1	-10.467480000	30.359170000	-3.006880000
6	-8.304900000	30.174690000	-3.192940000
1	-8.357200000	29.275230000	-3.811700000
6	-7.048810000	30.705590000	-2.841260000
1	-6.138040000	30.210590000	-3.187850000
6	-6.954120000	31.856070000	-2.038410000
1	-5.978460000	32.254420000	-1.746690000
6	-8.137770000	32.465080000	-1.594840000
6	-8.355510000	33.629640000	-0.720420000
6	-7.439600000	34.520630000	-0.147020000
1	-6.363060000	34.398320000	-0.288120000
6	-7.922490000	35.572350000	0.637840000
1	-7.215250000	36.242980000	1.127250000
1	-12.898580000	35.361920000	11.912930000
6	-16.070290000	28.141350000	6.316460000
6	-17.356850000	27.770500000	5.910170000
6	-18.033250000	28.529490000	4.934890000
6	-17.412110000	29.648420000	4.349420000
6	-16.124730000	30.029490000	4.750230000
1	-15.509990000	27.562850000	7.054380000
1	-17.835190000	26.888510000	6.343150000
1	-19.038160000	28.237160000	4.619330000
1	-17.928130000	30.217150000	3.571830000
1	-15.597340000	30.863100000	4.281710000
1	-11.162500000	25.665350000	8.566810000
1	-12.558950000	26.268250000	7.612800000
1	-10.967410000	26.015980000	6.817000000
1	-9.165160000	28.908800000	8.456970000

1	-9.213400000	27.244130000	9.136600000
1	-9.001110000	27.511800000	7.373640000
1	-11.467110000	27.517820000	10.240580000
1	-11.457780000	29.231350000	9.744240000
1	-12.856700000	28.186030000	9.316920000
1	-18.402200000	36.232590000	4.997720000
1	-18.460170000	36.987230000	6.619970000
1	-16.966000000	37.105710000	5.643700000
1	-18.180240000	33.321200000	7.466730000
1	-19.120560000	34.825040000	7.761910000
1	-19.177470000	34.032670000	6.154740000
1	-15.570760000	36.081530000	7.514230000
1	-17.053990000	36.075600000	8.529210000
1	-16.105470000	34.569250000	8.325110000
1	-15.565890000	30.563190000	7.738210000

References

[1] Kaib, P. S. J.; Schreyer, L.; Lee, S.; Properzi, R.; List, B. *Angew. Chem. Int. Ed*. **2016**, *55*, 13200–13203.
[2] Gatzenmeier, T.; Turberg, M.; Yepes, D.; Xie, Y.; Neese, F.; Bistoni, G.; List, B. *J. Am. Chem. Soc.* **2018**, *140*, 12671–12676.
[3] Schreyer, L.; Kaib, P. S. J.; Wakchaure, V. N.; Obradors, C.; Properzi, R.; Lee, S.; List, B. *Science* **2018**, *362*, 216–219.
[4] Godfrey, N. A.; Schatz, D. J.; Pronin, S. V. *J. Am. Chem. Soc.* **2018**, *140*, 12770–12774.
[5] Poock, C.; Kalesse, M. *Org. Lett.* **2017**, *19*, 4536–4539.
[6] Baker, R.; Castro, J. L. *J. Chem. Soc., Perkin Trans. 1*, **1990**, 47-65
[7] Boxer, M. B.; Yamamoto, H. *J. Am. Chem. Soc.* **2006**, *128*, 48–49
[8] Brady, P. B.; Yamamoto, H. *Angew. Chem. Int. Ed*. **2012**, *124*, 1978–1982.
[9] Evans, D. A.; Rieger, D. L.; Gage, J. R. *Tetrahedron Lett*. **1990**, *31*, 7099−7100.
[10] Su, C.; Williard, P. G. *Org. Lett.* **2010**, *12*, 5378 – 5381.
[11] Nielsen, C. D. T.; Burés, J. *Chem. Sci.* **2019**, *10*, 348–353
[12] Burés, J. *Angew. Chem Int. Ed.* **2016**, *55*, 2028–2031
[13] Burés, J. *Angew. Chem. Int. Ed*. **2016**, *55*, 16084–16087
[14] Martínez-Carrión, A.; Howlett, M. G.; Alamillo-Ferrer, C.; Clayton, A. D.; Bourne, R. A.; Codina, A.; Vidal-Ferran, A.; Adams, R. W.; Bures, J. *Angew. Chem. Int. Ed.* 2**019**, *58*, 10189-101931.
[15] Yakura,T.; Yoshimoto, Y.; Ishida, C.; Mabuchi, S. *Synlett*, **2006**, 930 – 932.
[16] Crimmins, M.T.; Azman, A. M. *Synlett* **2012**, *23*, 1489–1492.
[17] Lin, L.; Yamamoto, K.; Mitsunuma, H.; Kanzaki, Y.; Matsunaga, S.; Kanai, M. *J. Am. Chem. Soc.* **2015**, *137*, 15418–15421.
[18] Curti, C.; Sartori, A.; Battistini, L.; Brindani, N.; Rassu, G.; Pelosi, G.; Lodola, A.; Mor, M.; Casiraghi, G.; Zanardi, F. *Chem. Eur. J*. **2015**, *21*, 6433 – 6442.
[19] Chen, J. L.-Y.; Scott, H. K.; Hesse, M. J.; Willis, C. L.; Aggarwal, V. K. *J. Am. Chem. Soc*. **2013**, *135*, 5316−5319.
[20] Maeda, S.; Harabuchi, Y.; Takagi, M.; Taketsugu, T.; Morokuma, K. *Chem. Rec.* **2016**, *16*, 2232−2248.
[21] Maeda, S.; Ohno, K.; Morokuma, K. *Phys. Chem. Chem. Phys.* **2013**, *15*, 3683
[22] Grimme, S.; Bannwarth, C.; Shushkov, P. *J. Chem. Theory Comput.* **2017**, *13*, 1989−2009
[23] Chung, L. W.; Sameera, W. M. C.; Ramozzi, R.; Page, A. J.; Hatanaka, M.; Petrova, G. P.; Harris, T. V.; Li, X.; Ke, Z.; Liu, F.; Li, H.-B.; Ding, L.; Morokuma, K., *Chem. Rev.* **2015,** ***115***, 5678-5796.
[24] Gaussian 16, Revision A.03, M. J. Frisch, G. W. Trucks, H. B. Schlegel, G. E. Scuseria, M. A. Robb, J. R. Cheeseman, G. Scalmani, V. Barone, G. A. Petersson, H. Nakatsuji, X. Li, M. Caricato, A. V. Marenich, J. Bloino, B. G. Janesko, R. Gomperts, B. Mennucci, H. P. Hratchian, J. V. Ortiz, A. F. Izmaylov, J. L. Sonnenberg, D. Williams-Young, F. Ding, F. Lipparini, F. Egidi, J. Goings, B. Peng, A. Petrone, T. Henderson, D. Ranasinghe, V. G. Zakrzewski, J. Gao, N. Rega, G. Zheng, W. Liang, M. Hada, M. Ehara, K. Toyota, R. Fukuda, J. Hasegawa, M. Ishida, T. Nakajima, Y. Honda, O. Kitao, H. Nakai, T. Vreven, K. Throssell, J. A. Jr. Montgomery, J. E. Peralta, F. Ogliaro, M. J. Bearpark, J. J. Heyd, E. N. Brothers, K. N. Kudin, V. N. Staroverov, T. A. Keith, R. Kobayashi, J. Normand, K. Raghavachari, A. P. Rendell, J. C. Burant, S. S. Iyengar, J. Tomasi, M. Cossi, J. M. Millam, M. Klene, C. Adamo, R. Cammi, J. W. Ochterski, R. L. Martin, K. Morokuma, O. Farkas, J. B. Foresman, D. J. Fox, Gaussian, Inc., Wallingford CT, **2016**.
[25] Neese, F. *WIREs Comput. Mol. Sci.* **2012**, *2*, 73−78.
[26] Becke, A. D. *J. Chem. Phys.* **1993***, 98*, 5648-5652.
[27] Lee, C.; Yang, W.; Parr, R. G., *Phys. Rev. B* **1988***, 37*, 785-789.
[28] Grimme, S.; Antony, J.; Ehrlich, S.; Krieg, H. *J. Chem. Phys.* **2010**, *132*, 154104.
[29] Weigend, F.; Ahlrichs, R. *Phys. Chem. Chem. Phys.* **2005**, *7*, 3297−3305.
[30] Neese, F.; Wennmohs, F.; Hansen, A.; Becker, U. *Chem. Phys*. **2009**, *356*, 98.
[31] Cossi, M.; Rega, N.; Scalmani, G.; Barone, V. J. *Comput. Chem.* **2003**, *24*, 669.
[32] Bickelhaupt, F. M.; Houk, K. N.,. *Angew. Chem. Int. Ed*. **2017***, 56*, 10070-10086.
[33] Maji, R.; Champagne, P. A.; Houk, K. N.; Wheeler, S. E., *ACS Catal*. **2017***, 7*, 7332-7339.
[34] Pettersen, E. F.; Goddard, T. D.; Huang, C. C.; Couch, G. S.; Greenblatt, D. M.; Meng, E. C.; Ferrin, T. E., Pettersen, E. F.; Goddard, T. D.; Huang, C. C.; Couch, G. S.; Greenblatt, D. M.; Meng, E. C.; Ferrin, T. E. *J. Comput. Chem.* **2004**, *25*, 1605−1612.
[35] Contreras-García, J.; Johnson, E. R.; Keinan, S.; Chaudret, R.; Piquemal, J.-P.; Beratan, D. N.; Yang, W. *J. Chem. Theory Comput.* **2011**, *7*, 625−632.
[36] Humphrey, W.; Dalke, A.; Schulten, K. *J. Mol. Graphics* **1996**, *14*, 33-38.
[37] Grimme, S.; Ehrlich, S.; Goerigk, L. *J. Comput. Chem.* **2011**, *32*, 1456−1465.
